# PDBx/mmCIF Ecosystem: Foundational Semantic Tools for Structural Biology

**DOI:** 10.1016/j.jmb.2022.167599

**Published:** 2022-04-20

**Authors:** John D. Westbrook, Jasmine Y. Young, Chenghua Shao, Zukang Feng, Vladimir Guranovic, Catherine L. Lawson, Brinda Vallat, Paul D. Adams, John M Berrisford, Gerard Bricogne, Kay Diederichs, Robbie P. Joosten, Peter Keller, Nigel W. Moriarty, Oleg V. Sobolev, Sameer Velankar, Clemens Vonrhein, David G. Waterman, Genji Kurisu, Helen M. Berman, Stephen K. Burley, Ezra Peisach

**Affiliations:** 1- Research Collaboratory for Structural Bioinformatics Protein Data Bank, Rutgers, The State University of New Jersey, Piscataway, NJ 08854, USA; 2- Institute for Quantitative Biomedicine, Rutgers, The State University of New Jersey, Piscataway, NJ 08854, USA; 3- Cancer Institute of New Jersey, Rutgers, The State University of New Jersey, New Brunswick, NJ 08901, USA; 4- Molecular Biophysics and Integrated Bioimaging, Lawrence Berkeley National Laboratory, Berkeley, CA 94720, USA; 5- Department of Bioengineering, University of California at Berkeley, Berkeley, CA 94720, USA; 6- Protein Data Bank in Europe, European Molecular Biology Laboratory, European Bioinformatics Institute (EMBL-EBI), Wellcome Genome Campus, Hinxton, Cambridge CB10 1SD, UK; 7- Global Phasing Ltd, Sheraton House, Castle Park, Cambridge CB3 0AK, UK; 8- University of Konstanz, 78457 Konstanz, Germany; 9- Department of Biochemistry, Netherlands Cancer Institute, Amsterdam, the Netherlands; 10- Oncode Institute, 3521 AL Utrecht, the Netherlands; 11- UKRI-STFC Rutherford Appleton Laboratory, Didcot OX11 0FA, UK; 12- CCP4, Research Complex at Harwell, Rutherford Appleton Laboratory, Didcot OX11 0FA, UK; 13- Protein Data Bank Japan, Institute for Protein Research, Osaka University, Suita, Osaka 565-0871, Japan; 14- Department of Chemistry and Chemical Biology, Rutgers, The State University of New Jersey, Piscataway, NJ 08854, USA; 15- The Bridge Institute, Michelson Center for Convergent Bioscience, University of Southern California, Los Angeles, CA, USA; 16- Research Collaboratory for Structural Bioinformatics Protein Data Bank, San Diego Supercomputer Center, University of California, La Jolla, CA 92093, USA

**Keywords:** data standard, protein data bank (PDB), biological data, data management, macromolecular structure

## Abstract

PDBx/mmCIF, Protein Data Bank Exchange (PDBx) macromolecular Crystallographic Information Framework (mmCIF), has become the data standard for structural biology. With its early roots in the domain of small-molecule crystallography, PDBx/mmCIF provides an extensible data representation that is used for deposition, archiving, remediation, and public dissemination of experimentally determined three-dimensional (3D) structures of biological macromolecules by the Worldwide Protein Data Bank (wwPDB, wwpdb.org). Extensions of PDBx/mmCIF are similarly used for computed structure models by ModelArc-hive (modelarchive.org), integrative/hybrid structures by PDB-Dev (pdb-dev.wwpdb.org), small angle scattering data by Small Angle Scattering Biological Data Bank SASBDB (sasbdb.org), and for models computed generated with the AlphaFold 2.0 deep learning software suite (alphafold.ebi.ac.uk). Community-driven development of PDBx/mmCIF spans three decades, involving contributions from researchers, software and methods developers in structural sciences, data repository providers, scientific publishers, and professional societies. Having a semantically rich and extensible data framework for representing a wide range of structural biology experimental and computational results, combined with expertly curated 3D biostructure data sets in public repositories, accelerates the pace of scientific discovery. Herein, we describe the architecture of the PDBx/mmCIF data standard, tools used to maintain representations of the data standard, governance, and processes by which data content standards are extended, plus community tools/software libraries available for processing and checking the integrity of PDBx/mmCIF data. Use cases exemplify how the members of the Worldwide Protein Data Bank have used PDBx/mmCIF as the foundation for its pipeline for delivering Findable, Accessible, Interoperable, and Reusable (FAIR) data to many millions of users worldwide.

## Introduction

The Protein Data Bank (PDB)^1^ was established in 1971 as the first open-access digital data repository in biology. It has evolved to become the global archive of three-dimensional (3D) macromolecular structures determined using Macromolecular Crystallography (MX), Nuclear Magnetic Resonance (NMR), and 3D Electron Microscopy (3DEM) methods, for proteins and nucleic acids and their complexes with one another and with small molecule ligands (*e.g*., enzyme co-factors, substrates and substrate analogues, approved and experimental therapeutic agents, and other classes of compounds). During its first 50 years of operation, the archive grew in size from just seven MX protein structures to >188,000 structures (or entries). The PDB Core Archive is managed by the Worldwide PDB organization,^2,3^ consisting of the US-funded Research Collaboratory for Structural Bioinformatics Protein Data Bank (RCSB PDB, rcsb.org),^4,5^ Protein Data Bank in Europe (PDBe, pdbe.org),^6^ Protein Data Bank Japan (PDBj, pdbj.org),^7^ Electron Microscopy Data Bank (EMDB, www.ebi.ac. uk/emdb),^8^ and Biological Magnetic Resonance Bank (BMRB, bmrb.io).^9^ Within the wwPDB, RCSB PDB serves as the Archive Keeper, responsible for safe-guarding and distributing PDB data. wwPDB data centers operated by RCSB PDB, PDBe, and PDBj process new structure depositions, carry out archive-wide remediation activities, and mirror the wwPDB ftp archive, while maintaining distinct web portals for open access to identical archival information with no limitations on usage. The wwPDB has been certified by the CoreTrustSeal (coretrustseal. org). Central to the integrity of the archive is the use of PDBx/mmCIF, a standardized data representation that enables sharing of data in a machine-parsable and extensible format.

For nearly 30 years, PDB depositions and archive files were generated and distributed in what is commonly referred to as the “legacy PDB file format”,^10,11^ a strictly templated format with fixed column widths and positions (www.wwpdb.org/documentation/file-formats-and-the-pdb) based origi nally on 12-row/80-column Hollerith/IBM punched cards. Metadata were stored in REMARK or other keyworded records and atomic coordinate data in ATOM/HETATM records, adhering to specific templates. The uniformity of the atomic coordinate section made the legacy PDB file format a commonly accepted format for many molecular graphics viewers and software packages over the years, including BioJava,^12^ BioPython,^13^ cctbx,^14^ CCP4,^15^ VMD,^16^ Coot,^17^ PyMOL,^18^ SHELX,^19^ Chimera,^20^ Jmol,^21^ and Mol*.^22^

In 1990, the Crystallographic Information Framework (CIF) was adopted by the International Union of Crystallography (IUCr) as a series of exchange protocols based on dictionaries for small-molecule crystallography.^23^ It was based on the concept that all data (values) appear in an ASCII text file with dictionary-controlled labels (keys). The framework describing such a dictionary is regulated by a Dictionary Definition Language (DDL), a generic language that supports construction of dictionaries made up of data items grouped together in categories. The DDL supports primary data types (integers, real numbers, and text), boundary conditions, controlled vocabularies, and the ability to link data items together to express relationships (*e.g*., parent–child related data items). The DDL may be described by its own dictionary and is, therefore, self-validating.

**Figure 1.**
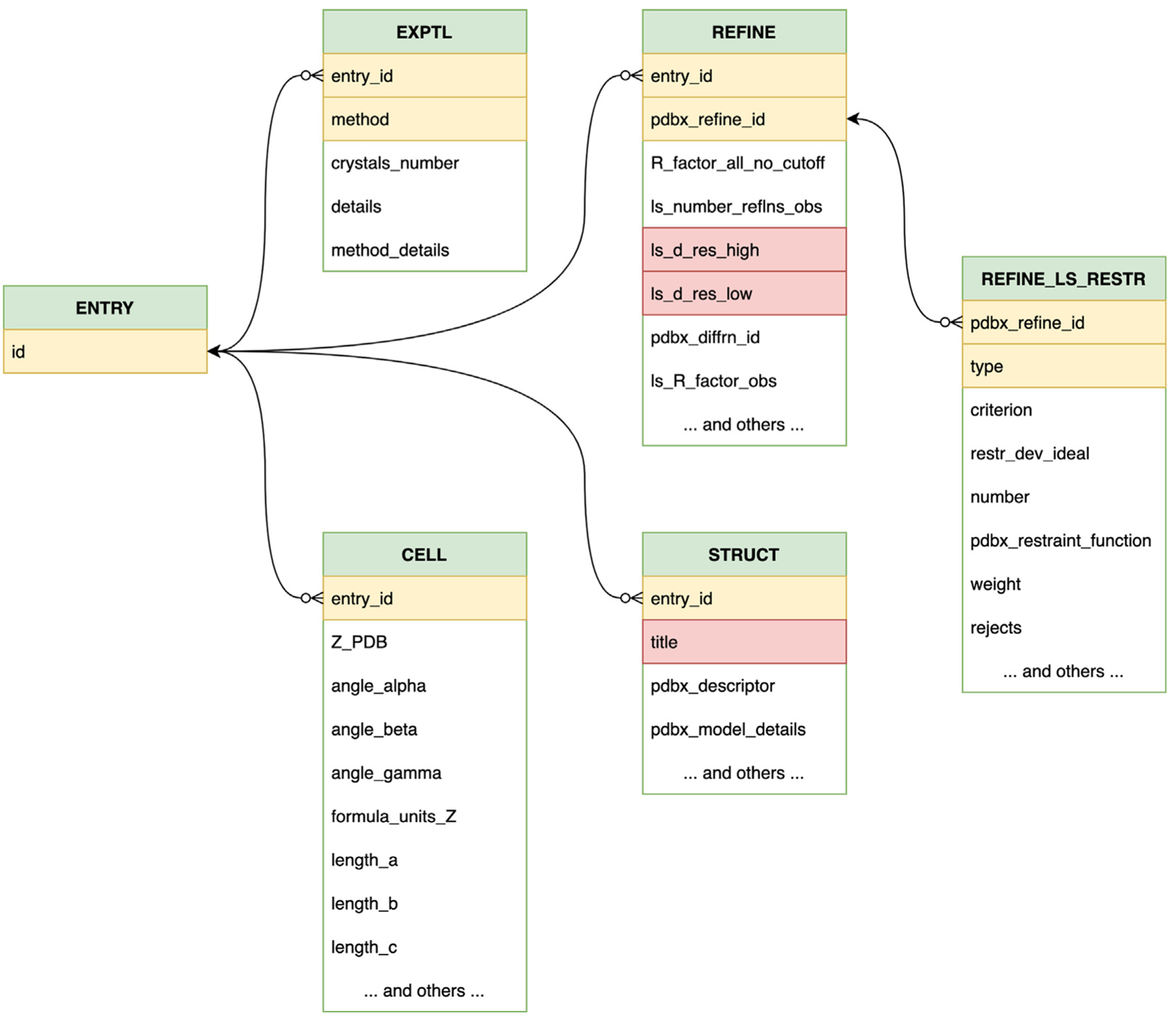
A partial schematic of the parent/child relationships between categories. Color coding: category names (green); mandatory primary category keys (yellow); additional mandatory items (red). Arrows depict child items pointing towards their parent.

In 1990, the IUCr created a working group to expand the CIF dictionary to include data items relevant for capturing the results of macromolecular crystallographic experiments. This working group, chaired by Paula Fitzgerald also included Enrique Abola, Helen M. Berman, Phil Bourne, Eleanor Dodson, Art Olson, Wolfgang Steigemann, Lynn Ten Eyck, and Keith Watenpaugh. Through a series of international meetings, by 1993 it was decided that a new DDL (DDL2) would be needed with stronger linkages between related items with parent/child relationships and the organization of data categories.^24^ This refinement became a part of a doctoral thesis research project carried out by John D. Westbrook and eventually became the second- generation DDL2.^25^ In 1997, the macromolecular mmCIF dictionary (mmcif.wwpdb.org/dictionaries/mmcif_std.dic/Index)^26,27^ was approved by the international Committee for the Maintenance of the CIF Standard (COMCIFS). While based on the original IUCr CIF-core dictionary, the mmCIF dictionary, utilizing DDL2, expanded data categories and attributes to reflect the complexity of macro-molecular structure studies, including support for protein and nucleic acid polymer types, polymer chains, ligands, binding sites, macromolecular assemblies, amino acid and nucleotide residues, atomic coordinates, and experimental data. The layout of DDL2 based dictionaries lends itself to representation as a relational database, with categories stored as tables and the linked items stored as foreign keys (exemplified for the PDB in Figure 1). Once the mmCIF dictionary was extended with a “*pdbx_*” namespace, and adopted as the PDB data exchange format (PDB exchange or PDBx), the name PDBx/mmCIF was adopted (mmcif.org/dictionaries/mmcif_pdbx_v50.dic/Index).^28^

**Figure 2.**
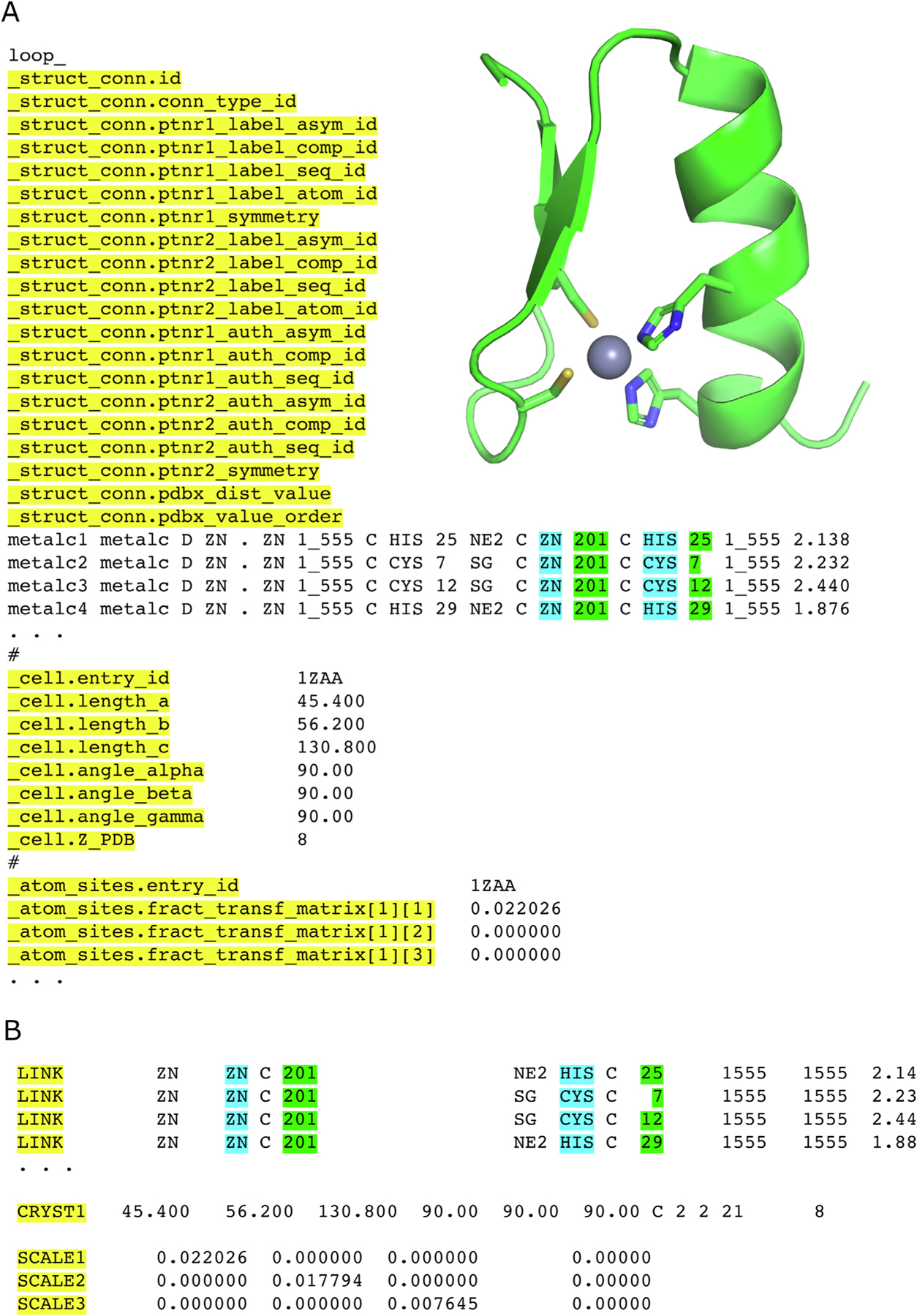
(A) Partial PDBx/mmCIF file for PDB ID 1ZAA.^31^ N.B.: Every data value has a key and multiple rows of data may be described in a table. The yellow highlighting describes the category and attributes. For the _struct_conn category, green depicts the residue numbers and cyan the component type. (B) Equivalent metadata records in legacy PDB format. Similar color coding depicts the mapping between category keys and record names as in (A), with LINK records highlighting the residue number and cyan the chemical component type. Inset figure, one of the zinc finger domains in 1ZAA depicting the sidechains that interact with the bound zinc ion codified in (A) and (B).

PDBx/mmCIF overcame serious shortcomings in the templated legacy PDB file format, allowing facile expansion as structural biology evolved as a scientific discipline. In 2014, PDBx/mmCIF became the master format for the PDB,^29^ addressing the legacy PDB file format limitations. Chief among them were hard limits of 62 polymer chains and 99,999 atomic coordinate x,y,z values that could be stored in legacy PDB files. In 2008, an interim solution was introduced by splitting larger 3D structures into multiple PDB files (entailing the inconvenient use of multiple PDB IDs). In 2014, with ever increasing numbers of larger entries and deposition of atomic level structures of the entire HIV-1 capsid (PDB ID 3j3q and 3j3y,^30^ which would have required division into 25 PDB files with 25 distinct PDB IDs) it became apparent that change could no longer be forestalled. In coordination with the wwPDB mmCIF Working Group (see below), wwPDB leadership adopted PDBx/mmCIF files as the official master archival format of the PDB Core Archive. Figure 2 illustrates a partial mapping between the legacy PDB and PDBx/mmCIF file for mats with a canonical zinc finger domain structure from PDB ID 1ZAA.^31^

Today, legacy PDB formatted files are only produced on a best-effort basis,^29^ with ~2650 of nearly 188,000 PDB structures currently incompatible with the legacy PDB file format. Although~1.5% of the PDB archive not being compatible with the legacy file format may not appear at first glance to be significant, many of these ~2650 PDB structures are among the most interesting data represented in the archive. A majority are the product of the electron microscopy “resolution revolution”,^32^ which has cleared the way to studying a host of new structure determination targets previously inaccessible by either MX or NMR.

During the transition to PDBx/mmCIF as the master archive format, wwPDB members worked with structural biology software developers to ensure that PDBx/mmCIF files would be easy to both generate and use. PyMOL,^18^ CCP4,^15^ Jmol,^21^ and Chimera^20^ developers all embraced the PDBx/mmCIF data standard. Software PDBx/mmCIF parsers are discussed in the CIF Parsers section below and a list may be found on the mmcif.wwpdb.org website.

PDBx/mmCIF formatted files are now used in all aspects of the data processing pipeline for the global wwPDB OneDep deposition, validation, and biocuration system^33^ (hereafter OneDep system). The PDBx/mmCIF file format is the only format accepted for deposition of MX structures.^34^ Atomic coordinates and other data files are stored in PDBx/mmCIF. Intermediate annotation data, which are incorporated into atomic coordinate files during the wwPDB biocuration process, are stored internally in the same format. To facilitate use of the PDBx/mmCIF dictionary to drive more of the wwPDB software stack, DDL2 extensions and additions to PDBx/mmCIF dictionary have been introduced to support enhanced data deposition and processing functionalities, including deposition-specific advisory ranges (*i.e*., soft *versus* hard limits for data items), descriptions, and vocabulary terms plus the ability to define data that are internal to the PDB data pipeline.

In addition to the model files, the PDB distributes other data files in PDBx/mmCIF format. These include the Chemical Component Dictionary (CCD),^35^ the Biologically Interesting molecule Reference Dictionary (BIRD),^36^ and MX experimental diffraction data files.

Since 2003, wwPDB partners and the structural biology community have continued developing tools for working with data in PDBx/mmCIF format (mmcif.wwpdb.org/docs/software-resources.html). These tools center around the dictionary. Their use is discussed below.

## Methods

### CIF parsers

Central to the use of PDBx/mmCIF as the master file format for the PDB Core Archive are efficient parsers and writers of dictionaries and mmCIF formatted files. Syntactically, PDBx/mmCIF follows the CIF 1.1 specification (www.iucr.org/resources/cif/spec/version1.1). Early in development of the dictionary, RCSB PDB developed a set of core C++ libraries for management of PDBx/mmCIF based files (CIFPARSE_OBJ).^37^ Subsequently, Python bindings were added to these libraries. Use of Python based parsers and writers enabled rapid development of the OneDep system. The Python bindings were originally written with Boost,^38^ but with the move to Python3, the pybind11 library^39^ was used. The publicly available “mmcif” package, combines the C++ parser with Python bindings, is available at PyPi (pypi.org/project/mmcif) and GitHub (github.com/rcsb/py-mmcif), and provides an API for accessing PDBx/mmCIF files and dictionaries. This core functionality is used throughout the wwPDB in managing and modifying PDBx/mmCIF files.

There are now a number of different PDBx/mmCIF parsers available in different programming languages, including JAVA (BioJava^12^), PERL (COD::CIF::Parser^40^), Python (BioPython^13^), and C++ (the MMDB library,^41^ the GEMMI library (Wojdyr, M. github.com/project-gemmi/gemmi), and the libcif++ library (Hekkelman, M.L. github.com/PDB-REDO/libcifpp)). A benchmark comparison of various Python based parsers may be found at github.-com/project-gemmi/mmcif-benchmark.

### Dictionary-related tools

While there are numerous tools for parsing and writing CIF formatted files and checking for syntactical errors, there are relatively few tools available for dictionary-based checking of file content (*e.g*., required data items, range limits, controlled vocabulary, enumerations, data types, and proper linking between related data items). Some examples that were developed for the IUCr CIF core standard include checkCIF^42^ and vcif.^43^ However, such tools were usually limited to DDL1 and had built in knowledge of the dictionary. Several examples of tools centered around DDL2 include vcif2,^44^ and cif-validate (Hekkelman, M.L. github.-com/PDB-REDO/cif-tools).

To enable support of DDL2 and the rapidly-evolving PDBx/mmCIF dictionary, the mmCIF Dictionary Suite was developed (sw-tools.rcsb.org/apps/MMCIF-DICT-SUITE/index.html,github.com/rcsb/cpp-dict-pack) to meet the needs of wwPDB partners. Originally developed at RCSB PDB and using the same mmCIF C++ parser as found in the “mmcif” package, the suite provides a series of tools for validating dictionaries, validating files against the dictionary, and other useful utilities to transform files. This software suite can be compiled on all modern Unix-based operating systems.

**Figure 3.**
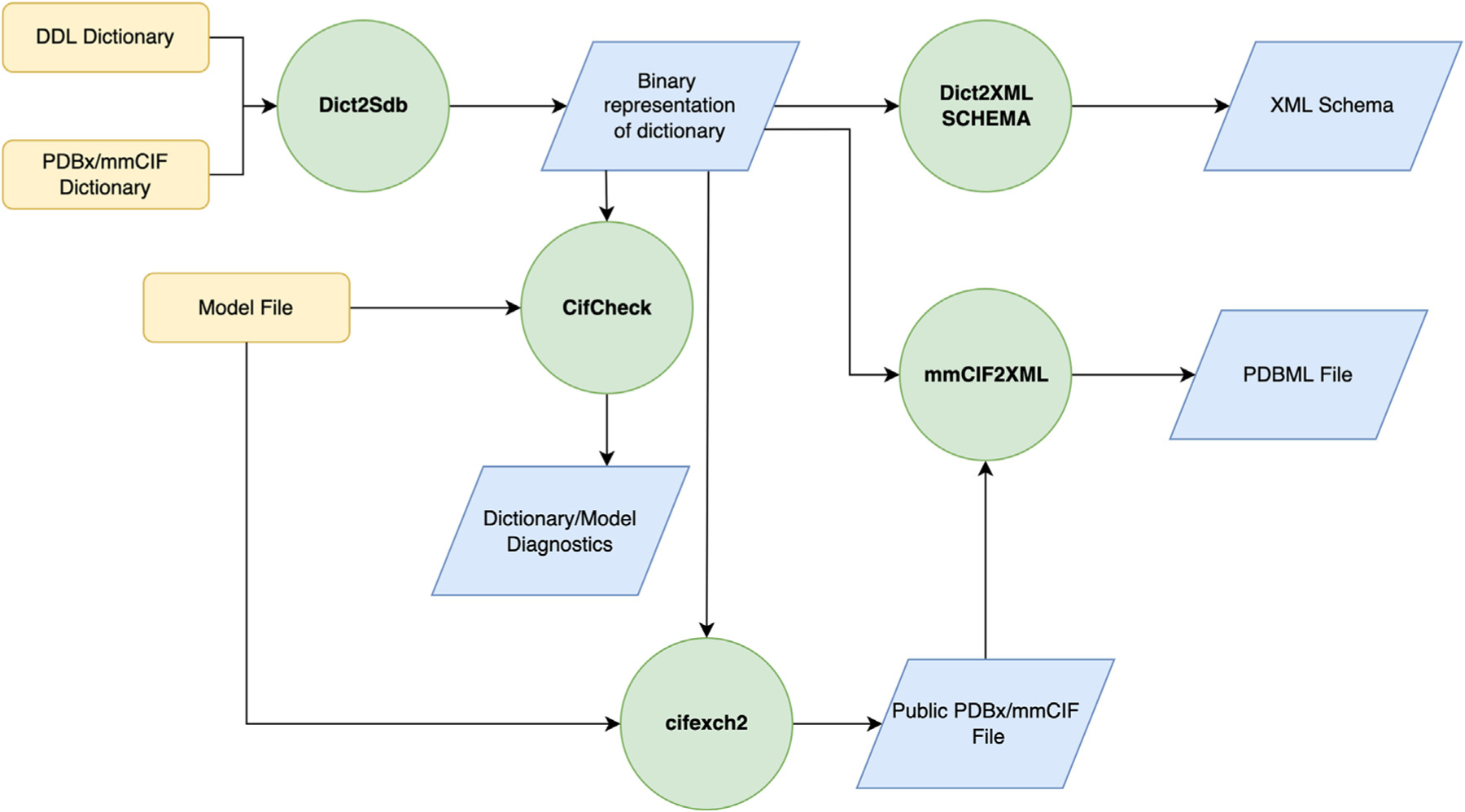
Process flow diagram for checking dictionaries and model files. The DDL dictionary is validated by a builtin minimal DDL2 specification (Dict2Sdb) and then the PDBx/mmCIF is validated against the latest DDL2 dictionary, producing a binary representation. This representation can be used to validate a PDBx/mmCIF based file or converted to a PDBML schema. The model file (containing the atomic coordinates) combined with the internal PDBx/mmCIF dictionary can also be converted to a public model file, which in turn can be converted to a PDBML file. Color coding: process inputs (yellow), programs run (named in green circles), and resulting output (blue tetrahedrons).

As outlined in Figure 3, tools available in the mmCIF Dictionary suite enforce the DDL2 specification.^37^ Two software programs are provided:

- (a) **Dict2Sdb:** Validates a dictionary against the DDL and creates an efficient binary representation. This software tool ensures that the dictionary is both syntactically and semantically correct. Such checks ascertain (i) internal compatibility between parent and child data types; (ii) that the keys for a category are flagged as mandatory; and (iii) that DDL2-required attributes are present for every definition; and (iv) allowed values in enumerations are compatible with the data type.
- (b) **CifCheck:** Using a binary representation of a dictionary and a PDBx/mmCIF file as inputs, this program validates the file against the dictionary. Data type checking, adherence to controlled vocabularies, and checking for ranges, mandatory data items, and parent/child relationships are performed.

The mmCIF Dictionary suite also provides tools for transforming both the dictionary and the data to alternative representations, including PDBML (schema and XML files)^45^ and HTML. The program **Dict2XMLSchema** creates an XML schema from the dictionary, and **mmcif2XML** translates a PDBx/mmCIF file to PDBML format (pdbml.wwpdb.org). In addition, **cifexch2** is used to filter out categories or items that are used within the wwPDB for data processing, have been deprecated, or are not used. Control of such filtering is encoded within the dictionary itself, necessitating fewer software updates.

### Database loader tool

The PDBx/mmCIF dictionary can be expressed as a series of tables with parent/child relationships between individual data items (Figure 1). This feature makes it similar to a relational database within which categories are stored as tables with the relationships represented as foreign keys. The types of columns in a table can be expressed as database primitives, such as integers, floating point numbers, strings, and dates.

To support this capability, RCSB PDB developed a database loader package (sw-tools.rcsb.org/apps/DB-LOADER/index.html).^37^ The **db-loader** program provides an all-in-one tool based around a mapping file describing how a mmCIF file would be converted to a database, including column widths, types, mandatory keys, and item aliasing. Specifically, the program allows for (i) creation of a mapping file from a dictionary; (ii) creation of a database schema from a mapping file definition; and (iii) use of a mapping file and a list of PDBx/mmCIF files to produce a series of commands for rapid loading of data into a database. Supported databases include MySQL, Sybase, Oracle, and Db2.

**Db-loader** is used to load PDBx/mmCIF formatted atomic coordinates, CCD components, and BIRD files into databases to provide support for rapid data searching. For example, by loading the list of chemical components from each structure into a central database, wwPDB can track usage of chemical components in both publicly released structures and in-process unreleased structures. This feature enables identification of relevant PDB structures that might require remediation if a CCD component is updated. This tool is used extensively within the OneDep system and is central to exchanging data between wwPDB partner sites, supporting weekly release of updated PDB and EMDB wwPDB Core Archives. (N.B.: Each week at 00:00 Universal Time on Wednesdays, ~250 new PDB structures and ~85 new EMDB 3DEM density maps are released to the public).

### mmCIF website

**Figure 4.**
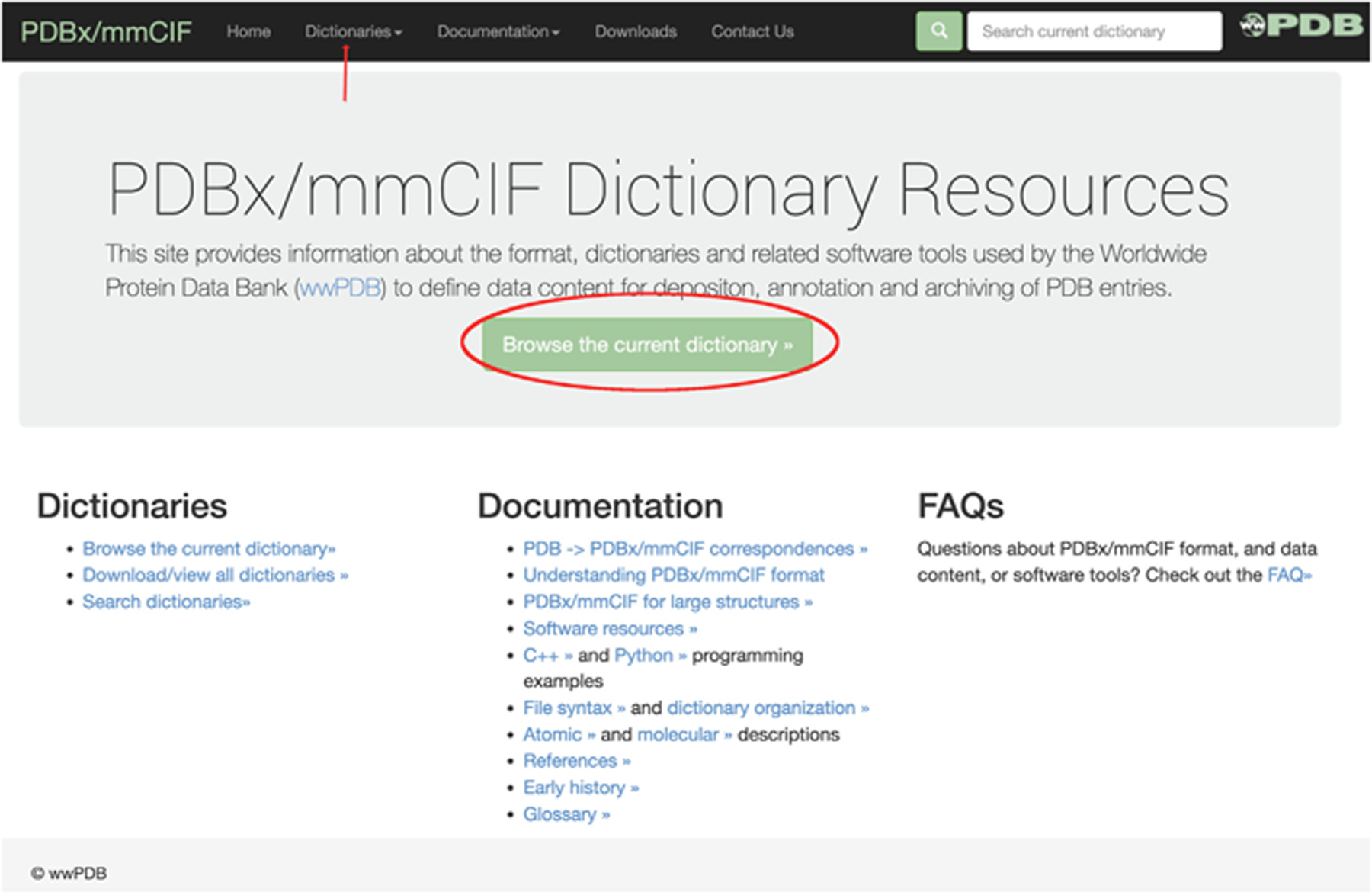
mmcif.wwpdb.org website homepage. Pressing the button highlighted with the red ellipse provides access to the latest PDBx/mmCIF library. Optionally, one can access other dictionaries through the pulldown menus at the end of the red arrow.

The mmCIF website (mmcif.wwpdb.org) is a one-stop shop for information on PDBx/mmCIF (Figure 4). This portal provides open access to CIF-related resources, including detailed descriptions of data definitions, graphical display of dictionaries, PDB legacy format to PDBx/mmCIF mappings, PDBx/mmCIF tutorials, and software resources utilizing PDBx/mmCIF. Several other dictionaries are available on this same website, including DDL2, the original mmCIF dictionary, and older versions of PDBx/mmCIF. Several extension dictionaries are also present. These include those created for representing diffraction image data (imgCIF),^46^ for archiving computed structure models in the ModelArchive,^47^ and integrative/hybrid methods (I/HM) structures archived in PDB-Dev.^48,49^ The mmCIF website supports searching and browsing dictionaries. The search functionality enables identification of specific data items or navigation through different dictionary categories. Relationships between categories can also be displayed using parent/child diagrams (similar to Figure 1).

**Figure 5.**
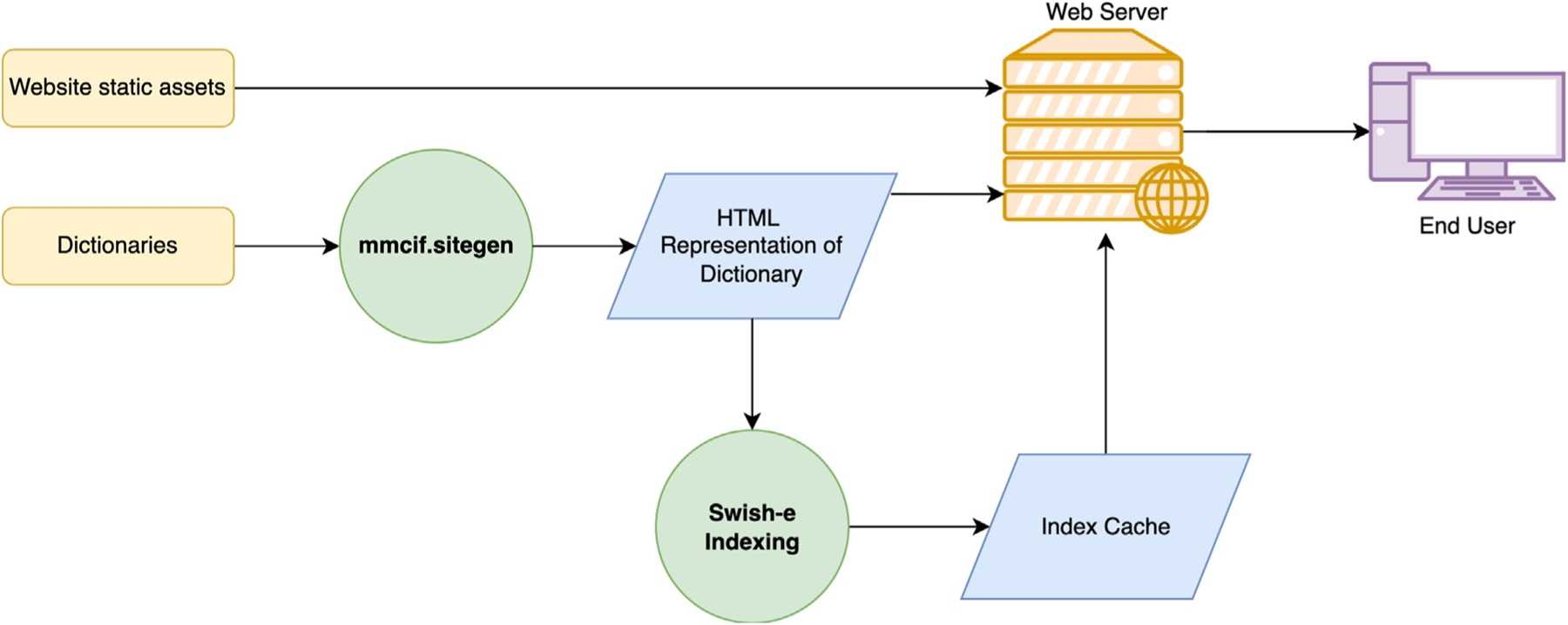
Process flow diagram of producing files for the mmcif.wwpdb.org website. The dictionary (storedingithub.com/rcsb/mmcif_website_file_assets) is converted to HTML using the mmcif.sitegen package. These HTML files are combined with the website assets (github.com/rcsb/mmcif_website) to serve as the static content of the site. The HTML files produced from the dictionaries are indexed with Swish-e to provide for search capability. Color coding: process inputs (yellow), programs run (identified in green circles), and resulting output files (blue).

To ensure 24–7–365 accessibility, the mmCIF website is managed by RCSB on machines located at both Rutgers, The State University of New Jersey and the San Diego Supercomputer Center-University of California San Diego. Data are delivered by an Apache server with views of both mmCIF and PDBML (pdbml.wwpdb.org). Only static files constructed from GitHub repositories are provided. As illustrated in Figure 5, dictionaries are converted to HTML and indexed with Swish-e.^50^ These HTML dictionary representations are combined with other static assets to produce the mmCIF website. A CGI script utilizing the Swish-e index enables search functionality. Bootstrap^51^ and jQuery (jquery.com) provide dynamic opening and closing of tabs. Category images are generated by the **dot** program in the Graphviz package.^52^

### Dictionary governance

Management and development of the PDBx/mmCIF dictionary is a collaborative effort shared between the wwPDB and a mmCIF Working Group (www.wwpdb.org/task/mmcif) (currently chaired by Paul D. Adams, Lawrence Berkeley National Laboratory). The Working Group provides guidance on dictionary content and promotes use of the data standard among structural biology methods developers. Membership includes expert experimentalists and representatives from structural biology software developer teams (*e.g*., CCP4,^15^ DIALS,^53^ Global Phasing, Ltd.,^54^ PDB-REDO,^55^ Phenix^56^ and XDS^57,58^).

Major changes to the organization of PDBx/mmCIF dictionary and the PDB archive are discussed within this group. Recent dictionary modifications include (i) the 2014 decision to make PDBx/mmCIF the master format of the PDB^29^; (ii) extension and support for representation of branched-chain carbohydrates^59^; (iii) extension and support for enhanced metadata associated with unmerged and anomalous “rotation” crystallography data; (iv) extension and support for new quality metrics for data with anisotropic diffraction limits (www.wwpdb.org/news/news?year=2021#60638-da1931d5660393084c3); and (v) the 2019 requirement that all MX structures be deposited into the PDB *via* the OneDep system in PDBx/mmCIF file format.^34^ Work is currently underway to create extensions that will support better archiving of serial crystallography data and metadata.

### Dictionary maintenance

The PDBx/mmCIF dictionary is typically updated once or twice each month, depending on requested changes. Change requests originate from wwPDB biocurators, the mmCIF Working Group, or outside users reporting issues *via* the dictionary’s GitHub site (github.com/wwpdb-dictionaries/mmcif_pdbx). Most dictionary updates pertain to expanding the controlled vocabulary enumerations to support ongoing improvements in the OneDep system. Updates are also added to support future enhancement of the OneDep system and improve data representation across the PDB Core Archive.

All wwPDB dictionaries must adhere to the DDL2 specification, plus any DDL extensions that they use. As previously described in the Dictionary-related Tools section, tools exist to validate a dictionary against the DDL2 specification. The DDL extension dictionary is first validated against the internal DDL implementation and then any other dictionary is validated against the DDL extension dictionary ensuring full compliance. wwPDB dictionaries may also require other self-consistency checks related to the domain being served. For example, in the PDBx/mmCIF dictionary, certain enumerations between categories need to be synchronized. To this end, an evolving set of automated tests have been developed using pytest.^60^ These tests can be run manually or through the use of automated GitHub tools on commits and pull requests.

### Use case pertaining to PDBx/mmCIF dictionary extensibility

Glycosylation is a process in which carbohydrate molecules are covalently linked to other biomolecules, typically proteins and lipids. Carbohydrate decoration of proteins supports a multitude of biological functions including molecular recognition, regulation, protease protection, molecular function switching, pathogen recognition, and signaling to name but a few.^61^

Historically, the PDB represented glycosylated proteins as carbohydrate ligands covalently linked to linear polymers. Searching for branched chain carbohydrates was impossible without writing custom software to search for connected carbohydrates. The challenge facing the wwPDB was how to describe branch chain carbohydrates in a standardized form.

wwPDB extended the PDBx/mmCIF dictionary with four new categories to represent non-linear sequences for carbohydrates.^59^ Specifically, a schema was developed to describe complex linkages within a carbohydrate chain and protein-carbohydrate linkages. Entity names for carbohydrate polymers are based on glycobiology community standards. Legacy PDB format files remain unchanged. The enriched annotation can only be retrieved from PDBx/mmCIF formatted files. The open-source molecular graphics tool Mol*,^22^ co-developed and used by both RCSB PDB and PDBe, has been adapted to present a branch chain carbohydrate rendering using these data. Examples of biologically-important macromolecular structures that utilize the carbohydrate extensions of the PDBx/mmCIF data dictionary include 3DEM structures of the SARS-Cov-2 Omicron Variant of Concern spike protein,^62^ and the entire Zika virus.^63^

## Results and Discussion

Formal development of PDBx/mmCIF data standards began in 1991. Initially, it was intended to provide macromolecular extension of the original IUCr CIF. During development, several far-reaching decisions were made. Arguably the most important of these choices was adoption of an improved DDL (DDL2) that had stronger data typing and introduction of formal links between items and categories to group related items together.^24^ Tighter linkage among data items was necessary to accommodate the more complicated relationships required for describing a biological macromolecule with groups of atoms constituting residues, groups of residues constituting polymers, and one or more polymers constituting entities. Such linkages ensure that references between data items are consistent and provide for more complete descriptions of biomolecules.

Use of a dictionary-based data archival storage format is not without tradeoffs. While PDBx/mmCIF offers a flexible and extensible format, file sizes and parsing times may be larger when compared to other fixed purpose, application specific file formats. For reference, legacy format PDB files with their fixed width column position and templated files, may be smaller in size, and faster to parse, but they are much harder to extend. CCP4 based MTZ files, used for storing diffraction data, are designed to be parsed by software only and store data values in a hardware byte-order based floating point representation.^15^ MTZ parsers need to convert these files for exchange with differing machine byte order. Hence, MTZ is not suitable for use as an archival format.

MMTF^64^ is another fixed binary format to represent macromolecular structures. While efficient to parse and compress, it is not human readable and represents only a subset of the metadata of macromolecule structures. While the technology may be applied to other types of data files, it is not readily extensible.

BinaryCIF^65^ as used by Mol*,^22^ utilizes a size efficient encoding of PDBx/mmCIF data. While it retains the original PDBx/mmCIF data, the contents are no longer readable by humans. One of the strengths of BinaryCIF is in the delivery of compressed MX electron density maps and 3DEM electric coulomb potential maps using a lossy quantization encoding. Such data could not be efficiently represented in PDBx/mmCIF format.

PDBML format^45^ on the other hand, is an ASCII based format derived from PDBx/mmCIF, in which each and every data item is written out as a separate XML element. While there are many XML parsers available and such files can be parsed by machines and humans, they are approximately ten-times the size of a PDBx/mmCIF file and take longer to parse.

Within the wwPDB, PDBx/mmCIF formatted files are also used to describe and validate CCD, BIRD, experimental diffraction data files, and, more recently, wwPDB validation reports. One of the advantages of PDBx/mmCIF file format *versus* binary formats is the use of the ASCII character set as an archival format, which will ensure its ability to be read in perpetuity. PDBx/mmCIF is infinitely extensible with a dictionary controlling descriptions, vocabulary, ranges, and relationships among data items. As described under dictionary governance, recent extensions pertain to serial crystallography data, branched-chain carbohydrates,^59^ and anisotropic diffraction data processing information.

While the PDBx/mmCIF dictionary enforces standards for data stored in the PDB archive (*e.g*., mandatory data items), DDL2 extensions allow the wwPDB to use the dictionary for more than simply supporting the PDB Core Archive. Specifically, extensions have been introduced to streamline processing of incoming structure data depositions and provide tighter controls on incoming data. While the PDBx/mmCIF dictionary supports the data accumulated over the 50-year history of the PDB, one of the goals by the OneDep system is for newer depositions to adhere to higher standards. For example, where free text items exist in dictionary definitions, DDL2 extensions allow the deposition system to utilize controlled vocabularies and more restrictive regular expressions. This approach allows the OneDep system to implement stricter data deposition requirements, on an as needed basis.

Another dictionary extension present in the DDL2 dictionary permits assignments of contexts to categories or individual data items. This feature is utilized by the dictionary tools package to selectively filter data from publicly-released PDB structures. Examples include internal data relevant to the processing of a PDB entry, deprecated items, and sensitive depositor information (*e.g*., email addresses, telephone numbers). A similar approach is used to filter information stored in the CCD and BIRD files.

Within the wwPDB, use of the **db-loader** tool allows a relational database to be used as an auxiliary source of information both during the wwPDB biocuration process and following public release. Entry status, citation information, contact authors, deposition titles, deposition information, chemical components, and more can be referenced from customized tools in the OneDep system. Loading of such information facilitates wwPDB remediation projects, wherein quality assessments can be performed by querying the database to examine archive-wide trends and identify outliers.

Just as PDBx/mmCIF is central to archiving efforts of the wwPDB partnership, dictionary extensions have been developed to support related archives. These extensions use the PDBx/mmCIF dictionary as a starting point, with domain specific information added. Such extensions have already been used for depositing, validating, and archiving computed structure models by the ModelArchive (modelarchive.org),^47^ integrative or hybrid methods experimental structures by PDBDev (pdb-dev.wwpdb.org),^49^ small angle scattering studies by Small Angle Scattering Biological Data Bank SASBDB (sasbdb.org),^66^ and recently for archiving computed structure models generated by AlphaFold 2.0 (alphafold.ebi.ac.uk).^67^ Widespread use of PDBx/mmCIF extensions ensures compatibility and interoperability between different biostructure data resources all using the common exchange format.

As described in more detail above, the PDBx/mmCIF dictionary itself and PDBx/mmCIF-related resources are provided on an open access basis by wwPDB partners. The mmcif.wwpdb.org website permits viewing of current dictionaries, DDL2, and several extension dictionaries (pdbdev, ModelArchive, wwPDB validation report, etc.). This website also supports the perpetual URL for the dictionary referenced in the audit records of PDBx/mmCIF PDB files (Figure 4).

Use of a standardized, machine readable, PDBx/mmCIF file format allows the wwPDB to satisfy all of the criteria required to be a purveyor of Findable, Accessible, Interoperable, and Reusable (FAIR) data.^68^ The wwPDB has been certified by Core-TrustSeal (coretrustseal.org). Tools used by wwPDB to create and maintain DDL2-based CIF dictionaries are publicly available, thereby ensuring that such standards are both readily accessible and durable.

Structural biology as a science is literally evolving before our eyes. At present, wwPDB is supporting the rapidly evolving 3DEM and serial crystallography methods, and emerging integrative/hybrid methods (I/HM). wwPDB partners are committed to working with stakeholder research communities to develop and incorporate extensions of PDBx/mmCIF dictionary into OneDep and PDB Core Archive. Efforts currently underway within the mmCIF Working Group include reorganization of experimental reflection data files to better support unmerged MX intensity data and standardization of computed structure model data files as broadly as possible.

## Supplementary Material

Article

## Abbreviations:

3D: three-dimensional
3DEM: three-dimensional Electron Microscopy
BIRD: Biologically Interesting molecule Reference Dictionary
BMRB: Biological Magnetic Resonance Bank
CCD: Chemical Component Dictionary
CIF: Crystallographic Information Framework
COMCIFS: Committee for the Maintenance of the CIF Standard
DDL: Dictionary Definition Language
FAIR: Findable, Accessible, Interoperable, and Reusable
I/HM: Integrative/hybrid method
IUCr: International Union of Crystallography
mmCIF: macromolecular Crystallographic Information Framework
MX: Macromolecular Crystallography
NMR: Nuclear Magnetic Resonance
PDB: Protein Data Bank
PDBe: Protein Data Bank in Europe
PDBj: Protein Data Bank Japan
PDBx: PDB Exchange
RCSB PDB: Research Collaboratory for Structural Bioinformatics PDB
wwPDB: Worldwide PDB
