# Supplementary material for: PDBx/mmCIF Ecosystem: Foundational Semantic Tools for Structural Biology
Source: J Mol Biol. Author manuscript; Available in PMC 2023 Jun 26. (PMC10292674; doi:10.1016/j.jmb.2022.167599)
Supplement: Article [file NIHMS1907597-supplement-Article.zip › PCA-MutPred--Prediction-of-Binding-Free-Energy-Change-Up_2022_Journal-of-Mol.pdf]

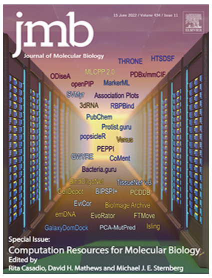

PCA-MutPred: Prediction of Binding Free Energy Change Upon Missense Mutation in Protein-carbohydrate Complexes

N. R. Siva Shanmugam [1], K. Veluraja [2] and M. Michael Gromiha [1]*

*1 - **Department of Biotechnology,** Bhupat and Jyoti Mehta School of Biosciences, Indian Institute of Technology Madras, Chennai 600036, India*
*2 - **PSN College of Engineering and Technology,** Melathediyoor, Tirunelveli, Tamil Nadu, India*

Correspondence to M. Michael Gromiha: *gromiha@iitm.ac.in (M. Michael Gromiha)*
https://doi.org/10.1016/j.jmb.2022.167526
Edited by Michael Sternberg

Abstract

Protein-carbohydrate interactions play an important role in several biological processes. The mutation of amino acid residues in carbohydrate-binding proteins may alter the binding affinity, affect the functions and lead to diseases. Elucidating the factors influencing the binding affinity change ($\Delta\Delta G$) of protein-carbohydrate complexes upon mutation is a challenging task. In this work, we have collected the experimental data for the binding affinity change of 318 unique mutants and related with sequence and structural features of amino acid residues at the mutant sites. We found that accessible surface area, secondary structure, mutation preference, conservation score, hydrophobicity and contact energies are important to understand the binding affinity change upon mutation. We have developed multiple regression equations for predicting the binding affinity change upon mutation and our method showed an average correlation of 0.74 and a mean absolute error of 0.70 kcal/mol between experimental and predicted $\Delta\Delta G$ on a 10-fold cross-validation. Further, we have validated our method using an independent test data set of 124 (62 unique) mutations, which showed a correlation and MAE of 0.79 and 0.56 kcal/mol, respectively. We have developed a web server PCA-MutPred, Protein-CArbohydrate complex Mutation affinity Predictor, for predicting the change in binding affinity of protein–carbohydrate complexes and it is freely accessible at https://web.iitm.ac.in/bioinfo2/pcamutpred. We suggest that the method could be a useful resource for designing protein-carbohydrate complexes with desired affinities.

© 2022 Elsevier Ltd. All rights reserved.

Introduction

Protein-carbohydrate interactions play a significant role in many biological and cellular processes including signalling, recognition and catalysis.[1–2] Understanding the recognition mechanism of protein-carbohydrate complexes provides valuable information on developing carbohydrate-derived therapeutics and glycomimetic drugs.[3] Petitou et al.[4] reported that synthetic pentasaccharide Fondaparinux interacts with antithrombin and inhibits coagulation factor Xa. Chen et al.[5] showed the importance of carbohydrate drug Voglibose in the treatment of diabetes by inhibiting α-glucosidase. These functions of protein-carbohydrate complexes as well as their recognition mechanisms are attributed with their binding affinities.[6–8] Recently, Veluraja et al.[9] reviewed the factors influencing the binding affinity of protein-carbohydrate complexes.

The binding affinities of protein-carbohydrate complexes are mediated by non-covalent interactions such as hydrogen bonds (direct and water-mediated), hydrophobic, electrostatic and van der Waals interactions at the interface.[9–11] Specifically, the residues Trp and Tyr predomi-

0022-2836/© 2022 Elsevier Ltd. All rights reserved.

nantly occur at the interface, which influence the formation of aromatic-aromatic interactions. The polar residues, Gln and Asn are also preferred at the interface, which are attributed with the formation of hydrogen bonds with carbohydrate hydroxyl groups.[12–13] Hudson et al.[11] analysed a set of protein-carbohydrate complexes from Protein Data Bank (PDB) and showed the importance of electrostatic and aromatic interactions. Houser et al.[14] emphasized the contribution of stacking interactions for the recognition of protein-carbohydrate complexes using computational analysis of binding site studies and *in vitro* analysis. Recently, Shanmugam et al.[15] developed a method PCA-Pred, to predict the binding free energy of protein-carbohydrate complexes using structure-based features such as binding site residues, interface area, interactions between various atoms and energetic contributions.

On the other hand, mutation of amino acid residues at the protein-carbohydrate interface changes the binding affinity. Shanmugam et al.[16] collected the data on experimentally determined binding affinity change upon mutation using frontal affinity chromatography (FAC), isothermal titration calorimetry (ITC), surface plasmon resonance (SPR) and fluorescence spectroscopy, and developed a database (ProCaff) for the binding affinity of protein-carbohydrate complexes and their mutants. Experimentally determining binding affinities are resource-intensive and time-consuming, and hence computational methods are necessary to predict the binding affinity change of protein-carbohydrate complexes upon mutation. On the other hand, binding affinity change upon mutation is reported to be important for understanding the effect of disease-causing mutations. Ni et al.[17] studied the effect of F95Y mutation in influenza B virus hemagglutinin and showed that this mutation changed the binding affinity by 4.08 kcal/mol and enhanced the pathogenicity. Ruiz et al.[18] investigated the mutation of F19 to Y in human galectin-8 with lactose, which is involved in rheumatoid arthritis, and it altered the conformation of the complex as well as binding affinity. Salomonsson et al.[19] evaluated a set of mutations in galectin-3 protein-β-galactoside complex and reported that binding affinity is an important factor to understand the potency to activate neutrophil leukocytes. Although several sequence and structure-based methods are reported in the literature for predicting the binding affinity change of protein–protein and protein-nucleic acid complexes upon mutation,[20–22] there is no method currently available to predict the change in binding free energy (ΔΔG) upon mutation in protein-carbohydrate complexes.

In this study, we collected the binding affinity change of 318 unique mutations in protein-carbohydrate complexes. The complexes are categorized into five groups based on the number of saccharides and protein chains, and the change in binding affinities are related to accessible surface area, secondary structure, conservation scores and hydrophobicity. Utilizing the information, we have developed multiple regression equations to relate the properties of protein-carbohydrate complexes and changes in binding affinities. We obtained a correlation and mean absolute error of 0.80 and 0.63 kcal/mol, respectively, between experimental and predicted ΔΔG on a training dataset and 0.79 and 0.56 kcal/mol on a test set of 124 mutations.

Dataset and methods

Dataset and classifications

We have searched ProCaff database for the binding affinity of protein-carbohydrate mutants, which have ΔG values for both wild-type and mutant, and we obtained a set of 318 mutations from 156 protein-carbohydrate complexes. We have computed the change in binding free energy using Eq. (1),

$$\Delta\Delta G = \Delta G_{mut} - \Delta G_{wt} \quad (1)$$

where ΔG_{mut} and ΔG_{wt} are the binding free energies of the mutant and wild-type of the complex, respectively. In addition, we have reversed the mutation from the mutant to wild-type by adding the negative sign to ΔΔG values[23–24] using Equation (2).

$$\Delta\Delta G(\Delta G_{mut} - \Delta G_{wt}) = -\Delta\Delta G(\Delta G_{wt} - \Delta G_{mut}) \quad (2)$$

where ΔG_{mut} and ΔG_{wt} are the binding free energies of the mutant and wild-type of the complex, respectively. Our final dataset contains 636 mutations and all of them are located at the binding sites of protein-carbohydrate complexes. These mutations are categorized into five groups based on the oligomeric nature of protein-carbohydrate complexes such as (i) monomer-monosaccharide, (ii) oligomer-monosaccharide, (iii) protein-disaccharide, (iv) protein-trisaccharide and (v) protein-oligosaccharide. These classifications considered the interface residues reliably predict the binding affinity of protein-carbohydrate complexes.[15] The complete dataset is available at https://web.iitm.ac.in/bioinfo2/pcamutpred/datasetdetails.html.

Our dataset contains 68 unique proteins, which belong to 64 non-homologous protein clusters with the cut-off of 40% sequence similarity using the CD-HIT.[25] We have divided the dataset into training and test set by 80:20 ratio. Moreover, we have evaluated the performance of the methods using stringent datasets that the mutations at the same position and protein clusters are either in the training or test set, making the training and test set evolutionarily independent.

Sequence-based features

We have initially generated a set of 137 sequence-based features and selected the

important properties for developing the method for predicting the binding free energy change upon mutation. These properties include (i) 69 physicochemical properties of amino acid residues,[26] (ii) 30 mutation matrices accounting the neighboring residue information,[27] (iii) 14 distance potentials and mutations matrices from AAIndex[28] and (iv) 24 position-specific scoring matrix (PSSM) and conservation scores.[29–30] The list of features used in this study is provided at https://web.iitm.ac.in/bioinfo2/pcamutpred/features.html. In addition, we used the information on the preference for aliphatic, aromatic, polar, charged and key residues (residues, which are involved in both binding and folding;[13] in the vicinity of the mutation sites with a window length of 3–19 residues (1–9 residues on both N- and C-directions). Further, we used feature selection procedure (discussed below) to reduce the number of features.

Structure-based features

We have obtained the structures of all the proteins considered in this study from AlphaFold DB[31] and AlphaFold 2.0.[32] Using the modeled structures, we have generated 17 structure-based features, which include (i) Residue depth obtained with the python package, Bio.PDB (ii) 3 hydrogen bond features from HBPLUS,[33] (iii) 10 atom-wise accessible surface area for the mutation position using NACCESS[34] and (iv) 3 residue-based accessible surface area using DSSP.[35]

Feature selection and model development

We have used the forward selection method for selecting the features.[15] In this method, we have carried out an exhaustive systematic search for all possible combinations of four features among all sequence and structure-based features. The best-performing combination was selected based on the highest correlation and lowest mean absolute error between experimental and predicted ΔΔG. Utilizing this combination as a starting point, we performed forward feature selection procedure to include additional features in each model. This procedure was continued until no increase in correlation (r) and a decrease in the mean absolute error (MAE) upon the addition of a new feature. To avoid overfitting, we restricted the number of features to be less than one-tenth of the number of data.[36] The selected features are used in the final model. We have utilized the python machine learning package scikit-learn for building linear regression models[37] using Eq. (3),

$$\Delta\Delta G = \beta_0 + \beta_1 x_{i1} + \beta_2 x_{i2} + \ldots + \beta_p x_{ip} \quad (3)$$

where, i is the number of observations, ΔΔG is a dependent variable (change in binding affinity), x_i are sequence and structure-based parameters, and β_0, β_1 … βp are regression coefficients. Further, we have used three univariate feature selection methods such as f_classif, f_regression, and mutual information for selecting the features based on different scores such as ANOVA f-value, F-statistic and p-value.

Performance validation

We have grouped the data into training/10-fold cross-validation and test sets with 512 and 124 mutations, respectively. The performance of the model was evaluated using Pearson's correlation coefficient (r) and mean absolute error (MAE) between predicted and experimental values. Further, we validated the model using leave-one-out cross validation, also known jack-knife test. In this method, the model is trained with n-1 data and the left-out mutation is used for prediction. The same procedure is repeated for n times, where n is the number of mutations in the dataset.[38] Additionally, we have examined the performance of the method using Spearman's rank correlation, root mean square error (RMSE) and the two tailed p-value.[39–40] (data not shown).

Results and discussion

The analysis on the binding affinity change of 636 mutations showed that it ranges from −6.12 to 6.12 kcal/mol. Specifically, The distribution of ΔΔG at various ranges is presented in Supplementary Figure S1. We observed that 68.6% of mutants altered the binding affinity within ± 1 kcal/mol. Interestingly, 31.4% of mutants decreased and increased the binding affinity by more than 1 kcal/mol, respectively.

We have grouped the 68 unique proteins into six classes based on their functions such as (i) lectins, (ii) transferases, (iii) sugar binding proteins, (iv) glycoside hydrolases, (v) carbohydrate binding modules, and (vi) periplasmic binding proteins, and our dataset contains 25, 13, 8, 14, 3 and 5, proteins respectively. Further, we compared the profile with ProCaff database and all the proteins, which have missense mutations in the database are used in the present study. Further, lectins are mostly distributed in oligomer-monosaccharide, protein-trisaccharide and protein-oligosaccharide classes whereas transferases are distributed in monomer-monosaccharide and protein-disaccharide classes. In addition, periplasmic binding proteins are present only in monosaccharide class.

We examined the factors influencing the binding affinity changes upon mutations using three-dimensional structures of specific complexes and a couple of examples are discussed below: The mutation M113F in α-hemolysin showed the binding free energy of −8.53 kcal/mol with the β-cyclodextrin whereas ΔG of wild-type (M113) is −3.02 kcal/mol. The change in affinity of −5.51 kcal/mol is attributed with the orientation of

phenylalanine, which interacts with β-cyclodextrin through CH-π interactions.[41] Further, Sit et al.[42] studied the interactions between *A. pleuropneumoniae* AfuA sugar-phosphate specific periplasmic binding protein and glucose-6-phosphate (G6P) to understand the importance of S37 using isothermal titration calorimetry. The substitution S37A reduced the binding free energy by 2.26 kcal/mol, which is mainly due to the loss of hydrogen bonds at the interface.

Supplementary Figure S2 shows the distribution of ΔΔG values of mutants in five classes of protein-carbohydrate complexes. Inspection of ΔΔG in five categories of protein-carbohydrate complexes revealed that the average ΔΔG of mutants in monomer-monosaccharide, protein-disaccharide and protein-trisaccharide are stronger than that in other complexes (oligomer-monosaccharide and protein-oligosaccharide).

Prediction models for different groups of protein-carbohydrate complexes

We have developed prediction models specific to five groups of protein-carbohydrate complexes and the performance obtained with training, 10-fold cross-validation and jack-knife test is presented in Table 1. It includes the number of properties used in the model, correlation coefficient and mean absolute error between experimental and predicted change in binding affinity upon mutation. We observed that the correlation coefficient lies in the range of 0.68 to 0.78 for jack-knife test with a MAE of 0.39 to 0.96 kcal/mol (Supplementary Figure S3). The detailed results for each class of protein-carbohydrate complex are presented in supplementary information. Further, the comparison of various feature selection methods showed that the performance with forward feature selection is better than other methods used in the present study (Supplementary Table S1).

Prediction of change in binding affinity

Overall, our method could relate the change in binding affinity of 512 mutations of with an average correlation of 0.80 and MAE of 0.63 kcal/mol on training. Further, it is capable of predicting ΔΔG with a correlation and MAE of 0.74 and 0.70 kcal/mol, respectively, on 10-fold cross-validation and 0.74 and 0.71 kcal/mol, respectively, in jack-knife test. Figure 1 shows the relationship between experimental and predicted binding affinities in training, 10-fold cross-validation and jack-knife test. We have checked the performance of the leave-one-cluster-out cross validation, which showed a correlation coefficient of 0.63 and MAE of 0.81 kcal/mol. We have compared the performance of the linear regression models with seven other machine algorithms and the results are presented in Supplementary Table S2. We observed that the performance of our method is better than other machine learning techniques based on correlation coefficient and MAE.

Validation of prediction methods

We have validated the performance of our methods on predicting the ΔΔG using the test set of 124 mutants in 39 protein-carbohydrate complexes. Figure 2 shows the relationship between experimental and predicted change in binding affinities. We observed that the ΔΔG is predicted within an MAE of 0.56 kcal/mol and the correlation between experimental and predicted affinities is 0.79. The MAE for the complexes belonging to five categories, monomer–monosaccharide, oligomer-monosaccharide, protein-disaccharide, protein-trisaccharide and protein-oligosaccharide are 0.52, 0.65, 0.52, 0.44 and 0.66 kcal/mol, respectively. Interestingly, 84.7% of the test set ΔΔG values are predicted within the deviation of ± 1 kcal/mol.

Bootstrapping results. We have evaluated the robustness of our method using bootstrapping. We have constructed redundant datasets by oversampling the mutations ten times and 80% data are used for prediction. We repeated this procedure 1000 times and the results are presented in Supplementary Table S3. We found that the results are consistent and our method performed with an average correlation of 0.80 ± 0.03 and an average MAE of 0.60 ± 0.16.

Table 1 Performance of prediction methods for five groups of protein-carbohydrate complexes obtained with training, 10-fold cross-validation and jack-knife test.

			Training		10-fold CV		Jack-knife	
**Classification**	**Features**	**N**	**CC**	**MAE**	**CC**	**MAE**	**CC**	**MAE**
Monomer-monosaccharide	14	132	0.76	0.85	0.68	0.96	0.68	0.96
Oligomer-monosaccharide	7	66	0.8	0.41	0.76	0.45	0.75	0.46
Protein-disaccharide	11	124	0.8	0.35	0.76	0.39	0.76	0.39
Protein-trisaccharide	9	76	0.82	0.66	0.76	0.74	0.75	0.76
Protein-oligosaccharide	10	114	0.82	0.78	0.78	0.86	0.78	0.87
**Overall**		**512**	**0.80**	**0.63**	**0.74**	**0.70**	**0.74**	**0.71**

N-Number of mutations; CC: correlation coefficient; MAE: mean absolute error.

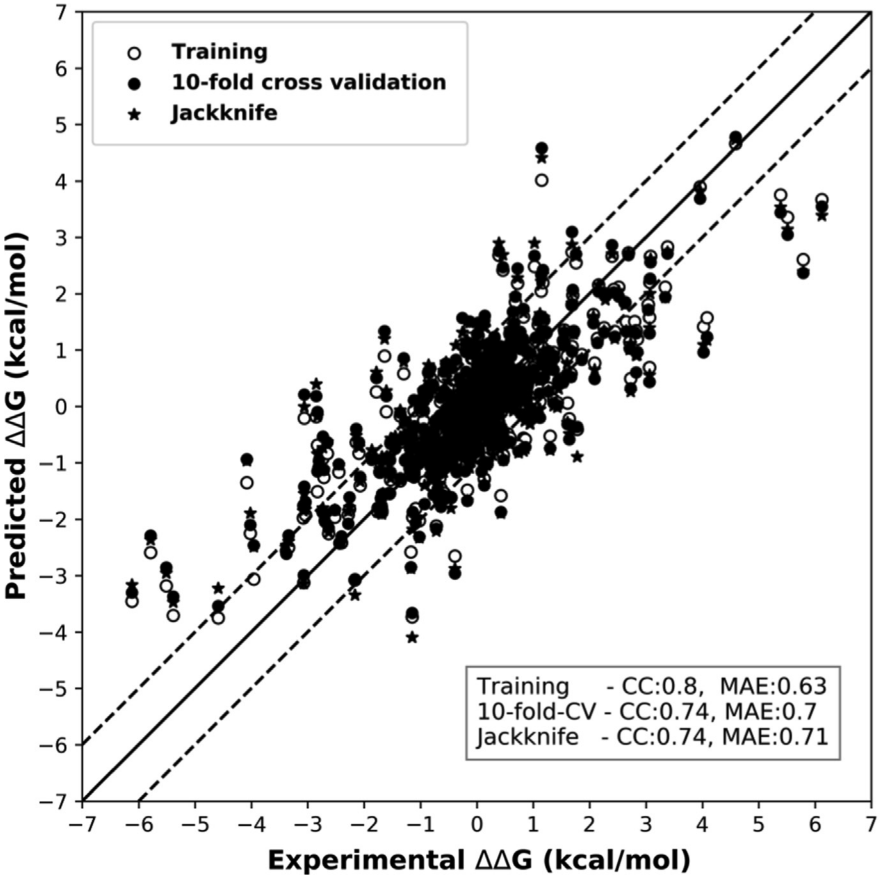

Figure 1. A scatter plot showing the relationship between experimental and predicted binding affinities on training, 10-fold cross-validation and jack-knife test. The solid line represents ideal prediction and the dotted line shows the mutations, which are predicted within a deviation of ± 1 kcal/mol.

These correlation and MAE are similar to the values obtained on training (Table 1), which showed that our method is robust and it avoided overfitting. Further, in each classification monomer-monosaccharide, oligomer-monosaccharide, protein-disaccharide, protein-trisaccharide and protein-oligosaccharide the present method predicted with a correlation of 0.75, 0.83, 0.79, 0.82 and 0.82, respectively and the MAE are 0.79, 0.45, 0.38, 0.62 and 0.76 kcal/mol, respectively.

Analysis of selected features

We observed that the properties, accessible surface area, secondary structure, conservation score, mutation preference, hydrophobicity, free energy and contact energies are important for predicting the change in binding affinity upon mutation. Additional parameters such as entropy, non-bonded energy, volume and side-chain interaction are specific to each class (Supplementary Tables S4 and S5 (a)). Further analysis showed that accessible surface area, secondary structures, mutation preference, conservation score are identified to be important in more than four classes. The properties PSSM, non-bonded energy, volume and side-chain interaction are specific to monomer-monosaccharides, oligomer-monosaccharides, protein-disaccharides and protein-oligosaccharides, respectively. The regression models for all five classes are presented in Supplementary Table S6.

Further, we have examined the features identified in the present method for predicting the change in binding affinities upon mutations and the structure-based features used in PCA-Pred for predicting the binding affinities of protein-carbohydrate complexes. The features such as key residues, propensity, side-chain accessibility, number of binding site residues, volume of binding site residues and accessible surface area are found to be important for predicting the binding affinity in PCA-Pred. In the present study, the properties accessible surface area, secondary structure, conservation score, mutation preference, hydrophobicity, free energy and contact energies are preferred for predicting the binding affinity change upon mutation. The comparison of features used in PCA-Pred and PCAMutPred (Supplementary Table S5 (b)), showed that contact energies, accessible surface area and side-chain interactions are in common. On the

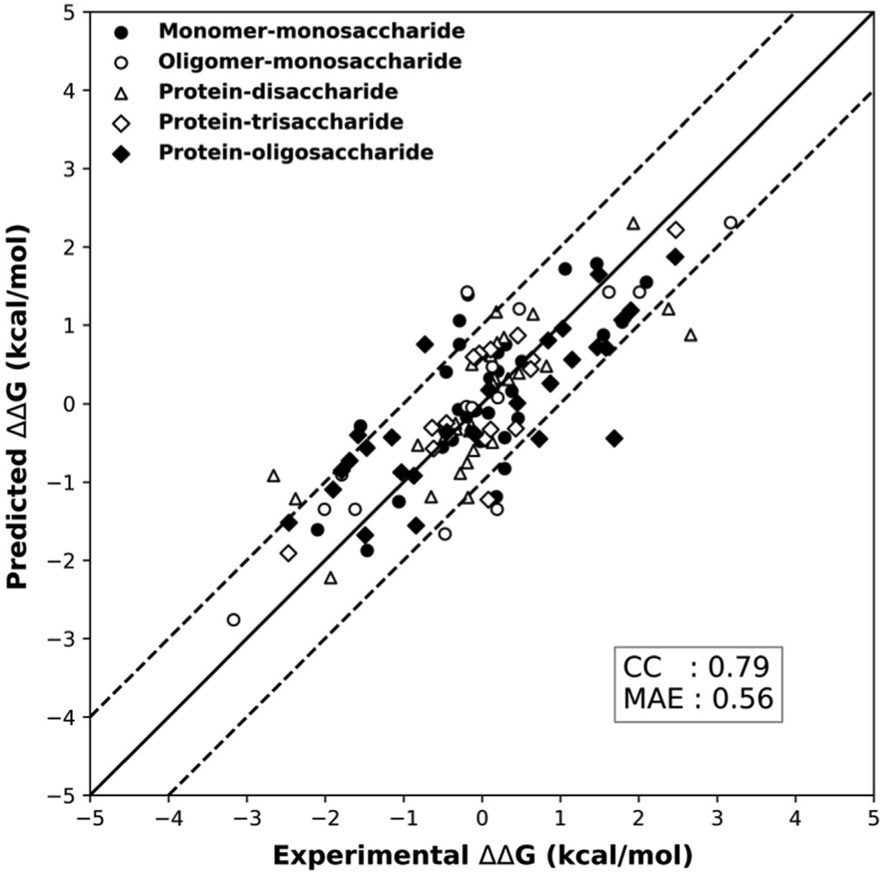

Figure 2. Experimental and predicted change in binding affinity on a test set of 124 mutants in 39 protein-carbohydrate complexes. The solid and dashed lines show the ideal prediction and a deviation of ± 1 kcal/mol, respectively.

other hand, PCAPred mainly depends on binding site residues and carbohydrate-based features, whereas location of mutants and sequence-based features are identified to be important in PCAMutPred.

Comparison of prediction performance using PCA-Pred and present work

We have compared the performance of predicting the changes in binding affinities of protein-carbohydrate complexes using PCAMutPred and PCA-Pred, which predict the binding affinity of protein-carbohydrate complexes using their three-dimensional structures. We have collected a set of 58 mutations in 9 protein-carbohydrate complexes, which have experimentally known structures in Protein Data Bank.[43] We have constructed the mutants using the mutagenesis option available in PyMOL.[44] Further, we obtained the binding energy (ΔG) values for both wild-type and mutant structures of the complexes using PCA-Pred and computed $\Delta\Delta G$ using Eq. (1). The comparison of predicted $\Delta\Delta G$ using PCA-Pred and PCAMutPred with experimental $\Delta\Delta G$ is shown in Supplementary Figure S4. We observed that the average MAE obtained with PCA-Pred and PCAMutPred are 1.26 kcal/mol and 0.51 kcal/mol, respectively. This result shows that the performance of the present method, which is specifically designed for the change in binding affinity of mutants is better than PCA-Pred.

Validation of PCAMutPred using a blind dataset

We have validated the performance of the model using a blind dataset of 16 mutations from 5 complexes collected from the literature, which were not used in the training and test sets, and not available in ProCaff database. We have doubled the data using reverse mutations as stated in Eq. (2) and the final validation dataset has 32 mutations, which are distributed into 14, 10 and 8 in protein-trisaccharide, protein-oligosaccharide and protein-disaccharide classes, respectively. We have predicted the binding free energy changes using our method and the results are presented in Supplementary Table S7. We observed that our method could predict the $\Delta\Delta G$ with an average MAE of 0.69 kcal/mol.

Limitations

Our method enables the prediction of changes in binding affinity caused by mutations using amino

acid sequence-based features. The mean absolute error is within a limit of 0.7 kcal/mol in most of the cases (Figures 1 and 2). However, our method is not able to predict the free energy change of few mutations accurately as these mutations are highly influenced with structural parameters of the complexes and contacts between protein and carbohydrate in a complex. Our method is designed for predicting the binding affinity change upon mutation at the interface of a protein-carbohydrate complex and binding site information can be obtained using other methods reported in the literature.[45–47] Further, experimental conditions, carbohydrate features, interactions with ions and multivalency/multiple binding sites are also not considered in the present method. Another limitation is the dataset of limited mutations and our method could be improved upon the availability of large number of experimental data.

Web server

We have developed a web interface PCA-MutPred, which can be accessed at (https://web.iitm.ac.in/bioinfo2/pcamutpred). It takes the protein sequence in FASTA format along with a list of mutations, UniProt ID or modelled/experimental structures and the appropriate classification model as input, and automatically predicts the $\Delta\Delta G$ values and display the output results. The interface also contains the details about the methodology of PCA-MutPred, performance on training and test sets, a detailed tutorial page and answers to frequently asked questions.

Conclusions

We have developed a novel sequence and structure-based machine-learning method to predict the changes in binding affinity in protein-carbohydrate complexes and our analysis showed that accessible surface area, mutation preference, secondary structure, conservation score, hydrophobicity and contact energies are essential for reliably predicting $\Delta\Delta G$ values. Our method showed a correlation and MAE of 0.79 and 0.56 kcal/mol between predicted and experimental binding free energy change upon mutation in a test set of 124 mutants. It is hoped that our method can provide deep insights into the effect of mutations in protein-carbohydrate complexes and predict the binding affinity change upon mutation. Further, it serves as a useful resource for relating the binding affinity change with disease-causing mutations.

Acknowledgement

We wish to acknowledge the reviewers for their constructive comments. Authors thank the Bioinformatics Facility, the Indian Institute of Technology for computational facilities. The authors also thank Nisha Harur Muralidharan for her critical reading of the manuscript.

Funding

The Department of Biotechnology, Government of India to M.M.G. Ministry of Education, India and DST-INSPIRE fellowship (IF170342) to N.R.S.S.

Declaration of Competing Interest

The authors declare that they have no known competing financial interests or personal relationships that could have appeared to influence the work reported in this paper.

Appendix A. Supplementary data

Supplementary data to this article can be found online at https://doi.org/10.1016/j.jmb.2022.167526.

Received 4 November 2021;
Accepted 1 March 2022;
Available online 5 March 2022

Keywords:
protein-carbohydrate complexes;
binding free energy change;
contact potentials;
sequence-based features

References

1. Williams, S.J., Davies, G.J., (2001). Protein–carbohydrate interactions: learning lessons from nature. *Trends Biotechnol.* **19**, 356–362.
2. Varki, A., Cummings, R.D., Esko, J.D., Freeze, H.H., Stanley, P., Bertozzi, C.R., et al., (2009). Essentials of glycobiology. Cold Spring Harbor Laboratory Press, New York.
3. Ernst, B., Magnani, J.L., (2009). From carbohydrate leads to glycomimetic drugs. *Nat. Rev. Drug Discov.* **8**, 661–677.
4. Petitou, M., Duchaussoy, P., Herbert, J.M., Duc, G., El Hajji, M., Branellec, J.F., et al., (2002). The synthetic pentasaccharide fondaparinux: first in the class of antithrombotic agents that selectively inhibit coagulation factor Xa. *In: Seminars in thrombosis and hemostasis, 48.* Thieme Medical Publishers, New York, pp. 393–402.
5. Chen, X., Zheng, Y., Shen, Y., (2006). Voglibose (Basen®, AO-128), one of the most important α-glucosidase inhibitors. *Curr. Med. Chem.* **13**, 109–116.
6. Nangia-Makker, P., Hogan, V., Raz, A., (2018). Galectin-3 and cancer stemness. *Glycobiology* **28**, 172–181.
7. Oda, M., Inaba, S., Kamiya, N., Bekker, G.J., Mikami, B., (2018). Structural and thermodynamic characterization of endo-1,3-b-glucanase: insights into the substrate recognition mechanism. *Biochim. Biophys. Acta Proteins Proteom.* **1866**, 415–425.

8. Ota, F., Hirayama, T., Kizuka, Y., Yamaguchi, Y., Fujinawa, R., Nagata, M., et al., (2018). High affinity sugar ligands of C-type lectin receptor langerin. *Biochim. Biophys. Acta Gen. Subj.* **1862**, 1592–1601.
9. Veluraja, K., Shanmugam, N.R.S., Blessy, J.J., Gromiha, M.M., (2020). Protein-carbohydrate complexes: binding site analysis, prediction, binding affinity and molecular dynamics simulations. In: Gromiha, M.M. (Ed.), *Protein Interactions: Computational Methods, Analysis and Applications*. World Scientific Press, Singapore, pp. 299–332.
10. Nishio, M., Umezawa, Y., Fantini, J., Weiss, M.S., Chakrabarti, P., (2014). CH–π hydrogen bonds in biological macromolecules. *Phys. Chem. Chem. Phys.* **16**, 12648–12683.
11. Hudson, K.L., Bartlett, G.J., Diehl, R.C., Agirre, J., Gallagher, T., Kiessling, L.L., Woolfson, D.N., (2015). Carbohydrate–aromatic interactions in proteins. *J. Am. Chem. Soc.* **137**, 15152–15160.
12. Gromiha, M.M., Veluraja, K., Fukui, K., (2014). Identification and analysis of binding site residues in protein-carbohydrate complexes using energy based approach. *Prot. Pept. Lett.* **21**, 799–807.
13. Shanmugam, N.R.S., Selvin, J.F.A., Veluraja, K., Gromiha, M.M., (2018). Identification and analysis of key residues involved in folding and binding of protein-carbohydrate complexes. *Protein Pept. Lett.* **25**, 379–389.
14. Houser, J., Kozmon, S., Mishra, D., Hammerová, Z., Wimmerová, M., Koča, J., (2020). The CH–π interaction in protein–carbohydrate binding: bioinformatics and in vitro quantification. *Chemistry–A* **26**, 10769–10780.
15. Shanmugam, N.R.S., Blessy, J.J., Veluraja, K., Gromiha, M.M., (2021). Prediction of protein–carbohydrate complex binding affinity using structural features. *Brief Bioinform.* **22** Article bbaa319.
16. Shanmugam, N.R.S., Blessy, J.J., Veluraja, K., Michael Gromiha, M., (2020). ProCaff: protein–carbohydrate complex binding affinity database. *Bioinformatics* **36**, 3615–3617.
17. Ni, F., Mbawuike, I.N., Kondrashkina, E., Wang, Q., (2014). The roles of hemagglutinin Phe-95 in receptor binding and pathogenicity of influenza B virus. *Virology* **450**, 71–83.
18. Ruiz, F.M., Scholz, B.A., Buzamet, E., Kopitz, J., André, S., Menéndez, M., et al., (2014). Natural single amino acid polymorphism (F19Y) in human galectin-8: detection of structural alterations and increased growth-regulatory activity on tumor cells. *FEBS J.* **281**, 1446–1464.
19. Salomonsson, E., Carlsson, M.C., Osla, V., Hendus-Altenburger, R., Kahl-Knutson, B., Öberg, C.T., et al., (2010). Mutational tuning of galectin-3 specificity and biological function. *J. Biol. Chem.* **285**, 35079–35091.
20. Gromiha, M.M., Yugandhar, K.A., Jemimah, S., (2017). Protein–protein interactions: scoring schemes and binding affinity. *Curr. Opin. Struct. Biol.* **44**, 31–38.
21. Jemimah, S., Kumar, Y., Gromiha, M.M., (2020). Binding affinity of protein–protein complexes: experimental techniques, databases and computational methods. In: Gromiha, M.M. (Ed.), *PROTEIN INTERACTIONS: Computational Methods, Analysis and Applications*. World Scientific Press, Singapore, pp. 87–108.
22. Jiang, Y., Liu, H.F., Liu, R., (2021). Systematic comparison and prediction of the effects of missense mutations on protein-DNA and protein-RNA interactions. *PLoS Comput. Biol.* **17** Article e1008951.
23. Capriotti, E., Fariselli, P., Rossi, I., Casadio, R., (2008). A three-state prediction of single point mutations on protein stability changes. *BMC Bioinformatics* **9**, Article S6.
24. Sanavia, T., Birolo, G., Montanucci, L., Turina, P., Capriotti, E., Fariselli, P., (2020). Limitations and challenges in protein stability prediction upon genome variations: towards future applications in precision medicine. *Comput. Struct. Biotechnol. J.* **18**, 1968–1979.
25. Li, W., Godzik, A., (2006). Cd-hit: a fast program for clustering and comparing large sets of protein or nucleotide sequences. *Bioinformatics* **22**, 1658–1659.
26. Chaudhary, P., Naganathan, A.N., Gromiha, M.M., (2015). Folding RaCe: a robust method for predicting changes in protein folding rates upon point mutations. *Bioinformatics* **31**, 2091–2097.
27. Anoosha, P., Huang, L.T., Sakthivel, R., Karunagaran, D., Gromiha, M.M., (2015). Discrimination of driver and passenger mutations in epidermal growth factor receptor in cancer. *Mutat Res.* **780**, 24–34.
28. Kawashima, S., Kanehisa, M., (2000). AAindex: amino acid index database. *Nucleic Acids Res.* **28**, 374.
29. Manning, J.R., Jefferson, E.R., Barton, G.J., (2008). The contrasting properties of conservation and correlated phylogeny in protein functional residue prediction. *BMC Bioinformatics* **9**, Article 51.
30. Valdar, W.S.J., (2002). Scoring residue conservation. *Proteins* **48**, 227–241.
31. Varadi, M., Anyango, S., Deshpande, M., Nair, S., Natassia, C., Yordanova, G., et al., (2022). AlphaFold Protein Structure Database: Massively expanding the structural coverage of protein-sequence space with high-accuracy models. *Nucleic Acids Res.* **50**, D439–D444.
32. Jumper, J., Evans, R., Pritzel, A., Green, T., Figurnov, M., Ronneberger, O., et al., (2021). Highly accurate protein structure prediction with AlphaFold. *Nature* **596**, 583–589.
33. McDonald, I.K., Thornton, J.M., (1994). Satisfying hydrogen bonding potential in proteins. *J. Mol. Biol.* **238**, 777–793.
34. Hubbard, S.J., Thornton, J.M., (1993). NACCESS. University College London, Department of Biochemistry and Molecular Biology.
35. Kabsch, W., Sander, C., (1983). Dictionary of protein secondary structure: pattern recognition of hydrogen-bonded and geometrical features. *Biopolymers* **22**, 2577–2637.
36. Liu, P., Long, W., (2009). Current mathematical methods used in QSAR/QSPR studies. *Int. J. Mol. Sci.* **10**, 1978–1998.
37. Pedregosa, F., Varoquaux, G., Gramfort, A., Michel, V., Thirion, B., Grisel, O., et al., (2011). Scikit-learn: machine learning in python. *J. Mach. Learning Res.* **12**, 2825–2830.
38. Friedman, J., Hastie, T., Tibshirani, R., (2001). The elements of statistical learning (Vol 1). Springer series in statistics, New York.
39. F.J.W.M. Dankers, A. Traverso, L. Wee, S.M.J. van Kuijk, Prediction modeling methodology, in: Fundamentals of clinical data science (Kubben, P., Dumontier, M., Dekker, A.), Springer, Cham, 2019, pp.101-120.
40. Botchkarev, A., (2019). A new typology design of performance metrics to measure errors in machine learning regression algorithms. *Interdisciplinary J. Inform., Knowledge & Manage.* **14**, 45–76.
41. Banerjee, A., Mikhailova, E., Cheley, S., Gu, L.Q., Montoya, M., Nagaoka, Y., et al., (2010). Molecular

bases of cyclodextrin adapter interactions with engineered protein nanopores. *Proc. Natl. Acad. Sci. U. S. A.* **107**, 8165–8170.

42. Sit, B., Crowley, S.M., Bhullar, K., Lai, C.C.L., Tang, C., Hooda, Y., et al., (2015). Active transport of phosphorylated carbohydrates promotes intestinal colonization and transmission of a bacterial pathogen. *PLoS Pathog* **11** Article e1005107.
43. Burley, S.K., Bhikadiya, C., Bi, C., Bittrich, S., Chen, L., Crichlow, G.V., et al., (2021). RCSB Protein Data Bank: powerful new tools for exploring 3D structures of biological macromolecules for basic and applied research and education in fundamental biology, biomedicine, biotechnology, bioengineering and energy sciences. *Nucleic Acids Res.* **49**, D437–D451.
44. DeLano, W.L., (2002). PyMOL. *DeLano Scientific: San Carlos, CA* **2002**, 700.
45. Banno, M., Komiyama, Y., Cao, W., Oku, Y., Ueki, K., Sumikoshi, K., et al., (2017). Development of a sugar-binding residue prediction system from protein sequences using support vector machine. *Comput. Biol. Chem.* **66**, 36–43.
46. Gattani, S., Mishra, A., Hoque, M.T., (2019). StackCBPred: A stacking based prediction of protein-carbohydrate binding sites from sequence. *Carbohydr. Res.* **486**, 107857
47. Malik, A., Firoz, A., Jha, V., Ahmad, S., (2010). PROCARB: a database of known and modelled carbohydrate-binding protein structures with sequence-based prediction tools. *Adv. Bioinformatics* **2010**, 436036
