# Supplementary material for: PDBx/mmCIF Ecosystem: Foundational Semantic Tools for Structural Biology
Source: J Mol Biol. Author manuscript; Available in PMC 2023 Jun 26. (PMC10292674; doi:10.1016/j.jmb.2022.167599)
Supplement: Article [file NIHMS1907597-supplement-Article.zip › Computational-Resources-for-Molecular-Biology_2022_Journal-of-Molecular-Biol.pdf]

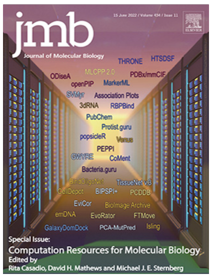

Computational Resources for Molecular Biology 2022

We are delighted to present this Special Issue reporting “Computational Resources for Molecular Biology 2022”. There is an ever-increasing sophistication of these resources. Many groups are reporting databases that integrate across modalities often building upon the wealth of information in the major sequence and structural databases such as UniProt[1] and the Protein Data Bank (PDB).[2] Other resources employ a range of machine learning techniques to develop novel predictive algorithms. As in previous years, the application domains span a wide range of topics in molecular biology including RNA structure analysis, transcriptomics, genome organisation, together with studies of protein structure, function, and interactions. Many resources focus on the implication of fundamental research to enhance our understanding of the molecular basis of disease and the use of drugs for treatment.

This year, a number of reports describe resources to analyse or query transcriptomic data. **Bacteria.guru**[3] and **Protist.guru**[4] are webservers that provide comparative genomic and transcriptomic analyses of bacterial and protistan species. This enables the discovery of pathways and comparison of pathways across species. Three reports focus on single-cell transcriptomics, which is timely as these data are increasingly available.[5] First, **popsicleR**[6] is an interactive R package for pre-processing and performing quality control assessment on single-cell RNA-seq data. Second, **APL**[7] is an R package for visualizing single cell transcriptomic data using association plots. These plots can provide a basis for clustering cells. Third, **CellDepot**[8] is a webserver to which single-cell RNAseq data can be uploaded and interactively explored. It also facilitates comparison to previously uploaded datasets.

Two reports describe tools focused on sequence analysis. **THRONE**[9] provides a web server for predicting guanine N^7-methylation sites. It uses machine learning to discern sites of methylation from background. **Isling**[10] is a software tool for identifying viral or vector integration events using paired-end next generation sequencing data. This is important for understanding the relationships between host genomes and viruses.

Four reports focus on predictions of nucleic acid structure. **RBPBind**[11] computes binding curves for protein-RNA binding, accounting for RNA secondary structure. **BINDOLIGONET**[12] estimates in linear time the cost of opening intramolecular RNA base pairs for interaction with other molecules. The tool **emDNA**[13] models mesoscale DNA structures, including DNA circles, using rigid-body parameters. **3dRNA**[14] is a tool that predicts all-atom RNA structures.

Several resources provide an approach to model binding sites in proteins. The **SVMyr**[15] web server identifies where a myristoyl group is covalently attached to an exposed glycine residue. The server detects both co- and post-translational myristoylation. The approach uses a Support Vector Machines to learn from sequence features including composition and physiochemical features of the octapeptides that are the substrate for myristoylation. **BIPSPI+**[16] is the next generation software that predicts protein binding interfaces. It learns from a carefully crafted dataset that, for example, distinguish between homo and hetero complexes. The web server can predict interfaces from two sequences, one sequence and one structure, and two structures. Once binding sites are predicted the user can dock two structures using the PatchDock software.[17] The **GalaxyDomDock**[18] web server predicts an ensemble of conformations for the docking of two protein domains. The linking region of domains provides constraints, and the conformation of this linking region is also modelled. **FTMove**[19] detects and analyses cryptic and allosteric binding sites by mapping multiple PDB structures of a candidate protein and finding hot spots and putative ligand binding sites.

Beyond pairwise association, studies are providing genome-wide maps of protein–protein interactions (PPIs). **PEPPI**[20] allows the identification of proteome-wide protein–protein interaction by integrating structural and sequence similarity, with functional association data and machine learning. **OpenPIP**[21] is an open-source customizable web portal that facilitates the hosting, visualization and analysis of PPI data. The authors consider that the resource can be particularly valuable in the early reporting and analysis of PPI data, such as on submission of a paper. The resource will complement deposition in standard databases.

With the explosion in genome sequencing, particularly in identifying human variations,

resources are widely used to predict the impact of a missense variant. **GWYRE**[22] (Genome Wide PhYRE) is a resource for mapping variants onto experimental and model structures of human protein complexes. The method integrates knowledge based tertiary structure prediction using Phyre2 and quaternary structure prediction using template based docking, and generate models of binary complexes which allow focusing on the human variome and the effect of pathological variations. **Venus** allows the interpretation of why a variation can be pathogenic by predicting its effect on protein stability and by assemblying the known multiple effects that are annotated in different data bases, including those pertaining to nearby residues. The **PCA-MutPred**[23] web server provides an approach to model the free energy change of a missense variant in a protein-carbohydrate complex. The study is based on a dataset of 318 unique mutants. Multiple regression equations are derived relating the difference in free energy to features such as surface area, conservation score, and hydrophobicity.

Sequencing has also provided extensive microbiome datasets and a major application is to discover features or biomarkers that distinguish two classes, such as case and control. **MarkerML**[24] is a web server that employs interpretable machine learning to discover metagenomic features. The paper reports a use case to identify metagenomic markers in human periodontis using data downloaded from the microbiome resource European Bioinformatics Institute. The authors point out that marker identification does not imply causality.

New methods provide insights for discovering structural and functional features in large data sets. **EvoRator**[25] implements a machine-learning regression algorithm for associating 3D structures to evolutionary-rate at specific protein sites, overpassing the need of a large set of homologous proteins. By this, it is possible to compute evolutionary rate of orphan proteins and of positions in gapped sequence alignments. **HTSDSF Explorer**[26] is a comprehensive tool to analyze high–throughput differential scanning fluorimetry (DSF) screenings for the identification of new drugs and novel therapeutic targets. It simplifies the determination of the melting temperature of the target and the change induced by ligand binding, allowing the determination of binding constants in dose–response assays. **MLCPP 2.0**[27] predicts cell-penetrating peptides and their uptake efficiency, outperforming state of the art methods and facilitating the design of hypothesis-driven experiments.

We have reports of updates to established and widely-used databanks.The PDB is the leading repository of structural coordinates. They report on the **PDBx/mmCIF Ecosystem**,[28] including details for the file standards, the governance of extending the standards, and a summary of software tools for processing and checking the data. The **Protein Circular Dichroism Data Bank (PCDDB)**[29] reports validated circular dichroism spectra with the associated metadata and links to publications. Entries are linked to the definitive protein data bank (PDB) and UniProt sequence entries. The database includes spectral details of wild-type and variant proteins. **PubChem**[30] is a public chemical database and has millions of users each month. The article reports the expansion of information in this resource linking chemicals to biological data including proteins, genes, pathways and organisms. The information can be accessed both interactively and programmatically.

A major target area of molecular biology resources is to further understanding of the molecular and genetic basis of disease. The **Organ-Disease Annotations (ODiseA)**[31] addresses the observation that often hereditary diseases are observed in one or a limited number of tissues. The **ODiseA** database contains a large depository of information, including substantial manual curation, for 2,181 hereditary diseases and 45 associated tissues. Users can search the database by disease, gene, and tissue. Analysis of the data showed that most diseases (91%) are inflicted only on up to three tissues. **TissueNet v.3**[32] is a related resource from the same group. It displays protein–protein interactions at the level of specific tissues where the particular proteins are highly expressed. Version 3 includes 125 adult and 7 embryonic tissues. The **EviCor**[33] toolbox is designed to facilitate exploration of anti-cancer drug responses as provided in the two widely-used public resources: The Cancer Genome Atlas[34] and The Cancer Cell Line Encyclopedia.[35] The resource provides information on phenotypes, molecules and pathways. Users can also access the resource via a REST interface. **CoMent**,[36] by detecting co-mentions in the scientific literature with text mining, offers a resource storing millions of relationships between thousands of terms representing diseases, symptoms, biological processes, molecular functions and cellular compartments, which allows searching for term association.

A novel resource at the EMBL-EBI is **The Bioimage Archive**,[37] storing all the images associated to biological data in publications with the aim of stirring new insights by the reuse of all type of images, with different resolutions, from molecular to organism scale. The database stores images in order to avoid duplication and particularly to generate a reliable and controlled archive for training and testing old and new tools for bio-image analysis.

This year we note an emphasis on resources that involve data storage and integration that facilitate enhanced interrogation of the biological information by users. At the end of 2020, the community witnessed a breakthrough in protein structure prediction delivered by AlphaFold that employed deep learning.[38] However, the limited

volumes of data in many biological domains leads to groups continuing to develop resources that employ well-established machine learning approaches. Finally, we would like to thank all our contributors to this Special Issue.

Grants

RC was supported by: FABIT-ECOI2020 and RFO-UNIBO. DHM was supported by National Institutes of Health grant R01GM132185. MJES was supported by Biotechnology and Biological Sciences Research Council (BBRSC) under grants BB/T010487/1 and BB/P023959/1 and by Imperial College London.

References

1. The UniProt Consortium, (2021). UniProt: the universal protein knowledgebase in 2021. *Nucleic Acids Res.* **49**, D480–D489. https://doi.org/10.1093/nar/gkaa1100.
2. Velankar, S., Burley, S.K., Kurisu, G., Hoch, J.C., Markley, J.L., (2021). The Protein Data Bank Archive. *In: Methods Mol. Biol.*. Springer, US, pp. 3–21. https://doi.org/10.1007/978-1-0716-1406-8_1.
3. Lim, P.K., Davey, E.E., Wee, S., Seetoh, W.S., Goh, J.C., Zheng, X., Phang, S.K.A., Seah, E.S.K., et al., (2021). Comparative Transcriptomics and Co-Expression Database for Bacterial Pathogens. *J. Mol. Biol.*, 167380. https://doi.org/10.1016/j.jmb.2021.167380.
4. Villanueva, E.M.F., Lim, P.K., Lim, J.J.J., Lim, S.C., Lau, P. Y., Koh, K.T.S., Tan, E., Kairon, R.S., et al., (2022). Protist. guru: A Comparative Transcriptomics Database for Protists. *J. Mol. Biol.*, 167502. https://doi.org/10.1016/j.jmb.2022.167502.
5. Navin, Nicholas E., Rozenblatt-Rosen, O., Zhang, N.R., (2021). New frontiers in single-cell genomics. *Genome Res.* **11**, 313–316. https://doi.org/10.1101/gr.276129.121.
6. Grandi, F., Caroli, J., Romano, O., Marchionni, M., Forcato, M., Bicciato, S., (2022). popsicleR: A R Package for Pre-processing and Quality Control Analysis of Single Cell RNA-seq Data. *J. Mol. Biol.*, 167560. https://doi.org/10.1016/j.jmb.2022.167560.
7. Gralinska, E., Kohl, C., Sokhandan Fadakar, B., Vingron, M., (2022). Visualizing Cluster-specific Genes from Single-cell Transcriptomics Data Using Association Plots. *J. Mol. Biol.*, 167525. https://doi.org/10.1016/j.jmb.2022.167525.
8. Lin, D., Chen, Y., Negi, S., Cheng, D., Ouyang, Z., Sexton, D., Li, K., Zhang, B., (2022). Cell Depot: A Unified Repository for scRNA-seq Data and Visual Exploration. *J. Mol. Biol.*, 167425. https://doi.org/10.1016/j.jmb.2021.167425.
9. Shoombuatong, W., Basith, S., Pitti, T., Lee, G., Manavalan, B., Throne,, (2022). A New Approach for Accurate Prediction of Human RNA N7-Methyl-guanosine Sites. *J. Mol. Biol.*, 167549. https://doi.org/10.1016/j.jmb.2022.167549.
10. Scott, S., Hallwirth, C.V., Hartkopf, F., Grigson, S., Jain, Y., Alexander, I.E., Bauer, D.C., Wilson, L.O.W., et al., (2022). A Tool for Detecting Integration of Wild-Type Viruses and Clinical Vectors. *J. Mol. Biol.*, 167408. https://doi.org/10.1016/j.jmb.2021.167408.
11. Gaither, J., Lin, Y.H., Bundschuh, R., (2022). RBPBind: Quantitative Prediction of Protein-RNA Interactions. *J. Mol. Biol.*, 167515. https://doi.org/10.1016/j.jmb.2022.167515.
12. Hess, J.M., Jannen, W.K., Aalberts, D.P., (2022). The four mRNA bases have quite different (un)folding free energies, applications to RNA splicing and translation initiation with BindOligoNet. *J. Mol. Biol.*, 167578. https://doi.org/10.1016/j.jmb.2022.167578.
13. Young, R.T., Clauvelin, N., Olson, W.K., (2022). emDNA – A Tool for Modeling Protein-decorated DNA Loops and Minicircles at the Base-pair Step Level. *J. Mol. Biol.*, 167558. https://doi.org/10.1016/j.jmb.2022.167558.
14. Zhang, Y., Wang, J., Xiao, Y., (2022). 3dRNA: 3D Structure Prediction from Linear to Circular RNAs. *J. Mol. Biol.*, 167452. https://doi.org/10.1016/j.jmb.2022.167452.
15. Madeo, G., Savojardo, C., Luigi Martelli, P., Casadio, R., (2022). SVMyr: a web server detecting co- and post-translational myristoylation in proteins. *J. Mol. Biol.*, 167605. https://doi.org/10.1016/j.jmb.2022.167605.
16. Sanchez-Garcia, R., Macias, J.R., Sorzano, C.O.S., Carazo, J.M., Segura, J., (2022). BIPSPI+: Mining Type-Specific Datasets of Protein Complexes to Improve Protein Binding Site Prediction. *J. Mol. Biol.*, 167556. https://doi.org/10.1016/j.jmb.2022.167556.
17. Schneidman-Duhovny, D., Inbar, Y., Nussinov, R., Wolfson, H.J., (2005). PatchDock and {SymmDock}: servers for rigid and symmetric docking. *Nucleic Acids Res.* **33**, W363–W367. https://doi.org/10.1093/nar/gki481.
18. Choi, J., Park, T., Yul Lee, S., Yang, J., Seok, C., (2022). GalaxyDomDock: An Ab Initio Domain–domain Docking Web Server for Multi-domain Protein Structure Prediction. *J. Mol. Biol.*, 167508. https://doi.org/10.1016/j.jmb.2022.167508.
19. Egbert, M., Jones, G., Collins, M., Kozakov, D., Vajda, S., (2022). FTMove: A Web Server for Detection and Analysis of Cryptic and Allosteric Binding Sites by Mapping Multiple Protein Structures. *J. Mol. Biol.*, 167587. https://doi.org/10.1016/j.jmb.2022.167587.
20. Bell, E.W., Schwartz, J.H., Freddolino, P.L., Zhang, Y., Peppi,, (2022). Whole-proteome Protein-protein Interaction Prediction through Structure and Sequence Similarity, Functional Association, and Machine Learning. *J. Mol. Biol.*, 167530. https://doi.org/10.1016/j.jmb.2022.167530.
21. Helmy, M., Mee, M., Ranjan, A., Hao, T., Vidal, M., Calderwood, M.A., Luck, K., Bader, G.D., (2022). openPIP: An open-source platform for hosting, visualizing and analyzing protein interaction data. *J. Mol. Biol.*, 167603. https://doi.org/10.1016/j.jmb.2022.167603.
22. Malladi, S., Powell, H.R., David, A., Islam, S.A., Copeland, M.M., Kundrotas, P.J., Sternberg, M.J.E., Vakser, I.A., et al., (2022). A resource for mapping variants onto experimental and modeled structures of human protein complexes. *J. Mol. Biol.*, 167608. https://doi.org/10.1016/j.jmb.2022.167608.
23. Siva Shanmugam, N.R., Veluraja, K., Michael Gromiha, M., (2022). PCA-MutPred: Prediction of Binding Free Energy Change Upon Missense Mutation in Protein-carbohydrate Complexes. *J. Mol. Biol.*, 167526. https://doi.org/10.1016/j.jmb.2022.167526.
24. Nagpal, S., Singh, R., Taneja, B., Mande, S.S., (2022). MarkerML – Marker feature identification in metagenomic datasets using interpretable machine learning. *J. Mol. Biol.*, 167589. https://doi.org/10.1016/j.jmb.2022.167589.

25. Nagar, N., Ben Tal, N., Pupko, T., (2022). EvoRator: Prediction of Residue-level Evolutionary Rates from Protein Structures Using Machine Learning. *J. Mol. Biol.*, 167538. https://doi.org/10.1016/j.jmb.2022.167538.
26. Martin-Malpartida, P., Hausvik, E., Underhaug, J., Torner, C., Martinez, A., Macias, M.J., (2021). HTSDSF Explorer, A Novel Tool to Analyze High-throughput DSF Screenings: HTSDSF Explorer, a tool for DSF screenings. *J. Mol. Biol.*. https://doi.org/10.1016/j.jmb.2021.167372 167372.
27. Manavalan, B., Chandra Patra, M., (2022). MLCPP 2.0: An updated cell-penetrating peptides and their uptake efficiency predictor. *J. Mol. Biol.*, 167604. https://doi.org/10.1016/j.jmb.2022.167604.
28. Westbrook, J.D., Young, J.Y., Shao, C., Feng, Z., Guranovic, V., Lawson, C.L., Vallat, B., Adams, P.D., et al., (2022). PDBx/mmCIF Ecosystem: Foundational Semantic Tools for Structural Biology. *J. Mol. Biol.*, 167599. https://doi.org/10.1016/j.jmb.2022.167599.
29. Ramalli, S.G., Miles, A.J., Janes, R.W., Wallace, B.A., (2022). The PCDDB (Protein Circular Dichroism Data Bank): A Bioinformatics Resource for Protein Characterisations and Methods Development. *J. Mol. Biol.*, 167441. https://doi.org/10.1016/j.jmb.2022.167441.
30. Kim, S., Cheng, T., He, S., Thiessen, P.A., Li, Q., Gindulyte, A., Bolton, E.E., (2022). PubChem Protein, Gene, Pathway, and Taxonomy Data Collections: Bridging Biology and Chemistry through Target-Centric Views of PubChem Data. *J. Mol. Biol.*, 167514. https://doi.org/10.1016/j.jmb.2022.167514.
31. Hekselman, I., Kerber, L., Ziv, M., Gruber, G., Yeger-lotem, E., (2022). The Organ-Disease Annotations (ODiseA) database of hereditary. *J. Mol. Biol.*, 167619. https://doi.org/10.1016/j.jmb.2022.167619.
32. Ziv, M., Gruber, G., Sharon, M., Vinogradov, E., Yeger-Lotem, E., (2022). The TissueNet vol 3 Database: Protein-protein Interactions in Adult and Embryonic Human Tissue contexts. *J. Mol. Biol.*, 167532. https://doi.org/10.1016/j.jmb.2022.167532.
33. Petrov, I., Alexeyenko, A., (2022). EviCor: Interactive Web Platform for Exploration of Molecular Features and Response to Anti-cancer Drugs. *J. Mol. Biol.*, 167528. https://doi.org/10.1016/j.jmb.2022.167528.
34. Weinstein, J.N., Collisson, E.A., Mills, G.B., Shaw, K.R.M., Ozenberger, B.A., Ellrott, K., Shmulevich, I., Sander, C., et al., (2013). The Cancer Genome Atlas Pan-Cancer analysis project. *Nature Genet.* **45**, 1113–1120. https://doi.org/10.1038/ng.2764.
35. Barretina, J., Caponigro, G., Stransky, N., Venkatesan, K., Margolin, A.A., Kim, S., Wilson, C.J., Lehár, J., et al., (2012). The Cancer Cell Line Encyclopedia enables predictive modelling of anticancer drug sensitivity. *Nature* **483**, 603–607. https://doi.org/10.1038/nature11003.
36. Pazos, F., Chagoyen, M., Seoane, P., Ranea, J.A.G., (2022). CoMent: Relationships Between Biomedical Concepts Inferred From the Scientific Literature. *J. Mol. Biol.*, 167568. https://doi.org/10.1016/j.jmb.2022.167568.
37. Hartley, M., Kleywegt, G.J., Patwardhan, A., Sarkans, U., Swedlow, J.R., Brazma, A., (2022). The BioImage Archive – Building a Home for Life-Sciences Microscopy Data. *J. Mol. Biol.*, 167505. https://doi.org/10.1016/j.jmb.2022.167505.
38. Jumper, J., Evans, R., Pritzel, A., Green, T., Figurnov, M., Ronneberger, O., Tunyasuvunakool, K., Bates, R., et al., (2021). Highly accurate protein structure prediction with AlphaFold. *Nature* **596**, 583–589. https://doi.org/10.1038/s41586-021-03819-2.

Rita Casadio
Biocomputing Group, FABIT-University of Bologna, Bologna I-40126, Italy
E-mail address: rita.casadio@unibo.it
Edited by P. Wright
David H. Mathews
Department of Biochemistry & Biophysics and Center for RNA Biology, University of Rochester, Rochester, NY 14642, USA
E-mail address: David_Mathews@urmc.rochester.edu
Michael J. E. Sternberg *
Department of Life Sciences, Imperial College London, London SW7 2AZ, UK
E-mail address: m.sternberg@imperial.ac.uk
