# Supplementary material for: PDBx/mmCIF Ecosystem: Foundational Semantic Tools for Structural Biology
Source: J Mol Biol. Author manuscript; Available in PMC 2023 Jun 26. (PMC10292674; doi:10.1016/j.jmb.2022.167599)
Supplement: Article [file NIHMS1907597-supplement-Article.zip › EviCor--Interactive-Web-Platform-for-Exploration-of-Mole_2022_Journal-of-Mol.pdf]

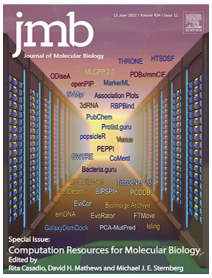

EviCor: Interactive Web Platform for Exploration of Molecular Features and Response to Anti-cancer Drugs

Iurii Petrov [1,2] and Andrey Alexeyenko [1,2,3]*

*1 - **Department of Microbiology,** Tumor and Cell Biology, Karolinska Institutet, Stockholm, Sweden*
*2 - **Science for Life Laboratory,** Box 1031, 17121 Solna, Sweden*
*3 - **Evi-networks,** enskild konsultföretag, Sweden*

Correspondence to Andrey Alexeyenko: *andrej.alekseenko@scilifelab.se (A. Alexeyenko), @AndreyAlexeyen1 (A. Alexeyenko)*
https://doi.org/10.1016/j.jmb.2022.167528
Edited by Michael Sternberg

Abstract

Experimental biologists are often left alone with the task to download, process, and analyze big datasets in order to perform correlation or other simpler analyses. To address these issues, we introduce EviCor, a handy toolbox for exploration of data from large public resources such as The Cancer Genome Atlas and The Cancer Cell Line Encyclopedia, complemented with follow-up information on same samples, which couples omics datasets with drug response profiles (https://www.evicor.org/). The data was processed for easy retrieval from the server-side database and includes pre-computed drug-feature correlation tables. Using information from multiple independent sources, the task-oriented web interface presents relations between phenotype, single-molecule, and pathway variables with graphical, statistical, and network analysis tools. Building custom multivariate models is enabled via user-friendly web interface and programmatic access via RESTinterface. Project code is available at https://github.com/aveviort/HyperSet.
© 2022 The Author(s). Published by Elsevier Ltd. This is an open access article under the CC BY license (http://creativecommons.org/licenses/by/4.0/).

Introduction

Cancer is one of the leading causes of death worldwide. Despite significant progress in understanding cancer biology as well as emergence of large-scale datasets for public use and advanced methods of diagnostics and treatment response prediction, we are still far from curing cancer completely. Partially, it can be explained by cancer heterogeneity: both patients and their tumors are unique. The clinical cancer research is further complicated by small sizes of cohorts and datasets, which makes most of the new findings poorly reproducible. Verification of identified correlates on independent datasets is an urgent need in modern biomedical research but is often overlooked because of absence of such data or difficulties in accessing it. Over a number of years, various projects generated molecular profiles of multiple clinical or in vitro samples as well as datasets describing the same samples phenotypically, such as drug sensitivity screens or immune cell composition. However, these sets remain dispersed across public resources in different machine-readable formats. For an experimentalist, the logistics of unifying such resources for the purpose of matching phenotypes to molecular profiles is often too costly. An example question would be: how does sensitivity to a certain drug correlate with a molecular feature, such as point mutation or protein expression? Answering via *in vitro* experiments would be expensive and time consuming. Further verification of the findings is even more challenging.[7,29]

0022-2836/© 2022 The Author(s). Published by Elsevier Ltd.This is an open access article under the CC BY license (http://creativecommons.org/licenses/by/4.0/).

While many online databases may already contain the desired information, studying associations between molecular correlates and e.g. drug response is limited to automated *in vitro* screens. Although agreement between such datasets was identified as a big issue,[13,12] respective web sites CellMiner,[21] GDSC,[30] or DepMap[6] present project-specific data without a possibility of comparison across datasets. There exist integrative public resources such as cBioPortal[5,10] with its specific focus on being a public data repository and wide possibilities for data exploration and retrieval, or the free version of OncoMine[22] with data access constraints, or PharmacoDB.[25] These still provide limited data exploration functionality and no higher order (multivariate, network, cross-validation etc.) analyses. None of such resources, up to our knowledge, contains the set of features offered by our new resource named EviCor: https://www.evicor.org/.

In addition to the ability to match drug resistance profiles from independent screens, EviCor provides access to clinical response data, namely patient survival upon administration of drugs in the clinic. More specifically, *in vitro* datasets contain information on many of the same drug treatments and molecular profiles as in clinical datasets, which enabled the long-desired opportunity to investigate consistency between pharmacological screens and clinical domains.

Further, there is a strong interest in pathway and network analysis of omics data, which should provide more systematic insight into mechanisms of drug response and its association with molecular features. Pathway-level variables were shown to also be more statistically efficient and reproducible compared to original, gene-level mutational and transcriptional data.[16,8] For generating, analyzing, and visualizing such profiles we employed a toolbox of network enrichment analysis (NEA).[1]

EviCor was designed to address these challenges and implements the following features:

1. Correlation analysis between single molecular or pathway features and drug response. These were pre-calculated off-line, followed by selection of most significant and reliable correlates which were deployed on the server side. The data is easy to retrieve according to user-defined constraints, navigate across, and investigate.
2. Access to network analysis, available both as pathway-level variable values to be used for multivariate models and via plots.
3. Creation of users' own multivariate models with a possibility to combine predictor variables of different data types.
4. Ability to retrieve, compare, and cross-validate findings of interest via access to molecular and phenotype variables from different, independently generated data sources.
5. Interactive data exploration and customization of output for minimum-effort integration into own research pipeline.
6. Access via documented REST API.
7. Detailed documentation and interactive demos (on the main page as well as at https://www.evicor.org/help/faq-evi/dr-faq.html=.

EviCor (Figure 1(A)) contains in its SQL database original data from TCGA- and CCLE-related data repositories as well as drug response and other complementing results on the same cell lines and patients which were published separately.[2,3,11,27,23,20] In addition, it stores pathway profiles from our network analysis as well as significant correlates based on all the data named above.

Results

Data sources

The original datasets (Supplementary Table 1) were retrieved from The Cancer Genome Atlas (TCGA)[4] and Cancer Cell Line Encyclopedia (CCLE). Further in the TCGA project, third party datasets emerged, such as the computation-based immune cell estimates.[27] These, together with updated TCGA clinical profiles[20] were added to our TCGA collection as immunoprofiles (IMMUNO). The CCLE component included large scale screens of cell line sensitivity to anti-cancer drugs, namely GDSC1, GDSC2,[15] and CTRPv2.0.[24]

The plots and tables using results of our network analysis provide web links to EviNet web site. While exploring such data, user clicks on specific sample data points open subnetwork views behind the respective sample's altered gene set (see detailed information in Methods for "NEA") and the pathway in question. A scheme of Network Enrichment Analysis (NEA) is presented in Figure 1(B). NEA not only produces basic scores for ranking (z-score) but also estimates statistical significance. However, in the analysis of associations for EviCor we used the z-scores because their close-to-Gaussian distribution is convenient both in statistical analyses and as sample coordinates for visualization.

Identification of significant correlates

Tab "Correlates of drug response" presents pre-computed results of correlation analyses between omics variables and response to specific drugs, detected with univariate or covariate linear models that included covariates. There are thousands significant correlates between single molecular features and response to different drugs (Supplementary Table 2). To retrieve correlations for TCGA or CCLE data sources, users can choose either all or specific, individual data type (GE, PE, MUT, etc.), cohort (if available), platform

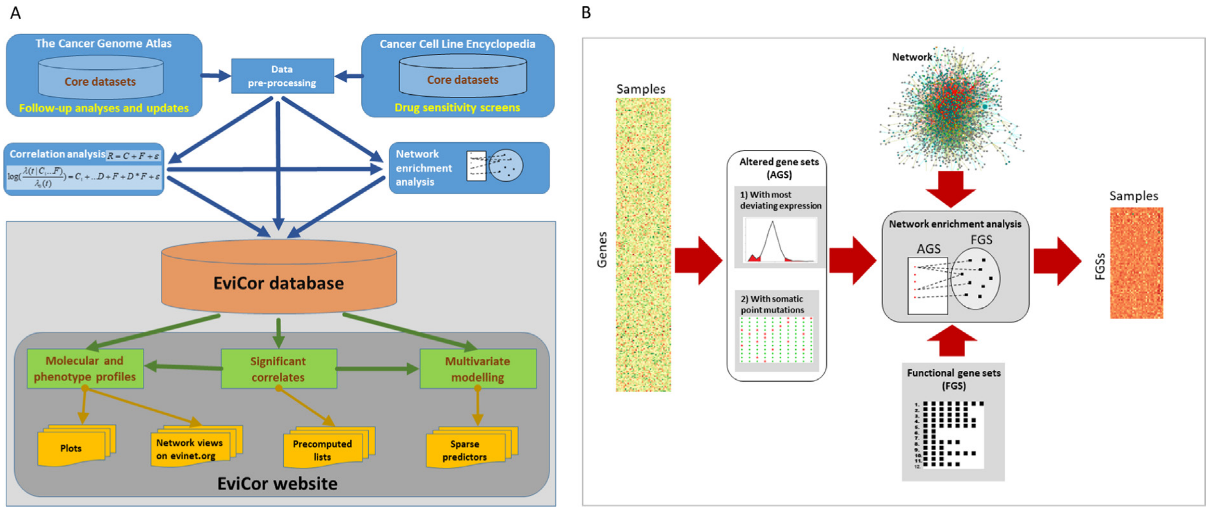

Figure 1. Structure of the web resource. (A) Data flow and main functionality of EviCor. Data is downloaded from public resources, pre-processed and uploaded to EviCor database, where it can be retrieved from for different purposes via either front-end or REST interfaces. (B) Scheme of network enrichment analysis.

(Agilent, or RNA-seq for mRNA expression, etc.), drug sensitivity screen (for CCLE), and identifier (drug, gene, protein, or pathway – depending on the platform). The table may be exported in different formats or copied to the clipboard. REST API (Application Programming Interface for REpresentational State Transfer, i.e. detailed URLs sent as requests from the user side) provides access to functionality of this tab and extends it by enabling more flexible retrieval criteria.

The table of retrieved correlations is sortable by individual columns, while the search box enables additional free text filtering. External information for individual entries is available via links to GeneCards, PubChem, MSigDB, WikiPathways, and KEGG databases. Desired level of significance can be defined by q-value thresholds which estimate false discovery rate calculated from relevant *p*-values.[26] In TCGA the threshold is applied to either feature or interaction term and in CCLE to the feature term in either univariate or covariate model. Then patterns most interesting for further investigation can be identified by sorting and filtering the output table.

It is known that CCLE collection combines cell lines from dozens different origin sites of primary tumors, which strongly affects cell lines' molecular features, such as gene expression.[11,2] In a correlation analysis, this might obscure truly existing correlations or falsely present spurious ones (which would then be due to cell origin rather than the seemingly correlated feature). Therefore in our CCLE analysis, the site (or tissue) of original tumor was used as a covariate. The linear model allowed to account for its effect and reduce significantly the number of identified drug-feature correlates, focusing on patterns that arise specifically due to molecular features rather than from cell line origin. As an example, erlotinib is known to interact with EGFR, suppress its activity in cancer tissues[28] and be most efficient against tumors with EGFR mutations.[18] In a straightforward 1-way linear model without the covariate, the relation between sensitivity to erlotinib and NEA score for EGFR signaling pathway would be insignificant ($p = 0.19$, red dotted line in Figure 2(A)). However, accounting for the covariate revealed a significant net effect of the EGFR signaling ($p = 5.5 * 10^{-13}$) as illustrated with regression lines for the largest groups of origin.

TCGA datasets included clinical variables (see Methods). While these were employed as covariates in order to account for relevant effects, only three *p*-values of interest are used for filtering: main effects of drug application and molecular feature as well as the effect of their interaction.

Sorting the output by relevant *q*-values can help to find specific correlation patterns. For example, dependence between administration of doxorubicin and overall patient survival in BRCA cohort was significant ($p_{drug} = 6.0 * 10^{-09}$) but did not depend on the mRNA expression of PGAM2 ($p_{feature} = 0.15$) (Figure 2(B)). On the KM plot, this appears as higher positions of the solid curves (drug applied) compared to the dotted curves (no drug). On the contrary, overall survival was not significantly associated with administration of gemcitabine in general ($p_{drug} = 0.50$) but depended on expression of ZNF541 ($p_{feature} = 4.5 * 10^{-04}$), hence the green curves are lower than the red curves (Figure 2(C)). The most interesting category can be detected via the interaction term. For example, in LUSC cohort the patients who received cisplatin (solid lines) strongly differed by survival depending on the levels of GPLD1. However, in the rest of the cohort GPLD1

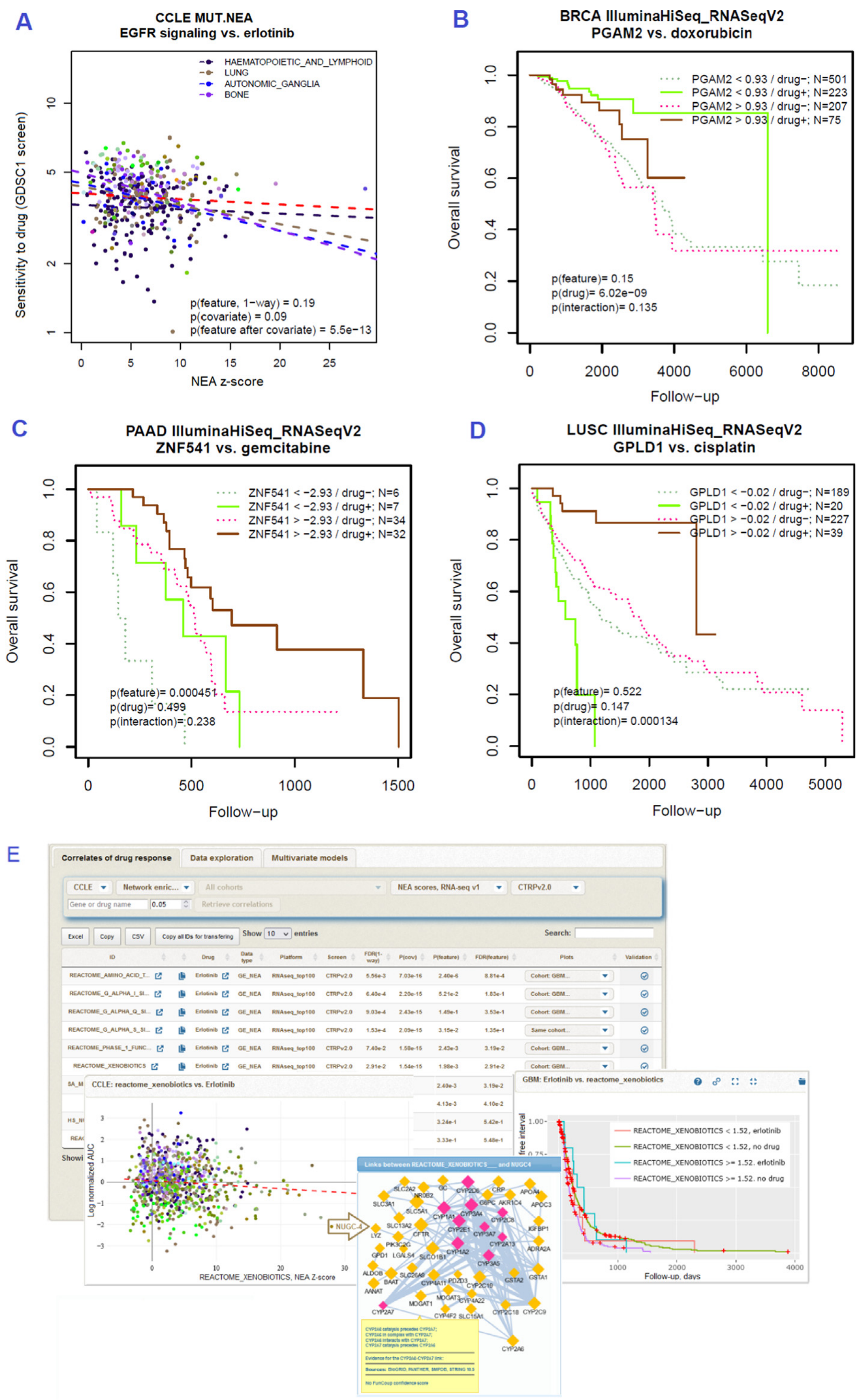

Figure 2. Discovery and analysis of single-feature correlates. (A) Relation between NEA z-scores for Reactome pathway "EGFR signaling" and sensitivity to erlotinib in large-scale drug screen GDCS1 using CCLE collection. Dotted lines are linear regression fits of all (red) and subsets of (other colors) the cancer cell lines. *P*-values represent linear models without and with covariate (line 1 vs. lines 2,3 respectively). (B, C, D) Differential survival of patients in TCGA cohort depending on mRNA expression levels in primary tumor samples. Upper right corner: sample strata according to gene expression (> or < the cutoff) versus drug administered or not (+/−). Bottom left corner: *p*-values from the linear model Cox-TCGA (*p*-values for covariate terms are not shown). (E) Screenshots of tabular view and visualization of correlates. NEA Z-scores for Reactome "Xenobiotics" pathway correlated with sensitivity to erlotinib in the *in vitro* screen CTRPv2.0. Similarly, progress-free interval in glioblastoma patients who received erlotinib could also be differentiated by "Xenobiotics" Z-score. The sub-network view for one relatively resistant cell line, NUGC-4 visualizes known functional relations between the cell line genes (yellow) and the pathway genes (magenta).

expression levels were irrelevant for survival (the dotted curves are close to each other). This was reflected by the *p*-values: the main effects were insignificant (p_{drug} = 0.147 and $p_{feature}$ = 0.52), whereas significance of the interaction was stronger ($p_{interaction}$ = 1.3^{-04}) (Figure 2(D)). Potentially, given this information available in advance the patients could had been stratified according to the GPLD1 level in order to avoid useless cisplatin treatment.

We earlier demonstrated a possibility to trace *in vitro* correlates discovered in CCLE to clinical outcomes of drug application.[8] The retrieved table of significant correlations between molecular features and drug response presents numeric estimates of significance and links to relevant plots. When a significant match between same molecular correlate and same drug was identified in a different *in vitro* and/or clinical dataset (three drug screens and ten TCGA cohorts in total), the retrieved correlates are accompanied by links to plots that visualize the correlation and to sub-network view, if relevant (Figure.2(E)).

Exploring and visualizing original data

All the pre-processed and stored data profiles are available via “Data exploration” tab and can be viewed as interactive plots of different types, presenting up to three variables at once (the 3rd pseudo-dimension would be represented by color and/or marker shape). The graph types (with additional statistical metrics displayed) include bar plot, histogram, pie chart, scatter plot, Venn diagram, boxplot, as well as Kaplan-Meier plots for survival data. To prevent user errors while selecting variables to be plotted, the choice of plot types in the menus is driven by already chosen data type combinations.

Sample subsets can be selected via menu box “Sample code”, which contains both original sample codes (tissue/organ of origin in CCLE and primary, metastatic, and normal samples in TCGA) and meta-codes for their unions (“any cancer”, “all”, etc.). While pre-processing, most of the data values have been rendered to have distributions as close to Gaussian or binomial as possible. In addition to this original scale, continuous variables can also be plotted upon logarithmic or square root transformations.

Plots are created in R using `plotly` framework,[14] which enables high interactivity. For example, point markers in scatter plots are clickable and show sample information. For NEA-based variables, the clicks retrieve sample-specific subnetwork views. In charts with different categories, the latter can be de-selected by mouse clicks. Most of the plots can be zoomed without loss of quality. In order to save computational resources and access time, the plots are stored on the server for one month and, if the user would order exactly the same plot again or open it from the archive, can be quickly retrieved. Otherwise, a plot can be shared or embedded into other websites via button “Get link” which would generate a URL and enable permanent storage on the server. There is no limit on amount of created and shared plots. Saving data for each generated plot in its respective plot file guarantees regenerated plots to always look the same, even if database data was updated.

Multivariate models

A number of publications dedicated to large drug screens presented multivariate models of drug resistance via combining single-gene molecular correlates.[3,2,8] This approach is equally popular in small-scale experimental studies, although might suffer from lack of data. Building reliable models is nonetheless challenging and requires properly designed validation strategies. In order to demonstrate potential sensitivity of multivariate models and provide access to alternative datasets suitable for independent validation, we implemented functionality for quick and interactive building of predictive models. A good model should combine simplicity (few variables), precision (low error rates), and reproducibility (ability to work equally well on novel datasets). The state-of-the-art approach to building multivariate models from omics data is to use sparse regression, which assumes that a full set of variables when $N \gg p$ would be most of the times redundant, resulting in overfitting and irreproducibility. Therefore, as the back-end engine we employed the popular lasso/ridge regression toolbox provided by R package `glmnet`.[9] Since all the tabs share the same scope of datasets and variables, this can be done by e.g. investigating the pre-computed correlation tables in tab “Correlates of drug response” and then pasting selected gene or pathway IDs from an internal clipboard into textbox “Independent variables” in tab “Multivariate models”. The interface also allows manual input or including all available IDs.

Data usability in the models varies between the variable types: some may be only used as response (dependent) variables, some only as predictors (independent variables), while some in both roles. Using drop-down menus, up to three different data types (or platforms of the same datatype) can be combined into a set of predictive features. In every case, the model would be built by the `glmnet` algorithm which attempts to create sparsity in the initial set of potential predictors.

The options can be classified as:

- Universal: selector for data source and cohort to be used for variable selection.
- Dependent variable: drop-down menus for response datatype and cohort. A multi-selector allows filtering for desirable samples (sample barcodes or meta-codes in TCGA and tissue/organ of primary tumor or meta-code in CCLE). This option applies to cohort-level variables, such as patient survival. If,

on the contrary, the chosen platform is entry-based (such as gene ID), then a special field enables selecting a particular ID and e.g. modeling its gene expression.

- Independent variables: drop-down menus of data type and platform for predictors. For entry-based platforms an unlimited number of IDs can be entered via autocomplete, pasted as a list or chosen altogether using keyword “all”.
- GLMnet parameters: all options related to model building.

1. Standardize: if independent variables should be standardized with mean = 0 and SD = 1.
2. Alpha: the mixing elastic net parameter, determining balance between lasso and ridge regression (alpha = 0 corresponds to pure ridge regression, alpha = 1 is pure lasso).
3. Number of lambda steps to be tested.
4. Lambda minimal ratio: a shrinkage coefficient, 0 means no shrinkage.
5. Family: distribution type for the response variable. At the moment, options “cox”, “binomial”, and “gaussian” are supported.
6. Model validation checkbox for a) number of folds for cross-validation (test vs. training sets inside `cv.glmnet` by sampling with replacement) and b) percentage of samples from the initial cohort retained for the final, independent cross-validation.

When the model is created, results are presented (Figure 3(D)) as:

- A graphical interpretation of model performance.
- Overview of model parameters (sample sizes $n_{training}$ and n_{test}, number of variables in model k, cross-validation folds, chosen family and alpha) as well as area under ROC curve (when applicable), classification accuracy (when applicable) and evaluation with information criteria (AIC and BIC).
- A graphical representation of agreement between observed and predicted response for the training and test (if chosen) sets.

(see Supplementary File 1 for the whole list of available metrics).

The variables in the model are listed with their coefficients, so that each can be plotted individually against the response variable (Figure 3(D)). Models can be exported in RData format. Model comparison is allowed for models of the same type. Due to the stochastic nature of cross-validation, certain variability is possible between repeatedly, from run to run, generated models, i.e. different sets of variables and variation of their coefficients.

We tested if using variable selection with our pre-computed results from “Correlates of drug response” tab would be advantageous over the “default” `glmnet` approach of starting from the full feature space, such as all gene expression profiles. Although AIC and BIC values did not differ between these two scenarios, the “full” ones tended to accumulate higher residual sums of squares and were poorer reproducible in terms of correlation between predicted and observed values when tested on retained 50% sets (Figure 3(A)). It can be also observed that inclusion of NEA-based pathway scores was superior to using gene expression only and would be particularly advantageous when the feature space is limited to only significant correlates (Figure 3(B)). As an example, sensitivity to tozasertib was explained by two alternative models, one using only GE data type and the other allowing both GE and NEA features. In the latter case, the rank correlation between actual sensitivity and model predictions was somewhat superior for GE model on the training sets, but inferior on the retained test set (Figure 3(C)).

In certain situations, a user may want to quickly create multiple models such as for testing different options, parameters, finding an optimal model or collecting statistics. In this case, using EviCor REST API might be helpful. A few batch job parameters must be specified:

- Number of iterations: how many models should be created with same parameters.
- Name of statistics file, to be used to accumulate information on the created models and links to relevant files.
- Email address for the notification to be sent after the job completion.

EviCor batch jobs use a queue system, so that only one job can be active at a time, which may result in postponing a job after assignment. Also, only one job per user may be queued at a time.

Discussion

EviCor possesses several valuable and often unique features, the combination of which, up to our knowledge, is not provided by any other resource. Molecular variables from the largest cancer-related *in vitro* and clinical data collections can be accessed and combined in a straightforward and seamless way. Data from various omics platforms and alternative sources can be matched to phenotype or clinical variables for the same samples. In addition to presenting and visualizing data from the original sources, we provided results of rather simple but systematic, full-scale statistical analyses – computing which would be even less feasible for most of the users. This enables quick exploration of associations between molecular variables and response to anti-cancer drugs, with the possibility to match results from different screens and platforms. The analysis is complemented with pathway-level features from network analysis, which was shown to be a

winning approach because of deeper functional insight and superior sensitivity and reproducibility of drug response correlates.

The interactive functionality for building multivariate models would be convenient for exploratory and didactic purposes. The web-platform is highly interactive, simple, and allows sharing and exporting results for further analysis or publication. Of course, the original clinical data do not represent systematic randomized trials and therefore the results should be interpreted with care. Users who would build multivariate models will also discover that seemingly efficient predictors very often (or even mostly) fail on

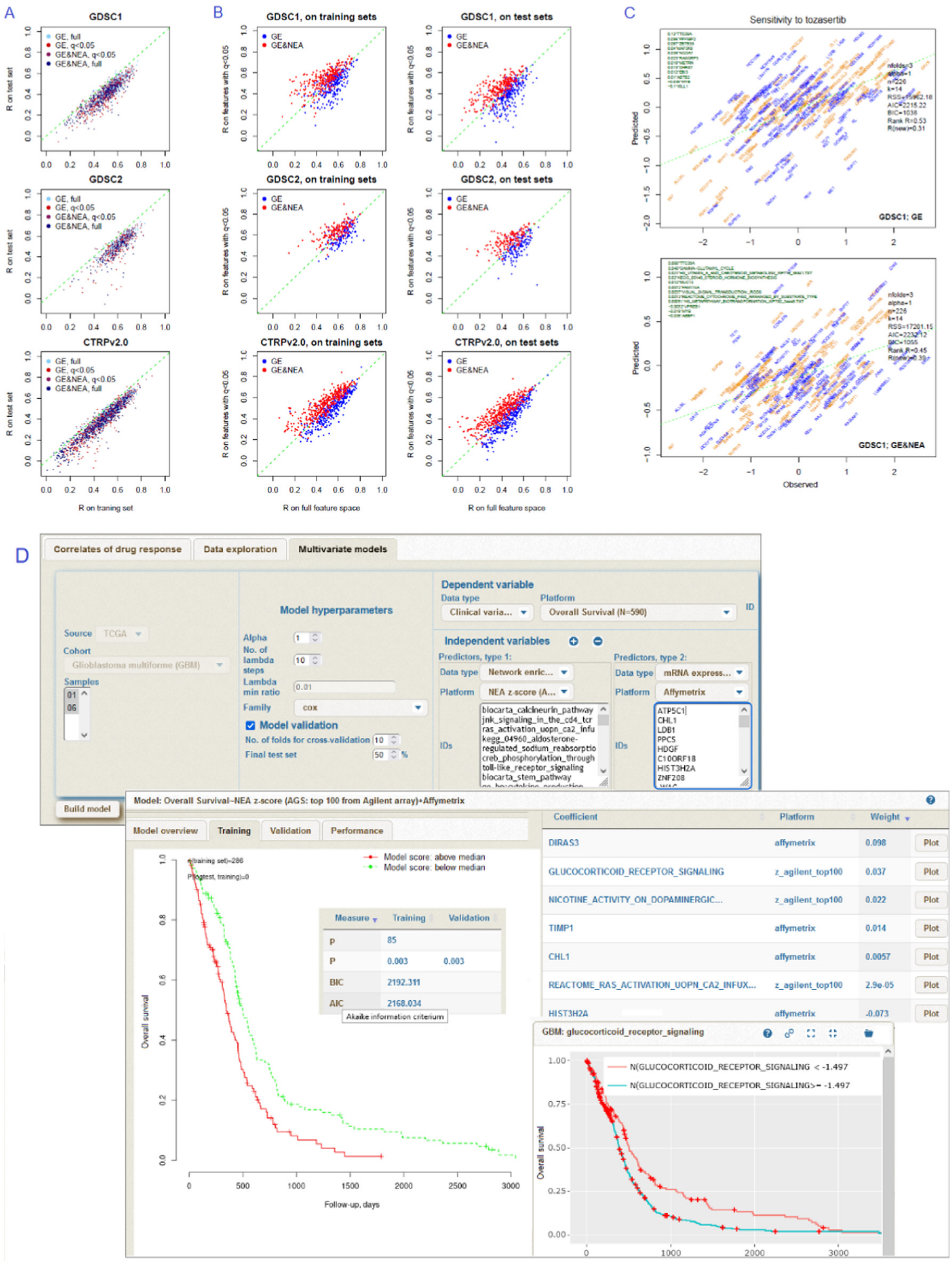

isolated test sets. EviCor should therefore be used for exploratory and pedagogical purposes rather than as a clinical discovery tool.

EviCor architecture was created with scalability in mind. We plan adding alternative machine learning methods and expanding the data collection following release and careful evaluation of novel sets and data types. Introducing additional clinical cohorts would be possible given their size enables sufficient statistical power for discovering drug response correlates.

Methods

Web server architecture and code availability

EviCor is an open-source project consisting of frontend and backend. The user interface is implemented in the form of tabs representing main functional sections. A number of interactive demos demonstrate most important functionality and are executed in a step-by-step manner, accompanied by comments. The frontend part uses HTML as well as code written in JavaScript (JS) with jQuery. Using Ajax, jQuery UI, datatables. net and plotly libraries ensures best user experience and compatibility with major browsers. The backend contains an SQL database with interface between JS and SQL used for data retrieval and formatting, which is implemented in Perl. Database operations involve either plain SQL queries or PostgreSQL procedural language (PL/pgSQL) stored procedures. Server-side scripts for plot generation and multivariate modeling are written in R language with the addition of widely supported third-party libraries, which enabled implementation of a variety of statistical methods.

Both frontend and backend parts are modular. Most of the modules may be used in an independent way, given that the modules' dependencies are met for correct operational mode. All required third-party packages are available through public R (CRAN, BioConductor), Perl (CPAN), and Linux repositories or websites (jQuery, datatables.net). Project code is available on GitHub under modified BSD license at https://github.com/aveviort/HyperSet.

Cookies and session storage keep user settings, such as latest menu choices or history of created plots for quick retrieval. Anonymized reports on data processing errors may be recorded for the purpose of debugging and further web site development.

REST API

EviCor web server offers REST API for all major functions, so that

1. Lists of correlates are retrieved in JSON format.
2. Plots can be created using REST interface, which returns both html pages and JSON code containing query parameters and values of accompanying statistics.
3. REST API for building multivariate models returns the model's name, using which a user can then retrieve the model (in RData file format) as well as linear coefficients (in JSON format), performance metrics (either JSON or CSV format), and a graphical model overview accompanied with results of model training and validation.
4. API for multivariate model building in a batch mode. Users can order creating multiple models. A web link to the results would be sent to the specified email address.

REST API is described in EviCor help pages under help icon "?" in the upper right corner. Alternatively, JS interfaces provided in the libraries can be used, which would require fewer parameters (see references in interactive help on the site).

Data download and processing

The cancer cell lines datasets were obtained from the following online sources:
CTRP v2.0[24] from
http://portals.broadinstitute.org/ctrp.v2.0/
and GDSC1 and GDSC2 datasets[15] from
https://www.cancerrxgene.org/downloads/bulk_download
https://cellmodelpassports.sanger.ac.uk/downloads
The TCGA[4] data were obtained via
https://portal.gdc.cancer.gov/
For downstream analyses and visualization, the datasets were rendered as follows:
Point mutations (MUT) by default represented as binary variables `Mutation OR Wild type` regardless of predicted functional consequences available from MAF files or elsewhere. Distribution of mutation types for specific genes in each cohort is available in "Data exploration" tab.
Gene copy number (CNA) values CN were presented as `log2 (CN)` (if not already done so in the original resources).
mRNA expression (GE) values from RNA-seq and Affymetrix were log-transformed (if not done so in the original resources).
Methylation (METH) beta values were transformed to M-values (a.k.a. logit units)
`log2(beta / (1 - beta))`.

Pre-calculated correlations for patients and cells

For "Correlates of drug response" tab, we estimated significance of statistical associations between gene features and cell line sensitivity to drugs in both 1-way ANOVA model (R package `base`):
`anova(lm(Sensitivity ~ Feature));` (model CCLE-1)

Figure 3. Multivariate modeling with EviCor. We performed a comparative analysis of alternative feature spaces to be used for modeling: 1) based on either RNA-seq gene expression data (GE) or a combination of both GE and NEA pathway scores and 2) either full or limited to only significant correlates (*q*-values below 0.05 in the linear model with covariate "organ or tissue of origin"), labeled "full" vs. "$q < 0.05$", respectively. Model performance was estimated with rank correlation on either training or test sets, each including 50% of all samples. Separate analyses were performed for drug screening datasets GDSC1, GDSC2, and CTRP v. 2.0. (A) Expectedly, correlation values on the test sets were lower than on the training sets. (B) Given the same drug, correlation values for GE&NEA models were higher when the feature space was limited to variables with $q < 0.05$ compared to the full space (red points). GE-only models exhibited the opposite tendency. This could be observed on both training and test sets. (C) Example models were built from the "$q < 0.05$" feature spaces. Model terms are given in green, sorted by coefficient values. GE-only model (upper pane, 12 gene expression terms) performed slightly better on the training set, whereas GE&NEA model (lower pane, 6 gene expression and 6 pathway-level terms) was superior on the test set.

and a 2-way model with a covariate:
`anova(lm(Sensitivity ~ Tissue + Feature))`; (model CCLE-2)
using variables:
`Feature`: an original molecular variable (MUT, CNA, GE, METH) or NEA score for the given gene or pathway;
`Tissue`: a covariate for organ or tissue of origin of the cancer cell line;
`Sensitivity`: resistance to the specified drug.
The *p*-values for `Feature` were of main interest in this analysis. For presentation in the first tab we adjusted them for multiple testing by Benjamini-Hochberg method.
In TCGA datasets, associations between molecular features and patient survival given a certain drug were estimated as:
`coxph(Surv(Time,Status) ~ [Tumor_stage] + [IHC] + Feature + Drug + Feature * Drug)` (model Cox-TCGA)
using variables:
`Feature`: an original molecular variable (MUT, CNA, GE, METH) or NEA score for the given gene;
`Drug`: binary variable: if the drug was administered to the given patient;
`[IHC]`: TCGA immunohistochemistry parameters (whichever available for the cohort), i.e.
`er_status_by_ihc`, `pr_status_by_ihc`, `her2_status_by_ihc`;
`[Tumor_stage]`: one of
`Karnofsky_score` (GBM);
`Gleason_score` (PRAD);
`AJCC_pathologic_tumor_stage` (rest of the cohorts).

NEa

The algorithm of NEA and R package NEArender were described elsewhere[1,16] and is accompanied with standard documentation for offline use. Interactive online platform EviNet[17] can perform NEA by user's request. In the present application however, it was used for visualizing the pre-calculated NEA results.
To perform NEA, three components are required:

1. Network – a version of global network of functional coupling between human genes and/or proteins.
2. Altered gene sets (AGS) – a collection of sample specific gene lists for the whole cohort.
3. Functional gene sets (FGS) – a list of genes with a well-defined functional role, most often pathway. We used a set of 1655 canonical pathways as provided by MSigDB[19] (collection C2, October 2018).

EviCor provides network analysis and visualization of CCLE and TCGA sample using two NEA-derived data types: NEA-GE and NEA-MUT. Therefore, at *step 1* each cell line or patient sample was characterized with an AGS: a gene list of type GE or MUT. The GE data was used from the available cohort data sets:

- Agilent platform for OV and GBM;
- Illumina HiSeq RNASeq v.2 platform for the other TCGA cohorts.

The AGS of type NEA-GE were compiled as sample-specific lists of top *N* genes ($N = 100$), normalized mRNA expression of which was most different from the respective cohort mean, using function `samples2ags(..., method = "topnorm")` from R package `NEArender`.
Point mutation data were used as is, i.e. all genes reported as mutated in each cancer sample genome were presented as AGSs of type NEA-MUT despite of mutation type.
At *step 2*, network enrichment scores were calculated by considering individual gene-gene network edges between each of the sample-specific AGS (n = [No. of samples]) and each FGS ($p = 1655$), thus producing a pXn matrix of NEA Z-scores. This matrix was further used for statistical analysis and is available for user requests in the same way as matrices of any original molecular variables, in which p would then be the number of genes.

CRediT authorship contribution statement. **Iurii Petrov:** Conceptualization, Data curation, Formal analysis, Methodology, Software, Visualization, Writing – original draft, Writing – review & editing. **Andrey Alexeyenko:** Conceptualization, Data curation, Formal analysis, Funding acquisition, Investigation, Methodology, Resources, Supervision, Validation, Writing – original draft, Writing – review & editing.

DATA AVAILABILITY

No data was used for the research described in the article.

Acknowledgements

The EviCor website uses data generated by the TCGA Research Network: https://www.cancer.gov/tcga. The authors thank Swedish Research Council (Vetenskapsrådet) for financial support.

Appendix A. Supplementary Data

Supplementary data to this article can be found online at https://doi.org/10.1016/j.jmb.2022.167528.

Received 30 November 2021;
Accepted 1 March 2022;
Available online 5 March 2022

Keywords:
cancer;
drugs;
correlation;
visualization;
multivariate model;
network enrichment analysis

Abbreviations:
NEA, network enrichment analysis; AGS, altered gene set; FGS, functional gene set; KM, Kaplan Meier

References

1. Alexeyenko, A., Lee, W., Pernemalm, M., Guegan, J., Dessen, P., Lazar, V., Lehtiö, J., Pawitan, Y., (2012). Network enrichment analysis: extension of gene-set enrichment analysis to gene networks. *BMC Bioinformatics* **13**, 226.
2. Barretina, J., Caponigro, G., Stransky, N., Venkatesan, K., Margolin, A.A., Kim, S., Wilson, C.J., Lehár, J., et al., (2012). The Cancer Cell Line Encyclopedia enables predictive modelling of anticancer drug sensitivity. *Nature* **483**, 603–607.
3. Basu, A., Bodycombe, N.E., Cheah, J.H., Price, E.V., Liu, K., Schaefer, G.I., Ebright, R.Y., Stewart, M.L., et al., (2013). An interactive resource to identify cancer genetic

and lineage dependencies targeted by small molecules. *Cell* **154**, 1151–1161.

4. Cancer Genome Atlas Research Network, (2008). Comprehensive genomic characterization defines human glioblastoma genes and core pathways. *Nature* **455**, 1061–1068.
5. Cerami, E., Gao, J., Dogrusoz, U., Gross, B.E., Sumer, S.O., Aksoy, B.A., Jacobsen, A., Byrne, C.J., et al., (2012). The cBio Cancer Genomics Portal: An Open Platform for Exploring Multidimensional Cancer Genomics Data. *Cancer Discov.* **2**, 401–404.
6. Corsello, S.M., Nagari, R.T., Spangler, R.D., Rossen, J., Kocak, M., Bryan, J.G., Humeidi, R., Peck, D., et al., (2020). Discovering the anticancer potential of non-oncology drugs by systematic viability profiling. *Nature Cancer* **1**, 235–248.
7. Domcke, S., Sinha, R., Levine, D.A., Sander, C., Schultz, N., (2013). Evaluating cell lines as tumour models by comparison of genomic profiles. *Nature Commun.* **4**, 2126.
8. Franco, M., Jeggari, A., Peuget, S., Böttger, F., Selivanova, G., Alexeyenko, A., (2019). Prediction of response to anti-cancer drugs becomes robust via network integration of molecular data. *Sci. Rep.* **9**, 2379.
9. Friedman, J.H., Hastie, T., Tibshirani, R., (2010). Regularization Paths for Generalized Linear Models via Coordinate Descent. *J. Stat. Softw.* **33**, 1–22.
10. Gao, J., Aksoy, B.A., Dogrusoz, U., Dresdner, G., Gross, B., Sumer, S.O., Sun, Y., Jacobsen, A., et al., (2013). Integrative analysis of complex cancer genomics and clinical profiles using the cBioPortal. *Sci. Signal.* **6**, pl1.
11. Garnett, M.J., Edelman, E.J., Heidorn, S.J., Greenman, C.D., Dastur, A., Lau, K.W., Greninger, P., Thompson, I.R., et al., (2012). Systematic identification of genomic markers of drug sensitivity in cancer cells. *Nature* **483**, 570–575.
12. Ghandi, M., Huang, F.W., Jané-Valbuena, J., Kryukov, G.V., Lo, C.C., McDonald, E.R., Barretina, J., Gelfand, E.T., et al., (2019). Next-generation characterization of the Cancer Cell Line Encyclopedia. *Nature* **569**, 503.
13. Haibe-Kains, B., El-Hachem, N., Birkbak, N.J., Jin, A.C., Beck, A.H., Aerts, H.J.W.L., Quackenbush, J., (2013). Inconsistency in large pharmacogenomic studies. *Nature* **504**, 389–393.
14. Inc, P.T., (2015). Collaborative data science. Plotly Technologies Inc., Montreal, QC.
15. Iorio, F., Knijnenburg, T.A., Vis, D.J., Bignell, G.R., Menden, M.P., Schubert, M., Aben, N., Gonçalves, E., et al., (2016). A Landscape of Pharmacogenomic Interactions in Cancer. *Cell* **166**, 740–754.
16. Jeggari, A., Alexeyenko, A., (2017). NEArender: an R package for functional interpretation of 'omics' data via network enrichment analysis. *BMC Bioinformatics* **18**
17. Jeggari, A., Alekseenko, Z., Petrov, I., Dias, J.M., Ericson, J., Alexeyenko, A., (2018). EviNet: a web platform for network enrichment analysis with flexible definition of gene sets. *Nucleic Acids Res.* **46**, W163–W170.
18. Kobayashi, K., Hagiwara, K., (2013). Epidermal growth factor receptor (EGFR) mutation and personalized therapy in advanced nonsmall cell lung cancer (NSCLC). *Target. Oncol.* **8**, 27–33.
19. Liberzon, A., Subramanian, A., Pinchback, R., Thorvaldsdóttir, H., Tamayo, P., Mesirov, J.P., (2011). Molecular signatures database (MSigDB) 3.0. *Bioinformatics* **27**, 1739–1740.
20. Liu, J., Lichtenberg, T., Hoadley, K.A., Poisson, L.M., Lazar, A.J., Cherniack, A.D., Kovatich, A.J., Benz, C.C., et al., (2018). An Integrated TCGA Pan-Cancer Clinical Data Resource to Drive High-Quality Survival Outcome Analytics. *Cell* **173**, 400–416.e11.
21. Reinhold, W.C., Sunshine, M., Liu, H., Varma, S., Kohn, K.W., Morris, J., Doroshow, J., Pommier, Y., (2012). Cell Miner: A Web-Based Suite of Genomic and Pharmacologic Tools to Explore Transcript and Drug Patterns in the NCI-60 Cell Line Set. *Cancer Res.* **72**, 3499–3511.
22. Rhodes, D.R., Yu, J., Shanker, K., Deshpande, N., Varambally, R., Ghosh, D., Barrette, T., Pander, A., et al., (2004). ONCOMINE: A Cancer Microarray Database and Integrated Data-Mining Platform. *Neoplasia* **6**, 1–6.
23. Saltz, J., Gupta, R., Hou, L., Kurc, T., Singh, P., Nguyen, V., Samaras, D., Shroyer, K.R., et al., (2018). Spatial Organization and Molecular Correlation of Tumor-Infiltrating Lymphocytes Using Deep Learning on Pathology Images. *Cell Rep.* **23**, 181–193.e7.
24. Seashore-Ludlow, B., Rees, M.G., Cheah, J.H., Cokol, M., Price, E.V., Coletti, M.E., Jones, V., Bodycombe, N.E., et al., (2015). Harnessing Connectivity in a Large-Scale Small-Molecule Sensitivity Dataset. *Cancer Discov.* **5**, 1210–1223.
25. Smirnov, P., Kofia, V., Maru, A., Freeman, M., Ho, C., El-Hachem, N., Adam, G.-A., Ba-alawi, W., et al., (2018). PharmacoDB: an integrative database for mining in vitro anticancer drug screening studies. *Nucleic Acids Res.* **46**, D994–D1002.
26. Storey, J.D., Tibshirani, R., (2003). Statistical significance for genomewide studies. *Proc. Natl. Acad. Sci. U. S. A.* **100**, 9440–9445.
27. Thorsson, V., Gibbs, D.L., Brown, S.D., Wolf, D., Bortone, D.S., Ou Yang, T.-H., Porta-Pardo, E., Gao, G.F., et al., (2018). The Immune Landscape of Cancer. *Immunity*.
28. Tsien, C.I., Nyati, M.K., Ahsan, A., Ramanand, S.G., Chepeha, D.B., Worden, F.P., Helman, J.I., D'Silva, N., et al., (2013). The effect of erlotinib on EGFR and downstream signaling in oral cavity squamous cell carcinoma. *Head Neck* **35**, 1323–1330.
29. Vanden Heuvel, J.P., Maddox, E., Maalouf, S.W., Reproducibility Project: Cancer Biology, (2018). Replication Study: Systematic identification of genomic markers of drug sensitivity in cancer cells. *ELife* **7**, e29747
30. Yang, W., Soares, J., Greninger, P., Edelman, E.J., Lightfoot, H., Forbes, S., Bindal, N., Beare, D., et al., (2013). Genomics of Drug Sensitivity in Cancer (GDSC): a resource for therapeutic biomarker discovery in cancer cells. *Nucleic Acids Res.* **41**, D955–D961.
