# Supplementary material for: PDBx/mmCIF Ecosystem: Foundational Semantic Tools for Structural Biology
Source: J Mol Biol. Author manuscript; Available in PMC 2023 Jun 26. (PMC10292674; doi:10.1016/j.jmb.2022.167599)
Supplement: Article [file NIHMS1907597-supplement-Article.zip › RBPBind--Quantitative-Prediction-of-Protein-RNA-_2022_Journal-of-Molecular-B.pdf]

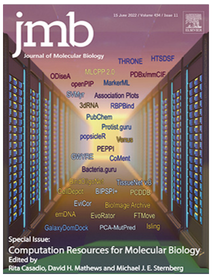

RBPBind: Quantitative Prediction of Protein-RNA Interactions

Jeff Gaither [1†], **Yi-Hsuan Lin** [2†‡] **and Ralf Bundschuh** [3*]

*1 - **Institute for Genomic Medicine,** Nationwide Children's Hospital, 700 Children's Dr, Columbus, OH 43205, USA*
*2 - **Department of Physics,** The Ohio State University, 191 West Woodruff Av, Columbus, OH 43210, USA*
*3 - **Department of Physics,** Department of Chemistry & Biochemistry, Division of Internal Medicine, Center for RNA Biology, The Ohio State University, 191 West Woodruff Av, Columbus, OH 43210, USA*

Correspondence to Ralf Bundschuh: *bundschuh@mps.ohio-state.edu (R. Bundschuh), @JeffBGaither (J. Gaither)*
https://doi.org/10.1016/j.jmb.2022.167515
Edited by David Mathews

Abstract

There are hundreds of RNA binding proteins in the human genome alone and their interactions with messenger and other RNAs in a cell regulate every step in an RNA's life cycle. To understand this interplay of proteins and RNA it is important to be able to know which protein binds which RNA how strongly and where. Here, we introduce RBPBind, a web-based tool for the quantitative prediction of the interaction of single-stranded RNA binding proteins with target RNAs that fully takes into account the effect of RNA secondary structure on binding affinity. Given a user-specified RNA and a protein selected from a set of several RNA-binding proteins, RBPBind computes their binding curve and effective binding constant. The server also computes the probability that, at a given protein concentration, a protein molecule will bind to any particular nucleotide along the RNA. The sequence specificity of the protein-RNA interaction is parameterized from public RNAcompete experiments and integrated into the recursions of the Vienna RNA package to simultaneously take into account protein binding and RNA secondary structure. We validate our approach by comparison to experimentally determined binding affinities of the HuR protein for several RNAs of different sequence contexts from the literature, showing that integration of raw sequence affinities into RNA secondary structure prediction significantly improves the agreement between computationally predicted and experimentally measured binding affinities. Our resource thus provides a quick and easy way to obtain reliable predicted binding affinities and locations for single-stranded RNA binding proteins based on RNA sequence alone.

© 2022 Elsevier Ltd. All rights reserved.

Introduction

RNA-binding proteins (or RBPs for short) are key players in every step of an RNA's life cycle and thus also in the process of post-transcriptional regulation. RBPs can control translation of a messenger RNA by being directly involved in recruiting the ribosome to the RNA, determining the stability of an RNA molecule, or directing the RNA to specific cellular compartments.[1,2] RBPs also play essential post-transcriptional roles in the splicing of different isoforms and the formation of poly(A) tails on the newly-transcribed pre-mRNA, and on a more global level, can form specialized complexes with non-coding RNAs.[3]

Recent technological advances have enabled comprehensive quantitative characterizations of the sequence dependence of protein-RNA interactions. Experiments such as RNAcompete,[4] RNA Bind-N-Seq,[5] and RNA-MaP[6] use high

0022-2836/© 2022 Elsevier Ltd. All rights reserved.

throughput approaches to determine the affinity of RNA binding proteins for every possible (or at least many in the case of RNA-MaP) RNA sequence in the footprint of the protein. However, when binding to an RNA molecule in the cell, protein binding competes with RNA secondary structures, which affects the affinity of a given RNA molecule for the protein beyond the dependence on the sequence of the binding site alone.

In the present work we introduce a web server, RBPBind, which combines the quantitative information on sequence dependence of the interaction of single stranded RBPs with RNA and the RNA secondary structure in order to compute the entire binding curve and hence also the effective binding constant of a single stranded RBP to an arbitrary RNA molecule. We delineate how the individual binding affinities from RNAcompete experiments can be integrated with secondary structure prediction by the well established Vienna RNA package[7] and validate our approach by comparison to biochemically determined RBP-RNA binding affinities. We believe RBPBind is unique in its ability to compute the probability of binding for a given RBP with an arbitrary RNA fully taking into account the experimentally determined sequence preferences of the RBP and RNA secondary structure.

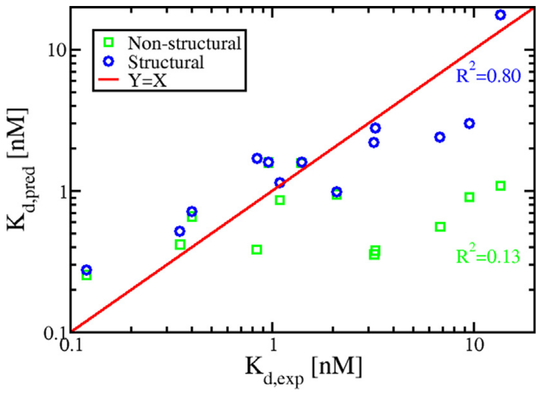

Figure 1. Comparison between biochemically determined and predicted effective binding constants for the binding of the RBP HuR to 13 different RNA molecules. The experimentally determined binding constant is shown on the x axis while the predicted binding constant is shown on the y axis. The green squares correspond to a prediction that only uses raw RNAcompete affinities for the different 7-mers in the RNA sequence. The blue circles correspond to RBPBind predictions that fully take RNA secondary structure into account. They are much closer to the ideal y = x line (red) than the green squares. The Pearson correlation coefficients of the logarithms of the dissociation constants are indicated for both data sets.

Results

Integration of RNAcompete data with RNA secondary structure prediction yields reliable RBP-RNA binding affinity prediction

High throughput experiments such as RNAcompete provide a table of the relative binding affinities for (nearly) every possible k-mer in the footprint of an RBP. Given the sequence of an RNA of N nucleotides, the binding affinities of the $N - k + 1$ potential binding sites on the given RNA can be looked up in this table and combined to determine the expected effective overall binding affinity of this RNA for the RBP (see Materials and Methods). The green squares in Figure 1 show a comparison of such determined binding affinities and experimentally determined binding affinities from the literature[8] for 13 different RNA molecules binding to HuR, a protein that ubiquitously binds the human transcriptome and is known for its regulatory importance.[9] While the experimentally determined affinities span over two orders of magnitude, the variation of the calculated affinities is only a factor of 5 and the overall Pearson correlation of $R^2 = 0.13$ between the two is low.

Since binding of a single-stranded RBP such as HuR precludes base pairing of the nucleotides of the RNA in the footprint of the RBP, there is a competition between protein binding and RNA secondary structure. Hackermüller *et al.*[10] pointed out that for an individual RBP binding site on an RNA this competition can be quantitatively incorporated into the recursion equations regularly used by RNA secondary structure prediction software. We later showed that this competition can still be quantitatively incorporated into the recursion equations if the RBP can bind *anywhere* along the RNA,[11] albeit in that publication without sequence specificity. We thus extended this framework and incorporated the competition between binding to an RBP at arbitrary positions along an RNA using the tabulated sequence dependent binding affinities from RNAcompete experiments and the RNA secondary structure formation of the RNA (see Materials and Methods). The blue circles in Figure 1 show the comparison between the resulting predicted effective binding affinities and the experimentally determined binding affinities for HuR and the same 13 different RNA molecules as above. While the correlation (Pearson's $R^2 = 0.80$) is not perfect, it is much stronger than when secondary structure is not taken into account and the predicted binding affinities span the same range as the experimentally determined ones.

The RBPBind web server allows calculation of effective binding affinities and binding locations for arbitrary RNA sequences

In order to make the RBP-RNA binding predictions widely accessible, we implemented the RBPBind web server at http://bioserv.mps.ohio-

state.edu/RBPBind/. The interface of RBPBind is shown in Figure 2. The user enters an RNA sequence of up to 250 bases, and chooses one of four available proteins selected based on availability of RNAcompete data and sufficient biochemical evidence to convert the relative binding constants derived from RNAcompete into absolute binding constants - HuR, RBFOX1, U2AF2 or KHDRBS3.

After the user clicks “Submit,” the server opens a window with the information shown in Figure 3(a). This output window shows the binding curve (the probability of an RBP being bound to the RNA as a function of free protein concentration), and the expected number of proteins bound to the RNA, also as a function of free protein concentration. The effective binding constant for the RBP-RNA interaction is calculated as the concentration at which half of the RNA molecules are bound by protein. The binding curve is shown as a function of *free* protein concentration, which approximately equals the input protein concentration only if the protein is in excess of the RNA, as appropriate for the determination of the effective binding constant. Thus, the server also allows entering an RNA concentration on the results page, in which case the probability of an RBP being bound to the RNA and the expected number of proteins bound to the RNA are shown in an additional graph as a function of input (total) protein concentration.

RBPBind also provides information about site-specific binding. Clicking a point on the graph, or entering a concentration in the box below it, will bring up a site-by-site profile, which gives the probability that protein will bind to any specific base. The graph in Figure 3(b) gives the probability that the site will be *covered* by protein, i.e., that this site is *anywhere* in the footprint of a binding protein; the graph in Figure 3(c) gives the probability that this site is the 5′ most site of the footprint of a binding protein.

Discussion

We have demonstrated through comparison with biochemical experiments that combining sequence dependent binding affinities from RNAcompete experiments with RNA secondary structure predictions allows reliable quantitative prediction of effective RBP-RNA binding affinities. Figure 1 shows that predictions that take structure into account and those that do not agree reasonably well for the strongest binding RNA molecules (on the left of the figure). This seems reasonable since strong binding likely indicates that the binding site is naturally exposed and since strong binding sites would be naturally able to compete better with RNA structure. However, for the more weakly binding RNAs (on the right of the figure) taking into account the RNA structure makes more of a difference, suggesting that the experimentally observed weaker binding of these RNAs is caused by the competition with the RNA secondary structure.

There are many web servers in the realm of RNA protein interactions. Several servers are dedicated to the determination of the residues on an RBP that contact the RNA[12–15] or to recognize RBPs in the first place.[16] There are also several servers that perform three dimensional docking of RBPs and RNAs to predict their joint structure.[17–19] However, none of these servers predicts binding affinities of the RBP-RNA interaction. To our knowledge, there are three web servers, Spot-Seq-RNA,[20]

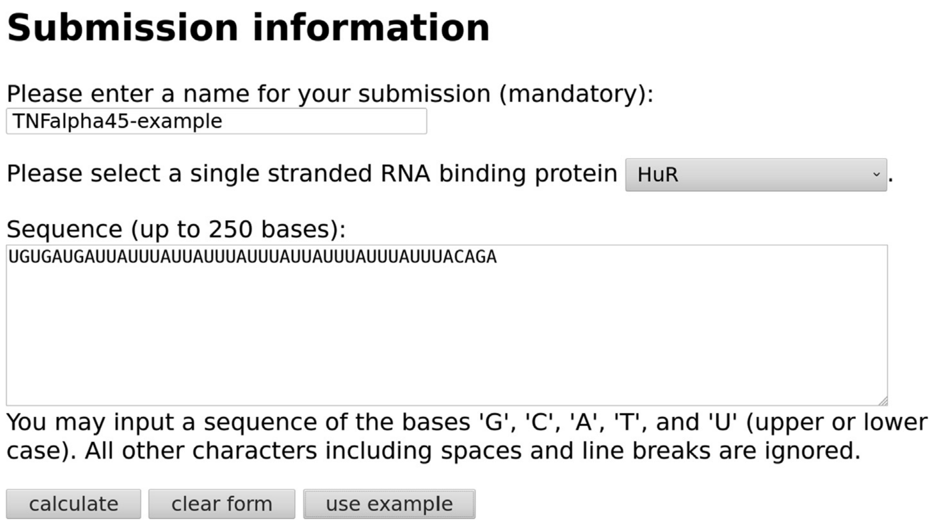

Figure 2. Submission page of the RBPBind web server. The server only requires the selection of an RBP and an RNA sequence (in addition to a job name) in order to start the prediction of the effective binding affinity of the RBP-RNA interaction.

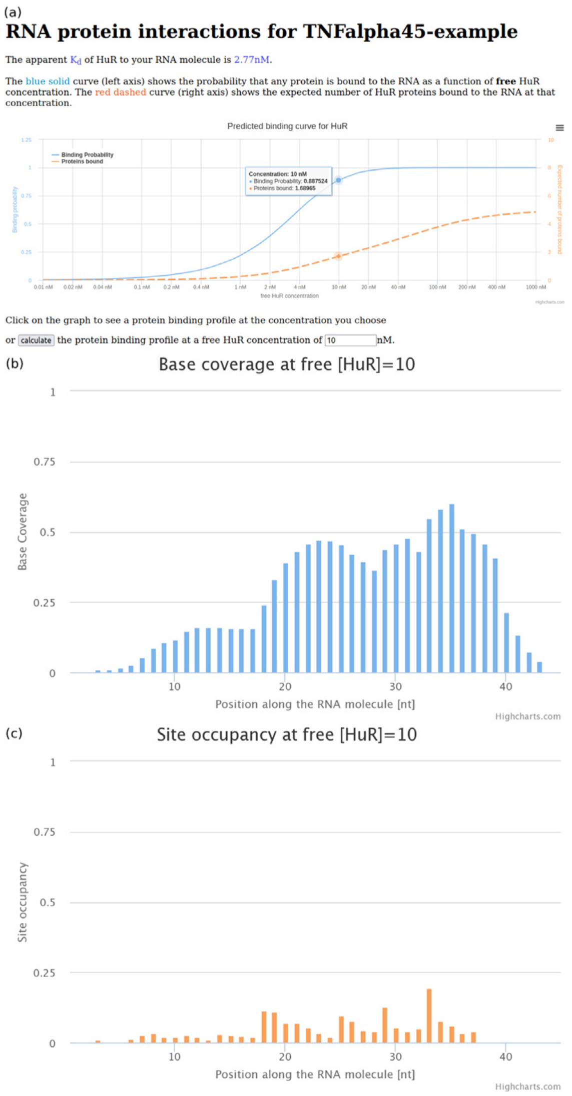

Figure 3. Output of the RBPBind web server. When provided with an RNA sequence and choice of an RBP, the web server initially responds with the effective K_d for the RNA-RBP interaction, the binding curve, and the expected number of proteins bound to the RNA as a function of free protein concentration (a). When clicking on a point in the graph or manually entering a protein concentration, the server further reports the probability of each base along the RNA to be covered by an RBP (b) and to be the first base in the footprint of an RBP (c) at the chosen free concentration of the RBP.

PredPRBA[21] and catRAPID,[22] that provide affinities for RBP-RNA interactions. The (currently defunct) Spot-Seq-RNA server largely focuses on the prediction of whether a given protein is an RNA binding protein or not and does not even consider the sequence of the RNA. PredPRBA takes the three-dimensional structure of the RNA protein complex as an input and thus cannot be applied to RNAs of arbitrary sequence. CatRAPID is conceptually the closest to our server in that it does consider RNA secondary structure albeit in a more heuristic way than our approach and the interaction propensity it reports is not an actual calibrated binding affinity that can be directly compared to biochemical experiments. Thus, RBPBind is unique in that it predicts actual binding affinities for RNAs of any sequence for a set of single-stranded RNA binding proteins taking full account of all sites on the RNA as well as the protein's competition with the RNA's secondary structure.

Currently, our server only supports four RNA binding proteins, HuR, RBFOX1, U2AF2, and KHDRBS3. Adding more proteins requires (i) the full set (for all 4^k possible sequences within a footprint of length k) of relative dissociation constants as well as (ii) absolute dissociation constants measured for a few RNA molecules to calibrate the offset unknown from the relative dissociation constants. Relative dissociation constants have been measured using RNAcompete[4] for hundreds of RNA binding proteins and while we have focused here on RNAcompete derived data sets, RNA Bind-N-Seq[5] provides relative dissociation constants as well. RNA-MaP[6] experiments tend to focus on a smaller set of possible binding sequences and do not yield a full set of all 4^k relative dissociation constants, although affinity models that are parameterized by RNA-MaP experiments could also be used even though they do not encode the full correlation structure of the 4^k independently measured relative dissociation constants. The main bottleneck for adding new proteins to the server is thus the availability of biochemical measurements of absolute binding constants for a few individual RNA molecules. We therefore explicitly encourage users to submit references for such individual measurements to us in the footer of all server pages. Since we provide the source code (as well as the processed relative dissociation constant files for the four proteins already implemented) at http://bioserv.mps.ohio-state.edu/RBPBind/download.php users are also able to experiment with how their own protein binding data interacts with RNA secondary structure.

Given the importance of protein-RNA interactions we expect our server to be of broad use for the RNA biology community. In the future, we anticipate adding support for additional proteins as well as for miRNA-RNA interactions as more biochemical data becomes available. We also plan to extend our server to allow prediction of the simultaneous interactions of multiple proteins with an RNA, as well as to integrate our recent work on double-stranded RBPs[23] into the server.

Materials and Methods

Determination of relative dissociation constants from RNAcompete data

RNAcompete's protein binding preference data is reported after several normalization and preprocessing steps described in the supplementary information of Ref.4. Since the final step of calcu-

lating z-scores perturbs the interpretation of the data as effective binding constants, the authors of Ref.[4] provided us with their intermediate data. We then extracted the relative dissociation constants $K_{d,n}^{(rel)}$ as one over the exponential of the raw values obtained in the data-processing procedure described in the supplementary information of Ref.[4] immediately before the final column z-score calculation, where n enumerates (nearly) all 4^k possible k-mers and k is the footprint size of the protein. For RNAcompete in Ref.,[4] $k = 7$. Since RNAcompete for technical reasons does not report affinities for certain (few) k-mers we imputed raw values for these missing k-mers as the mean raw value over the central 50% of the $3k$ k-mers varying from the missing k-mer by exactly one nucleotide.

Selection of proteins

As RNAcompete only provides relative but not absolute dissociation constants, we need biochemically measured absolute dissociation constants for calibration. We thus largely relied on the Protein-RNA Interface Database (PRIDB)[24] to identify publications, which report measured dissociation constants for proteins for which RNAcompete data is available. Due to the uncertainty in measuring the dissociation constants, we require that the biochemically measured dissociation constants for a given protein binding different RNA molecules cover a range of at least one order of magnitude.

We found four proteins, HuR, RBFOX1, U2AF2, and KHDRBS3, for which biochemically measured reference dissociation constants covering at least an order of magnitude in range were available. The range of biochemically measured dissociation constants was 40 for KHDRBS3 and notably larger for the three other proteins. The publications from which we derive our calibration dissociation constants are: Ref. 8 for HuR, Ref. 25 for RBFOX1, Ref. 26 for U2AF2; and Ref. 27 for KHDRBS3.

In order to make subsequent calculations more reliable, and also because the results of RBPBind are more useful for more selective proteins, we quantified the selectivity of each protein by the proportion of k-mers for which the affinity of the protein is <0.001 times that of the maximal-affinity k-mer. Proteins for which this “trivial k-mer” proportion is large (and thus the proportion of k-mers with affinities >0.001 times that of the maximal affinity k-mer is small) are more selective. We expect the performance of our model to be correlated with a protein’s selectivity. The “trivial k-mer” proportions of the four proteins HuR, RBFOX1, U2AF2, and KHDRBS3 were 78%, 58%, 29% and 1%, respectively.

Converting relative to absolute dissociation constants

To calibrate our relative dissociation constants $K_{d,n}^{(rel)}$ into absolute dissociation constants $K_{d,n}$, we use, as indicated above, measured dissociation constants $K_{d,eff}^{(exp)}$ obtained from the literature for interactions of the selected proteins and specific (long) RNA molecules. We list the sequences of the measured RNAs and their measured dissociation constants $K_{d,eff}^{(exp)}$ in Table S1.

The absolute k-mer dissociation constants are derived from the relative k-mer dissociation constants by multiplying with a global factor γ, i.e., as $K_{d,n} = \gamma K_{d,n}^{(rel)}$. We thus calculate for every RNA sequence s with a biochemically measured dissociation constant $K_{d,eff,s}^{(exp)}$ a preliminary effective dissociation constant $K_{d,eff,s}^{(prelim)}$ using the approach described below but with the uncalibrated, relative dissociation constants $K_{d,n}^{(rel)}$ as inputs to the algorithm and determine the global calibration factor γ by minimizing

$$\sum_s \left(\ln \frac{\gamma K_{d,eff,s}^{(prelim)}}{K_{d,eff,s}^{(exp)}} \right)^2 .$$

This corresponds to adjusting the global y axis offset in Figure 1. Figures S1–S3 show the calibration data for RBFOX1, U2AF2, and KHDRBS3, respectively.

Effective dissociation constant in the absence of secondary structure

To compute the probability that a single protein binding site is occupied, we note that by definition we have $K_d = \frac{[R]c}{[RP]}$, where [R] and [RP] are the concentrations of unbound and bound RNA respectively and c is the concentration of free protein. This can be converted to $[RP]/[R] = c/K_d$. Therefore, the probability that a single protein binding site is occupied is given by $P(site\ occupied) = \frac{[RP]}{[R]+[RP]} = \frac{[RP]/[R]}{1+[RP]/[R]} = \frac{c/K_d}{1+c/K_d}$. Thus, the free protein concentration at which half of the binding sites are occupied is given by $1 + c/K_d = 2$. In this expression the 1 corresponds to the unoccupied state and the c/K_d represents the occupied state. For an unstructured RNA of length N and a protein with footprint size k, there are $N - k + 1$ possible binding sites, each with their own dissociation constant $K_{d,i}$ for $i = 1,\ldots,N - k + 1$. These binding constants are given by the k-mer in the RNA sequence starting at the ith nucleotide derived from RNAcompete experiments as described above. To calculate the total contribution of all these binding sites while taking their overlaps into account, we define $Q_i(c)$ to be the contribution of all binding sites on the first i nucleotides of the RNA. Since no protein can bind an RNA shorter than the protein footprint, $Q_i(c) = 1$ for $i = 1,\ldots,k - 1$. For longer pieces of RNA, protein binding configurations can be split into the configurations where nucleotide i is not occupied by a protein and configurations where a protein binds at the site starting at nucleotide $i - k + 1$ and ending at nucleotide i. This leads to the recursion

$$Q_i(c) = Q_{i-1}(c) + Q_{i-k}(c) \cdot c/K_{d,i-k+1}$$

for all $i = k,\ldots,N$. This allows numerical evaluation of $Q_N(c)$ for any protein concentration c in $O(N)$ time and thus the numerical solution of the equation $Q_N(K_{d,eff}) = 2$ yielding the effective dissociation constant $K_{d,eff}$ for the RNA of the given sequence by bisection using the monotonicity of $Q_N(c)$.

Structural free energy in the presence of an RBP

To calculate the structural free energy in the presence of an RBP, we followed our previous approach[11] and replaced each occurrence of a single-stranded region in the recursion equations for the full partition function over the secondary structure ensemble with a partition function $Q_{i,j}(c)$ taking into account all protein binding configurations on that particular piece of the RNA from the ith to the jth nucleotide of the RNA. The latter use the dissociation constants $K_{d,l}$ for each k-mer of the RNA sequence and can be precomputed for a given protein concentration in $O(N^2)$ time analogously to the computation of $Q_i(c)$ above. Thus, the computation of the entire protein concentration partition function $Z(c)$ remains $O(N^3)$. The free energy of the structural and protein binding ensemble is then $\Delta G = -k_B T \ln Z(c)$.

Binding curve

Given the partition function $Z(c)$ for the RNA secondary structure ensemble in the presence of an RBP at concentration c, the probability of an RNA molecule to be bound by at least one protein as a function of RBP concentration c is given by

$$p_{bound}(c) = \frac{Z(c) - Z(0)}{Z(c)}$$

Effective dissociation constant in the presence of secondary structure

The effective dissociation constant $K_{d,eff}$ is the concentration at which half of the RNA molecules are occupied by at least one protein. Since the binding curve $p_{bound}(c)$ can be calculated numerically and is a monotonic function of free protein concentration c, the effective dissociation constant can be found by numerically solving the equation $p_{bound}(K_{d,eff}) = \frac{1}{2}$ via bisection.

Number of bound proteins and binding curve as a function of input protein concentration

The average number of bound proteins (per RNA) is given by $\langle N(c)\rangle = c \frac{\partial}{\partial c} \ln Z(c)$. In practice, we calculate it as $\langle N(c)\rangle \approx \ln[Z(1.01c)/Z(c)]/0.01$. Given this average number of bound proteins, the free protein concentration c, the input protein concentration c_{tot}, and the RNA concentration c_{RNA} are related as $c_{tot} = c + \langle N(c)\rangle c_{RNA}$. This allows calculation of the binding probability p_{bound} and the average number of bound proteins $\langle N\rangle$ as a function of free protein concentration c and then plotting them as a function of input protein concentration c_{tot}.

Protein occupancies

The probability P_i that a protein is bound with the footprint starting at the ith nucleotide is given as the derivative of the logarithm of the partition function $Z(c)$ with respect to the dissociation constant $K_{d,i}$ for the protein binding event at that position. In practice, we calculate for every position i the perturbed partition function $Z_i(c)$, in which $K_{d,i}$ is replaced by $K_{d,i}/1.01$, to obtain a numerical approximation of the derivative as $P_i \approx \ln[Z_i(c)/Z(c)]/0.01$. The probability that nucleotide i is occupied by any protein is then $\sum_{j=i-k+1}^{i} P_j$ as protein binding events at sites within one footprint length are mutually exclusive.

CRediT authorship contribution statement. **Jeff Gaither:** Methodology, Software, Validation, Investigation, Writing – original draft, Writing – review & editing. **Yi-Hsuan Lin:** Methodology, Software, Validation, Investigation, Writing – review & editing. **Ralf Bundschuh:** Conceptualization, Methodology, Software, Resources, Writing – original draft, Writing – review & editing, Supervision, Project administration, Funding acquisition.

DATA AVAILABILITY

Data will be made available on request.

DECLARATION OF COMPETING INTEREST

The authors declare that they have no known competing financial interests or personal relationships that could have appeared to influence the work reported in this paper.

Acknowledgements

We are grateful for useful discussions with Quaid Morris from the University of Toronto and for his sharing raw RNAcompete data and analysis code with us. We also acknowledge server coding help by Mark Bundschuh. This material is based upon work supported by the National Science Foundation under Grants No. DMS-0931642 (JG) DMR-1410172 (RB), and DMR-1719316 (RB).

Appendix A. Supplementary Data

Supplementary data to this article can be found online at https://doi.org/10.1016/j.jmb.2022.167515.

Received 29 November 2021;
Accepted 22 February 2022;
Available online 26 February 2022

Keywords:
protein-RNA interactions;
RNA secondary structure;
binding affinity;
web server

† These authors equally contributed to this work.
‡ present address: HTuO Biosciences Inc., Vancouver V5N 1R1, Canada.

References

1. Hentze, M.W., Castello, A., Schwarzl, T., Preiss, T., (2018). A brave new world of RNA-binding proteins. *Nat. Rev. Mol. Cell Biol.* **19**, 327–341.
2. Singh, G., Pratt, G., Yeo, G.W., Moore, M.J., (2015). The Clothes Make the mRNA: Past and Present Trends in mRNP Fashion. *Annu. Rev. Biochem.* **84**, 325–354.
3. Glisovic, T., Bachorik, J.L., Yong, J., Dreyfuss, G., (2008). RNA-binding proteins and post-transcriptional gene regulation. *FEBS Lett.* **582**, 1977–1986.
4. Ray, D., Kazan, H., Chan, E.T., Peña Castillo, L., Chaudhry, S., Talukder, S., Blencowe, B.J., Morris, Q., et al., (2009). Rapid and systematic analysis of the RNA recognition specificities of RNA-binding proteins. *Nat. Biotechnol.* **27**, 667–670.
5. Lambert, N., Robertson, A., Jangi, M., McGeary, S., Sharp, P.A., Burge, C.B., Bind-n-Seq, R.N.A., (2014). quantitative assessment of the sequence and structural binding specificity of RNA binding proteins. *Mol. Cell* **54**, 887–900.
6. Buenrostro, J.D., Araya, C.L., Chircus, L.M., Layton, C.J., Chang, H.Y., Snyder, M.P., Greenleaf, W.J., (2014). Quantitative analysis of RNA-protein interactions on a massively parallel array reveals biophysical and evolutionary landscapes. *Nat. Biotechnol.* **32**, 562–568.
7. Lorenz, R., Bernhart, S.H., Höner Zu Siederdissen, C., Tafer, H., Flamm, C., Stadler, P.F., Hofacker, I.L., (2011). ViennaRNA Package 2.0. *Algorithms Mol. Biol.* **6**, 26.
8. Meisner, N.-C., Hackermüller, J., Uhl, V., Aszódi, A., Jaritz, M., Auer, M., (2004). mRNA Openers and Closers: Modulating AU-Rich Element-Controlled mRNA Stability by a Molecular Switch in mRNA Secondary Structure. *ChemBioChem.* **5**, 1432–1447. https://doi.org/10.1002/cbic.200400219.
9. Brennan, C.M., Steitz, J.A., (2001). HuR and mRNA stability. *Cell. Mol. Life Sci.* **58**, 266–277.

10. Hackermüller, J., Meisner, N.-C., Auer, M., Jaritz, M., Stadler, P.F., (2005). The effect of RNA secondary structures on RNA-ligand binding and the modifier RNA mechanism: a quantitative model. *Gene* **345**, 3–12.
11. Forties, R.A., Bundschuh, R., (2010). Modeling the interplay of single-stranded binding proteins and nucleic acid secondary structure. *Bioinformatics* **26**, 61–67.
12. Terribilini, M., Sander, J.D., Lee, J.-H., Zaback, P., Jernigan, R.L., Honavar, V., Dobbs, D., (2007). RNABindR: a server for analyzing and predicting RNA-binding sites in proteins. *Nucleic Acids Res.* **35**, W578–W584.
13. El-Manzalawy, Y., Abbas, M., Malluhi, Q., Honavar, V., (2016). FastRNABindR: Fast and Accurate Prediction of Protein-RNA Interface Residues. *PLoS One.* **11**, e0158445
14. Yan, J., Kurgan, L., (2017). DRNApred, fast sequence-based method that accurately predicts and discriminates DNA- and RNA-binding residues. *Nucleic Acids Res.* **45**, e84
15. Luo, J., Liu, L., Venkateswaran, S., Song, Q., Zhou, X., (2017). RPI-Bind: a structure-based method for accurate identification of RNA-protein binding sites. *Sci. Rep.* **7**, 614.
16. Ghosh, P., Mathew, O.K., Sowdhamini, R., (2016). RStrucFam: a web server to associate structure and cognate RNA for RNA-binding proteins from sequence information. *BMC Bioinf.* **17**, 411.
17. Tuszynska, I., Magnus, M., Jonak, K., Dawson, W., Bujnicki, J.M., (2015). NPDock: a web server for protein-nucleic acid docking. *Nucleic Acids Res.* **43**, W425–W430.
18. Huang, Y., Li, H., Xiao, Y., (2018). 3dRPC: a web server for 3D RNA-protein structure prediction. *Bioinformatics* **34**, 1238–1240.
19. Zheng, J., Hong, X., Xie, J., Tong, X., Liu, S., (2020). P3DOCK: a protein-RNA docking webserver based on template-based and template-free docking. *Bioinformatics* **36**, 96–103.
20. Yang, Y., Zhao, H., Wang, J., Zhou, Y., (2014). SPOT-Seq-RNA: predicting protein-RNA complex structure and RNA-binding function by fold recognition and binding affinity prediction. *Methods Mol. Biol.* **1137**, 119–130.
21. Deng, L., Yang, W., Liu, H., (2019). PredPRBA: Prediction of Protein-RNA Binding Affinity Using Gradient Boosted Regression Trees. *Front. Genet.* **10**, 637.
22. Armaos, A., Colantoni, A., Proietti, G., Rupert, J., Tartaglia, G.G., (2021). catRAPID omics v2.0: going deeper and wider in the prediction of protein-RNA interactions. *Nucleic Acids Res.* **49**, W72–W79.
23. Shatoff, E., Bundschuh, R., (2022). dsRBPBind: Modeling the effect of RNA secondary structure on double stranded RNA-protein binding. *Bioinformatics* **38**, 687–693.
24. Lewis, B.A., Walia, R.R., Terribilini, M., Ferguson, J., Zheng, C., Honavar, V., Dobbs, D., (2011). PRIDB: a Protein-RNA interface database. *Nucleic Acids Res.* **39**, D277–D282.
25. Auweter, S.D., Fasan, R., Reymond, L., Underwood, J.G., Black, D.L., Pitsch, S., Allain, F.-H.-T., (2006). Molecular basis of RNA recognition by the human alternative splicing factor Fox-1. *EMBO J.* **25**, 163–173.
26. Mackereth, C.D., Madl, T., Bonnal, S., Simon, B., Zanier, K., Gasch, A., Rybin, V., Valcárcel, J., et al., (2011). Multi-domain conformational selection underlies pre-mRNA splicing regulation by U2AF. *Nature* **475**, 408–411.
27. Galarneau, A., Richard, S., (2009). The STAR RNA binding proteins GLD-1, QKI, SAM68 and SLM-2 bind bipartite RNA motifs. *BMC Mol. Biol.* **10**, 47.
