# Supplementary material for: PDBx/mmCIF Ecosystem: Foundational Semantic Tools for Structural Biology
Source: J Mol Biol. Author manuscript; Available in PMC 2023 Jun 26. (PMC10292674; doi:10.1016/j.jmb.2022.167599)
Supplement: Article [file NIHMS1907597-supplement-Article.zip › 3dRNA--3D-Structure-Prediction-from-Linear-to-Ci_2022_Journal-of-Molecular-B.pdf]

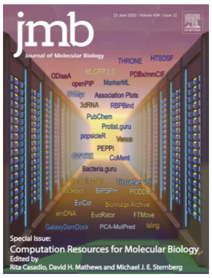

3dRNA: 3D Structure Prediction from Linear to Circular RNAs

Yi Zhang, Jun Wang and Yi Xiao *

***Institute of Biophysics,** School of Physics, Huazhong University of Science and Technology, Wuhan 430074, Hubei, China*

Correspondence to Yi Xiao:
https://doi.org/10.1016/j.jmb.2022.167452
Edited by David Mathews

Abstract

3D structures of RNAs are the basis for understanding their biological functions. However, experimentally solved RNA 3D structures are very limited. Therefore, many computational methods have been proposed to solve this problem, including our 3dRNA. 3dRNA is an automated template-based method of building RNA 3D structures from sequences and secondary structures by using the smallest secondary elements (SSEs) (http://biophy.hust.edu.cn/new/3dRNA). The first version of 3dRNA simply predicts an assembled structure for a target RNA. Later, it is improved to generate a set of assembled models and a method to further optimize them using experimental or theoretical restraints. In particular, pseudoknot base pairings are treated as restraints to solve the problem of no 3D templates for pseudoknots. Here 3dRNA is further extended to predict the 3D structures of circular RNAs since thousands of circular RNAs have been found recently but no 3D structures of them have been determined up to now. We show that circular RNAs can be divided into four types and two types show similar 3D structures with their linear counterparts while two types very different. We also show that the predicted structures of circular RNAs can bind to their ligands more stable than those of their linear counterparts, consistent with experimental results.
© **2022 Elsevier Ltd. All rights reserved.**

Introduction

Three dimensional (3D) structures of RNAs are the basis of their biological functions. However, experimentally solved RNA 3D structures are very limited in comparison with known RNA sequences up to now. Therefore, many computational methods have been proposed to solve this problem,[1–12] including our 3dRNA.[13–17] Current prediction programs can be roughly divided into two categories. The first category is ab initial prediction based on molecular dynamics simulation, like NAST[4] and iFoldRNA.[6] The second category is template-based approach, like FARNA/FRAFRA,[1,2] Vfold[8,9] and RNAComposer[10,11] and 3dRNA proposed in our laboratory.

The methods above all are for linear RNAs. Recently, circular RNAs (circRNAs), the 3′ and 5′ ends of which are covalently linked, constitute a class of non-coding RNA discovered to be widespread and abundant.[18] Unlike the standard shearing pattern of conventional linear RNA, circRNAs are generally formed by backsplicing of pre-mRNA.[19,20] Although the current knowledge of circRNA is very limited, a large number of circular RNAs (circRNAs) in fungi, protists, plants, fruit flies, mice and human cells have been discovered and proposed to carry out diverse functions in cells.[21] They can act as miRNA sponge to compete for miRNA with other endogenous RNAs to affect the expression of miRNA target genes, they can interact with RNA binding proteins to regulate their functions, they can act as “Scaffolding” for RNA-binding proteins by binding to a variety of proteins to promote protein assemble. These functions of circRNAs suggest that they should have 3D

structures. However, no experimental 3D structures of circRNAs have been measured currently. This greatly hinders the understanding of the mechanism of their functions. Computational algorithms of predicting secondary (2D) structures of circRNAs have already been implemented in Mfold Package[22] and Vienna RNA Package.[23] Here we extend our 3dRNA to include circRNAs and hope this can help to understand their functions.

In this paper we first give a brief introduction of 3dRNA for linear RNAs since it has been described previously and here we mainly show the new aspects of 3dRNA for building the 3D structures of circular RNAs.

3dRNA for linear RNAs

3dRNA is an automated templated-based method of building RNA 3D structures from sequences and secondary structures. Figure 1 shows the workflow of 3dRNA in structure prediction and the details can be found in our previous papers.[16]

(1) Inputs: The inputs of 3dRNA are the sequence and secondary structure of the target RNA. The secondary structure can be predicted from 2dRNA[36] or other popular methods. The length of the target RNA is not limited in 3dRNA, though the accuracy will become lower for longer ones.
(2) SSE decomposition: The input secondary structure is decomposed into a tree, each node of which corresponds to an SSE and each edge connects two successive SSEs. The SSEs are defined as stems and loops. The latter includes hairpin loops, internal loops, bulge loops, and junction loops. Two base-pairs are attached to the ends of every loops, thus we can assemble two successive templates more accurately by overlapping them together (Figure 2).
(3) SSE 3D templates searching: For each of the SSEs suitable 3D templates are searched from the 3D template library of SSEs built from experimental 3D structures of RNAs in PDB databank.[17] The 3D template library of SSEs contains two sub-libraries: helix library and loop library. The helix library includes all kinds of helices extracted from experimentally determined RNA structures of different lengths and different sequences. The loop library consists of different types of loops including hairpin loops, bulge loops, internal loops and multi-branch loops. Hairpin loops, bulge loops, internal loops involve all available types and sequences. The multi-branch loops (or named junction) contain 3-way junction, 4-way junction, 5-way junction, and so on. Now, there are 27,163 helices and 27,826 loops in the libraries, respectively.[17]
(4) 3D SSE template generating: In the case of absence of 3D templates for a SSE, 3dRNA will use a bi-residues method or Distance-Geometry method[24,25] to generate its 3D templates. So, 3dRNA can also generate 3D templates for all SSEs of an RNA by these two methods and use them to assemble the RNA structure instead of the 3D templates extracted from experimental structures.

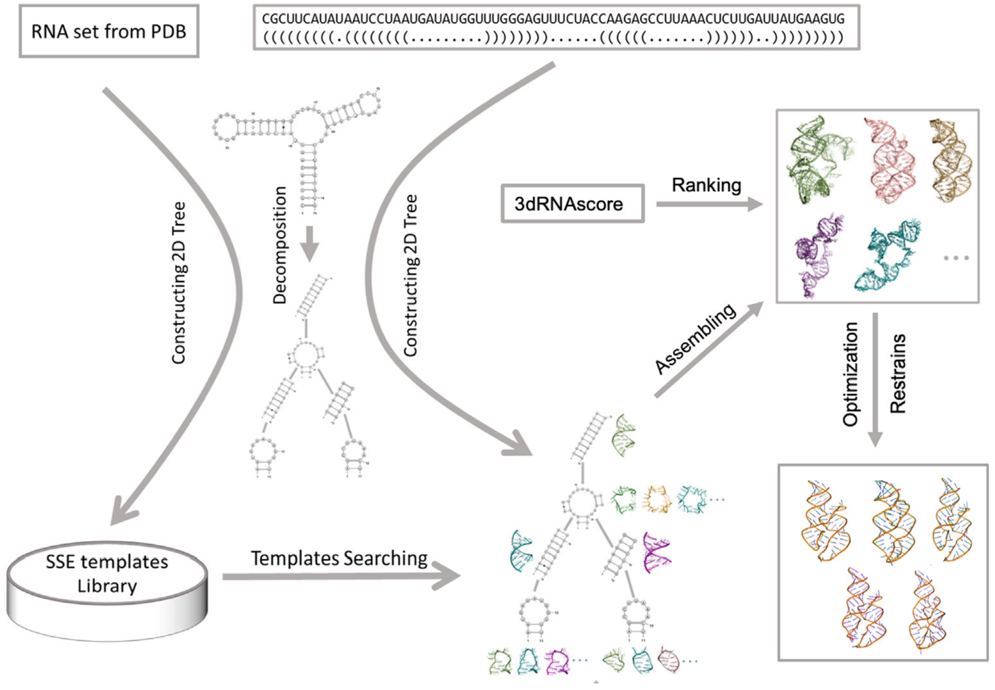

Figure 1. Workflow of 3dRNA in prediction of RNA 3D structure.

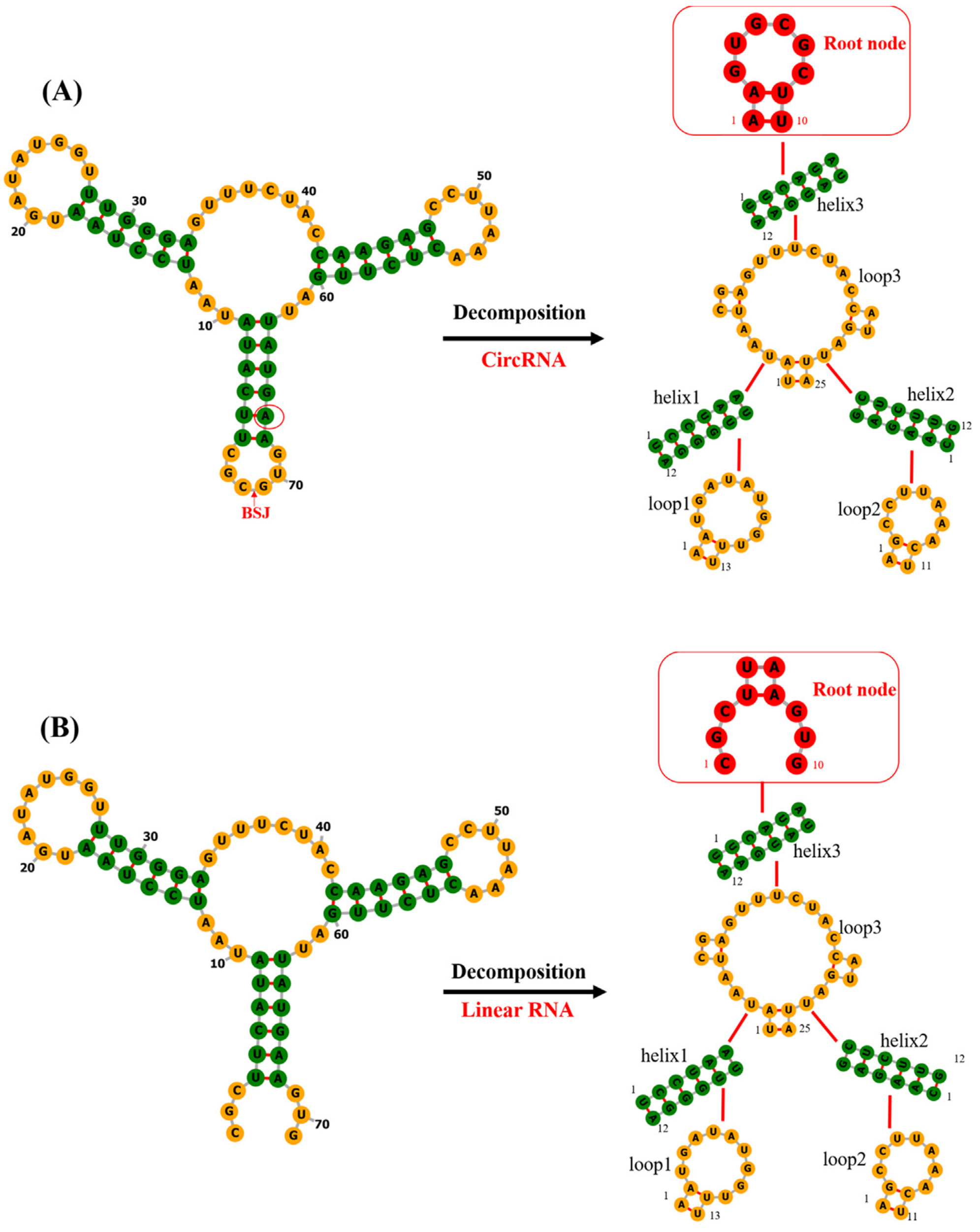

Figure 2. An example showing the SSE decomposition of a circRNA and its linear counterpart in 3dRNA. (A) and (B) are the 2D structures and all the SSEs that together form secondary structure trees for the circRNA and its linear counterpart, respectively. The root node is a hairpin in (A) but a simple open loop in (B). It is noted that for the circRNA the sequence of the SSE containing BSJ is re-numbered to have the 5′-end residue as the first residue like in a standard SSE, e.g., the red-circled nucleotides in the left of (A) is taken as the first residue of the root node (see Method for the details).

(5) 3D SSE templates assembling: Starting from the root node, the selected 3D template of the SSE at each node is assembled with that of its parent node. The Kabsch's method[26] is used as the superposition method. After traversing through the tree, an assembled structure of the target RNA is obtained. Since each node (SSE) may have more than one 3D templates, the template having the highest sequence homology with the query SSE will be selected firstly. If users want to get more than one predictions, 3dRNA repeats this step and other templates will be selected randomly. Then the assembled 3D structures are clustered by the k-means algorithm according to their RMSD values from each other and the centroids of each clusters are evaluated by 3dRNAscore.[13] 3dRNAscore is an all-atom statistical potential scoring function of atom–atom distances and backbone dihedral angles. The dihedral-based energy can describe the flexibility

of RNA molecules more efficiently. Further-more, 3dRNAscore also considers the RNA stacking interactions in adjacent bases in the calculation of the distance-based energy.

(6) Optimization of assembled structures. Users can further optimize these assembled structures by using an optimization procedure with or without addition of restraints from experiments and/or theoretical predictions.[27] The optimization method used in 3dRNA is the Simulated Annealing Monte Carlo (SAMC) optimization [27]. The optimizied structures are clustered by using the k-means clustering algorithm according to their RMSD values from each other and the centroid of each cluster is ranked by a coarse-grained energy function consisting of base pairing and base stacking energy terms. The ranked top N predictions are given to the user finally. The optimization is usually done for two cases: when 3D templates of some SSEs cannot be found and when the restraints (distance or pairing, including pseudoknot pairing) between residues are available. In these cases the prediction accuracy of 3dRNA could be significantly improved by optimization. In particular, the base pairings in pseudoknots are treated as restraints in 3dRNA and this avoids the difficulty of finding 3D templates for pseudoknots.[16]

3dRNA for Circular RNAs

CircRNA is a unique class of RNAs with covalently closed structure that lack 5′ caps and 3′ poly(A) tails. The head-to-end joint of circRNA sequence, namely the cyclization site, is referred as backsplice junction (BSJ) region[28,29] (Figure 2(A)). It is at the BSJ region that the SSE decomposition and 3D-template assembling of circRNAs are different from linear RNAs (Figure 2(B)).

The SSE containing BSJ can be divided into helix, hairpin, internal loop (including bulge) and junction loop, as shown in Figure S1. For an example, in Figure S1(A) the root node is an open loop in the linear RNA but a helix in circRNAs. Accordingly, circRNAs can be divided into four types depending on the features of their SSEs containing BSL: Helix-circRNA, Hairpin-circRNA, Internal-circRNA and Junction-circRNA. We shall analyze and build 3D structures for each type. For comparison, we shall also build 3D structures of their linear counterparts, i.e., the circRNA with broken BSJ. For convenience, the linear counterpart of a circRNA is called as lineRNA for short.

We predicted the 3D structures of circRNAs in the dataset from Chen's group.[30] This dataset has 34 circRNAs with lengths from 161nt to 435nt, including 4 Helix-circRNAs, 6 Hairpin-circRNAs, 14 Internal-circRNAs and 10 Junction-circRNAs. Their secondary structures were calculated by RNAfold integrated with SHAPE reactivity profiles.[30,31] The detailed information of the circRNAs in the dataset is available at http://biophy.hust.edu.cn/new/3dRNA.

As shown in Figures S2–S5, an example of a predicted structure is shown for each of the four types circRNAs, as well as a comparison with their corresponding linear counterpart. Also, the RMSD distribution of the circRNA and lineRNA for the assembled structure of the dataset are shown in Figure S6. To eliminate the influence of SAMC optimization, we use the assembled structures for comparison.

For Helix-circRNA and Hairpin-circRNA, the assembled 3D structures of circRNAs and their lineRNAs are almost the same. Figure S2 shows an example of Helix-circRNA, circASXL1, which consists of 195 residues. The RMSD (Root Mean Standard Deviation) between the predicted structures of the circRNA and lineRNA is 2.24 Å. The slight difference is due to the structural deviation of the second sub-node fragment connected with the root node. Although the root-node SSE is different, an open loop "(())(())" for lineRNA and a helix "(((())))" for circRNA, similar structures of them cause the similarity of overall 3D structures. Figure S3 shows an example of Hairpin-circRNA, circSNHG4, which consists of 161 residues. The RMSD between the 3D structures of circSNHG4 and its lineRNA is 2.20 Å. The major difference of the circRNA and lineRNA is in the SSE containing BSJ, which are hairpins in the circRNAs but open loops in their lineRNAs.

For Internal-circRNA, the assembled structures are very different from those of their lineRNAs due to the large difference in the SSE containing BSJ. Figure S4 shows an example of Internal-circRNA, circFGFR1_1, which consists of 179 residues. The RMSD between the predicted structures of circFGFR1_1 and its lineRNA is 15.16 Å. The SSE containing BSJ of circFGFR1_1 is an internal loop "((..(())..))" and it is different from the open loop "..(())..(())" of the lineRNA.

Junction-circRNAs are the most special and their assembled structures are globally different from their lineRNAs due to the great difference in the SSE containing BSJ. The SSEs containing BSJ in Junction-circRNAs are multi-way junctions, but open-loops in the lineRNAs, which leads to completely different 2D and 3D structures of the two SSEs and this further affects the orientation of the branches of the junctions in space, which results in different global structures of the Junction-circRNAs and their lineRNAs. Figure S5 gives an example of Junction-circRNA, circEPHB4, which consists of 362 residues. The RMSD between the assembled structures of circEPHB4 and its lineRNA is 37.9 Å. The 4-way junction "((((()).. .. . .(())(()).........))" marked by red circle in Figure S5(A) of circEPHB4 greatly differs from the open loop ".......(())(()).............(())

(()).." of its lineRNA, which directly leads to the complete deviation of the domain containing the three-way junction and four-way junction(yellow circle) to others domain of circRNA and lineRNA and this greatly affects their overall structures.

Since no experimental 3D structures of circRNAs are available currently, the performance of 3dRNA cannot be validated directly. So, here an indirect validation is performed. Recent studies revealed that many circRNAs tend to form intra-molecularly imperfect RNA duplexes (dsRNA) to distinguish themselves from their linear cognate RNAs, which allow them prefer to bind to dsRNA-binding proteins, such as the innate immune dsRNA receptor PKR.[30,32,33] Therefore, we see if the predicted structures of the circRNAs can bind to PKR more strongly than those of their linear RNAs.

PKR is an interferon-induced enzyme that plays a key role in the control of viral infections and cellular homeostasis,[34] which is a protein with 551 amino acids and consisting of two functional domains: a N-terminal dsRNA binding domain (dsRBD) that comprises two dsRNA binding motifs (dsRBMs) and a C-terminal kinase domain that contains the major sites for phosphorylation. Available structure of dsRBD is PDB ID 1QU6 with a length of 169nt, and a crystal structure of the kinase domain complexed with eIF2a substrate can also be found in PDB with PDB ID 2A1A.

In order to explore the binding ability of circRNAs and PKR, dsRBD-circRNA and dsRBD-lineRNA complexes are built by HDock[35] for ten circRNAs and their linear RNA cognates[30] (see Table S1). 2D structures of these circRNAs and their linear RNAs are obtained by circSHAPE-MaP and linearSHAPE-MaP. For most circRNAs, their dsRNA regions can bind to both dsRBDs of PKR, but for their linear RNAs, they can at most bind to one of the dsRBDs. This suggests that the binding between circRNAs and PKR is more stable than those between their linear RNAs and PKR. Figure 3 shows a typical example, where the circRNA is circPOLR2A with a length of 336nt. Comparing with circPOLR2A, the predicted 3D structure of the linear POLR2A is looser and has more unpaired loop regions. For circPOLR2A, dsRBD1 binds perfectly to a 19 bp dsRNA in Domain2, and dsRBD2 binds to two hairpins in Domain1 and Domain3, while for linear POLR2A only the dsRBD1 of PKR bind to a 17 bp dsRNA in the Domain1.

In summary, we have developed a template-based method to build the 3D structures of linear RNAs and here it is extended to include circRNAs. Although the prediction accuracy for circular RNAs cannot be directly evaluated currently, we expect that it is similar to that for linear RNAs since the principles of building the 3D structures of linear and circular RNAs are the same. The indirect

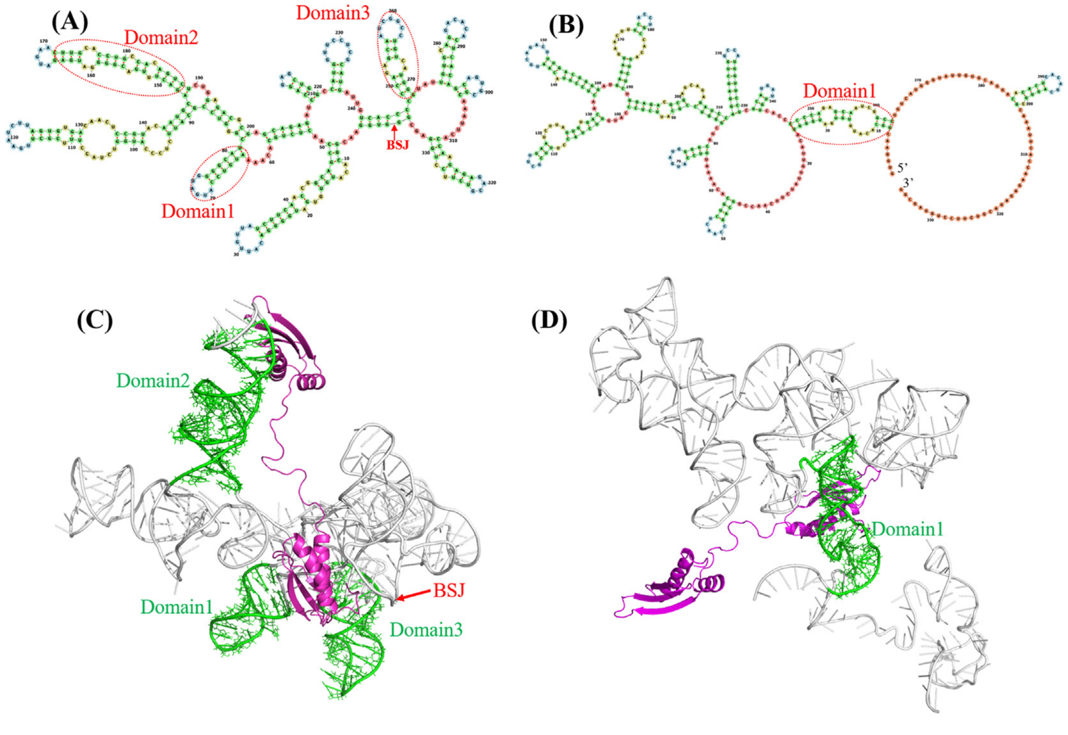

Figure 3. Binding of circPOLR2A with dsRBD. (A) and (B) The 2D structure of circPOLR2A and its linear RNA. The binding domains are marked with red circles. (C) and (D) The docked 3D structures of circular POLR2A (grey) and their linear RNA interacted with dsRBD (magenta), respectively. The binding regions Domain1(hairpin), Domain2 (dsRNA) and Domain3 (hairpin) are marked with green.

validation of 3dRNA for circular RNAs also shows that the predicted 3D structures of circular RNAs are reasonable. This extended version of 3dRNA may be helpful to understand more about the details of the structure and interaction of circRNAs.

Availability

The web server of building 3D structures of circRNAs and the related data are accessible at http://biophy.hust.edu.cn/new/3dRNA.

CRediT authorship contribution statement

Yi Zhang: Software, Writing – review & editing. **Jun Wang:** Investigation, Methodology, Formal analysis, Visualization, Software, Writing – original draft, Writing – review & editing. **Yi Xiao:** Conceptualization, Supervision, Funding acquisition, Writing – review & editing.

DATA AVAILABILITY

I have shared the link of my used data.

Acknowledgement

This work is supported by the NSFC under Grant No. 32071247.

Declaration of Competing Interest

The authors declare that they have no known competing financial interests or personal relationships that could have appeared to influence the work reported in this paper.

Appendix A. Supplementary data

Supplementary data to this article can be found online at https://doi.org/10.1016/j.jmb.2022.167452.

Received 11 November 2021;
Accepted 7 January 2022;
Available online 13 January 2022

Keywords:
RNA 3D structure prediction;
linear RNA;
circular RNA

Abbreviations:
3D, Three-Dimensional; SSE, Smallest Secondary Element; RMSD, Root Mean Square Deviation; circRNA, circular RNA; lineRNA, linear RNA; BSJ, Backsplice Junction

References

1. Das, R., Baker, D., (2007). Automated de novo prediction of native-like RNA tertiary structures. *Proc. Natl. Acad. Sci. USA* **104**, 14664–14669.
2. Das, R., Karanicolas, J., Baker, D., (2010). Atomic accuracy in predicting and designing noncanonical RNA structure. *Nature Methods* **7**, 291–294.
3. Parisien, M., Major, F., (2008). The MC-Fold and MC-Sym pipeline infers RNA structure from sequence data. *Nature* **452**, 51–55.
4. Jonikas, M.A., Radmer, R.J., Laederach, A., Das, R., Pearlman, S., Herschlag, D., et al., (2009). Coarse-grained modeling of large RNA molecules with knowledge-based potentials and structural filters. *RNA* **15**, 189–199.
5. Gherghe, C.M., Leonard, C.W., Ding, F., Dokholyan, N.V., Weeks, K.M., (2009). Native-like RNA tertiary structures using a sequence-encoded cleavage agent and refinement by discrete molecular dynamics. *J. Am. Chem. Soc.* **131**, 2541–2546.
6. Sharma, S., Ding, F., Dokholyan, N.V., (2008). iFoldRNA: three-dimensional RNA structure prediction and folding. *Bioinformatics* **24**, 1951–1952.
7. Jossinet, F., Ludwig, T.E., Westhof, E., (2010). Assemble: an interactive graphical tool to analyze and build RNA architectures at the 2D and 3D levels. *Bioinformatics* **26**, 2057–2059.
8. Cao, S., Chen, S.J., (2011). Physics-based de novo prediction of RNA 3D structures. *J. Phys. Chem. B* **115**, 4216–4226.
9. Xu, X., Zhao, P., Chen, S.J., (2014). Vfold: a web server for RNA structure and folding thermodynamics prediction. *PLoS One* **9**, e107504.
10. Popenda, M., Szachniuk, M., Antczak, M., Purzycka, K.J., Lukasiak, P., Bartol, N., et al., (2012). Automated 3D structure composition for large RNAs. *Nucleic Acids Res.* **40**, e112.
11. Biesiada, M., Purzycka, K.J., Szachniuk, M., Blazewicz, J., Adamiak, R.W., (2016). Automated RNA 3D Structure Prediction with RNAComposer. *Methods Mol. Biol.* **1490**, 199–215.
12. Jain, S., Schlick, T., (2017). F-RAG: Generating Atomic Coordinates from RNA Graphs by Fragment Assembly. *J. Mol. Biol.* **429**, 3587–3605.
13. Wang, J., Zhao, Y., Zhu, C., Xiao, Y., (2015). 3dRNAscore: a distance and torsion angle dependent evaluation function of 3D RNA structures. *Nucleic Acids Res.* **43**, e63.
14. Zhao, Y., Huang, Y., Gong, Z., Wang, Y., Man, J., Xiao, Y., (2012). Automated and fast building of three-dimensional RNA structures. *Sci. Rep.* **2**, 734.
15. Wang, J., Xiao, Y., (2017). Using 3dRNA for RNA 3-D Structure Prediction and Evaluation. *Curr. Protoc. Bioinformatics*.
16. Wang, J., Wang, J., Huang, Y., Xiao, Y., (2019). 3dRNA v2.0: An Updated Web Server for RNA 3D Structure Prediction. *Int. J. Mol. Sci.* **20**
17. Zhang, Y., Wang, J., Xiao, Y., (2020). 3dRNA: Building RNA 3D structure with improved template library. *Comput. Struct. Biotechnol. J.* **18**, 2416–2423.

18. Salzman, J. et al, (2012). Circular RNAs Are the Predominant Transcript Isoform from Hundreds of Human Genes in Diverse Cell Types. *Plos One* **7**, e30733
19. Schindewolf, C., Braun, S., Domdey, H., (1996). In vitro generation of a circular exon from a linear pre-mRNA transcript. *Nucleic Acids Res.* **24**, 1260–1266.
20. Starke, S., Jost, I., Rossbach, O., Schneider, T., Schreiner, S., Hung, L.-H., Bindereif, A., (2015). Exon circularization requires canonical splice signals. *Cell Rep.* **10**, 103–111.
21. Wilusz, J.E., (2018). A 360 degrees view of circular RNAs: From biogenesis to functions. *Wiley Interdiscip. Rev. RNA* **9**, e1478.
22. Zuker, M., (2003). Mfold web server for nucleic acid folding and hybridization prediction. *Nucleic Acids Res.* **31**, 3406–3415.
23. Hofacker, I.L., Stadler, P.F., (2006). Memory efficient folding algorithms for circular RNA secondary structures. *Bioinformatics* **22**, 1172–1176.
24. Havel, T.F., (1998). *Distance geometry: Theory, algorithms, and chemical applications*, **120**, pp 723–742.
25. Hubbard, J.M., Hearst, J.E., (1991). Computer modeling 16 S ribosomal RNA. *J. Mol. Biol.* **221**, 889–907.
26. Kabsch, W., (1978). A discussion of the solution for the best rotation to relate two sets of vectors[J]. *Acta Crystallographica Section A: Crystal Physics, Diffraction, Theoretical and General Crystallography* **34**, 827–828.
27. Wang, J., Mao, K., Zhao, Y., Zeng, C., Xiang, J., Zhang, Y., et al., (2017). Optimization of RNA 3D structure prediction using evolutionary restraints of nucleotide-nucleotide interactions from direct coupling analysis. *Nucleic Acids Res.* **45**, 6299–6309.
28. Barrett, S.P., Salzman, J., (2016). Circular RNAs: analysis, expression and potential functions. *Development* **143**, 1838–1849.
29. Kristensen, L.S., Andersen, M.S., Stagsted, L.V.W., et al., (2019). The biogenesis, biology and characterization of circular RNAs. *Nature Rev. Genet.* **20**, 675–691.
30. Liu, C.X., Li, X., Nan, F., Jiang, S., Gao, X., Guo, S.K., et al., (2019). Structure and Degradation of Circular RNAs Regulate PKR Activation in Innate Immunity. *Cell* **177**, 865–880.
31. Busan, S., Weeks, K.M., (2018). Accurate detection of chemical modifications in RNA by mutational profiling (MaP) with ShapeMapper 2. *RNA* **24**, 143–148.
32. Nanduri, S., Carpick, B.W., Yang, Y., Williams, B.R., Qin, J., (1998). Structure of the double-stranded RNA-binding domain of the protein kinase PKR reveals the molecular basis of its dsRNA-mediated activation. *EMBO J.* **17**, 5458–5465.
33. Kim, Y., Park, J., Kim, S., Kim, M., Kang, M.G., Kwak, C., et al., (2018). PKR Senses Nuclear and Mitochondrial Signals by Interacting with Endogenous Double-Stranded RNAs. *Mol. Cell* **71**, 1051–1063.
34. Nallagatla, S.R., Toroney, R., Bevilacqua, P.C., (2011). Regulation of innate immunity through RNA structure and the protein kinase PKR. *Curr. Opin. Struct. Biol.* **21**
35. Huang, S., Zou, X., (2014). A knowledge-based scoring function for protein-RNA interactions derived from a statistical mechanics-based iterative method. *Nucleic Acids Res.* **42**, e55.
36. Mao, K., Wang, J., Xiao, Y., (2020). Prediction of RNA secondary structure with pseudoknots using coupled deep neural networks. *Biophys. Rep.* **6** (4), 146–154.
