# Supplementary material for: PDBx/mmCIF Ecosystem: Foundational Semantic Tools for Structural Biology
Source: J Mol Biol. Author manuscript; Available in PMC 2023 Jun 26. (PMC10292674; doi:10.1016/j.jmb.2022.167599)
Supplement: Article [file NIHMS1907597-supplement-Article.zip › PEPPI--Whole-proteome-Protein-protein-Interaction-Predictio_2022_Journal-of-.pdf]

Web Server

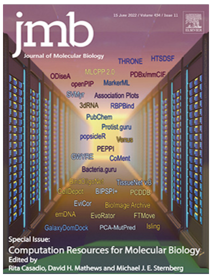

PEPPI: Whole-proteome Protein-protein Interaction Prediction through Structure and Sequence Similarity, Functional Association, and Machine Learning

Eric W. Bell [1], **Jacob H. Schwartz** [1], **Peter L. Freddolino** [1,2*] **and Yang Zhang** [1,2*]

*1 - **Department of Computational Medicine and Bioinformatics,** University of Michigan, Ann Arbor, MI 48109, USA*
*2 - **Department of Biological Chemistry,** University of Michigan, Ann Arbor, MI 48109, USA*

Correspondence to Peter L. FreddolinoYang Zhang: Department of Computational Medicine and Bioinformatics, University of Michigan, 100 Washtenaw Avenue, Ann Arbor, MI 48109-2218, USA (Yang Zhang). Department of Biological Chemistry, University of Michigan, 1150 W. Medical Center Dr., Ann Arbor, MI 48109-0600, USA (Peter Freddolino). *petefred@umich.edu (P.L. Freddolino), zhng@umich.edu (Y. Zhang)*
https://doi.org/10.1016/j.jmb.2022.167530
Edited by Rita Casadio

Abstract

Proteome-wide identification of protein-protein interactions is a formidable task which has yet to be sufficiently addressed by experimental methodologies. Many computational methods have been developed to predict proteome-wide interaction networks, but few leverage both the sensitivity of structural information and the wide availability of sequence data. We present PEPPI, a pipeline which integrates structural similarity, sequence similarity, functional association data, and machine learning-based classification through a naïve Bayesian classifier model to accurately predict protein-protein interactions at a proteomic scale. Through benchmarking against a set of 798 ground truth interactions and an equal number of non-interactions, we have found that PEPPI attains 4.5% higher AUROC than the best of other state-of-the-art methods. As a proteomic-scale application, PEPPI was applied to model the interactions which occur between SARS-CoV-2 and human host cells during coronavirus infection, where 403 high-confidence interactions were identified with predictions covering 73% of a gold standard dataset from PSICQUIC and demonstrating significant complementarity with the most recent high-throughput experiments. PEPPI is available both as a webserver and in a standalone version and should be a powerful and generally applicable tool for computational screening of protein-protein interactions.

© 2022 Elsevier Ltd. All rights reserved.

Introduction

The biological function of many proteins is conferred through their interactions with other proteins. Therefore, to fully understand the function of each protein in an organism, one must first attain a comprehensive network of the protein-protein interactions (PPIs) that occur within the cell. The discovery of critical interactions within this interaction network, or "interactome", can lead to drug development[1] or protein engineering[2–3] targeting these interactions. However, many of these experiments do not guarantee that the interactions detected are, in fact, direct physical contacts between the proteins; some of the earliest databases for PPI prediction involve features that assert only a functional association between proteins.[4] While these databases can be useful for prediction of physical interactions, as all physical interactions are functionally associated, the converse is not true; many biological applications, such as drug target discovery, require knowledge of which proteins come into physical contact. The methods for elucidating these direct physical interactions are at present either prohibitively costly for whole-proteome analysis (such as structure solving

0022-2836/© 2022 Elsevier Ltd. All rights reserved.

or crosslinking mass spectrometry) or are too susceptible to errors (such as yeast-two hybrid).[5] As an alternative, computational methods can be used to model proteome-wide interactions, as well as refine existing interaction datasets.

One of the most straightforward methods of computational interaction prediction is to determine whether the query protein pair is similar to an already known interaction. Many programs directly leverage sequence similarity for this purpose because the sequence comparison operation is quick and sequence data is plentiful.[6–7] However, since structure is more evolutionarily conserved than sequence, structural similarity is much more effective at detecting distantly similar PPIs; methods which leverage this structural information[8–11] grow more powerful as modern structural biology methods such as cryo-EM facilitate the solving of complicated protein complex structures and as computational approaches offer improved accuracy in predicting the folds of individual proteins.[12] In addition, structures provide a clear ground truth as to whether two proteins interact physically; if a solved structure of the interaction exists, the proteins are likely to interact *in vivo*. Therefore, an effective similarity-based program should consider both structural and sequence similarity.

Another common method for PPI prediction is the application of machine learning-based classifiers. In the earlier days of machine learning, the major novelty of machine learning-based PPI predictors was in how they extracted features from the input amino acid sequences in order to create a fixed-length vector that could be utilized in standard machine learning algorithms, such as the conjoint triad method[13] or autocorrelation.[14] As more modern deep learning techniques became available, improved PPI predictions were achieved with features solely from evolutionary profiles[15] or even based on sequence alone.[16–17] Despite these initial studies into deep learning, to our knowledge these methods have yet to demonstrate success in a species agnostic context; more commonly, they are benchmarked by either combining a few species-specific datasets[16] or by training on one species and testing on another.[15,17]

Here, we present a Pipeline for the Extraction of Predicted Protein-protein Interactions (PEPPI), which offers high-accuracy PPI predictions through a consensus of sequence and structural similarity, functional association, and neural network classification. While the source code for PEPPI can be found at https://github.com/ewbell94/PEPPI, an online webserver implementation of this pipeline can be found at https://zhanggroup.org/PEPPI/, which allows users to create PPI predictions from sequences alone. We additionally present an application of PEPPI to make predictions of the inter-species interactome between human host cells and SARS-CoV-2. Through the following benchmarks and examples, we demonstrate that PEPPI is a useful tool for predicting both pairwise and systems-level PPIs.

Results

Pipeline overview and module cross-validation

PEPPI is a protein-protein interaction prediction pipeline which takes in a pair of query sequences and quantifies their likelihood of interaction as a natural log-transformed likelihood ratio (log(LR)) through a consensus of five independent prediction modules (Figure 1). This consensus is determined by a naïve Bayesian classifier model trained on a set of 800 high-confidence interactions from IntAct[18] and 800 curated non-interactions from the Negatome 2.0 database[19] (see Supplementary Methods).

Figure 2(a) presents the 10-fold cross validation performance of each individual module and the full pipeline on this training set. The best-performing individual modules are SPRING and SEQ, which implement structure and sequence-based similarity approaches, respectively. The SPRING module uses the dimeric threading program SPRING[20] to identify dimer structure templates out of a database of interacting proteins extracted from the PDB, while SEQ uses BLAST[21] sequence searching to identify similar interactions in a database of direct interactions identified by high-throughput experiment (HTE) data. These homology-based modules will perform well for any cases which have homologous similarity to existing interactions, which is the case for many true interactions. The next best-performing module is the neural network-based CT module, which transforms the input amino acid sequences into a fixed-length vector according to the conjoint triad method[13] and classifies the resulting vector through a neural network model. This module helps PEPPI retrieve true positive predictions in case there is only loose homologous similarity to existing interactions. The STRING module, a module which extracts various query functional association features from the STRING database,[22] performs relatively poorly on its own. This is expected because of its focus on functional association data instead of physical interaction data and because of its inability to provide data if the interaction is not located in STRING. SPRINGNEG has a nearly identical methodology to SPRING but it performs least well because it searches through a database of non-interacting protein structures. Therefore, a hit in this database will only lower the interaction score because by design it only provides information to filter out functionally associated non-interactions. Overall, the combination of all modules clearly outperforms any single module, demonstrating that the modules are complimentary in classification.

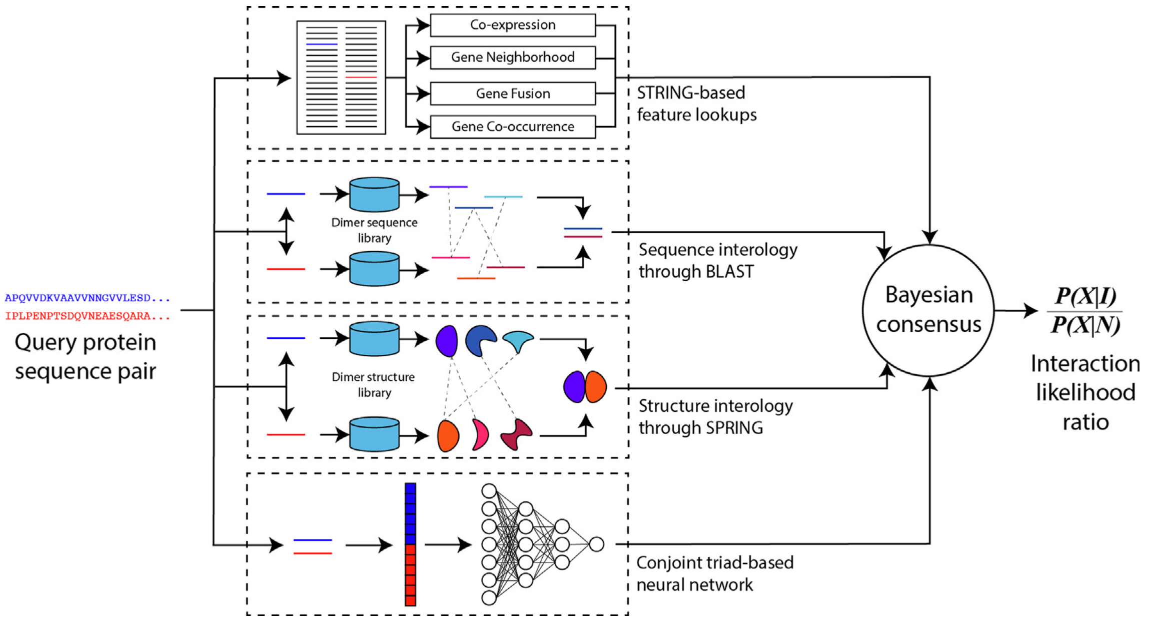

Figure 1. An illustration of the PEPPI pipeline. This pipeline functions by analyzing a pair of input sequences via a series of independent modules, including structure similarity, sequence similarity, neural network classification, and functional association data. These modules are combined using a naïve Bayesian consensus classifier, which provides the final interaction score as a log-likelihood ratio.

PEPPI benchmark and performance

In order to quantify the classification performance of PEPPI against existing methods, we benchmarked PEPPI alongside PRISM,[8] a structure-based similarity predictor, SPRINT,[6] a sequence-based similarity predictor, PIPR,[16] a deep learning predictor which utilizes a combination of recurrent and convolutional layers in its architecture, and D-SCRIPT,[17] a "structure aware" deep learning predictor improving upon PIPR. These benchmarks were performed using a randomly selected test set of 798 interacting structure pairs and an equal number of structure pairs involving chains from the same protein complex but known to not come into physical contact (and thus do not form a physical interaction). All structures of this test set were taken from the PDB and were confirmed to have < 50% sequence identity to the PEPPI training protein pairs. It should be noted that the protein pairs in our dataset are classified through their sequence alone, and therefore, even though many of the corresponding PDB structures contain more than one interaction, each pair of chains can be considered independently.

The results of the PPI predictions are summarized presented in Figure 2(b) and Table S1, where it is shown that PEPPI significantly outperforms all competing methods in terms of area under ROC (AUROC), average precision (similar to area under precision-recall curve, AUPRC), and all but SPRINT in maximum achievable Matthews correlation coefficient (MCC). PRISM had the lowest performance in this benchmark, which is likely due to its outdated interface structure library, which misses many structures which have been solved since its release to the public. Interestingly, the highest performance from a competing program was seen from SPRINT, a sequence motif-based similarity classifier, and not from either D-SCRIPT or PIPR, the more sophisticated deep-learning pipelines. The deep learning architectures of PIPR and D-SCRIPT were originally trained and benchmarked based on performance of species-specific interaction datasets; when these methods were applied to the species agnostic dataset used in our benchmarks, the deep learning methods' ability to accurately classify interactions decreased. As a result, the competing method which draws its conclusions from explicit similarity (SPRINT) outperforms the methods which try to sub-optimally learn the interaction problem itself (PIPR & D-SCRIPT). However, we still found that D-SCRIPT outperforms PIPR, as is consistent with D-SCRIPT's benchmarks.[17]

One particular case of interest in our benchmark dataset is the interaction between chain A and chain B of PDB code 1F3M (corresponding to the N- and C-terminal domains of the human kinase PAK1). While this is a true interaction (and PEPPI correctly classifies it as such), no hit was found by PEPPI in either the sequence or structure databases after homologous template removal, leading to poor scores for those pipelines

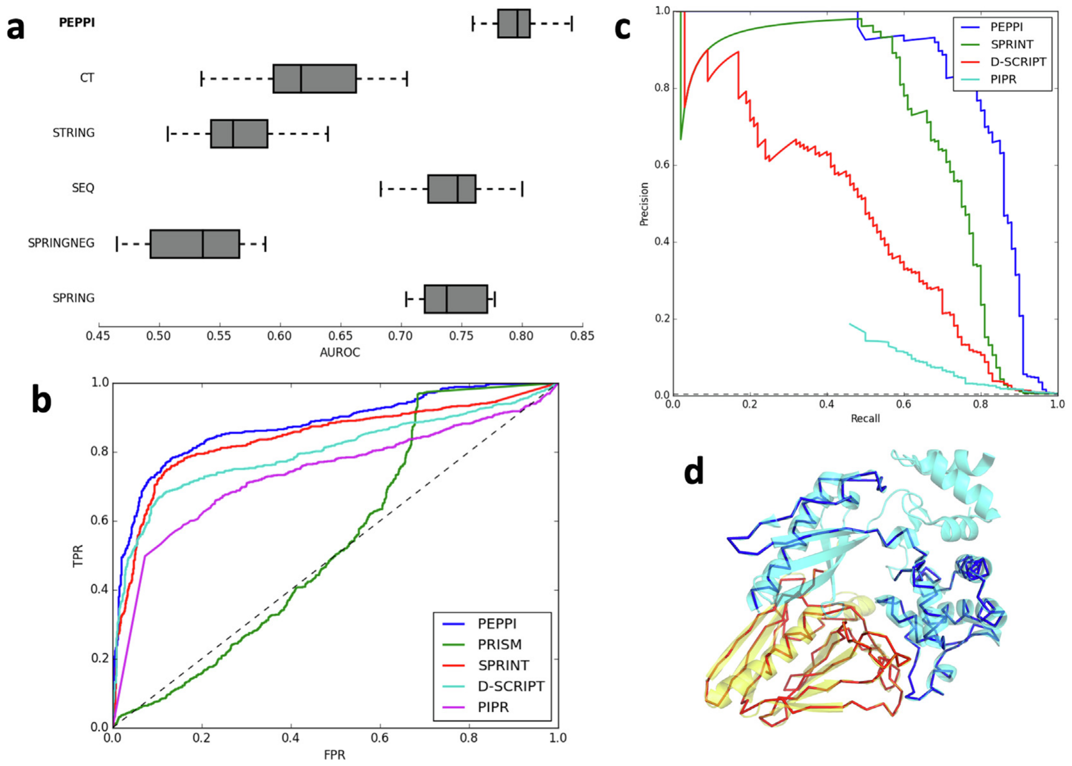

Figure 2. PEPPI benchmark results. (a) 10-fold cross validation AUROC reveals that the full PEPPI pipeline outperforms its component modules: the neural network classifier (CT), the functional association data (STRING), the sequence similarity method (SEQ), the structure similarity method (SPRING), and the non-interaction similarity method (SPRINGNEG). (b) An ROC curve of the performance of PEPPI against PRISM, a structure similarity-based method, SPRINT, a sequence similarity-based method, PIPR, a deep learning-based method, and D-SCRIPT, a structure-aware deep learning-based method on a balanced testing set. The dotted line represents the performance of random classification. (c) Precision-recall curve of the performance of PEPPI against several other comparable programs on an unbalanced testing set. The dotted line represents the performance of random classification. (d) A superposition of an example dimer model (PDB 3CI0; chain J in red, chain K in blue) on its dimer template structure (PDB 5VTM; chain W in yellow, chain X in cyan).

(SPRING: 8.937, STRING: not found, SEQ: 0.129, SPRINGNEG: 4.618). However, this interaction was still classified as positive with a log(LR) of 0.056 due to a high interaction probability from the CT module (0.999), thus demonstrating the utility of CT for rescuing interactions that do not attain significant similarity. On the side of non-interaction classification, the potential interaction of chain G and chain I of 3CJH (corresponding to two Tim13 chains of the yeast Tim8-Tim13 complex) poses an interesting case, as these chains are present in the same complex but do not directly interact with each other. PEPPI was able to classify this as non-interacting (log(LR) = -0.939) where competing programs could not, despite attaining a high SPRING score (34.208), a high CT confidence (1.0), and loose SEQ homology (0.217). The reason for the correct classification is a high score from SPRINGNEG (36.108) which pushes down the total interaction likelihood, thus demonstrating a case where SPRINGNEG's false positive identification ability rescues the pipeline from misclassifying the interaction.

While the previous balanced dataset is convenient for benchmarking, it is not fully reflective of the context in which an interactome prediction algorithm is applied because true interactions are much sparser relative to the total set of pairwise combinations of query proteins in almost all contexts. Therefore, we randomly sampled 100 interacting pairs from the previous test set and paired the 200 chains from these interactions in an all-by-all fashion (excluding self pairings), resulting in an unbalanced test set of 100 true interacting pairs and 19,890 decoy pairs. These decoys are not confirmed non-interactions, but due to the low likelihood of finding interactions by random chance, benchmarking using this dataset is still valuable. Due to the high number of protein pairs, PRISM was excluded from this benchmark because of its slow speed and poor performance on the preceding benchmark. The results of this unbalanced benchmark are presented in Figure 2(c) and Table S2. The outcome is similar to the previous benchmark, with PEPPI outperforming all other programs,

followed most closely by the sequence-based algorithm SPRINT. In this benchmark, however, the superiority of PEPPI is much clearer, as SPRINT is on average more susceptible to false positive detection for comparable recalls. This resulted in a statistically significant difference in max MCC performance between PEPPI and SPRINT, which was not the case on the balanced benchmark set. Also made clearer is the relatively poor performance of PIPR with respect to false positive errors; so many pairs are classified with the highest confidence score that the maximum achievable precision is 0.186. D-SCRIPT again clearly outperforms PIPR but fails to perform as well as the similarity-based methods. One particular case of interest in this benchmark is the true interaction between chain J and chain K of PDB code 3CI0 (part of the type 2 secretion system of enterotoxigenic *E. coli*), an interaction which was detected only by PEPPI (Figure 2(d)). This interaction was able to be detected solely through structural similarity (SPRING: 51.45), with all other modules failing to detect the interaction (STRING: not found, SPRINGNEG: 4.09, SEQ: 0.279, CT: 0.026), leading to a log(LR) of 1.433.

SARS-CoV-2 and human interactome modeling

The COVID-19 pandemic, caused by the SARS-CoV-2 virus, has disrupted the lives of almost every person to some degree, and as of November 2021, over 5 million people have lost their lives to the disease worldwide.[23] SARS-CoV-2 has thus become an essential entity to understand, as our expedient comprehension of this virus translates to the development of therapeutic medicines, such as antiviral drugs and vaccines, for current and future coronavirus infections. One fundamental step towards understanding the function of the virus is to model the virus-host interactome, with which we can begin to identify the purpose of each viral protein through our functional understanding of the human proteins with which each viral protein interacts. To this end, we have predicted the set of interactions which occur between SARS-CoV-2 and human proteins using PEPPI.

Our SARS-CoV-2/Human interactome model consists of 403 interactions whose likelihood ratios were determined to be greater than 1, i.e., interactions which are more likely to be interacting than not. As shown in Figure 3(a)-(b), the SARS-CoV-2 protein which has the highest number of predicted interactions is the Spike protein (86 interactions), followed by the 2′-O-methyltransferase nsp16 (46 interactions), and the RNA polymerase nsp12 (41 interactions). The highest confidence interaction of this network was the Spike/ACE2 interaction (Figure 3(c)), which is expected given the extensive study of this interaction due to its essential role in viral entry.[24] PEPPI also correctly predicted Spike to interact with two other host proteins important to viral entry: Furin, a protease which cleaves Spike during entry of SARS-CoV-2 but is not involved in SARS-CoV-1 entry,[25] and TMPRSS2, a cell-surface protease involved in viral entry of both SARS-CoV-2 and SARS-CoV-1.[24] The PEPPI results also demonstrated the power of structure similarity-based PPI prediction through the prediction of the PARP15-nsp3 interaction (Figure 3(d)); this interaction was predicted with high confidence (log(LR) = 1.435), mainly due to the SPRING module's high confidence score (35.6). The PARP proteins are known to interact with the nsp3 macrodomain in other coronaviruses,[26] so detection of this interaction in our dataset stands as an important validation.

To evaluate PEPPI's overall performance at recapitulating known biology, we constructed a gold standard dataset of known interactions between human and SARS coronavirus proteins from PSICQUIC[27] for comparison. This dataset consisted of 128 interactions, 94 (73%) of which were predicted by PEPPI. In addition, we compared the overlap between our predicted dataset and a recently published high-throughput experimental dataset.[28] PEPPI's predictions only shared one interaction with this dataset (an interaction between MARK3 and ORF9b), but the experimental dataset presents only functional associations due to their use of AP-MS, which is known to pull down entire interacting complexes instead of only the "prey" protein of interest. Compared to PEPPI, the experimental dataset also misses crucial interactions, such as the interactions involving the Spike protein with ACE2, Furin, and TMPRSS2. In fact, only 3 of the 128 (2%) gold standard interactions we isolated from PSICQUIC are present in this dataset. Thus, even in the presence of a high throughput experimental dataset, PEPPI provides a demonstrably useful complement and reveals many direct physical interactions which would otherwise be missed.

Lastly, PEPPI made the potentially significant predictions that nsp3 interacts with the post-translational modifiers NEDD8 and UBD (FAT10). While it is well-documented that the papain-like protease (PLPro) of SARS-CoV-2 nsp3 both deISGylates and deubiquitinates viral proteins to avoid host detection and thus evade immune response,[29] there has been less study of the role of the related, ubiquitin-like, post-transcriptional modifiers NEDD8 and UBD in SARS-CoV-2 disease. NEDD8 tags proteins for degradation, has been implicated in the innate immune response to viruses,[30] and is a target by some viruses for modulation of host immune response.[31] UBD has been shown to be a ubiquitin-independent and cytokine-inducible modifier which targets proteins for proteasomal degradation[32] and has additionally been shown to have roles in viral infection defense.[33] Furthermore, the top structural templates PEPPI found for nsp3/NEDD8 (Figure 3(e)) and nsp3/UBD (Figure 3(f)) were a PLPro in complex with free ubiquitin

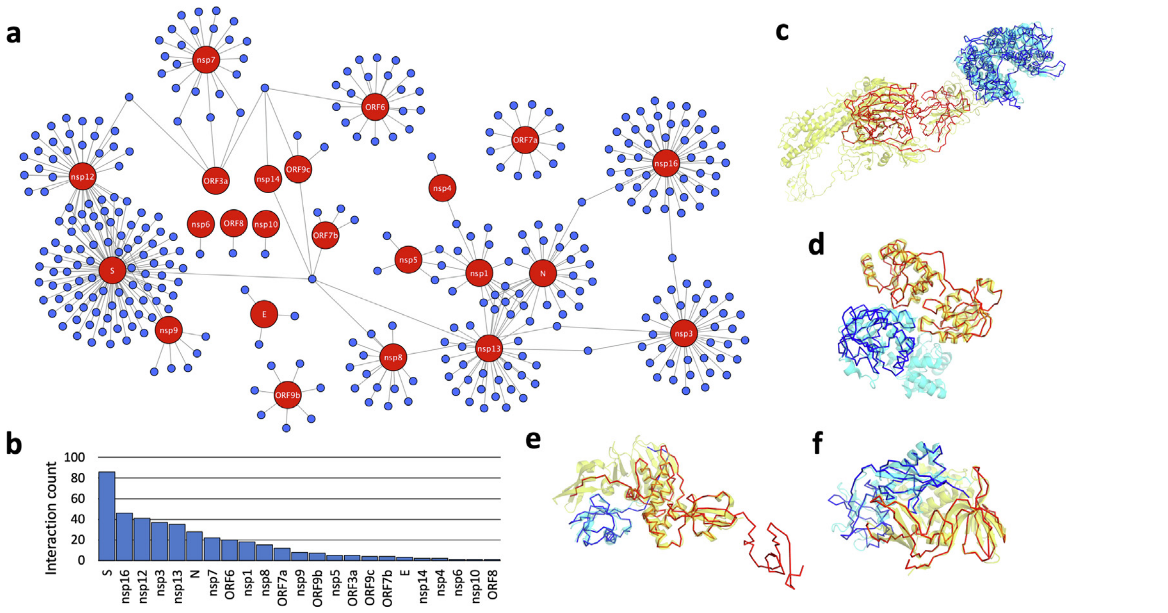

Figure 3. A summary of SARS-CoV-2/human interactome prediction. (a) A network overview of the full interactome of human/SARS-CoV-2 protein pairs, with SARS-CoV-2 proteins colored red and human proteins colored blue. (b) A bar chart of the number of predicted interactions involving each SARS-CoV-2 protein. Proteins which were not predicted to have any interactions were excluded. (c) A superposition of a dimer model of the top-ranked SARS-CoV-2 Spike (in red) and Human ACE2 (in blue) interaction on its dimer template structure (PDB 6ACG; chain D in cyan, chain C in yellow). (d) A superposition of a dimer model of the SARS-CoV-2 nsp3 macrodomain (in red) and human PARP15 macrodomain (in blue) on its dimer template structure (PDB 2W2G; chain A in cyan, chain B in yellow). (e) A superposition of a dimer model of a domain of the SARS-CoV-2 nsp3 (in red) and human NEDD8 (in blue) on its dimer template structure (PDB 5WFI; chain C in cyan, chain A in yellow). (f) A superposition of a dimer model of a domain of the SARS-CoV-2 nsp3 (in red) and human UBD (in blue) on its dimer template structure (PDB 6BI8; chain C in cyan, chain A in yellow).

and a PLPro in complex with the ubiquitin-like and innate-immune-modulating protein ISG15, respectively. We therefore hypothesize that SARS-CoV-2 modulates host innate immune response through interaction of nsp3 with NEDD8 and UBD in a similar manner that nsp3 interacts with ubiquitin.

Discussion & conclusion

We have presented a novel PPI prediction pipeline which demonstrates superior performance relative to other approaches. In addition to performance, this method presents a few unique advantages. Firstly, because the structure-based analysis makes use of threading rather than structural alignment, it is much faster than pipelines which need to explicitly model the input chains, while retaining the flexibility of not requiring an input structure. Second, because structure-based analysis is a component of the pipeline, PEPPI can produce rough structural models of the interactions, which can help deepen biological insights such as interface residue determination and can guide follow-up experiments. Finally, because PEPPI is a consensus model, it is not solely dependent on any one methodology to make its predictions; even if all modules classify an interaction with low confidence, if these classifications agree, the final prediction will have reasonable confidence (as we have shown in a previous example above). In addition, the consensus classifier is constructed such that if any modules are intentionally excluded or fail to produce a score, a prediction can still be made from the remaining modules.

A few shortcomings and assumptions of the pipeline should also be discussed. First, the interaction predictions made in this pipeline are based largely on similarity to known PPIs, and these modules will only detect interactions with similarity to solved structures or to interactions detected in high-throughput screens. Therefore, the method's performance will depend on the coverage of our knowledge of the existing interaction space, which is currently far from fully comprehensive. However, this knowledge will expand as more interactions are discovered, so the power of the similarity-based method will improve over time. Second, because PEPPI is similarity-dependent, if an interaction is predicted between proteins of two given families, all other proteins in those families will likely also be predicted to interact. In this case, interactions involving proteins of the same families can be sorted by LR; the highest rated interaction is the most likely to be true. Finally, this pipeline predicts

the capability for proteins to interact regardless of biological context. As a result, it is possible for some of the interactions predicted here to not exist within the context of the cell due to factors such as incompatible subcellular localization or insufficient expression of the proteins of interest *in vivo*. While this additional biological insight can be useful in pruning the interaction space in a proteome-wide interactome modeling study, it is not explicitly considered in PEPPI's interaction predictions. Therefore, it is worth validating the interactions predicted with this program with more focused small-scale biochemical studies, such as crosslinking mass spectrometry experiments. Despite these shortcomings, the whole proteome interaction networks modeled by PEPPI can help biologists retrieve existing biology and derive novel biology for their system of interest, as we did for the SARS-CoV-2/Human interaction system. Through the understanding of PPI networks on the whole-proteome scale that PEPPI provides, future studies will be able to better understand the systems-level complexity that underpins biological phenomena as well as target individual edges of the network for therapeutic benefit.

Methods

Pipeline overview

The PEPPI pipeline performs predictions through a set of independent modules, each of which score the interaction likelihood in their own way. These modules include a conjoint triad trained neural network, a STRING database lookup module, and two "interology" based modules: a threading-based module using a modified version of SPRING and a sequence-based module using BLAST. Scores from each of these modules are transformed into a ratio of likelihood based on pre-trained score probability distributions, and the final likelihood ratio is calculated as the product of likelihood ratios from each independent module (i.e., the sum of log-likelihood ratios). A full description of the pipeline methodology can be found in the Supplementary Material.

SARS-CoV-2 virus and human host protein sequence collection

The SARS-CoV-2 proteome was collected from the UniProtKB pre-release. Replicase polyprotein 1ab was split into nsp1-16 (excluding nsp11) according to its "chain" regions as described in the "Protein Processing" subsection of its UniProtKB entry; nsp11 was extracted from replicase polyprotein 1a in a similar fashion. As a result, the SARS-CoV-2 sequence set consisted of 31 protein sequences in total. The human proteome, consisting of 20,600 proteins, was also collected from the Uniprot database.[34] All 20600*31 = 638,600 putative interactions were analyzed with PEPPI; any pairs resulting in a log(LR) greater than 0 were classified as interacting. The "gold standard" dataset is comprised interactions listed in the PSICQUIC database[27] annotated as "direct interaction" as of April 2021 between the proteins of either SARS-CoV-1 or SARS-CoV-2 and human proteins, a total of 128 interactions.

CRediT authorship contribution statement

Eric W. Bell: Conceptualization, Methodology, Software, Validation, Formal analysis, Data curation, Writing – original draft, Writing – review & editing. **Jacob H. Schwartz:** Investigation, Writing – original draft, Writing – review & editing. **Peter L. Freddolino:** Conceptualization, Writing – review & editing, Supervision, Project administration, Funding acquisition. **Yang Zhang:** Conceptualization, Writing – review & editing, Supervision, Project administration, Funding acquisition.

DATA AVAILABILITY

The relevant data have been shared as supplementary material, and source code is available with the web server itself.

Acknowledgements

We would like to thank Dr. Gilbert S. Omenn for helpful discussion. This work is supported in part by the National Institute of General Medical Sciences (GM136422, S10OD026825), the National Institute of Allergy and Infectious Diseases (AI134678), and the National Science Foundation (IIS1901191, DBI2030790, MTM2025426). The work in this manuscript was performed in part using the Extreme Science and Engineering Discovery Environment (XSEDE), which is supported by the National Science Foundation (ACI1548562).

Declaration of Competing Interest

The authors declare that they have no known competing financial interests or personal relationships that could have appeared to influence the work reported in this paper.

Appendix A. Supplementary data

Supplementary data to this article can be found online at https://doi.org/10.1016/j.jmb.2022.167530.

Received 30 November 2021;
Accepted 3 March 2022;
Available online 5 March 2022

Keywords:
direct interaction prediction;
interology;
dimer threading;
interactome;
SARS-CoV-2

References

1. D.E. Scott, A.R. Bayly, C. Abell, J. Skidmore, Small molecules, big targets: Drug discovery faces the protein-protein interaction challenge, Nature Publishing Group, 2016. p. 533-50.
2. Bryan, C.M., Rocklin, G.J., Bick, M.J., Ford, A., Majri-Morrison, S., Kroll, A.V., et al., (2021). Computational design of a synthetic PD-1 agonist. *Proc. Natl. Acad. Sci.* **118** e2102164118-e.
3. Huang, X., Pearce, R., Zhang, Y., (2020). De novo design of protein peptides to block association of the SARS-CoV-2 spike protein with human ACE2. *Aging* **12**, 11263–11276.
4. Snel, B., Lehmann, G., Bork, P., Huynen, M.A., (2000). STRING: a web-server to retrieve and display the repeatedly occurring neighbourhood of a gene. *Nucleic Acids Res.* **28**, 3442–3444.
5. Fields, S., (2005). High-throughput two-hybrid analysis: The promise and the peril. *FEBS J.* **272**, 5391–5399.
6. Li, Y., Ilie, L., (2017). SPRINT: Ultrafast protein-protein interaction prediction of the entire human interactome. *BMC Bioinf.* **18**, 1–11.
7. Pitre, S., Dehne, F., Chan, A., Cheetham, J., Duong, A., Emili, A., et al., (2006). PIPE: A protein-protein interaction prediction engine based on the re-occurring short polypeptide sequences between known interacting protein pairs. *BMC Bioinf.* **7**, 1–15.
8. Baspinar, A., Cukuroglu, E., Nussinov, R., Keskin, O., Gursoy, A., (2014). PRISM: A web server and repository for prediction of protein-protein interactions and modeling their 3D complexes. *Nucleic Acids Res.* **42**, W285–W289.
9. R. Hosur, J. Peng, A. Vinayagam, U. Stelzl, J. Xu, N. Perrimon, et al., A computational framework for boosting confidence in high-throughput protein-protein interaction datasets, 2012, pp. 76-.
10. Garzón, J.I., Deng, L., Murray, D., Shapira, S., Petrey, D., Honig, B., (2016). A computational interactome and functional annotation for the human proteome. *eLife.* **5**, 1–27.
11. Gong, W., Guerler, A., Zhang, C., Warner, E., Li, C., Zhang, Y., (2021). Integrating Multimeric Threading With High-throughput Experiments for Structural Interactome of Escherichia coli. *J. Mol. Biol.* **433**, 166944
12. Jumper, J., Evans, R., Pritzel, A., Green, T., Figurnov, M., Ronneberger, O., et al., (2021). Highly accurate protein structure prediction with AlphaFold. *Nature* **596**, 583–589.
13. Shen, J., Zhang, J., Luo, X., Zhu, W., Yu, K., Chen, K., et al., (2007). Predicting protein-protein interactions based only on sequences information. *PNAS* **104**, 4337–4341.
14. Guo, Y., Yu, L., Wen, Z., Li, M., (2008). Using support vector machine combined with auto covariance to predict protein-protein interactions from protein sequences. *Nucleic Acids Res.* **36**, 3025–3030.
15. Hashemifar, S., Neyshabur, B., Khan, A.A., Xu, J., (2018). Predicting protein-protein interactions through sequence-based deep learning. *Bioinformatics* **34**, i802–i810.
16. M. Chen, C.J.T. Ju, G. Zhou, X. Chen, T. Zhang, K.W. Chang, et al., Multifaceted protein-protein interaction prediction based on Siamese residual RCNN, 14 ed. p. i305-i14.
17. Sledzieski, S., Singh, R., Cowen, L., Berger, B., (2021). D-SCRIPT translates genome to phenome with sequence-based, structure-aware, genome-scale predictions of protein-protein interactions. *Cell Syst.* **12** 969-82.e6.
18. Orchard, S., Ammari, M., Aranda, B., Breuza, L., Briganti, L., Broackes-Carter, F., et al., (2014). The MIntAct project - IntAct as a common curation platform for 11 molecular interaction databases. *Nucleic Acids Res.* **42**, D358–D363.
19. Blohm, P., Frishman, G., Smialowski, P., Goebels, F., Wachinger, B., Ruepp, A., et al., (2014). Negatome 2.0: A database of non-interacting proteins derived by literature mining, manual annotation and protein structure analysis. *Nucleic Acids Res.* **42**, D396–D400.
20. Guerler, A., Govindarajoo, B., Zhang, Y., (2013). Mapping monomeric threading to protein-protein structure prediction. *J. Chem. Inf. Model.* **53**, 717–725.
21. Altschul, S.F., Gish, W., Miller, W., Myers, E.W., Lipman, D.J., (1990). Basic local alignment search tool. *J. Mol. Biol.* **215**, 403–410.
22. Szklarczyk, D., Gable, A.L., Lyon, D., Junge, A., Wyder, S., Huerta-Cepas, J., et al., (2019). STRING v11: Protein-protein association networks with increased coverage, supporting functional discovery in genome-wide experimental datasets. *Nucleic Acids Res.* **47**, D607–D613.
23. Organization WH, WHO Coronavirus (COVID-19) Dashboard, WHO Coronavirus (COVID-19) Dashboard With Vaccination Data, 2021, pp. 1-5.
24. Hoffmann, M., Kleine-Weber, H., Schroeder, S., Krüger, N., Herrler, T., Erichsen, S., et al., (2020). SARS-CoV-2 Cell Entry Depends on ACE2 and TMPRSS2 and Is Blocked by a Clinically Proven Protease Inhibitor. *Cell* **181** 271-80.e8.
25. Johnson, B.A., Xie, X., Bailey, A.L., Kalveram, B., Lokugamage, K.G., Muruato, A., et al., (2021). Loss of furin cleavage site attenuates SARS-CoV-2 pathogenesis. *Nature* **591**, 293–299.
26. Grunewald, M.E., Chen, Y., Kuny, C., Maejima, T., Lease, R., Ferraris, D., et al., (2019). The coronavirus macrodomain is required to prevent PARP-mediated inhibition of virus replication and enhancement of IFN expression. *PLoS Pathogens* **15** e1007756-e.
27. B. Aranda, H. Blankenburg, S. Kerrien, F.S.L. Brinkman, A. Ceol, E. Chautard, et al., PSICQUIC and PSISCORE: Accessing and scoring molecular interactions, Nature Publishing Group, 2011, pp. 528–529.
28. Gordon, D.E., Jang, G.M., Bouhaddou, M., Xu, J., Obernier, K., White, K.M., et al., (2020). A SARS-CoV-2 protein interaction map reveals targets for drug repurposing. *Nature* **583**, 459–468.
29. Shin, D., Mukherjee, R., Grewe, D., Bojkova, D., Baek, K., Bhattacharya, A., et al., (2020). Papain-like protease regulates SARS-CoV-2 viral spread and innate immunity. *Nature* **587**, 657–662.
30. K. Han, J. Zhang, Roles of neddylation against viral infections, Nature Publishing Group, 2018, pp. 292–294.
31. Kumar, R., Mehta, D., Mishra, N., Nayak, D., Sunil, S., (2021). Role of host-mediated post-translational

modifications (PTMS) in RNA virus pathogenesis. *Multidisciplinary Digital Publishing Institute*, 1–26.

32. Hipp, M.S., Kalveram, B., Raasi, S., Groettrup, M., Schmidtke, G., (2005). FAT10, a Ubiquitin-Independent Signal for Proteasomal Degradation. *Mol. Cell. Biol.* **25**, 3483–3491.

33. Basler, M., Buerger, S., Groettrup, M., (2015). The ubiquitin-like modifier FAT10 in antigen processing and antimicrobial defense. *Pergamon*, 129–132.

34. The UniProt Consortium, (2021). UniProt: the universal protein knowledgebase in 2021. *Nucleic Acids Research* **49** (D1), D480–D489. https://doi.org/10.1093/nar/gkaa1100.
