# Supplementary material for: PDBx/mmCIF Ecosystem: Foundational Semantic Tools for Structural Biology
Source: J Mol Biol. Author manuscript; Available in PMC 2023 Jun 26. (PMC10292674; doi:10.1016/j.jmb.2022.167599)
Supplement: Article [file NIHMS1907597-supplement-Article.zip › emDNA---A-Tool-for-Modeling-Protein-decorated-DNA-Loops_2022_Journal-of-Mole.pdf]

Research Article

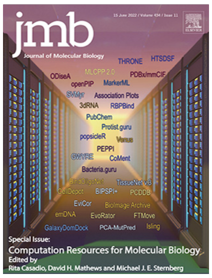

emDNA – A Tool for Modeling Protein-decorated DNA Loops and Minicircles at the Base-pair Step Level

Robert T. Young, Nicolas Clauvelin and Wilma K. Olson *

Department of Chemistry & Chemical Biology, *Center for Quantitative Biology, Rutgers, The State University of New Jersey, Piscataway, NJ 08854, USA*

Correspondence to Wilma K. Olson: *wilma.olson@rutgers.edu (W.K. Olson), @SciNerdRobb (R.T. Young)*
https://doi.org/10.1016/j.jmb.2022.167558
Edited by David Mathews

Abstract

Computational modeling of nucleic acids plays an important role in molecular biology, enhancing our general understanding of the relationship between structure and function. Biophysical studies have provided a wealth of information on how double-helical DNA responds to proteins and other molecules in its local environment but far less understanding of the larger scale structural responses found in protein-decorated loops and minicircles. Current computational models of DNA range from detailed all-atom molecular dynamics studies, which produce rich time and spatially dependent depictions of small DNA fragments, to coarse-grained simulations, which sacrifice detailed physical and chemical information to treat larger-scale systems. The treatment of DNA used here, at the base-pair step level with rigid-body parameters, allows one to develop models hundreds of base pairs long from local, sequence-specific features found from experiment. The emDNA software takes advantage of this framework, producing optimized structures of DNA at thermal equilibrium with built-in or user-generated elastic models. The program, in combination with the case studies included in this article, allows users of any skill level to develop and investigate mesoscale models of their own design. The functionality of emDNA includes a tool to incorporate experiment-specific configurations, e.g., protein-bound and/or melted DNA from known high-resolution structures, within higher-order 3D models by fixing the orientation and position of user-specified base pairs. The software provides a new avenue into multiscale genetic modeling, giving a wide range of users a deeper understanding of DNA mesoscale organization and the opportunity to pose new questions in genetic research. The publicly available emDNA software, including build instructions and usage information, is available on GitHub (https://nicocvn.github.io/emDNA/).
© 2022 Elsevier Ltd. All rights reserved.

Introduction

The DNA in living systems simultaneously exists in a largely inactive B-form double-helical state with bases protected from damage and an active, highly varied state with localized structural distortions necessary for reading, expressing, and re-arranging the encoded genetic information. Access to this information occurs in the context of a tightly packaged, higher-order 3D structure, with DNA wrapped in small loops around various architectural proteins[1,2] and the protein-decorated DNA chains folding into successively larger looped forms.[3,4] The complex interplay among the local features of DNA and protein, the larger-scale biomolecular organization, and the resulting genetic function is of fundamental importance to an understanding of biology.

Physical understanding of how various proteins and small molecules work in combination with

DNA to control the overall structure and function of the genetic material is beginning to come to light. The growing library of high-resolution structures[5] is making it possible to decipher the effects of base-pair sequence, bound proteins, and small molecules on the local architecture of DNA.[6–8] State-of-the-art computational treatments that take direct account of these local DNA features help to envision ways in which higher-order, constrained systems might assemble and function.[9–18] Access to these 3D models has been limited until now to the experts who have been developing the computational technologies. The general user has not been able to introduce arbitrary constraints in DNA models, such as placing a specific protein within a loop or a closed circular DNA structure. Popular 3D modeling tools currently allow users to generate simple, ligand-free circular structures for atomic-level simulations, e.g., Nucleic Acid Builder,[19] or to construct protein-decorated fragments along unconstrained, linear DNA chains, e.g., 3DNA.[20,21] The capability to impose constraints both on the ends and within the DNA molecule opens new avenues to study large- and small-scale dynamics of genetic systems.[22]

Our new method for determining optimized configurations of DNA at the level of successive base-pair steps makes it possible to model the presence of bound proteins on spatially constrained DNA and to examine the structural landscape of DNA loops and minicircles hundreds of base pairs long.[16–18] This is done using emDNA, a command-line software package that gives users the capability to develop spatially constrained, energy-optimized DNA models using previously described mathematical methods.[16] A hallmark of emDNA is the level of structural control users have over the calculations, particularly in the ability to freeze specific tracts of DNA during optimization, thereby maintaining details that are useful in elucidating the structural response of free DNA to protein-binding and other localized deformations. In addition, the software allows for sequence-dependent control via intrinsic base-pair step-level parameters. These parameters can range from simplistic – an idealized model of isotropic B DNA – to a fully sequence-dependent, knowledge-based potential derived from high-resolution structural information.

Case studies presented in this work highlight applications of emDNA to a well-characterized minicircle with unique sequence-dependent features.[23–26] Initially, readers learn how to generate and optimize circular forms of DNA with elastic models built into the emDNA software. Readers then build a protein-decorated DNA minicircle utilizing a linear ramping technique with DNA models collected from experimentation, illustrating how the positioning of a protein with respect to base-pair sequence influences global folding. Finally, readers use the ramping tool to generate multiple looped configurations starting from a minicircle, seeing how changes in the end points alter the pathway of the loop. The software is stored for public access at the emDNA GitHub repository (https://nicocvn.github.io/emDNA/), which contains download instructions and a detailed user guide. Commands, options, and arguments for each case study are supplied with additional support files in the supplementary information and on the repository site.

Methods

The emDNA software package works with DNA configurations built from rigid-body base-pair frames. These reference frames approximate the atomic structure of each Watson-Crick base pair as a rectangular plane (Figure 1(A)). This simplification reduces the description of the ~60 atoms in a base pair to a reference frame with 12 values — the 3 × 1 position vector of the origin and the 3 × 3 orthonormal matrix describing the directions of the local axes. These reference frames can then be used to generate a vector of six base-pair step parameters {tilt, roll, twist, shift, slide, rise} that describes the relative orientation and displacement of successive base pairs[27] (Figure 1(A)). The interconversion between reference frames and base-pair steps entails straightforward linear algebraic methods,[16] conveniently implemented in software such as 3DNA[20] and in an internal parser within emDNA (see below).

Use of *emDNA*[1] requires a file specifying the initial configuration of DNA and a series of options to describe the end conditions and elastic parameters guiding the optimization of structure[2] (Figure 1(B)). The initial file takes the form of either a collection of base-pair reference frames or a collection of step parameters. The step-parameter file (*--x3DNA-bp-step-params-input*) contains the base-pair sequence as well as the step parameters. The reference-frame files can be organized with or without the sequence (*--x3DNA-bp-input* or *--bp-list-input*, respectively) as sequence is not a requirement if the chain is modeled as a simple homopolymer. These files can be made directly from Protein Data Bank (.pdb) files using 3DNA,[20] either through the interactive 3DNA web interface (web.x3DNA.org) or with the open-source code downloadable from the 3DNA Forum (https://forum.x3dna.org).

The elastic parameters used in the optimization combine a set of rest states with elastic stiffness constants associated with the six base-pair step parameters. The energy at each step is proportional to the sum of the squared differences

1 The italicized font throughout the text represents command-line code and specific file names.

2 Users can get a list of specific options (or flags) with brief descriptions by executing *emDNA --help* in the command-line terminal.

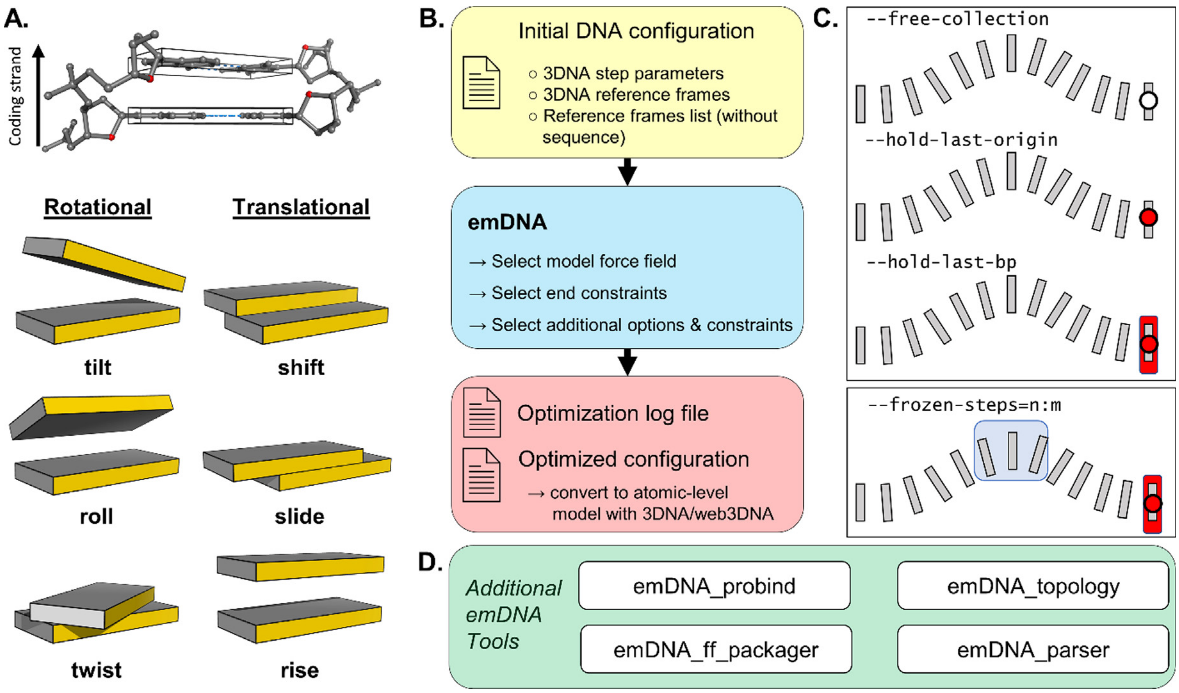

Figure 1. (A) An all-atom model of a DNA base-pair step with O4′ atoms highlighted in red and hydrogen bonds between Watson-Crick base pairs in blue. The orientation and displacement of successive base pairs, enclosed in rectangular slabs (*top*), are described in terms of three rotational and three translational parameters. Schematics (*bottom*) illustrating positive values of each parameter are positioned such that the coding strand (i.e., the specified sequence as opposed to the reverse complement) is at the left and the minor-groove edge (gold) faces the reader. (B) The standard *emDNA* workflow. Starting with an initial configuration of DNA (yellow), the user specifies a series of commands and options for optimization (blue). The output (pink) includes two files — the optimization log file and the optimized configuration file, which can be used with *emDNA_parser* and software such as 3DNA to produce a .pdb file for 3D molecular modeling. (C) Choice of base-pair constraints. (*top*) The last base pair is either free to move (white circle), fixed at its origin but allowed to rotate freely (red circle), or spatially fixed (red circle and slab). (*bottom*) An additional constraint can be used to freeze user-defined steps during optimization. (D) The set of additional tools built into emDNA, each of which is independent of the others. See main text for further details.

between the rigid-body parameters in the modeled DNA configuration and the rest-state values associated with a linear equilibrium structure.[16] The rest-state values and stiffness constants, which can depend upon sequence, allow the user to incorporate specific local conformational features, such as intrinsic curvature,[28] within the DNA model. The total energy of a configuration is the sum of the energies over all deformable steps. The current version of the software includes a simple homopolymeric model of idealized B DNA[29] and a fully sequence-dependent model derived from high-resolution crystal structures.[6] The minimization of total energy uses a gradient-descent method involving the step parameters. Users can customize the minimization parameters, including the maximum number of iterations in the optimization and the gradient threshold used to finalize the calculation. Once an optimization is complete, emDNA produces a configuration file which can then be converted with 3DNA into a .pdb file for 3D molecular modeling.

A highlight of using emDNA is the level of control the user has over the optimization process, particularly the positions and orientations of the 3′-ends of the DNA fragments. If the optimization were initiated with every base-pair step free to move, the result would be a linear configuration identical to the equilibrium rest state. This option, initiated with the *--free–collection* flag, is useful in troubleshooting and offers insights into structural features, such as curvature, that may depend upon sequence (Figure 1(C), top). Restricting the DNA collection during optimization requires one of two flags, which hold either the last base pair in place (*--hold–last–bp*) or only the base-pair origin (*--hold–last–origin*). The latter flag makes it possible to treat nicked minicircles and loops. Any optimization initiated with emDNA requires one of these three commands. The molecular origin remains fixed at the 5′–end throughout.

The level of base-pair control is not restricted to the ends of the chain. Use of the *--frozen–steps* option (Figure 1(C), bottom) allows users to

specify one or more subsets of base-pair steps that should remain constant during optimization. This is useful when a tract within the initial configuration represents a particular DNA model of interest, such as a local region with a highly distorted conformation or a protein-DNA binding interaction. The *--frozen–steps* option is also useful in positioning spatially-distant base pairs such as those at the ends of a protein-mediated loop.

The optimization of a collection of DNA base pairs is one feature of the emDNA software. The software also includes a data-parsing tool (*emDNA_parser*) to convert between reference frames and step parameters (Figure 1(D)). If users want to customize modeling with specific elastic parameters, such as highlighting sequence-specific roll and/or twist values in curved A-tracts,[30,31] the software contains a tool that builds a binary file from selected step parameters and elastic constants (*emDNA_ff_packager*). Users can get additional structural insights into the modeled DNA configurations from the writhe (Wr), total twist (Tw), and linking number (Lk) values obtained at any stage of optimization with the built-in topology tool (*emDNA_topology*). The writhe is a standard measure of the global configuration of a constrained DNA molecule and is used with the intertwining of the double-helical strands (total twist) to determine the linking number (Lk = Wr + Tw), a measure of DNA supercoiling.[32,33] Finally, users can design and build protein-decorated models with a built-in modeling tool that utilizes a linear ramping technique (*emDNA_probind*), which incorporates a specified DNA configuration into some larger target base-pair collection.[16] Each tool is independent of the others, allowing users full control over when and how to collect selected information within the workflow. The following case studies present examples of how these tools can be used.

Case studies

Elastic energy minimization with emDNA yields an optimized configuration of DNA and a detailed log file that contains energetic values (in units of $k_B T$) (Figure 1(B)). The energetic data include initial and final energy values and a detailed 6×6 matrix of contributions to the elastic energy from all pairs of step parameters, e.g., a diagonal matrix when optimizing with the built-in homopolymeric *IdealDNA* model. The collected energies exclude any region(s) frozen during optimization. Additional structural information regarding either the initial or optimized configurations can be obtained at any stage of the optimization using the aforementioned *emDNA_parser* and *emDNA_topology* tools (Figure 1(D)). The initial and final energies are presented in Supplemental Table S1 for the following case studies.

The three case studies highlight the functionality of emDNA. Each sample application starts with a perfectly circular configuration, either a 336-bp sequence used in gene therapy[34] (*N336.par*) with Lk 32 or a 195 bp segment of the longer sequence (*circ195.par*) with Lk 18. Both circles bear a partially curved 180-bp *att*R region that is a remnant of the λ integrase-mediated recombination process used to generate the larger minicircle (Figure 2(A) in red).[35] An important detail regarding the design and use of DNA minicircle models concerns the last base-pair step. For circular DNA, the ends of the collection must be joined in perfect register. This is achieved by using a virtual last base pair, where the first base-pair reference frame is copied to the end of the collection, so that a final, closing step is generated. Users can introduce their own starting circular configurations or make use of a method that allows for customized chain length, linking number, and sequence, included in Supplemental Protocol S1 and Supplemental Table S2.

The first case study involves optimization of the *N336.par* minicircle with the commands found in Figure 2(B).[3] The examples illustrate the effects of two different sets of elastic parameters on the same initial configuration: the *IdealDNA* model[29] and the built-in sequence-specific model (*Olson1998*).[6] Each optimization specifies that the position and orientation of the final base pair must be held constant to maintain the end-to-end connection (*--hold–last–bp*). An optional flag is used to customize the name of the output files (*--output–file*); without the flag, the naming defaults to *emDNA_minim*. Once an optimization is complete, the directory will contain an optimized step-parameter file (always ending in *_opt.txt*) and a detailed log file. Topological data for each optimized structure can be obtained using the *emDNA_topology* tool with the *--virtual–last–bp* flag, which specifies that the two ends of the chain are connected. Although both models are similar in general configuration and final energy (Supplemental Table S1), the examples in Figure 2 (C) reveal a regular planar circle (Wr = 0) for the *IdealDNA* chain and a curved circle with an out-of-plane bend (Wr = 0.05) for the sequence-dependent chain. Note the greater localized distortion within the *att*R region of the latter model (Figure 2 (C) right, red). The circular plot in the figure highlights the variation of twisting and bending at individual base-pair steps along the sequence-dependent model compared to the uniform values associated with *IdealDNA* (34.3° and 1.1°, respectively). The difference in topological parameters between the two models arises from the greater average bend ($2.9°_{\pm 1.7°}$) and the reduction in local twist ($34.2°_{\pm 2.6°}$) found in the sequence-dependent chain.

3 A note for beginners: the start of a command line is denoted with a '$' character. From there, a command is typed as a single line. The '\' character is used in Bash script files to break up a single command line into a format that is easier to read, write, and edit. The '< >' symbols used here denote unique strings and lists to be designated by the user.

A.

```
TTTCACCGAT ATCACCGAAA CGCGCGAGGC AGCTGTATGG CATGAAAGAG TTCTTCCCGG AAAACGCGGT GGAATATTTC GTTTCCTACT
ACGACTACTA TCAGCCGGAA GCCTATGTAC CGAGTTCCGA CACTTTCATT GAGAAAGATG CCTCAGCTCT GTTACAGGTC ACTAATACCA
TCTAAGTAGT TGATTCATAG TGACTGCATA TGTTGTGTTT TACAGTATTA TGTAGTCTGT TTTTTATGCA AAATCTAATT TAATATATTG
ATATTTATAT CATTTTACGT TTCTCGTTCA GCTTTTTTAT ACTAACTTGA GCGAAACGGG AAGGGT
```

B.

```
$ emDNA \
--x3DNA-bp-step-params-input=N336.par \
--DNA-seqdep-model=IdealDNA --hold-last-bp \
--output-name=N336_ideal_circle
$ emDNA_topology \
--x3DNA-bp-step-params-
input=N336_ideal_circle_opt.txt
>topo_N336_ideal_circle_opt.txt \
--virtual-last-bp
```

```
$ emDNA \
--x3DNA-bp-step-params-input=N336.par \
--DNA-seqdep-model=Olson1998 --hold-last-bp \
--output-name=N336_seqdep_circle
$ emDNA_topology \
--x3DNA-bp-step-params-
input=N336_seqdep_circle_opt.txt
>topo_N336_seqdep_circle_opt.txt \
--virtual-last-bp
```

C.

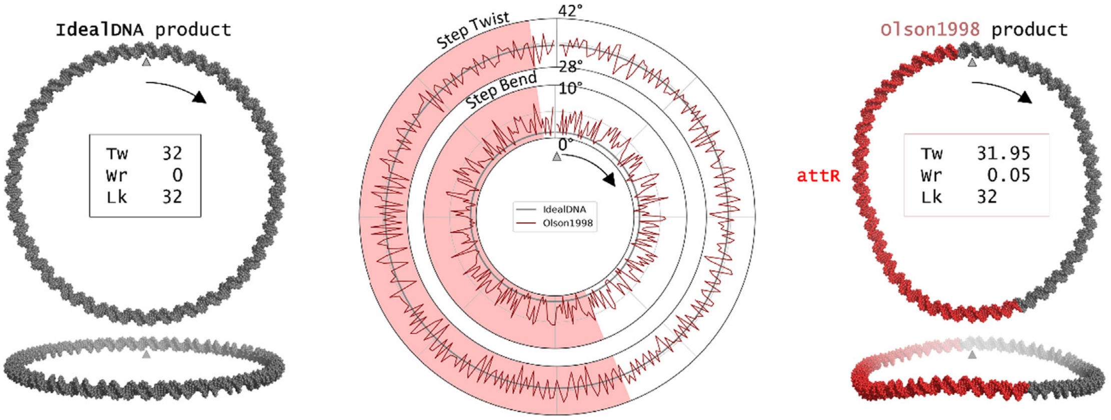

Figure 2. (A) A 336-bp sequence used in genetic studies containing a 180-bp *att*R region (red).[24] (B) The emDNA commands and options used in optimizing the file of base-pair step parameters (*N336.par*) bearing the preceding sequence. Each code block (beige) starts with the *emDNA* command followed by options to select the initial configuration file and to specify the built-in elastic parameters to be used in optimization: (*left*) the homopolymeric *IdealDNA* or (*right*) the sequence-dependent *Olson1998* model. The last two options specify the end constraint that fixes the last base pair in space and define the output name. The instructions end with the *emDNA_topology* command that characterizes the global topology with the optimized step-parameters file as the input. The *–virtual-last-bp* option ensures that the chain ends are connected. The '>' symbol is required to generate a file with topological values; without this, the unsaved data will only appear on the command-line terminal window. (C) Two views, rendered in PyMol,[44] of each optimized minicircle below its respective code block. The lower images are obtained by rotating the top images about the horizontal axis by $-80°$. The *att*R sequence is highlighted in red in the model that accounts for sequence (*right*). The values of the global topological parameters are listed at the center of each model. The circular plot of optimized twist (*exterior*) and bend (*interior*) angles between successive base pairs along the sequence (*center*) highlights the local differences between the *IdealDNA* (grey) and *Olson1998* (maroon) optimized models. The Δ symbol in each image denotes the first base pair and the black arrow the 5′-3′ direction of the sequence.

The next two case studies highlight how emDNA can incorporate specific models into a naked DNA fragment using the *emDNA_probind* command. This tool applies a linear ramping function that shapes select regions of a target base collection as if bound to protein while maintaining the end-to-end distance and rotation between DNA fragment ends.[16] This is done by iteratively incrementing the base-pairstep parameters within the target region(s) to match those in known protein-DNA complexes, optimizing all non-targeted base-pair steps after each iteration. This tool requires three initial files: the initial DNA configuration, the model of protein-bound DNA to be incorporated, and a list of necessary emDNA options. This list, found in the boxed inset in Figure 3(A), is described in further detail in Supplemental Table S3. Execution of the ramping technique simply entails typing the name of the file containing the list of options after *emDNA_probind* in the command line (see the first command at the top left of Figure 3(A)).

The second case study uses *emDNA_probind* to produce a circular minichromosome bearing the superhelical pathway taken from a nucleosome-core-particle structure.[2] Here, the *N336.par* structure acts as the initial *base_collection* with the first 141 bp, outside the *att*R sequence region, as the site of protein incorporation (depicted in gold and red, respectively, in Figure 3(B)). The central 141 bp of the currently best-resolved nucleosome core particle structure (Protein Data Bank file 1KX5)[2] serves as the *protein_collection* model

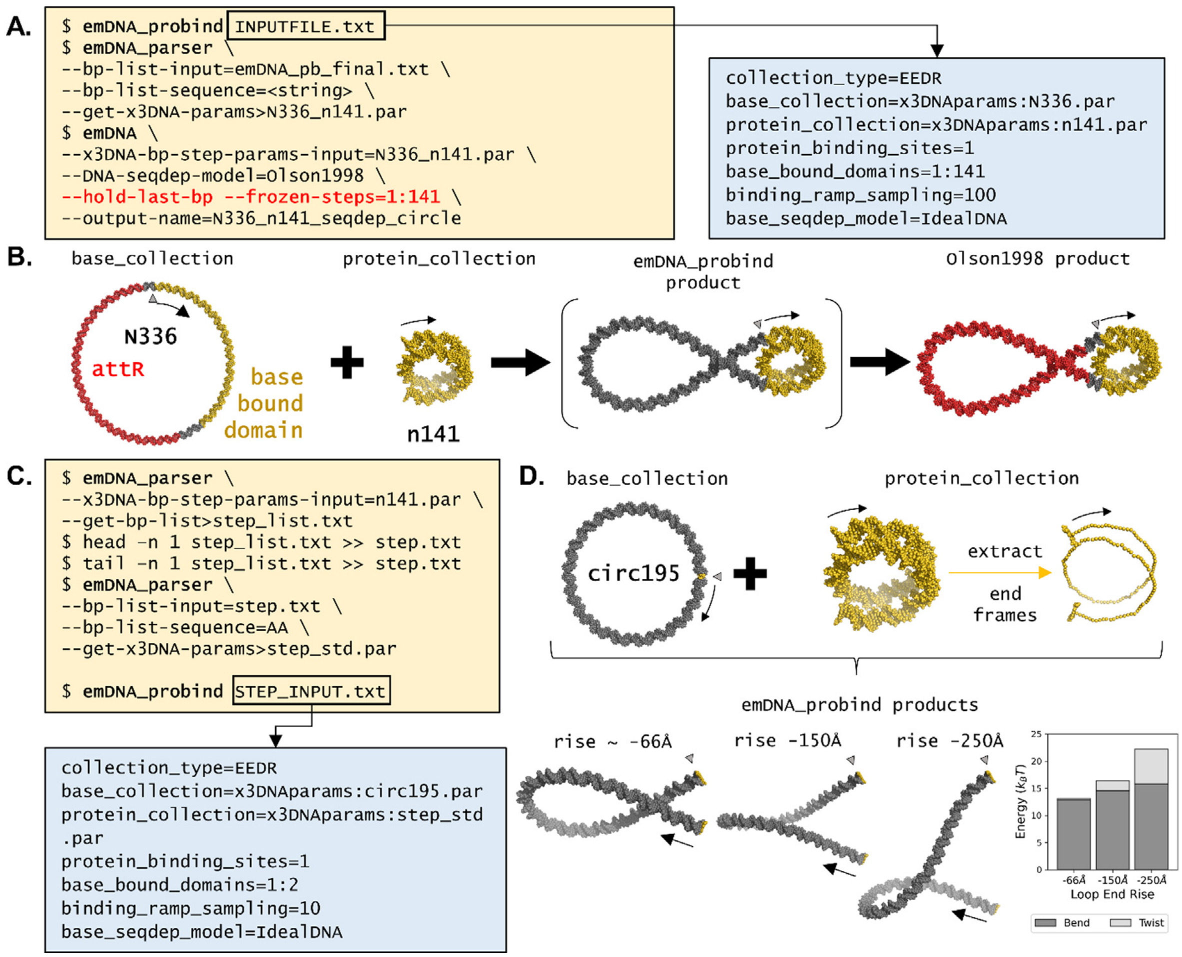

Figure 3. (A) Commands and options required for producing a 336-bp nucleosome-decorated minicircle. The three command lines (i) execute the ramping procedure using the *INPUTFILE.txt* commands list (*right inset*), (ii) convert the ramped output file from a base-pair list file to a step-parameter file with the sequence in Figure 2A (<*string*> replaced by sequence in Figure 2A), and (iii) optimize the ramped product with sequence-dependent elastic parameters. The –*hold–last–bp* and –*frozen–bp* options respectively maintain the circular configuration and the ramped protein-bound region. See Supplemental Table S3 for details of options found in inset. (B) Molecular schematic of the ramping process starting from an ideal circular pathway and a 141-bp model of nucleosomal DNA. The product of the ramping step, shown in brackets, can be further used for optimizing sequence-dependent features (far right). The color coding highlights the *att*R sequence (red) and the nucleosome model/target (Protein Data Bank file 1KX5 in gold). (C) Commands and options required for producing a 195-bp loop: (i) convert the step-parameter file of *n141.par* to a reference frame list file (*step_list.txt*); (ii) extract the first and last base-pair frames from *step_list.txt* to produce a new reference frame file (*step.txt*); (iii) convert *step.txt* to a new step-parameter file *step_std.par*, and (iv) insert *step_std.par* into the minicircle using the *STEP_INPUT.txt* command list file (blue box below). Unlike the previous ramping application, this example requires only a few ramped iterations (here 10). (D) (*top*) Molecular schematic of the ramping process starting from a 195-bp ideal circular pathway and a virtual step extracted from the ends of the 141-bp model. The associated case study (*bottom left*) explores the effect of change in the virtual step rise parameter from ~-66 Å in the standard model to values of 150 Å and50 Å. The bar chart (*bottom, right*) highlights the increase in bend energy (*bottom, dark grey*) and twist energy (*top, light grey*) with change in rise. See Figure 2 for descriptions of the Δ symbols and black arrows.

(small gold superhelix in Figure 3(B), *n141.par*). Application of the *IdealDNA* model over 100 ramped iterations produce the optimized protein-decorated structure found within brackets in Figure 3(B). Three output data files accompany the ramping procedure, including: a list of the reference frames of the final optimized state (*emDNA_pb_final.txt*) without sequence information; a list of the reference frames for all intermediate structures (*emDNA_pb_confs.txt*); and a list of the total energy, gradient normal, number of optimization iterations, and return codes for each intermediate structure (*emDNA_bp_stats.txt*). See Supplemental Video S1 to visualize the linear ramping process. Further optimization with emDNA following ramping requires an additional option (--*frozen–steps*, seen

in Figure 1(C)) to exclude regions with fully bound proteins from the energy calculation.

Consideration of DNA sequence after use of the ramping procedure requires an additional step. The user needs the *emDNA_parser* tool to convert from the optimized reference-frame-list file to a structure file that includes sequence information, such as a step-parameter file. The sequence in Figure 2(A) is added using *--bp-list-sequence*, making sure to copy the first base (a thymine) to the end to account for the circular configuration for the <*string*> sequence input. This case study yields the sequence-dependent minichromosomal structure depicted at the far right of Figure 3(B), again highlighting the *att*R sequence region in red and the bound nucleosomal DNA in gold. The uptake of a nucleosome is limited here to the 156-bp segment outside the *att*R region, allowing for limited sliding of the 141-bp superhelical pathway along the minichromosome. The sliding of the nucleosome along this 15-bp region has pronounced effects on the energy and configuration of the protein-free loop (Supplemental Figure S1). The *att*R sequence is needed to make the minicircle and for this reason the nucleosome-positioning sequence is located outside this region.

The final case study showcases how users can construct and study the influence of chain end conditions on DNA loops. The first step entails generation of a set of virtual step parameters that describe the orientation and displacement of a user's choice of base pairs. This is done by extracting two reference frames from a collection of interest and determining the 'end-to-end' base-pair step parameters using *emDNA_parser* (Figure 3(C)). Once collected, users can incorporate this virtual step into a naked DNA fragment using *emDNA_probind*. Here, a virtual step was generated using the first and last base-pair frames of the *n141* nucleosomal DNA ({–2.2°, –135.1°, –174.2°, 1.46 Å, –30.48 Å, –65.92 Å}, top of Figure 3(D) in gold), and inserted into a 195-bp minicircle, identical in length to the optimized loop found in Figure 3(B) (*circ195*, produced in Supplemental Protocol S1). In addition, users can alter the step parameters in any desired fashion, e.g., changing the step rise parameter (Figure 3 (D) bottom). Here are two loops with rise values changed to −150 Å and 250 Å, leading to increasingly stressed configurations of higher bending and twist energy (Figure 3(D) bottom right and Supplemental Table S1).

Discussion

The emDNA software offers researchers a new avenue in DNA functionality studies. Users can control chain length, sequence composition, placement of fragment ends, local structural deformations, and binding interactions over the course of the energy minimization of polymeric structures. These capabilities offer useful insights into the interplay between local regions of interest and the configuration of the DNA as a whole, such as the manner in which designed ligands may modulate looping propensities and associated genetic activity.[18] Structures such as those modeled in Figure 3 provide a jumping-off point for further investigation of the unwrapping, or breathing, of nucleosomal DNA away from the histone protein core.[17] Protein-bound DNA can also be deformed, such as by replacing one model of a nucleosome with another[17] or by opening and rotating binding sites found on proteins that mediate DNA looping.[36] The global DNA configuration can be altered by choice of intrinsic properties at the base-pair step level (see Figure 2 and 18,37). Simulated chains may take up multiple ligands and/or small molecules.[16,18] A series of related structures can be used in approximate treatments of large-scale molecular motions and individual configurations can serve as the starting state in detailed atomic-level studies.[22]

The command-line environment offered with this initial release of emDNA allows users direct interaction with the software for large, batch-scale jobs and for series-based optimizations, such as length-dependent looping profiles[18,36] or nucleosomal DNA breathing.[17] This degree of customizability makes emDNA a versatile modeling toolkit for both local use and high-performance computations. For example, users can develop DNA elastic models of their own design with the *emDNA_ff_packager* tool, such as from ensembles of structures in online repositories[6,31] or collected in detailed, large-scale molecular simulations.[38] Users can also control the degree of supercoiling within constrained systems through the choice of intrinsic parameters, notably the base-pair step twist.[18]

While emDNA is designed to be user friendly, there are limitations to consider. The optimization and protein-decoration ramping tools can be computationally time consuming, depending on DNA fragment size and number of ramped models. Depending on available computational resources, optimization of a 100-bp configuration may take less than two minutes while that of a 1000-bp configuration may take days. This is a particular concern when using the *emDNA_probind tool* as the number of iterations is akin to the number of optimizations to run.

There may be some cases where an optimized configuration will self-intersect and change the linking number, i.e., the integer value that describes the entanglement of the complementary strands of a closed DNA structure.[32,33] Application of electrostatic control during the optimization process with the *--dh–electrostatics* flag prevents inter-strand collisions but lengthens the computation time.

The next steps in emDNA software development will address broader functionality and enhanced usability. This includes an expandable base-pair step option, allowing users to work in the context of the 4^2 possible dimer steps or the 4^4 possible tetramer steps. Atomic-level simulations[39–41] and our preliminary studies of high-resolution structural data show that nucleotide context, i.e., the immediate neighbors of a dimer step, perturbs average step-level properties and modulates global structure of representative DNA chains. In addition, application of forces on the 3′-ends of DNA fragments will expand emDNA uses to simulations of single-molecule biophysical experiments.[42,43] Other planned enhancements include options for building initial fragments, customizable log files, and collision checking during optimization.

In summary, the emDNA software brings advanced DNA modeling technology to students and researchers of any skill level. The capability to manipulate and visualize higher-order conformations of DNA with methods previously available only to experts allows general users to develop and investigate models of their own design. The deeper understanding of mesoscale organization gained with these new tools may lead to new avenues of research, such as ligand design that improves the likelihood of stable loop formation and subsequent control of genetic processes.

Code availability

emDNA is free, open-source software built from C ++ programming language and requires CMake version 3.18.0 or later for compilation and installation. The emDNA code supports macOS (10.13 or later), Windows (8.1 or later), and Ubuntu (20.04 LTS or later) operating systems. The code for emDNA is available on GitHub (https://github.com/nicocvn/emDNA) with additional support, including installation instructions for all major operating systems and a detailed how-to guide, at https://nicocvn.github.io/emDNA/. All dependencies are accounted for in the current emDNA build and do not require additional installation by the user. The initial-configuration method described in the Supplementary Materials requires Python 3.X; the script is found in the emDNA repository.

CRediT authorship contribution statement

Robert T. Young: Conceptualization, Methodology, Validation, Data curation, Writing – original draft, Writing – review & editing. **Nicolas Clauvelin:** Conceptualization, Software. **Wilma K. Olson:** Conceptualization, Supervision, Resources, Validation, Funding acquisition, Project administration, Writing – original draft, Writing – review & editing.

DATA AVAILABILITY

Data will be made available on request.

Acknowledgement

This work was generously supported by the U.S. Public Health Service under research grant GM34809. The authors would like to thank Benjamin Cohen (Rutgers University) and Zoe Wefers (McGill University), whose undergraduate research contributed to the improvement of emDNA.

Conflict of Interest Statement

The authors declare no conflicts of interest in the publication of this paper.

Appendix A. Supplementary material

Supplementary data to this article can be found online at https://doi.org/10.1016/j.jmb.2022.167558.

Received 30 November 2021;
Accepted 18 March 2022;
Available online 24 March 2022

Keywords:
molecular modeling;
energy optimization;
protein-DNA interactions;
DNA loops;
DNA minicircles

References

1. Rice, P.A., Yang, S., Mizuuchi, K., Nash, H.A., (1996). Crystal structure of an IHF-DNA complex: a protein-induced DNA U-turn. *Cell* **87**, 1295–1306. https://doi.org/10.1016/S0092-8674(00)81824-3.
2. Davey, C.A., Sargent, D.F., Luger, K., Maeder, A.W., Richmond, T.J., (2002). Solvent mediated interactions in the structure of the nucleosome core particle at 1.9 Å resolution. *J. Mol. Biol.* **319**, 1097–1113. https://doi.org/10.1016/S0022-2836(02)00386-8.
3. Marti-Renom, M.A., Mirny, L.A., (2011). Bridging the resolution gap in structural modeling of 3D genome organization. *PLoS Comput. Biol.* **7**, e1002125. https://doi.org/10.1371/journal.pcbi.1002125.
4. Todolli, S., Perez, P.J., Clauvelin, N., Olson, W.K., (2017). Contributions of sequence to the higher-order structures of DNA. *Biophys. J.* **112**, 416–426. https://doi.org/10.1016/j.bpj.2016.11.017.
5. Berman, H.M., Westbrook, J., Feng, Z., Gilliland, G., Bhat, T.N., Weissig, H., Shindyalov, I.N., Bourne, P.E., (2000). The Protein Data Bank. *Nucleic Acids Res.* **28**, 235–242. https://doi.org/10.1093/nar/28.1.235.

6. Olson, W.K., Gorin, A., Lu, X.J., Hock, L.M., Zhurkin, V.B., (1998). DNA sequence-dependent deformability deduced from protein-DNA crystal complexes. *Proc. Natl. Acad. Sci., USA* **95**, 11163–11168. https://doi.org/10.1073/pnas.95.19.11163.
7. Svozil, D., Kalina, J., Omelka, M., Schneider, B., (2008). DNA conformations and their sequence preferences. *Nucleic Acids Res.* **36**, 3690–3706. https://doi.org/10.1093/nar/gkn260.
8. Rohs, R., West, S.M., Sosinsky, A., Liu, P., Mann, R.S., Honig, B., (2009). The role of DNA shape in protein–DNA recognition. *Nature* **461**, 1248–1253. https://doi.org/10.1038/nature08473.
9. Swigon, D., Coleman, B.D., Olson, W.K., (2006). Modeling the Lac repressor-operator assembly: the influence of DNA looping on Lac repressor conformation. *Proc. Natl. Acad. Sci., USA* **103**, 9879–9884. https://doi.org/10.1073/pnas.0603557103.
10. Balaeff, A., Mahadevan, L., Schulten, K., (2006). Modeling DNA loops using the theory of elasticity. *Phys. Rev. E* **73**, 031919. https://doi.org/10.1103/PhysRevE.73.031919.
11. Zhang, Y., McEwen, A.E., Crothers, D.M., Levene, S.D., (2006). Statistical-mechanical theory of DNA looping. *Biophys. J.* **90**, 1903–1912. https://doi.org/10.1529/biophysj.105.070490.
12. Harris, S.A., Laughton, C.A., Liverpool, T.B., (2007). Mapping the phase diagram of the writhe of DNA nanocircles using atomistic molecular dynamics simulations. *Nucleic Acids Res.* **36**, 21–29. https://doi.org/10.1093/nar/gkm891.
13. Czapla, L., Swigon, D., Olson, W.K., (2008). Effects of the nucleoid protein HU on the structure, flexibility, and ring-closure properties of DNA deduced from Monte Carlo simulations. *J. Mol. Biol.* **382**, 353–370. https://doi.org/10.1016/j.jmb.2008.05.088.
14. Wei, J., Czapla, L., Grosner, M.A., Swigon, D., Olson, W. K., (2014). DNA topology confers sequence specificity to nonspecific architectural proteins. *Proc. Natl. Acad. Sci., USA* **111**, 16742–16747. https://doi.org/10.1073/pnas.1405016111.
15. Biton, Y.Y., (2018). Effects of protein-induced local bending and sequence dependence on the configurations of supercoiled DNA minicircles. *J. Chem. Theory Comput.* **14**, 2063–2075. https://doi.org/10.1021/acs.jctc.7b01090.
16. Clauvelin, N., Olson, W.K., (2021). Synergy between protein positioning and DNA elasticity: energy minimization of protein-decorated DNA minicircles. *J. Phys. Chem. B* **125**, 2277–2287. https://doi.org/10.1021/acs.jpcb.0c11612.
17. Todolli, S., Young, R.T., Watkins, A.S., Bu Sha, A., Yager, J., Olson, W.K., (2021). Surprising twists in nucleosomal DNA with implication for higher-order folding. *J. Mol. Biol.* **433**, 167121. https://doi.org/10.1016/j.jmb.2021.167121.
18. Tse, D.H., Becker, N.A., Young, R.T., Olson, W.K., Peters, J.P., Schwab, T.L., Clark, K.J., Maher, L.J., (2021). Designed architectural proteins that tune DNA looping in bacteria. *Nucleic Acids Res.* **49**, 10382–10396. https://doi.org/10.1093/nar/gkab759.
19. Macke, T.J., Case, D.A., (1997). Modeling unusual nucleic acid structures. In: Leontis, N.B., SantaLucia, J. (Eds.), *ACS Symposium Series*. American Chemical Society, Washington, D.C., pp. 379–393.
20. Lu, X.-J., Olson, W.K., (2008). 3DNA: a versatile, integrated software system for the analysis, rebuilding and visualization of three-dimensional nucleic-acid structures. *Nature Protoc.* **3**, 1213–1227. https://doi.org/10.1038/nprot.2008.104.
21. Li, S., Olson, W.K., Lu, X.-J., (2019). Web 3DNA 2.0 for the analysis, visualization, and modeling of 3D nucleic acid structures. *Nucleic Acids Res.* **47**, W26–W34. https://doi.org/10.1093/nar/gkz394.
22. Machado, M.R., Pantano, S., (2015). Exploring LacI-DNA dynamics by multiscale simulations using the SIRAH force field. *J. Chem. Theory Comput.* **11**, 5012–5023. https://doi.org/10.1021/acs.jctc.5b00575.
23. Zechiedrich, E.L., Khodursky, A.B., Cozzarelli, N.R., (1997). Topoisomerase IV, not gyrase, decatenates products of site-specific recombination in Escherichia coli. *Genes Dev.* **11**, 2580–2592. https://doi.org/10.1101/gad.11.19.2580.
24. Fogg, J.M., Kolmakova, N., Rees, I., Magonov, S., Hansma, H., Perona, J.J., Zechiedrich, E.L., (2006). Exploring writhe in supercoiled minicircle DNA. *J. Phys.: Condens. Matter* **18**, S145–S159. https://doi.org/10.1088/0953-8984/18/14/S01.
25. Irobalieva, R.N., Fogg, J.M., Catanese, D.J., Sutthibutpong, T., Chen, M., Barker, A.K., Ludtke, S.J., Harris, S.A., et al., (2015). Structural diversity of supercoiled DNA. *Nature Commun.* **6**, 8440. https://doi.org/10.1038/ncomms9440.
26. Fogg, J.M., Judge, A.K., Stricker, E., Chan, H.L., Zechiedrich, L., (2021). Supercoiling and looping promote DNA base accessibility and coordination among distant sites. *Nature Commun.* **12**, 5683. https://doi.org/10.1038/s41467-021-25936-2.
27. Dickerson, R.E., (1989). Definitions and nomenclature of nucleic acid structure components. *Nucleic Acids Res.* **17**, 1797–1803. https://doi.org/10.1093/nar/17.5.1797.
28. Koo, H.-S., Wu, H.-M., Crothers, D.M., (1986). DNA bending at adenine · thymine tracts. *Nature* **320**, 501–506. https://doi.org/10.1038/320501a0.
29. Czapla, L., Swigon, D., Olson, W.K., (2006). Sequence-dependent effects in the cyclization of short DNA. *J. Chem. Theory Comput.* **2**, 685–695. https://doi.org/10.1021/ct060025+.
30. Kabsch, W., Sander, C., Trifonov, E.N., (1982). The ten helical twist angles of B-DNA. *Nucleic Acids Res.* **10**, 1097–1104. https://doi.org/10.1093/nar/10.3.1097.
31. Zhurkin, V.B., Tolstorukov, M.Y., Xu, F., Colasanti, A.V., Olson, W.K., (2005). Sequence-dependent variability of B-DNA: an update on bending and curvature. In: Ohyama, T. (Ed.), *DNA Conformation and Transcription*. Landes Bioscience/Eurekah.com, Georgetown, TX, pp. 18–34.
32. Călugăreanu, G., (1961). Sur les classes d'isotopie des noeuds tridimensionels et leurs invariants. *Czechoslovak Math. J.* **11**, 588–625.
33. White, J.H., (1969). Self-linking and the Gauss integral in higher dimensions. *Am. J. Math.* **91**, 693. https://doi.org/10.2307/2373348.
34. Zechiedrich, L., Fogg, J.M., (2019). Biophysics meets gene therapy: how exploring supercoiling-dependent structural changes in DNA led to the development of minivector DNA. *Technol. Innov.* **20**, 427–439. https://doi.org/10.21300/20.4.2019.427.
35. Grindley, N.D.F., Whiteson, K.L., Rice, P.A., (2006). Mechanisms of site-specific recombination. *Annu. Rev. Biochem.* **75**, 567–605. https://doi.org/10.1146/annurev.biochem.73.011303.073908.

36. Perez, P., Clauvelin, N., Grosner, M., Colasanti, A., Olson, W., (2014). What controls DNA looping? *Int. J. Mol. Sci.* **15**, 15090–15108. https://doi.org/10.3390/ijms150915090.
37. Perez, P.J., Olson, W.K., (2016). Insights into genome architecture deduced from the properties of short Lac repressor-mediated DNA loops. *Biophys. Rev.* **8**, 135–144. https://doi.org/10.1007/s12551-016-0209-7.
38. Lankaš, F., Šponer, J., Langowski, J., Cheatham, T.E., (2003). DNA basepair step deformability inferred from molecular dynamics simulations. *Biophys. J.* **85**, 2872–2883. https://doi.org/10.1016/S0006-3495(03)74710-9.
39. Beveridge, D.L., Barreiro, G., Suzie Byun, K., Case, D.A., Cheatham, T.E., Dixit, S.B., Giudice, E., Lankas, F., et al., (2004). Molecular dynamics simulations of the 136 unique tetranucleotide sequences of DNA oligonucleotides. I. Research design and results on d(CpG) steps. *Biophys. J.* **87**, 3799–3813. https://doi.org/10.1529/biophysj.104.045252.
40. Dixit, S.B., Beveridge, D.L., Case, D.A., Cheatham, T.E., Giudice, E., Lankas, F., Lavery, R., Maddocks, J.H., et al., (2005). Molecular dynamics simulations of the 136 unique tetranucleotide sequences of DNA oligonucleotides. II: Sequence context effects on the dynamical structures of the 10 unique dinucleotide steps. *Biophys. J.* **89**, 3721–3740. https://doi.org/10.1529/biophysj.105.067397.
41. Pasi, M., Maddocks, J.H., Beveridge, D., Bishop, T.C., Case, D.A., Cheatham, T., Dans, P.D., Jayaram, B., et al., (2014). μABC: a systematic microsecond molecular dynamics study of tetranucleotide sequence effects in B-DNA. *Nucleic Acids Res.* **42**, 12272–12283. https://doi.org/10.1093/nar/gku855.
42. Clauvelin, N., Audoly, B., Neukirch, S., (2009). Elasticity and electrostatics of plectonemic DNA. *Biophys. J.* **96**, 3716–3723. https://doi.org/10.1016/j.bpj.2009.02.032.
43. Norouzi, D., Zhurkin, V.B., (2018). Dynamics of chromatin fibers: comparison of Monte Carlo simulations with force spectroscopy. *Biophys. J.* **115**, 1644–1655. https://doi.org/10.1016/j.bpj.2018.06.032.
44. The PyMOL molecular graphics system, version 2.4, (2020).
