# Supplementary material for: PDBx/mmCIF Ecosystem: Foundational Semantic Tools for Structural Biology
Source: J Mol Biol. Author manuscript; Available in PMC 2023 Jun 26. (PMC10292674; doi:10.1016/j.jmb.2022.167599)
Supplement: Article [file NIHMS1907597-supplement-Article.zip › The-TissueNet-v-3-Database--Protein-protein-Interaction_2022_Journal-of-Mole.pdf]

Web Server

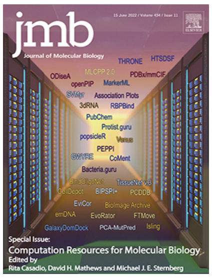

The TissueNet v.3 Database: Protein-protein Interactions in Adult and Embryonic Human Tissue contexts

Maya Ziv [1], Gil Gruber [1], Moran Sharon [1], Ekaterina Vinogradov [1] and Esti Yeger-Lotem [1,2*]

1 - ***Department of Clinical Biochemistry and Pharmacology,*** *Faculty of Health Sciences, Ben-Gurion University of the Negev, Beer-Sheva, Israel*

2 - ***The National Institute for Biotechnology in the Negev,*** *Ben-Gurion University of the Negev, Beer-Sheva, Israel*

Correspondence to Esti Yeger-Lotem: Department of Clinical Biochemistry and Pharmacology, Faculty of Health Sciences, Ben-Gurion University of the Negev, Beer-Sheva, Israel. *estiyl@bgu.ac.il (E. Yeger-Lotem)*
https://doi.org/10.1016/j.jmb.2022.167532
Edited by Michael Sternberg

Abstract

Tissue contexts are extremely valuable when studying protein functions and their associated phenotypes. Recently, the study of proteins in tissue contexts was greatly facilitated by the availability of thousands of tissue transcriptomes. To provide access to these data we developed the TissueNet integrative database that displays protein-protein interactions (PPIs) in tissue contexts. Through TissueNet, users can create tissue-sensitive network views of the PPI landscape of query proteins. Unlike other tools, TissueNet output networks highlight tissue-specific and broadly expressed proteins, as well as over- and under-expressed proteins per tissue. The TissueNet v.3 upgrade has a much larger dataset of proteins and PPIs, and represents 125 adult tissues and seven embryonic tissues. Thus, TissueNet provides an extensive, quantitative, and user-friendly interface to study the roles of human proteins in adulthood and embryonic stages. TissueNet v3 is freely available at https://netbio.bgu.ac.il/tissuenet3.

© 2022 Elsevier Ltd. All rights reserved.

Introduction

The context in which proteins function holds valuable phenotypic and clinical information.[1–3] Context is especially important when studying the roles of human proteins. Composed of hundreds of tissues and cell types, the human body portrays vast phenotypic diversity that is both derived from and leads to distinct, sometimes unique, gene expression profiles.[4] These in turn lead to context-specific PPIs that determine tissue characteristics and functionalities.[5–7] However, most of the experimentally-detected PPIs between human proteins lack tissue contexts, calling for integrative approaches that associate PPIs with tissue contexts to explore and improve our understanding of the large variety of biological components and their functionalities.[8–9]

Acknowledging the importance of tissue contexts, many efforts were invested in mapping tissue transcriptomes via high-throughput RNA-sequencing (RNA-seq). Major resources include Human Protein Atlas (HPA)[10] and Genotype-Tissue Expression consortium (GTEx),[4] which led to unprecedented amounts of accessible tissue transcriptomes. The availability of hundreds and thousands of tissue transcriptomes allowed for quantitative description of tissue differences, focusing on regulation,[7,11–13] phenotypes[14–16] and biological processes.[17–18] Importantly, tissue transcriptomes were harnessed for associating PPIs with tissue contexts.[19] For example, PPIs involving genes that were lowly expressed in a given tissue were not associated with that tissue.[9,20]

We previously published the TissueNet database, which was one of the first tools to associate PPIs

0022-2836/© 2022 Elsevier Ltd. All rights reserved.

with tissue contexts.[21–22] TissueNet users select a transcriptomic resource, a query protein, and a tissue, and obtain a graphical network view of the PPIs of the query protein in the selected tissue (Figure 1). TissueNet is not alone, as additional web tools have addressed the need for inferring tissue-sensitive PPIs and PPI networks (interactomes), including HumanBase,[5] SPECTRA,[23] HIPPIE V.2,[24] IID,[25] and TissueNexus.[16] Differences between the web tools include the type of relationships that they portray (e.g., predicted PPIs or functional interactions), their transcriptomic resources and supported tissues, and the visualization of the output networks (Table 1). Notably, TissueNet remains unique in calculating and portraying tissue-specific versus broadly expressed proteins, as well as the over- and under-expressed proteins per tissue (Table 1) (Figure 1(B)).

The tissue contexts supported by most tools were inferred from adult tissue transcriptomes, however, tissue transcriptomes change with age.[26] For example, RNA-seq analysis of seven organs from early organogenesis to adulthood showed that genes expressed earlier in development were more broadly expressed both spatially and temporally.[27] These differences lead to various age-related phenotypes, such as early- versus late-onset diseases.[28]

Motivated by the increase in the coverage of tissue transcriptomes and experimentally-detected PPIs,[9] we constructed TissueNet v.3. This new version of TissueNet offers a two-fold increase in the number of PPIs and 30-fold increase in the number of tissue transcriptomic profiles.[4] Importantly, TissueNet v.3 includes for the first time transcriptomes of seven embryonic tissues, therefore providing a unique functional view into tissue development (Figure 1). TissueNet is freely available at https://netbio.bgu.ac.il/tissuenet3/.

Results and Discussion

Below we describe the construction of TissueNet v.3 and highlight its new features.

To construct TissueNet, we gathered data of experimentally-detected PPIs from the up-to-date BioGRID[29] and MIntAct[30] databases. We included only PPIs that were detected by at least one well-established method for detecting physical interactions between proteins (e.g., proximity-based methods were disregarded). The resulting PPI dataset, henceforth denoted the generic human interactome, consisted of 482,693 PPIs between 19,907 human proteins.

To associate PPIs with tissue contexts we gathered transcriptomic and proteomic profiles of adult and embryonic human tissues. Transcriptomic profiles of adult tissues were obtained from the GTEx[4] and HPA[10] resources. Notably, the number of tissue samples in GTEx profiles increased considerably relative to TissueNet v.2 and included measurements for 19,225 proteins in 52 tissues that we collapsed into 37 main tissues (Methods). HPA transcriptomic data included measurements for 20,090 protein-coding genes in 41 tissues. HPA proteomic data included measurements for 14,578 proteins in 47 adult tissues. Transcriptomic profiles of seven embryonic tissues were obtained from ref.[27] These profiles were sampled from embryos aged 4–19 weeks post conception, and included 13 time points. Per tissue, the expression level of each protein was set to its median expression level across tissue samples (Methods). Lastly, a PPI was associated with a specific tissue if the expression level in that tissue of each interacting protein exceeded a user-defined threshold.

Users can query TissueNet by expression dataset, protein, and tissue. For each query, TissueNet integrates PPI and expression data and presents the PPIs of the query protein in the selected tissue according to the specific dataset. TissueNet uses a graphical interface to portray PPIs as a network. Unlike other tools, the network is colored to highlight the tissue-specificity of each protein: Broadly-expressed proteins (expressed above a user-defined threshold in $\geq$ 80% of the tissues) in blue; tissue-specific proteins (expressed above a user-defined threshold in $\leq$ 20% of the tissues) in orange; and other proteins in grey. TissueNet additionally supports a quantitative view that portrays the preferential expression of proteins in the selected tissue relative to other tissues,[11] highlighting over-expressed proteins in red and under-expressed proteins in blue. TissueNet users can obtain information about the presented proteins (expression Gene Ontology annotations, etc.) and about the PPI detection methods. Additionally, users can dynamically explore the PPI network of the query protein by an interactive menu that allows moving between tissues, expression thresholds and network views.

The integration scheme used by TissueNet and other tools assumes that expression is a necessary condition for a PPI to occur, however PPI occurrence is not guaranteed. A recent analysis of protein complexes in seven mouse tissues found a significant yet low overlap between predicted and experimentally-detected complexes.[31] Nevertheless, protein complexes were best captured by prediction based on gene expression, and to a lesser extent protein expression and gene co-expression.

Compared to TissueNet v.2, the data in TissueNet v.3 expanded considerably (Table S1). In addition to a 2-fold increase in the number of experimentally-detected PPIs, the number of adult tissue samples increased over 30-fold, thereby enhancing its reliability. The other important advantage of TissueNet v.3 is the ability to explore PPIs in embryonic tissue contexts. To assess the differences between adult and embryonic

Table 1 Web tools for analyzing human tissue PPI interactomes.

	Interaction type	Number of experimentally-detected PPIs	Number of adult tissues	Number of embryonic tissues	Coloring by tissue-specificity	Coloring by tissue preferential expression
**TissueNet v.3**	PPI	482,693	125	**7**	**V**	**V**
**TissueNet v.2**	PPI	243,706	116	0	**V**	**V**
**IID**	PPI	560,628	120	No tissue context[2]	X	X
**HIPPIE**	PPI	273,900	53	0	X	X
**SPECTRA**	PPI	175,841	107	0	X	V (only against user-selected tissues)
**HUMANBASE**	Functional[1]	NA	144	No tissue context[2]	X	X
**TissueNexus**	Functional	NA	49	No tissue context[2]	X	X

[1] Functional interactions include diverse relationships such as regulatory relationship, co-expression, and protein-protein interaction.
[2] A single embryonic state or embryonic cells lacking tissue contexts.

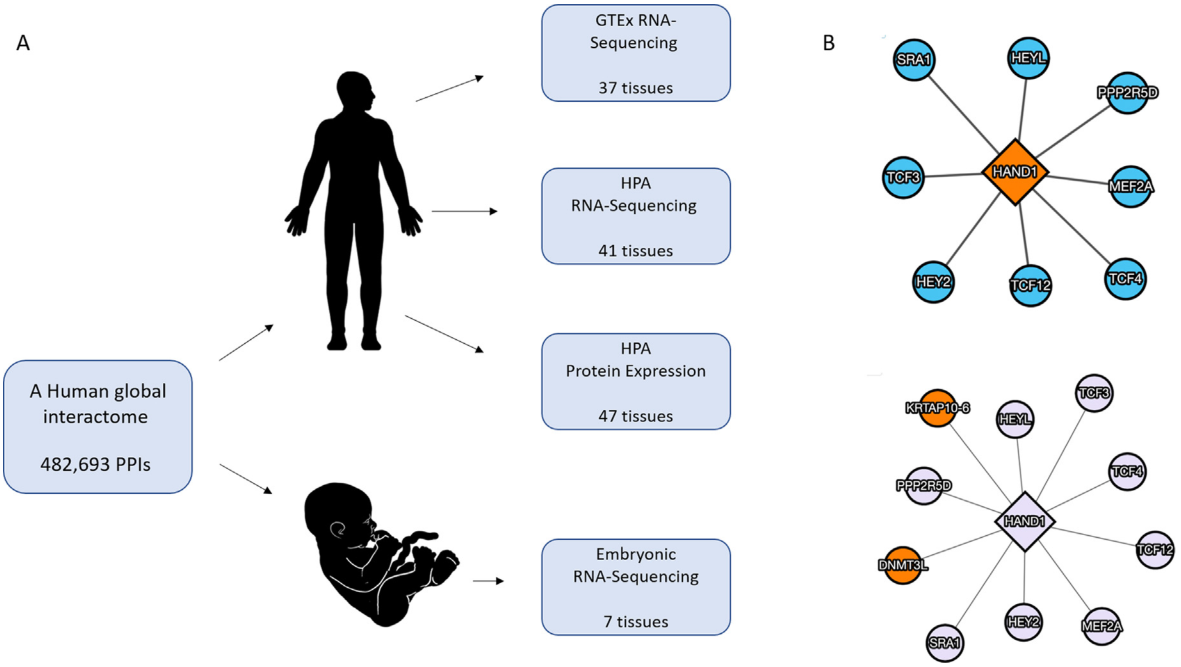

Figure 1. An overview of TissueNet. A. TissueNet includes 482,693 PPIs between 19,907 proteins. Proteins and PPIs are filtered according to the transcriptomic dataset that is selected by the user. Tissue profiling resources include adult and embryonic human tissue transcriptomes and adult tissue proteomes. B. The PPI network surrounding the query protein Hand1 in testis in adult (GTEx, top) and embryonic (bottom) tissues shows changes in the tissue-specificity of Hand1 and its partners. Node colors reflect the tissue-specificity of the correposnding protein: Orange = tissue-specific proteins; blue = broadly expressed proteins; grey = other.

interactomes we analyzed their tissue-specificity. With a conventional expression threshold of 5 TPM, according to both GTEx and HPA about 30% of the proteins were tissue-specific in adult tissues, compared to 10% in embryonic tissues (Figure 2(A)). In contrast, about 40% of the proteins were broadly-expressed in adult tissues, whereas in embryonic tissues they were about 70% (Figure 2(A)). Next, we asked how many PPIs involved tissue-specific proteins. In adult, this was about 50,000 PPIs, whereas in embryonic tissues only 17,000 PPIs (Figure 2(B)). The embryonic tissue with the largest number of tissue-specific proteins was liver. Cerebrum and testis had the smallest numbers of tissue-specific proteins and PPIs, potentially because both tissues have permissive expression. We repeated this analysis with an expression threshold of 10 TPM and observed similar trends (Figure 2). Thus, there are marked differences in the tissue-specificity of proteins and PPIs between adult and embryonic tissues, implying that age-related phenotypes should be studied in suitable contexts.

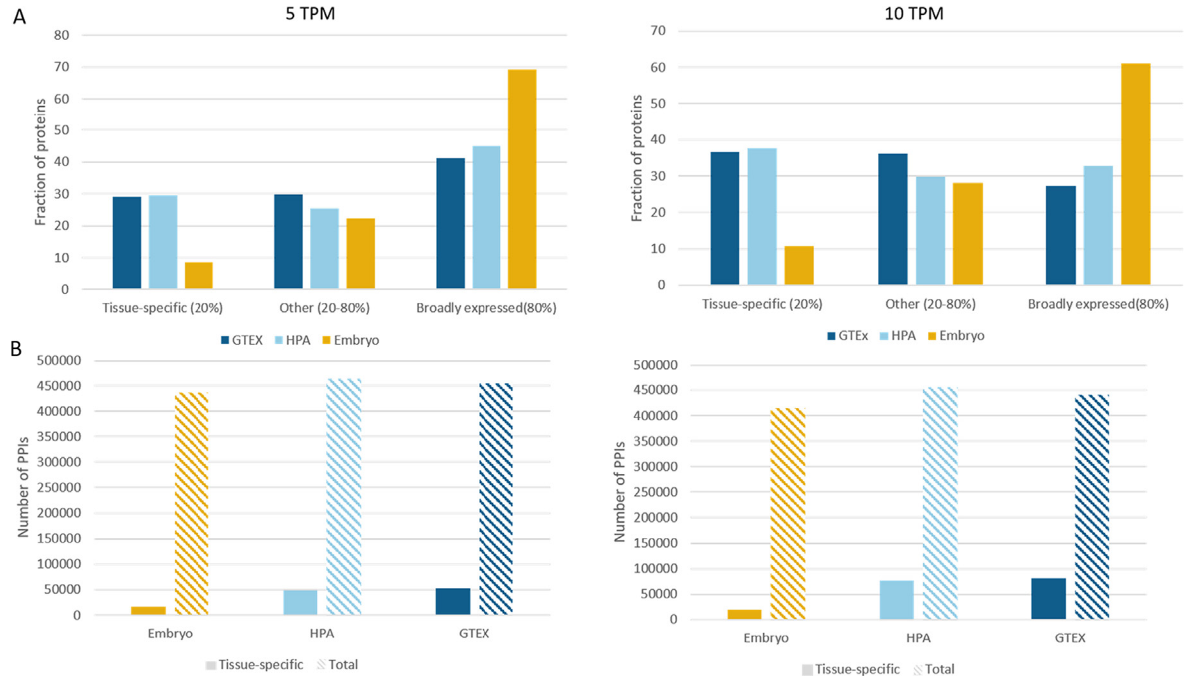

Figure 2. The tissue-specificity of proteins and PPIs in **adult and embryonic tissue contexts.** A. The numbers of proteins that were categorized as tissue-specific, broadly-expressed or other, per transcriptomic dataset. Embryonic tissues had the smallest fraction of tissue-specific proteins and the largest fraction of broadly expressed proteins. The numbers of proteins expressed at 5 TPM or higher in GTEx, HPA and embryonic tissues were 16,555, 18,083, 14,549, respectively. The numbers of proteins expressed at 10 TPM or higher in GTEx, HPA and embryonic tissues were 15,267, 16,949, 13,115, respectively. B. The numbers of PPIs involving tissue-specific proteins and the total numbers of PPIs per dataset. Whereas the total numbers of PPIs were similar across datasets, embryonic tissues had the smallest number of PPIs involving tissue-specific proteins.

In summary, TissueNet synergizes between large-scale data of PPIs, proteomes and transcriptomes of tens of human tissues, to create an extensive and easy-to-use database of PPIs in adult and embryonic tissue contexts.

Methods

PPI data. Human PPIs were downloaded from BioGrid[29] and MIntAct[30] and consolidated by using the MyProteinNet webserver.[32] The usage of MyProteinNet ensured that only PPIs that were detected by established experimental methods for detection of physical protein interactions were considered. This resulted in a generic PPI network. PPI data will be updated every six months.

Expression data sources. Expression profiles of adult tissues were obtained from GTEx[4] and HPA.[10] We downloaded data of GTEx v8 that reported the median TPM (transcript per million) counts per gene and tissue (file 'GTEx_Analysis_2017-06 05_v8_RNASeQCv1.1.9_gene_median_tpm.gct.gz). We united brain sub tissues according to Supplementary Table S2, such that the TPM value of each gene in a tissue was set to its median TPM across sub tissues. We removed cells and tissues with less than five samples. We downloaded data of HPA from ArrayExpress, accession number: E-MTAB-2836 and from HPA site (file 'rna_tissue_hpa.tsv.zip'). These data included protein TPM (pTPM) counts per gene and tissue. We united all brain sub tissues and all spinal cord sub tissues into brain and spinal cord tissues, respectively, such that the pTPM value of each gene in a tissue was set to its median pTPM across sub tissues. HPA proteomic data were obtained from ref.[33]

Expression profiles of embryonic tissues were obtained from ref.[27] TPM counts of genes in seven developing human organs were obtained from ArrayExpress, accession number: E-MTAB-6814. We considered only time points measured in post conception samples (weeks 4–19). TPM counts of each gene in each organ was set to its median TPM across time points.

Preferential expression analysis. The preferential expression of a gene in a tissue relative to all tissues was calculated by using a z-score like calculation, similar to ref[11] (Equation 1). To avoid division by 0 we added 1 to the denominator. Below, *t* denotes tissue, *T* denotes all tissues, *g* denotes gene, *e* denotes expression, *med* denotes median, *IQR* denotes interquartile range.

Equation 1:

$$\forall t \in T, \forall g \in G : pref_g^{(t)} = \frac{\left[med\left(e_g^{(t)}\right) - med\left(med\left(e_g^{\left(\widehat{\forall t \in T}\right)}\right)\right)\right]}{IQR\left(med\left(e_g^{\left(\widehat{\forall t \in T}\right)}\right)\right) + 1}$$

Associating PPIs with tissue contexts. A PPI is associated with a specific tissue if the expression level in that tissue of each of the interacting proteins exceeds a user-defined (or default) threshold. The threshold can be changed dynamically via an interactive menu.

Implementation. The TissueNet server was implemented in Python, using the Flask framework, with data stored on a MySQL database. The website client was programmed using the ReactJS framework and designed with Semantic-UI CSS. The network view is displayed by the Cytoscape.js plugin.[34] The website supports all major browsers. Recommended viewing resolution is 1440 × 900 and above.

Download. The TissueNet database is available for download under the permissive Creative Commons license. The download page offers the user the ability to download data separately for each data source.

CRediT authorship contribution statement

Maya Ziv: Data curation, Formal analysis, Writing – original draft, Writing – review & editing. **Gil Gruber:** Software. **Moran Sharon:** Writing – original draft. **Ekaterina Vinogradov:** Visualization. **Esti Yeger-Lotem:** Conceptualization, Supervision, Funding acquisition, Writing – original draft, Writing – review & editing.

Acknowledgements

This study was funded by the Israel Science Foundation [317/19 to E.Y.-L].

Declaration of Competing Interest

The authors declare that they have no known competing financial interests or personal relationships that could have appeared to influence the work reported in this paper.

Appendix A. Supplementary data

Supplementary data to this article can be found online at https://doi.org/10.1016/j.jmb.2022.167532.

Received 30 November 2021;
Accepted 3 March 2022;
Available online 12 March 2022

Keywords:
protein-protein interactions;
tissue-specificity;
data integration;
gene expression;
interaction networks;
tissue interactomes

References

1. Qin, Y., Huttlin, E.L., Winsnes, C.F., Gosztyla, M.L., Wacheul, L., Kelly, M.R., et al., (2021). A multi-scale map of cell structure fusing protein images and interactions. *Nature*.
2. Cummings, B.B., Marshall, J.L., Tukiainen, T., Lek, M., Donkervoort, S., Foley, A.R., et al., (2017). Improving genetic diagnosis in Mendelian disease with transcriptome sequencing. *Sci. Transl. Med.* **9**
3. Duffy, A., Verbanck, M., Dobbyn, A., Won, H.H., Rein, J.L., Forrest, I.S., et al., (2020). Tissue-specific genetic features inform prediction of drug side effects in clinical trials. *Sci. Adv.* **6**
4. GTEx Consortium, (2020). The GTEx Consortium atlas of genetic regulatory effects across human tissues. *Science* **369**, 1318–1330.
5. Greene, C.S., Krishnan, A., Wong, A.K., Ricciotti, E., Zelaya, R.A., Himmelstein, D.S., et al., (2015). Understanding multicellular function and disease with human tissue-specific networks. *Nat. Genet.* **47**, 569–576.
6. Malod-Dognin, N., Petschnigg, J., Windels, S.F.L., Povh, J., Hemingway, H., Ketteler, R., et al., (2019). Towards a data-integrated cell. *Nat. Commun.* **10**, 805.
7. Marbach, D., Lamparter, D., Quon, G., Kellis, M., Kutalik, Z., Bergmann, S., (2016). Tissue-specific regulatory circuits reveal variable modular perturbations across complex diseases. *Nat. Methods* **13**, 366–370.
8. Huttlin, E.L., Bruckner, R.J., Paulo, J.A., Cannon, J.R., Ting, L., Baltier, K., et al., (2017). Architecture of the human interactome defines protein communities and disease networks. *Nature* **545**, 505–509.
9. Luck, K., Kim, D.K., Lambourne, L., Spirohn, K., Begg, B. E., Bian, W., et al., (2020). A reference map of the human binary protein interactome. *Nature* **580**, 402–408.
10. Uhlen, M., Fagerberg, L., Hallstrom, B.M., Lindskog, C., Oksvold, P., Mardinoglu, A., et al., (2015). Proteomics. Tissue-based map of the human proteome. *Science* **347**, 1260419.
11. Sonawane, A.R., Platig, J., Fagny, M., Chen, C.Y., Paulson, J.N., Lopes-Ramos, C.M., et al., (2017). Understanding tissue-specific gene regulation. *Cell Rep.* **21**, 1077–1088.
12. Saha, A., Kim, Y., Gewirtz, A.D.H., Jo, B., Gao, C., McDowell, I.C., et al., (2017). Co-expression networks reveal the tissue-specific regulation of transcription and splicing. *Genome Res.* **27**, 1843–1858.
13. Ben Guebila, M., Lopes-Ramos, C.M., Weighill, D., Sonawane, A.R., Burkholz, R., Shamsaei, B., et al.,

(2022). GRAND: a database of gene regulatory network models across human conditions. *Nucleic Acids Res.* **50**, D610–D621.

14. Gamazon, E.R., Segre, A.V., van de Bunt, M., Wen, X., Xi, H.S., Hormozdiari, F., et al., (2018). Using an atlas of gene regulation across 44 human tissues to inform complex disease- and trait-associated variation. *Nat. Genet.* **50**, 956–967.
15. A.N. Barbeira, R. Bonazzola, E.R. Gamazon, Y. Liang, Y. Park, S. Kim-Hellmuth, et al., Widespread dose-dependent effects of RNA expression and splicing on complex diseases and traits. 2019:814350.
16. Lin, C.X., Li, H.D., Deng, C., Guan, Y., Wang, J., (2022). TissueNexus: a database of human tissue functional gene networks built with a large compendium of curated RNA-seq data. *Nucleic Acids Res.* **50**, D710–D718.
17. Bryan, J.M., Fufa, T.D., Bharti, K., Brooks, B.P., Hufnagel, R.B., McGaughey, D.M., (2018). Identifying core biological processes distinguishing human eye tissues with precise systems-level gene expression analyses and weighted correlation networks. *Hum. Mol. Genet.* **27**, 3325–3339.

[18]. Sharon, M., Vinogradov, E., Argov, C.M., Lazarescu, O., Zoabi, Y., Hekselman, I., et al., (2022). The differential activity of biological processes in tissues and cell subsets can illuminate disease-related processes and cell type identities. *Bioinformatics*. https://doi.org/10.1093/bioinformatics/btab883.

19. Hekselman, I., Yeger-Lotem, E., (2020). Mechanisms of tissue and cell-type specificity in heritable traits and diseases. *Nat. Rev. Genet.* **21**, 137–150.
20. Magger, O., Waldman, Y.Y., Ruppin, E., Sharan, R., (2012). Enhancing the Prioritization of Disease-Causing Genes through Tissue Specific Protein Interaction Networks. *PLoS Comput. Biol.* **8**, e1002690
21. Barshir, R., Basha, O., Eluk, A., Smoly, I.Y., Lan, A., Yeger-Lotem, E., (2013). The TissueNet database of human tissue protein-protein interactions. *Nucleic Acids Res.* **41**, D841–D844.
22. Basha, O., Barshir, R., Sharon, M., Lerman, E., Kirson, B. F., Hekselman, I., et al., (2017). The TissueNet vol 2 database: A quantitative view of protein-protein interactions across human tissues. *Nucleic Acids Res.* **45**, D427–D431.
23. Micale, G., Ferro, A., Pulvirenti, A., Giugno, R., (2015). SPECTRA: An Integrated Knowledge Base for Comparing Tissue and Tumor-Specific PPI Networks in Human. *Front. Bioeng. Biotechnol.* **3**, 58.
24. Alanis-Lobato, G., Andrade-Navarro, M.A., Schaefer, M.H., (2017). HIPPIE v2.0: enhancing meaningfulness and reliability of protein-protein interaction networks. *Nucleic Acids Res.* **45**, D408–D414.
25. Kotlyar, M., Pastrello, C., Ahmed, Z., Chee, J., Varyova, Z., Jurisica, I., (2022). IID 2021: towards context-specific protein interaction analyses by increased coverage, enhanced annotation and enrichment analysis. *Nucleic Acids Res.* **50**, D640–D647.
26. Ham, S., Lee, S.V., (2020). Advances in transcriptome analysis of human brain aging. *Exp. Mol. Med.* **52**, 1787–1797.
27. Cardoso-Moreira, M., Halbert, J., Valloton, D., Velten, B., Chen, C., Shao, Y., et al., (2019). Gene expression across mammalian organ development. *Nature* **571**, 505–509.
28. The portal for rare diseases and orphan drugs, 2021.
29. Stark, C., Breitkreutz, B.J., Reguly, T., Boucher, L., Breitkreutz, A., Tyers, M., (2006). BioGRID: a general repository for interaction datasets. *Nucleic Acids Res.* **34**, D535–D539.
30. Orchard, S., Ammari, M., Aranda, B., Breuza, L., Briganti, L., Broackes-Carter, F., et al., (2014). The MIntAct project–IntAct as a common curation platform for 11 molecular interaction databases. *Nucleic Acids Res.* **42**, D358–D363.
31. Skinnider, M.A., Scott, N.E., Prudova, A., Kerr, C.H., Stoynov, N., Stacey, R.G., et al., (2021). An atlas of protein-protein interactions across mouse tissues. *Cell* **184**, (4073–89) e17
32. Basha, O., Flom, D., Barshir, R., Smoly, I., Tirman, S., Yeger-Lotem, E., (2015). MyProteinNet: build up-to-date protein interaction networks for organisms, tissues and user-defined contexts. *Nucleic Acids Res.* **43**, W258–W263.
33. Wilhelm, M., Schlegl, J., Hahne, H., Gholami, A.M., Lieberenz, M., Savitski, M.M., et al., (2014). Mass-spectrometry-based draft of the human proteome. *Nature* **509**, 582–587.
34. Otasek, D., Morris, J.H., Boucas, J., Pico, A.R., Demchak, B., (2019). Cytoscape Automation: empowering workflow-based network analysis. *Genome Biol.* **20**, 185.
