# Supplementary material for: PDBx/mmCIF Ecosystem: Foundational Semantic Tools for Structural Biology
Source: J Mol Biol. Author manuscript; Available in PMC 2023 Jun 26. (PMC10292674; doi:10.1016/j.jmb.2022.167599)
Supplement: Article [file NIHMS1907597-supplement-Article.zip › Protist-guru--A-Comparative-Transcriptomics-Datab_2022_Journal-of-Molecular-.pdf]

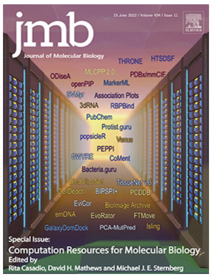

Protist.guru: A Comparative Transcriptomics Database for Protists

Erielle Marie Fajardo Villanueva[1], Peng Ken Lim[1], Jolyn Jia Jia Lim[1], Shan Chun Lim[1], Pei Yi Lau[1], Kenny Ting Sween Koh[1], Emmanuel Tan[1], Ryanjit Singh Kairon[1], Wei An See[1], Jian Xiang Liao[1], Ker Min Hee[1], Varsheni Vijay[1], Ishani Maitra[1], Chong Jun Boon[1], Kevin Fo[1], Yee Tat Wang[1], Ryan Jaya[1], Li Anne Hew[1], Yong Yee Lim[1], Wei Quan Lee[1], Zhi Qi Lee[1], Herman Foo[1], Adriana Lopes dos Santos[2] and Marek Mutwil[1]*

*1 - **School of Biological Sciences,** Nanyang Technological University, 60 Nanyang Drive, Singapore 637551, Singapore*
*2 - **Asian School of the Environment,** Nanyang Technological University, 60 Nanyang Drive, Singapore 637551, Singapore*

Correspondence to Marek Mutwil:Corresponding author at: School of Biological Sciences, Nanyang Technological University, 60 Nanyang Drive, 637551 Singapore, Singapore. *mutwil@ntu.edu.sg (M. Mutwil), @labmutwil (M. Mutwil)*
https://doi.org/10.1016/j.jmb.2022.167502
Edited by David Mathews

Abstract

Summary: During the last few decades, the study of microbial ecology has been enabled by molecular and genomic data. DNA sequencing has revealed the surprising extent of microbial diversity and how microbial processes run global ecosystems. However, significant gaps in our understanding of the microbial world remain, and one example is that microbial eukaryotes, or protists, are still largely neglected. To address this gap, we used gene expression data from 17 protist species to create protist.guru: an online database equipped with tools for identifying co-expressed genes, gene families, and co-expression clusters enriched for specific biological functions. Here, we show how our database can be used to reveal genes involved in essential pathways, such as the synthesis of secondary carotenoids in *Haematococcus lacustris*. We expect protist.guru to serve as a valuable resource for protistologists, as well as a catalyst for discoveries and new insights into the biological processes of microbial eukaryotes.
Availability: The database and co-expression networks are freely available from http://protist.guru/. The expression matrices and sample annotations are found in the supplementary data.
© **2022 Elsevier Ltd. All rights reserved.**

Introduction

Microbial eukaryotes, or protists, are a phylogenetically broad collection of single-celled organisms. The term "protist" was employed in the 19th century by the artist and biologist Ernest Haeckel[8] to all eukaryotes that were not plants, animals, or fungi. Today, we know that although most of the described species of eukaryotes belong to the multicellular collection of animals (Metazoa), plants, and fungi, these lineages only represent a very small proportion of the eukaryotic diversity.[11,15] Together with bacteria and fungi, protists form the engine of every biogeochemical cycle central to all ecosystems on earth. Through their microbial processes, they drive the cycling of nutrients and the energy flow between all planet spheres (e.g., biosphere and atmosphere).

0022-2836/© 2022 Elsevier Ltd. All rights reserved.

Despite their impact on human health and on a planetary scale, the understanding of gene function in protists has lagged behind other microbial taxa. The reasons for this are numerous and range from technical challenges to a lack of readily cultivable strains. In addition, protists have much larger genomes and more complicated gene expression patterns when compared to bacteria. These challenges have resulted in minimal knowledge about the gene number, identity, and function within several protistan lineages.[3] Gene function can be predicted by using sequence similarity analyses, but these analyses fail for genes that do not show sequence similarity to characterized genes.[22] Consequently, methods based on gene expression have increasingly been used to predict gene function.[25] Co-expression analysis finds functionally related genes by identifying genes that exhibit similar expression profiles across different growth conditions and genotypes.[19] However, previous approaches have only studied a few organisms with limited computational analyses,[6] precluding us from understanding the biology of protists.

To improve our understanding of protists' gene functions and expressions, the protist.guru database was constructed based on 2,342 transcriptomes. Protist.guru is equipped with a plethora of tools, empowering users to analyze gene expression profiles and co-expression networks across 17 protist species and allowing for the study of novel genes essential for biological processes in protists.

Materials and methods

Download of genomic and transcriptomic data

The protist.guru database is using the CoNekT framework, an open-source platform that facilitates comparative genomic and transcriptome analysis[21]. For 15 protist species, RNA-seq data was sourced from the publicly available Sequence Read Archive (SRA)[13] from NCBI. IDs of Illumina-based sequencing runs and corresponding experimental metadata were used to stream fastq files and annotate runs, respectively. Gene expression data and experiment annotations for *Chlamydomonas reinhardtii* (605 RNA-seq experiments)[21] and *Cyanophora paradoxa* (79 experiments)[7] were downloaded from the cited studies.

For each species, coding sequence (CDS) files were downloaded from various sources (Table S1). The CDS files were subsequently used to generate Kallisto index files using Kallisto v0.46.0[1] with default parameters. By applying the LSTrAP-Cloud[24] pipeline, each experiment was streamed as a fastq file from the European Nucleotide Archive (ENA).[13] In total, 2,482 experiments were downloaded (Table S2). TPM (transcripts per million) expression values were extracted from the files generated from Kallisto's quant function with default parameters. To annotate the RNA-seq experiments, the aforementioned metadata from the run tables was used to include information such as culture medium, genotype and other experimental variables. Annotation data was also supplemented by existing publications that are associated with the runs.

Quality control of gene expression data

For quality control, RNA-sequencing experiments containing >1 million reads for the number of processed reads (NPR) (with the exception of *Cladocopium* sp. *clade C*, *Micromonas pusilla*, *Porphyridium purpureum*, and *Thalassiosira pseudonana*) were kept. Furthermore, percentage of pseudoaligned reads (PPR) threshold was set for each species. The NPR and PPR values were obtained from Kallisto index files. The PPR threshold was set by manual observation of scatter-plots which displayed graphs of NPR values on the x-axis against PPR values on the y-axis (Supplementary Figure 1). These thresholds were set to remove outlier samples that had an insufficient NPR or PPR value. The 1,658 samples which passed the thresholds were subsequently used to generate expression matrices for all 15 protists (Supplemental Data S1–S15), available from https://doi.org/10.6084/m9.figshare.17295365.v1.

Functional annotation of proteins

To predict gene function, protein IDs from pep files were obtained using the conversion feature onboard the CoNekT framework. For each protein, the Pfam domains and Gene Ontology (GO) terms were obtained via Interproscan-5.51–85.0.[12] Orthogroups were identified and inferred phylogenetic trees were obtained via the use of Orthofinder v2.3.12[5] and Diamond[2] with default settings.

Construction of protist.guru database

The database was constructed with the above data using the CoNekT framework admin panel. The coexpression networks were constructed using the Highest Reciprocal Rank metric.[17] Coexpression clusters for each species were generated via Heuristic Cluster Chiseling Algorithm (HCCA)[17] where cluster sizes were limited to 100 genes. The database runs on an Apache server with Windows OS.

Implementation

Our database has multiple tools to allow for different analyses of the genomic and transcriptomic data for 17 protists (Table 1). The data can be viewed with ease through pages such as species (www.protist.guru/species), genes (example of photosynthesis gene https://protists.sbs.ntu.edu.sg/sequence/view/47536), gene families (example of gene family involved in

Table 1 17 protist species featured on Protist.guru and their statistics. The number of RNA-seq experiments indicates the samples that passed quality control and all processed samples (in parentheses).

Protist species	Class	Number of genes	RNA-seq experiments
Chlamydomonas reinhardtii	Chlorophyta	17,741	605
Chlorella sorokiniana	Chlorophyta	10,384	33 (102)
Chlorella vulgaris	Chlorophyta	27,303	34 (52)
Chromochloris zofingiensis	Chlorophyta	15,369	179 (180)
Cladocopium sp. *clade C*	Dinoflagellata	35,912	52 (106)
Cyanophora paradoxa	Glaucophyta	24,702	79
Dunaliella sp.	Chlorophyta	16,697	88 (114)
Emiliania huxleyi	Haptophyta	38,554	288 (369)
Haematococcus lacustris	Chlorophyta	28,279	56 (57)
Micromonas commoda	Chlorophyta	10,041	65 (76)
Micromonas pusilla	Chlorophyta	10,242	30 (232)
Ostreococcus tauri	Chlorophyta	7668	113 (130)
Phaeodactylum tricornutum	Ochrophyta	12,178	347 (463)
Porphyridium purpureum	Rhodophyta	9898	55 (64)
Seminavis robusta	Ochrophyta	37,718	221 (231)
Thalassiosira pseudonana	Ochrophyta	11,566	70 (261)
Volvox carteri	Chlorophyta	14,247	27 (45)

photosynthesis https://protists.sbs.ntu.edu.sg/family/view/4435), co-expression clusters (photosynthetic cluster https://protists.sbs.ntu.edu.sg/cluster/graph/522), neighborhoods (photosynthetic neighborhood https://protists.sbs.ntu.edu.sg/network/graph/25954), phylogenetic trees (the tree of the photosynthetic family https://protists.sbs.ntu.edu.sg/tree/view/4435), Pfam domains (PsaD domain involved in photosynthesis https://protists.sbs.ntu.edu.sg/interpro/view/3008), and Gene Ontology terms (photosystem I GO term https://protists.sbs.ntu.edu.sg/go/view/7394). Each page contains additional information relevant to the type of data being displayed. For instance, the gene pages contain information about CDS and protein sequences, functional annotations, expression profiles, co-expression neighborhoods, and more. On the other hand, Gene Ontology pages show GO annotations, the genes in the 17 protists with the same GO term, and enriched co-expressed clusters for genes that have that particular GO term. The database can be queried in multiple ways, e.g., by using gene identifiers, keywords (https://protists.sbs.ntu.edu.sg/search/advanced), BLAST, GO terms, protein domain identifiers or enriched co-expression clusters (https://protists.sbs.ntu.edu.sg/search/enriched/clusters). The features page (www.protist.guru/features) lists a complete description of search functions and tools.

To exemplify how our tool can be used to uncover novel genes and conserved gene clusters in biosynthetic pathways, we analyzed the secondary carotenoid biosynthesis pathway in *Haematococcus lacustris* via co-expression analysis. *Haematococcus lacustris* is a unicellular freshwater microalga that is a rich source of astaxanthin, a highly valued red xanthophyll known for its potent antioxidant activity.[9] Since phytoene is the first carotenoid precursor for astaxanthin biosynthesis, we queried our database with phytoene desaturase, one of the first two fundamental enzymes that catalyzes the conversion of C40 phytoene to ζ-carotene, an essential precursor for beta carotene, and hence astaxanthin.

The gene page for phytoene desaturase (*GFH06801*) (https://protists.sbs.ntu.edu.sg/sequence/view/216508) contains the gene's expression profile, nucleotide and protein sequences, and links to co-expression and functional information. Stress-inducing conditions have been shown to increase the yield of astaxanthin in *Haematococcus lacustris* cells by causing a morphological transformation of vegetative, green, motile cells to mature, non-motile cysts filled with red astaxanthin.[16] Thus, to increase the production of high-value compounds, we can analyze the gene expression profiles of different strains, developmental stages and growth conditions, to reveal when the key enzymes are most highly expressed. In line with this, *GFH06801* shows highest expression in strain CCAP34/8 under nitrate limitation (Figure 1(A)),[10] and in NIES-144 strain grown under high light for 24 hours. Consequently, these two conditions can be used alone or in combination to increase astaxanthin production.

To reveal other genes likely to be important for astaxanthin biosynthesis, we clicked on the co-expression cluster of the phytoene desaturase gene (Cluster 7: https://protists.sbs.ntu.edu.sg/cluster/view/3493). The cluster page can be navigated from the gene page and displays the average expression profile of all genes in the cluster, significantly enriched Gene Ontology (GO) terms (corrected p-value <0.05), InterPro Domains, and gene families found in the cluster.

The co-expression Cluster 7 consists of 76 genes that are involved in biosynthetic processes of carotenoid, pyruvate synthesis and fatty acids

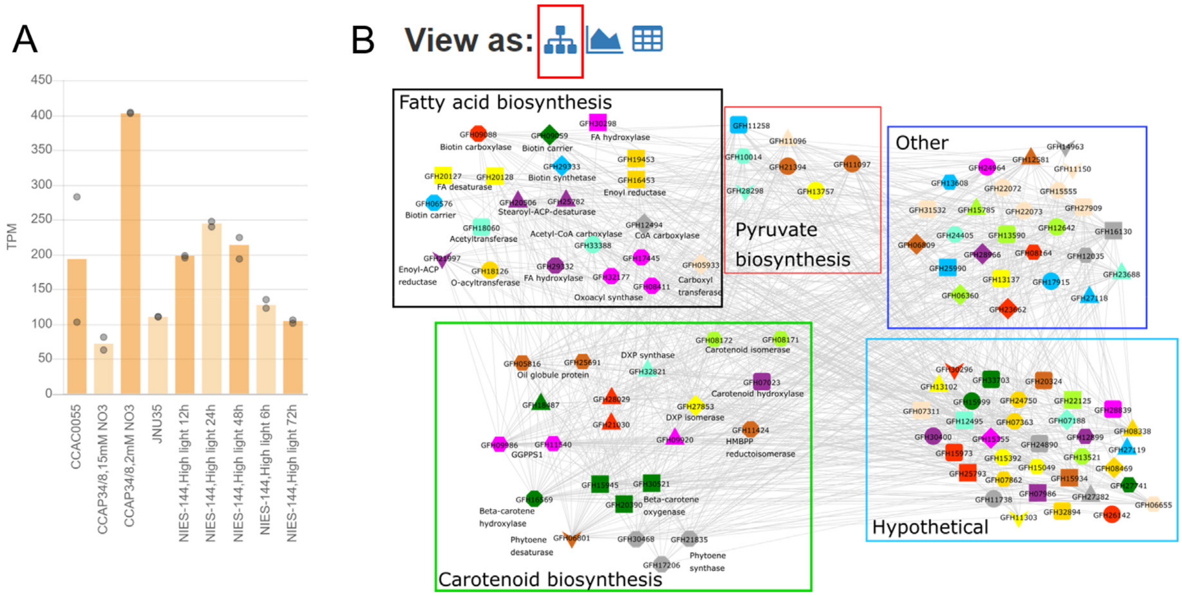

Figure 1. Expression profiles and co-expression networks of *Haematococcus lacustris*. (A) Expression profile of *GFH06801*. Different strains and sample conditions are represented on the x-axis whereas gene expression values in Transcript Per Million (TPM) are represented on the y-axis. Dots and bars represent the maximum/minimum gene expression values and average gene expression values, respectively. (B) Co-expression cluster 7 containing the phytoene desaturase *GFH06801*. Nodes represent genes and co-expressed genes are connected by edges in the network. Node colors and shapes indicate genes belonging to same orthogroups. To view the cluster, please click on the graph icon (indicated by red rectangle on top of the figure), found on the cluster page.

(Figure 1(B)). The carotenoid biosynthesis genes contain several enzymes involved in the biosynthesis of lycopene, a precursor for astaxanthin (green box, Figure 1(B)).[23] Pyruvate is an even more upstream precursor for carotenoid biosynthesis (red box), which is fed into mevalonate (MVA) or MEP pathway for isopentenyl pyrophosphate (IPP) and dimethylallyl pyrophosphate (DMAPP) synthesis.[14] We also observed many genes involved in fatty acid biosynthesis (black box). Stoichiometric coordination and interdependence between fatty acid biosynthesis and astaxanthin production pathways were observed in *Haematococcus lacustris,* with some fatty acid biosynthesis acyltransferases postulated to be involved in astaxanthin esterification.[4] Interestingly, we found two oil globule proteins (green box), which is in line with astaxanthin being found in oil bodies in *Haematococcus lacustris*.[20] Furthermore, we also found other genes not directly involved in carotene biosynthesis (dark blue box), and many hypothetical proteins (light blue box). The hypothetical proteins are good candidates for further study in their involvement in astaxanthin biosynthesis.

The gene pages also provide links to the respective gene families and gene trees. For example, the phytoene desaturase gene (*GFH06801*) belongs to a gene family (https://protists.sbs.ntu.edu.sg/family/view/1687) comprising 25 genes found in 16 species (Figure 2(A)). The phylogenetic relationships between the genes can also be viewed by clicking on the phylogenetic tree link (https://protists.sbs.ntu.edu.sg/tree/view/44863), available on the gene page or gene family page. The gene family and phylogenetic tree revealed that the phytoene desaturase gene family comprises genes with at most three duplications in *Cladocopium* sp. *clade C* (Figure 2(A)), with some of the duplications taking place within the analyzed species (e.g., clades of CLADO and EMIHU, Figure 2(B)).

Co-expression clusters can be conserved across species, which is a powerful approach to identify functionally related groups of genes across species.[18] Cluster 7 from *Haematococcus lacustris*'s page contains a 'Similar Clusters' table (https://protists.sbs.ntu.edu.sg/cluster/view/3493), where the Jaccard Index is used to indicate cluster similarity.[21] The Jaccard index ranges from 0 (genes in cluster A do not belong to any orthogroups of genes in cluster B) to 1 (all genes in cluster A belong to orthogroups of genes in cluster B, and vice versa). The most similar cluster to Cluster 7 was found by clicking on the Jaccard Index column and sorting the values in descending order, which revealed a similar cluster in another astaxanthin producing microalga, *Chromochloris zofingiensis*. Upon clicking 'Compare' to compare the clusters, the tool showed the co-expression networks comprising genes in the two conserved clusters (Figure 2(C)). The genes conserved between these

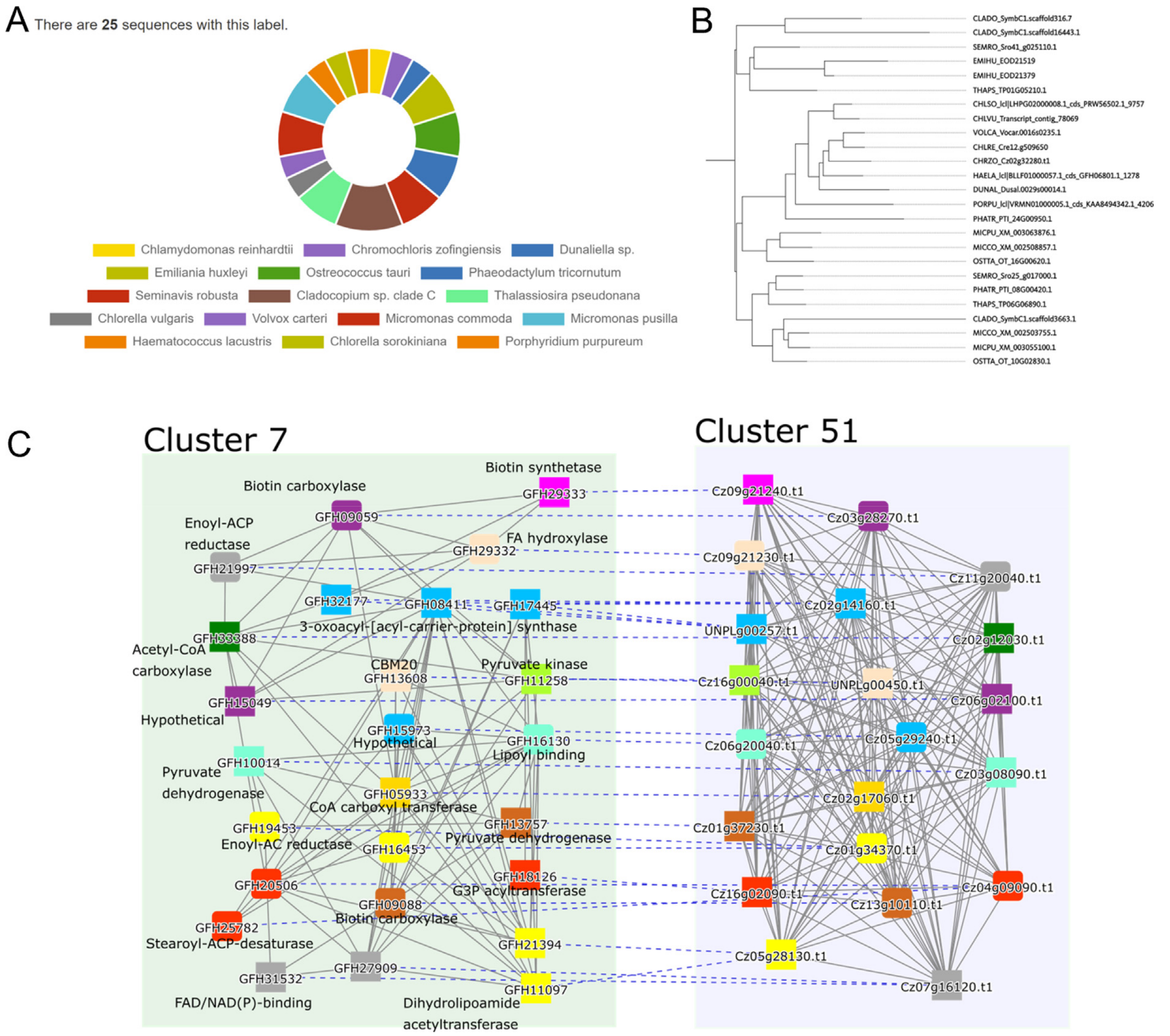

Figure 2. Comparative genomic and transcriptomic analyses. (A) Distribution of 25 genes in orthogroup OG_01_0001688 across all 17 protists. The colors indicate the different species. (B) Phylogenetic tree for OG_01_0001688 built using neighbor joining. (C) Comparative gene co-expression networks for similar clusters in *Haematococcus lacustris* and *Chromochloris zofingiensis*. Only genes that belong to orthogroups found in the two clusters are shown. Node colors and shapes indicate genes belonging to same orthogroups. Solid lines denote co-expressed genes whereas dashed lines denote genes belonging to the same orthogroups in the two species.

clusters are involved in acetyl-CoA, pyruvate, and fatty acid biosynthesis, suggesting that a part of the transcriptional programs is conserved across the two algae. Interestingly, we also observed some hypothetical proteins, and their presence indicates a strong likelihood of their involvement in these processes.

Conclusion

We developed protist.guru, a comparative transcriptomics database with multiple tools to analyze and visualize gene co-expression networks and expression profiles among 17 protists. Through this database, we hope to aid the investigation of gene function in protists and facilitate a deeper understanding of these microbial eukaryotes. While our database is currently mainly focused on Archaeplastida and Ochrophyta due to limited data from other supergroups, our aim is to expand protist.guru with data from other protists.

Declaration of interests

The authors declare that they have no known competing financial interests or personal relationships that could have appeared to influence the work reported in this paper.

DATA AVAILABILITY

We used publicly available data

Appendix A. Supplementary material

Supplementary data to this article can be found online at https://doi.org/10.1016/j.jmb.2022.167502.

Received 22 December 2021;
Accepted 10 February 2022;
Available online 18 February 2022

Keywords:
protist;
expression;
co-expression;
function;
comparative

References

1. Bray, N.L. et al, (2016). Near-optimal probabilistic RNA-seq quantification. *Nature Biotechnol.* **34**, 525–527.
2. Buchfink, B. et al, (2014). Fast and sensitive protein alignment using DIAMOND. *Nature Methods* **12**, 59–60.
3. del Campo, J. et al, (2014). The others: our biased perspective of eukaryotic genomes. *Trends Ecol. Evol.* **29**, 252–259.
4. Chen, G. et al, (2015). Molecular mechanisms of the coordination between astaxanthin and fatty acid biosynthesis in Haematococcus pluvialis (Chlorophyceae). *Plant J.* **81**, 95–107.
5. Emms, D.M., Kelly, S., (2015). OrthoFinder: solving fundamental biases in whole genome comparisons dramatically improves orthogroup inference accuracy. *Genome Biol.* **16**
6. Ferrari, C. et al, (2018). PhytoNet: Comparative co-expression network analyses across phytoplankton and land plants. *Nucleic Acids Res.* **46**, W76–W83.
7. Ferrari, C., Mutwil, M., (2019). Gene expression analysis of Cyanophora paradoxa reveals conserved abiotic stress responses between basal algae and flowering plants. *New Phytol.*.
8. E. Haeckel, Generelle Morphologie der Organismen. Bd. 1, 574 pp.; Bd. 2, 462 pp, G. Reiner, Berlin, 1866.
9. Han, S.-I. et al, (2019). A novel approach to enhance astaxanthin production in Haematococcus lacustris using a microstructure-based culture platform. *Algal Res.* **39**, 101464
10. Hoys, C. et al, (2021). Unveiling the underlying molecular basis of astaxanthin accumulation in Haematococcus through integrative metabolomic-transcriptomic analysis. *Bioresour. Technol.* **332**, 125150
11. Ibarbalz, F.M. et al, (2019). Global Trends in Marine Plankton Diversity across Kingdoms of Life. *Cell* **179**, 1084–1097.e21.
12. Jones, P. et al, (2014). InterProScan 5: Genome-scale protein function classification. *Bioinformatics* **30**, 1236–1240.
13. Leinonen, R. et al, (2011). The Sequence Read Archive. *Nucleic Acids Res.* **39**, D19–D21.
14. Li, C. et al, (2019). Modular engineering for microbial production of carotenoids. *Metab. Eng. Commun.* **10**, e00118
15. Mahé, F. et al, (2017). Parasites dominate hyperdiverse soil protist communities in Neotropical rainforests. *Nature Ecol. Evol.* **1**, 91.
16. Minhas, A.K. et al, (2016). A Review on the Assessment of Stress Conditions for Simultaneous Production of Microalgal Lipids and Carotenoids. *Front. Microbiol.* **7**, 546.
17. Mutwil, M. et al, (2010). Assembly of an interactive correlation network for the Arabidopsis genome using a novel Heuristic Clustering Algorithm. *Plant Physiol.* **152**, 29–43.
18. Mutwil, M. et al, (2008). GeneCAT–novel webtools that combine BLAST and co-expression analyses. *Nucleic Acids Res.* **36**
19. Mutwil, M. et al, (2011). PlaNet: Combined sequence and expression comparisons across plant networks derived from seven species. *Plant Cell* **23**, 895–910.
20. Peled, E. et al, (2011). Isolation of a novel oil globule protein from the green alga Haematococcus pluvialis (Chlorophyceae). *Lipids* **46**, 851–861.
21. Proost, S., Mutwil, M., (2018). CoNekT: An open-source framework for comparative genomic and transcriptomic network analyses. *Nucleic Acids Res.* **46**, W133–W140.
22. Rhee, S.Y., Mutwil, M., (2014). Towards revealing the functions of all genes in plants. *Trends Plant Sci.* **19**, 212–221.
23. Shah, M.M.R. et al, (2016). Astaxanthin-Producing Green Microalga Haematococcus pluvialis: From Single Cell to High Value Commercial Products. *Front. Plant Sci.* **7**, 531.
24. Tan, Q.W. et al, (2020). LSTrAP-Cloud: A User-Friendly Cloud Computing Pipeline to Infer Coexpression Networks. *Genes* **11**, 428.
25. Usadel, B. et al, (2009). Co-expression tools for plant biology: Opportunities for hypothesis generation and caveats. *Plant Cell Environ.* **32**, 1633–1651.
