# Supplementary material for: PDBx/mmCIF Ecosystem: Foundational Semantic Tools for Structural Biology
Source: J Mol Biol. Author manuscript; Available in PMC 2023 Jun 26. (PMC10292674; doi:10.1016/j.jmb.2022.167599)
Supplement: Article [file NIHMS1907597-supplement-Article.zip › OpenPIP--An-Open-source-Platform-for-Hosting--Visualizi_2022_Journal-of-Mole.pdf]

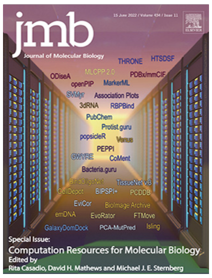

OpenPIP: An Open-source Platform for Hosting, Visualizing and Analyzing Protein Interaction Data

Mohamed Helmy [1†‡], Miles Mee [2‡], Aniket Ranjan [3], Tong Hao [4,5,6], Marc Vidal [4,5], Michael A. Calderwood [4,5,6], Katja Luck [7] and Gary D. Bader [8,9,10,11,12*]

1 - ***Department of Computer Science,*** *Lakehead University, Thunder Bay, ON, Canada*
2 - ***Ontario Institute for Cancer Research,*** *Toronto, ON, Canada*
3 - ***Indian Institute of Technology,*** *Kharagpur, India*
4 - ***Center for Cancer Systems Biology (CCSB),*** *Dana-Farber Cancer Institute (DFCI), Boston, MA, USA*
5 - ***Department of Genetics,*** *Blavatnik Institute, Harvard Medical School (HMS), Boston, MA, USA*
6 - ***Department of Cancer Biology,*** *Dana-Farber Cancer Institute (DFCI), Boston, MA, USA*
7 - ***Institute of Molecular Biology (IMB),*** *Mainz, Germany*
8 - ***The Donnelly Centre,*** *University of Toronto, Toronto, ON, Canada*
9 - ***Department of Molecular Genetics,*** *University of Toronto, Toronto, ON, Canada*
10 - ***Department of Computer Science,*** *University of Toronto, Toronto, ON, Canada*
11 - ***Princess Margaret Cancer Centre,*** *University Health Network, Toronto, ON, Canada*
12 - ***The Lunenfeld-Tanenbaum Research Institute,*** *Sinai Health System, Toronto, ON, Canada*

Correspondence to Gary D. Bader: The Donnelly Centre, University of Toronto, Toronto, ON, Canada. *gary.bader@utoronto.ca (G.D. Bader) @garybader1 (G.D. Bader)*
https://doi.org/10.1016/j.jmb.2022.167603
Edited by Michael Sternberg

Abstract

Knowing which proteins interact with each other is essential information for understanding how most biological processes at the cellular and organismal level operate and how their perturbation can cause disease. Continuous technical and methodological advances over the last two decades have led to many genome-wide systematically-generated protein–protein interaction (PPI) maps. To help store, visualize, analyze and disseminate these specialized experimental datasets via the web, we developed the freely-available Open-source Protein Interaction Platform (openPIP) as a customizable web portal designed to host experimental PPI maps. Such a portal is often required to accompany a paper describing the experimental data set, in addition to depositing the data in a standard repository. No coding skills are required to set up and customize the database and web portal. OpenPIP has been used to build the databases and web portals of two major protein interactome maps, the Human and Yeast Reference Protein Interactome maps (HuRI and YeRI, respectively). OpenPIP is freely available as a ready-to-use Docker container for hosting and sharing PPI data with the scientific community at http://openpip.baderlab.org/ and the source code can be downloaded from https://github.com/BaderLab/openPIP/.

© 2022 The Author(s). Published by Elsevier Ltd. This is an open access article under the CC BY license (http://creativecommons.org/licenses/by/4.0/).

Introduction

Cellular components such as proteins, RNA, and metabolites rarely function in isolation, but rather form immense molecular interaction networks that mediate cellular function and organization.[1] Over the last two decades, protein interactome mapping has been undertaken for many organisms, including

0022-2836/© 2022 The Author(s). Published by Elsevier Ltd.This is an open access article under the CC BY license (http://creativecommons.org/licenses/by/4.0/).

human, using techniques such as yeast two-hybrid (Y2H), affinity purification or co-fractionation coupled to mass spectrometry,[2] as well as DNA sequencing-based techniques.[3] These systematically generated data resources represent unprecedented opportunities to identify novel protein complexes, predict functions for uncharacterized genes, predict molecular mechanisms of disease, and study organizational principles of cellular function.[4,5] To enable easy access for a wide community of researchers, these protein–protein interaction (PPI) data must be provided on publicly available web servers for browsing, search, filtering, visualization, annotation, analysis, integration, and download.

There are several services and platforms to publicly host and visualize PPI data such as IntAct[6] and BioGRID.[7] These services allow the results of PPI mapping projects to be hosted online together with all other curated, predicted, and uploaded PPI data.[8] Ideally, all published PPI data is deposited with one of these general and standard PPI databases. However, producers of large-scale PPI datasets and users have needs that are not met by these existing databases and may need additional, more specialized databases. First, many biologist researchers want to be able to query a specific large-scale dataset separate from others to ease access and also because each dataset has different properties and types of data it contains (e.g. mass-spectrometry vs. yeast two-hybrid data). Second, users should have access to as detailed experimental information as possible to be able to interpret the data correctly and increase reproducibility in follow-up experiments. Information such as protein isoforms, number of screens in which a PPI was detected, tags, fusion configurations (N- or C-terminal tagging), and orientations (which interaction partner was bait, which one prey) are often not provided in agglomerated PPI databases. Third, funding agencies and journals generally require public access to generated PPI data prior to publication to accelerate scientific discoveries and peer-review, but public PPI web servers generally do not accept unpublished data that may also have specialized updating needs (e.g. quarterly reporting to a funding agency). Fourth, reviewers often expect a custom data portal to accompany a publication to enable them to more easily peer-review the data, in addition to requiring the data to be submitted to a public, centralized and standard database. For these reasons, producers of large protein interactome mapping projects, as well as their users, benefit from dataset-specific web portals.

To address these needs, we developed the Open-source Protein Interaction Platform (openPIP), a web-based software tool that provides the protein interactome research community with a deployment-ready platform to store, search, analyze, and visualize PPI data associated with a single experimental method. OpenPIP is deployed using a Docker container, PPI data is uploaded using Proteomics Standards Initiative Molecular Interactions (PSI-MI)[9] formatted files, and an administrator interface enables customization of the appearance and page contents, without any need to modify source code. It also links to multiple analyses for protein sets including Gene Ontology (GO), pathway, and protein complex enrichment analyses.

The source code for openPIP is written in PHP and is freely available for customization as needed. OpenPIP was successfully implemented to build the web portals of two major, frequently accessed protein interactome datasets, the Human and Yeast Reference Interactomes (HuRI and YeRI, respectively).[5,10]

Materials and Methods

Implementation

OpenPIP is developed using open source technologies. The UI of the system (Supplementary Figure 1) is built using the Symfony PHP framework and makes use of several JavaScript libraries including jQuery [https://jquery.com/], jQuery UI [https://jqueryui.com/], Cytoscape.js[11] for network visualization, FooTable for creating results tables [https://fooplugins.github.io/FooTable], qTip2 for pop-ups [https://www.qtip2.com/], Spectrum for color pickers [bgrins.github.io/spectrum], and TinyMCE for text editing [https://www.tinymce.com] (Figure 1). OpenPIP also links to web services including g:Profiler for enrichment analysis,[12] and UniProt and Ensembl[13,14] to access information on proteins. The database in openPIP is implemented using MySQL which runs as a containerized microservice over Docker Engine on a Linux operating system. Supplementary Figure 2 shows the database schema of openPIP. The openPIP application is served using the php:Apache web server.

The system is developed to create an interface to a protein interaction database built based on PSI-MI TAB format. Each page in openPIP has a navigation header containing links to the main functionalities of the system. The navigation menu changes based on whether the system is in use by a user or an administrator. If no user is logged in, the navigation menu shows links to Home, Search, Downloads, About, FAQs, Contact, Register and Login pages (Supplementary Figure 1(A)). If a user is logged in, the menu shows links to Profile and Logout instead of Register and Login pages, respectively (Supplementary Figure 1(B)). If an administrator is logged in, the navigation menu shows links to admin pages including Data upload, Files upload, Announcements and Settings pages, in addition to the content pages (Supplementary Figure 1(C)).

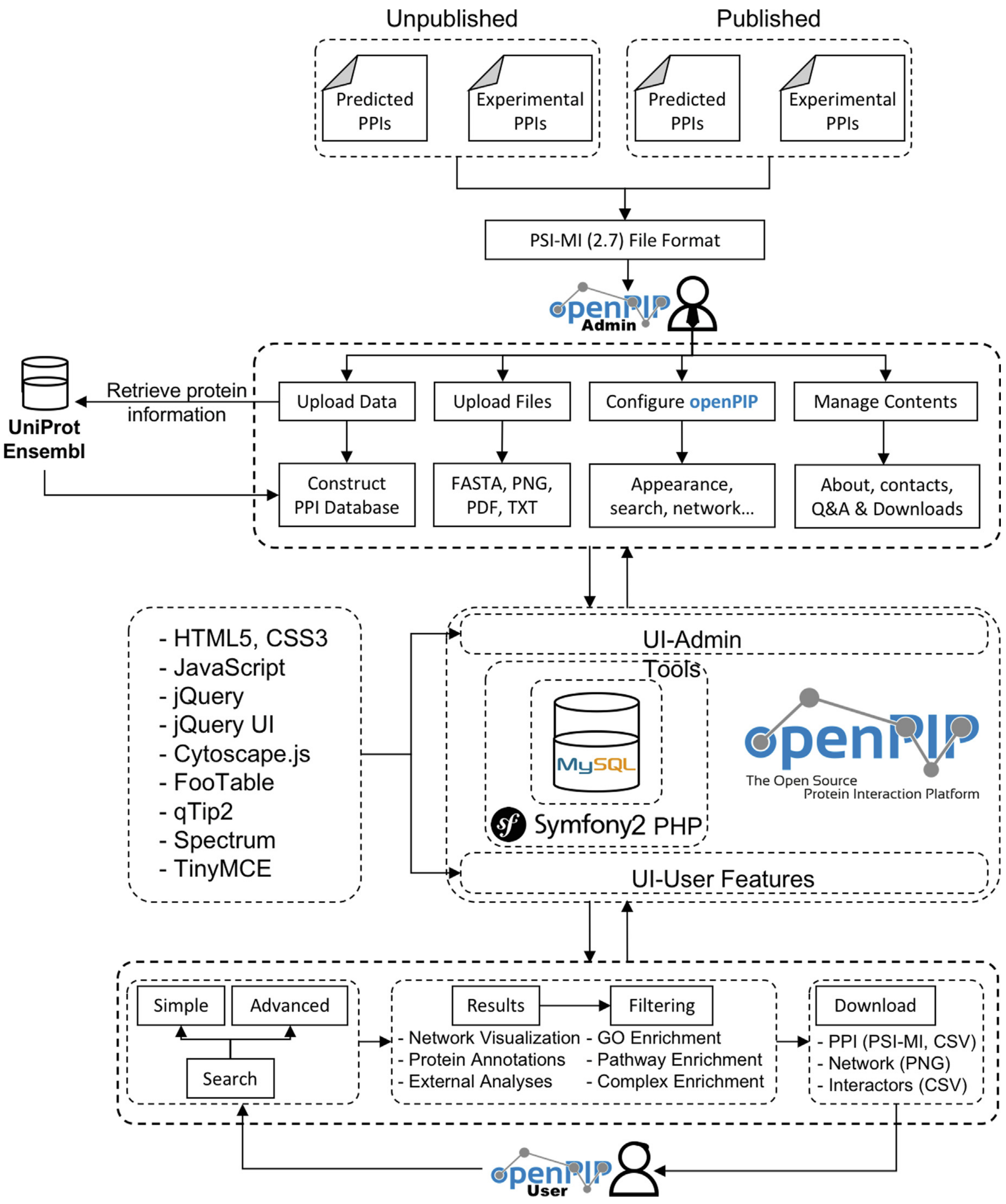

Figure 1. The architecture and workflow of openPIP.

Each of the administrator functionalities is described below.

Deployment

OpenPIP can be installed using a pre-configured Docker container available in the openPIP GitHub repository [github.com/BaderLab/openPIP]. Alternatively, openPIP can be installed directly from the source code available on the openPIP GitHub repository on a Linux server. The Docker container includes microservices of openPIP php:7.2.0-apache server and MySQL8.0 instance running through Docker Compose over Docker Engine on the Ubuntu 20.04 operating system. The source code runs on Linux operating systems. It only requires Docker Engine installed in the system and the other applications run as a combination of microservices defined in a Docker Compose file. It can be configured to use Apache

or Nginx servers, and requires PHP 5.6 or later and MySql 5.0 or later.

Results

Portal usage

PPI database construction. OpenPIP converts the PPI information provided in the PSI-MI TAB format into a relational database (Figure 1). During this process, openPIP collects various information about the interacting proteins from online resources. For each protein, openPIP collects the protein name, sequence and description from UniProt and Ensembl databases.[13,14] The collected information enriches the database contents and helps facilitate the search process.

Querying the PPI data. OpenPIP provides a search form where one or more protein names, UniProt identifiers (IDs) or Ensembl IDs can be entered as a new-line separated list to search for interactions involving these proteins. When the user begins typing a search term, openPIP will show a list of terms containing the input, enabling the user to autocomplete their search term. If any of the search terms are not present in the database, the user is notified and given the option to remove them from the search. Advanced search options are also available to filter the search results based on score, interaction category, and output format. The default search result returns all direct interactions with the query proteins as well as all interactions among the interactor proteins. The user can also choose to only return interactions between query proteins or only direct interactions with the query proteins and not among the interactor proteins. These restricted PPI search features are absent from widely used public PPI databases and have been designed in collaboration with PPI data generation groups based on their needs. For example, BioGRID only allows searching with a single query protein while IntAct can only retrieve all interactions involving any of the query proteins without being able to restrict it to interactions between query proteins only (Table 1). OpenPIP also allows the administrator to define search terms that can serve as examples in the query interface (Figure 2).

The search form on openPIP provides a set of filters and cutoffs to help perform more specific search queries (Figure 2). It provides an 'interactions' filter that creates a query to search the interactions between the proteins provided in the search box only, and an 'interaction status' filter that filters the interactions for a specific type or status. The type/status refers to the source of the interaction, i.e. a method or dataset or publication. This information is extracted from the PSI-MI files. Results can also be filtered using interaction scores, if provided in the PSI-MI file. In all cases, the search form allows the user to choose between the visualization of the network or to return the search results in a downloadable file format directly. This is particularly useful for big queries when web-browser-based network display might be too slow. The "Interactors" tab on the search form is automatically filled with the genes from the search results, allowing the user to remove interactors from the network or add new proteins to the query gene interactors list.

Results visualization, refinement, and download. The search results page of openPIP has several features to present the results in a user-friendly way. OpenPIP provides the user with (1) a summary of the search parameters and results (Figure 2), (2) a network visualization of the search result (Figure 2), (3) a table of interactions (Figure 2), (4) refinement and filtering options for the results (Figure 2), (5) options for downloading interaction and protein search results (Figure 2) and (6) links to various analysis tools based on the search results.

The returned PPI network is visualized using Cytoscape.js[11] (Figure 2). The nodes are color-coded to distinguish the query proteins from their interactors. The edges are color-coded according to the type of the interaction, and the edge weight (width) reflects the confidence score of the interaction, if available. All the color codes are customizable by the administrator (see below). Hovering the cursor over a node in the network causes the connected interaction edges and protein nodes to be highlighted. A button at the bottom left of the visualization panel allows the user to toggle whether the highlighting on hover is active. OpenPIP provides five different network layouts, provided by Cytoscape.js, that can be used to change the visualization of the interaction network including CoLa, CoSE,[15] concentric, circle, and grid layouts. CoLa is the default layout for single query gene networks and provides a nice animation and node spacing for relatively small networks. CoSE is the default multi-query gene and provides better spacing and is the default for larger networks (Supplementary Figure 4). Clicking a node (protein) will display a popup that shows more information about the protein including a brief description of its function, UniProt and Ensembl IDs, and a link to the protein sequence, as well as an option to submit a new search using this protein (Figure 2). Clicking an edge will display a popup that shows interaction details such as screens and datasets in which the PPI was identified along with genetic construct configuration and orientation information as well as interaction score(s), if available. Any experimental detail that is provided in the PSI-MI file can in this way be made easily accessible to the user for PPIs of interest, in contrast to most public PPI databases,

Table 1 Comparison between the features of openPIP, BioGRID and IntAct.

Task	openPIP	BioGRID	IntAct
Querying or filtering results for a specific dataset	✓	✗	✗
Retrieve isoform-specific PPI data, if available from experiment	✓	✗	✓
Retrieve screen-specific info from a selected dataset	✓	✗	✗
Filter results for type of interaction data (i.e. association versus direct interaction)	✓	✗	✓
Allow simultaneous search with multiple query proteins	✓	✗	✓
Allow search for PPIs only between query proteins	✓	✗	✗
Retrieve information on fusions, configurations, and orientations of both interaction partners from experiment	✓	✗	✓
Filter results for tissue expression	✓	✗	✗
Visualize search results in interactive network in browser	✓	✓	✓
Computation of enrichment statistics on search result	✓	✗	✗

which do not store or display this information (Table 1). Hovering over any node will highlight all its interactors and will hide the rest of the nodes.

The interactions table lists all the interactions in the returned network. For each interaction, the table displays the two interactors, the interaction confidence score and the dataset(s) where this interaction is identified, if available. Clicking on any row in the interactions table will highlight the corresponding nodes and edge in the interactions network above. The data in the table can be sorted using the interactors' names or the interaction scores. The interaction table has options to sort by interactor gene names alphabetically and by scores. The interactions table also provides a search field to enable the user to search within the interaction table. By entering a gene name in the search field, interactions containing that name will be shown in the table. This allows users to find specific interactions within large networks by searching the interaction table and clicking on the row to highlight the interaction in the network.

The search results can be further filtered using the interaction confidence scores, the interaction type, or protein annotation information, i.e. tissue expression or cellular localization data (Figure 2).

The interactions returned in the search results can be downloaded in PSI-MI tab (v2.7), SIF and CSV formats. The set of interactors can also be downloaded in CSV format. The protein sequences for the set of interactors can be downloaded in FASTA format. An image of the network can be downloaded in PNG format (Figure 2).

Integration with other tools. The search results can be exported to several publicly available analysis tools. The network analysis and gene function prediction can be performed by submitting interactors of the interaction networks to GeneMANIA[16] and STRING.[17] Linking to pathway information can be performed by exporting the data to Reactome[18] and Pathway Commons.[19] Gene set enrichment analysis can be performed using DAVID[20] and g:Profiler.[12] Proteins can also be submitted for querying in the cBioPortal,[21] the Complex Portal,[22] and to IntAct[6] databases. The interaction network can be exported and opened directly in the Cytoscape desktop software with one click.[23] This requires Cytoscape desktop software and the Cytoscape 'cyREST' app[24] to be installed and running. Cytoscape desktop can handle large networks that are not viewable in the web-browser within openPIP due to browser technology limitations.

Support for touch and small screens. OpenPIP provides several features and support for making the system friendly for mobile devices, touch screens and small screens to improve accessibility. Supplementary Figure 3 shows the openPIP main page and search results page displayed on a mobile phone screen.

Portal customization

OpenPIP is highly customizable. Through the administrator tools, the website appearance and its content can be changed thereby reducing the need to modify source code and for computational expertise. However, the source code is freely available and can be modified to meet specific needs, if required. Through the administrator tools, the website administrator can perform the following adjustments:

Interface customization. The Global Settings tab on the website settings page enables changing the global appearance of the website. This includes changing the website title, adding a short title, and choosing a color scheme for the whole website (main color, header color, logo color and buttons color). The administrator can also add custom text for the website footer in a rich text box that supports HTML formatting. The website's URL can be set to customize internal links. The links, in this case, can be relative to this variable instead of adding absolute URLs. Therefore, when switching the host server and changing this URL variable accordingly, all internal links will be automatically updated (Supplementary Figure 5).

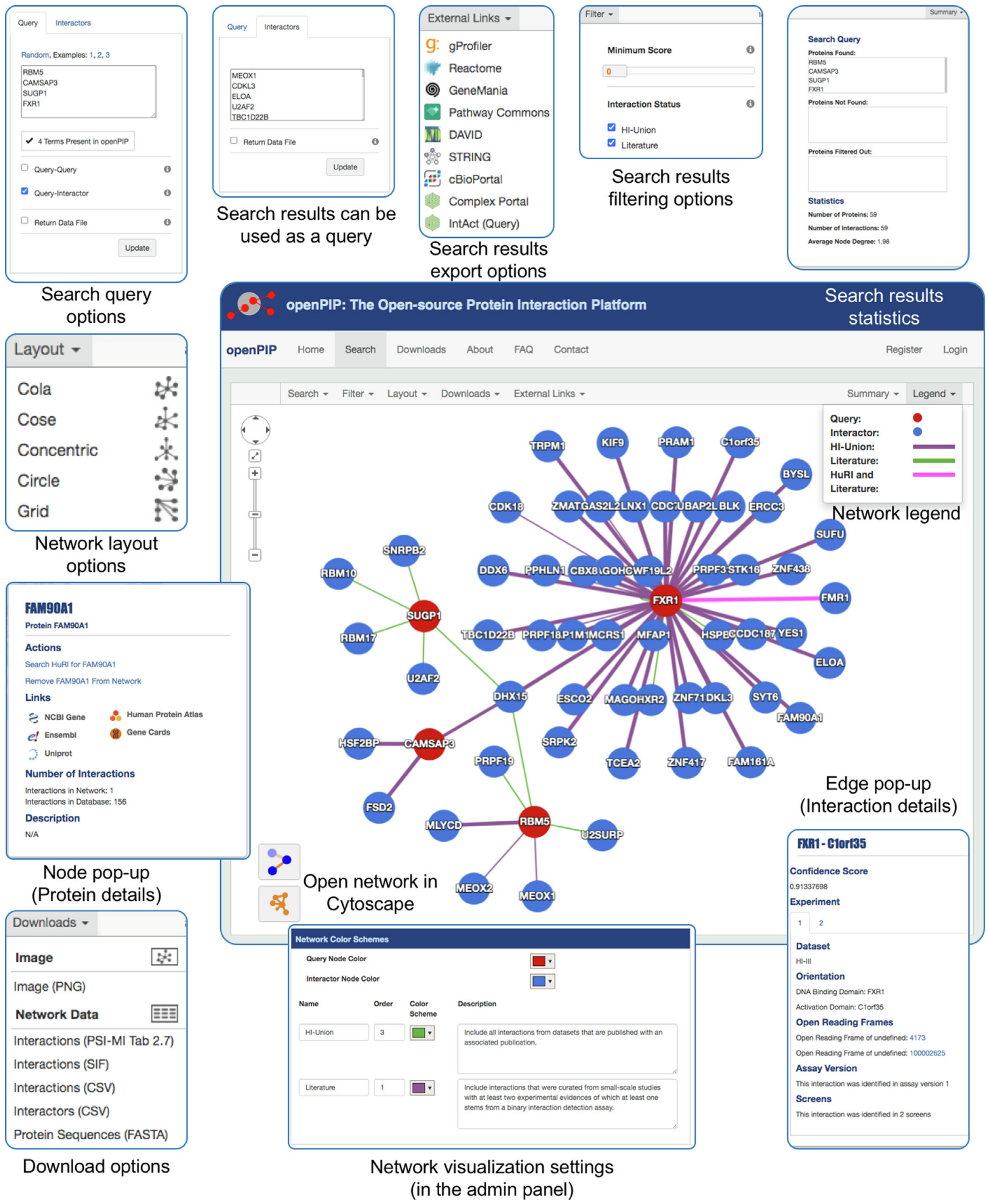

Figure 2. The search and results visualization and download options of openPIP.

Search option customization. Search forms should have examples for possible search keywords to improve the usability of web-based databases.[25] The administrator can define up to three sets of search examples (Supplementary Figure 6(A), top panel). Furthermore, a random example can be generated on-demand from the search form.

Customization of the network visualization. The color-coding of the network is important for interpreting the search results, i.e. for distinguishing query proteins from their interactors. The administrator can customize the network color-coding by choosing the colors for the query and interactor proteins as well as for each interaction type. The types of interactions are

dynamically obtained from the PSI-MI file during the data upload process. Then, openPIP creates a list of unique types and lists them in the Network Color Schemes section in the administrator tools. This section allows editing the name of each type, choosing a color for the edge of this type, and adding a description that the user can read when clicking the help sign (?) next to each type in the advanced search form (Supplementary Figure 6 (A), bottom panel).

Data upload. The data upload function allows the administrator to upload protein interaction data in PSI-MI TAB format. The PSI-MI file should follow the standard PSI-MI TAB format v2.7. The data upload function automatically collects pertinent information from the PSI-MI file (e.g. interaction types, see above) during the upload. The upload time increases for larger PPI datasets. We provide an estimator for the data upload time with a countdown timer. The data upload function also performs all other data manipulation processes required, such as delete and update.

File upload. OpenPIP provides a file upload function where the website administrator can upload a single file, multiple files or a folder (all files in the folder), which will then be available for download by users on the Downloads web page. Each file upload starts an independent process that can be monitored through a progress bar. This is particularly useful with large file uploads to the server (e.g. individual FASTA files for all protein sequences). The openPIP administrator can manage such tasks easily via the administrator user interface.

Editable page contents. To ease adding and editing the content of the various web pages in openPIP, the administrator tools, openPIP provides four preconfigured pages, About, FAQs, Contact, and Downloads that can be edited through a rich text box that supports HTML code. The content can be written directly in the editor or copied from word-processing software, such as Microsoft Word or Google Docs, or copied as HTML code. The editor retains the HTML formatting of the source (Supplementary Figure 61 (B)).

The Downloads page is editable, similar to the other three pages, but it has one more feature. It comes with two preconfigured download options (1) Download All Datasets, which links to a downloadable PSI-MI file and a CSV file of all the data in the database and, (2) Dataset Downloads, which creates a downloadable PSI-MI file and a CSV file of each dataset in the database. Both options can be enabled or disabled by the administrator (Supplementary Figure 6(C)).

Register new administrators. OpenPIP enables the administrator to add new administrators with identical privileges.

Add/delete news and announcements. The administrator can add news and announcements, such as the release of a new version of the data or server maintenance times through a designated form in openPIP administrator tools. The administrator can choose to show or hide the announcements and which announcement to show on the home page.

OpenPIP Implementations

HuRI

We used openPIP to set up a PPI database and web portal to host more than 77,000 human binary PPIs of which 64,000 (the HuRI dataset) were generated in a series of genome-wide screens using Y2H as a primary screening method and other binary PPI assays operated in mammalian expression systems for validation.[5] The remaining 13,000 PPIs represent a carefully filtered subset of literature-curated PPIs with multiple lines of experimental evidence of which at least one is a binary assay. Experimental tests have shown that HuRI PPIs reproduce at a rate that is indistinguishable from the rate at which these literature-curated PPIs reproduce in orthogonal PPI assays.[5] This web portal thus hosts the largest, high-quality dataset of human binary PPIs. We used the default capabilities of openPIP to display on the results page which PPIs originate from HuRI, literature, or both sources (Figure 2). Furthermore, upon clicking an edge, we provide experimental details about PPIs, which are important for follow-up studies and which are difficult to obtain from public PPI databases such as IntAct and BioGRID that also contain HuRI data. All this information was provided in a single PSI-MI-TAB upload file. We used openPIP's protein annotation feature to add tissue gene expression data from the GTEx consortium to the web portal.[26] Users can filter a result network for proteins expressed in user-selected tissues. Via the administrator panel we added extensive documentation to the web portal as part of the About and FAQs web pages, which, among other things, explain how the data was generated and how it should be interpreted to avoid misuse. This important level of detail cannot currently be provided in the more commonly used PPI databases, which aggregate data from thousands of different sources. The HuRI web portal was used to periodically release HuRI data as it was generated, long before its official publication, and to host the final published data.

YeRI

OpenPIP was also used to set up the PPI database and web portal of YeRI,[10] an all-versus-all budding yeast binary protein interaction dataset. The data contains a binary map of 9,500 interactions between 4,000 yeast proteins. YeRI's generated data was integrated with three previously published yeast binary maps and compared against systematic co-complex association network maps and different global functional profile similarity networks (PSN). The YeRI PPI database and portal used a modified openPIP installation where the source code was modified to better match the needs of the project. Specifically, we removed the tissue expression filtering feature, which is not applicable to budding yeast, and an option to change the network color codes was added to the bottom of the network legend. Pathway Commons and cBioPortal were removed from the list of external links, as they are human-focused databases. The rest of YeRI's portal features are similar to the HuRI installation.

These two public instances of openPIP demonstrate the utility and versatility of the platform to host a project's data from pre-publication to post-publication and either to be used with its default features or to be customized by modifying its source code.

Discussion

Scientific data should be made easily accessible to the research community. For that purpose, standardized data formats, databases, and web portals have been developed that facilitate data sharing. Molecular interaction data is generated by an immense diversity of different assays producing vastly different types of data, ranging from direct, biophysical evidence for interaction all the way to co-localization, which is biochemically indirect. Proteins exist in different isoforms and are tested for interaction in full length or as fragments. Especially for large PPI datasets to which many users seek access, data producers seek user-friendly ways (and are requested for by funding agencies and peer-reviewers) to provide access to their data at various levels, from interactions at the gene, protein product or isoform levels, as well as experimental parameters, such as tag type, screen identity, and other details. Despite the fact that the PSI-MI format has the capacity to contain detailed experimental information, many public PPI resources do not share these annotations in search results. They have also not implemented dataset-specific opportunities for search and visualization nor dataset-specific documentation. In Table 1 we provide a more comprehensive comparison of features offered by openPIP, BioGRID, and IntAct. It is therefore of high interest and relevance for data producers and the research community alike, to efficiently develop dataset-specific portals, in parallel to also depositing their data in widely known public repositories upon publication. Existing examples for dataset-specific portals are numerous, such as the BioPlex portal (https://bioplex.hms.harvard.edu/), the integration of various protein complex datasets by Marcotte and collaborators (https://proteincomplexes.org/), or the human BioID proximity dataset (https://cell-map.org/). OpenPIP has been developed to meet this need. It is an open-source software platform that enables building a PPI database and a web portal with a customizable web interface that enables searching, visualizing, analyzing and downloading of interaction data. OpenPIP provides protein interaction projects and individual laboratories in the field with an easy and customizable solution to build and deploy their own database and web portal with relatively low effort and high quality, compared to the traditional approach of implementing a new software portal from scratch for each project.

OpenPIP supports hosting multiple PPI datasets obtained with different methods. Datasets can be published or unpublished, experimental or computationally predicted and it is able to merge them and keep track of all these differences. However, they must be from the same organism. OpenPIP can in principle host any molecular interaction data that can be uploaded in PSI-MI format, especially if the data represents gene-gene relationships such as co-expression or genetic interaction data. However, the openPIP GUI is specifically designed for binary PPI data. Our future plan for openPIP is to add support for interaction data that involves other molecules than protein-coding genes or proteins, such as RNA, non-genic DNA, metabolites, or chemicals, and other physical interaction data types, such as Affinity Purification Mass Spectrometry (AP-MS). We will also implement additional demonstration datasets, such as affinity purification-mass spectrometry-based PPI data. Currently, web-portals implemented using openPIP can only make data available through the web interface. There is no application programming interface (API) access. Also, adding API access to the system so that the hosted data can be accessed remotely using scripting, independent from the web interface and automatically using PSICQUIC.[27] We furthermore plan to add support to the PSI-MI TAB format 2.8. Currently, some of the PPI data annotation features are only applicable to multicellular organisms and had to be manually removed from the code for the web portal implementation to host the YeRI dataset. In the future, we will adapt openPIP to enable administrators to enable or disable support for external tools via the admin panel.

Conclusion

OpenPIP provides a ready-to-use platform for building a database of gene-gene or protein–protein relationships, such as PPIs, including a web portal, search engine, and several on-demand analyses and annotations, simply by providing molecular interaction data in PSI-MI TAB format. Once installed, most administration, customization, data manipulation, and content update tasks are done through a GUI administrator tool. OpenPIP is useful for consortia implementing large protein interactome or other gene-gene functional relationship mapping efforts as well as for individual laboratories that generate molecular interaction data to make available their own user-friendly online repository to host, query, analyze, and visualize their data.

CRediT Author Statement

Mohamed Helmy: Formal analysis, Conceptualization, Writing – original draft. **Miles Mee:** Formal analysis. **Aniket Ranjan:** Formal analysis, Writing – review & editing. **Tong Hao:** Conceptualization, Writing – review & editing. **Marc Vidal:** Writing – review & editing. **Michael A.Calderwood:** Conceptualization, Writing – review & editing. **Katja Luck:** Conceptualization, Writing – original draft. **Gary D.Bader:** Conceptualization, Writing – review & editing, Supervision.

DATA AVAILABILITY

All source code is freely available and open source at https://github.com/BaderLab/openPIP/.

Acknowledgements

We thank Harold Rodriguez and Ruth Isserlin (The Donnelly Centre, University of Toronto) and Changyu Fan (Dana-Farber Cancer Institute, Harvard University) for technical support for openPIP and HuRI web portal servers.

Funding

OpenPIP development is funded by the US National Institutes of Health, “A human binary interactome reference map by 2020” grant number U41-HG001715 and through Google Summer of Code (GSoC) program to the National Resources for Network Biology (NRNB) to MM and AR. KL is funded by the Deutsche Forschungsgemeinschaft (DFG, German Research Foundation) – LU2568/1-1. This work was supported by NRNB (U.S. National Institutes of Health, National Center for Research Resources grant number P41 GM103504).

Declaration of Interest

The authors declare no financial/personal conflict of interest.

Appendix A. Supplementary material

Supplementary data to this article can be found online at https://doi.org/10.1016/j.jmb.2022.167603.

Received 30 November 2021;
Accepted 19 April 2022;
Available online 26 April 2022

Keywords:
protein interactions;
interactome;
protein interaction database

† Current address: Bioinformatics Institute (BII), Agency for Science, Technology and Research (A*STAR), Singapore, Singapore.
‡These authors contributed equally.

References

1. Vidal, M., (2001). A biological atlas of functional maps. *Cell* **104**, 333–339.
2. Luck, K., Sheynkman, G.M., Zhang, I., Vidal, M., (2017). Proteome-Scale Human Interactomics. *Trends Biochem. Sci.* **42**, 342–354.
3. Johnson, K.L., Qi, Z., Yan, Z., Wen, X., Nguyen, T.C., Zaleta-Rivera, K., Chen, C.-J., Fan, X., et al., (2021). Revealing protein-protein interactions at the transcriptome scale by sequencing. *Mol. Cell* **81**, 3877.
4. Huttlin, E.L., Bruckner, R.J., Navarrete-Perea, J., Cannon, J.R., Baltier, K., Gebreab, F., Gygi, M.P., Thornock, A., et al., (2021). Dual proteome-scale networks reveal cell-specific remodeling of the human interactome. *Cell* **184**, 3022e28–3040e28.
5. Luck, K., Kim, D.-K., Lambourne, L., Spirohn, K., Begg, B. E., Bian, W., Brignall, R., Cafarelli, T., et al., (2020). A reference map of the human binary protein interactome. *Nature* **580**, 402–408.
6. Orchard, S., Ammari, M., Aranda, B., Breuza, L., Briganti, L., Broackes-Carter, F., Campbell, N.H., Chavali, G., et al., (2014). The MIntAct project - IntAct as a common curation platform for 11 molecular interaction databases. *Nucleic Acids Res.* **42**, D358–D363.
7. Oughtred, R., Rust, J., Chang, C., Breitkreutz, B.-J., Stark, C., Willems, A., Boucher, L., Leung, G., et al., (2021). The BioGRID database: A comprehensive biomedical resource of curated protein, genetic, and chemical interactions. *Protein Sci.* **30**, 187–200.
8. Jeanquartier, F., Jean-Quartier, C., Holzinger, A., (2015). Integrated web visualizations for protein-protein interaction databases. *BMC Bioinformatics* **16**, 195.

9. Kerrien, S., Orchard, S., Montecchi-Palazzi, L., Aranda, B., Quinn, A.F., Vinod, N., Bader, G.D., Xenarios, I., et al., (2007). Broadening the horizon–level 2.5 of the HUPO-PSI format for molecular interactions. *BMC Biol.* **5**, 44.
10. Lambourne, L., Yadav, A., Wang, Y., Desbuleux, A., Kim, D.-K., Cafarelli, T., Pons, C., Kovács, I.A., et al., (2021). Binary Interactome Models of Inner- Versus Outer-Complexome Organization. *BioRxiv*.
11. Franz, M., Lopes, C.T., Huck, G., Dong, Y., Sumer, O., Bader, G.D., (2016). Cytoscape.js: a graph theory library for visualisation and analysis. *Bioinformatics* **32**, 309–311.
12. Reimand, J., Arak, T., Adler, P., Kolberg, L., Reisberg, S., Peterson, H., Vilo, J., (2016). g:Profiler-a web server for functional interpretation of gene lists (2016 update). *Nucleic Acids Res.* **44**, W83–W89.
13. Pundir, S., Martin, M.J., O'Donovan, C., (2017). UniProt Protein Knowledgebase. *Methods Mol. Biol.* **1558**, 41–55.
14. Yates, A., Akanni, W., Amode, M.R., Barrell, D., Billis, K., Carvalho-Silva, D., Cummins, C., Clapham, P., et al., (2016). Ensembl 2016. *Nucleic Acids Res.* **44**, D710–D716.
15. Dogrusoz, U., Giral, E., Cetintas, A., Civril, A., Demir, E., (2009). A layout algorithm for undirected compound graphs. *Inf. Sci. (Ny)* **179**, 980–994.
16. Warde-Farley, D., Donaldson, S.L., Comes, O., Zuberi, K., Badrawi, R., Chao, P., Franz, M., Grouios, C., et al., (2010). The GeneMANIA prediction server: biological network integration for gene prioritization and predicting gene function. *Nucleic Acids Res.* **38**, W214–W220.
17. Szklarczyk, D., Gable, A.L., Nastou, K.C., Lyon, D., Kirsch, R., Pyysalo, S., Doncheva, N.T., Legeay, M., et al., (2021). The STRING database in 2021: customizable protein-protein networks, and functional characterization of user-uploaded gene/measurement sets. *Nucleic Acids Res.* **49**, D605–D612.
18. Gillespie, M., Jassal, B., Stephan, R., Milacic, M., Rothfels, K., Senff-Ribeiro, A., Griss, J., Sevilla, C., et al., (2022). The reactome pathway knowledgebase 2022. *Nucleic Acids Res.* **50**, D687–D692.
19. Rodchenkov, I., Babur, O., Luna, A., Aksoy, B.A., Wong, J. V., Fong, D., Franz, M., Siper, M.C., et al., (2020). Pathway Commons 2019 Update: integration, analysis and exploration of pathway data. *Nucleic Acids Res.* **48**, D489–D497.
20. Huang, D.W., Sherman, B.T., Lempicki, R.A., (2009). Systematic and integrative analysis of large gene lists using DAVID bioinformatics resources. *Nat. Protoc.* **4**, 44–57.
21. Gao, J., Aksoy, B.A., Dogrusoz, U., Dresdner, G., Gross, B., Sumer, S.O., Sun, Y., Jacobsen, A., et al., (2013). Integrative analysis of complex cancer genomics and clinical profiles using the cBioPortal. *Sci. Signal.* **6**, pl1.
22. Meldal, B.H.M., Bye-A-Jee, H., Gajdoš, L., Hammerová, Z., Horácková, A., Melicher, F., Perfetto, L., Pokorný, D., et al., (2019). Complex Portal 2018: extended content and enhanced visualization tools for macromolecular complexes. *Nucleic Acids Res.* **47**, D550–D558.
23. Shannon, P., Markiel, A., Ozier, O., Baliga, N.S., Wang, J. T., Ramage, D., Amin, N., Schwikowski, B., Ideker, T., (2003). Cytoscape: a software environment for integrated models of biomolecular interaction networks. *Genome Res.* **13**, 2498–2504.
24. Ono, K., Muetze, T., Kolishovski, G., Shannon, P., Demchak, B., (2015). Cyrest: turbocharging cytoscape access for external tools via a restful API. [version 1; peer review: 2 approved]. *F1000Research* **4**, 478.
25. Helmy, M., Crits-Christoph, A., Bader, G.D., (2016). Ten simple rules for developing public biological databases. *PLoS Comput. Biol.* **12**, e1005128.
26. Melé, M., Ferreira, P.G., Reverter, F., DeLuca, D.S., Monlong, J., Sammeth, M., Young, T.R., Goldmann, J.M., et al., (2015). The human transcriptome across tissues and individuals. *Science* **348**, 660–665.
27. del-Toro, N., Dumousseau, M., Orchard, S., Jimenez, R.C., Galeota, E., Launay, G., Goll, J., Breuer, K., et al., (2013). A new reference implementation of the PSICQUIC web service. *Nucleic Acids Res.* **41**, W601–W606.
