# Supplementary material for: PDBx/mmCIF Ecosystem: Foundational Semantic Tools for Structural Biology
Source: J Mol Biol. Author manuscript; Available in PMC 2023 Jun 26. (PMC10292674; doi:10.1016/j.jmb.2022.167599)
Supplement: Article [file NIHMS1907597-supplement-Article.zip › GalaxyDomDock--An-Ab-Initio-Domain-domain-Docking-Web-Se_2022_Journal-of-Mol.pdf]

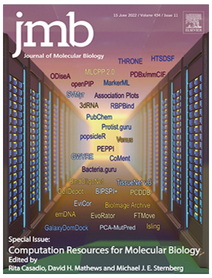

GalaxyDomDock: An *Ab Initio* Domain–domain Docking Web Server for Multi-domain Protein Structure Prediction[1]

Jayun Choi [1†], Taeyong Park [2†], Seung Yul Lee [3], Jinsol Yang [1] and Chaok Seok [1,2*]

*1 - **Department of Chemistry,** Seoul National University, Seoul 08826, Republic of Korea*
*2 - **Galux Inc,** Gwanak-gu, Seoul 08738, Republic of Korea*
*3 - **Department of Computer Science,** Seoul National University, Seoul 08826, Republic of Korea*

Correspondence to Chaok Seok: Department of Chemistry, Seoul National University, Seoul 08826, Republic of Korea. *jychoi9809@snu.ac.kr (J. Choi), 1ty.park@galux.co.kr (T. Park), triomphant1@snu.ac.kr (S. Yul Lee), m4u@snu.ac.kr (J. Yang), chaok@snu.ac.kr (C. Seok)*
https://doi.org/10.1016/j.jmb.2022.167508
Edited by Michael Sternberg

Abstract

A significant proportion of proteins comprise multiple domains. Domain–domain docking is a tool that predicts multi-domain protein structures when individual domain structures can be accurately predicted but when domain orientations cannot be predicted accurately. GalaxyDomDock predicts an ensemble of domain orientations from given domain structures by docking. Such information would also be beneficial in elucidating the functions of proteins that have multiple states with different domain orientations. GalaxyDomDock is an *ab initio* domain–domain docking method based on GalaxyTongDock, a previously developed protein–protein docking method. Infeasible domain orientations for the given linker are effectively screened out from the docked conformations by a geometric filter, using the Dijkstra algorithm. In addition, domain linker conformations are predicted by adopting a loop sampling method FALC. The proposed GalaxyDomDock outperformed existing *ab initio* domain–domain docking methods, such as AIDA and Rosetta, in performance tests on the Rosetta benchmark set of two-domain proteins. GalaxyDomDock also performed better than or comparable to AIDA on the AIDA benchmark set of two-domain proteins and two-domain proteins containing discontinuous domains, including the benchmark set in which each domain of the set was modeled by the recent version of AlphaFold. The GalaxyDomDock web server is freely available as a part of GalaxyWEB at http://galaxy.seoklab.org/domdock.

© 2022 Elsevier Ltd. All rights reserved.

Introduction

As protein structure predictions become more accurate, an increasing demand emerges for functional studies from predicted protein structures.[1] However, the structure prediction of proteins with multiple domains remains relatively unreliable when structures or sequences of related proteins that can be used to infer relative domain orientations are insufficient. For example, if multiple states with different domain orientations are involved in protein function and the structures of similar cases are unavailable, it is difficult to predict the domain orientations even when structures of individual domains are well defined.

Many multidomain proteins are expected to undergo conformational changes involving domain orientations. For example, c-Met, a receptor tyrosine kinase, undergoes orientational changes of its internal domains, as the receptor becomes active by forming a complex with HGF or NK1.[2] GPCRs of classes B, C, and F also undergo conformational shifts involving orientational changes between extracellular and transmembrane domains

0022-2836/© 2022 Elsevier Ltd. All rights reserved.

when bound to an agonist, from an inactive to an active state.[3]

Ab initio domain–domain docking can be adopted to predict the entire multi-domain protein structure when reliable structures are available for individual domains but when domain orientations are difficult to predict from information-based methods. Because *ab initio* docking methods can generate multiple orientations, models generated by docking can be used to test existing hypothesis or propose novel hypothesis on different functional states.

Several domain assembly methods, such as DEMO,[4] pyDockTET,[5] Rosetta,[6] and AIDA,[7] have been reported previously. DEMO performs "template-based" structure prediction for domain assembly. It is not compared in this paper because cases with available templates can be predicted accurately by using more recent structure prediction methods such as AlphaFold.[8] The pyDockTET method, not available for comparison, re-ranks the assembled domain structures by ZDOCK[9] based the scoring function of pyDock[10] with an additional term related to linker length. Rosetta and AIDA, compared in this paper, are both *ab initio* domain–domain docking methods that rescore the resulting docking poses generated by a protein–protein docking method. Web servers for these methods are not available anymore. Rosetta employs a sampling method used for small protein chains for linker modeling and scores docking poses by Rosetta interaction energy. AIDA rescores domain poses obtained by sampling linker conformations based on a scoring function which considers protein–protein interaction and the stability of the linker conformation.

Here, we introduce a new domain–domain docking web server called GalaxyDomDock, which exhibits increased performance on four benchmark sets, including a set comprising the domain structures predicted by the most recent version of AlphaFold.[8]

The proposed GalaxyDomDock server produces possible docking orientations from the given structures of two domains and the sequence of the connecting linker(s). GalaxyDomDock is also based on a protein–protein docking, but in an approach that differs from Rosetta or AIDA. First, a more recent protein–protein docking method, called GalaxyTongDock,[11] is adopted. Second, domain orientations generated by GalaxyTongDock are subject to an effective filtering process that involves the Dijkstra algorithm to screen out geometrically infeasible orientations for the given linker(s). Finally, for the top 5 or 50 models (as chosen by the user), linker conformations are modeled by adopting a loop sampling method called fragment assembly with loop closure (FALC).[12] The GalaxyDomDock web server is freely available as a part of GalaxyWEB[13–14] at http://galaxy.seok-lab.org/domdock.

Results and discussion

The performance of GalaxyDomDock was compared with those of available *ab initio* docking programs, on four benchmark sets described in the "Materials and Methods" section. Three sets, Rosetta-2dom, AIDA-2dom, and AIDA-2dis, comprise two-domain proteins with the known crystal domain structures, while the remaining set, AIDA-AF, comprises two-domain proteins with domain structures predicted by AlphaFold.[8] The AIDA-2dis set comprises two-domain proteins connected by two linkers, so one of the domains is discontinuous in sequence. All other sets comprise two domain proteins connected by a single linker.

Assembly of the known domain structures

The authors of Ref. 7 compared the performance of Rosetta and AIDA on the Rosetta-2dom set by evaluating top 5 models selected from a total of 5000 models, which were generated by protein–protein docking for each protein target. The performance of GalaxyDomDock was evaluated on the same set with the same evaluation measure. The evaluation measure for this set, called "Success_Rate_RMSD" in this paper, is the proportion of proteins in which at least one of the top 5 structures has a Cα RMSD below 3 Å, after the superposition of the predicted domain–domain docking pose to the crystal 2-domain structure. The linker residues were excluded from the RMSD calculation, following Refs. 6 and 7. GalaxyDomDock exhibited a success rate of 80.3%, which was higher than those of Rosetta (50.0%) and AIDA (68.4%), as presented in Table 1**(a)**. The success rate based on ligand RMSD (RMSD calculated on smaller one of the domains when the other is superimposed onto the crystal structure) is 79.8% for GalaxyDomDock. Those for the other methods are not available.

Evaluations of the results on the AIDA sets were in accordance with that in Ref. 7, in which a different measure, called "Success_Rate_TM" in this paper, was adopted. This measure considers the fact that a high structural similarity of a docking pose originates from two contributions, one from the accuracy of the individual domain structures and the other from the accuracy of the domain orientation. Success_Rate_TM is defined as the proportion of proteins in which at least one of the top 5 structures has a TM score[15] relative to the crystal structure above the threshold value $TM^{cut-off}$. TM score is a similarity measure ranging

Table 1 Performance comparison of GalaxyDomDock with other methods on four sets.

	GalaxyDomDock	AIDA	Rosetta
(a) Success_Rate_RMSD on Rosetta-2dom set (76 targets)	80.3[c] (78.9[d])	68.4[b]	50.0[a]
(b) Success_Rate_TM on AIDA-2dom set (136 targets)	76.5[c]	53.7[b]	-
(c) Success_Rate_TM on AIDA-2dis set (20 targets)	75.0[c]	70.0[b]	-
(d) Success_Rate_TM on AIDA-AF set (136 targets)	57.4[c]	33.8[c]	-

[a] Data obtained from Ref. 6.
[b] Data obtained from Ref. 7.
[c] Data generated in this study.
[d] Success rate based on ligand RMSD generated in this study.

from 0 to 1 for the proteins of the same length. The threshold value is defined considering the domain structure accuracy as

$$TM^{cut-off} = Dom\_TM^{max} + 0.5 \times \left(\sum_{i=1}^{m} Dom\_TM^{i} - Dom\_TM^{max} \right),$$

where Dom_TM^i refers to the TM score of the *i*th domain (or split domain if two linkers connect two domains) structure relative to the full crystal structure whose maximum value is the ratio of the number of the domain residues to the number of the full protein residues, Dom_TM^{max} is the maximum value among Dom_TM^i, and m is 2 (one-linker case) or 3 (two-linker case). Dom_TM^{max} and $\sum_{i=1}^{m} Dom\_TM^i$ correspond to the minimum and maximum possible TM scores of a docked pose, respectively.

The performance of AIDA on the AIDA sets were evaluated on the top 5 structures selected from 50 models generated for each protein, in accordance with Ref. 7. On both the AIDA-2dom set, comprising two-domain proteins with a linker, and the AIDA-2dis set, comprising two-domain proteins connected by two linkers, the proposed GalaxyDomDock exhibited higher success rates than AIDA (76.5% versus 53.7% for the AIDA-2dom set and 75.0% versus 70.0% for the AIDA-2dis set), as presented in Tables 1**(b)** and **(c)**. Comparison with the Rosetta method was not performed because the Rosetta protocol or web server is not available to our understanding.

Assembly of the domain structures predicted by AlphaFold

For the performance test of the domain assembly cases in which only predicted domain structures are available, the individual domain structures of the proteins in the AIDA-2dom set were predicted by AlphaFold.[8] This new set is named AIDA-AF. The docking performance is evaluated in terms of Success_Rate_TM, in accordance with Ref. 7. The top 5 models of AIDA were generated by running the AIDA program on local computers by randomly generating a total of 50 models for each protein. GalaxyDomDock exhibits a better performance with 57.4% than the 33.8% obtained using AIDA, as presented in Table 1**(d)**. Performances of both GalaxyDomDock and AIDA on this predicted domain structure set are reduced by about 20%, compared to those on the crystal domain structure set for the same proteins in Table 1**(b)**. This result implies that further method development is necessary so that induced conformational changes of domains upon domain interactions are considered during domain–domain docking.

GalaxyDomDock server

The GalaxyDomDock code comprises GalaxyTongDock, which is written in Fortran 90, and its overall procedure is implemented using Python. The server runs on a cluster of 8 Linux servers of 2.20 GHz Intel Xeon 24-core processors. The web application adopts the Python programming language and MySQL database. In addition, the predicted models are visualized using the JavaScript Protein Viewer (http://biasmv.github.io/pv/).

The GalaxyDomDock server is designed to perform in two cases: one-linker case (two domains and a linker) and two-linker case (two domains and two linkers).

The required input information includes the entire protein sequence, two domain structures (either experimentally resolved or predicted), and sequence regions of the two domains and the linker(s). The entire protein sequence must contain the parts corresponding to the two domains and the linker(s). For computational efficiency, each domain structure, provided in PDB format, is restricted to retain no more than 600 residues. Users can choose whether linker structures are modeled for the top 5 or 50 domain orientations. The average run time for a single job is approximately 3 h and 1–2 d if linker sampling is performed for the top 5 and 50 domain orientations, respectively.

Users can also specify residues to be preferred at the domain interface and those to be avoided from the interface by utilizing the interface and block options, respectively. The top 5 models with and without the linkers are illustrated on the report page. Users can download the top 50 models

without the linkers and the top 5 or 50 models with linkers. The GalaxyDomDock scores of the top 5 models are provided in the summary table on the report page.

Application examples of GalaxyDomDock

Among the crystal structures of multi-domain proteins released after the publication of the recent version of AlphaFold,[8] we identified a case [illustrated in Figure 1**(a)**, PDB ID: 6ZEP, chain A] in which the GalaxyDomDock run with the domain structures predicted by AlphaFold was more accurate than the AlphaFold run for the entire protein chain. The protein was split into two domains using an in-house modeling-unit prediction algorithm called GalaxyDom (http://galaxy.seoklab.org/cgi-bin/submit.cgi?type=DOM), which identifies domains to be predicted individually based on multiple sequence alignment of related proteins and the availability of template structures. AlphaFold was run by setting the date of the template database on the date of the training database.

In this case, the TM-score of the five AlphaFold models were 0.8564, 0.8554, 0.8557, 0.8572, and 0.8574, which do not exceed the TM-score threshold of 0.88725. In contrast, the TM score of the model with the second highest GalaxyDomDock score was 0.9068, exceeding the TM threshold score. This example demonstrates that an *ab initio* domain–domain docking program can be beneficial when the accurate modeling of individual domains is possible, but the domain orientation is difficult to predict, owing to the limited sequence/structure information of related proteins on domain orientations.

In Figure 1**(b)**, another example is shown in which AlphaFold predicted the domain orientation of a multidomain protein only roughly, while GalaxyDomDock did not generate any successful orientation. This does not imply failure of AlphaFold because it is not known yet whether the observed domain orientation in the crystal is not an artifact of the crystal environment.

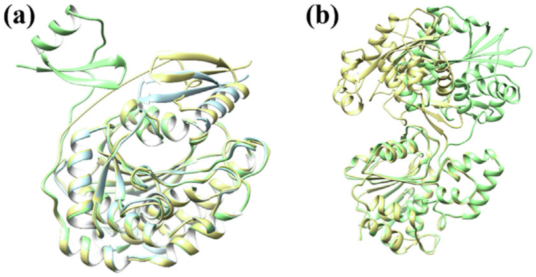

Figure 1. (a) Case (PDB ID: 6ZEP, chain A) in which GalaxyDomDock is more successful than AlphaFold in predicting domain orientation observed in the crystal structure. The crystal structure (yellow), the first model of AlphaFold of the full protein (green), and the second model of GalaxyDomDock generated by docking AlphaFold domain structures (blue). (b) Another example (PDB ID: 6Z6D, chain A) where the AlphaFold model exhibits a relatively unsuccessful domain orientation (green) compared to the crystal structure (yellow). No successful domain orientation was obtained with GalaxyDomDock for this protein.

Conclusion

The GalaxyDomDock web server proposed in this study provides an ensemble of domain orientations given structures for two domains and the linker sequence(s). The server exhibited enhanced performance compared to existing *ab initio* domain–domain docking programs. An interesting case in which the server generated a more accurate domain orientation than AlphaFold was also identified. Based on these results, the proposed *ab initio* server could be beneficial role in predicting domain orientations when they are difficult to predict via current information-based methods. It could be further inferred that the GalaxyDomDock may provide useful hypotheses on multiple functional states involving different domain orientations.

Materials and methods

Benchmark sets

Four benchmark sets are employed in this paper. First, the Rosetta benchmark set,[6] named "Rosetta-2dom," is used for simultaneous comparisons with both AIDA and Rosetta. This set comprises 76 two-domain proteins, and each domain structure is obtained from the crystal structure of the two-domain protein. For a separate comparison with AIDA, we adopted the AIDA benchmark set,[7] which comprises 136 two-domain proteins, named "AIDA-2dom" set, and 20 two-domain proteins in which one of the domains is connected to the other by two linkers and exhibits discontinuity in sequence, named "AIDA-2dis" set. The remaining part of the AIDA set, called two-unbound set in Ref. 7, was excluded here because of challenges involving domain structures with insufficient secondary structures and ill-defined domain linkers. Another benchmark set of 136 two-domain targets of the AIDA benchmark set, called "AIDA-AF," was prepared by predicting the domain structures using AlphaFold. The AlphaFold source code (https://github.com/deepmind/alphafold) was compiled and run in-house with the default options, and only the first models were adopted.

Protein–protein docking

The default options of the protein–protein docking program, GalaxyTongDock, was adopted to generate domain–domain docking poses, roughly considering the linker region(s) by excluding geometrically infeasible docking poses in which the C_a distance between the two anchor residues, to which the two end residues of the linker are connected, is longer than the maximum possible linker length, $L_{\max} = (\text{numberoflinkerresidues} + 1) \times 3.4$. In the case of the proteins with two linkers, this step is performed for each linker. A more sophisticated restraint considering the specific geometry that the linker(s) has to pass is applied in the next step, as explained in the next subsection.

During docking, user-defined residues can be prioritized at the domain interface (when the interface option is adopted) or avoided from the domain interface (when the block option is adopted). Poses with preferred interfacial residues within 8 Å from any residue of the partner domain are assigned as high priority during docking. Conversely, poses with specified block residues within 8 Å from any residue of the partner domain are assigned a very high penalty.

A maximum of the top 1000 remaining poses were clustered to eliminate redundancy, as in GalaxyTongDock, and they are subject to geometric filtering as described in the next section.

Geometric filtering

This filtering process utilizes the Dijkstra's algorithm[16] to determine the minimum distance between the C_a atoms of the two anchor residues, considering the possible paths that do not clash with the protein atoms that form steric barriers. The algorithm is based on the Taxicab geometry which considers the possible paths connecting the three-dimensional lattice points in the presence of geometrical barriers. The algorithm introduced in Ref. 16 was implemented in-house, and the relevant parameters are defined as follows. First, lattice points are generated within the sphere of the diameter $(2 \times [L_{\max}] + 1)$ centered at the C_a atom of the anchor residue belonging to the first domain with separations of 1 Å in each of the three dimensions. Next, the shortest path between the two anchor residues in the presence of steric barriers is determined via the Dijkstra's algorithm. The starting point of the path is set to the center gird, and the ending point is defined as the point located within the vdW radius of carbon from the C_a atom of the anchor residue belonging to the second domain. Any grid point within the range of the steric barriers is excluded during the search for the shortest path. The grid points that trigger steric clashes are defined as those within the sum of the vdW radii of the closest atom to the grid point and nitrogen (the smallest among the backbone heavy atoms). Paths compromising the segments that connect only the grid points within $\sqrt{6}$ Å are considered. The shortest distance in Taxicab geometry is the distance with the minimum value for the sum of the segment distances between the grid points it passes through.

Docking poses whose minimum distance in Taxicab geometry between the anchor residues is longer than $L_{\max}$ are filtered out. Regarding two-domain proteins connected by two linkers, this process is conducted for each of the two linkers.

Linker modeling

After geometric filtering, for each of the top 5 or 50 domain orientations, as selected by the user, the linker conformation is modeled by adopting a loop sampling method FALC that performs analytical loop closure[17] to sample six backbone torsion degrees of freedom and fragment assembly to sample the remaining degrees of freedom[12]. FALC is run by setting the maximum number of generated linker conformations to 500, and the linker conformation with the lowest GalaxyLoop-PS2 energy[18] is selected. Users may use a separate loop modeling server, GalaxyLoop,[19] to predict linker structures more accurately with more computational cost.

CRediT authorship contribution statement

Jayun Choi: Validation, Investigation, Writing – original draft. **Taeyong Park:** Conceptualization, Software. **Seung Yul Lee:** Software, Methodology. **Jinsol Yang:** Software. **Chaok Seok:** Supervision, Writing – review & editing, Funding acquisition.

Acknowledgements

This work was supported by the National Research Foundation of Korea (NRF) grant (No. 2020M3A9G7103933) and Institute of Information & communications Technology Planning & Evaluation (IITP) grant (No. 2021-0-02068, Artificial Intelligence Innovation Hub) funded by the Korea government (MSIT).

Declaration of Competing Interest

The authors declare that they have no known competing financial interests or personal relationships that could have appeared to influence the work reported in this paper.

Received 29 November 2021;
Accepted 16 February 2022;
Available online 22 February 2022

Keywords:
domain assembly;
domain–domain orientation prediction;
ensemble of domain–domain conformations;
multiple conformational states;
linker modeling

† These authors contributed equally to this paper.

References

1. Seok, C., Baek, M., Steinegger, M., Park, H., Lee, G.R., Won, J., (2021). Accurate protein structure prediction: what comes next? *Biodesign* **9** (3), 47–50.
2. Uchikawa, E., Chen, Z., Xiao, G.Y., Zhang, X., Bai, X.C., (2021). Structural basis of the activation of c-MET receptor. *Nat. Commun.* **12** (1), 4074.
3. Latorraca, N.R., Venkatakrishnan, A.J., Dror, R.O., (2017). GPCR dynamics: structures in motion. *Chem. Rev.* **117** (1), 139–155.
4. Zhou, X., Hu, J., Zhang, C., Zhang, G., Zhang, Y., (2019). Assembling multidomain protein structures through analogous global structural alignments. *Proc. Natl. Acad. Sci. U. S. A.* **116** (32), 15930–15938.
5. Cheng, T.M., Blundell, T.L., Fernandez-Recio, J., (2008). Structural assembly of two-domain proteins by rigid-body docking. *BMC Bioinformatics* **9**, 441.
6. Wollacott, A.M., Zanghellini, A., Murphy, P., Baker, D., (2007). Prediction of structures of multidomain proteins from structures of the individual domains. *Protein Sci.: A Publ. Protein Soc.* **16** (2), 165–175.
7. Xu, D., Jaroszewski, L., Li, Z., Godzik, A., (2015). AIDA: ab initio domain assembly for automated multi-domain protein structure prediction and domain-domain interaction prediction. *Bioinformatics (Oxford, England)* **31** (13), 2098–2105.
8. Jumper, J., Evans, R., Pritzel, A., Green, T., Figurnov, M., Ronneberger, O., Tunyasuvunakool, K., Bates, R., Žídek, A., Potapenko, A., Bridgland, A., Meyer, C., Kohl, S., Ballard, A.J., Cowie, A., Romera-Paredes, B., Nikolov, S., Jain, R., Adler, J., Back, T., Hassabis, D., (2021). Highly accurate protein structure prediction with AlphaFold. *Nature* **596** (7873), 583–589.
9. Chen, R., Li, L., Weng, Z., (2003). ZDOCK: an initial-stage protein-docking algorithm. *Proteins* **52** (1), 80–87.
10. Cheng, T.M., Blundell, T.L., Fernandez-Recio, J., (2007). pyDock: electrostatics and desolvation for effective scoring of rigid-body protein-protein docking. *Proteins* **68** (2), 503–515.
11. Park, T., Baek, M., Lee, H., Seok, C., (2019). GalaxyTongDock: Symmetric and asymmetric ab initio protein-protein docking web server with improved energy parameters. *J. Comput. Chem.* **40** (27), 2413–2417.
12. Lee, J., Lee, D., Park, H., Coutsias, E.A., Seok, C., (2010). Protein loop modeling by using fragment assembly and analytical loop closure. *Proteins* **78** (16), 3428–3436.
13. Shin, W.H., Lee, G.R., Heo, L., Lee, H., Seok, C., (2014). Prediction of protein structure and interaction by GALAXY protein modeling programs. *BIODESIGN* **2** (1), 1–11.
14. Ko, J., Park, H., Heo, L., Seok, C., (2012). GalaxyWEB server for protein structure prediction and refinement. *Nucl. Acids Res.* **40(Web Server issue)**, W294–W297.
15. Zhang, Y., Skolnick, J., (2004). Scoring function for automated assessment of protein structure template quality. *Proteins* **57** (4), 702–710.
16. Dial, R.B., (1969). Algorithm 360: Shortest-path forest with topological ordering [H]. *Commun. ACM* **12** (11), 632–633.
17. Coutsias, E.A., Seok, C., Jacobson, M.P., Dill, K.A., (2004). A kinematic view of loop closure. *J. Comput. Chem.* **25** (4), 510–528.
18. Park, H., Lee, G.R., Heo, L., Seok, C., (2014). Protein loop modeling using a new hybrid energy function and its application to modeling in inaccurate structural environments. *PloS One* **9**, (11) e113811
19. Park, H., Seok, C., (2012). Refinement of unreliable local regions in template-based protein models. *Proteins* **80** (8), 1974–1986.
