# Supplementary material for: PDBx/mmCIF Ecosystem: Foundational Semantic Tools for Structural Biology
Source: J Mol Biol. Author manuscript; Available in PMC 2023 Jun 26. (PMC10292674; doi:10.1016/j.jmb.2022.167599)
Supplement: Article [file NIHMS1907597-supplement-Article.zip › MLCPP-2-0--An-Updated-Cell-penetrating-Peptides-and-T_2022_Journal-of-Molecu.pdf]

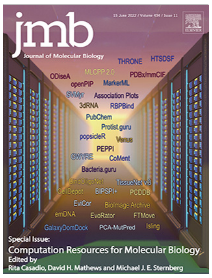

MLCPP 2.0: An Updated Cell-penetrating Peptides and Their Uptake Efficiency Predictor

Balachandran Manavalan [1]* and Mahesh Chandra Patra [2]

*1 - **Computational Biology and Bioinformatics Lab,** Department of Integrative Biotechnology, College of Biotechnology & Bioengineering, Sungkyunkwan University, Seobu-ro, Jangan-gu, Suwon-si, Gyeonggi-do 16419, Republic of Korea*
*2 - **Arontier Co.,** 241 Gangnam-daero, Seocho-gu, Seoul 06735, Republic of Korea*

Correspondence to Balachandran Manavalan: *bala2022@skku.edu (B. Manavalan), @bala_CBB (B. Manavalan).*
https://doi.org/10.1016/j.jmb.2022.167604
Edited by Rita Casadio

Abstract

Cell-penetrating peptides (CPPs) translocate into the cell as various biologically active conjugates and possess numerous biomedical applications. Several machine learning-based predictors have been proposed in the past, but they mostly focus on identifying only CPPs. We proposed a two-layered predictor in 2018 in order to predict CPPs and their uptake efficiency simultaneously. While MLCPP has gained widespread access to research, further improvements are needed to enhance its practical application. A new version of MLCPP is presented in this study called MLCPP 2.0, an interpretable stacking model that identifies CPPs and their strength of uptake efficiency. We updated the benchmarking dataset, explored 17 different sequence-based feature encoding algorithms, and used seven different conventional machine learning classifiers. With multiple 10-fold cross-validation, we constructed 119 baseline models whose predicted probability values were merged and treated as a new feature vector. In a systematic way, a feature set and a classifier are identified that are optimal for predicting the CPP and uptake efficiency separately. The MLCPP 2.0 model achieved outstanding performance on the independent test set, significantly outperforming the existing state-of-the-art predictors. Hence, we expect that our proposed MLCPP 2.0 will facilitate the design of hypothesis-driven experiments by enabling the discovery of novel CPPs. MLCPP 2.0 is freely accessible at https://balalab-skku.org/mlcpp2/.

© 2022 Elsevier Ltd. All rights reserved.

Introduction

The effective delivery of a pharmaceutical product to its target site of interest is essential in drug development for minimizing off-target effects and improving patient compliance. To this end, cell-penetrating peptides (CPPs) have emerged as a critical factor that has shaped the contemporary drug delivery paradigms, demonstrating numerous clinical successes.[1] CPPs are short (5–30 amino acids), cationic, amphipathic or hydrophobic peptides that can penetrate the cell membrane through an energy–dependent or –independent manner.[2] CPPs can deliver a wide variety of therapeutic cargos such as small molecule drugs,[3] peptides,[4] proteins,[5] nucleic acids,[6] nanoparticles and liposomes[7] to their site of action. In recent years, CPP-based drug delivery systems have undergone considerable progress in various preclinical disease models and in clinical trials.[8] Thus, CPPs or chemically modified analogs represent an effective therapeutic avenue for delivering drug molecules to the desired subcellular targets. With the advent of next-generation proteomics technologies, sequencing of peptide or protein molecules has become less cumbersome; however, identification and characterization of specific CPPs with good uptake efficiency through conventional in vitro assays are yet

0022-2836/© 2022 Elsevier Ltd. All rights reserved.

time-consuming and labor-intensive.[9] It is evident that the translocation of a peptide through membrane lipids is largely governed by its amino acid sequence and intrinsic physiochemical properties. Therefore, the development of computational methods is a rational choice to accurately identify prospective CPPs, thereby reducing the experimental burden on researchers. Recently, Agarwal et al. collected the experimentally characterized CPPs and compiled them in the CPPsite 2.0 (URL: https://crdd.osdd.net/raghava/cppsite/) database, where the majority were derived from natural sources.[10] Peptide sequences in CPPsite 2.0 are listed with multiple information, including their nature and origin, subcellular localization, uptake mechanism and efficiency, chemical modifications, and physicochemical properties. CPPs have been validated on various cell types, such as HeLa, Chinese hamster ovary, Human bowe's melanoma, Jurkat, EBTr and NIN-3T3 cells.[11,12] Notably, comparing CPPs′ uptake efficiency to their positive control, uptake efficiency has been classified into Low (<25%); medium (between 26% and 75%); and high (>75%). This database acted as the primary source for the development of various prediction models.

Few statistical methods have been proposed for the prediction of CPPs before 2010.[13,14] Thereafter, several publicly accessible machine-learning (ML)-based CPP models have been proposed: CPPpred,[15] CellPPD,[16] C2Pred,[17] SkipCPP-Pred,[18] CPPred-RF,[19] MLCPP,[20] KLEM-CPPpred,[21] StackCPPred,[22] and, and BChemRF-CPPred.[23] In recent studies,[24,25] a detailed description of the existing CPP prediction methods has been presented in terms of algorithms, feature encodings, and evaluation strategies. Three methods (CPPred-RF, StackCPPred, and MLCPP) predict CPP and uptake efficiencies simultaneously. MLCPP is our previous method developed in 2018, using 854 samples, four different feature encodings, and two different machine learning (ML) classifiers. MLCPP performed excellently in two independent validation studies[24,25] and was heavily accessed in the research community. In spite of MLCPP's good performance, MLCPP should be updated by utilizing a larger training dataset and a variety of sequence-derived features and conventional ML classifiers.

The overall framework of MLCPP 2.0 is illustrated in Figure 1. MLCPP 2.0 is distinguishable from MLCPP in the following aspects: a larger training dataset size, additional 12 feature encodings, four additional ML classifiers, and a stacking strategy for constructing the model. MLCPP 2.0 uses a two-layer prediction framework, in which the first layer predicts whether a given peptide belongs to CPP, while the second layer predicts if the predicted CPP will have a low or high uptake efficiency. Based on empirical results of benchmarking tests, MLCPP 2.0 offers better prediction accuracy than existing state-of-the-art methods. Furthermore, we employed the Shapley Additive Explanation Algorithm to better understand MLCPP 2.0′s superior performance on both layers.

Materials and Methods

Dataset construction

Due to the disparate nature of the training datasets used by the existing methods, we combined these samples to generate a reliable training dataset. Therefore, we downloaded training datasets from the following six methods: C2Pred, CellPPD, CPPred-RF, KELM-CPPpred, MLCPP, and BChemRF-CPPred. Firstly, the positive and negative samples (CPPs and non-CPPs) were grouped independently. Then, CD-HIT[26] was applied on positive samples and excluded CPPs that shared more than 85.0% sequence identity with other CPPs, resulting in 573 CPPs. Similarly, we excluded non-CPPs which share >85.0% sequence identity with other non-CPPs or CPPs, resulting in a large number of non-CPPs. For the model training, we randomly selected 573 non-CPPs to balance the CPPs. Henceforth, we refer to this dataset as the layer1 training dataset.

It is crucial to check the robustness of the trained model. Among the six methods mentioned above, only four methods (CellPPD,KELM-CPPpred, MLCPP, and BChemRF-CPPred) were tested with independent datasets. We first downloaded the CPPs from these methods and supplemented them with the CPPs from CPPsite 2.0 and Basith et al. CPP methods evaluation.[24] Consequently, the resultant sequences that share a high similarity with the training dataset were excluded. With a CD-HIT cutoff of 85.0% (as applied for training) on independent dataset resulted in 60 CPPs. Based on these small CPPs, it would be difficult to draw a statistically significant difference between the two methods. Therefore, we applied a slightly higher threshold (90.0%) and obtained 157 CPPs. Similarly, non-CPPs collected from the existing methods and excluded the sequences that shared above 70% sequence identity with the training samples, resulting in 2184 non-CPPs. Henceforth, we refer to this dataset as the layer1 independent dataset.

CPPs with high and low uptake efficiency were collected from MLCPP dataset and CPPsite 2.0. Redundant sequences were discarded by applying a CD-HIT threshold of 0.99, resulting in 186 CPPs with low uptake efficiency and 156 with high uptake efficiency. This high threshold of sequence identity was mandatory due to the small dataset size. Finally, 140 peptides with low and 140 peptides with high uptake efficiency were randomly selected from the original dataset for model training, and the remaining peptides (46 peptides with low uptake efficiency and 16 with

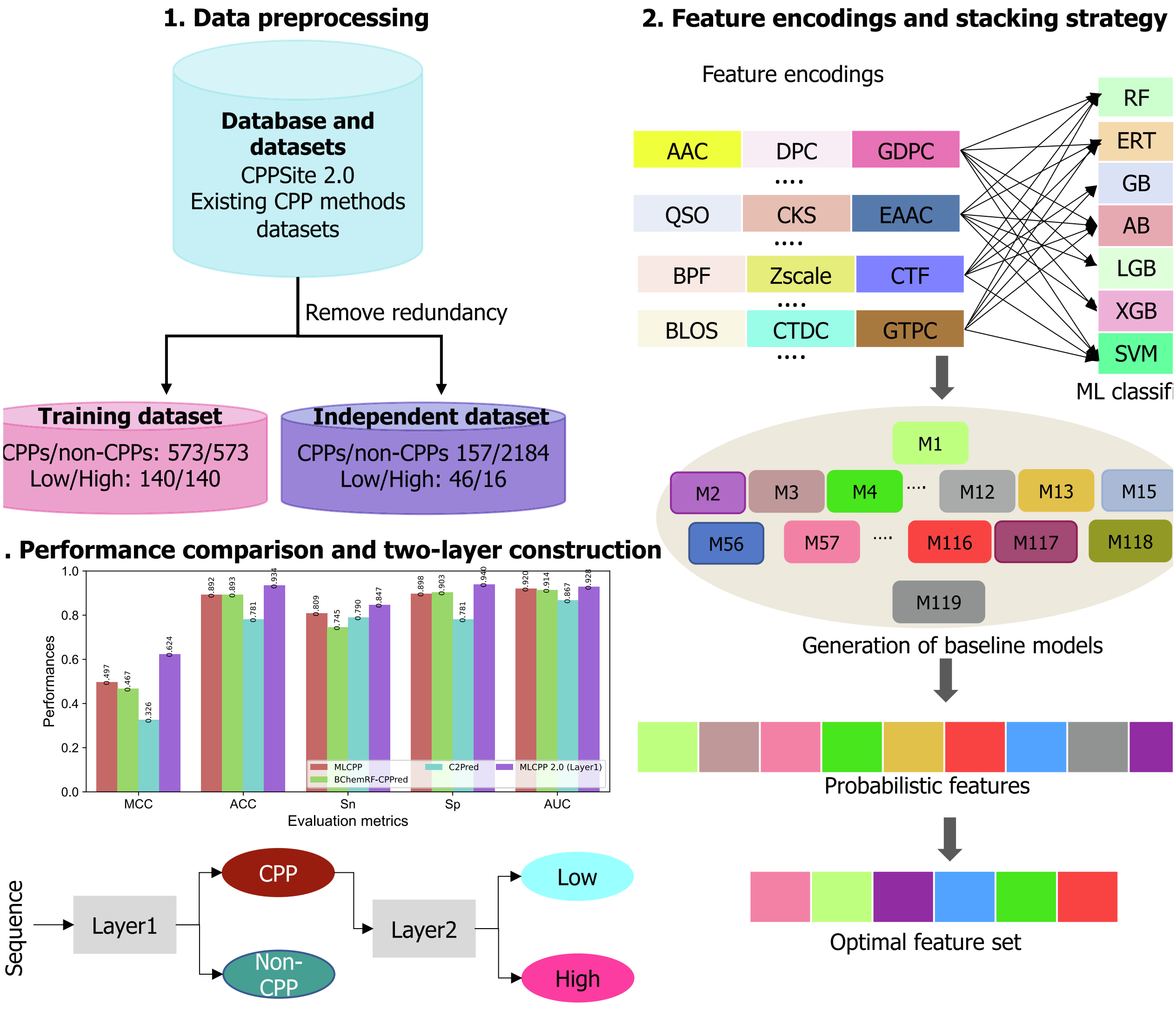

Figure 1. An Overview of MLCPP 2.0 framework for predicting CPP and their uptake efficiency. Schematic display of the three stages in the construction of MLCPP 2.0 is shown.

high uptake efficiency) for testing model's transferability. This dataset is called layer2.

Stacking ensemble learning framework of MLCPP 2.0

MLCPP 2.0 is a meta-learning framework that aims to improve prediction of CPPs and their uptake efficiency. A stacking approach allows the integration of information from multiple classifiers to build a robust prediction model. In recent years, this approach has been applied successfully to a number of bioinformatics and computational biology studies.[27–30] As a first step, we comprehensively evaluated the performance of 17 different types of feature encoding with the seven commonly used ML classifiers individually on layer1 and layer2 datasets. Overall, we generated 119 baseline models (17 encodings × 7 ML classifiers) for each layer. The employed ML classifiers are support vector machine (SVM), random forest (RF), AdaBoosting (AB), light gradient boosting (LGB), gradient boosting (GB), eXtreme GB (XGB), and extremely randomized tree (ERT). Feature encoding algorithms are dipeptide composition (DPC), amino acid composition (AAC), composition transition and distribution (CTDC, CTDT, and CTDD), grouped DPC (GDPC), grouped tripeptide composition (GTPC), enhanced AAC (EAAC), binary profile (BPF), enhanced grouped AAC (EGAAC), K-spaced conjoint triad (KSC) composition of k-spaced amino acid pairs (CKS), composition of k-spaced amino acid group pairs (CKSAAGP), dipeptide deviation from expected mean (DDE), BLOSUM62 (BLOS), Zscale, and quasi sequence order (QSO).[31,32] The supplementary information provides details on each feature encoding, cross-validation procedure, and performance evaluation. The ML classifiers were assessed by using a 10-fold cross-validation strategy multiple times for each feature set for the

purposes of determining the optimal parameter. As the second step, we ranked these baseline models according to the Matthews correlation coefficient (MCC), and generated eleven sets, each with an increment of 10 models. Using the predicted probability scores of CPPs (layer1) or low uptake efficiency (layer2), seven different classifiers were evaluated and determined the best classifier. Finally, we selected the best model and the corresponding optimal feature set for layer1 and layer2 independently based on the comparison of eleven different sets of features.

Results and Discussion

Construction of MLCPP 2.0 using the training dataset

(i) Layer1 model

We compared the CPP prediction performance of individual encodings against seven different classifiers, whose results are summarized in Tables S1–S7. Results in terms of MCC are given in Figure 2(A), where the QSO encoding is shown to have excellent discriminative capacity when compared to 17 encodings for four classifiers (ERT, AB, XGB, and LGB). As for the remaining GB and SVM, CKSAAP encoding produced the best results, while AAC produced the best results for RF. However, EGAAC produced the lowest results regardless of the classifier used. Rather than selecting the best model from 119 baselines, a stacking strategy was used to develop a more robust predictor. First, the predicted probabilities of CPPs from the 119 baseline models were gathered and considered a novel probabilistic feature (PF) vector. After that, we ranked the PF according to their MCC performance based on their baseline model performance and divided them into eleven groups. Basically, we incremented 10D PF at every group, from the first to the 11th group. Each of these 11 groups then input seven different classifiers again and developed their respective models. The performance of the seven classifiers for each group is shown in Table S8, and the selected final model is in bold. A SVM generated superior performance in eight groups, while three classifiers (AB, ERT, and XGB) contributed one to the remaining three groups.

On top of these eleven groups-based meta-models, we developed individual meta-models for each classifier that took its own PFs into account. For example, the SVM-based meta-model uses only 17 PFs based on SVM and 17 encodings. The performance of seven classifier meta-models is provided in Table S9. Figure 2(B) shows the performance comparison between meta-models, demonstrating that the meta-model using mixed classifier PF showed an improved performance compared to individual classifier PF. Overall, top 80 features displayed the best performance among the 11 group models with MCC, accuracy (ACC), sensitivity (Sn), specificity (Sp), and area under the ROC curve (AUC) of 0.827, 0.913, 0.885, 0.941, 0.949, respectively. The distribution of the 80D optimal PF set shown in Figure 2(C) shows 13/17 feature encodings contributing to the

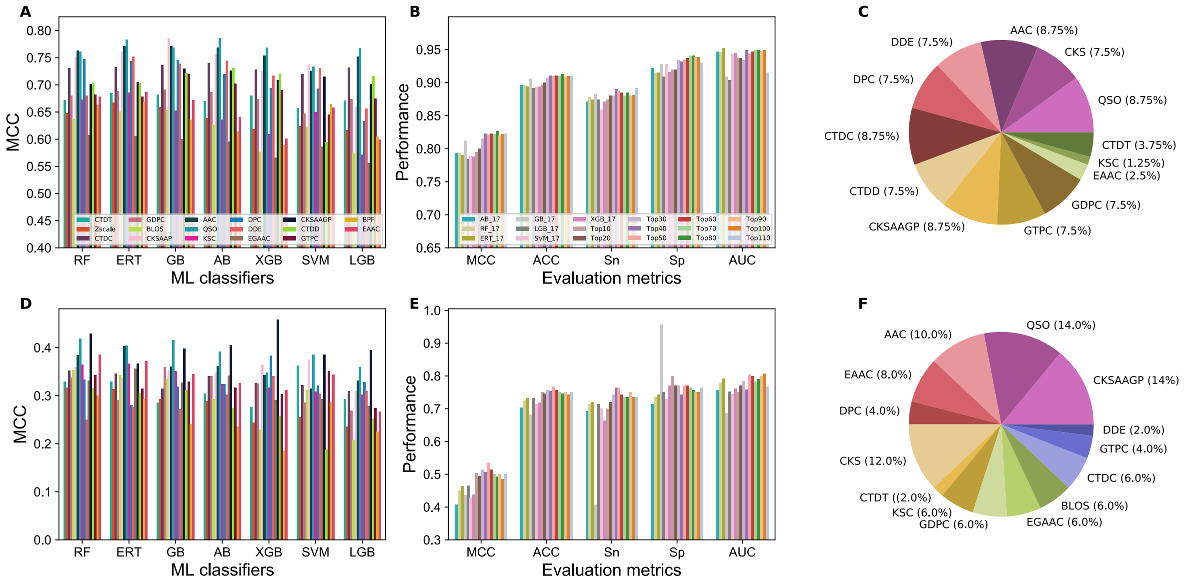

Figure 2. Performance comparison of different models on Layer1 and Layer2 training datasets. (A) CPP prediction performance of 119 baseline models, (B) Meta-model performance in CPP prediction, (C) Analysis of the distribution of the optimal probabilistic feature set for CPP prediction, (D) Uptake efficiency prediction performance of 119 baseline models, (E) Meta-model performance in uptake efficiency prediction, and (F) Analysis of the distribution of the optimal probabilistic feature set for uptake efficiency prediction.

80D set, but their respective shares of the contribution varied. Ultimately, this model was selected for layer1 prediction in MLCPP 2.0.

(ii) Layer2 model

The individual encoding capability was also tested with respect to seven different classifiers for identifying low versus high uptake efficiency CPPs (Tables S1–S7). According to Figure 2(D), CKSAAGP encoding is the best performer among five different classifiers (RF, AB, XGB, SVM, and LGB), and QSO encoding is the best performer among ERT and GB. In layer1, we did not find any stand out models; however, we did find one model (XGB-CKSAAGP) that performed significantly better among 119 baseline models. Our strategy took into account all available models rather than selecting the best.

Similar to layer1, we evaluated 11 different groups with respect to seven classifiers. As we can see from Table S8, during the construction of the meta-model, the performance of each classifier varies with the feature set, showing the need to explore different classifiers rather than rely exclusively on particular classifier as employed in previous studies.[33] A performance comparison is shown in Figure 2(E) between the best meta-models from 11 groups and the meta-models for seven classifiers. Results showed that the top 50 feature set achieved the highest performance, which was marginally better than the other 10 groups and significantly better than individual classifier-based meta-models. In particular, the top 50 optimal feature set and the GB achieved MCC, ACC, Sn, Sp, and AUC of 0.536, 0.768, 0.764, 0.771, and 0.804. As a result, this model was selected for layer2 prediction in MLCPP 2.0. It is noteworthy that 14 of 17 feature encodings contributed to the final prediction, but their contributions varied, as shown in Figure 2(F). Most importantly, the individual encoding contributions of layers 1 and 2 differ significantly.

Comparison of MLCPP 2.0 with the top five baseline models based on the training dataset

MLCPP 2.0 showed notable improvement with 4.1–5.6% in MCC and 2.03–2.93% in ACC on layer1 (Figure S1(A)), and 7.8–13.0% in MCC, and 3.94–6.56% in ACC on layer2 (Figure S1(B)) when compared to the top five baseline models. The improvement can be attributed to our systematic approach employed in each step of the stacking process. MLCPP 2.0 cross-validation performances cannot be directly compared with the previous version (MLCPP) due to the differences in training datasets. To get an overview between two models, we compared the MLCPP 2.0 with the MLCPP and the result shows that there is a notable improvement on layer1, with an increase in MCC of 5.8% and an increase in ACC of 2.9%. On layer2, the updated model showed an increase of 9.1% in MCC and a 4.3% increment in ACC, indicating the effectiveness of the proposed stacking approach.

Evaluation of the MLCPP 2.0 and the existing predictors on an independent dataset

Several CPP prediction methods have been reported, but only a few are publicly available (CellPPD, C2Pred, MLCPP, and BChemRF-CPPred) on a web server, which is working with the given sequence. Using an independent dataset (layer1), we evaluated these four methods as well as the newly proposed method (Figure 3(A)). MLCPP 2.0 had superior performance with MCC, ACC, Sn, Sp, and AUC of 0.624, 0.934, 0.847, 0.940, and 0.928, respectively. In MCC, improvements ranged from 12.7% to 29.8% while in ACC, they ranged from 4.1% to 15.3%. McNemar's Chi-square test[34] was used to compute a statistical significance between MLCPP 2.0 and other methods. The results show that MLCPP 2.0 is significantly superior to the existing methods (Table S10). This demonstrates that systematic approaches, integrating multiple encodings and classifiers, as well as stacking strategies, are the main factors causing improved performance.

Currently, no method allows one to compute low/high uptake efficiency based on a given peptide. Consequently, MLCPP 2.0 cannot be compared with the existing methods. Therefore, we compared with the seven classifier-based meta-models. As shown in Figure 3(B), MLCPP 2.0 achieved the best performance with MCC, ACC, Sn, Sp, and AUC of 0.354, 0.677, 0.652, 0.750, and 0.708, respectively. The improvement ranged from 9.38% to 27.24% for MCC and 4.84% to 11.29% for ACC, demonstrating the performance consistency between cross-validation and independent assessment.

Comparison of MLCPP 2.0 with the deep learning-based models

Furthermore, we developed a deep learning-based approach for predicting CPP and uptake efficiency using pre-trained embeddings of seq2vec and 1DCNN-BiLSTM. Feature extraction approach and the deep learning architecture for model development are similar to those reported in recent studies.[35,36] When compared to deep learning models, MLCPP 2.0 performed significantly better on layers 1 and 2 (Table S11) training datasets, showing that a stacking approach based on systematic analysis resulted in improved performance. Nevertheless, these smaller training datasets may not be sufficient to construct reliable deep learning predictors.

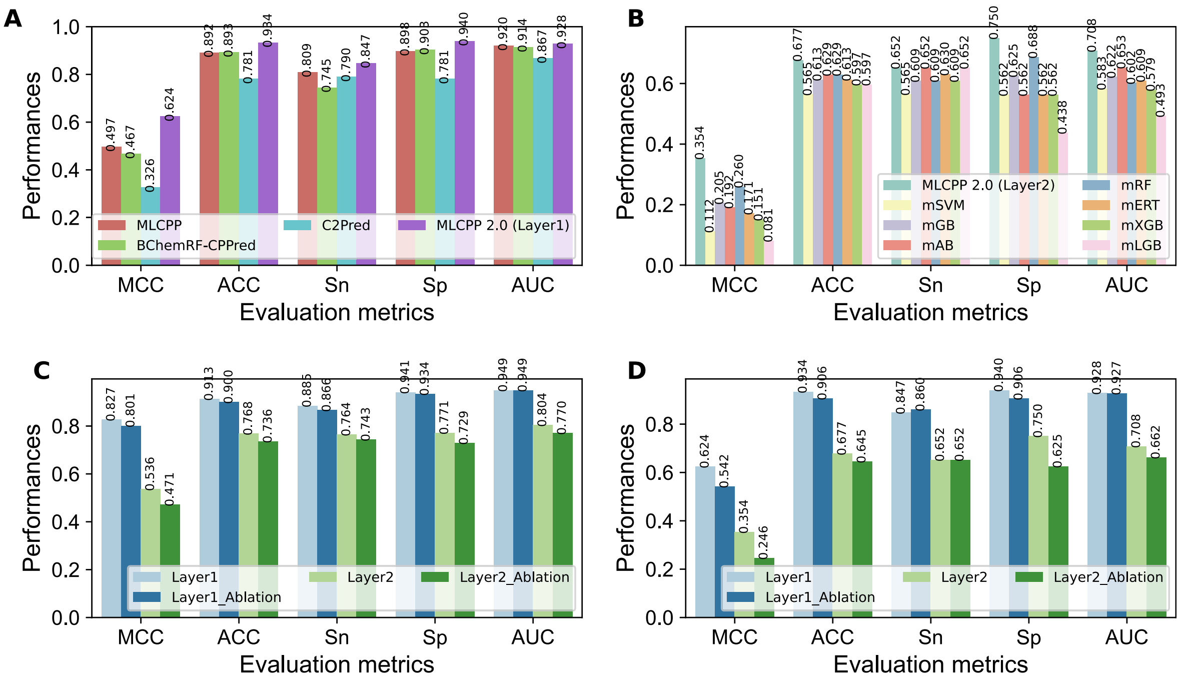

Figure 3. Comparison of MLCPP 2.0 and other methods on an independent dataset. (A) Comparison based on CPP prediction, (B) Comparison based on uptake efficiency, (C) Comparison of cross-validation performance between MLCPP 2.0 and the model lacking SHAP identified top 20 features for both layers, and (D) Independent assessment of MLCPP 2.0 and the model lacking SHAP identified top 20 features.

Model interpretation and ablation analysis

The SHAP algorithm was applied to estimate the SHAP values for 80D PF (layer1) and 50D PF (layer2), respectively. Figure S2(A) shows the top 20 features that contributed to layer1 prediction. It is interesting to note that 7 encodings (AAC, QSO, CTDC, DDE, CKS, DPC, CKSAAGP) based PF play a significant role in CPP prediction. On the other hand, for uptake efficiencies, the ten encodings based PF play an important role (Figure S2(B)) that includes four encodings (CKSAAGP, DPC, AAC, and DPC) involved in layer1. Additionally, we conducted an ablation analysis to determine the importance of each layer's top 20 features. Following that, we compared the performance of MLCPP 2.0 with the models lacking these top 20 features using 10-fold cross-validation and independent assessment. Compared with MLCPP 2.0, Ablation model's cross-validation performance decreased, with 3.0% MCC at layer1 and 6.6% at layer2 (Figure 3(C)). According to an independent assessment (Figure 3(D)), the performance gap between MLCPP 2.0 and the ablation model has widened significantly. This confirms the essential importance of the top 20 PF in MLCPP 2.0.

Webserver implementation

For the convenience of users, a web-server has been developed to implement the MLCPP 2.0 algorithm, which is freely available at https://balalab-skku.org/mlcpp2/. The web-server was constructed with Django, Python, HTML, CSS, and JavaScript programming languages, as well as a PostgreSQL database for storing and retrieving job results. On MLCPP 2.0′s home page, users can download the curated datasets used in the study and find instructions on how to use the web-server**.** A user may either paste one or more query sequences in FASTA format or upload a file containing multiple FASTA sequences for the prediction. When a job is completed successfully, the results are displayed in a separate interface, where the results can also be downloaded in CSV format for offline use. In MLCPP 2.0, results of previously completed jobs can be retrieved by typing the job ID into the 'find job' option on the submission page.

Conclusion

We presented a stacked ensemble learning approach called MLCPP 2.0 in this study. In the first step, we created a pool of 119 baseline models using 17 different feature encodings and 7 ML classifiers. Subsequently, the optimal baseline models and appropriate classifiers were determined for building a stacked model independently for layer1 and layer2. Furthermore, we demonstrated that the stacking approach involving multiple ML-based baseline models is

essential to achieve a better performance than individual meta-models and deep learning models. The results of the independent tests showed that MLCPP 2.0 achieved a superior performance to existing predictors. Additionally, we confirm the importance of the top 20 features independently for both layers in the interpretation of the model using the SHAP algorithm. The major limitation of MLCPP 2.0 is that false negatives predicted at the first layer will not be processed for the second layer prediction. MLCPP 2.0 has a webserver, which can be accessed for free from the following address: https://balalab-skku.org/mlcpp2/.

MLCPP 2.0′s improved performance is primarily due to two attributes: (i) extensive evaluation of multiple encodings and classifiers during baseline model construction and (ii) identification of the optimal feature set (predicted probabilities) and classifier to construct the stacking strategy. This framework can be applied to other sequence-based function prediction problems, such as peptide therapeutic function predictions and DNA/RNA epigenetic modification site predictions.[37–39] Developing a comprehensive CPP database through thorough literature research is one of the most essential aspects of future CPP research.

Acknowledgments

This work is supported by the National Research Foundation of Korea (NRF) funded by the Korean government (MSIT) (2021R1A2C1014338).

Author contributions

B.M., conceived the project and designed the experiments. B.M., and M.C.P., performed the experiments and analyzed the data, and B.M, and M.C.P., wrote the manuscript. All authors read and approved the final manuscript.

Conflict of interest

The authors declare no competing interests.

Appendix A. Supplementary material

Supplementary data to this article can be found online at https://doi.org/10.1016/j.jmb.2022.167604.

Received 30 November 2021;
Accepted 19 April 2022;
Available online 28 April 2022

Keywords:
cell-penetrating peptides;
uptake efficiency;
stacking framework;
feature optimization;
machine learning

References

1. Vargason, A.M., Anselmo, A.C., Mitragotri, S., (2021). The evolution of commercial drug delivery technologies. *Nature Biomed. Eng.* **5**, 951–967.
2. Pooga, M., Langel, U., (2015). Classes of Cell-Penetrating Peptides. *Methods Mol. Biol.* **1324**, 3–28.
3. Shi, N.Q., Gao, W., Xiang, B., Qi, X.R., (2012). Enhancing cellular uptake of activable cell-penetrating peptide-doxorubicin conjugate by enzymatic cleavage. *Int. J. Nanomed.* **7**, 1613–1621.
4. Boisguerin, P., Giorgi, J.M., Barrere-Lemaire, S., (2013). CPP-conjugated anti-apoptotic peptides as therapeutic tools of ischemia-reperfusion injuries. *Curr. Pharm. Des.* **19**, 2970–2978.
5. Nasrollahi, S.A., Fouladdel, S., Taghibiglou, C., Azizi, E., Farboud, E.S., (2012). A peptide carrier for the delivery of elastin into fibroblast cells. *Int. J. Dermatol.* **51**, 923–929.
6. Lehto, T., Kurrikoff, K., Langel, U., (2012). Cell-penetrating peptides for the delivery of nucleic acids. *Expert Opin. Drug Deliv.* **9**, 823–836.
7. Dekiwadia, C.D., Lawrie, A.C., Fecondo, J.V., (2012). Peptide-mediated cell penetration and targeted delivery of gold nanoparticles into lysosomes. *J. Pept. Sci.* **18**, 527–534.
8. Glogau, R., Blitzer, A., Brandt, F., Kane, M., Monheit, G.D., Waugh, J.M., (2012). Results of a randomized, double-blind, placebo-controlled study to evaluate the efficacy and safety of a botulinum toxin type A topical gel for the treatment of moderate-to-severe lateral canthal lines. *J. Drugs Dermatol.* **11**, 38–45.
9. Liu, J., Afshar, S., (2020). In Vitro Assays: Friends or Foes of Cell-Penetrating Peptides. *Int. J. Mol. Sci.* **21**
10. Agrawal, P., Bhalla, S., Usmani, S.S., Singh, S., Chaudhary, K., Raghava, G.P., (2016). CPPsite 2.0: a repository of experimentally validated cell-penetrating peptides. *Nucleic Acids Res.* **44**, D1098–D1103.
11. Gautam, A., Singh, H., Tyagi, A., Chaudhary, K., Kumar, R., Kapoor, P., (2012). CPPsite: a curated database of cell penetrating peptides. *Database (Oxford)* **2012**, bas015.
12. Kardani, K., Bolhassani, A., (2021). Cppsite 2.0: An Available Database of Experimentally Validated Cell-Penetrating Peptides Predicting their Secondary and Tertiary Structures. *J. Mol. Biol.* **433**, 166703.
13. Hällbrink, M., Kilk, K., Elmquist, A., Lundberg, P., Lindgren, M., Jiang, Y., (2005). Prediction of Cell-Penetrating Peptides. *Int. J. Pept. Res. Ther.* **11**, 249–259.
14. Hansen, M., Kilk, K., Langel, U., (2008). Predicting cell-penetrating peptides. *Adv. Drug Deliv. Rev.* **60**, 572–579.
15. Holton, T.A., Pollastri, G., Shields, D.C., Mooney, C., (2013). CPPpred: prediction of cell penetrating peptides. *Bioinformatics* **29**, 3094–3096.
16. Gautam, A., Chaudhary, K., Kumar, R., Sharma, A., Kapoor, P., Tyagi, A., (2013). In silico approaches for designing highly effective cell penetrating peptides. *J. Transl. Med.* **11**, 74.
17. Tang, H., Su, Z.D., Wei, H.H., Chen, W., Lin, H., (2016). Prediction of cell-penetrating peptides with feature selection techniques. *Biochem. Biophys. Res. Commun.* **477**, 150–154.

18. Wei, L., Tang, J., Zou, Q., (2017). SkipCPP-Pred: an improved and promising sequence-based predictor for predicting cell-penetrating peptides. *BMC Genomics* **18**, 742.
19. Wei, L., Xing, P., Su, R., Shi, G., Ma, Z.S., Zou, Q., (2017). CPPred-RF: A Sequence-based Predictor for Identifying Cell-Penetrating Peptides and Their Uptake Efficiency. *J. Proteome Res.* **16**, 2044–2053.
20. Manavalan, B., Subramaniyam, S., Shin, T.H., Kim, M.O., Lee, G., (2018). Machine-Learning-Based Prediction of Cell-Penetrating Peptides and Their Uptake Efficiency with Improved Accuracy. *J. Proteome Res.* **17**, 2715–2726.
21. Pandey, P., Patel, V., George, N.V., Mallajosyula, S.S., (2018). KELM-CPPpred: Kernel Extreme Learning Machine Based Prediction Model for Cell-Penetrating Peptides. *J. Proteome Res.* **17**, 3214–3222.
22. Fu, X., Cai, L., Zeng, X., Zou, Q., (2020). StackCPPred: a stacking and pairwise energy content-based prediction of cell-penetrating peptides and their uptake efficiency. *Bioinformatics* **36**, 3028–3034.
23. de Oliveira, E.C.L., Santana, K., Josino, L., Lima, E.L.A.H., de Souza de Sales Junior, C., (2021). Predicting cell-penetrating peptides using machine learning algorithms and navigating in their chemical space. *Sci. Rep.* **11**, 7628.
24. Basith, S., Manavalan, B., Hwan Shin, T., Lee, G., (2020). Machine intelligence in peptide therapeutics: A next-generation tool for rapid disease screening. *Med. Res. Rev.* **40**, 1276–1314.
25. Su, R., Hu, J., Zou, Q., Manavalan, B., Wei, L., (2020). Empirical comparison and analysis of web-based cell-penetrating peptide prediction tools. *Brief Bioinform.* **21**, 408–420.
26. Fu, L., Niu, B., Zhu, Z., Wu, S., Li, W., (2012). CD-HIT: accelerated for clustering the next-generation sequencing data. *Bioinformatics* **28**, 3150–3152.
27. Basith, S., Lee, G., Manavalan, B., (2021). STALLION: a stacking-based ensemble learning framework for prokaryotic lysine acetylation site prediction. *Brief Bioinform.*.
28. Hasan, M.M., Basith, S., Khatun, M.S., Lee, G., Manavalan, B., Kurata, H., (2021). Meta-i6mA: an interspecies predictor for identifying DNA N6-methyladenine sites of plant genomes by exploiting informative features in an integrative machine-learning framework. *Brief Bioinform.* **22**
29. Wei, L., He, W., Malik, A., Su, R., Cui, L., Manavalan, B., (2021). Computational prediction and interpretation of cell-specific replication origin sites from multiple eukaryotes by exploiting stacking framework. *Brief Bioinform.* **22**
30. Xie, R., Li, J., Wang, J., Dai, W., Leier, A., Marquez-Lago, T.T., (2021). DeepVF: a deep learning-based hybrid framework for identifying virulence factors using the stacking strategy. *Brief Bioinform.* **22**
31. Li, H.L., Pang, Y.H., Liu, B., (2021). BioSeq-BLM: a platform for analyzing DNA, RNA and protein sequences based on biological language models. *Nucleic Acids Res.* **49**, e129.
32. Liu, B., Gao, X., Zhang, H., (2019). BioSeq-Analysis2.0: an updated platform for analyzing DNA, RNA and protein sequences at sequence level and residue level based on machine learning approaches. *Nucleic Acids Res.* **47**, e127.
33. Li, F., Guo, X., Jin, P., Chen, J., Xiang, D., Song, J., (2021). Porpoise: a new approach for accurate prediction of RNA pseudouridine sites. *Brief Bioinform.* **22**
34. Mc, N.Q., (1947). Note on the sampling error of the difference between correlated proportions or percentages. *Psychometrika* **12**, 153–157.
35. Sharma, R., Shrivastava, S., Singh, S.K., Kumar, A., Singh, A.K., Saxena, S., (2021). Deep-AVPpred: Artificial intelligence driven discovery of peptide drugs for viral infections. *IEEE J. Biomed. Health Inform.* **PP**
36. Singh, V., Shrivastava, S., Kumar Singh, S., Kumar, A., Saxena, S., (2022). StaBle-ABPpred: a stacked ensemble predictor based on biLSTM and attention mechanism for accelerated discovery of antibacterial peptides. *Brief Bioinform.* **23**
37. Chenarani, N., Emamjomeh, A., Allahverdi, A., Mirmostafa, S., Afsharinia, M.H., Zahiri, J., (2021). Bioinformatic tools for DNA methylation and histone modification: A survey. *Genomics* **113**, 1098–1113.
38. Lv, H., Dao, F.Y., Zhang, D., Yang, H., Lin, H., (2021). Advances in mapping the epigenetic modifications of 5-methylcytosine (5mC), N6-methyladenine (6mA), and N4-methylcytosine (4mC). *Biotechnol. Bioeng.* **118**, 4204–4216.
39. Dao, F.Y., Lv, H., Su, W., Sun, Z.J., Huang, Q.L., Lin, H., (2021). iDHS-Deep: an integrated tool for predicting DNase I hypersensitive sites by deep neural network. *Brief Bioinform.* **22**
