# Supplementary material for: PDBx/mmCIF Ecosystem: Foundational Semantic Tools for Structural Biology
Source: J Mol Biol. Author manuscript; Available in PMC 2023 Jun 26. (PMC10292674; doi:10.1016/j.jmb.2022.167599)
Supplement: Article [file NIHMS1907597-supplement-Article.zip › MarkerML---Marker-Feature-Identification-in-Metagenomic_2022_Journal-of-Mole.pdf]

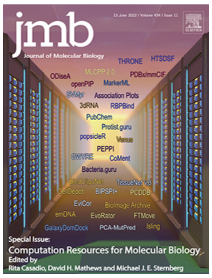

MarkerML – Marker Feature Identification in Metagenomic Datasets Using Interpretable Machine Learning

Sunil Nagpal [1,2,3], Rohan Singh [1], Bhupesh Taneja [2,3] and Sharmila S. Mande [1]*

*1 - **TCS Research,** Tata Consultancy Services Ltd, Pune 411 013, India*
*2 - **CSIR-Institute of Genomics and Integrative Biology (CSIR-IGIB),** New Delhi 110 025, India*
*3 - **Academy of Scientific and Innovative Research (AcSIR),** Ghaziabad 201 002, India*

Correspondence to Sharmila S. Mande: *sharmila.mande@tcs.com (S.S. Mande)*
https://doi.org/10.1016/j.jmb.2022.167589
Edited by Michael Sternberg

Abstract

Identification of environment specific marker-features is one of the key objectives of many metagenomic studies. It aims to identify such features in microbiome datasets that may serve as markers of the contrasting or comparable states. Hypothesis testing and black-box machine learnt models which are conventionally used for identification of these features are generally not exhaustive, especially because they generally do-not provide any quantifiable relevance (context) of/between the identified features. We present MarkerML web-server, that seeks to leverage the emergence of interpretable machine learning for facilitating the contextual discovery of metagenomic features of interest. It does so through a comprehensive and automated application of the concept of Shapley Additive Explanations in companionship to the compositionality accounted hypothesis testing for the multi-variate microbiome datasets. MarkerML not only helps in identification of marker-features, but also enables insights into the role and interdependence of the identified features in driving the decision making of the supervised machine learnt model. Generation of high quality and intuitive visualizations spanning prediction effect plots, model performance reports, feature dependency plots, Shapley and abundance informed cladograms (Sungrams), hypothesis tested violin plots along-with necessary provisions for excluding the participant bias and ensuring reproducibility of results, further seek to make the platform a useful asset for the scientists in the field of microbiome (and even beyond). The MarkerML web-server is freely available for the academic community at https://microbiome.igib.res.in/markerml/.

© 2022 Elsevier Ltd. All rights reserved.

Introduction

Hypothesis testing and its variants have consistently enabled reliable class comparisons between contrasting (e.g., case-control) states for identifying differentiating microbial features (or biomarkers).[1,2] Rooted in the classical concept of class discrimination, it aims to mine the compositional metagenomic next-generation sequencing (NGS) data for identification of features that can quantifiably distinguish one class from the other (e.g, comparing microbial abundances present in the stool of IBD and Healthy cohort).[2,3] Methods that account for compositionality of microbiome data along-with considerations to sampling fraction bias and confounding factors are often preferred for a reliable class comparison.[4–6] While such methods can reveal significantly differentiating features (e.g. taxonomic units) with reportable false discovery rates for individual taxa, they do not necessarily indicate class predicting capabilities of the differentially abundant features (in combination or isola-

tion). Class prediction, though apparently synonymous, is a foundationally different concept from class comparison.[2,7,8] It employs features in the data that can aid development of a (mathematical) model for predicting the class affiliation of data. For example, it can help in identifying the state of health by examining the stool microbiome of a subject. Based on the fundamental concept of supervised machine learning (ML), class prediction approach has not only been enabling development of diagnostic or predictive methods but has also aided the discovery of marker-features.[7,9–11] This is achieved through identification of composite features of the machine learnt model. Currently, microbiome community relies on regression (e.g. ridge or lasso rooted linear and logistic regression) and decision tree (e.g. Random Forest, gradient boosted trees like XGboost, LightGBM) based supervised machine learning methods available in different packages designed for specific programming languages.[12–14] A limited set of these methods are available in a handful of web-servers.[15,16] However, accessibility aside, in the current state of the art of metagenomics research, use of machine learnt models and composite features follow a 'black-box paradigm'. In other words, it is based on a set of microbial-features that are fed to the 'black-box' ML frameworks, for building predictive models which are often complex, without contextual knowledge of the drivers of the decisions made by the developed model. The latter requirement comes under the purview of 'interpretable machine learning' wherein algorithms are adopted to enable interpretation of the contribution of each feature of the model to the predictable outcomes.[17,18] These interpretability algorithms can be of two types, namely, (i) intrinsic, wherein the learnt model is self-explainable and (ii) post-hoc, wherein a downstream or companion algorithm attempts to decipher the machine learnt model.[19] Notably, interpretability of self-explainable models (e.g. coefficients in linear logistic regression and node/ branch tracing of decision trees) is mostly dependent on the complexity of the learnt model. For example, the decision tree-based learner developed using a high-dimension metagenomic dataset is often too deep and complex to trace. Post-hoc explainability algorithms, particularly the use of SHapley Additive exPlanations (SHAP), are emerging as preferred methods for decoding the black-box as well as self-explainable models because of their model-agnostic and uniformly characterized approach to model explanation.[17–20] Briefly, Lloyd Shapley introduced a solution concept in 1951 which enables fair distribution of awards and costs of a game's outcome to the cooperating players of the game.[21] Analogously, the decision of a machine learnt model can be treated as the game outcome and the features (e.g. metagenomic features) may be perceived as actors/players of the game (where game represents the complex interplay of features). Importantly, unlike other available methods of model interpretation through fair-payout assessment, reliable feature identification is possible by leveraging three key properties of Shapley values.[20] These three properties pertain to 'Efficiency for ensuring that the total outcome is distributed amongst all participating players, 'Symmetry' for ensuring equal values to players influencing equally and 'Null player/ Dummy' for ensuring zero value to the player having no influence on the game.[20,21] A schematic description of the concept of Shapley Additive Explanations, inherited from the use of Shapley values in coalition game theory is presented in Supplementary File 1. More than 4000 published studies (since Dec 2017) from various disciplines have utilized and reviewed the unified method of SHapley Additive exPlanations (SHAP) as it accounts for majority of the community trusted model explainability methods. This indexes the growing preference for simplified interpretations for otherwise black-box or complex models.[20]

Given the high and often multi-dimensional nature of the metagenomic datasets, it is common to observe the development of complex/black box machine learnt models.[22] This doesn't add significant value to the process of marker-feature identification, which has traditionally been reliant on limited scope of class comparison methods (hypothesis testing) in identifying differentially abundant features (which do not necessarily imply predictivity). Interpretable machine learning can add a much-needed context to the hitherto complex models while enabling discovery of meaningful metagenomic – features. The development of explainable models can be further useful when they are coupled with the statistical identification of the microbial markers using class comparison approaches that account for sparsity, compositionality, confounder and sampling biases, characteristic of microbiome datasets. A handful of recent microbiome-research studies, attempting metagenomic feature identification using explainable artificial intelligence (explainable AI) and machine learning, are highly encouraging developments in this regard.[23–28] However, while tools (standalone or web-servers) for class comparison and 'black-box machine learning' based discovery of metagenomic marker-features are quite prevalent, explainable AI/machine learning is yet to attract similar attention. This is particularly true for development of methods that aim to unite the process of marker-feature discovery through class comparisons and interpretable class predictions.[5] In addition, existing tools in ML based marker-feature identification could become useful to metagenomics research community if key pitfalls in a machine learning workflow are automatically avoided or the end-user is given sufficient information to account for the potential biases. For example, unbalanced class sizes, training–testing data leakage and participant (or sample dependency) bias are some of the common issues that

can percolate into a machine learning workflow. Here, unbalanced class size refers to the frequently observed skew in sampling size between the controls and the cases. This has the potential to cause unbalanced learning (model preferentially trained for one class having a larger sample size). Training-testing data leakage happens when model development process has already sniffed the entire data, a subset of which is employed for model validation and performance reporting. This can happen due to improper splitting or segregation of training and testing datasets or appriori selection of features on entire dataset followed by model development/validation/testing. Another common pitfall frequently faced in ML model development for metagenomic datasets (and even other biological datasets) is patient or sample dependency bias. This refers to the presence of multiple or dependent samples from the same patient. If a traditional random splitting of training–testing datasets is employed on a dataset containing multiple samples from same patient (which is very common in clinical research – e.g. lesional and non-lesional samples of a participant suffering from a skin disorder), then a biased assessment of such models may happen if samples from the same patient are present in training as well as testing sets. In such cases, it is critical to ensure that data-splitting is participant-ID or dependency aware, so that training as well as testing datasets are mutually exclusive with respect to the study-participants. To our knowledge, tools (especially programming language-independent web-servers) addressing the aforementioned pitfalls, and facilitating companionship of explainable AI (interpretable machine learning) with compositionality accounted hypothesis testing are currently not available. Such resources, in addition to being generally useful can be valued assets to clinicians and researchers who are limited by the access/awareness of offline programming languages, general pitfalls and required computational resources. A summarized comparison of the existing commonly used web-based tools in metagenomic biomarker discovery is presented in Table 1. Briefly, most of these tools are meant for offering a one-stop platform for a variety of analytical goals and are therefore not specialized for a given objective. While one of the key foci of these tools is class comparison, the integration of conventional machine learning methods is available in very few tools (e.g. in MicrobiomeAnalyst, Calypso and METAGENassist), that too for basic predictivity assessment without due consideration to the avoidance of key pitfalls or model explain-ability (as described earlier). Notably, LefSe was first to attempt explainable class comparison using linear discriminant analysis (LDA). While the method has machine learning fundamentals, LefSe employed LDA for meaningful class comparison and corresponding effect size estimation for the features, rather than for class prediction.

We present MarkerML that seeks to provide a unique web-based platform enabling seamless union of interpretable machine learning with statistical (hypothesis) testing for contextual discovery of marker-features in metagenomic datasets (Figure 1). Following are some of the key properties of the platform which are expected to enable easy identification of marker features:

(1) Simple tabulated input data
(2) Comprehensive data normalization options and considerations for data compositionality
(3) Back-end feature-lineage databases specific to Taxonomic,[31] KEGG,[32] COG,[33] PFAM[34] and TIGRFAM[35] hierarchies to enable automated and intelligent augmentation of marker-feature characterization
(4) Machine learning framework with comprehensive feature selection and data sharing in conformity with TRIPOD/DOME[36,37] guidelines for reproducible research
(5) Automated provisioning for common pitfalls pertaining to unbalanced sampling, data leakage during training and sample dependency due to multiple samples from same participant
(6) Choice of different machine learning algorithms (XGBoost,[12] Random Forest,[14] Logistic regression[38] and LightGBM) to address assumptions of non-linearity and linearity in microbial feature interactions
(7) SHAP[20] based in-depth model interpretability for marker feature identification
(8) Hypothesis testing in conformity with characteristics of metagenomic datasets (e.g. data compositionality, sampling fraction bias) using ALDEx2/ANCOMBC for complementing or benchmarking the predictive marker features against the class comparison approach

Table 1 Existing commonly used web-based tools for metagenomic biomarker discovery.

Tool	Comparison[#]	Prediction[#]	Interpretable ML	Reference
LefSe	☑	☒	☒	2
MicrobiomeAnalyst	☑	☑	☒	16
Calypso	☑	☑	☒	15
METAGENassist	☑	☑	☒	29
MG-RAST	☑	☒	☒	30

[#] Comparison refers to hypothesis testing based class comparison. Prediction refers to machine learning based predictive model development.

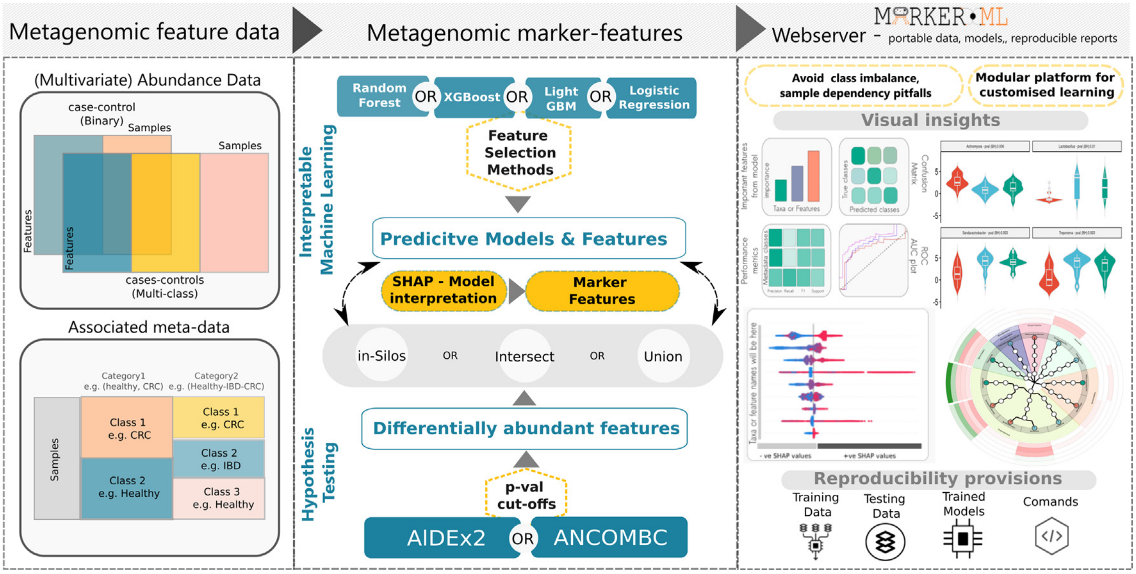

Figure 1. Graphical summary of the overall workflow of MarkerML.

(9) Customizable and intuitive visualizations spanning prediction effect plots, model performance reports, feature dependency plots, '**s**hapley, ab**un**dance and taxonomy' informed clado**gram**s (Sungrams) and hypothesis tested violin plots

Methods

Implementation

MarkerML is deployed on a scalable Linux based operating system (Ubuntu 20.04.3 x86_64) hosted at the computational facility of CSIR-IGIB, New Delhi, India (https://microbiome.igib.res.in/markerml/). The backend is powered by Python 3.8.10, R and bash while the front end is built over HTML/CSS with appropriate use of jquery and asynchronous javascript and XML (AJAX) for communication between the client and server end using PHP (a server scripting language). The platform, in addition to using the standard libraries of Pandas,[39] Scikit-learn,[40] Xgboost,[12] SHAP,[20] Graphlan[41] and Yellowbrick[42] includes use of extensive in-house coding as well as scripting to enable the implementation of various dynamic workflows and customizable visualizations. A previously described local job history tracking system has also been implemented for convenient access to the submitted (during the past seven days) tasks.[43] The platform has been tested for working on commonly used modern browsers like Mozilla firefox (version 92.0.1), Chrome (Version 95.0.4638.54) and Microsoft Edge (Version 95.0.1020.40).

Application structure

Following are the two key application interfaces or modules in MarkerML:

(a) ***Main submission module:*** Primary module for metagenomic marker discovery
(b) ***Lineages DB:*** A queryable database of hierarchical lineages of metagenomic features

(a) Submission module

Submission module accepts user-provided multivariate data-tables in order to identify marker features using the dynamic user-driven workflows. The module is further structured into following 4 sub-modules:

(i) '**Data and parameter selection section**' wherein feature abundance data (e.g. taxonomic occurrence table) and metadata are uploaded alongwith selection of state of the art data normalization methods (TSS, CSS, CLR), file formats (TSV/CSV, presence/absence of lineage information), machine learning algorithms (RandomForest, XGBoost, Logistic regression, LightGBM), model training–testing parameters (data split size, cross fold validations, feature selection methods, sample dependency) among more.
(ii) **Visualizations section** contains five containers for depicting various graphical outcomes of the submitted task. The graphical results are generated based on the input parameters provided by the end user for the chosen metadata categories, as auto-detected by MarkerML.
(iii) **Textual results section** provides access to the statistical summaries and the textual files that form the basis of the graphical visualizations.
(iv) **For reproducibility** section has been built with an aim to follow the DOME and TRIPOD guidelines for accessibility and reproducibility in machine learning workflows.[36,37]

(b) LineagesDB

LineagesDB is a curated database of taxonomic lineage (compiled using RDP's Bergey's tree – 2,175 entries), Enzyme lineage (mapped to KEGG pathways – 5,842 entries), PFAM lineage (19,179 entries), TIGRFAM lineage (4,306 entries), COG lineage (4,875 entries), all compiled using the information validated using respective data portals.[31–35] This unique one-stop lineage database enables convenient access to lineage information for the metagenomic-features when the said information is unavailable with the end-user. Given that MarkerML (and even other tools) has a provision to mine for (as well as visualize) marker features at user-selected levels of feature lineage, availability of a resource that can provide seamless access to the required information in proper format is anticipated to be of value to the community. To facilitate the same LineagesDB has been developed with a highly simplified query system, wherein given a list of names from the most specific level of a metagenomic-feature lineage, the module instantly provides the lineage information in textual format for the features that matched the database.

Application workflow/algorithm design:

MarkerML relies on two fundamental concepts in biomarker discovery:

(i) Class comparison (CC)
(ii) Class prediction (CP)

Class comparison is performed through the differential abundance analysis of the metagenomic feature-count data (with due consideration to bias arising due to different sampling fractions, data sparsity and compositionality) as previously recommended for the microbiome (HTS) data using ANCOM-BC and ALDEx2.[4–6] ANCOM-BC involves use of a linear regression framework for supplied microbiome data assuming that each sample is an unknown fraction of a unit volume in the ecosystem and attempts estimating said fractions for an unbiased hypothesis testing.[4] Compositionality is accounted through log scale implementation of regression framework while zero-inflation is adjusted using ANCOM-II. The decision for binary or multi-class comparison (hypothesis testing) is auto-inferred by ANCOM-BC. ALDEx2 involves addressing the sparsity/zero inflation through Monte Carlo sampling on the Dirichlet distribution (corrected with a uniform prior) followed by the cumulative log-ratio (CLR) transformation to account for compositionality.[6] Subsequently, based on the number of classes (e.g. healthy, diseased in a metadata category like HealthState) that the user wishes to compare, MarkerML auto-enables the Wilcoxon rank sum test (for binary comparison) or Kruskal-Wallis test (for grouped comparison) mode of ALDEx2. The p-values for both ANCOM-BC and ALDEx2 are corrected using Benjamini–Hochberg procedure and well annotated violin plots are auto-generated for the CLR transformations of the significantly differentiating features. By default, MarkerML uses ALDEx2 for class comparison, while users are free to use ANCOM-BC.

Class prediction is performed using four state-of-the-art machine learning methods (XgBoost, Random Forest,Logistic Regression and LightGBM) in a binary or multi-class (One vs Rest)[44] setting (as per the number of predicted classes). As introduced previously, given that class prediction is rooted in machine learning, due care is needed in provisioning for unbiased training, sufficient cross-validation, feature-selection, held-out-testing and reproducibility.[36] These goals have constantly been stressed upon by the research community in machine learning/artificial intelligence (e.g. DOME and TRIPOD guidelines).[36,37] The class prediction workflow of MarkerML has been designed to address these community requirements/recommendations.[36,37] The input data is first split into user defined proportions of training and held-out testing subsets (e.g. 80:20 split by default). A preliminary step of unsupervised feature selection is performed here on the training data. This involves trimming of features that fail the variance threshold specified by the user (default: 0 i.e. absolutely non-varying or constant features across training samples are trimmed). This is followed by user defined cycles of cross-validation of user-chosen machine learning method for the given data (k-fold cross validation). In each fold, the data is proportioned into sub-train and sub-test (validation) using the same user defined split ratio as employed for creating the training and held-out test data. Models are generated using sub-train and tested on sub-test of each fold to report average performance metrics (Accuracy ± std and ROCAUC ± std) for the k-folds validation. The cross-validation cycles are also employed for feature selection using either of the two user-specified supervised feature selection techniques – (i) Successive aggregation (ii) Recursive feature elimination. In successive aggregation, a model is developed using the subset-training data and model embedded feature importance are used to filter out null-importance features. Rest of the informative features are stored in a data frame which is merged with the informative features of next fold. Once all (k) folds are completed, features are sorted according to their aggregate importance across all iterations and top (user-defined, default 50) N features are selected for model development using the entire training data. Recursive feature elimination (as adopted from python's scikit) on the other hand iteratively removes two uninformative features (elimination of which improves model performance) in each cycle from total-pool of features until user-specified number (N) of features are retained for developing the model on the original training data. Results of the performance of devel-

oped model on held-out test-data (confusion matrix, accuracy, precision, recall, f1-score, ROC AUC) as well as results from the cross-validation folds (mean and standard deviation of accuracy and ROC AUC) are reported in a unified multi-panel report as previously described by the authors.[45] These model performance reports allow informed decision making regarding the suitability/confidence of predicted markers in subsequent stage of marker feature identification. The marker feature identification is performed through post-hoc model interpretability using SHapley Addivitive ExPlanations (SHAP) (Supplementary File 1).[20,46] Here, each feature is assigned a SHAP value against each prediction it participated in. A null SHAP value would indicate an uninformative feature, while a negative/positive SHAP value indicates the impact it has on the model outcome. In other words, while a positive value would favor the case prediction in control-case study, a negative value would favor the contribution towards control prediction. Therefore, higher the magnitude of SHAP value, greater is its impact on model outcome. Consequently, there is a scope for feature filtration by setting a minimum needed SHAP threshold, thereby retaining only highly informative features in case of feature plurality. Notably, SHAP values can have dataset specific ranges. We have therefore attempted to provide an added interface to trim the features based on SHAP values of features. MarkerML allows the end-users to define the threshold through a convenient slider interface, enabling instant update of informative features under different stringency cut-offs. Choice of SHAP thresholds is an informed decision that the end-user should make based on the dispersions observed in the SHAP plots. This can be instantly applied without the need for re-running the entire workflow. In addition, as highlighted in supplementary Figures S2, S4, S6, SHAP plots (generated only using training data) can also enable choice of N (number of useful features) by observing the sharp drop in SHAP values (point of inflection). Supplementary Figure S1 provides a schematic flow of the methods employed in CC and CP components of MarkerML.

With an aim to offer flexibility to end-user for customization of marker feature identification workflow, MarkerML provides three simple but intuitive workflow-modes:

(1) **Joint (CC ∩ CP):** In this workflow, Interpretable machine learning (iML) is performed only on the marker features inferred by hypothesis testing (effectively the intersection between class comparison and prediction). Briefly, once the training and held-out test data is created, hypothesis testing is initiated on training data using user-defined method (ANCOMBC or ALDEx2). Thereafter, the features selected through successive aggregation or recursive feature elimination steps of ML workflow are intersected with the significantly differentiating features inferred from hypothesis testing. The resultant set of features are used to develop the final model which is tested using the held-out test data and the features of these model are used for SHAP value computation i.e. for interpreting the model. This workflow can enable end-users test the predictivity, model contributions and inter-dependence of features observed to be statistically significantly differentiating. Notably, we have designed this workflow to perform hypothesis testing on the training data (and not on 'entire data') to ensure that there is no data/information leakage for downstream model development. If the entire data is employed for hypothesis testing, then the derived (significantly differentiating) features would also be representative of the held-out test data (which is a subset of the entire data). This would lead to over-estimated model performance. We therefore discourage the post-hoc model development using features derived from hypothesis testing performed on entire datasets. It can serve as a potential source of data leakage, which should be strictly avoided in machine learning.

(2) **Post-hoc unite (CC ∪ CP):** This workflow involves the reporting of the union of class comparison and class prediction markers. In the previous steps of feature finalization as explained in 'Joint mode', instead of performing the intersection, a union of features selected in ML workflow and those inferred through hypothesis testing is performed. This ensures that features learnt in ML workflow are also considered for assessing the performance on held-out test data.

(3) **Independent flow (CC ⫫ CP):** Marker features are independently reported without any communication between class comparison and class prediction approach. Here, instead of hypothesis testing only on training data, the entire data is utilized (as there is no interaction between class comparison and class prediction). Machine learning workflow is carried out independently. Results are independently reported to enable end-users make an informed decision or inference regarding the corroboration between class comparison and class prediction.

Results & Discussion

MarkerML generates the following five key graphical results - Supplementary File 2 provides a detailed guide for the interpretation of plots generated by MarkerML.

(a) the effect of outcome defining contextual-marker-features on the class-predictions using bee-swarm plot (for binary metadata choice) or grouped bar chart (for multi-category metadata choice).

(b) the violin plots for marker features detected by compositionality accounted hypothesis testing.
(c) a comprehensive multi-panel model performance report (showing the non-contextual model derived top-5 important features, namely, accuracy, precision, recall, f1-score, ROC-AUC curve and confusion matrix.
(d) a unique clado**gram** (termed Sungram plot) that incorporates the hierarchical lineage information of the marker features, the prediction influence of features, **S**HAP values and original ab**un**dance values in a single plot.
(e) multiple dependency scatter-plots reporting the tripartite relationship between the SHAP value of a top ranked feature against its original abundance value as well as the abundance of another top ranked feature.

Textual section of MarkerML contains results corresponding to the cross-validation (as chosen by the end-user), global SHAP and median abundance values for each feature (in chosen classes of metadata) in tabulated/textual formats for further analysis/reference of the end-users (refer supplementary file 1 for an intuition towards SHAP values and model interpretability). The detailed tabulated summary of class-comparison based marker features (hypothesis testing and results thereof) is also generated in this section. Supplementary File 3 provides additional details for interpreting the textual results of MarkerML.

In addition to the (graphical and textual results), MarkerML dynamically generates the trained/interpreted model files, training–testing datasets and the commands used for data handling followed by training, testing and cross-validation. This is done to aid the accessibility and reproducibility goals of the research studies linked with MarkerML.[36,37]

Workflow benchmarking

Two types of benchmarkings were performed for MarkerML using – (i) a publicly available dataset for corroboration with literature (ii) multiple simulated datasets of different sizes for run-time estimation.

Additionally, the demo section of MarkerML provides access to 4 case studies which can be utilized for exploring the functionalities of the web-server. Further studies will be uploaded in the demo section in future for creating a repository of accessible metagenomic data and relevant models. We demonstrate here one of the use-cases pertaining to the metagenomic-marker identification in human periodontitis.[47] The data employed for the case-study was downloaded from the European Bioinformatics Institute's microbiome analysis resource (Study ID: MGYS00002146).[48] The taxonomic assignments (as available in the demo section of MarkerML) were generated by the EBI MGnify using the pipeline version 4.1.

The data contained a total of 91 16S rDNA (V1-V2) amplicon sequencing samples pertaining to 59 periodontal samples (shallow-pocket samples: 30, deep-pocket samples: 29) and 32 healthy controls. The periodontal samples were dependent since one shallow and one deep-pocket sample was collected from every participant affected with periodontitis. One sample was observed to lack the deep pocket counterpart but was regardless retained. While the taxonomic lineage information was available in the original data, we also used LineagesDB module of MarkerML to create the formatted lineage information for the genus level taxa in the abundance data.

We sought to mine marker features for the comparable classes of 'Control-Periodontitis', 'Control-ShallowPocket (PS)', 'Control-DeepPocket (PD)' and 'Control-PS-PD'. These binary and grouped choices of comparisons were conveniently opted using the dynamic drop-down options available in the data submission section of MarkerML. In order to avoid sample dependency bias during 'Control-Periodontitis' specific model development, MarkerML was made aware of the participant-ID information through advanced settings. All available methods for machine learning, hypothesis testing, and feature selection were tried for all comparable classes (results for all jobs are tabulated in Supplementary Table 1) for lineage level 5 (i.e. genus level), a 80:20 train: test split, 10-fold cross-validation and N = 20 (important features). Here we share the results of RandomForest based estimator employed with ALDEx2 which yielded a CV ROC-AUC of 0.88 ± 0.17 and a held out ROC-AUC of 0.94 across all modes of workflow for successive importance aggregation method of feature selection (N = 20). These results, including other combinations of ML, hypothesis testing, and feature selection method are available in Supplementary Table 1. A post-hoc observation of SHAP value dispersions was additionally attempted (Supplementary Figure S2) which indicated suitability of 7 features for high impact predictivity, which was subsequently attempted (specifying N = 7 for total features to be selected) and found to be useful in achieving comparable results (against N = 20), as described subsequently. The Benjamini Hochberg corrected p-value threshold of 0.01 was chosen for the hypothesis testing (class comparison). The min. SHAP and p-value thresholds can also be changed post feature-learning for dynamic exploration of (un)informative feature space.

Figure 2, supplementary Figures S2–7 and Supplementary Table 1 represent the graphical visualizations/results of the marker feature identification on chosen comparable classes. The

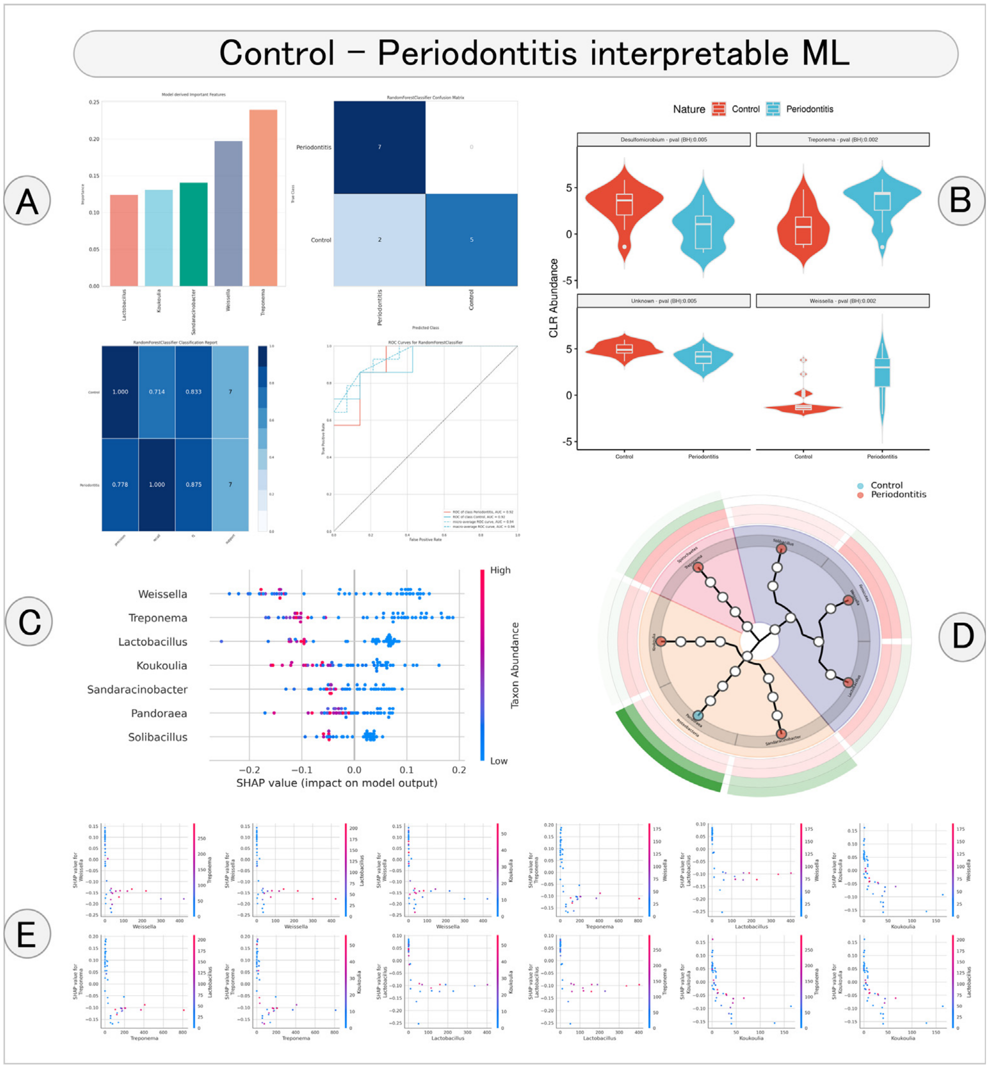

Figure 2. Summary of MarkerML analysis reports for Periodontal vs Control samples. Panel A represents the performance metrics of the RandomForest based ML model. Panel B represents ALDEx2 based hypothesis testing-based boxplots of significantly differentiating features at BH corrected $p < 0.01$. Panel C represent SHAP impact beeswarm plot. Negative values indicate Periodontal outcome while positive values indicate control outcome. Panel D represent the Sungram (Shap, abundance and lineage informed cladogram) wherein inner ring of each pair of rings around the Phylum names pertain to SHAP value and abundance value of the features for Periodontal class. Panel E represent the feature dependency plot for Top 4 features of the SHAP plot of panel A towards control outcome.

non-contextual (conventional) five most informative taxa specific genera (*Treponema, Weissella, Sandaracinobacter, Koukoulia and Lactobacillus*) for the trained 'Control-Periodontits' (accounting for sample dependency) model performance are presented in Figure 2(A). These genera have earlier been founded to have role in oral health, particularly periodontitis/gingivitis.[47,49] The ROC-AUC values (0.92, 0.9 and 0.98 for Held-out-testing in Independent, Joint and Unite modes respectively; and 0.86 ± 0.18 for CV-testing) were similar to the previous reported (held out ROC-AUC: 0.95) prediction outcomes despite setting a very low feature count (7) for the development of final model.[50] A good model performance is indicative of reliable interpretable machine learning outcomes; hence the achieved performance metrics provide sufficient confidence for post-hoc interpretation of the model. Compositionality accounted hypothesis testing (BH corrected p-value < 0.01) indicated the presence of 4 differentially abundant OTUs pertaining to *Treponema, Weissella, Desulfomicrobium* and *an unassigned OTU* (Figure 2 (B)) genera. Previous interest in the role of *Treponema, Desulfomicrobium* and *Weissella* in oral health is well founded.[47,49,51] SHAP based interpretation of ML model interestingly indicated that the absence or low abundance of OTUs pertaining to *Weissella, Treponema, Lactobacillus, Koukoulia, Sandaracinobacter, Pandoraea and Solibacillus* in

a sample mostly contributed the Healthy outcome in ML prediction, while their presence or high abundance drove the opposite (Periodontitis) outcome of prediction by the model (Figure 2(C)). This is not true for *Weissella* which was observed to contribute to Periodontal outcome even at lower abundances (indicating a probable dependency on other features). Notably, as indicated by the dispersion in the SHAP values (Figure 2(C)) many of these marker features (and abundances thereof) do not act alone in conclusively deciding the prediction by the model. Periodontitis outcome (negative SHAP values in this case), in particular, is a function of interaction between the features (Figure 2(C, E)). As observed in the Sungram shown in Figure 2(D) (SHAP and Abundance informed cladogram), *Spirochaetes, Proteobacteria and Firmicutes* are the three phyla to which all observed marker features pertain to. All taxa have a relatively high mean impact (SHAP value) on Periodontal outcome when their abundance increases (inner rings in each ring pair of Sungram) (Figure 2(D)). Dependency plot (Figure 2(E)) indicated that near zero abundances of the top 4 features consistently yielded control outcome at higher SHAP value. The SHAP value would drop if these features had non-zero or higher abundance. Particularly, the higher abundance of *Treponema* seemed to reverse the prediction direction for *Weissella* even if the latter is present at lower abundance (Figure 2(E)). Supplementary File 4 contains the Training and Testing datasets along-with the trained model and commands employed for performance metric calculation. This is expected to aid the reproduction of these results in addition to operation of MarkerML sever using shared parameters.

In order to estimate run-time for MarkerML using different data sizes, two sets of 10 simulated datasets each of different dimensions were created using SparseDOSSA2.[52] Set 1 contained incremental number of samples (100–1000) with a fixed feature size of 500, while set 2 contained incremental number of features (100–1000) with a fixed sample size of 500. Supplementary file 5 provides access to these datasets and the commands used to generate the said data. All 20 jobs executed on MarkerML for these datasets (using default parameters) had a run-time less than 90 secs. The increase in time for incremental dimensions was approximately linear as depicted in Supplementary Figure S8. This may vary based on number of features that are employed for SHAP computation and model performance evaluation. Time to upload the data at step 1 was not considered in run-time, which varied between 1–10 seconds for all attempted dimensions. However, this step is dependent on upload speed of end-user network connection and hence the time to upload data may vary for different users.

Limitations and Future Directions

Causality analysis is a key goal that succeeds identification of biomarkers/ marker features.[53] It seeks to decipher the role of identified features towards establishing the state of health or disease. A cause-effect relationship is however difficult to describe in absence of experimental evidence and mathematical proofs.[54] While class comparison and class prediction can aid marker features identification, these methods do not describe causal-effects. Interpretable machine learning particularly needs to be carefully 'interpreted'. The interpretation is confined towards explanation of the contribution of each feature (and abundance thereof) in driving the predictions of the machine learnt model. The model explanations should not be seen as causal correlations/inferences. The future versions of MarkerML aim to incorporate methods for deriving the causality associations of identified marker features. This will require development of robust/reliable domain knowledgebases powered by evidence from publicly available clinical trials, medical databases and literature (among more).[54] Another major planned development for MarkerML server pertains to enhancing of Demo section with public datasets to create an integrated repository of microbiome datasets and disorder specific interpretable models. Improvement of MarkerML through incremental feature addition like resources and provisions for hyper-parameter tuning, extended job-queuing system, actions on bug reports by community will continue regularly.

CRediT authorship contribution statement

Sunil Nagpal: Conceptualization, Methodology, Software, Formal Analysis, Visualization, Investigation, Validation, Writing – original draft, Writing – review & editing. **Rohan Singh:** Validation, Writing – review & editing. **Bhupesh Taneja:** Conceptualization, Project Administration, Writing – review & editing. **Sharmila S. Mande:** Conceptualization, Supervision, Writing – review & editing.

DATA AVAILABILITY

Data employed in the web server validation is provided in source links as well as supplementary information and on the MarkerML Demo section

Acknowledgements

Authors would like to thank the IT facility and Mr. Amit Khulve, Mr. Tanuj Kumar and Mr. Vikas Pandey of CSIR IGIB for facilitating the hosting of the MarkerML.

SN, RS and SSM are salaried research scientists at TCS Research, Tata Consultancy Services Ltd, India. TCS Research has portfolio of intellectual property in metagenomic marker discovery. SN is an industry sponsored PhD fellow at CSIR-IGIB.

Funding

SN, RS and SSM are salaried research scientists at TCS Research, TCS Research, Tata Consultancy Services Ltd, India. BT is a Senior Principal Scientist at government funded Council of Scientific and Industrial Research – Institute of Genomics and Integrative Biology (CSIR-IGIB), India.

Declaration of Competing Interest

SN, RS and SSM are salaried research scientists at TCS Research, Tata Consultancy Services Ltd, India. TCS Research has portfolio of intellectual property in metagenomic marker discovery. SN is an industry sponsored PhD fellow at CSIR-IGIB.

Appendix A. Supplementary material

Supplementary data to this article can be found online at https://doi.org/10.1016/j.jmb.2022.167589.

Received 30 November 2021;
Accepted 12 April 2022;
Available online 18 April 2022

Keywords:
metagenomic biomarkers;
interpretable machine learning;
SHAP;
microbiome;
marker features

References

1. la Rosa, P.S., Brooks, J.P., Deych, E., Boone, E.L., Edwards, D.J., Wang, Q., Sodergren, E., Weinstock, G., et al., (2012). Hypothesis Testing and Power Calculations for Taxonomic-Based Human Microbiome Data. *PLoS ONE* **7** https://doi.org/10.1371/journal.pone.0052078.
2. Segata, N., Izard, J., Waldron, L., Gevers, D., Miropolsky, L., Garrett, W.S., Huttenhower, C., (2011). Metagenomic biomarker discovery and explanation. *Genome Biol.* **12** https://doi.org/10.1186/gb-2011-12-6-r60.
3. White, J.R., Nagarajan, N., Pop, M., (2009). Statistical methods for detecting differentially abundant features in clinical metagenomic samples. *PLoS Comput. Biol.* **5**, https://doi.org/10.1371/journal.pcbi.1000352 e1000352.
4. Lin, H., das Peddada, S., (2020). Analysis of compositions of microbiomes with bias correction. *Nature Commun.* **11** https://doi.org/10.1038/s41467-020-17041-7.
5. Gloor, G.B., Macklaim, J.M., Pawlowsky-Glahn, V., Egozcue, J.J., (2017). Microbiome datasets are compositional: And this is not optional. *Front. Microbiol.* **8** https://doi.org/10.3389/fmicb.2017.02224.
6. Fernandes, A.D., Reid, J.N.S., Macklaim, J.M., McMurrough, T.A., Edgell, D.R., Gloor, G.B., (2014). Unifying the analysis of high-throughput sequencing datasets: Characterizing RNA-seq, 16S rRNA gene sequencing and selective growth experiments by compositional data analysis. *Microbiome* **2** https://doi.org/10.1186/2049-2618-2-15.
7. Harris, Z.N., Dhungel, E., Mosior, M., Ahn, T.H., (2019). Massive metagenomic data analysis using abundance-based machine learning. *Biol. Direct* **14** https://doi.org/10.1186/s13062-019-0242-0.
8. Feldbauer, R., Schulz, F., Horn, M., Rattei, T., (2015). Prediction of microbial phenotypes based on comparative genomics. *BMC Bioinf.* **16** https://doi.org/10.1186/1471-2105-16-S14-S1.
9. Fang, W., Chang, X., Su, X., Xu, J., Zhang, D., Ning, K., (2012). A machine learning framework of functional biomarker discovery for different microbial communities based on metagenomic data. *In: 2012 IEEE 6th International Conference on Systems Biology, ISB 2012*, pp. 106–112. https://doi.org/10.1109/ISB.2012.6314121.
10. Wang, X., Su, X., Cui, X., Ning, K., (2015). MetaBoot: A machine learning framework of taxonomical biomarker discovery for different microbial communities based on metagenomic data. *PeerJ* **2015**, e993. https://doi.org/10.7717/PEERJ.993/SUPP-1.
11. Alshawaqfeh, M., Bashaireh, A., Serpedin, E., Suchodolski, J., (2017). Consistent metagenomic biomarker detection via robust PCA. *Biol. Direct* **12**, 1–16. https://doi.org/10.1186/S13062-017-0175-4/FIGURES/9.
12. T. Chen, C. Guestrin, XGBoost, in: 2016. Doi: 10.1145/2939672.2939785.
13. Dong, M., Li, L., Chen, M., Kusalik, A., Xu, W., (2020). Predictive analysis methods for human microbiome data with application to Parkinson's disease. *PLoS ONE* **15** https://doi.org/10.1371/journal.pone.0237779.
14. Breiman, L., (2001). Random Forests. *Mach. Learn.* **45** (1), 5–32. https://doi.org/10.1023/A:1010933404324.
15. Zakrzewski, M., Proietti, C., Ellis, J.J., Hasan, S., Brion, M. J., Berger, B., Krause, L., (2017). Calypso: A user-friendly web-server for mining and visualizing microbiome-environment interactions. *Bioinformatics* **33** https://doi.org/10.1093/bioinformatics/btw725.
16. Dhariwal, A., Chong, J., Habib, S., King, I.L., Agellon, L.B., Xia, J., (2017). MicrobiomeAnalyst: A web-based tool for comprehensive statistical, visual and meta-analysis of microbiome data. *Nucleic Acids Res.* **45** https://doi.org/10.1093/nar/gkx295.
17. Ahmad, M.A., Teredesai, A., Eckert, C., (2018). Interpretable machine learning in healthcare. *In: Proceedings – 2018 IEEE International Conference on Healthcare Informatics, ICHI 2018*. https://doi.org/10.1109/ICHI.2018.00095.

18. Molnar, C., Casalicchio, G., Bischl, B., (2020). Interpretable Machine Learning – A Brief History, State-of-the-Art and Challenges. *Commun. Comput. Inf. Sci.*. https://doi.org/10.1007/978-3-030-65965-3_28.
19. Du, M., Liu, N., Hu, X., (2020). Techniques for interpretable machine learning. *Commun. ACM* **63** https://doi.org/10.1145/3359786.
20. Lundberg, S.M., Lee, S.I., (2017). A unified approach to interpreting model predictions. *Adv. Neural Inf. Process. Syst.*.
21. Shapley, L.S., (1951). Notes on the n-Person Game II: The Value of an n-Person Game. Rand Corporation Research Memoranda. RM-670.
22. Marcos-Zambrano, L.J., Karaduzovic-Hadziabdic, K., Loncar Turukalo, T., Przymus, P., Trajkovik, V., Aasmets, O., Berland, M., Gruca, A., et al., (2021). Applications of Machine Learning in Human Microbiome Studies: A Review on Feature Selection, Biomarker Identification, Disease Prediction and Treatment. *Front. Microbiol.* **12** https://doi.org/10.3389/fmicb.2021.634511.
23. Levi, I., Gurevich, M., Perlman, G., Magalashvili, D., Menascu, S., Bar, N., Godneva, A., Zahavi, L., et al., (2021). Potential role of indolelactate and butyrate in multiple sclerosis revealed by integrated microbiome-metabolome analysis. *Cell Rep. Med.* **2** https://doi.org/10.1016/j.xcrm.2021.100246.
24. Balvers, M., Deschasaux, M., van den Born, B.J., Zwinderman, K., Nieuwdorp, M., Levin, E., (2021). Analyzing type 2 diabetes associations with the gut microbiome in individuals from two ethnic backgrounds living in the same geographic area. *Nutrients* **13** https://doi.org/10.3390/nu13093289.
25. Gou, W., Ling, C.-W., He, Y., Jiang, Z., Fu, Y., Fengzhe, X., Miao, Z.-L., Ting-yu, S., et al., (2020). Interpretable Machine Learning Algorithm Reveals Novel Gut Microbiome Features in Predicting Type 2 Diabetes. *Curr. Dev. Nutrit.* **4** https://doi.org/10.1093/cdn/nzaa062_016.
26. Wong, C.W., Yost, S.E., Lee, J.S., Gillece, J.D., Folkerts, M., Reining, L., Highlander, S.K., Eftekhari, Z., et al., (2021). Analysis of Gut Microbiome Using Explainable Machine Learning Predicts Risk of Diarrhea Associated With Tyrosine Kinase Inhibitor Neratinib: A Pilot Study. *Front. Oncol.* **11** https://doi.org/10.3389/fonc.2021.604584.
27. Gou, W., Ling, C.W., He, Y., Jiang, Z., Fu, Y., Xu, F., Miao, Z., Sun, T.Y., et al., (2021). Interpretable machine learning framework reveals robust gut microbiome features associated with type 2 diabetes. *Diabetes Care* **44** https://doi.org/10.2337/dc20-1536.
28. Carrieri, A.P., Haiminen, N., Maudsley-Barton, S., Gardiner, L.J., Murphy, B., Mayes, A.E., Paterson, S., Grimshaw, S., et al., (2021). Explainable AI reveals changes in skin microbiome composition linked to phenotypic differences. *Sci. Rep.* **11** https://doi.org/10.1038/s41598-021-83922-6.
29. Arndt, D., Xia, J., Liu, Y., Zhou, Y., Guo, A.C., Cruz, J.A., Sinelnikov, I., Budwill, K., et al., (2012). METAGENassist: A comprehensive web server for comparative metagenomics. *Nucleic Acids Res.* **40** https://doi.org/10.1093/nar/gks497.
30. Wilke, A., Bischof, J., Gerlach, W., Glass, E., Harrison, T., Keegan, K.P., Paczian, T., Trimble, W.L., et al., (2016). The MG-RAST metagenomics database and portal in 2015. *Nucleic Acids Res.* **44** https://doi.org/10.1093/nar/gkv1322.
31. Cole, J.R., Wang, Q., Fish, J.A., Chai, B., McGarrell, D.M., Sun, Y., Brown, C.T., Porras-Alfaro, A., et al., (2014). Ribosomal Database Project: Data and tools for high throughput rRNA analysis. *Nucleic Acids Res.* **42** https://doi.org/10.1093/nar/gkt1244.
32. Kanehisa, M., Furumichi, M., Sato, Y., Ishiguro-Watanabe, M., Tanabe, M., (2021). KEGG: Integrating viruses and cellular organisms. *Nucleic Acids Res.* **49** https://doi.org/10.1093/nar/gkaa970.
33. Galperin, M.Y., Wolf, Y.I., Makarova, K.S., Alvarez, R.V., Landsman, D., Koonin, E.V., (2021). COG database update: Focus on microbial diversity, model organisms, and widespread pathogens. *Nucleic Acids Res.* **49** https://doi.org/10.1093/nar/gkaa1018.
34. Mistry, J., Chuguransky, S., Williams, L., Qureshi, M., Salazar, G.A., Sonnhammer, E.L.L., Tosatto, S.C.E., Paladin, L., et al., (2021). Pfam: The protein families database in 2021. *Nucleic Acids Res.* **49** https://doi.org/10.1093/nar/gkaa913.
35. Li, W., O'Neill, K.R., Haft, D.H., Dicuccio, M., Chetvernin, V., Badretdin, A., Coulouris, G., Chitsaz, F., et al., (2021). RefSeq: Expanding the Prokaryotic Genome Annotation Pipeline reach with protein family model curation. *Nucleic Acids Res.* **49** https://doi.org/10.1093/nar/gkaa1105.
36. Walsh, I., Fishman, D., Garcia-Gasulla, D., Titma, T., Pollastri, G., Capriotti, E., Casadio, R., Capella-Gutierrez, S., et al., (2021). DOME: recommendations for supervised machine learning validation in biology. *Nature Methods*. https://doi.org/10.1038/s41592-021-01205-4.
37. Collins, G.S., Reitsma, J.B., Altman, D.G., Moons, K.G.M., (2015). Transparent reporting of a multivariable prediction model for individual prognosis or diagnosis (TRIPOD): The TRIPOD Statement. *Eur. Urol.* **67** https://doi.org/10.1016/j.eururo.2014.11.025.
38. Sperandei, S., (2014). Understanding logistic regression analysis. *Biochemia Medica* **24** https://doi.org/10.11613/BM.2014.003.
39. W. McKinney, Pandas: a Foundational Python Library for Data Analysis and Statistics, Python for High Performance and Scientific Computing. (2011).
40. Pedregosa, F., Varoquaux, G., Gramfort, A., Michel, V., Thirion, B., Grisel, O., Blondel, M., Prettenhofer, P., et al., (2011). Scikit-learn: Machine Learning in Python. *J. Mach. Learn. Res.* **12**, 2825–2830.
41. Asnicar, F., Weingart, G., Tickle, T.L., Huttenhower, C., Segata, N., (2015). Compact graphical representation of phylogenetic data and metadata with GraPhlAn. *PeerJ* **2015** https://doi.org/10.7717/peerj.1029.
42. Bengfort, B., Bilbro, R., (2019). Yellowbrick: Visualizing the Scikit-Learn Model Selection Process. *J. Open Source Softw.* **4** https://doi.org/10.21105/joss.01075.
43. Nagpal, S., Haque, M.M., Singh, R., Mande, S.S., (2019). IVikodak-A platform and standard workflow for inferring, analyzing, comparing, and visualizing the functional potential of microbial communities. *Front. Microbiol.* **10** https://doi.org/10.3389/fmicb.2018.03336.
44. Gupta, M.R., Bengio, S., Weston, J., (2014). Training highly multiclass classifiers. *J. Mach. Learn. Res.* **15**
45. Nagpal, S., Pinna, N.K., Srivastava, D., Singh, R., Mande, S.S., (2021). (Machine) Learning the mutation signatures of SARS-CoV-2: a primer for predictive prognosis. *BioRxiv*.
46. Messalas, A., Kanellopoulos, Y., Makris, C., (2019). Model-Agnostic Interpretability with Shapley Values. *In: 10th International Conference on Information, Intelligence,*

Systems and Applications, IISA 2019. https://doi.org/10.1109/IISA.2019.8900669.

47. Griffen, A.L., Beall, C.J., Campbell, J.H., Firestone, N.D., Kumar, P.S., Yang, Z.K., Podar, M., Leys, E.J., (2012). Distinct and complex bacterial profiles in human periodontitis and health revealed by 16S pyrosequencing. *ISME J.* **6** https://doi.org/10.1038/ismej.2011.191.
48. Mitchell, A.L., Almeida, A., Beracochea, M., Boland, M., Burgin, J., Cochrane, G., Crusoe, M.R., Kale, V., et al., (2020). MGnify: The microbiome analysis resource in 2020. *Nucleic Acids Res.* **48** https://doi.org/10.1093/nar/gkz1035.
49. Huang, S., He, T., Yue, F., Xu, X., Wang, L., Zhu, P., Teng, F., Sun, Z., et al., (2021). Longitudinal multi-omics and microbiome meta-analysis identify an asymptomatic gingival state that links gingivitis, periodontitis, and aging. *MBio.* **12** https://doi.org/10.1128/mBio.03281-20.
50. Fernández-Edreira, D., Liñares-Blanco, J., Fernandez-Lozano, C., (2021). Machine Learning analysis of the human infant gut microbiome identifies influential species in type 1 diabetes. *Expert Syst. Appl.* **185** https://doi.org/10.1016/j.eswa.2021.115648.
51. Fusco, V., Quero, G.M., Cho, G.S., Kabisch, J., Meske, D., Neve, H., Bockelmann, W., Franz, C.M.A.P., (2015). The genus Weissella: Taxonomy, ecology and biotechnological potential. *Front. Microbiol.* **6** https://doi.org/10.3389/fmicb.2015.00155.
52. Ma, S., Ren, B., Mallick, H., Moon, Y.S., Schwager, E., Maharjan, S., Tickle, T.L., Lu, Y., et al., (2021). A statistical model for describing and simulating microbial community profiles. *PLoS Comput. Biol.* **17** https://doi.org/10.1371/JOURNAL.PCBI.1008913.
53. Sazal, M., Stebliankin, V., Mathee, K., Yoo, C., Narasimhan, G., (2021). Causal effects in microbiomes using interventional calculus. *Sci. Rep.* **11** https://doi.org/10.1038/s41598-021-84905-3.
54. Prosperi, M., Guo, Y., Sperrin, M., Koopman, J.S., Min, J. S., He, X., Rich, S., Wang, M., et al., (2020). Causal inference and counterfactual prediction in machine learning for actionable healthcare. *Nature Mach. Intell.* **2** https://doi.org/10.1038/s42256-020-0197-y.
