# Supplementary material for: PDBx/mmCIF Ecosystem: Foundational Semantic Tools for Structural Biology
Source: J Mol Biol. Author manuscript; Available in PMC 2023 Jun 26. (PMC10292674; doi:10.1016/j.jmb.2022.167599)
Supplement: Article [file NIHMS1907597-supplement-Article.zip › THRONE--A-New-Approach-for-Accurate-Prediction-of-Hum_2022_Journal-of-Molecu.pdf]

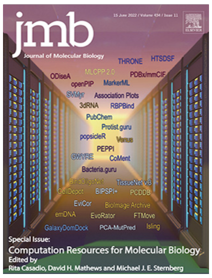

THRONE: A New Approach for Accurate Prediction of Human RNA N7-Methylguanosine Sites

Watshara Shoombuatong [1†], Shaherin Basith [2,3†], Thejkiran Pitti [4], Gwang Lee [2,3*] and Balachandran Manavalan [2,4*]

1 - ***Center of Data Mining and Biomedical Informatics,*** *Faculty of Medical Technology, Mahidol University, Bangkok 10700, Thailand*

2 - ***Department of Physiology,*** *Ajou University School of Medicine, Suwon 16499, Republic of Korea*

3 - ***Department of Molecular Science and Technology,*** *Ajou University, Suwon 16499, Republic of Korea*

4 - ***Computational Biology and Bioinformatics Laboratory,*** *Department of Integrative Biotechnology, College of Biotechnology and Bioengineering, Sungkyunkwan University, Suwon 16419, Gyeonggi-do, Republic of Korea*

Correspondence to Gwang Lee and Balachandran Manavalan: *glee@ajou.ac.kr (G. Lee), bala2022@skku.edu (B. Manavalan), @bala_CBB (B. Manavalan)*
https://doi.org/10.1016/j.jmb.2022.167549
Edited by David Mathews

Abstract

N^7-methylguanosine (m7G) is an essential, ubiquitous, and positively charged modification at the 5′ cap of eukaryotic mRNA, modulating its export, translation, and splicing processes. Although several machine learning (ML)-based computational predictors for m7G have been developed, all utilized specific computational framework. This study is the first instance we explored four different computational frameworks and identified the best approach. Based on that we developed a novel predictor, THRONE (A **t**hree-layer ensemble predictor for identifying **h**uman **R**NA N7-methylguan**o**si**n**e sit**e**s) to accurately identify m7G sites from the human genome. THRONE employs a wide range of sequence-based features inputted to several ML classifiers and combines these models through ensemble learning. The three-step ensemble learning is as follows: 54 baseline models were constructed in the first layer and the predicted probability of m7G was considered as a new feature vector for the sequential step. Subsequently, six meta-models were created using the new feature vector and their predicted probability was yet again considered as novel features. Finally, random forest was deemed as the best super classifier learner for the final prediction using a systematic approach incorporated with novel features. Interestingly, THRONE outperformed other existing methods in the prediction of m7G sites on both cross-validation analysis and independent evaluation. The proposed method is publicly accessible at: http://thegleelab.org/THRONE/ and expects to help the scientific community identify the putative m7G sites and formulate a novel testable biological hypothesis.

© **2022 Elsevier Ltd. All rights reserved.**

Introduction

N^7-methylguanosine (m7G) has been recognized as a ubiquitous post-transcriptional RNA modification.[1] During transcription initiation, a methyl group is added at the N^7 position of riboguanosine.[2] The modification of m7G at the 5′ cap catalyzed by methyltransferase co-transcriptionally results in a positively charged RNA modification.[3–5] The presence of this modification in transfer RNA (tRNA) variable loop, eukaryotic S ribosomal RNA (rRNA) and internal

0022-2836/© 2022 Elsevier Ltd. All rights reserved.

messenger RNA (mRNA) indicates its involvement in various biological processes,[5] including gene expression, RNA processing and metabolism, stability of transcripts, protein synthesis, and cell viability.[1,4,6] Every phase of mRNA life cycle, including transcription elongation, mRNA splicing, polyadenylation, and nuclear export are regulated by m7G modification.[7,8]

Besides its involvement in various biological functions, m7G modification is also associated with several diseases, such as growth deficiency,[9] microcephalic primordial dwarfism, brain malformation, and development of specific autoimmune disorders.[10,11] Due to its importance in regulation, numerous biological processes, and connection with several diseases, accurate analysis of m7G distribution is necessary for a comprehensive understanding of its mechanism and biological functions. Several experimental methods have been used to identify m7G sites, including AlkAniline-seq, MeRIP-seq, chemical-assisted m7G -seq, and miCLIP-seq.[12–14] Even though these experimental methods could accurately identify m7G sites, they seem intricate, laborious, and cost-ineffective for performing transcriptome-wide detections. Hence, there is a pressing need to develop effective computational approaches for the accurate identification of m7G sites.

Recent advances in computational approaches has given rise to numerous machine learning (ML)-based prediction models to identify m7G sites from the human RNA sequences. Chen et al.[15] developed the first ML method namely, iRNA-m7G[15] using feature fusion strategy incorporated into support vector machine (SVM) classifier. After that, several methods have been proposed, m7GFinder,[16] Yang et al.[17] standalone m7G model, m7Gpredictor,[18] XG-m7G,[19] m7G-IFL,[20] m7G-DLSTM,[21] BERT-m7G.[22] Among these methods, iRNA-m7G, XG-m7G, and m7G-IFL are publicly available predictors. Notably, all these methods contributed to the progress of post-transcriptional epigenetic modification research. Unlike previous methods that employed direct computational approach, we attempted to explore four different frameworks, including two variants of stacking frameworks[23,24] iterative feature representation[25,26] and a novel three-layer ensemble predictor for m7G prediction.

We present THRONE (A **t**hree-layer ensemble predictor for identifying **h**uman **R**NA N7-methylguan**o**si**n**e sit**e**s) to accurately identify m7G sites from the human genome, whose overall framework is shown in Figure 1. THRONE employs a wide range of sequence-based features inputted to several ML classifiers and combines these models through three-layer ensemble learning. The three layers involve the following steps: (i) We constructed 54 baseline models by employing nine different encodings and six different classifiers. Then, the output of baseline models (predicted probability of m7Gs) were combined into a 54-D probabilistic feature vector; (ii) Subsequently, six classifiers were trained with 54-D feature vectors and developed their respective meta-models, whose predicted probability values are integrated to generate 6D novel features; (iii) Lastly, six classifiers were trained with 6D novel features and developed their respective super learner. The performance comparison shows that the RF classifier is marginally superior; hence, we selected it for the final prediction. Gaining from the three-layer ensemble learning, THRONE performed better than the two stacking approaches and iterative feature representation (IFR) employed in this study. Furthermore, THRONE outperformed existing state-of-the-art predictors for m7G prediction on both cross-validation analysis and independent evaluation. Thus, we expect that our proposed method will allow effective and accurate screening of putative m7Gs, thereby expediting experimental validation for unraveling their functional mechanisms in the future.

Materials and Methods

Dataset and feature encodings

A high-quality training dataset is required to develop a prediction model. This paper employed the same training dataset proposed in the previous work,[15] considered the standard training dataset, and used it to construct existing predictors. The non-redundant dataset contains 741 m7G and 741 non-m7G samples. Notably, m7G validated experimentally derived from human cell lines (HeLa and HepG2 cells), including 41 nucleotides with 20 upstream and 20 downstream, and the modified site at the center. However, the negative samples containing guanosine at the center with 41 nucleotides derived from the human genome and not detected by the MeRIP-seq method, which resulted in a massive number. Therefore, to avoid potential bias (high specificity (Sp) and low sensitivity (Sn)) caused by the imbalanced data, they selected 741 sequences that shared <80% sequence similarity and were considered non-m7G. Hence, developing a prediction model using such high-quality data fairly compares our prediction model with the existing predictors.

Since the existing methods did not evaluate with an independent dataset, it is essential to construct such a dataset to check the trained model's robustness. In this regard, we constructed an independent dataset with the following procedure. Firstly, m7G sequences were downloaded from m6A-Atlas[27] and considered only HeLa cells sequences. Subsequently, we excluded the sequences that shared 80% sequence identity with our training dataset, resulting in 334 m7G sequences. Secondly, negative samples were con-

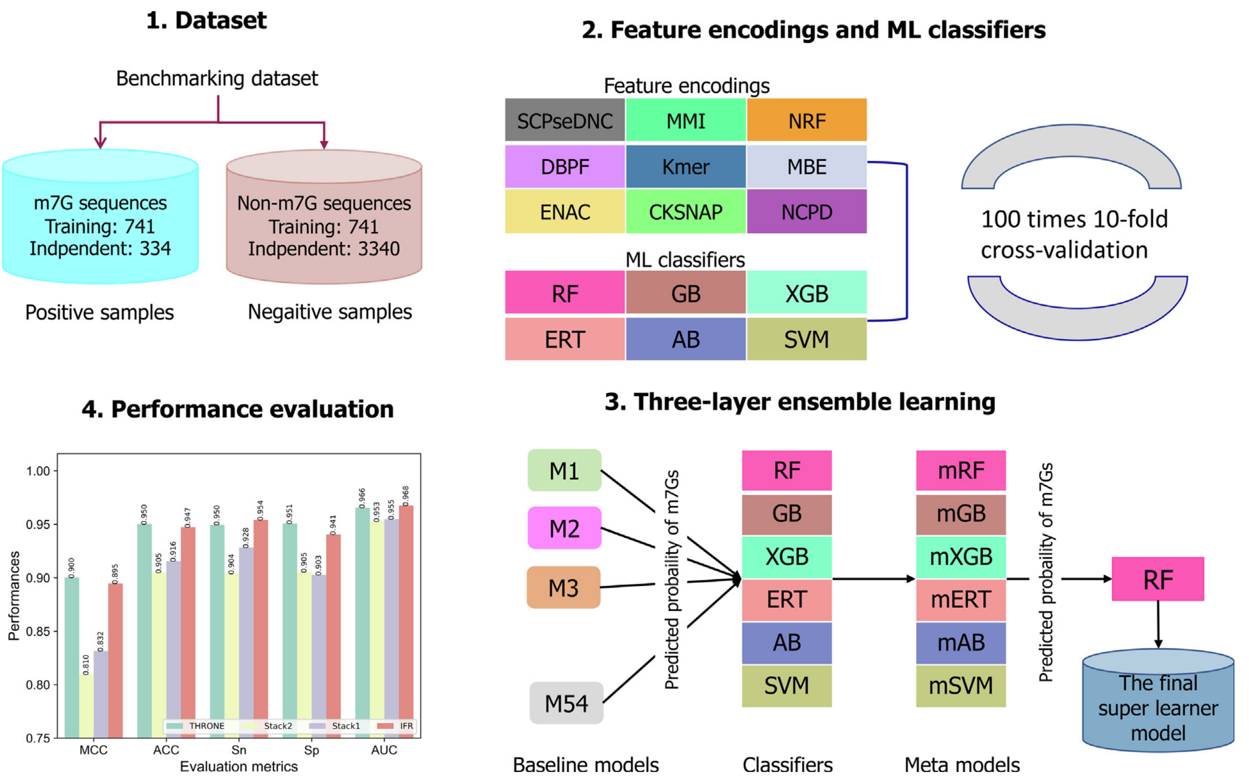

Figure 1. An Overview of THRONE framework for predicting m7G sites. Schematic display of the four stages in the construction of THRONE is shown.

structed according to the Chen et al. procedure and randomly selected 3340 sequences.[15]

Here, we employed nine different encodings [mononucleotide binary encoding (MBE), NCPD is a combination of nucleotide chemical property and nucleotide density, DBPF is a combination of dinucleotide binary profile and dinucleotide frequency, essential nucleic acid composition (ENAC), numerical representation features (NRF), the composition of *K*-spaced nucleic acid pairs (CKSNAP), integration of Kmer composition (Kmer), series correlation pseudo-dinucleotide composition (SCPseDNC), and maximum mutual information (MMI)] and six ML classifiers [random forest (RF), SVM, extremely randomized tree (ERT), gradient boosting (GB), AdaBoost (AB), and eXtreme GB (XGB)]. A detailed description of feature encodings, conventional classifiers, cross-validation and performance evaluation metrics is provided in the supplementary information under the 'Materials and Methods' section.

Results and Discussion

Performance evaluation of six different classifiers with respect to nine encodings based on 100-times 10-fold cross-validation

This study employed nine encodings that extracted features from sequence information in different perspectives and six different classifiers and evaluated their potential role in classifying m7Gs from non-m7Gs. Generally, a one-time run of the k-fold cross-validation on the training dataset may result in a noisy estimation of model performance because different partitions of the training data may result in varied results.[28] However, repeated k-fold cross-validation provides a way to improve the estimated performance of an ML model,[29] but it is computationally expensive. To this end, we opted for a computationally expensive procedure and estimated each feature-based specific classifier model (single feature encoding (SF) model) using a 100-times 10-fold cross-validation test on the training dataset, whose average performance and standard deviation are shown in Figure S1.

We found a similar overall performance pattern for nine SF models for three classifiers (RF, ERT, and GB). For the sake of clarity, we grouped these SF model performances in terms of Matthews correlation coefficient (MCC) into three categories (C1: {MBE, DBPF, and NCPD}; C2: {ENAC, Kmer, CKSNAP, and NRF}; and C3: {SCPseDNC and MMI}), where we noticed that C1 achieved similar performances, which is slightly superior to C2 and significantly better than C3. Furthermore, in the case of XGB, except for MMI, the rest of the eight SF models achieved similar performances. Whereas for the remaining two classifiers (SVM and AB), nine SF model

performances are incredibly varied, and still it is possible to cluster them into three groups, but the members between three groups varied between SVM and AB, and also different from three classifiers (RF, ERT, and GB) mentioned above. Overall, we observed the following perspectives: (i) the MBE-based model consistently achieved the best performance regardless of the classifiers. (ii) the same feature (e.g., CKSNAP) with the variation in performance for different classifiers, emphasizing the importance of exploiting multiple classifiers during model building. (iii) members in C3 groups achieved an MCC of ∼0.6 regardless of classifiers, indicating that they also have a reasonable discriminative capability.

Construction of THRONE

We employed the three-layer approach to construct the THRONE. Firstly, we considered all 54 SF models and collectively called baseline models or layer1 models. Although several ways to integrate baseline models were reported in the literature,[30–33] we employed the meta-predictor approach.[34,35] Briefly, we obtained the output (predicted probability of m7G) of these layer1 models and integrated a 54-D feature vector. Subsequently, six classifiers were trained with 54-D feature vector independently and developed their respective meta-models (collectively called layer2 models) using 100-times 10-fold cross-validation (Figure 2(A)). The performance comparison analysis shows that meta(m)AB and mSVM achieved a similar performance, which is better than the remaining four meta classifiers (mRF, mERT, mGB, and mXGB).

Instead of selecting best model from the meta classifiers, we computed the predicted probability of m7G from six meta-classifier and integrated a 6D novel feature vector as an input to the development of layer3 models. Then, utilizing the exact repeated cross-validation techniques, we trained six classifiers and developed their respective super learner model. Figure 2(B) shows that all super classifier learners achieved similar performances, and super learner (s)RF is marginally better MCC than other models. Hence, we selected sRF as the final model and named it THRONE. THRONE achieved MCC, accuracy (ACC), Sn, Sp, the area under ROC curve (AUC) of 0.900, 0.950, 0.950, 0.951, 0.966, respectively. Overall, THRONE improvement is significant compared to the layer2 and layer1 models. Specifically, THRONE improvement is 4.0–5.5% in MCC, 2.0–2.8% in ACC, 1.7–2.6% in Sn, 1.5–3.8% in Sp compared to layer2 models. Compared to 53 SF models (excluded an outlier), THRONE improvement is 8.89–33.9% in MCC and 4.45–17.06% in ACC. The comparative analysis demonstrated that the three-layer approach significantly improved the prediction performance by integrating layer1 and layer2 models.

Construction of models using different approaches

We also employed two different versions of the stacking framework and the IFR, apart from the THRONE framework. A brief description of each framework construction and its performance is as follows:

(i) Stack1: Inspired by the recent methods STALLION[23] and ACPredStackL,[36] we developed the first stacking model with a similar approach. Firstly, we concatenated all nine feature encodings, generated hybrid features containing 1634D, input them into six classifiers independently, and developed their respective models. As shown in Figure S2(A), AB and XGB achieved a similar performance with an MCC range of 0.826–0.830 and significantly better than the remaining four classifiers. Notably, the hybrid feature dimension is too large and may contain overlapping and unnecessary information, which affects the model performance. Hence, we applied a two-step feature selection technique, including ranking features and sequential forward search (SFS), to exclude less informative features from hybrid features. Generally, feature ranking methods (RF and F-score) assign a score for all given features.[25,37] However, XGB assigns a score only to the critical features and excludes most features by assigning zero.[23] Here, we applied the XGB classifier, selected 448 features, and sorted them according to their rank. Subsequently, SFS was applied on 448 features with the step size of two features, whose performance is shown in Figure S2(B). For four classifiers (RF, ERT, GB, and SVM), the performance is steadily increased and reached the maximum ACC and declined performance as the features kept added. Whereas in the case of SVM and AB, after reaching the maximum ACC, the performance remains in equilibrium. Interestingly, RF, ERT, GB, AB, SVM, and XGB achieved their best performance with 36, 48, 34, 388, 106, and 392-D optimal feature sets (Figure S2(B)). The performance comparison among six classifiers shows that again AB is superior (Figure S2(C)). Then, the predicted probability values of m7G from six classifiers were concatenated and trained with six different classifiers separately and recorded performance (Figure S2(D)). The result shows that five classifiers achieved a similar performance except for GB, and we selected AB as the final model for Stack1 due to its consistent performance in all steps. Furthermore, the stacking approach for each classifier significantly improves their model compared to the hybrid features.

(ii) Stack2: Inspired by the recent method Porpoise,[24] we developed the second stacking model with a similar approach. Firstly, we selected the best SF model for each classifier from Figure S1. Notably, MBE encoding achieved the best performance for all six classifiers. Secondly, the predicted probability of m7Gs from MBE-based six classifiers

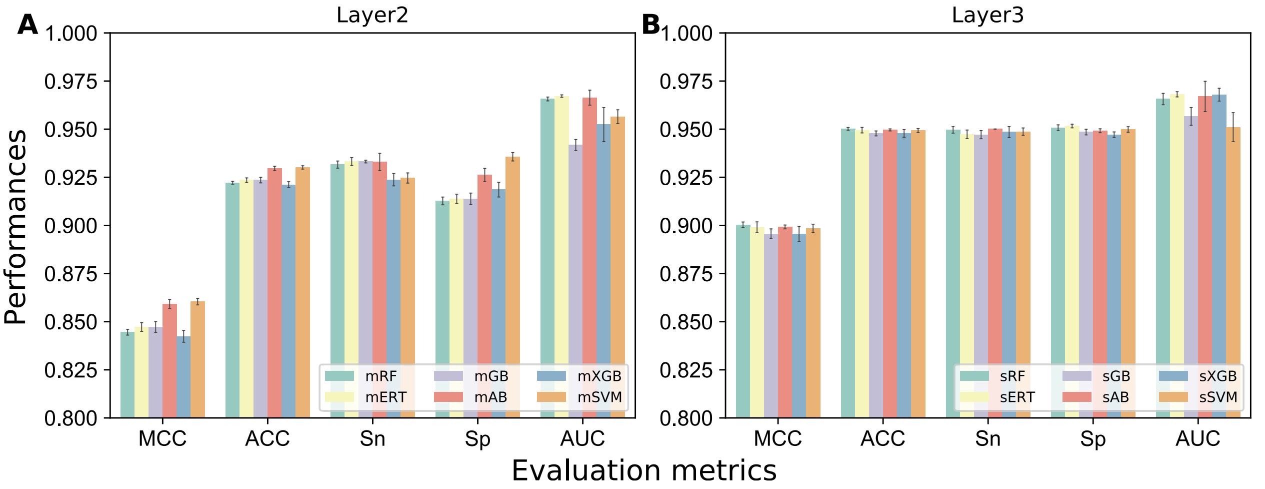

Figure 2. Performance comparison of different classifiers on layer2 and layer3 of three-layer ensemble learning. Notably, we presented the average value and standard deviation (error bar) for each metric. (A) Performances of six classifiers based on the 54D probabilistic features obtained from the baseline models. (B) Performances of six classifiers based on the 6D probabilistic features obtained from the meta-classifier.

is computed and integrated as a new feature, again trained with all six classifiers independently and compared their performances. Figure S3 shows that the AB-based model achieved superior performance with MCC, ACC, Sn, Sp, and AUC of 0.810, 0.905, 0.904, 0.906, and 0.953. Specifically, the ACC improvement of AB is 0.28–1.03% higher compared to the other classifiers. Hence, we selected the AB-based model for Stack2.

(iii) IFR: Stacking models of each classifier obtained from Figure S2(D) and employed IFR with the identical procedure employed in previous studies.[20,25] Notably, each model has 6D input features, and the predicted probability of m7G from the trained model was incorporated into 6D features and obtained 7D features before the first round of the iteration process. We repeated this procedure 20 times, and the respective performance is shown in Figure S4(A). The result shows that all classifier performance gradually increases and reaches the maximum ACC after the 12th iteration and remains the plateau (except SVM). Next, we compared the performances among the best model for each classifier obtained from IFR. The result shows that four classifiers (RF, ERT, GB, and SVM) performances are similar with MCC in the range of 0.893–0.894 and ACC of 0.947, slightly superior to the AB and XGB (Figure S4(B)). However, we selected the RF-based model for IFR because it is fractionally superior to other classifiers in terms of MCC.

Comparison of THRONE performances with other approaches and the existing predictors on training dataset

Before comparing THRONE performance with the existing predictors, we compared it with three other approaches (Stack1, Stack2, and IFR) investigated in this study (Figure 3(A)). Among the three approaches, the IFR achieved the best performance in all five metrics. Specifically, IFR achieved 6.5–8.32% higher in MCC and 3–4.2% higher in ACC. However, IFR is very competitive compared to THRONE and has marginally lower performance in global metrics, including MCC (0.895 Vs. 0.900) and ACC (0.947 Vs. 0.950). As the THRONE achieves the best performance among the different approaches employed in this study, we selected this as the final predictor for m7G prediction. Our result demonstrated that the advantage of exploiting multiple computational approaches on the same dataset is that one can understand the pros and cons of each approach, which can ultimately lead to the selection of the best approach/model.

Secondly, we compared the THRONE with the publicly available three state-of-the-art predictors, namely iRNA-m7G, XG-m7G, and m7G-IFL. Notably, all the existing methods and the current approach developed utilizing the same training dataset. Therefore, a performance comparison between these methods is fair and will give an overview to the experimentalists to select the appropriate tool. Figure 3(B) shows that the THRONE improvement is significant in four out of five metrics. Specifically, THRONE achieved 5–10.0% higher in MCC, 2.5–5.2% higher in ACC, 2.6–6.3% higher in Sn, 2.6–4.0% higher in Sp than the existing methods. In the AUC, THRONE is similar to the XG-m7G and better than the rest of the existing predictors.

Performance comparison of THRONE and the existing methods on independent dataset

Unlike the existing methods, we evaluated THRONE along with the existing methods on the independent datasets. Table 1 shows that

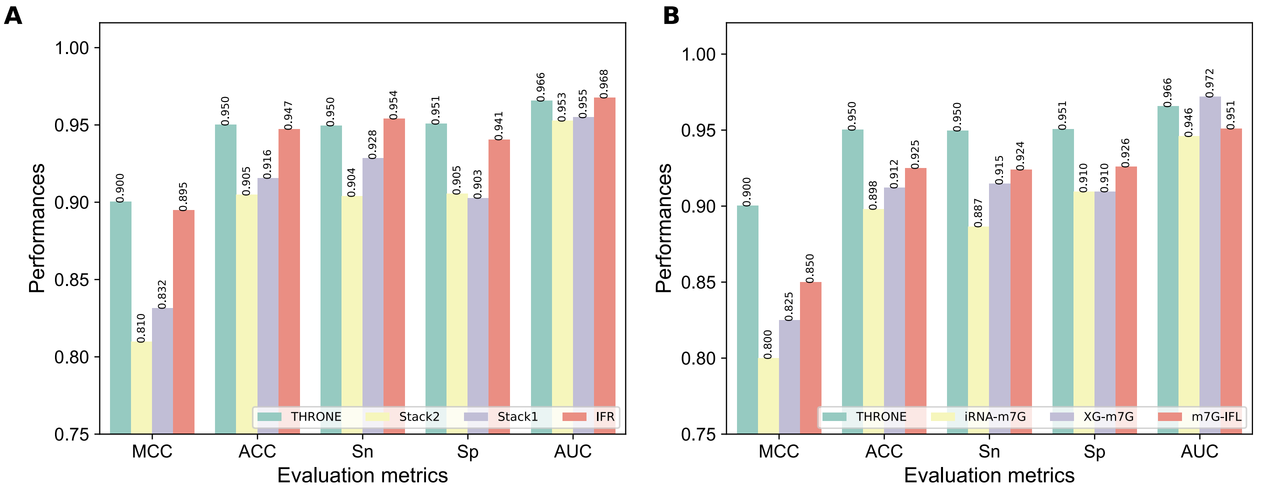

Figure 3. Performance comparison of THRONE with the different approaches and the existing methods. (A) THRONE with the other computational frameworks employed here, (B) THRONE with the state-of-the-art methods.

THRONE achieved MCC, ACC, Sn, Sp, and AUC of 0.568, 0.886, 0.877, 0.887, and 0.871, respectively. As the evaluation dataset is imbalanced, the MCC metric is appropriate to compare the two methods. Specifically, THRONE improvement is 19.0–27.3% in MCC compared to the iRNA-m7G and XG-m7G. Among the existing methods, the performance of m7G-IFL is inferior to random predictions. It is likely that the predictor is overoptimized during the training. Table 1 also includes the performance of our other three approaches, where the two stack models showed superior performance compared to the existing predictors and lower than the THRONE.

The performance of THRONE was considerably improved in both cross-validation and independent evaluation, indicating that it has a strong generalization ability. The improvement of THRONE is mainly attributed to the following reasons: (i) utilization of different feature encodings and multiple classifiers; (ii) three-layer ensemble learning; (iii) exploitation of multiple computational frameworks and the selection of the best approach.

Table 1 Performance comparison of different methods on the independent dataset.

Methods	MCC	ACC	Sn	Sp	AUC
THRONE	0.568	0.886	0.877	0.887	0.871
iRNA-m7G	0.371	0.849	0.614	0.872	NA
XG-m7G	0.288	0.809	0.572	0.833	0.802
m7G-IFL	−0.188	0.324	0.368	0.320	0.329
Stack2	0.385	0.851	0.634	0.872	0.859
Stack1	0.516	0.860	0.868	0.859	0.889
IFR	0.356	0.802	0.716	0.811	0.837

Note: iRNA-m7G predictor provides the probability score only for m7G site prediction, therefore AUC values cannot be calculated. Hence, it is mentioned as not available (NA). Italicized methods represent different approaches used in this study.

Visualization of feature representations and analysis

The t-distributed stochastic neighbor embedding (t-SNE) plot was employed to visualize the feature representation map in a 2D space from which THRONE automatically learned. Figure S5(A) shows the representation map for original hybrid features (L1) (linear concatenation of nine different encodings), where we observed significant overlap between the m7Gs and non-m7Gs features. Compared to the original features, the predicted probability features (L2) obtained from the baseline models improve the feature representation capability (Figure S5(B)). There is still overlap between m7G and non-m7G. Figure S5(C) shows that the predicted probability (L3) of the meta-classifier significantly improves the ability to discriminative m7G and non-m7G. As a result, two separate clusters could be well distinguished. These results suggest that THRONE could learn good feature representations in a stepwise manner for classifying m7Gs effectively. Shapley Additive explanation (SHAP) analysis was conducted on L2 and L3 probabilistic features to obtain an overview of each feature (Figure S6), the results are presented in the supplementary information.

Webserver implementation

We implemented THRONE as an online webserver to facilitate a community-wide effort to predict m7Gs in a high-throughput and cost-effective manner. THRONE is freely accessible at https://thegleelab.org/THRONE. To submit a job to the THRONE web server, we provided step-by-step instructions: (i) the users either paste their

sequences in the text area or upload the file. Most importantly, the sequences should be in FASTA format. (ii) the users have to click the 'Submit' button to submit the job after completing the first step. (iii) once the calculation is completed, the users can view the prediction results. Moreover, the curated benchmark dataset employed in this study can be downloaded from the THRONE web server.

Conclusion

Several interesting computational frameworks have been reported identifying the sequence-based function predictions in the last three years.[38–42] Most of these frameworks rely on feature engineering, multiple classifiers' implementation, reducing original feature dimension into multiview information, and the integration of multiple models. Consequently, recent studies suggested the importance of exploring multiple frameworks on the same dataset or problem and identify the best one rather than using the specific framework.[43,44] In this regard, we exploited four different computational frameworks in m7G predictions, including two variants of the stacking frameworks, IFR, and a new three-layer ensemble learning approach. Based on the best-performing approach, we reported a novel predictor termed THRONE to identify human m5C sites. The extensive benchmarking test demonstrated that THRONE represents a comprehensive, state-of-the-art predictor, which has outperformed all other existing methods for m7G prediction. The proposed approach can be applied to develop novel bioinformatics tools for other sequence-based function predictions, including post replication, transcription, and translational events of biomolecules.[45–48] Furthermore, we expect that our THRONE methodology and online web server will facilitate the detection of putative m7G sites from the human genome and understand their role through functional characterization.

Acknowledgments

This work is supported by the National Research Foundation of Korea (NRF) funded by the Korean government (MSIT) (2021R1A2C1014338 and 2020R1A4A4079722)

Author contributions

B.M. conceived the project and designed the experiments. B.M., G.L., W.S., and S.B., performed the experiments and analyzed the data, and B.M, S.B., G.L., and T.P., wrote the manuscript. All authors read and approved the final manuscript.

Conflict of Interest

The authors declare no competing interests.

Appendix A. Supplementary material

Supplementary data to this article can be found online at https://doi.org/10.1016/j.jmb.2022.167549.

Received 16 November 2021;
Accepted 10 March 2022;
Available online 16 March 2022

Keywords:
RNA N7-methylguanosine sites;
sequence analysis;
bioinformatics;
ensemble learning;
machine learning

† Equally contributed.

References

1. Cole, M.D., Cowling, V.H., (2009). Specific regulation of mRNA cap methylation by the c-Myc and E2F1 transcription factors. *Oncogene* **28**, 1169–1175.
2. Komal, S., Zhang, L.R., Han, S.N., (2021). Potential regulatory role of epigenetic RNA methylation in cardiovascular diseases. *Biomed. Pharmacother.* **137**, 111376
3. Cowling, V.H., (2009). Regulation of mRNA cap methylation. *Biochem. J.* **425**, 295–302.
4. Furuichi, Y., (2015). Discovery of m(7)G-cap in eukaryotic mRNAs. *Proc. Jpn. Acad. Ser. B Phys. Biol. Sci.* **91**, 394–409.
5. Wu, Y., Zhan, S., Xu, Y., Gao, X., (2021). RNA modifications in cardiovascular diseases, the potential therapeutic targets. *Life Sci.* **278**, 119565
6. Pandolfini, L., Barbieri, I., Bannister, A.J., Hendrick, A., Andrews, B., Webster, N., et al., (2019). METTL1 Promotes let-7 MicroRNA Processing via m7G Methylation. *Mol. Cell.* **74**, (1278–90) e9
7. Konarska, M.M., Padgett, R.A., Sharp, P.A., (1984). Recognition of cap structure in splicing in vitro of mRNA precursors. *Cell* **38**, 731–736.
8. Lewis, J.D., Izaurralde, E., (1997). The role of the cap structure in RNA processing and nuclear export. *Eur. J. Biochem.* **247**, 461–469.
9. Alexandrov, A., Grayhack, E.J., Phizicky, E.M., (2005). tRNA m7G methyltransferase Trm8p/Trm82p: evidence linking activity to a growth phenotype and implicating Trm82p in maintaining levels of active Trm8p. *RNA* **11**, 821–830.
10. Lin, S., Liu, Q., Lelyveld, V.S., Choe, J., Szostak, J.W., Gregory, R.I., (2018). Mettl1/Wdr4-Mediated m(7)G tRNA Methylome Is Required for Normal mRNA Translation and Embryonic Stem Cell Self-Renewal and Differentiation. *Mol. Cell.* **71** 244–55 e5.
11. Pereira, P.L., Magnol, L., Sahun, I., Brault, V., Duchon, A., Prandini, P., et al., (2009). A new mouse model for the

trisomy of the Abcg1-U2af1 region reveals the complexity of the combinatorial genetic code of down syndrome. *Hum. Mol. Genet.* **18**, 4756–4769.
12. Marchand, V., Ayadi, L., Ernst, F.G.M., Hertler, J., Bourguignon-Igel, V., Galvanin, A., et al., (2018). AlkAniline-Seq: Profiling of m(7) G and m(3) C RNA Modifications at Single Nucleotide Resolution. *Angew. Chem. Int. Ed. Engl.* **57**, 16785–16790.
13. Zhang, L.S., Liu, C., Ma, H., Dai, Q., Sun, H.L., Luo, G., et al., (2019). Transcriptome-wide Mapping of Internal N (7)-Methylguanosine Methylome in Mammalian mRNA. *Mol. Cell.* **74** 1304–16 e8.
14. Malbec, L., Zhang, T., Chen, Y.S., Zhang, Y., Sun, B.F., Shi, B.Y., et al., (2019). Dynamic methylome of internal mRNA N(7)-methylguanosine and its regulatory role in translation. *Cell Res.* **29**, 927–941.
15. Chen, W., Feng, P., Song, X., Lv, H., Lin, H., (2019). iRNA-m7G: Identifying N(7)-methylguanosine Sites by Fusing Multiple Features. *Mol. Ther. Nucleic Acids* **18**, 269–274.
16. Song, B., Tang, Y., Chen, K., Wei, Z., Rong, R., Lu, Z., et al., (2020). m7GHub: deciphering the location, regulation and pathogenesis of internal mRNA N7-methylguanosine (m7G) sites in human. *Bioinformatics* **36**, 3528–3536.
17. Yang, Y.H., Ma, C., Wang, J.S., Yang, H., Ding, H., Han, S. G., et al., (2020). Prediction of N7-methylguanosine sites in human RNA based on optimal sequence features. *Genomics* **112**, 4342–4347.
18. Liu, X., Liu, Z., Mao, X., Li, Q., (2020). m7GPredictor: An improved machine learning-based model for predicting internal m7G modifications using sequence properties. *Anal. Biochem.* **609**, 113905.
19. Bi, Y., Xiang, D., Ge, Z., Li, F., Jia, C., Song, J., (2020). An Interpretable Prediction Model for Identifying N(7)-Methylguanosine Sites Based on XGBoost and SHAP. *Mol. Ther. Nucleic Acids.* **22**, 362–372.
20. Dai, C., Feng, P., Cui, L., Su, R., Chen, W., Wei, L., (2021). Iterative feature representation algorithm to improve the predictive performance of N7-methylguanosine sites. *Brief Bioinform.* **22**
21. Ning, Q., Sheng, M., (2021). m7G-DLSTM: Intergrating directional Double-LSTM and fully connected network for RNA N7-methlguanosine sites prediction in human. *Chemomet. Intell. Lab. Syst.* **217**, 104398.
22. Zhang, L., Qin, X., Liu, M., Liu, G., Ren, Y., (2021). BERT-m7G: A Transformer Architecture Based on BERT and Stacking Ensemble to Identify RNA N7-Methylguanosine Sites from Sequence Information. *Comput. Math. Methods Med.* **2021**, 7764764.
23. Basith, S., Lee, G., Manavalan, B., (2021). STALLION: a stacking-based ensemble learning framework for prokaryotic lysine acetylation site prediction. *Brief Bioinform.*.
24. Li, F., Guo, X., Jin, P., Chen, J., Xiang, D., Song, J., et al., (2021). Porpoise: a new approach for accurate prediction of RNA pseudouridine sites. *Brief Bioinform.*.
25. Manavalan, B., Basith, S., Shin, T.H., Lee, G., (2021). Computational prediction of species-specific yeast DNA replication origin via iterative feature representation. *Brief Bioinform.* **22**
26. Wei, L., Su, R., Luan, S., Liao, Z., Manavalan, B., Zou, Q., et al., (2019). Iterative feature representations improve N4-methylcytosine site prediction. *Bioinformatics* **35**, 4930–4937.
27. Tang, Y., Chen, K., Song, B., Ma, J., Wu, X., Xu, Q., et al., (2021). m6A-Atlas: a comprehensive knowledgebase for unraveling the N6-methyladenosine (m6A) epitranscriptome. *Nucleic Acids Res.* **49**, D134–D143.
28. Li, F., Guo, X., Xiang, D., Pitt, M.E., Bainomugisa, A., Coin, L.J.M., (2022). Computational analysis and prediction of PE_PGRS proteins using machine learning. *Comput. Struct. Biotechnol. J.* **20**, 662–674.
29. Mastery ML. A gentle introduction to k-fold cross-validation. Retrieved from machinelearningmastery com/k-fold-cross-validation. 2019.
30. Basith, S., Manavalan, B., Shin, T.H., Lee, G., (2019). SDM6A: A Web-Based Integrative Machine-Learning Framework for Predicting 6mA Sites in the Rice Genome. *Mol. Ther. Nucleic Acids* **18**, 131–141.
31. Zhang, Y., Yu, S., Xie, R., Li, J., Leier, A., Marquez-Lago, T.T., et al., (2020). PeNGaRoo, a combined gradient boosting and ensemble learning framework for predicting non-classical secreted proteins. *Bioinformatics* **36**, 704–712.
32. Li, F., Chen, J., Ge, Z., Wen, Y., Yue, Y., Hayashida, M., et al., (2021). Computational prediction and interpretation of both general and specific types of promoters in Escherichia coli by exploiting a stacked ensemble-learning framework. *Brief Bioinform.* **22**, 2126–2140.
33. Xie, R., Li, J., Wang, J., Dai, W., Leier, A., Marquez-Lago, T.T., et al., (2021). DeepVF: a deep learning-based hybrid framework for identifying virulence factors using the stacking strategy. *Brief Bioinform.* **22**
34. Manavalan, B., Basith, S., Shin, T.H., Wei, L., Lee, G., (2019). Meta-4mCpred: A Sequence-Based Meta-Predictor for Accurate DNA 4mC Site Prediction Using Effective Feature Representation. *Mol. Ther. Nucleic Acids* **16**, 733–744.
35. Hasan, M.M., Schaduangrat, N., Basith, S., Lee, G., Shoombuatong, W., Manavalan, B., (2020). HLPpred-Fuse: improved and robust prediction of hemolytic peptide and its activity by fusing multiple feature representation. *Bioinformatics* **36**, 3350–3356.
36. Liang, X., Li, F., Chen, J., Li, J., Wu, H., Li, S., et al., (2021). Large-scale comparative review and assessment of computational methods for anti-cancer peptide identification. *Brief Bioinform.* **22**
37. Wei, L., He, W., Malik, A., Su, R., Cui, L., Manavalan, B., (2021). Computational prediction and interpretation of cell-specific replication origin sites from multiple eukaryotes by exploiting stacking framework. *Brief Bioinform.* **22**
38. Basith, S., Hasan, M.M., Lee, G., Wei, L., Manavalan, B., (2021). Integrative machine learning framework for the identification of cell-specific enhancers from the human genome. *Brief Bioinform.*.
39. Wei, L., Ye, X., Xue, Y., Sakurai, T., Wei, L., (2021). ATSE: a peptide toxicity predictor by exploiting structural and evolutionary information based on graph neural network and attention mechanism. *Brief Bioinform.* **22**
40. Zhang, Z.Y., Yang, Y.H., Ding, H., Wang, D., Chen, W., Lin, H., (2021). Design powerful predictor for mRNA subcellular location prediction in Homo sapiens. *Brief Bioinform.* **22**, 526–535.
41. Wei, L., Ye, X., Sakurai, T., Mu, Z., Wei, L., (2022). ToxIBTL: prediction of peptide toxicity based on information bottleneck and transfer learning. *Bioinformatics*.

42. Yang, X., Ye, X., Li, X., Wei, L., (2021). iDNA-MT: Identification DNA Modification Sites in Multiple Species by Using Multi-Task Learning Based a Neural Network Tool. *Front. Genet.* **12**, 411.
43. Hasan, M.M., Shoombuatong, W., Kurata, H., Manavalan, B., (2021). Critical evaluation of web-based DNA N6-methyladenine site prediction tools. *Brief Funct. Genomics* **20**, 258–272.
44. Manavalan, B., Hasan, M.M., Basith, S., Gosu, V., Shin, T.-H., Lee, G., (2020). Empirical Comparison and Analysis of Web-Based DNA N4-Methylcytosine Site Prediction Tools. *Mol. Therapy-Nucleic Acids* **22**, 406–420.
45. Sethi, A., Gu, M., Gumusgoz, E., Chan, L., Yan, K.K., Rozowsky, J., et al., (2020). Supervised enhancer prediction with epigenetic pattern recognition and targeted validation. *Nature Methods* **17**, 807–814.
46. Basith, S., Lee, G., Manavalan, B., (2022). STALLION: a stacking-based ensemble learning framework for prokaryotic lysine acetylation site prediction. *Brief Bioinform.* **23**
47. Lv, H., Zhang, Y., Wang, J.S., Yuan, S.S., Sun, Z.J., Dao, F.Y., et al., (2022). iRice-MS: An integrated XGBoost model for detecting multitype post-translational modification sites in rice. *Brief Bioinform.* **23**
48. Malik, A., Subramaniyam, S., Kim, C.B., Manavalan, B., (2022). SortPred: The first machine learning based predictor to identify bacterial sortases and their classes using sequence-derived information. *Comput. Struct. Biotechnol. J.* **20**, 165–174.
