# Supplementary material for: PDBx/mmCIF Ecosystem: Foundational Semantic Tools for Structural Biology
Source: J Mol Biol. Author manuscript; Available in PMC 2023 Jun 26. (PMC10292674; doi:10.1016/j.jmb.2022.167599)
Supplement: Article [file NIHMS1907597-supplement-Article.zip › HTSDSF-Explorer--A-Novel-Tool-to-Analyze-High-throu_2022_Journal-of-Molecula.pdf]

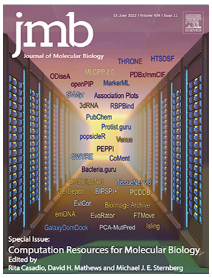

HTSDSF Explorer, A Novel Tool to Analyze High-throughput DSF Screenings

Pau Martin-Malpartida [1*], Emil Hausvik [2], Jarl Underhaug [3], Carles Torner [1], Aurora Martinez [2] and Maria J. Macias [1,4*]

1 - ***Institute for Research in Biomedicine,*** *The Barcelona Institute of Science and Technology, Baldiri Reixac, 10, Barcelona 08028, Spain*

2 - ***Department of Biomedicine,*** *University of Bergen, Jonas Lies vei 91, 5009 Bergen, Norway*

3 - ***Department of Chemistry,*** *University of Bergen, Allégaten 41, 5007 Bergen, Norway*

4 - ***Institució Catalana de Recerca i Estudis Avançats (ICREA),*** *Passeig Lluís Companys 23, Barcelona 08010, Spain*

Correspondence to Pau Martin-MalpartidaMaria J. Macias: Institute for Research in Biomedicine, The Barcelona Institute of Science and Technology, Baldiri Reixac, 10, Barcelona 08028, Spain. pau.martin@irbbarcelona.org *(P. Martin-Malpartida),* maria.macias@irbbarcelona.org *(M.J. Macias),* @medofak_uib, @biorecognition *(A. Martinez),* @maciaslab1 *(M.J. Macias)*
https://doi.org/10.1016/j.jmb.2021.167372
Edited by Rita Casadio

Abstract

The identification of new drugs for novel therapeutic targets requires the screening of libraries containing tens of thousands of compounds. While experimental screenings are assisted by high-throughput technologies, in target-based biophysical assays, such as differential scanning fluorimetry (DSF), the analysis steps must be calculated manually, often combining several software packages. To simplify the determination of the melting temperature (T_m) of the target and the change induced by ligand binding (ΔT_m), we developed the HTSDSF explorer, a versatile, all-in-one, user-friendly application suite. Implemented as a server-client application, in the primary screenings, HTSDSF explorer pre-analyzes and displays the T_m and ΔT_m results interactively, thereby allowing the user to study hundreds of conditions and select the primary hits in minutes. This application also allows the determination of preliminary binding constants (K_D) through a series of subsequent dose–response assays on the primary hits, thereby facilitating the ranking of validated hits and the advance of drug discovery efforts.

© 2021 Elsevier Ltd. All rights reserved.

Introduction

In recent years, the discovery of novel molecules with pharmacological applications has been accelerated thanks to the use of high-throughput screening (HTS) assays that can scan libraries with thousands of molecules each day.[1]

Some of these libraries are organized and distributed to users through actions such as the EU-OPENSCREEN.[2] Supported by HTS platforms throughout Europe, this initiative provides access to a rationally selected compound collection of up to 140,000 commercial and proprietary compounds. Among the strategies used to identify binders for therapeutic targets, differential scanning fluorimetry (DSF) has gained recognition as an affordable and efficient HTS technique to discover innovative candidates in drug discovery projects.[3] The screening is based on the identification of low molecular weight ligands through changes in protein thermal stability upon binding. It is performed using a real-time polymerase chain reaction (RT-PCR) system and fluorescent dyes, such as SYPRO Orange in the case of soluble proteins. The dye binds to hydrophobic patches of the protein that become exposed upon thermal denaturation.[3–6] The same

0022-2836/© 2021 Elsevier Ltd. All rights reserved.

DSF technique and equipment can be used to acquire a series of dose–response assays (DRAs), thus allowing the determination of preliminary binding constants (K_D) with values often comparable to those obtained by isothermal titration calorimetry[7] and surface plasmon resonance (SPR).[8] These apparent K_D values help categorize the hits prior to performing other orthogonal validation strategies, thus contributing to the hit-to-lead optimization phase.

In most laboratories, the first part of the screening process, which is related to compound and protein handling, is highly automated thanks to the use of 96- or 384-well plates and pipetting robots as well as well-established protocols for protein expression and purification in mg scale (Figure 1 (A)). These automated steps facilitate the reproducibility of the screening and replicates, and the comparison of results between laboratories and users. However, the capacity to screen large libraries of compounds quickly generates huge amounts of data, with the analysis step being one of the main bottlenecks of drug screening. To speed data analysis, several tools have been developed in the last decade. Most tools focus on studying protein stability under different buffer conditions or in low to medium range screening assays.[9–12] However, they do not incorporate information of the molecules nor do they combine the results in a single file displaying the final ranking of best compounds. In addition, hit validation is not included, and this process requires the use of additional general-purpose data analysis software like GraphPad Prism (www.graphpad.com) or OriginLab (originlab.com) to determine the apparent K_D values. To simplify the analysis of large HTS datasets and DRAs in a systematic and user-friendly manner, we developed an open-source package named HTSDSF explorer (Figure 1(B)).

Results

Program description

The HTSDSF explorer is designed as a server-client application that runs locally. The server is coded in python3 and implemented as a custom webserver, which can be downloaded at https://github.com/maciaslab/htsdsf_explorer. The software is compatible with DSF data acquired using different qPCR systems (LightCycler, BioRad and QuantStudio formats), and either 96- or 384-well plates. DSF data are stored as data points containing the fluorescence value and the associated temperature. A ready-to-use version for MS Windows that does not require the installation of python3 is also available.

The client is a web app, coded in JavaScript, which is executed in the browser at the user end. This approach ensures high efficiency and compatibility, as most computing devices and operating systems have a python3 port available and a modern web browser. The server is responsible for loading and managing the DSF data acquired regardless of the qPCR system used, and for converting them into the internal data model that is sent to the client for displaying. The display is user-friendly and highly intuitive. Both the server and client communicate using standard HTTP requests, and data are interchanged using the JavaScript Object Notation (json) format. The server is also responsible for storing persistent data and generating the reports requested by the user, including hit ranking and K_D calculation for hit compounds. A description of the software is included in the accompanying video and in the documentation provided with the package.

Experimental design for HTS binding assays

As an example of a HTS experiment, the screening of 100,000 compounds generates about 300 × 384-well plates and 120,000 experiments to examine, including references. The analysis requires the definition of a threshold for the assay response, which might need to be modified, along with the number of conditions analyzed and the observed melting temperature (T_m) of the target protein without the compound (reference wells or DMSO controls) and with compound, as well as the ΔT_m (T_m with compound – averaged T_m in reference wells. For instance, after analyzing 25,000 compounds and the hits observed, the user might need to increase or decrease the threshold and re-score all the compounds. Also, when manually analyzed, the user has to go through each of the 120,000 experiments, define proper and unique signals, thereby excluding experimental artifacts, and finally, select the list of preliminary hits. In our case, after studying approximately 60,000 compounds provided by EU-OPENSCREEN, we obtained a list of more than 500 promising hits that perturb the target T_m by ±1 °C in the HTS assay. The cutoff value depends on the SD determined in the DMSO control (usually, ±1 °C $\geq$ 3-fold SD). The molecules that destabilize or stabilize the target (the latter also known as pharmacological chaperones in our case study) then need further verification at lower concentrations. This process is typically performed as a dilution series, with the aim to obtain an indicator to rank the hits on the basis of affinity. The results are collected in the form of a K_D score.[13] In our case, this represented 30 additional 384-well plates and 11,000 conditions and the determination of the corresponding 500 K_Ds.

Data analysis

T_m screening

The program starts by displaying a list of files associated with the experimental plates. These files contain the raw experimental data (melting

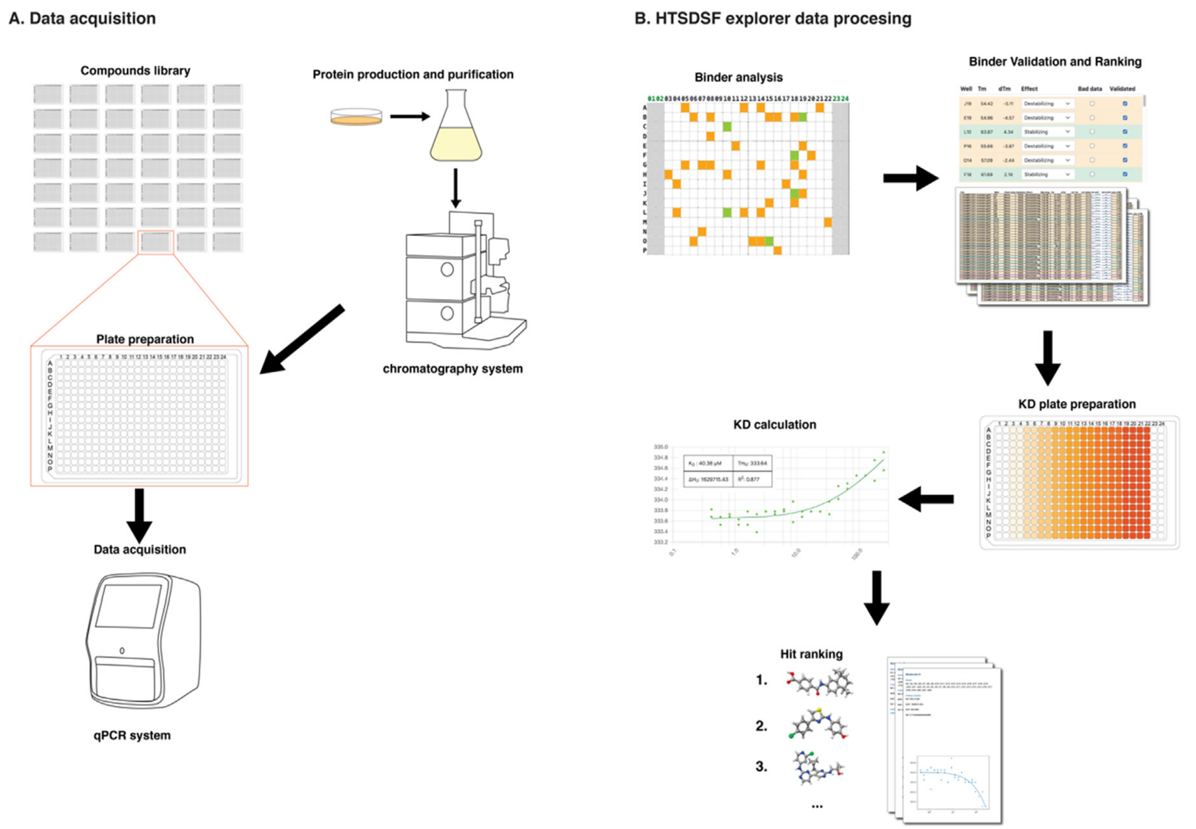

Figure 1. Experimental workflow. (A) Schematic representation of HTS assays. (B) Interface and outputs generated by HTSDSF explorer.

curves of Fluorescence (F) vs. temperature (T) for each well). If the plates belong to a defined library, each file can be correlated to a “plate name” containing information about the compounds dispensed per well. Once a plate is selected from the list (Figure 2(A)), the browser starts showing the results as a table, including the T_m and the ΔT_m (dT_m in Figure 2(B)) for each well, and as an interactive representation of the plate. Both representations simultaneously allow the selection of a given condition. In both, the conditions with an effect on the T_m are highlighted in green (ΔT_m higher than the threshold) or orange (lower) (Figure 2(B, C)). By default, the threshold is set at ±1 °C with respect to the reference target, but the cutoff value can be modified by the user to better fit the temperature changes observed for each specific protein target. Typically, we selected the cutoff at ΔT_m = 5-fold the SD of the T_m of the reference wells. The software supports any number of arbitrarily defined reference wells. In the example, 64 wells were used (shown as two gray columns in Figure 2(B)). When a well or row is selected, the corresponding melting curve and the first derivative are displayed as an additional panel for visual inspection (Figure 2(D)). The determination of the T_m is robust, and works even at signal-to-noise ratios as low as 3:1 (Figure 2 (E)). The user can either validate the T_m-value or flag it as poor or uncertain data. Once this has been done, the next well is automatically loaded and the user repeats the validation procedure until all preselected wells have been evaluated. The validation takes less than five seconds and requires only one mouse-click per well. After validation, the program generates an Excel report ranking all molecules from all selected plates on the basis of T_m changes. The Excel report contains values and the curves for all wells, allowing the user to visualize the curves in the file without the need to go back and forth to the program. For the best conditions, the user can prepare high quality plots from the DSF data.

Hit validation and K_D determination

Once some compounds have been identified as potential hits, it is advisable to prepare DRA plates to estimate apparent K_D values, since the primary screenings are normally performed at high compound excess (e.g., 250 μM). DRA plates are normally designed with the concentrations of the compounds varying along a given row and with an arbitrary number of experimental replicates (Figure 3). The software includes a dose–response plate editor.

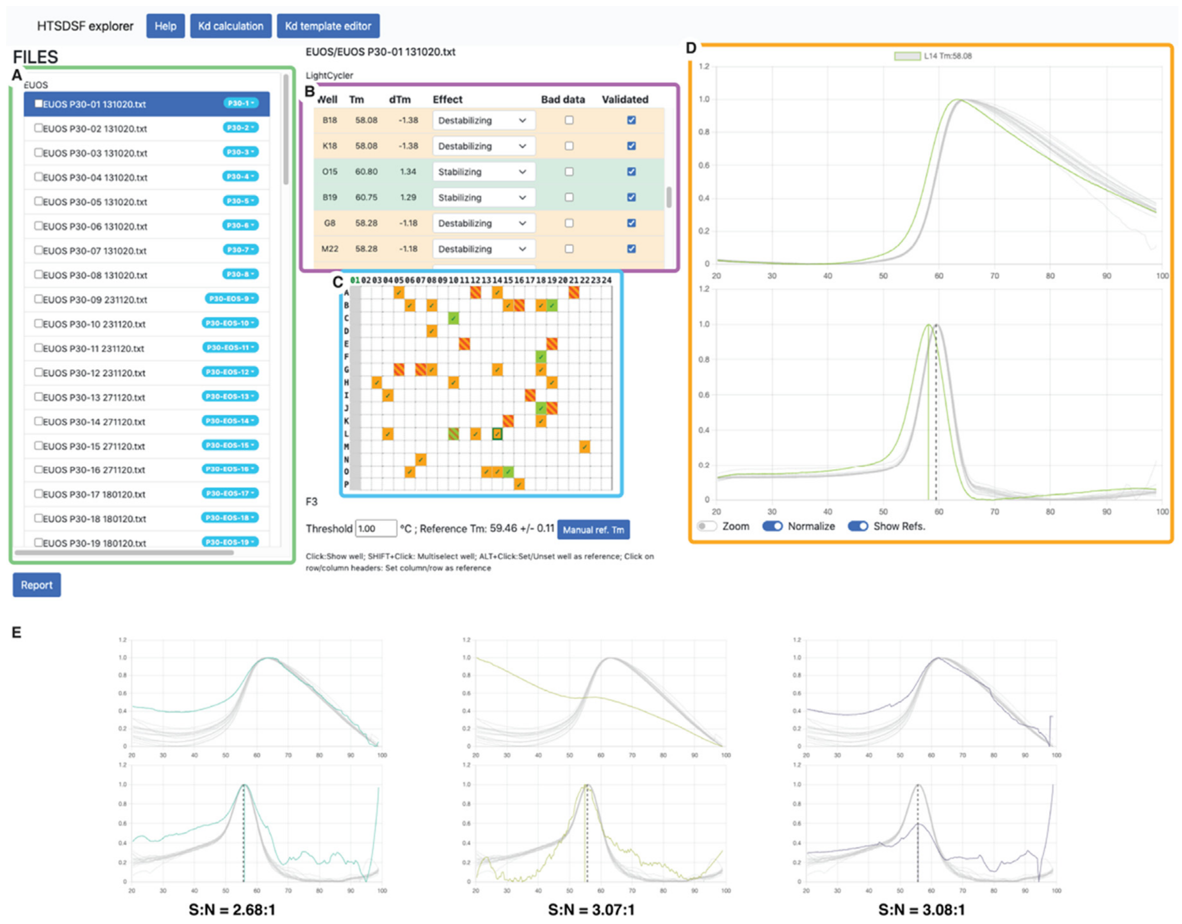

Figure 2. Plate and well browser, with the different components highlighted. (A) File browser, (B) Well table, (C) Plate representation, (D) Fluorescence vs. Temperature with calculation of T_m-values. A video showing these features is included. (E) Robust T_m estimation in unfavorable experimental cases with poor signal-to-noise ratios (S:N), S:N is calculated as the mean of the data divided by the standard deviation (SD).

The DR plate-designs stored in the editor can be loaded into the K_D module for K_D calculation. The program will fit the T_m-values for each concentration to the equation described in the methods section.[6,14] In the K_D module, the user can easily disable outliers by clicking on the graph points, and the K_D and ΔH_0 estimations are automatically recalculated after each modification.

Conclusion

HTSDSF explorer is an all-in-one open-source application suite able to analyze HTS DSF data in a highly intuitive and rapid manner. This software reads input files acquired in the most common qPCR systems, and the data are visualized through a user-friendly interface that allows the user to customize conditions for the analysis and validate the results. HTSDSF explorer has a web interface, but it is run locally, ensuring its reliability and quick access to large amounts of data. The output is a report containing the main features of the experiments (Excel tables and graphs) and it can include either single plate or multiple plate analyses. The same software is also able to design and analyze dose–response assays rapidly and easily and determine apparent K_D constants to consistently categorize hits, allowing to start defining potential pharmacophores. The comparison of chemical properties of hits with other tested molecules belonging to the library is also advantageous in the preparation of the pharmacophores and clustering of compounds. This comparison aids to reduce the number of compounds to be validated and optimized in other expensive and time-consuming orthogonal *in vitro* assays.

Methods

Expression and purification of the protein

Human SMAD4 MH2 domain (272–552) was cloned using an ‘In Fusion Cloning strategy’. The

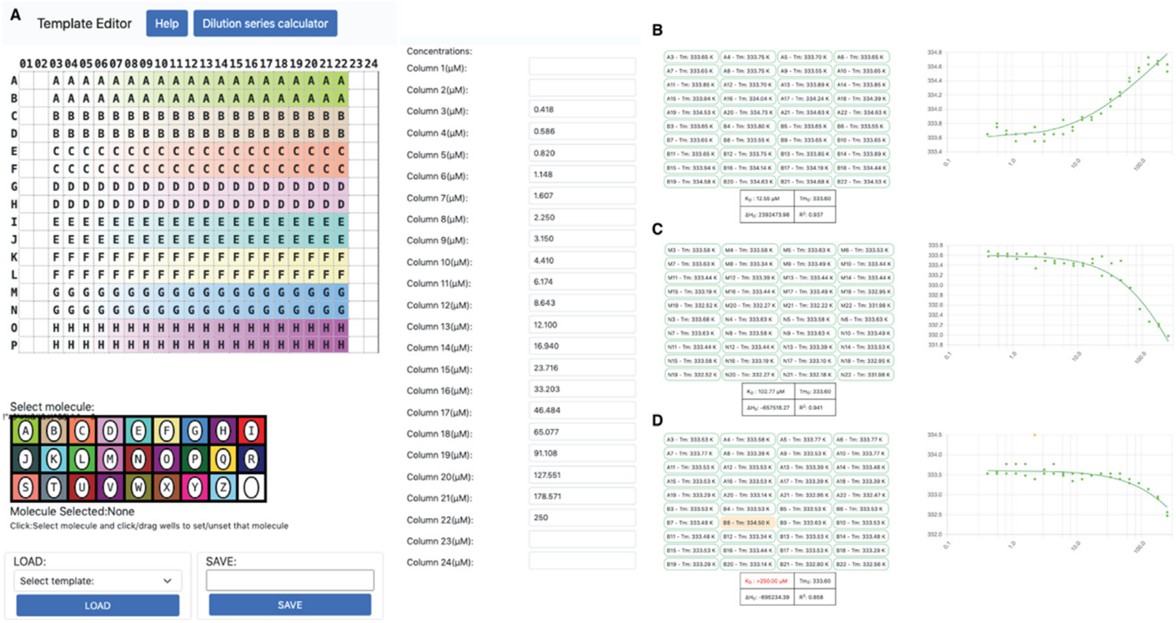

Figure 3. (A) Experimental design for an 8-molecule dose–response assay (DRA) with duplicates. (B) Outputs of the K_D module. Stabilizer hit compound. (C) Destabilizer hit compound. (D) Low affinity destabilizer compound, where the maximum concentration is far from saturation.

insert was synthesized by Thermo Fisher Scientific. Codons were optimized for expression in *E. coli* using LB medium at 37 °C. Protein expression was induced with IPTG (0.5 mM) and after induction, the bacteria cultures were incubated O/N at 20 °C. Cultures were centrifuged at 3500*g* for 15 min at 4 °C and the pellet was resuspended in a "lysis buffer" containing 50 mM Tris pH8.0, 400 mM NaCl, 400 mM Imidazole, 0.1 % Tween, and 1 mM TCEP. Protein was purified following standard procedures essentially as described.[15,16] The MH2 domain was further purified by size exclusion chromatography using a preparative grade HiLoad™ 16/60 Superdex75 from GE Healthcare and then concentrated at 7–10 mg/mL, in 20 mM pH 7.5, 100 mM NaCl, 2 mM TECP buffer. We purified ~150 mg of protein for the screening. Protein preparations were verified by Mass Spectrometry and characterized by NMR and SAXS (BMRB: 50737; SASBDB: SASDKG9).[16]

High-throughput screening

The initial HTS step by DSF was performed essentially as described in[6]. Briefly, the experiments were performed with the purified SMAD4 MH2 domain with and without compounds, in a LightCycler 480 Real-Time PCR System (Roche Applied Science), using a total volume of 10 µL in 384-well microplates (Roche Applied Science). Protein was diluted to 50 µM in 20 mM Tris pH 7.5, 100 mM NaCl, and 2 mM TCEP, with 5X SYPRO Orange. Binding results were exported to txt format for analysis with HTSDSF Explorer. Compounds were dissolved in DMSO and then added to the protein and SYPRO Orange solution to a final concentration of 80 µg/mL (corresponding to an averaged compound concentration of 200 µM) and 4% DMSO. Samples were incubated at room temperature for at least 10 min before loading into the PCR-instrument. Controls with 4% DMSO were performed on each plate. Unfolding curves were registered from 20 °C to 95 °C at a scan rate of 2 °C/min.

Accepted data formats

The software has a user-definable data directory to collect the DSF files. HTSDSF Explorer accepts data exported from Bio-Rad (.xlsx), Roche LightCycler (.txt), Applied Biosystems QuantStudio and StepOnePlus (.txt).

In addition, a generic dsf file format (.gdsf) has been defined to allow the use of data acquired in different instruments. This format is a simple text file, with three columns separated by spaces containing, in order, the well, temperature and fluorescence. Details about the file formats can be found at the project web-page.

As the software is open-source, additional formats can be implemented by the user or by us upon request.

Data processing

Melting curves are used to obtain the T_m by calculating the gradient using the *numpy* gradient function and smoothed using a Savitzky-Golay

filter.[17] This curve is used to find local maxima (peak picking), which correspond to the protein T_m. This procedure generates curves that are easier to understand than the row data, without altering the T_m.[11] Peak picking is performed using the *scipy* find_peaks function, with prominence = 30% of the vertical curve range. In cases where the compound induces multiple observable transitions, we select the temperature that is closest to the reference T_m value.

Data export and storage

All user-validated T_m data are locally stored in a human-readable format that can be exported to Excel. Reports can either be generated for each plate or for various plates combined as a final report file.

The software allows the user to correlate each well in each plate with a ligand using plate-template information. For this feature, the user needs to fill in a text file with information about the plate ID, the well, the molecule ID and a smiles/InChI string. This information, if available, is added to the report.

K_D calculation

K_D calculation is performed by fitting the data points (ligand concentration and the corresponding T_m) to the equation described in[6].

$$T_{m,l} = \frac{\frac{\Delta H_0}{n} T_{m,0}}{\frac{\Delta H_0}{n} - R T_{m,0} \ln\left(\frac{K_d + [L]}{K_d}\right)}$$

where $T_{m,l}$ is the melting temperature at a concentration of a given ligand ([L]), K_D is the dissociation constant, $T_{m,0}$ is the melting temperature in the absence of ligand, ΔH_0 is the enthalpy of the unfolding of the protein at $T_{m,0}$, n is the number of binding sites, and R is the gas constant. $T_{m,0}$, ΔH_0 and K_D are obtained after the curve fitting and n is assumed to be 1. Starting values for the fitting are obtained by differential evolution[18] as implemented in *scipy*, and then using these values as initial conditions for a least-squares fitting.[19]

Video link:

http://maciasnmr.net/HTSDSF/HTSDSFvideo.mp4

CRediT authorship contribution statement

Pau Martin-Malpartida: Conceptualization, Software, Formal analysis, Writing – original draft, Writing – review & editing. **Emil Hausvik:** Investigation, Formal analysis. **Jarl Underhaug:** Software. **Carles Torner:** Investigation, Formal analysis. **Aurora Martinez:** Conceptualization, Supervision, Writing – review & editing, Funding acquisition. **Maria J. Macias:** Conceptualization, Supervision, Writing – original draft, Writing – review & editing, Funding acquisition.

Acknowledgements

We acknowledge the support received from the EU-OPENSCREEN team, the BiSS core facility (Department of Biomedicine, University of Bergen (UiB)) and the UiB-node of NOR-OPENSCREEN. We also thank Dr. N. Berrow (IRB Barcelona, Protein Expression Unit) for providing reagents and helping with protein purification.

Funding

The HTS work was supported by EU-OPENSCREEN INSTRUCT-ERIC through funding from the European Union's Horizon 2020 Research and Innovation programme, grant agreement No 823893 (EU-OPENSCREEN-DRIVE). Access to the NMR facility (Grenoble-FR2) was granted by the INSTRUCT-Eric Integrating Biology program (grant 7192, title: Smad4 MH2 domain) and access to Bio-SAXS BM29 was part of the MX-1941 BAG proposal. The work was also financed through the Spanish Ministry of Economy, Industry and Competitiveness (MINECO) (BFU2017-82675-P, M.J.M), IRB Barcelona, AGAUR (SGR-50, M.J.M), the Research Council of Norway (NOR-OPENSCREEN 245922/F50 and FRIMEDBIO 261826, A.M.) and the Western Norway Regional Health Authorities (project 912246 to A.M.). C.T. is a recipient of a PhD fellowship from the Government of Catalonia (Generalitat de Catalunya) (FI) (2021, ref. BDNS 525925). We gratefully acknowledge institutional funding from the CERCA Programme of the Government of Catalonia and from MINECO through the Centres of Excellence Severo Ochoa Award. M.J.M is an ICREA Programme Investigator.

Declaration of interests

The authors declare that they have no known competing financial interests or personal relationships that could have appeared to influence the work reported in this paper.

Received 1 October 2021;
Accepted 15 November 2021;
Available online 19 November 2021

Keywords:
high-throughput compound screening;
thermal shift assay;
differential scanning fluorimetry;
automatic T_m;
automatic K_D analysis

References

1. Macarron, R., Banks, M.N., Bojanic, D., Burns, D.J., Cirovic, D.A., Garyantes, T., et al., (2011). Impact of high-throughput screening in biomedical research. *Nature Rev. Drug Discov.* **10**, 188–195.
2. Brennecke, P., Rasina, D., Aubi, O., Herzog, K., Landskron, J., Cautain, B., et al., (2019). EUOPENSCREEN: A novel collaborative approach to facilitate chemical biology. *SLAS Discov.* **24**, 398–413.
3. Niesen, F.H., Berglund, H., Vedadi, M., (2007). The use of differential scanning fluorimetry to detect ligand interactions that promote protein stability. *Nature Protoc.* **2**, 2212–2221.
4. Boyd, R.E., Lee, G., Rybczynski, P., Benjamin, E.R., Khanna, R., Wustman, B.A., et al., (2013). Pharmacological chaperones as therapeutics for lysosomal storage diseases. *J. Med. Chem.* **56**, 2705–2725.
5. Pantoliano, M.W., Petrella, E.C., Kwasnoski, J.D., Lobanov, V.S., Myslik, J., Graf, E., et al., (2001). Highdensity miniaturized thermal shift assays as a general strategy for drug discovery. *J. Biomol. Screen.* **6**, 429–440.
6. Støve, S.I., Flydal, M.I., Hausvik, E., Underhaug, J., Martinez, A., (2020). Differential scanning fluorimetry in the screening and validation of pharmacological chaperones for soluble and membrane proteins, Chapter 15. Academic Press, Walthum.
7. Gao, K., Oerlemans, R., Groves, M.R., (2020). Theory and aapplications of differential scanning fluorimetry in earlystage drug discovery. *Biophys. Rev.* **12**, 85–104.
8. Martin, I., Underhaug, J., Celaya, G., Moro, F., Teigen, K., Martinez, A., Muga, A., (2013). Screening and evaluation of small organic molecules as ClpB inhibitors and potential antimicrobials. *J. Med. Chem.* **56** (18), 7177–7189.
9. Lee, P.H., Huang, X.X., Teh, B.T., Ng, L.M., (2019). TSACRAFT: A Free Software for Automatic and Robust Thermal Shift Assay Data Analysis. *SLAS Discov.* **24**, 606–612.
10. Rosa, N., Ristic, M., Seabrook, S.A., Lovell, D., Lucent, D., Newman, J., (2015). Meltdown: A tool to help in the interpretation of thermal melt curves acquired by differential scanning fluorimetry. *J. Biomol. Screen.* **20**, 898–905.
11. Sun, C., Li, Y., Yates, E.A., Fernig, D.G., (2020). SimpleDSFviewer: A tool to analyze and view differential scanning fluorimetry data for characterizing protein thermal stability and interactions. *Protein Sci.* **29**, 19–27.
12. Wang, C.K., Weeratunga, S.K., Pacheco, C.M., Hofmann, A., (2012). DMAN: A Java tool for analysis of multiwell differential scanning fluorimetry experiments. *Bioinformatics* **28**, 439–440.
13. Bai, N., Roder, H., Dickson, A., Karanicolas, J., (2019). Isothermal analysis of thermofluor data can readily provide quantitative binding affinities. *Sci. Rep.* **9**, 2650.
14. Owen, T., (1994). The origin of inner planet atmospheres. *Philos. Trans. Phys. Sci. Eng.* **349**, 209–211. discussion 12.
15. Aragon, E., Wang, Q., Zou, Y., Morgani, S.M., Ruiz, L., Kaczmarska, Z., et al., (2019). Structural basis for distinct roles of SMAD2 and SMAD3 in FOXH1 pioneerdirected TGFbeta signaling. *Genes Dev.* **33**, 1506–1524.
16. Gomes, T., Martin, P., Malpartida, Ruiz, L., Aragón, E., Cordeiro, T.N., Macias, M.J., (2021). Conformational landscape of multidomain SMAD proteins. *Comput. Struct. Biotechnol. J.* **14** (19), 5210–5224.
17. Savitzky, A., Golay, M.J.E., (1964). Smoothing and differentiation of data by simplified least squares procedures. *Anal. Chem.* **36**, 1627–1639.
18. Storn, R., Price, K., (1997). Differential evolution – A simple and efficient heuristic for global optimization over continuous spaces. *J. Global Optim.* **11**, 341–359.
19. Branch, M.A., Coleman, T.F., Li, Y., (1999). A subspace, interior, and conjugate gradient method for large-scale bound-constrained minimization problems. *SIAM J. Sci. Comput.* **21**, 1–23.
