# Supplementary material for: PDBx/mmCIF Ecosystem: Foundational Semantic Tools for Structural Biology
Source: J Mol Biol. Author manuscript; Available in PMC 2023 Jun 26. (PMC10292674; doi:10.1016/j.jmb.2022.167599)
Supplement: Article [file NIHMS1907597-supplement-Article.zip › The-Organ-Disease-Annotations--ODiseA--Database-of-Her_2022_Journal-of-Molec.pdf]

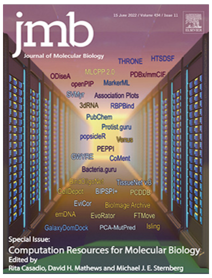

The Organ-Disease Annotations (ODiseA) Database of Hereditary Diseases and Inflicted Tissues

Idan Hekselman [1], Lior Kerber [1†], Maya Ziv [1†], Gil Gruber [1] and Esti Yeger-Lotem [1,2*]

1 - ***Department of Clinical Biochemistry and Pharmacology,*** *Ben-Gurion University of the Negev, Be'er Sheva, Israel*
2 - ***The National Institute for Biotechnology in the Negev,*** *Ben-Gurion University of the Negev, Be'er Sheva, Israel*

Correspondence to Esti Yeger-Lotem: Department of Clinical Biochemistry and Pharmacology, Ben-Gurion University of the Negev, Be'er Sheva, Israel. *estiyl@bgu.ac.il (E. Yeger-Lotem), @IdanXL Esti (I. Hekselman), @YegerLotemLab (E. Yeger-Lotem).*
https://doi.org/10.1016/j.jmb.2022.167619
Edited by Michael Sternberg

Abstract

Hereditary diseases tend to manifest clinically in few selected tissues. Knowledge of those tissues is important for better understanding of disease mechanisms, which often remain elusive. However, information on the tissues inflicted by each disease is not easily obtainable. Well-established resources, such as the Online Mendelian Inheritance in Man (OMIM) database and Human Phenotype Ontology (HPO), report on a spectrum of disease manifestations, yet do not highlight the main inflicted tissues. The Organ-Disease Annotations (ODiseA) database contains 4,357 thoroughly-curated annotations for 2,181 hereditary diseases and 45 inflicted tissues. Additionally, ODiseA reports 692 annotations of 635 diseases and the pathogenic tissues where they emerge. ODiseA can be queried by disease, disease gene, or inflicted tissue. Owing to its expansive, high-quality annotations, ODiseA serves as a valuable and unique tool for biomedical and computational researchers studying genotype-phenotype relationships of hereditary diseases. ODiseA is available at https://netbio.bgu.ac.il/odisea.

© **2022 Elsevier Ltd. All rights reserved.**

Introduction

Hereditary diseases affect 6% of the population.[1] Since many hereditary diseases still lack cure and treatment options remain limited, efforts to advance treatment have focused on better understanding the disease mechanisms. A major part of understanding disease mechanisms is to consider the tissues and organs (briefly 'tissues') that show pathophysiological changes in patients.[2] These changes are identified via clinical manifestations, or through additional laboratory or imaging tests. Knowledge of disease-inflicted tissues can help genetic diagnosis and molecular understanding of disease. For example, patients with rare muscle disorders were genetically diagnosed by comparing their genetic sequence to the sequence of transcripts expressed in muscle tissues.[3]

Tissues inflicted by each disease may be inferred from several curated databases. The Online Mendelian Inheritance in Man (OMIM) database organized the clinical synopsis of hereditary diseases into anatomical systems,[4] for example, documenting Parkinson's disease as neurologic. Orphanet classified rare diseases by their major affected organ systems, e.g., hepatic, immune and renal disease.[5] The Human Phenotype Ontology (HPO) associated hereditary diseases with their phenotypic abnormalities in major organ systems, such as cardiovascular, respiratory and musculature systems.[6] Disease Ontology grouped disease-related biomedical terms into anatomical systems, such as gastrointestinal and nervous systems.[7] However, inferring the inflicted tissues from these databases is not always straightforward. First, their resolution is of systems rather than

0022-2836/© 2022 Elsevier Ltd. All rights reserved.

specific tissues. For instance, the disease ‘familial primary hyperparathyroidism’ (OMIM: 145000) was associated by OMIM and HPO with the endocrine system, although the parathyroid glands are the only tissues inflicted by this disease. Second, these databases aim to be comprehensive, and therefore report on anecdotal tissue abnormalities, like tissues with negligible changes or symptoms that occur in a small subset of the patients. Lastly, not all abnormalities imply on the tissues where the pathology emerges, denoted pathogenic tissues. For example, the disease ‘familial erythrocytosis type 1′ (OMIM: 133100), which is pathogenic in blood and blood-forming tissues, was associated with six additional non-pathogenic tissues.

Recently, computational efforts leveraged knowledge of disease-inflicted tissues to illuminate disease mechanisms and unravel mechanistic trends in hereditary diseases.[2] For instance, it was shown that normal tissues that express pathology tend to overexpress disease genes,[8] to downregulate compensatory genes,[9,10] and to be enriched for tissue-specific regulatory interactions.[11–13] However, the many efforts analyzed subsets of tissue-specific traits and diseases, which were never combined into a single repository. Future efforts could benefit from an expansive curated database that is dedicated to the annotation of hereditary diseases to their inflicted tissues.

We present the Organ-Disease Annotations (ODiseA) database that is devoted to providing high-quality, thoroughly-curated annotations of hereditary diseases and their inflicted tissues. These annotations relied on clinical features and empirical measurements that indicated pathophysiological changes in the inflicted tissues. We curated 2,181 hereditary diseases to 45 different tissues, resulting in 4,357 annotations. Additionally, we annotated the pathogenic tissues for 635 diseases, resulting in 692 annotations. ODiseA is available at https://netbio.bgu.ac.il/odisea. Users can easily query ODiseA by disease, tissue, or disease gene of interest.

Results

ODiseA reports on the tissues inflicted by genetically-solved hereditary diseases. To annotate disease-inflicted tissues, we conducted a thorough curation process (Figure 1(A); Methods). For each disease, a curator searched for candidate inflicted tissues by querying OMIM[4] and HPO,[6] and in case of limited information also by querying PubMed. A tissue was considered as candidate inflicted if clinical, laboratory, or imaging evidence that was relevant to that tissue was identified. Next, a candidate tissue was annotated as *inflicted* if all the following conditions were met: (1) The evidence pointed to a pathophysiological change in that tissue; (2) The pathophysiological change was not negligible compared to disease-related changes in other tissues; and (3) The pathophysiological change was not documented in a small subset of patients. If any of these conditions were not met, the candidate tissue was annotated as *non-inflicted*. Annotations of all candidate tissues were reassessed by a different curator based on the same criteria. Overall, ODiseA contained 4,357 annotations of 2,181 diseases and their 1,920 disease genes to 45 inflicted tissues (Figure 1(B)).

The expansive ODiseA dataset increased the number of diseases previously annotated to inflicted tissues by at least two-fold.[2,9,10,14–16] Therefore, we used these data to revisit previous findings. First, we counted the number of inflicted tissues per disease (Figure 1(C)). We found that 91% of the diseases inflicted upon at most three tissues. Second, we tested whether disease genes were overexpressed in the normal tissues that express pathology, as previously shown.[8,17] For this, we extracted gene expression profiles of disease genes from 52 tissues available through the Genotype-Tissue Expression (GTEx) consortium[13] (Methods), 39 of which were annotated as inflicted by diseases in ODiseA. Indeed, disease genes were highly expressed in inflicted versus unaffected tissues (Figure 1(D), $p < E\text{-}62$, Mann-Whitney U test). Hence, the data obtained from ODiseA recapitulates trends shown in hereditary diseases at a larger scale.

Notably, not all inflicted tissues are also pathogenic. For example, the disease ‘stress-induced myopathic carnitine palmitoyltransferase II deficiency’ (OMIM: 255110) is characterized by muscle breakdown, followed by renal failure due to renal myoglobin accumulation. Hence, whereas this disease inflicts on skeletal muscle and kidney, its pathophysiological change emerges in skeletal muscle, and subsequently inflicts on kidney. We conducted an additional curation process to identify pathogenic tissues, focusing on diseases that inflict on blood and bone marrow, kidney, lung, and trachea (Figure 2(A); Methods). Per disease, an inflicted tissue was annotated as *pathogenic* if the pathophysiological change originated in that tissue. Otherwise, the tissue was annotated as *non-pathogenic.* As before, all annotations were reassessed by a different curator based on the same criteria. Additionally, non-inflicted tissues were annotated as *non-pathogenic*. Overall, we curated 1,226 inflicted tissue annotations, resulting in 692 pathogenic annotations pertaining to 635 diseases (Figure 2 (B)). The different tissues showed varying proportions of pathogenic annotations (Figure 2 (C)). The pathogenicity of trachea was the most problematic to curate. In almost half of the kidney and lung cases, the inflicted tissue was also pathogenic, rising to 72% in blood and bone marrow. This difference corresponds to the sampling frequency of these tissues, as blood and

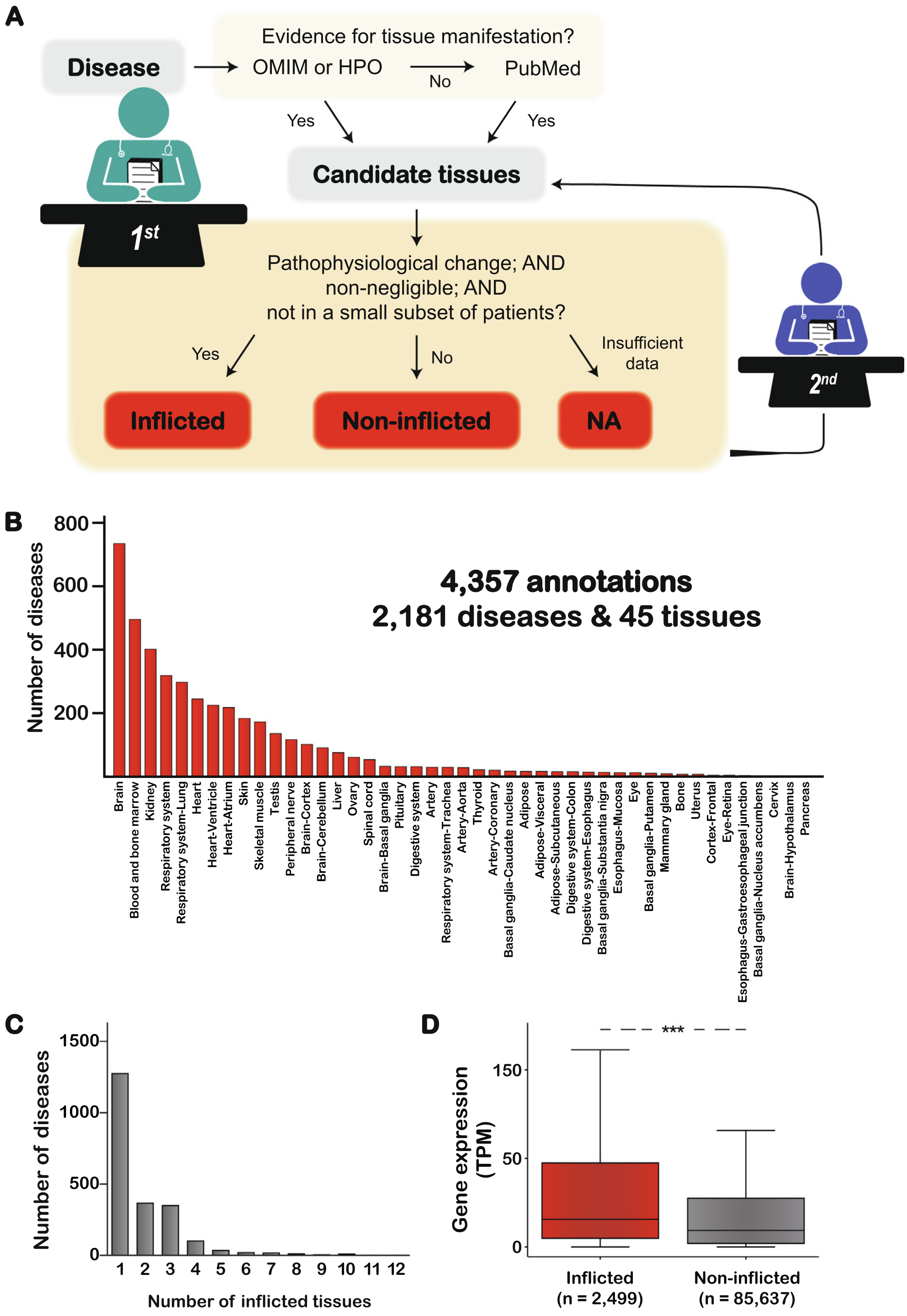

Figure 1. An overview of ODiseA database. (A) A schematic overview of the curation of hereditary diseases to inflicted tissues. The curation process was done by two curators. The first curator defined a set of candidate tissues, and then assessed whether they are inflicted by a disease. The second curator reassessed the annotations of these candidate tissues. (B) A bar plot representing the numbers of diseases (Y axis) annotated to each inflicted tissue (X axis). ODiseA includes 4,357 annotations of 2,181 diseases to 45 inflicted tissues. (C) A bar plot representing the numbers of diseases (Y axis) grouped by the number of tissues they inflict on (X axis). To prevent overrepresentation of diseases inflicting on ten brain-related tissues, they were collated as inflicting on a single brain tissue. (D) A box plot showing the expression level of disease genes (Y axis) in their inflicted versus unaffected tissues (X axis). Gene expression was extracted from Genotype-Tissue Expression (GTEx) version 8,[13] as transcripts per million (TPM; Methods). 'n' represents the number of pairs of disease genes and tissues. *** $p < E-62$, Mann-Whitney U test.

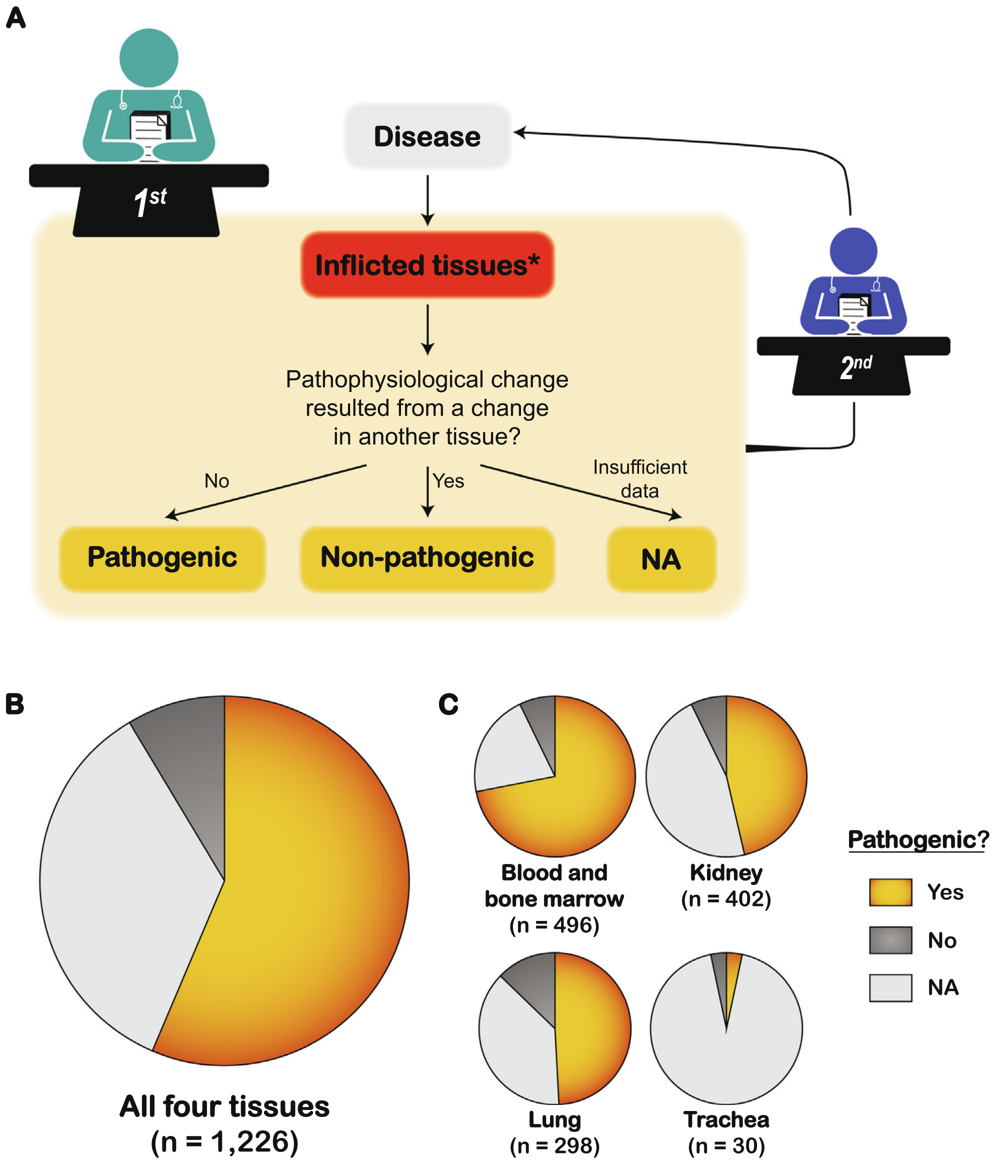

Figure 2. Annotations of diseases to pathogenic tissues. (A) A schematic overview of the curation of hereditary diseases to pathogenic tissues. The curation process was done by two curators that separately assessed whether the diseases inflicting on blood and bone marrow, kidney, lung, and trachea, emerge from those tissues. (B) A pie chart showing the proportion of inflicted-tissue annotations that were determined as pathogenic (orange), non-pathogenic (dark grey), or NA (light grey). 'n' represents the total number of annotations across the four tissues. (C) Same as B, per tissue. 'n' represents the total number of annotations in each tissue.

bone marrow are sampled routinely in clinical settings.

ODiseA can be queried by a tissue, a disease, or a disease gene. Anatomic relationships between tissues can be handily explored through a drop-down list. Diseases belonging to the same OMIM phenotypic series (i.e., diseases with similar clinical manifestation) can be queried together. The output page includes a table indicating the inflicted and pathogenic tissues per disease or disease gene, as well as the expression of the disease gene in the corresponding tissue, as reported by GTEx version 8. To easily sift through the output results, a bar plot to the right of the table indicates the number of diseases inflicting on each tissue. Lastly, all data can be downloaded from the main and output pages of ODiseA.

Discussion

ODiseA provides expansive high-quality annotations of 2,181 diseases to their inflicted tissues (Figure 1). Unlike current repositories that record the full spectrum of clinical manifestations in anatomical systems (e.g., OMIM, HPO, DO and Orphanet),[4–7] ODiseA reports annotations at the

resolution of a specific tissue, and highlights the mainly affected tissues. By that, ODiseA alleviates the need to infer tissue contexts for many hereditary diseases inflicting upon a variety of tissues. Additionally, ODiseA is unique in annotating pathogenic tissues for diseases, which is important for understanding the predisposition of normal tissues.[8,11,15] Despite the challenging curation of pathogenic tissues, ODiseA contains pathogenic tissue annotations for 635 diseases (Figure 2).

Knowledge of disease-inflicted tissues is beneficial for investigating diseases and their associated genes in relevant contexts. Disease genes were identified for over 6,000 hereditary diseases,[4] yet how their aberrations lead to disease phenotypes often remains unclear. For example, the molecular mechanisms by which individual aberrations in multiple genes lead to cardiomyopathy are yet to be resolved.[18] By acknowledging the tissue context of a disease, molecular characteristics of diseases can be revealed. Computational analyses revealed the tendency of disease genes for overexpression,[8] (Figure 1(D)), involvement in molecular interactions[12,19,20] and involvement in preferentially-active processes in inflicted tissues.[21] Additionally, similarity in inflicted tissues was shown to reflect similarity in disease genes.[22]

We found that most diseases inflicted on up to three tissues (91%; Figure 1(C)). However, certain tissues were more prone to disease than others. Whereas 10/45 (22%) tissues were inflicted by at most ten diseases, brain was inflicted by 735/2,181 (34%) diseases. Similarly, a recent large-scale study of 2,183 probands with rare diseases included 30% probands with intellectual disability, neurologic or neurodevelopmental disorders.[23] Hence, the varying numbers of annotations across tissues may be, at least in part, due to the varying tendency of tissues to be inflicted by hereditary diseases.

ODiseA can be extended in several ways. First, pathogenic tissue annotations can be extended to additional tissues. In addition to extensive manual curation, annotating pathogenic tissues of diseases could be accelerated by allowing users to submit corrections or suggest annotations. Alternatively, annotations could be accelerated by using prediction methods. For instance, tissues related to complex diseases were inferred by the tissue-specific expression of disease-associated genes.[24] In both cases, suggested annotations will be verified by ODiseA team. Second, ODiseA does not specifically deal with genetic heterogeneity, namely the differential infliction of tissues by different genes that are associated with the same Mendelian disease. Nevertheless, genetic heterogeneity within a specific phenotypic series can be explored through ODiseA. In total, ODiseA contains 260 phenotypic series that include two or more disease genes, six of which show genetic heterogeneity. For example, peroxisome biogenesis disorder (OMIM: PS214100) includes six disease genes that inflict on kidney (PEX1, PEX3, PEX5, PEX12, PEX13 and PEX19) and one disease gene that does not (PEX6). Lastly, the next frontier is to annotate the cell types inflicted by diseases.[25,26] This could be facilitated by single-cell technologies. For example, single-cell RNA sequencing of the airway epithelium in mouse and human revealed the pathogenic cell type associated with the pulmonary phenotype of cystic fibrosis.[27,28] We will continuously update ODiseA with high-quality annotations, to aid genetic and clinical efforts to discover mechanisms underlying hereditary diseases.

Methods

Hereditary diseases dataset. Hereditary diseases and their associated genes were extracted from OMIM.[4] Only diseases with dominant or recessive inheritance and a known molecular basis (phenotype mapping key 3) were analyzed.

Curation of inflicted tissues. Curation was performed by medical students in their clinical rotations, which were randomly assigned a subset of diseases. For each disease, curators searched for candidate inflicted tissues by querying OMIM[4] and HPO.[6] A tissue was considered as candidate inflicted if clinical, laboratory, or imaging evidence that was relevant to that tissue was identified. We also included candidate tissue annotations from previous studies.[2,9,10,14–17] In case no such tissues were found, curators also searched PubMed. Next, a candidate tissue was annotated as *inflicted* if: (1) The evidence pointed to a pathophysiological change in that tissue; (2) The pathophysiological change was not negligible compared to disease-related changes in other tissues; and (3) The pathophysiological change was not documented in a small subset of patients. A candidate tissue was annotated as *non-inflicted* if: (1) The evidence did not point to a pathophysiological change, or (2) the pathophysiological change was negligible compared to pathophysiological changes in other tissues, or (3) The pathophysiological change was evident in a small subset of the patients. A candidate tissue that did not fit the above criteria was annotated as *NA* under the inflicted category. Annotations of all candidate tissues were reassessed by a different curator based on the same criteria. Eventually, tissue names were consolidated and grouped together according to their anatomic relations.

Curation of pathogenic tissues. Curation of pathogenic tissues was performed for diseases inflicting on blood and bone marrow, kidney, lung, and trachea. Per disease, an inflicted tissue was annotated as *pathogenic* if the pathophysiological change originated in the same tissue. A tissue

was annotated as *non-pathogenic* if: (1) The change originated from another tissue; or (2) The tissue was annotated as *non-inflicted*. A tissue that did not fit the above criteria was annotated as *NA* under the pathogenic category. All annotations were reassessed by a different curator based on the same criteria.

Gene expression. Transcriptomic profiles of non-diseased human tissues measured via RNA-sequencing were downloaded from the GTEx portal version 8, as median transcripts per million (TPM[13]). 39/52 of these tissues matched annotated tissues in ODiseA (Table S1). In case multiple tissues in GTEx matched a single tissue in ODiseA (e.g., sun-exposed and non-exposed skin tissues in GTEx matched skin tissue in ODiseA), the expression value of each gene was set to the maximal median TPM in the relevant tissues in GTEx.

Implementation. ODiseA server was implemented in Python, using the Flask framework, with data stored on a MySQL database. The website client was programmed using the ReactJS framework and designed with Semantic-UI CSS. All bar plots were displayed by using React Google Charts. The website supports all major browsers. Recommended viewing resolution is 1440 $\times$ 900 and above.

CRediT authorship contribution statement

Idan Hekselman: Methodology, Formal analysis, Data curation, Writing – original draft, Writing – review & editing. **Lior Kerber:** Data curation. **Maya Ziv:** Data curation. **Gil Gruber:** Software. **Esti Yeger-Lotem:** Supervision, Conceptualization, Writing – original draft, Writing – review & editing.

DECLARATION OF COMPETING INTEREST

The authors declare that they have no known competing financial interests or personal relationships that could have appeared to influence the work reported in this paper.

Acknowledgements

This study was funded by the Israel Science Foundation [317/19 to E.Y.-L].

Appendix A. Supplementary Data

Supplementary data to this article can be found online at https://doi.org/10.1016/j.jmb.2022.167619.

Received 30 November 2021;
Accepted 26 April 2022;
Available online 30 April 2022

Keywords:
hereditary diseases;
tissue-specificity;
data integration;
pathogenicity;
clinical manifestation

† These authors contributed equally to this work.

References

1. Ferreira, C.R., (2019). The burden of rare diseases. *Am. J. Med. Genet. A* **179**, 885–892.
2. Hekselman, I., Yeger-Lotem, E., (2020). Mechanisms of tissue and cell-type specificity in heritable traits and diseases. *Nature Rev. Genet.* **21**, 137–150.
3. Cummings, B.B., Marshall, J.L., Tukiainen, T., Lek, M., Donkervoort, S., Foley, A.R., et al., (2017). Improving genetic diagnosis in Mendelian disease with transcriptome sequencing. *Sci. Transl. Med.* **9**
4. Amberger, J.S., Bocchini, C.A., Scott, A.F., Hamosh, A., (2019). OMIM.org: leveraging knowledge across phenotype-gene relationships. *Nucleic Acids Res.* **47**, D1038–D1043.
5. Orphanet: an online database of rare diseases and orphan drugs. https://www.orpha.net/.
6. Kohler, S., Gargano, M., Matentzoglu, N., Carmody, L.C., Lewis-Smith, D., Vasilevsky, N.A., et al., (2021). The Human Phenotype Ontology in 2021. *Nucleic Acids Res.* **49**, D1207–D1217.
7. Schriml, L.M., Munro, J.B., Schor, M., Olley, D., McCracken, C., Felix, V., et al., (2022). The Human Disease Ontology 2022 update. *Nucleic Acids Res.* **50**, D1255–D1261.
8. Lage, K., Hansen, N.T., Karlberg, E.O., Eklund, A.C., Roque, F.S., Donahoe, P.K., et al., (2008). A large-scale analysis of tissue-specific pathology and gene expression of human disease genes and complexes. *Proc. Natl. Acad. Sci. USA* **105**, 20870–20875.
9. Jubran, J., Hekselman, I., Novack, L., Yeger-Lotem, E., (2020). Dosage-sensitive molecular mechanisms are associated with the tissue-specificity of traits and diseases. *Comput. Struct. Biotechnol. J.* **18**, 4024–4032.
10. Barshir, R., Hekselman, I., Shemesh, N., Sharon, M., Novack, L., Yeger-Lotem, E., (2018). Role of duplicate genes in determining the tissue-selectivity of hereditary diseases. *PLoS Genet.* **14**, e1007327
11. Gamazon, E.R., Segre, A.V., van de Bunt, M., Wen, X., Xi, H.S., Hormozdiari, F., et al., (2018). Using an atlas of gene regulation across 44 human tissues to inform complex disease- and trait-associated variation. *Nature Genet.* **50**, 956–967.
12. Marbach, D., Lamparter, D., Quon, G., Kellis, M., Kutalik, Z., Bergmann, S., (2016). Tissue-specific regulatory circuits reveal variable modular perturbations across complex diseases. *Nature Methods* **13**, 366–370.
13. GTEx Consortium. (2020). The GTEx Consortium atlas of genetic regulatory effects across human tissues. *Science* 369, 1318–1330.
14. Simonovsky, E., Sharon, M., Ziv, M., Mauer, O., Hekselman, I., Jubran, J., et al., (2021). A tissue-aware machine learning framework enhances the mechanistic

understanding and genetic diagnosis of Mendelian and rare diseases. *bioRxiv*. 2021.02.16.430825.
15. Shemesh, N., Jubran, J., Dror, S., Simonovsky, E., Basha, O., Argov, C., et al., (2021). The landscape of molecular chaperones across human tissues reveals a layered architecture of core and variable chaperones. *Nature Commun.* **12**, 2180.
16. Basha, O., Argov, C.M., Artzy, R., Zoabi, Y., Hekselman, I., Alfandari, L., et al., (2020). Differential network analysis of multiple human tissue interactomes highlights tissue-selective processes and genetic disorder genes. *Bioinformatics* **36**, 2821–2828.
17. Barshir, R., Shwartz, O., Smoly, I.Y., Yeger-Lotem, E., (2014). Comparative analysis of human tissue interactomes reveals factors leading to tissue-specific manifestation of hereditary diseases. *PLoS Comput. Biol.* **10**, e1003632
18. Li, X., Zhang, P., (2017). Genetic determinants of myocardial dysfunction. *J. Med. Genet.* **54**, 1–10.
19. Greene, C.S., Krishnan, A., Wong, A.K., Ricciotti, E., Zelaya, R.A., Himmelstein, D.S., et al., (2015). Understanding multicellular function and disease with human tissue-specific networks. *Nature Genet.* **47**, 569–576.
20. Boyle, E.A., Li, Y.I., Pritchard, J.K., (2017). An Expanded View of Complex Traits: From Polygenic to Omnigenic. *Cell* **169**, 1177–1186.
21. Sharon, M., Vinogradov, E., Argov, C.M., et al., (2022). The differential activity of biological processes in tissues and cell subsets can illuminate disease-related processes and cell type identities. *Bioinformatics (Oxford, England)*, btab883.
22. Luo, L., Zheng, C., Wang, J., Tan, M., Li, Y., Xu, R., (2019). Analysis of disease organ as a novel phenotype towards disease genetics understanding. *J. Biomed. Inform.* **95**, 103235
23. Investigators, G.P.P., Smedley, D., Smith, K.R., Martin, A., Thomas, E.A., McDonagh, E.M., et al., (2021). 100,000 Genomes Pilot on Rare-Disease Diagnosis in Health Care - Preliminary Report. *N. Engl. J. Med.* **385**, 1868–1880.
24. Jia, P., Dai, Y., Hu, R., Pei, G., Manuel, A.M., Zhao, Z., (2020). TSEA-DB: a trait-tissue association map for human complex traits and diseases. *Nucleic Acids Res.* **48**, D1022–D1030.
25. Eraslan, G., Drokhlyansky, E., Anand, S., Subramanian, A., Fiskin, E., Slyper, M., et al., (2021). Single-nucleus cross-tissue molecular reference maps to decipher disease gene function. *bioRxiv*.
26. Dai, Y., Hu, R., Manuel, A.M., Liu, A., Jia, P., Zhao, Z., (2021). CSEA-DB: an omnibus for human complex trait and cell type associations. *Nucleic Acids Res.* **49**, D862–D870.
27. Plasschaert, L.W., Zilionis, R., Choo-Wing, R., Savova, V., Knehr, J., Roma, G., et al., (2018). A single-cell atlas of the airway epithelium reveals the CFTR-rich pulmonary ionocyte. *Nature* **560**, 377–381.
28. Montoro, D.T., Haber, A.L., Biton, M., Vinarsky, V., Lin, B., Birket, S.E., et al., (2018). A revised airway epithelial hierarchy includes CFTR-expressing ionocytes. *Nature* **560**, 319–324.
