# Supplementary material for: PDBx/mmCIF Ecosystem: Foundational Semantic Tools for Structural Biology
Source: J Mol Biol. Author manuscript; Available in PMC 2023 Jun 26. (PMC10292674; doi:10.1016/j.jmb.2022.167599)
Supplement: Article [file NIHMS1907597-supplement-Article.zip › SVMyr--A-Web-Server-Detecting-Co--and-Post-translatio_2022_Journal-of-Molecu.pdf]

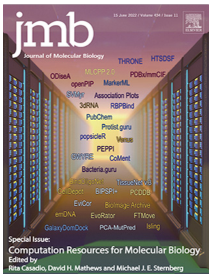

SVMyr: A Web Server Detecting Co- and Post-translational Myristoylation in Proteins

Giovanni Madeo [†] **Castrense Savojardo** [†] **Pier Luigi Martelli** [*] **and Rita Casadio**

Biocomputing Group, *Dept. of Pharmacy and Biotechnology, University of Bologna, Italy* [1]

Correspondence to Pier Luigi Martelli: *pierluigi.martelli@unibo.it (P.L. Martelli)*
https://doi.org/10.1016/j.jmb.2022.167605
Edited by Michael Sternberg

Abstract

Myristoylation (MYR) is a protein modification where a myristoyl group is covalently attached to an exposed (N-terminal) glycine residue. Glycine myristoylation occurs during protein translation (co-translation) or after (post-translation). Myristoylated proteins have a role in signal transduction, apoptosis, and pathogen-mediated processes and their prediction can help in functionally annotating the fraction of proteins undergoing MYR in different proteomes. Here we present SVMyr, a web server allowing the detection of both co- and post-translational myristoylation sites, based on Support Vector Machines (SVM). The input encodes composition and physicochemical features of the octapeptides, known to act as substrates and to physically interact with N-myristoyltransferases (NMTs), the enzymes catalyzing the myristoylation reaction. The method, adopting a cross validation procedure, scores with values of Area Under the Curve (AUC) and Matthews Correlation Coefficient (MCC) of 0.92 and 0.61, respectively. When benchmarked on an independent dataset including experimentally detected 88 medium/high confidence co-translational myristoylation sites and 528 negative examples, SVMyr outperforms available methods, with AUC and MCC equal to 0.91 and 0.58, respectively. A unique feature of SVMyr is the ability to predict post-translational myristoylation sites by coupling the trained SVMs with the detection of caspase cleavage sites, identified by searching regular motifs matching upstream caspase cleavage sites, as reported in literature. Finally, SVMyr confirms 96% of the UniProt set of the electronically annotated myristoylated proteins (31,048) and identifies putative myristoylomes in eight different proteomes, highlighting also new putative NMT substrates. SVMyr is freely available through a user-friendly web server at https://busca.biocomp.unibo.it/lipipred.

© 2022 The Author(s). Published by Elsevier Ltd. This is an open access article under the CC BY license (http://creativecommons.org/licenses/by/4.0/).

Introduction

Glycine myristoylation is a type of protein lipidation in which a myristoyl group is covalently bound by an amide bond to the alpha-amino group of an exposed glycine residue, co- or post-translationally.[1] In the former case, the myristoylation involves a glycine exposed after methionine excision (N-terminal glycine) and the process occurs on the new forming chain still in complex with the ribosome. In the latter case, the involved glycine is exposed upon caspase cleavage, mainly during apoptosis[1] and in other processes where caspases are involved, such as cell proliferation, tumor suppression, cell differentiation, and neural development.[2] Myristoylated proteins have a role in signal transduction, apoptosis and pathological processes induced by viruses and fungi.[3,4] They represent about 2% of the proteins in some eukaryotic proteomes.[4]

Both co- and post-translation myristoylations are catalyzed by the enzymes N-myristoyltransferases (NMTs),[1] whose substrate is a myristoyl-CoA

0022-2836/© 2022 The Author(s). Published by Elsevier Ltd.This is an open access article under the CC BY license (http://creativecommons.org/licenses/by/4.0/).

group. Only 35 NMT sequences, from 23 eukaryotes and 3 viruses, are manually reviewed, and included in SwissProt (the manually annotated and curated section of UniProt, (https://www.uniprot.org/)). Crystallography studies of NMTs from seven organisms are reported in the Protein Data Bank (PDB, 34 entries, https://www.rcsb.org/). Ten structures, all from humans and yeast, where the NMT enzyme is resolved in complex with substrates, show that only the first eight residues of the protein undergoing myristoylation directly interact with the enzyme: the first five residues, including the initial Gly are accommodated in the pocket, while the remaining three residues interact with the enzyme surface.[5–9] A recent high resolution crystallographic study on human NMT suggests that also N-terminal lysine can undergo myristoylation.[10] Myristoylation has been detected at the N-terminal Cys residues in few bacterial inner and outer membrane proteins.[11] However, protein examples with functional annotations consistent with the above observations are still lacking in UniProt/SwissProt.

Presently, UniProt lists NMT enzymes in 4,845 organisms, including eukaryotes, viruses, bacteria, and archaea. Consequently, we may infer that the number of putative myristoylated (MYR) proteins can be very high.[11]

Several experimental methods are available to study myristoylation *in vivo* or *in vitro*.[11] The classical approach relies on radioactive labeled myristic acid (see [1] and references therein). Myristic acid analogs like alkyne or azide moieties are adopted in bio-orthogonal approaches. They are metabolically incorporated in proteins and have affinity to various probes, such as fluorophores or biotin.[12] A high-throughput technique introduced macroarrays to measure NMTs activity in the presence of octapeptides by monitoring the formation of NADH by fluorescence.[13]

In parallel to experimental methods, trying to identify what is called the myristoylome,[11] several *in silico* approaches have been developed, tackling the problem of detecting myristoylated proteins. A regular expression for MYR proteins is in PROSITE (https://prosite.expasy.org/; Entry:PS00008)[14]: G [^DEFHKPRWY]XX[ACGNST][^P] (where ^ denotes the exclusion of residues listed and X represents any residue). The pattern has been further refined after crystallography studies into G[^DEFRWY]X[^DEKR][ACGST][KR].[9] Besides regular expressions, computational methods are available. The first method proposed was the NMT predictor,[15] based on a 17-residues motif derived by crystal structures and biochemical analysis of NMT proteins, and known substrate sequences. Afterwards, a neural networks-based method named Myristoylator (NMT_NN) was developed,[16] trained on the same dataset of myristoylated proteins used by the MYR predictor, and introducing a negative training set of non-myristoylated proteins. Finally, another available method is TermiNator3, which makes predictions based on pattern scanning.[17] These methods can only detect co-translational myristoylation. Interestingly, UniProt annotates experimentally determined N-myristoylation sites and sites predicted by NMT and NMT_NN predictors, provided that the prediction is consistent with other known properties of the protein, which include belonging to a family in which the modification is known to occur (https://www.uniprot.org/help/lipid).

Here, we present SVMyr, a novel web server that, differently from other previous approaches, can address both the detection of co- and post-translational myristoylation sites. The method behind is an ensemble of Support Vector Machines (SVMs) processing information extracted from the Glycine-N-terminal octapeptides which are directly involved in the interaction with the NMT enzymes. The input of SVMs consists of a combination of features including a scoring function computed from a novel Position Specific Scoring Matrix (PSSM) developed in this study, and physicochemical characteristics of the octapeptide.

SVMyr was trained adopting a cross validation procedure on a set comprising 232 experimentally validated co-translationally myristoylated proteins, from 37 organisms, and it scores with values of Area Under the Curve (AUC) and Matthews Correlation Coefficient (MCC) of 0.92 and 0.61, respectively. Furthermore, in a stringent benchmark derived from literature,[9] and comprising 88 and 528 co-translationally myristoylated (positive) and non-myristoylated (negative) proteins, (derived from *Homo sapiens*, *Arabidopsis thaliana* and four parasites for which the myristoylome was tested *in vivo*,[18–21] SVMyr reports the highest results among all the co-translational myristoylation predictors available, scoring with a MCC value of 0.58.

SVMyr is the first method allowing the detection of internal (post-translational) myristoylation sites in Metazoa. This task is performed with a pattern search for putative caspase cleavage sites, followed by the prediction of the exposed Gly-starting octapeptide. In a benchmarking experiment, SVMyr detects all the post-translational (internal) myristoylation sites functionally annotated in SwissProt with an experimental label, and 7 out of 11 human proteins of a previous experimentally validated data set not included in SwissProt.[22]

SVMyr is provided to the scientific community via a user-friendly and freely accessible web server available at https://busca.biocomp.unibo.it/lipipred.

Materials and Methods

Datasets for training/testing the method

For training/testing the method, three datasets were generated in this study: (i) a training dataset;

(ii) a blind test set adopted for evaluating SVMyr generalization performance on co-translational myristoylation prediction; (iii) a testing dataset for evaluating SVMyr performance on post-translational (internal) myristoylation site prediction. The first two datasets include both positive (i.e., myristoylated proteins) and negative (non-myristoylated proteins) examples. Datasets are summarized in numbers in Table 1S and available at: https://busca.biocomp.unibo.it/lipipred/datasets.

Training dataset

The positive training dataset comprises 232 protein sequences derived from UniProKB/SwissProt (release 2021_4).[23] Proteins are from 37 organisms, including Viruses (Figure 1S). For downloading, we constrained our search to experimentally validated (evidence code ECO:0000269) proteins with the annotation “N-myristoyl glycine” in PTM field, ending up with 272 proteins. For each protein, the Gly-starting octapeptide was extracted and proteins with identical octapeptides were clustered together, choosing one representative per cluster for a total of 232 examples.

A negative training dataset, comprising non-myristoylated proteins, was derived from an *in vitro* study described in literature.[9] In this study, authors measure the enzymatic activity of the N-myristoyltransferase (NMT) in the presence of diverse octapeptides using macro-arrays. The study provides a dataset of 834 positive examples, including 291 proteins from *Homo sapiens* and 495 from *Arabidopsis thaliana*. From the same experiment we extracted 1126 negative examples with a corresponding univocal mapping to UniProt proteins, including 268 octapeptides from *Homo sapiens* and 857 from *Arabidopsis thaliana*.

Optimal training requires balanced positive and negative sets and non-redundancy among cross-validation sets. For this reason, we randomly selected 232 of the 1126 negative examples.

As a result, the training data set includes overall 464 octapeptides from proteins, apparently endowed with experimental validation both for the positive and negative sets. As a measure of similarity, we adopted a Hamming distance, requiring pairs of octapeptides with distance lower than four residues to be in the same subset. We then adopted a ten-fold cross validation procedure. Sequence redundancy among the 10 subsets was avoided by collecting similar octapeptides into the same cross-validation subset. By this, the number of octapeptides in each cross-validation set ranges from 42 to 58.

Blind test set for co-translational myristoylation

To test SVMyr in the myristoylated protein detection task, and to compare its performances with other methods available, we derived a blind test set from *in vitro* and *in vivo* experiments.[9,18–21] We collected the substrate octapeptides with the experimental constraint “high/medium confidence” and applied a Hamming distance threshold of <4 residues to avoid redundancy. Our positive blind test set includes 88 positive examples: 15 from *Homo sapiens,* 34 from *Arabidopsis thaliana,* 18 from *Trypanosoma brucei,* 5 from *Trypanosoma cruzi*, 11 from *Leishmania donovani*, and 5 from *Plasmodium falciparum* (see https://busca.biocomp.unibo.it/lipipred/datasets).

528 negative examples for the blind test set were derived after reducing by similarity from the negative set described in 9, (503 octapeptides not included in the training set), and from 25 proteins in SwissProt, with an annotated N-terminal acetyl glycine experimentally validated (ECO:0000269) (see https://busca.biocomp.unibo.it/lipipred/datasets).

Test set for post-translational myristoylation

To test our SVMyr in the task of detecting internal, post-translational myristoylation sites, we derived from SwissProt an additional dataset including 4 proteins experimentally annotated with the feature (3 human and one bovin). Furthermore, we included 11 proteins with a reported post-translational myristoylation site, described in an *in vivo* experiment with induced apoptosis,[22] and not included in UniProt (see https://busca.biocomp.unibo.it/lipipred/datasets).

Datasets for large scale SVMyr application

UniProt MYR proteins and complete proteomes

Searching for “N-myristoyl glycine” in the Post Translational Modification (PTM) Lipidation field we find 32,750 entries, being 95.5% of them from viruses. The remaining proteins (1476) are from Eukaryotes and Bacteria (only one). However very few are labeled as experimentally validated and present in the manually curated SwissProt database, included in UniProt (272, see Training dataset).

The remaining automatically annotated proteins (either with UniRule (https://www.uniprot.org/help/unirule), propagating similarity, or predicted with NMT or NMT_NN[15]) include 32,476 proteins that we filtered with SVMyr.

SVMyr predicts co- and post-translational myristoylation sites in complete proteomes. For this analysis, we downloaded complete reference proteomes from UniProt (https://www.uniprot.org/proteomes/),[23] including *Homo sapiens, Arabidopsis thaliana, Mus musculus, Saccharomyces cerevisiae,* and the parasites *Trypanosoma brucei*,

Trypanosoma cruzi, *Leishmania donovani*, (strain BPK282A1) and *Plasmodium falciparum*.[9,18–21]

Svmyr implementation

Input scheme

SVMyr is the first web server publicly available implementing a Support Vector Machine (SVM)-based method tackling the problem of detecting co-translational and post-translational myristoylation sites. Due to the paucity of available data for training and the low dimensionality of the problem at hand, more sophisticated and complex models, including deep learning, were not considered. Indeed, only some 232 proteins in SwissProt have PTM myristoylated sites with experimental validation (see Training set).

Gly-octapeptide input includes seven scores (with the exception of the first Gly position, which is conserved) towards a Position-Specific Scoring Matrix (PSSM) (see below), and average physicochemical characteristics (including hydrophobicity, charge, secondary structure propensity, and size).

For each of the ten cross-validation runs, PSSM is computed by stacking in a gapless mode all the Gly-octapeptides included in the positive training set. A profile is then computed as the frequency of the 20 residue types in each column in the alignment. A second, background profile to compute the log-odd scores in the PSSMs, is obtained collecting all the N-terminal octapeptides of Gly or Met-Gly starting eukaryotic proteins included in SwissProt (14,304 non identical octapeptides). Excluding the first Gly residue position, the PSSM value for each one of the remaining seven positions is computed as:

$$PSSM_{R,i} = -\log\frac{f_{R,i}}{b_{R,i}} \quad (1)$$

where $f_{R,i}$ is the frequency observed for residue type R in position i in the profile of myristoylated proteins and $b_{R,i}$ is the frequency observed for residue type R in position i in the background distribution.

The input Gly-octapeptide is then represented with seven positional scores (Gly is excluded) derived from the PSSM, and with 5 physicochemical features averaged over the seven variable positions of the octapeptide.

Physicochemical residue features include charge (considering charge +1 for Arg and Lys, and −1 for Asp and Glu), size as derived from AAindex[24] (https://www.genome.jp/aaindex), hydrophobicity according to the Kyte-Doolittle scale,[25] and propensity towards alpha-helix and beta-strand secondary structures.[26] By this, each Gly-octapeptide is encoded by a 12-dimensional vector.

We optimized the hyperparameters of the SVM adopting a 10-fold cross-validation and a grid-search. Each run of cross-validation was carried out using eight subsets for training, one for validation and one for testing. The optimal regularization parameters C and γ were determined over the validation sets and found to be equal to 1 and 0.07, respectively. In order to train and test SVMs, we adopt the Python package scikit-learn (https://scikit-learn.org).

SVM ensemble prediction and evaluation procedure

SVMyr predicts myristoylation sites by combining, with an ensemble majority scheme, the results of the 10 SVM models trained during the cross-validation procedure. Associated myristoylation probability is computed as the average of the output probabilities of the 10 SVMs in the ensemble and the input octapeptide is MYR predicted when the probability value is $\geq$0.50.

We assessed the performance of SVMyr on the blind test (see above Blind test set for co-translational myristoylation). We evaluated the True Positive Rate (TPR), the Positive Predictive Value (PPV), the False Positive Rate (FPR), the Matthews Correlation Coefficient (MCC) and the harmonic mean between TPR and PPV (F1-score). Index definitions are in Supplementary Material. Moreover, Receiver Operating Characteristic (ROC) curves and their relative Area Under the Curve (AUC) are computed, when necessary.

Detection of post-translational myristoylation sites

SVMyr can search for internal myristoylation sites, in which the attachment of the myristic group takes place at N-terminal glycine residues of polypeptides generated upon proteolytic cleavage by caspase enzymes. This phenomenon is documented in Metazoa.[15] Apoptotic caspases seem to be mainly involved in the cleavage of post-translational MYR proteins. To retrieve caspase cleavage site motifs, we refer to the Eukaryotic Linear Motifs (ELM) database.[27] In this database, we found four apoptotic caspase cleavage site motifs (Table 2S): one validated motif (ELME000321), for caspases 3/7, and three motifs reported as candidates in ELM (http://elm.eu.org/elms/candidates), for caspases 2, 6 and 9. Gly-starting octapeptides identified with this procedure are then classified with the ensemble SVMyr procedure.

Results

Predictive performance of SVMyr

SVMyr was tested by adopting a 10-fold cross-validation procedure over the training set, which includes 232 myristoylated proteins and 232 non-myristoylated proteins. In this testing procedure, SVMyr scores with True Positive Rate (TPR), Positive Predicted Value (PPV), False Positive Rate (FPR), Matthews Correlation Coefficient

(MCC), and F1-score equal to 65%, 87%, 8%, 0.61, and 75%, respectively (Table 1).

We benchmark SVMyr together with all the different available methods on the SVMyr blind test set (Table 1). The comparative benchmark includes NMT,[15] Myristoylator (NMT_NN),[16] TermiNator3[17] and two available regular expressions (Regular Motif A[9] and B[14]). SVMyr outperforms all the other available methods in terms of Positive Predictive Value (PPV) (62%), MCC (0.58) and F1-score (64%). TermiNator3 scores with a higher True Positive Rate (TPR) value (81%); however, it is endowed with a much higher rate of false positives (42% compared to SVMyr 7%). This is confirmed also by the lower MCC value. Similar considerations hold for results obtained with Regular Motif B.[14] In turn, Regular Motif A[9] scores with a higher PPV (69%), but with poorer TPR (20%). NMT reports a slightly lower False Positive Rate (5%; SVMyr obtains 7%), however associated to a much lower TPR (44%). To assess whether the performances are affected by residual redundancy between training and testing set, we implemented a simple nearest-neighbor baseline predictor based on the Hamming distance. Briefly, the method assigns to a test peptide the class of the closest peptide in the training set. The performance of this baseline method is very low (MCC = 0.15) and far away from that achieved by the worst-performing non-trivial method tested in our comparison (achieving MCC = 0.27, see Table 1).

In Figure 1, ROC curves are computed for SVMyr and Myristoylator, over the blind test set. The two methods are the only ones providing a score that allows computing false and true positive rates at different score thresholds. The performances of all the other available methods are represented by single dots (Figure 1). SVMyr scores with the highest AUC (0.91); Myristoylator has an AUC of 0.79. NMT, TermiNator3 and the two Regular Motif -based methods score lower.

Detection of post-translational myristoylation sites

As a unique feature for Metazoa, SVMyr allows searching for internal myristoylation sites, which are exposed upon caspase cleavage. These are detected by scanning for the regular expressions reported in literature.[27] Considering the test set for post-translational myristoylation including 15 proteins (see data sets), the procedure correctly identifies the upstream caspase cleavage site motifs and the post-translational myristoylation of the exposed Glycine in 11 of them.

SVMyr large scale application

We finally tested SVMyr on 31,048 proteins in UniProt, annotated with the PTM modification N-glycine co-translational myristoylation without experimental validation. We confirmed 96% of this set.

Proteome-wide analyses

We filtered with SVMyr complete reference proteomes of eight organisms: *Homo sapiens, Arabidopsis thaliana, Mus musculus, Saccharomyces cerevisiae*, *Trypanosoma brucei, Trypanosoma cruzi, Leishmania donovani*, and *Plasmodium falciparum*. For each proteome, we predict co-translational myristoylation sites (Tables 3S and 4S). For *H. sapiens* and *M. musculus* we searched also for post-translational myristoylation sites (Table 3S). In *H. sapiens* and *M. musculus*, SVMyr predicts 902 and 719 proteins with co-translational myristoylation sites, and 1422 and 1147 proteins with post-translational myristoylation, respectively (Table 3S). Table 4S lists the number of proteins predicted co-myristoylated in *Arabidopsis thaliana* (615), *S. cerevisiae* (39), *T. brucei* (119), *T. cruzi* (194), *L. donovani* (119), and *P. falciparum* (61). As expected,[11] the percentage of predicted co-myristoylation sites ranges from 1 to 2% of the entire proteome.

For all organisms, SVMyr correctly identifies most of the annotated co-translational myristoylation sites (Tables 3S and 4S). Indeed, the overall TPR of SVMyr with respect to previous annotations is 74% (902 predicted proteins over 1227 annotated ones). Considering only experimental annotations, the overall TPR is 72% (732 over 1014) and

Table 1 SVMyr benchmarking.

Method	Dataset	TPR° (%)	PPV° (%)	FPR° (%)	MCC°	F1° (%)
SVMyr	Cross-validation	65	87	8	0.61	75
SVMyr	Blind test set*	67	62	7	0.58	64
NMT[15]		44	60	5	0.46	52
Myristoylator[16]		48	40	12	0.33	43
TermiNator3[17]		81	24	42	0.27	37
Regular Motif A[9]		20	69	2	0.33	32
Regular Motif B[14]		72	36	23	0.39	48

* Blind test set (blind for SVMyr). SVMyr is scored adopting a cross validation procedure and compared on a blind test set to available methods[15–17,9,14] performing the task of co-translational myristoylation prediction.

° Scoring indexes are defined in Supplementary Material.

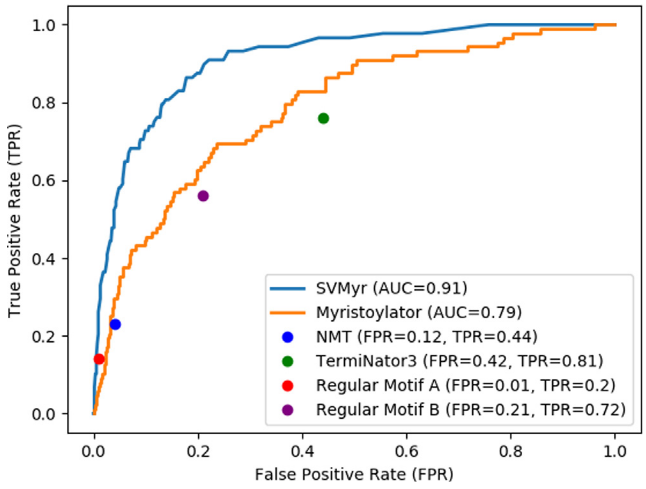

Figure 1. Comparison with other methods. Receiver Operating Characteristic (ROC) curves of SVMyr and Myristoylator (NMT_NN)[16] over the blind test set (see Methods for details). For the other methods, it was not possible to produce a ROC curve since they provide a binary prediction as output and not a score. Therefore, their performance is represented by dots.

ranges across species from 64% (for human, 238 over 373) to 100% (for *S. cerevisiae*, 12 over 12). These values are slightly lower when restricting to proteins not included in the training set: the overall TPR is 69% (576 over 832), ranging from 54% (for human, 130 over 240) to 100% (for Mouse, 9 over 9, and *S. cerevisiae*, 1 over 1). These data are largely in agreement with the evaluation scores rigorously evaluated on an independent test set (Table 1).

The list of all co-translational myristoylation targets predicted with SVMyr on the eight proteome is available in Supplementary Data. For each target, we indicate: (i) if it is a new annotation or it was previously annotated, (experimentally, computationally, or by transfer from annotated isoforms of the same gene); (ii) the N-terminal octapeptide; (iii) if the protein is part of the training set of SVMyr; (iv) the score provided by SVMyr; (v) the annotation in terms of Gene Ontology and Pfam/InterPro domains, as recovered from the corresponding UniProt entry.

As to the co-translational myristoylated predicted portions of the 8 different proteomes, we find proteins that, consistently with the training dataset, are predominantly covered by similar Pfam domains, including Pkinase, Arf, EF-hand_7, PK_Tyr_Ser-Thr and G-alpha (see Figure 2S), suggesting that predictions retrieve the same protein families present in the training set (compare also with genes highlighted in 28). MYR substrates without any experimental validation or electronic annotation in UniProt, available for future experimental validations, are 931 (Tables 3S and 4S) and are available for downloading from the web site (https://busca.biocomp.unibo.it/lipipred/datasets/).

The SVMyr web server

SVMyr is released as a public web server accessible at https://busca.biocomp.unibo.it/lipipred. In the server home page, the user can upload up to 5000 protein sequences in FASTA format, either pasting them in the input window or by file uploading. The user can choose to perform different types of predictions: (i) co-translational myristoylation only, (ii) post-translational myristoylation only, or (iii) both type of predictions at the same time. Post-translational myristoylations are relevant only when Metazoan sequences are submitted. When searching for post-translational myristoylation cleavage sites, the user can decide whether adopting all caspase cleavage-site motifs available at the Eukaryotic Linear Motif (ELM)[27] database (Caspase 2, 6, 3–7 and 9), or restricting the search to the Caspase 3–7 only (this cleavage-site motif is the only one currently validated in ELM). After submission, a waiting page redirects towards the result page organized into different tables (Figure 2).

In the top-page table (Figure 2(A)), the system reports main information about the submitted job, including: the job ID, submission time, the number of submitted sequences, the type of prediction performed (co-/post-translational or both) and, when post-translational prediction is performed, the set of caspase cleavage-site motifs adopted.

When co-translational prediction is performed, a second table (Figure 2(B)) lists the results for all the input proteins. For each protein the reported information includes the protein accession ID, the prediction, the N-terminal octapeptide analysed by SVMyr, the probabilistic score associated to the

(A) Summary of submitted job

**Job ID:**	7f32b91d-90e3-4997-919a-cc9778beed66
**Submission time:**	Feb. 17, 2022, 10:25 a.m. (GMT)
**Number of submitted sequences:**	4
**Prodiction mode:**	Co- and post-translational
**Caspase-cleavage motif set:**	Caspase 3-7, Caspase 2, Caspase 6, Caspase 9

(B) Co-translational myristoylation prediction

Protein Accession/ID	Prediction	Position	Octapeptide	Score	Note
A8MQ27	N-myristoyl glycine (co-translational)	2	GNTVHRTL	0.87	Highly probable
Q9H6R6-2	N-myristoyl glycine (co-translational)	2	GTFCSVIK	0.84	Highly probable
Q06002	Not myristoylated (co-translational)	-	-	-	No N-terminal Glycine
P42858	Not myristoylated (co-translational)	-	-	-	No N-terminal Glycine

(C) Post-translational myristoylation prediction

Protein Accession/ID	Prediction	Position	Caspase cleavage I Octapeptide	Score	Caspase type	Note
A8MQ27	N-myristoyl glycine (post-translational)	161	SVND I GEPVLFHC	0	Cleavage; by Caspase 3-7	Improbable
P42858	N-myristoyl glycine (post-translational)	551	DLND I GTQASSPI	0.9	Cleavage; by Caspase 3-7	Highly probable
P42858	N-myristoyl glycine (post-translational)	524	SATD I GDEEDILS	0	Cleavage; by Caspase 3-7	Improbable
Q06002	N-myristoyl glycine (post-translational)	433	DVPD I GGKISKAF	0.74	Cleavage; by Caspase 3-7	Probable
Q06002	N-myristoyl glycine (post-translational)	533	EKED I GLKEEGGP	0	Cleavage; by Caspase 2	Improbable
Q9H6R6-2	Not myristoylated (post-translational)	-	-	-	-	No caspase cleavage sites found

Figure 2. Examples of the main SVMmyr output tables. Proteins with ID in blue color are linked to detailed results showing a graphical visualization of the post-translational myristoylated sites.

prediction. On this basis, myristoylation sites are labeled as highly probable (score $\geq$ 0.8), probable (0.5 $\leq$ score < 0.8), or improbable (score < 0.5).

Post-translational myristoylation results (when included) are shown in a third table (Figure 2(C)). Besides the fields mentioned for the co-translational table, two additional columns report the caspase cleavage motif matching the sequence and the segment of the sequence containing the caspase cleavage site followed by the Gly-starting octapeptide.

Each entry of the post-translational table is linked to a page reporting detailed results. An interactive feature viewer shows the predicted caspase cleavage sites and myristoylation sites along the residue sequence. Detailed information on the caspase cleavage motif matching the sequence (including the caspase type, the cleavage site and the position of the match along the sequence) are reported in three additional tables, specific for: (i) all caspase-cleavage sites that generate a Gly-starting peptide, independently of their prediction; (ii) predicted post-translational myristoylation sites labelled as highly-probable and probable (score $\geq$ 0.5); and (iii) predicted post-translational myristoylation sites labelled as improbable (score < 0.5).

Conclusions

We present SVMyr, a web server implementing a new method based on an ensemble of SVMs for the detection of myristoylated proteins. The input is based on the composition of known myristoylated substrate octapeptides and their physicochemical features (hydrophobicity, charge, size, tendency towards helix or strand formation). SVMyr is trained on a dataset including only experimentally validated substrate proteins as downloaded from SwissProt and from selected experiments described in literature. When tested on a non-redundant independent dataset of experimentally validated proteins, SVMyr outperforms all the available methods tackling this task, including NMT and Myristoylator (NMT_NN) adopted by UniProt in order to annotate co-translational myristoylation sites.

Moreover, as a distinctive feature, SVMyr allows the detection of post-translational myristoylation sites in Metazoa, implementing a pattern search for caspase-cleavage sites. The system reports good performances when tested on the small set of validated proteins available.

SVMyr is a fast method, able to scan the entire human proteome in about 15 minutes, with an average running time of about 0.1 seconds per protein. This makes SVMyr well-suited for genome-wide analysis.

The SVMyr server is freely available for the scientific community at https://busca.biocomp.unibo.it/lipipred. Furthermore, SVMyr provides a usable and accessible user interface where all the predicted and the relevant information can be exported in JSON format.

CRediT authorship contribution statement

Giovanni Madeo: Data curation, Formal analysis, Software, Validation, Writing – original draft, Writing – review & editing. **Castrense Savojardo:** Conceptualization, Supervision, Formal analysis, Software, Validation, Writing – original draft, Writing – review & editing. **Pier Luigi Martelli:** Conceptualization, Supervision, Formal analysis, Validation, Writing – original draft, Writing – review & editing. **Rita Casadio:** Conceptualization, Supervision, Formal analysis, Validation, Writing – original draft, Writing – review & editing.

DATA AVAILABILITY

Data are available on the web server

Acknowledgements

The work was supported by PRIN2017 grant (project 2017483NH8_002), delivered to CS by the Italian Ministry of University and Research. We acknowledge ELIXIR-IIB, the Italian node of the ELIXIR infrastructure.

Declaration of interests

The authors declare that they have no known competing financial interests or personal relationships that could have appeared to influence the work reported in this paper.

Appendix A. Supplementary material

Supplementary data to this article can be found online at https://doi.org/10.1016/j.jmb.2022.167605.

Received 29 November 2021;
Accepted 19 April 2022;
Available online 26 April 2022

Keywords:
protein myristoylation;
post-translational modification;
lipidation;
support vector machines;
position specific scoring matrix

† Equally contributed to this work.

References

1. Jiang, H., Zhang, X., Chen, X., Aramsangtienchai, P., Tong, Z., Lin, H., (2018). Protein Lipidation: Occurrence, Mechanisms, Biological Functions, and Enabling Technologies. *Chem. Rev.* **118**, 919–988.
2. Shalini, S., Dorstyn, L., Dawar, S., Kumar, S., (2015). Old, new and emerging functions of caspases. *Cell. Death Differ.* **22**, 526–539.
3. Martin, D.D.O., Beauchamp, E., Berthiaume, L.G., (2011). Post-translational myristoylation: Fat matters in cellular life and death. *Biochimie* **93**, 18–31.
4. Meinnel, T., Dian, C., Giglione, C., (2020). Myristoylation, an Ancient Protein Modification Mirroring Eukaryogenesis and Evolution. *Trends Biochem. Sci.* **45**, 619–632.
5. Weston, S.A., Camble, R., Colls, J., Rosenbrock, G., Taylor, I., Egerton, M., Tucker, A.D., Tunnicliffe, A., et al., (1998). Crystal structure of the anti-fungal target N-myristoyl transferase. *Nature Struct. Biol.* **5**, 213–221.
6. Farazi, T.A., Waksman, G., Gordon, J.I., (2001). Structures of *Saccharomyces cerevisiae N* -myristoyltransferase with Bound MyristoylCoA and Peptide Provide Insights about Substrate Recognition and Catalysis. *Biochemistry* **40**, 6335–6343.
7. Bhatnagar, R.S., Fütterer, K., Farazi, T.A., Korolev, S., Murray, C.L., Jackson-Machelski, E., Gokel, G.W., Gordon, J.I., et al., (1998). Structure of N-myristoyltransferase with bound myristoylCoA and peptide substrate analogs. *Nature Struct. Biol.* **5**, 1091–1097.
8. Maurer-Stroh, S., Eisenhaber, B., Eisenhaber, F., (2002). N-terminal N -myristoylation of proteins: refinement of the sequence motif and its taxon-specific differences. *J. Mol. Biol.* **317**, 523–540.
9. Castrec, B., Dian, C., Ciccone, S., Ebert, C.L., Bienvenut, W.V., Le Caer, J.-P., Steyaert, J.M., Giglione, C., et al., (2018). Structural and genomic decoding of human and plant myristoylomes reveals a definitive recognition pattern. *Nature Chem. Biol.* **14**, 671–679.
10. Dian, C., Pérez-Dorado, I., Rivière, F., Asensio, T., Legrand, P., Ritzefeld, M., Shen, M., Cota, E., et al., (2020). High-resolution snapshots of human N-myristoyltransferase in action illuminate a mechanism promoting N-terminal Lys and Gly myristoylation. *Nature Commun.* **11** Article 1132.
11. Giglione, C., Meinnel, T., (2022). Mapping the myristoylome through a complete understanding of protein myristoylation biochemistry. *Prog. Lipid Res.* **85**, 101139.

12. Hang, H.C., Wilson, J.P., Charron, G., (2011). Bioorthogonal Chemical Reporters for Analyzing Protein Lipidation and Lipid Trafficking. *Acc. Chem. Res.* **44**, 699–708.
13. Traverso, J.A., Giglione, C., Meinnel, T., (2013). High-throughput profiling of *N*-myristoylation substrate specificity across species including pathogens. *Proteomics* **13**, 25–36.
14. Hulo, N., (2006). The PROSITE database. *Nucleic Acids Res.* **34**, D227–D230.
15. Maurer-Stroh, S., Eisenhaber, B., Eisenhaber, F., (2002). N-terminal N -myristoylation of proteins: prediction of substrate proteins from amino acid sequence. *J. Mol. Biol.* **317**, 541–557.
16. Bologna, G., Yvon, C., Duvaud, S., Veuthey, A.-L., (2004). N-Terminal myristoylation predictions by ensembles of neural networks. *Proteomics* **4**, 1626–1632.
17. Martinez, A., Traverso, J.A., Valot, B., Ferro, M., Espagne, C., Ephritikhine, G., Zivy, M., Giglione, C., et al., (2008). Extent of N-terminal modifications in cytosolic proteins from eukaryotes. *Proteomics* **8**, 2809–2831.
18. Wright, M.H., Paape, D., Price, H.P., Smith, D.F., Tate, E. W., (2016). Global Profiling and Inhibition of Protein Lipidation in Vector and Host Stages of the Sleeping Sickness Parasite Trypanosoma brucei. *ACS Infect. Dis.* **2**, 427–441.
19. Roberts, A.J., Fairlamb, A.H., (2016). The N-myristoylome of Trypanosoma cruzi. *Sci. Rep.* **6** Article 31078.
20. Wright, M.H., Paape, D., Storck, E.M., Serwa, R.A., Smith, D.F., Tate, E.W., (2015). Global Analysis of Protein N-Myristoylation and Exploration of N-Myristoyltransferase as a Drug Target in the Neglected Human Pathogen Leishmania donovani. *Chem. Biol.* **22**, 342–354.
21. Schlott, A.C., Holder, A.A., Tate, E.W., (2018). N-Myristoylation as a Drug Target in Malaria: Exploring the Role of N-Myristoyltransferase Substrates in the Inhibitor Mode of Action. *ACS Infect. Dis.* **4**, 449–457.
22. Thinon, E., Serwa, R.A., Broncel, M., Brannigan, J.A., Brassat, U., Wright, M.H., Heal, W.P., Wilkinson, A.J., et al., (2014). Global profiling of co- and post-translationally N-myristoylated proteomes in human cells. *Nature Commun.* **5** Article 4919.
23. The UniProt Consortium, (2021). UniProt: the universal protein knowledgebase in 2021. *Nucleic Acids Res.* **49**, D480–D489.
24. Kawashima, S., (2000). AAindex: Amino Acid index database. *Nucleic Acids Res.* **28**, 374.
25. Kyte, J., Doolittle, R.F., (1982). A simple method for displaying the hydropathic character of a protein. *J. Mol. Biol.* **157**, 105–132.
26. Chou, P.Y., Fasman, G.D., (1974). Prediction of protein conformation. *Biochemistry* **113**, 222–245.
27. Kumar, M., Gouw, M., Michael, S., Sámano-Sánchez, H., Pancsa, R., Glavina, J., Diakogianni, A., Valverde, J.A., et al., (2020). ELM—the eukaryotic linear motif resource in 2020. *Nucleic Acids Res.* **48**, D296–D306.
28. Yuan, M., Song, Zh., Ying, Md., et al., (2020). N-myristoylation: from cell biology to translational medicine. *Acta Pharmacol Sin.* **41**, 1005–1015. https://doi.org/10.1038/s41401-020-0388-4.
