# Supplementary material for: PDBx/mmCIF Ecosystem: Foundational Semantic Tools for Structural Biology
Source: J Mol Biol. Author manuscript; Available in PMC 2023 Jun 26. (PMC10292674; doi:10.1016/j.jmb.2022.167599)
Supplement: Article [file NIHMS1907597-supplement-Article.zip › PubChem-Protein--Gene--Pathway--and-Taxonomy-Data-Collectio_2022_Journal-of-.pdf]

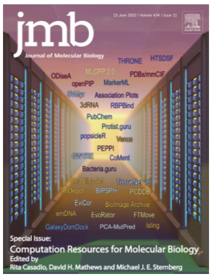

PubChem Protein, Gene, Pathway, and Taxonomy Data Collections: Bridging Biology and Chemistry through Target-Centric Views of PubChem Data

Sunghwan Kim [†] **Tiejun Cheng** [†] **Siqian He, Paul A. Thiessen, Qingliang Li, Asta Gindulyte and Evan E. Bolton** [*]

National Center for Biotechnology Information, *National Library of Medicine, National Institutes of Health, Bethesda, MD 20894, USA*

Correspondence to Evan E. Bolton: *bolton@ncbi.nlm.nih.gov (E.E. Bolton), @pubchem (E.E. Bolton)*
https://doi.org/10.1016/j.jmb.2022.167514
Edited by Michael Sternberg

Abstract

PubChem (https://pubchem.ncbi.nlm.nih.gov) is a public chemical database at the U.S. National Institutes of Health. Visited by millions of users every month, it plays a role as a key chemical information resource for biomedical research communities. Data in PubChem is from hundreds of contributors and organized into multiple collections by record type. Among these are the Protein, Gene, Pathway, and Taxonomy data collections. Records in these collections contain information on chemicals related to a given biological target (*i.e.*, protein, gene, pathway, or taxon), helping users to analyze and interpret the biological activity data of molecules. In addition, annotations about the biological targets are collected from authoritative or curated data sources and integrated into the four collections. The content can be programmatically accessed through PubChem's web service interfaces (including PUG View). A machine-readable representation of this content is also provided within PubChemRDF.

Published by Elsevier Ltd. This is an open access article under the CC BY license (http://creativecommons.org/licenses/by/4.0/).

Introduction

PubChem (https://pubchem.ncbi.nlm.nih.gov)[1–4] is a public chemical biology database, developed and maintained by the National Center for Biotechnology Information (NCBI), a center within the National Library of Medicine (NLM) at the U.S. National Institutes of Health (NIH). It is a popular information resource, visited by millions of users every month.[1] While developed to support biomedical research communities, PubChem serves a very broad user base, including data scientists, researchers, patent agents, chemical safety officers, students, and educators.

PubChem provides a large corpus of chemical information, collected from hundreds of data sources (824 sources, as of November 23, 2021) (https://pubchem.ncbi.nlm.nih.gov/source/). While the majority of these (723 sources) deposit chemical data in the form of substance and bioassay records, some serve as annotation sources, meaning that their data are integrated by PubChem to annotate chemicals and related entities (e.g., genes, proteins, pathways, and taxa). About 100 data sources in PubChem provide only annotation information.

While the majority of chemical substances in PubChem are small molecules, it contains a number of entity types, such as siRNAs, miRNAs, lipids, carbohydrates, and chemically modified nucleotides. PubChem data content includes a very large quantity of experimentally determined bioactivity data and biomedical annotations for chemicals. Importantly, it also contains information on the relationship of chemicals with other entities, such as genes, proteins, and biological pathways. This information is very useful to understand the role of chemicals in biological phenomena.

The bioactivity data contained in PubChem is primarily from large-scale high-throughput screening (HTS) initiatives. Of the 293 million bioactivities in total, 243 million (82.9%) are HTS data from the now-concluded Molecular Libraries Program (MLP) at the NIH. PubChem also contains a substantial amount of literature-extracted bioactivity data,[5] contributed by several data contributors. An example is 12 million bioactivities (4.1% of the total bioactivities) from ChEMBL.[6] It is noteworthy that the assays derived from literature typically contain a much smaller number of bioactivity data than HTS assays. On average, for example, ChEMBL assays have 8.7 bioactivities, while MLP assays have an average of 41 thousand bioactivities per assay. More detailed discussions about HTS data and literature-derived data are given in our previous papers.[2,5]

PubChem data can be accessed interactively through a web browser or programmatically through computer programs or scripts. PubChem provides multiple programmatic interfaces, such as PUG-REST[7–9] and PUG-View,[10] allowing users to exploit PubChem data in computational pipelines for machine learning and data science studies. In addition, PubChem supports bulk data download through its web-based FTP site (https://ftp.ncbi.nlm.nih.gov/pubchem) and via the download facility in PubChem Search. Especially, the FTP site hosts PubChemRDF[11] content, which refers to PubChem data formatted using the Resource Description Framework (RDF). With PubChemRDF, users can exploit semantic web technologies to readily integrate PubChem data with data from other sources.

Chemicals contained in PubChem are submitted by individual data depositors. When the depositors provide the descriptions of chemicals, they are archived in a data collection called "Substance".[12] Some depositors provide the descriptions and test results of biological assay experiments, which are archived in another data collection called "BioAssay". Because a single chemical can be provided multiple times (either by the same depositor or by multiple depositors), the Substance collection has a great deal of redundancy in its chemical coverage. To address this issue, unique chemical structures are extracted from Substance through chemical structure standardization[13] and stored in another data collection called "Compound".[12] Each record in the Substance, Compound, and BioAssay collections is called a substance, compound, and bioassay, respectively, and is assigned a numeric unique identifier called Substance ID (SID), Compound ID (CID), and Assay ID (AID), respectively. In addition, each record in PubChem data collections has a dedicated web page (called a Summary page) that presents all information available in PubChem for that record. The summary page for a given record can be accessed through a uniform resource locator (URL) in the following format:

- https://pubchem.ncbi.nlm.nih.gov/COLLECTION_NAME/RECORD_ID

where COLLECTION_NAME is the name of a data collection and RECORD_ID is the identifier assigned to a given record. For example, the following URL is for the Summary page of CID 60823 (atorvastatin):

- https://pubchem.ncbi.nlm.nih.gov/compound/60823

While the Substance and BioAssay collections are data archives, the Compound collection could be considered a knowledgebase for chemicals. Each compound record is annotated with a wide range of additional information, which is presented within its Compound Summary page. While the majority of users visit PubChem to find information on a given chemical, many users look for information on chemicals associated with a given target (*e.g.*, a particular protein, gene, pathway, or organism). However, because this information is scattered across multiple compound records, it is not easy to access through the Compound collection. For this reason, PubChem introduced additional data collections (*i.e.*, Protein, Gene, Pathway, and Taxonomy), in which each record contains information on all chemicals associated with the corresponding biological target (*i.e.*, protein, gene, pathway, and organism). Essentially, these collections provide an alternative, target-centric data view of PubChem content. In this paper, an overview of these data collections is given, including the data coverage, interactive access, programmatic access, and their addition to PubChemRDF.

Results and Discussion

Protein and Gene collections

The Protein and Gene collections contain proteins and genes, respectively, that have associated biological activity records in any bioassay archived in PubChem or that are found in any pathway in the PubChem Pathway collection (discussed in the next section). As of November 23, 2021, these two collections have 97 thousand proteins and 89 thousand genes, as shown in Table 1. While about 22 thousand proteins, encoded by eight thousand genes, are tested against in at least one biochemical assay, 56 thousand genes are tested against in RNA interference (RNAi) screening assays. On the other hand, the records in the Pathway collection involve 85 thousand proteins and 50 thousand genes. Five thousand proteins and 19 thousand genes are associated with both assay and pathway records.

Each record in these collections has a Summary page that shows bioactivity data of chemicals that are tested against the corresponding protein/gene,

Table 1 Record counts in PubChem data collections as of November 23, 2021. Current statistics can be found at the PubChem Statistics page (https://pubchemdocs.ncbi.nlm.nih.gov/statistics).

Data Collection	Live Count	Description
Substance	277,195,271	Descriptions about chemical entities provided by PubChem contributors
Compound	111,050,895	Unique chemical structures extracted from PubChem Substance records
BioAssay	1,391,562	Biological assay descriptions and test results, provided by PubChem contributors
Proteins	97,652	Protein targets tested in PubChem BioAssays and those involved in PubChem Pathways
Genes	89,270	Gene targets tested in PubChem BioAssays and those involved in PubChem Pathways
Pathway	238,597	A series of actions among molecules (chemicals, genes, and proteins) in a cell that leads to a certain product or a change in a cell.
Taxonomy	8,841	Organisms of targets tested in PubChem BioAssays and those involved in PubChem Pathways

along with additional information on the protein/gene collected from authoritative and curated sources (see the Methods and Materials section). The Protein and Gene Summary pages can be accessed via a URL containing the corresponding NCBI Protein accession or NCBI Gene ID, as shown in these examples:

- Protein Summary for NCBI Protein accession P00742 (human coagulation factor X (F10)):Protein Summary for NCBI Protein accession P00742 (human coagulation factor X (F10)): https://pubchem.ncbi.nlm.nih.gov/protein/P00742
- Gene Summary for NCBI Gene ID 1019 (human cyclin-dependent kinase 4 (CDK4)) https://pubchem.ncbi.nlm.nih.gov/gene/1019

Each section or subsection of the Summary page is assigned a fragment identifier (which indicates a location in an HTML document and is introduced by the "#" character in the URL), allowing the user to bookmark the page and section. For instance, the following URL leads to the section that shows the bioactivity data for the human 3-hydroxy-3-methylglutaryl-CoA reductase (HMGCR) gene (Gene ID 3156):

- https://pubchem.ncbi.nlm.nih.gov/gene/3156#section=Chemicals-and-Bioactivities

Note that this section shows bioactivity data from assays archived in PubChem BioAssay as well as curated bioactivity data from other resources, including DrugBank,[14] ChEMBL,[6] and the Guide to Pharmacology.[15]

These organism-specific Gene Summary pages can also be accessed through an URL containing the gene symbol and organism name, as in this example for the human epidermal growth factor receptor (EGFR) gene:

- https://pubchem.ncbi.nlm.nih.gov/gene/egfr/human
- https://pubchem.ncbi.nlm.nih.gov/gene/egfr/homo_sapiens

The symbol of a gene can be used to access an organism non-specific Gene Summary page, as in the following example for the EGFR gene:

- https://pubchem.ncbi.nlm.nih.gov/gene/egfr

This page presents a list of organism-specific EGFR genes with data in PubChem, along with the links to respective Summary pages. These organism non-specific Gene Summary pages reflect the fact that a gene in different species often has the same name and symbol when capitalization is ignored.

Pathway collection

The Pathway collection was constructed by integrating data from multiple pathway information resources,[16–26] as explained in the Materials and Methods section, and it currently has about 239,000 records (Table 1). It is noteworthy that, while different data sources may have similar records for the same pathway, PubChem does not attempt to further merge or combine them into a single record. As a result, the PubChem Pathway collection may have multiple closely-related records for a given pathway from different sources. In addition, there may be multiple near-identical pathways from the same data source where the primary difference is the species in which the pathway exists. To distinguish these closely-related records, the URL for the Summary page for each record in the Pathway collection takes the following form:

- https://pubchem.ncbi.nlm.nih.gov/pathway/SOURCE:RECORD_ID

where SOURCE is the information source for the pathway record and RECORD_ID is the identifier used for that record by the source. For example, the following URLs correspond to the Pathway Summary pages for "Glycolysis and Gluconeogenesis" from the Integrating Network Objects with Hierarchies (INOH) database (ID: MI0035772)[25] and WikiPathways (ID: WP534),[20] respectively:

- https://pubchem.ncbi.nlm.nih.gov/pathway/INOH:MI0035772
- https://pubchem.ncbi.nlm.nih.gov/pathway/WikiPathways:WP534

Note that these two pages show slightly different data content for the same pathway. The Pathway Summary page for a given record lists chemicals, proteins, and genes involved in or associated with that pathway, along with information on interactions or reactions among them.

PubChem Pathway supersedes the NCBI BioSystems database,[27] which is scheduled to be retired in 2022. For most of the BioSystems records, there are corresponding Pathway Summary pages, which can be accessed via an URL containing an NCBI BioSystems ID (BSID), as shown in this example (for BSID 198814):

- https://pubchem.ncbi.nlm.nih.gov/pathway/BSID:198814

Note that this URL also leads to the Pathway Summary for glycolysis and gluconeogenesis from WikiPathways (ID: WP534), because BSID 198814 was derived from the same WikiPathways record.

Taxonomy collection

The PubChem Taxonomy collection contains nearly nine thousand taxa that are associated with bioassay or pathway records in PubChem (Table 1). The Summary page for a taxon can be accessed through an URL containing the corresponding identifier used in the NCBI Taxonomy database. For example, the following URL directs to the Summary page for severe acute respiratory syndrome coronavirus 2 (SARS-CoV-2) (Taxonomy ID 2697049):

- https://pubchem.ncbi.nlm.nih.gov/taxonomy/2697049

This page shows the chemicals tested in the bioassays associated with SARS-CoV-2 or those involved in the pathways for SARS-CoV-2. Importantly, the Taxonomy Summary page presents the whole-organism bioassays, which were performed against the whole taxon without a specific target gene or macromolecule, and their bioactivity data. For example, the following URL leads to the whole-organism bioactivity data for SARS-CoV-2:

- https://pubchem.ncbi.nlm.nih.gov/taxonomy/2697049#section=Whole-Organism-Bioactivities

In addition to the NCBI Taxonomy ID, the common name or scientific name of an organism can also be used to access its Taxonomy page. For instance, the following URL examples lead to the Summary page of *Oryctolagus cuniculus* (rabbit; Taxonomy ID 9986):

- https://pubchem.ncbi.nlm.nih.gov/taxonomy/Oryctolagus+cuniculus
- https://pubchem.ncbi.nlm.nih.gov/taxonomy/rabbit
- https://pubchem.ncbi.nlm.nih.gov/taxonomy/9986

Programmatic access

As explained in our previous papers,[7,8,10] PubChem provides multiple programmatic access routes. Among them is PUG-View,[10] which is a Representational State Transfer (REST)-style web service interface specialized for accessing annotation data contained in PubChem. PUG-View is also used as a backend service to provide the annotation content displayed on the Summary pages for PubChem records. The annotation data for a given record can be accessed through the PUG-View request URL containing the corresponding record identifier, as shown in the following examples:

- Human CDK4 gene (Gene ID 1019)
  https://pubchem.ncbi.nlm.nih.gov/rest/pug_view/data/gene/1019/JSON
- Human Coagulation Factor X (protein accession P00742)
  https://pubchem.ncbi.nlm.nih.gov/rest/pug_view/data/protein/P00742/JSON
- Glycolysis (Pathway accession Reactome:R-HSA-70171)
  https://pubchem.ncbi.nlm.nih.gov/rest/pug_view/data/pathway/Reactome:R-HSA-70171/JSON
- SARS-CoV-2 (Taxonomy ID 2697049)
  https://pubchem.ncbi.nlm.nih.gov/rest/pug_view/data/taxonomy/2697049/JSON

Programmatic access to these data through PUG-View allows users to automate repetitive tasks or build computational pipelines for further analysis. It is noteworthy that there are programmatic access request volume limits, which are dynamically adjusted based on web traffic to PubChem, as explained in the following document:

- https://pubchemdocs.ncbi.nlm.nih.gov/dynamic-request-throttling

Therefore, users should moderate the speed at which access requests are sent to PubChem, according to the usage policies described in the "Programmatic Access" help page, available at:

- https://pubchemdocs.ncbi.nlm.nih.gov/programmatic-access$_RequestVolumeLimitations

Violation of the usage policies may result in being temporarily blocked from accessing PubChem resources.

PubChemRDF

PubChemRDF (https://pubchemdocs.ncbi.nlm.nih.gov/rdf)[11] refers to machine-readable PubChem data formatted using the Resource Description Framework (RDF). In RDF, knowledge is broken down into machine-readable discrete pieces, called

"triples". Each triple is organized as a trio of "subject-predicate-object", where the predicate defines the relationship between the subject and object. For example, in the phrase "asbestos may cause lung cancer", the subject is "asbestos", the predicate is "may cause", and the object is "lung cancer". In essence, RDF represents knowledge into a labeled, directed graph, where the nodes represent the subject and object and the edge between them corresponds to the predicate.

PubChemRDF contains information on various entities contained in PubChem (for example: chemicals, genes, proteins, pathways, organisms, patents, and scientific articles) and their relationship. The data in the Protein, Gene, Pathway, and Taxonomy collections are added to PubChemRDF and the corresponding subdomains are created. Currently, the protein, gene, pathway, and taxonomy subdomains contain 1.9 million, 5.8 million, 3.0 million, and 357 thousand triples, respectively, as of November 23, 2021. More detailed statistics on RDF data are available on the RDF statistics page (https://pubchemdocs.ncbi.nlm.nih.gov/rdf-statistics). The following are examples of the RDF formatted data for a gene, protein, pathway, and taxon:

- https://rdf.ncbi.nlm.nih.gov/pubchem/gene/GID1019.html
- https://rdf.ncbi.nlm.nih.gov/pubchem/pathway/PWID1294790.html
- https://rdf.ncbi.nlm.nih.gov/pubchem/taxonomy/TAXID2697049.html

Note that the above example URL for a pathway contains a pathway identifier (PWID1294790), which corresponds to the "glycolysis and gluconeogenesis" pathway from INOH (ID: MI0035772). This identifier mapping information can be found at the FTP site (https://ftp.ncbi.nlm.nih.gov/pubchem/Target/pwid2pwacc.gz). With PubChemRDF, users can readily integrate PubChem data with their in-house RDF data or those from other resources in different domains. The PubChemRDF data are available at the PubChem FTP site (https://ftp.ncbi.nlm.nih.gov/pubchem/RDF/). The RDF-formatted data for each subdomain are stored in its own subdirectory. This allows users to download only the desired type of information, without downloading all PubChemRDF data. The downloaded data can be imported into a triplestore (*e.g.*, Apache Jena TDB and OpenLink Virtuoso) and queried using a SPARQL query interface. Alternatively, the RDF data can be loaded into RDF-aware graph database (*e.g.*, Neo4j) and queried using graph traversal algorithms.

Discussion

The Summary page for a given record in a PubChem data collection displays all available information in PubChem for that record. This includes various biological entities contained in other data collections. These entities are linked to the corresponding PubChem pages, providing quick access to more detailed information on them. For example, as shown in Figure 1, the following URLs direct to the Chemicals, Proteins, Genes sections of the Summary page for the Glycolysis and Gluconeogenesis pathway from INOH:

- https://pubchem.ncbi.nlm.nih.gov/pathway/INOH:MI0035772#section=Chemicals
- https://pubchem.ncbi.nlm.nih.gov/pathway/INOH:MI0035772#section=Proteins
- https://pubchem.ncbi.nlm.nih.gov/pathway/INOH:MI0035772#section=Genes

These sections list the chemicals, proteins, and genes involved in the pathway, along with the links to their corresponding Summary pages. Conversely, the Summary page for this pathway can be accessed from the Summary pages of other records related to it, as in the following examples:

- Pathways section of the Compound Summary for CID 439191: https://pubchem.ncbi.nlm.nih.gov/compound/439191#section=Pathways
- Pathways section of the Protein Summary for accession number O00757: https://pubchem.ncbi.nlm.nih.gov/protein/O00757#section=Pathways
- Pathways section of the Gene Summary for Gene ID 226: https://pubchem.ncbi.nlm.nih.gov/gene/226#section=Pathways

In addition, the Summary for a taxon presents interactions among chemicals, genes, and proteins in the Interaction section, as shown in the following example:

- https://pubchem.ncbi.nlm.nih.gov/pathway/INOH:MI0035772#section=Interactions

Note that these URLs end with the string "#section=" followed by the section name. As mentioned previously, each section of the Summary page of a PubChem record has a unique URL, which can be used to bookmark that section for quick access later.

Besides, the annotation data presented on the Summary page come with their provenance information (*e.g.*, on where the data originate from), along with a link to the original data source, where the user can get additional information about the PubChem record of interest. All information found on the Summary page can be downloaded. Often the downloaded data contains

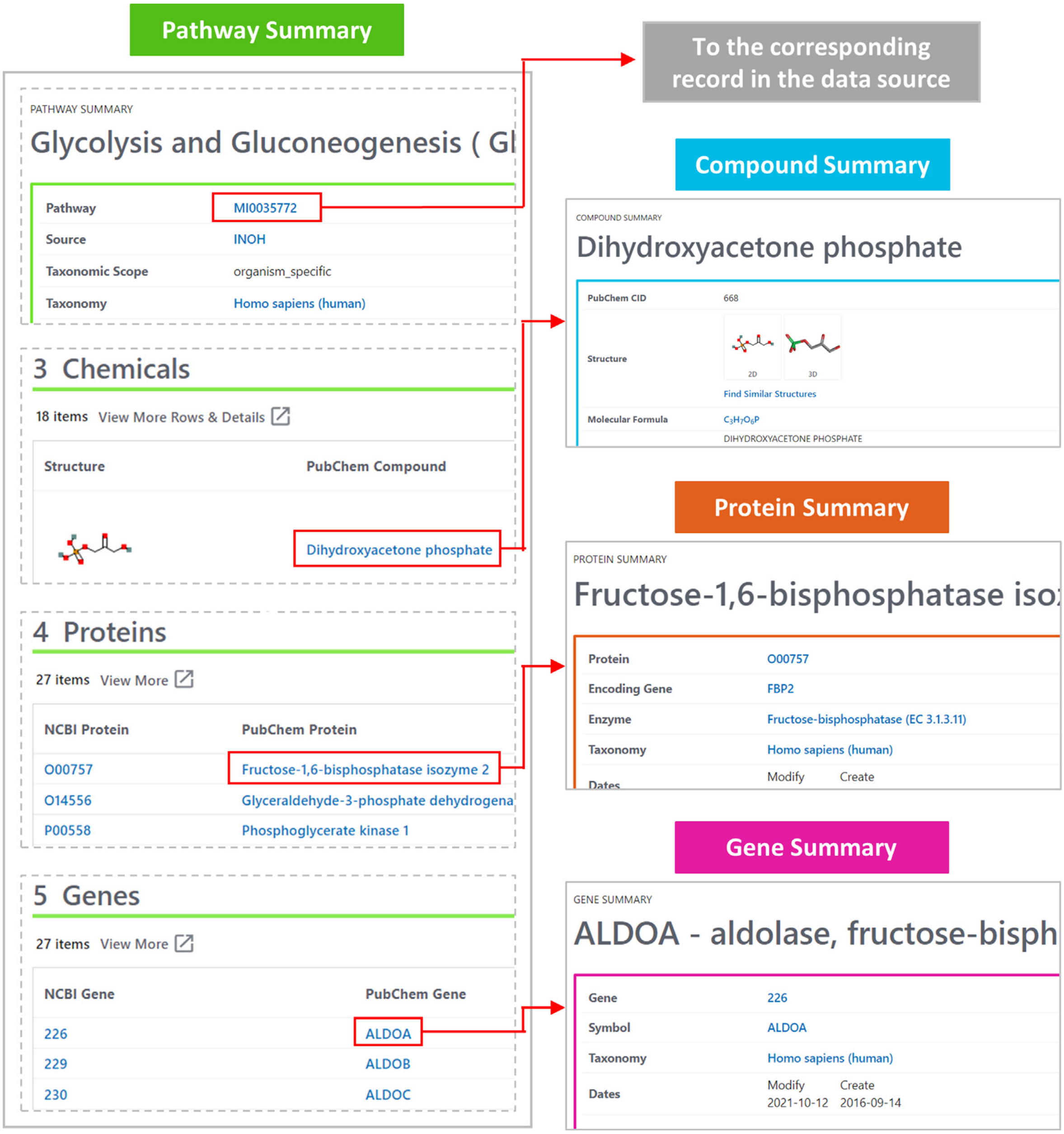

Figure 1. Getting information on entities involved in the "Glycolysis and Gluconeogenesis" pathway from its Pathway Summary page (https://pubchem.ncbi.nlm.nih.gov/pathway/INOH:MI0035772). The Summary page shows lists of chemicals, proteins, and genes associated with the pathway. Clicking the items on the lists leads to their Compound, Protein, or Gene Summary pages, which provide more detailed information on them. The Pathway Summary page has a link to the corresponding record in the original data source, which helps users to get additional information on the pathway.

more information than can be readily shown on a Summary page. Each (sub)section of the page has a link to a fullscreen mode, which shows most, if not all, of the downloadable data, as shown in this example:

- https://pubchem.ncbi.nlm.nih.gov/gene/226#section=Pathways&fullscreen=true

While individual records in PubChem's data collections can be accessed via direct URLs as described above, they are also accessible through the search interface available on the PubChem home page. As an example, Figure 2 shows how to search the Pathway collection for the glycolysis pathway from INOH. When a text query (e.g., "glycolysis") is provided in the search box (step ① in Figure 2), all data collections are searched simultaneously, and hits are presented together on the search result page. Clicking the "Pathway" tab on this page (step ②) shows hits from the Pathway collection. The user can refine the

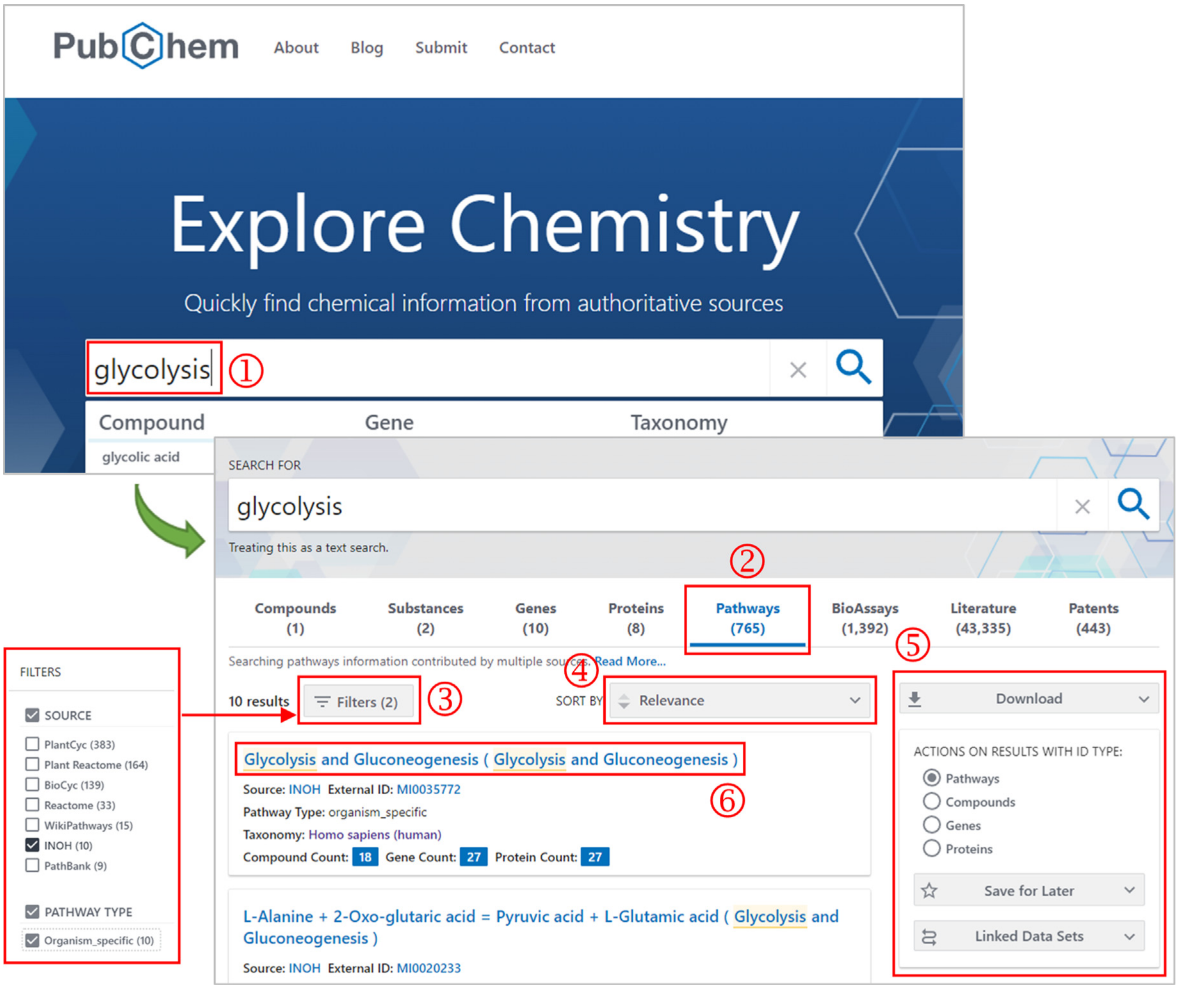

Figure 2. Searching PubChem using a text query, with “glycolysis” as an example. When a text query is provided in the search box (step ①), all data collections within PubChem are searched simultaneously and matching records found in each are returned together. Clicking the “Pathways” tab (step ②) shows the hits from the Pathway collection. The hit list can be refined or sorted by selected attributes (steps ③and ④). The additional controls on the right column allow users to download the hit list, save it for later use, or get other records associated with the hits (step ⑤). Clicking one of the pathway records directs to its Summary page, which provides comprehensive information on the record (step ⑥).

search results by filtering them by certain attributes (such as the data source or whether the pathway is conserved or organism-specific) (step ③) or sort the results by the criterion selected from the dropdown menu (step ④). Additional controls are available for downloading the hit records, saving them for later use in the browser, and getting other records related to them (step ⑤). Clicking one of the hit pathways directs the user to the corresponding Pathway Summary page (step ⑥). More detailed information on how to explore PubChem data using its web interfaces is given in our tutorial-style paper.[3]

The information contained in the Protein, Gene, Pathway, and Taxonomy collections provides a context to PubChem’s bioactivity data, helping users to analyze and interpret them. However, because bioactivity data for a given target in these collections are from multiple assays provided by different sources, they are very heterogeneous in the nature of tested compounds, experimental techniques and conditions, measurement accuracy, and so on. Therefore, care should be taken when working with these data to ensure proper interpretation of the results. Besides, it is important to keep in mind that a single gene may be associated with multiple protein sequences. For example, the human EGFR gene is associated with three protein sequences that are biological targets within PubChem assays (*i.e.*, protein accessions: P00533, CAA25240, and ADZ75461), as indicated in the “Protein Targets” section of its Gene Summary page available at:

- https://pubchem.ncbi.nlm.nih.gov/gene/1956#section=Protein-Targets

As a result, the bioactivity data for the EGFR gene, presented on its Summary page, are the aggregation of those for the three protein sequences, which can be accessed on the corresponding Protein Summary pages:

- https://pubchem.ncbi.nlm.nih.gov/protein/P00533#section=Tested-Compounds
- https://pubchem.ncbi.nlm.nih.gov/protein/CAA25240#section=Tested-Compounds
- https://pubchem.ncbi.nlm.nih.gov/protein/ADZ75461#section=Tested-Compounds

Note that a ligand may or may not have the same bioactivity against these three proteins, although they are associated with the same gene. This may introduce some undesired effects when the aggregated bioactivity data for the gene are used to perform quantitive structure–activity relationship analysis or to develop bioactivity prediction models.

Conclusions

Protein, Gene, Pathway, and Taxonomy collections are available within the PubChem resource. Records in these collections contain information on chemicals related to a given biological target (*i.e.*, protein, gene, pathway, or organism). A wide range of annotations for the targets are collected from various sources and integrated into the four data collections. In essence, these collections help provide users with a biological target-centric view of PubChem data.

All four collections are searchable from the PubChem home page (https://pubchem.ncbi.nlm.nih.gov/). In addition, each record in these collections has information on other related records in PubChem (e.g., compounds, substances, and bioassays) and a record in one data collection can readily be accessed from the Summary page of an associated record in the same or different data collection. This helps users to quickly get the desired information.

Importantly, programmatic access to the four data collections through PUG-View enables construction of a computational pipeline that exploits them. Moreover, the data in these collections are added to PubChemRDF and made available through the PubChem FTP site. These RDF-formatted data can be downloaded and readily integrated with in-house data or data from other resources using semantic web technologies.

Materials and Methods

The PubChem Gene and Protein collections contain genes and proteins, respectively, that are tested against bioassays archived in PubChem or that appear in any pathway records in the PubChem Pathway collection. The bioactivity data of chemicals against genes and proteins are from PubChem BioAssay as well as other curated sources including, DrugBank,[14] ChEMBL,[6] and the IUPHAR/BPS Guide to PHARMACOLOGY.[15] Additional information on genes and proteins is collected from several NLM/NCBI databases[4] as well as other data sources, including the Comparative Toxicogenomics Database (CTD),[28] Drug Gene Interaction Database (DGIdb),[29] Rhea,[30] RCSB Protein Data Bank (PDB),[31] UniProt,[32] Pfam,[33] Online Mendelian Inheritance in Man (OMIM),[34] Kyoto Encyclopedia of Genes and Genomes (KEGG),[35] The GlyCosmos Portal,[36] Gene Ontology,[37] and the Protein Ontology.[38]

The PubChem Pathway collection was constructed with records from multiple pathway information resources, including Reactome,[16] Plant Reactome,[17] BioCyc,[18] PlantCyc,[19] WikiPathways,[20] PathBank,[21] LIPID MAPS,[22] PharmGKB,[23] Pathway Interaction Database,[24] INOH[25] and the COVID-19 Disease Map.[26] It is noteworthy that, while different data sources may have records for the same pathway, PubChem does not merge or combine them into a single record. As a result, for a given pathway, multiple records originating from different sources can exist in the PubChem Pathway collection.

The PubChem Taxonomy collection contains taxa that are associated with macromolecule targets for bioassays archived in PubChem or pathways contained in PubChem Pathway. Additional information on taxa is collected from multiple sources, including the Catalogue of Life (https://www.catalogueoflife.org/), Integrated Taxonomic Information System (ITIS) (https://www.itis.gov/), NCI Thesaurus,[39] UniProt,[32] and the GlyCosmos Portal.[36]

The Protein, Gene, Pathway, and Taxonomy data collections are made searchable from the PubChem homepage (Figure 2). The search interface accepts various types of keyword queries (including: chemical names, gene names and symbols, pathway names, organism names, and record identifiers of the original data sources). Search results can be refined by filtering based on select attributes (*e.g.*, data source). In addition, the annotation data contained in these collections are also made programmatically accessible through PUG-View,[10] which is a REST-style interface to PubChem. More detailed information on PUG-View is available in our previous paper[10] as well as the PubChem Help documentation (https://pubchemdocs.ncbi.nlm.nih.gov/pug-view).

The data in the Protein, Gene, Pathway, and Taxonomy collections are formatted using RDF and added to PubChemRDF.[11] To define domain-specific knowledge related to the data contained in these collections, a set of standard ontologies were used, including the Simple Knowledge Organization System (SKOS) (https://www.w3.org/2004/02/skos/), Dublin Core Metadata Initiative (DCMI) Metadata Terms (https://dublincore.org/specifica-

tions/dublin-core/dcmi-terms/), BioPAX,[40] Citation Typing Ontology (CiTO),[41] and the Semantic-science Integrated Ontology (SIO).[42] New PubChemRDF subdomains (*i.e.*, protein, gene, pathway, and taxonomy) were created and their respective RDF data were stored in gzip-compressed turtle format on the PubChem FTP site (https://ftp.ncbi.nlm.nih.gov/pubchem/RDF/). Additional details about the construction of PubChemRDF can be found in our previous paper[11] and the PubChemRDF Help page (https://pubchemdocs.ncbi.nlm.nih.gov/rdf).

CRediT authorship contribution statement

Sunghwan Kim: Writing – original draft, Validation. **Tiejun Cheng:** Data curation, Software, Resources. **Siqian He:** Software, Resources. **Paul A. Thiessen:** Software. **Qingliang Li:** Data curation, Software. **Asta Gindulyte:** Software. **Evan E. Bolton:** Conceptualization, Project administration, Supervision.

DATA AVAILABILITY

All PubChem data, tools, and services are provided to the public free of charge.

Acknowledgments

This work was supported by the National Center for Biotechnology Information of the National Library of Medicine (NLM), National Institutes of Health.

Declaration of interests

The authors declare that they have no known competing financial interests or personal relationships that could have appeared to influence the work reported in this paper.

Received 24 November 2021;
Accepted 22 February 2022;
Available online 25 February 2022

Keywords:
public chemical database;
cheminformatics;
bioinformatics;
bioactivity;
drug discovery

† These authors contributed equally to this work.

Abbreviations:
CID, Compound identifier; SID, Substance identifier; AID, Assay identifier; RDF, resource description framework; REST, representational state transfer; FTP, file transfer protocol; URL, uniform resource locator; SPARQL, SPARQL Protocol and RDF Query Language

References

1. Kim, S., Chen, J., Cheng, T., Gindulyte, A., He, J., He, S., Li, Q., Shoemaker, B.A., et al., (2021). PubChem in 2021: new data content and improved web interfaces. *Nucleic Acids Res.* **49**, D1388–D1395.
2. Kim, S., (2016). Getting the most out of PubChem for virtual screening. *Expert Opin. Drug Discov.* **11**, 843–855.
3. Kim, S., (2021). Exploring Chemical Information in PubChem. *Curr. Protoc.* **1**, e217
4. Sayers, E.W., Beck, J., Bolton, E.E., Bourexis, D., Brister, J.R., Canese, K., Comeau, D.C., Funk, K., et al., (2021). Database resources of the National Center for Biotechnology Information. *Nucleic Acids Res.* **49**, D10–D17.
5. Kim, S., Thiessen, P.A., Cheng, T., Yu, B., Shoemaker, B. A., Wang, J., Bolton, E.E., Wang, Y., et al., (2016). Literature information in PubChem: associations between PubChem records and scientific articles. *J. Cheminform.* **8**, 32. https://doi.org/10.1186/s13321-016-0142-6.
6. Mendez, D., Gaulton, A., Bento, A.P., Chambers, J., De Veij, M., Felix, E., Magarinos, M.P., Mosquera, J.F., et al., (2019). ChEMBL: towards direct deposition of bioassay data. *Nucleic Acids Res.* **47**, D930–D940.
7. Kim, S., Thiessen, P.A., Cheng, T.J., Yu, B., Bolton, E.E., (2018). An update on PUG-REST: RESTful interface for programmatic access to PubChem. *Nucleic Acids Res.* **46**, W563–W570.
8. Kim, S., Thiessen, P.A., Bolton, E.E., Bryant, S.H., (2015). PUG-SOAP and PUG-REST: web services for programmatic access to chemical information in PubChem. *Nucleic Acids Res.* **43**, W605–W611.
9. Kim, S., Thiessen, P.A., Bolton, E.E., (2019). Programmatic Retrieval of Small Molecule Information from PubChem Using PUG-REST. *In: Methods in Pharmacology and Toxicology*. Humana Press, Totowa, NJ. https://doi.org/10.1007/7653_2018_30.
10. Kim, S., Thiessen, P.A., Cheng, T., Zhang, J., Gindulyte, A., Bolton, E.E., (2019). PUG-View: programmatic access to chemical annotations integrated in PubChem. *J. Cheminform.* **11**, 56. https://doi.org/10.1186/s13321-019-0375-2.
11. Fu, G., Batchelor, C., Dumontier, M., Hastings, J., Willighagen, E., Bolton, E., (2015). PubChemRDF: towards the semantic annotation of PubChem compound and substance databases. *J. Cheminform.* **7**, 34.
12. Kim, S., Thiessen, P.A., Bolton, E.E., Chen, J., Fu, G., Gindulyte, A., Han, L.Y., He, J.E., et al., (2016). PubChem Substance and Compound databases. *Nucleic Acids Res.* **44**, D1202–D1213.
13. Hähnke, V.D., Kim, S., Bolton, E.E., (2018). PubChem chemical structure standardization. *J. Cheminform.* **10**, 36.
14. Wishart, D.S., Feunang, Y.D., Guo, A.C., Lo, E.J., Marcu, A., Grant, J.R., Sajed, T., Johnson, D., et al., (2018). DrugBank 5.0: a major update to the DrugBank database for 2018. *Nucleic Acids Res.* **46**, D1074–D1082.
15. Harding, S.D., Armstrong, J.F., Faccenda, E., Southan, C., Alexander, S.P.H., Davenport, A.P., Pawson, A.J., Spedding, M., et al., (2022). The IUPHAR/BPS guide to PHARMACOLOGY in 2022: curating pharmacology for

COVID-19, malaria and antibacterials. *Nucleic Acids Res.* **50**, D1282–D1294.
16. Jassal, B., Matthews, L., Viteri, G., Gong, C.Q., Lorente, P., Fabregat, A., Sidiropoulos, K., Cook, J., et al., (2020). The reactome pathway knowledgebase. *Nucleic Acids Res.* **48**, D498–D503.
17. Naithani, S., Gupta, P., Preece, J., D'Eustachio, P., Elser, J.L., Garg, P., Dikeman, D.A., Kiff, J., et al., (2020). Plant Reactome: a knowledgebase and resource for comparative pathway analysis. *Nucleic Acids Res.* **48**, D1093–D1103.
18. Karp, P.D., Billington, R., Caspi, R., Fulcher, C.A., Latendresse, M., Kothari, A., Keseler, I.M., Krummenacker, M., et al., (2019). The BioCyc collection of microbial genomes and metabolic pathways. *Brief. Bioinform.* **20**, 1085–1093.
19. Schläpfer, P., Zhang, P.F., Wang, C.A., Kim, T., Banf, M., Chae, L., Dreher, K., Chavali, A.K., et al., (2017). Genome-Wide Prediction of Metabolic Enzymes, Pathways, and Gene Clusters in Plants. *Plant Physiol.* **173**, 2041–2059.
20. Slenter, D.N., Kutmon, M., Hanspers, K., Riutta, A., Windsor, J., Nunes, N., Melius, J., Cirillo, E., et al., (2018). WikiPathways: a multifaceted pathway database bridging metabolomics to other omics research. *Nucleic Acids Res.* **46**, D661–D667.
21. Wishart, D.S., Li, C., Marcu, A., Badran, H., Pon, A., Budinski, Z., Patron, J., Lipton, D., et al., (2020). PathBank: a comprehensive pathway database for model organisms. *Nucleic Acids Res.* **48**, D470–D478.
22. O'Donnell, V.B., Dennis, E.A., Wakelam, M.J.O., Subramaniam, S., (2019). LIPID MAPS: Serving the next generation of lipid researchers with tools, resources, data, and training. *Sci. Signal.* **12**, eaaw2964.
23. Whirl-Carrillo, M., McDonagh, E.M., Hebert, J.M., Gong, L., Sangkuhl, K., Thorn, C.F., Altman, R.B., Klein, T.E., (2012). Pharmacogenomics Knowledge for Personalized Medicine. *Clin. Pharmacol. Ther.* **92**, 414–417.
24. Schaefer, C.F., Anthony, K., Krupa, S., Buchoff, J., Day, M., Hannay, T., Buetow, K.H., (2009). PID: the Pathway Interaction Database. *Nucleic Acids Res.* **37**, D674–D679.
25. Yamamoto, S., Sakai, N., Nakamura, H., Fukagawa, H., Fukuda, K., Takagi, T., (2011). INOH: ontology-based highly structured database of signal transduction pathways. *Database* **2011**, bar052.
26. Ostaszewski, M., Mazein, A., Gillespie, M.E., Kuperstein, I., Niarakis, A., Hermjakob, H., Pico, A.R., Willighagen, E. L., et al., (2020). COVID-19 Disease Map, building a computational repository of SARS-CoV-2 virus-host interaction mechanisms. *Sci. Data* **7**, 136.
27. Geer, L.Y., Marchler-Bauer, A., Geer, R.C., Han, L.Y., He, J., He, S.Q., Liu, C.L., Shi, W.Y., et al., (2010). The NCBI BioSystems database. *Nucleic Acids Res.* **38**, D492–D496.
28. Davis, A.P., Grondin, C.J., Johnson, R.J., Sciaky, D., Wiegers, J., Wiegers, T.C., Mattingly, C.J., (2021). Comparative Toxicogenomics Database (CTD): update 2021. *Nucleic Acids Res.* **49**, D1138–D1143.
29. Freshour, S.L., Kiwala, S., Cotto, K.C., Coffman, A.C., McMichael, J.F., Song, J.J., Griffith, M., Griffith, O.L., et al., (2021). Integration of the Drug-Gene Interaction Database (DGIdb 4.0) with open crowdsource efforts. *Nucleic Acids Res.* **49**, D1144–D1151.
30. Bansal, P., Morgat, A., Axelsen, K.B., Muthukrishnan, V., Coudert, E., Aimo, L., Hyka-Nouspikel, N., Gasteiger, E., et al., (2022). Rhea, the reaction knowledgebase in 2022. *Nucleic Acids Res.* **50**, D693–D700. https://doi.org/10.1093/nar/gkab1016.
31. Burley, S.K., Berman, H.M., Bhikadiya, C., Bi, C.X., Chen, L., Di Costanzo, L., Christie, C., Dalenberg, K., et al., (2019). RCSB Protein Data Bank: biological macromolecular structures enabling research and education in fundamental biology, biomedicine, biotechnology and energy. *Nucleic Acids Res.* **47**, D464–D474.
32. Bateman, A., Martin, M.J., Orchard, S., Magrane, M., Alpi, E., Bely, B., Bingley, M., Britto, R., et al., (2019). UniProt: a worldwide hub of protein knowledge. *Nucleic Acids Res.* **47**, D506–D515.
33. Mistry, J., Chuguransky, S., Williams, L., Qureshi, M., Salazar, G.A., Sonnhammer, E.L.L., Tosatto, S.C.E., Paladin, L., et al., (2021). Pfam: The protein families database in 2021. *Nucleic Acids Res.* **49**, D412–D419.
34. Amberger, J.S., Bocchini, C.A., Scott, A.F., Hamosh, A., (2019). OMIM.org: leveraging knowledge across phenotype–gene relationships. *Nucleic Acids Res.* **47**, D1038–D1043.
35. Kanehisa, M., Furumichi, M., Sato, Y., Ishiguro-Watanabe, M., Tanabe, M., (2021). KEGG: integrating viruses and cellular organisms. *Nucleic Acids Res.* **49**, D545–D551.
36. Yamada, I., Shiota, M., Shinmachi, D., Ono, T., Tsuchiya, S., Hosoda, M., Fujita, A., Aoki, N.P., et al., (2020). The GlyCosmos Portal: a unified and comprehensive web resource for the glycosciences. *Nature Methods* **17**, 649–650.
37. The Gene Ontology Consortium, (2020). The Gene Ontology resource: enriching a GOld mine. *Nucleic Acids Res.* **49**, D325–D334.
38. Natale, D.A., Arighi, C.N., Blake, J.A., Bona, J., Chen, C., Chen, S.-C., Christie, K.R., Cowart, J., et al., (2017). Protein Ontology (PRO): enhancing and scaling up the representation of protein entities. *Nucleic Acids Res.* **45**, D339–D346.
39. Sioutos, N., de Coronado, S., Haber, M.W., Hartel, F.W., Shaiu, W.L., Wright, L.W., (2007). NCI Thesaurus: A semantic model integrating cancer-related clinical and molecular information. *J. Biomed. Inform.* **40**, 30–43.
40. Demir, E., Cary, M.P., Paley, S., Fukuda, K., Lemer, C., Vastrik, I., Wu, G., D'Eustachio, P., et al., (2010). The BioPAX community standard for pathway data sharing. *Nature Biotechnol.* **28**, 935–942.
41. Peroni, S., Shotton, D., (2012). FaBiO and CiTO: Ontologies for describing bibliographic resources and citations. *J. Web Semant.* **17**, 33–43.
42. Dumontier, M., Baker, C.J.O., Baran, J., Callahan, A., Chepelev, L., Cruz-Toledo, J., Del Rio, N.R., Duck, G., et al., (2014). The Semanticscience Integrated Ontology (SIO) for biomedical research and knowledge discovery. *J. Biomed. Semant.* **5**, 14.
