# Supplementary material for: PDBx/mmCIF Ecosystem: Foundational Semantic Tools for Structural Biology
Source: J Mol Biol. Author manuscript; Available in PMC 2023 Jun 26. (PMC10292674; doi:10.1016/j.jmb.2022.167599)
Supplement: Article [file NIHMS1907597-supplement-Article.zip › Bacteria-guru--Comparative-Transcriptomics-and-Co-Expr_2022_Journal-of-Molec.pdf]

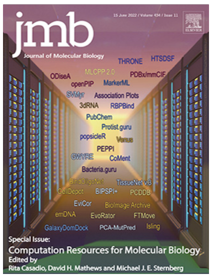

Bacteria.guru: Comparative Transcriptomics and Co-Expression Database for Bacterial Pathogens

Peng Ken Lim, Emilia Emmanuelle Davey, Sean Wee, Wei Song Seetoh, Jong Ching Goh, Xinghai Zheng, Sean Kia Ann Phang, Eugene Sheng Kai Seah, Janice Wan Zhen Ng, Xavier Jia Hui Wee, Aloysius Jun Hui Quek, Jordan JingHeng Lim, Edbert Edric Rodrigues, Heesoo Lee, Chin Yong Lim, Wei Zhi Tan, Yuet Ruh Dan, Bronson Lee, Samuel En Le Chee, Zachary Ze En Lim, Jia Sheng Guan, Ivan Jia Le Tan, Trinidad Jeremiah Arong and Marek Mutwil *

School of Biological Sciences, *Nanyang Technological University, 60 Nanyang Drive, Singapore 637551, Singapore*

Correspondence to Marek Mutwil: *mutwil@ntu.edu.sg (M. Mutwil) @labmutwil (M. Mutwil)*
https://doi.org/10.1016/j.jmb.2021.167380
Edited by David Mathews

Abstract

Summary: While bacteria can be beneficial to our health, their deadly pathogenic potential has been an ever-present concern exacerbated by the emergence of drug-resistant strains. As such, there is a pressing urgency for an enhanced understanding of their gene function and regulation, which could mediate the development of novel antimicrobials. Transcriptomic analyses have been established as insightful and indispensable to the functional characterization of genes and identification of new biological pathways, but in the context of bacterial studies, they remain limited to species-specific datasets. To address this, we integrated the genomic and transcriptomic data of the 17 most notorious and researched bacterial pathogens, creating bacteria.guru, an interactive database that can identify, visualize, and compare gene expression profiles, coexpression networks, functionally enriched clusters, and gene families across species. Through illustrating antibiotic resistance mechanisms in *P. aeruginosa*, we demonstrate that bacteria.guru could potentially aid in discovering multi-faceted antibiotic targets and, overall, facilitate future bacterial research.
Availability: The database and coexpression networks are freely available from https://bacteria.guru/. Sample annotations can be found in the supplemental data.

© 2021 Elsevier Ltd. All rights reserved.

Introduction

Bacteria are ubiquitous unicellular organisms that constitute a diverse kingdom. Despite their fundamental role in the biosphere, the rapid evolution and acute virulence of pathogenic varieties constitute an urgent need for novel antimicrobials,[5] especially considering the negligent use of such medications. The combination of these factors precipitated an epidemic of antibiotic resistance; for example, following the introduction of β-lactams, which inhibit cell wall peptidoglycan synthesis, many bacterial pathogens increased the production of β-lactamases, rendering these drugs ineffective. As bacteria continue to evolve such novel resistance mechanisms, our inability to combat them reaches an alarming state.

Several methodologies can be used to study genes essential for virulence and antibiotic resistance, such as sequence similarity, gene

0022-2836/© 2021 Elsevier Ltd. All rights reserved.

expression, and coexpression network analyses.[40,12] While predicting gene function from gene sequence alone often results in an incorrect or, at most, partial prediction,[7,28] coexpression networks serve as the state-of-the-art mechanism through which genomicists obtain insight into the evolution and interspecific conservation of gene modules,[31] gene function, gene regulation, and subcellular localization of gene products.[19] In these networks, which are based on the observation that genes that exhibit similar expression profiles across different growth conditions and genotypes are functionally related,[19] nodes correspond to genes, and edges represent their significant coexpression relationships.[29] Their utility has exponentiated our understanding of gene function in multiple kingdoms of life, as the broad trends they detect cannot be revealed by more specific assays.[17] The exploitation of these transcriptomic analyses to elucidate the human genome has proved instrumental in understanding and treating endogenous disease,[17,41] but has not been utilized to the same effect in exogenous diseases caused by bacteria and other pathogens. As case in point, only one bacterial coexpression web-base, COLOMBOS,[18] http://www.colombos.net/, is available for use at present, whereas numerous such resources have been developed for alike quantities of eukaryotic organisms.[11,16,19,21,22,27,37,39]

Since the advent of next-generation sequencing, bacterial gene expression data have been generated at an unprecedented rate via RNA-sequencing (RNA-seq) and made publicly available.[15] The ongoing and worsening antimicrobial resistance crisis coinciding with the increase in published transcriptomic and genomic data for bacteria[32,4,14] not only affords the means for but compels the creation of a platform enabling expansive exploration of inter-and intraspecific gene expression. To that end, we present bacteria.guru, a database built from RNA-seq and genomic data of the 17 most notorious bacterial pathogens responsible for nosocomial infections, of which 11 are not featured on any existing coexpression database. In addition to identifying and analyzing coexpression networks and conserved gene modules, bacteria.guru enables the within-and across-species analysis of gene expression profiles, gene families, ontology terms, and possible gene-cluster redundancy.

Implementation

Bacteria.guru is based on the CoNekT framework.[25] Coding sequences (CDSs) (Table S1) and gene expression data of 17 bacterial species (Table 1) were obtained from a recent crowd-sourced analysis of bacterial RNA sequencing data,[9] where gene expression was quality-controlled (Table S2) and quantified via Kallisto pseudoalignment.[1] To identify orthogroups, CDSs were fed into OrthoFinder v2.312[6] using Diamond[2] with default settings, and the FastTree algorithm[24] was used to construct gene trees. To identify Pfam domains and Gene Ontology (GO) terms, we used the onboard conversion function of CoNekt[25] to generate PEP files from CDSs, and subjected them to Interproscan-5.51-85.0-5.44-79[13] analysis. For each species, Pearson Correlation Coefficients (PCCs) of gene pairs were calculated based on expression across experiments. Highest Reciprocal Rank (HRR)[20] was used to construct coexpression networks, and coexpression clusters were generated via Heuristic Cluster Chiseling Algorithm (HCCA),[20] with a cluster size of 100 genes. Experiment annotations (Table S2), including the specific bacterial strains and growth perturbations, were derived from SRA runtables downloaded from the NCBI (National Center for Biotechnology Information) database, and were used to construct the expression profiles. Gene symbols, locus tags, and uniprotIDs[38]; if any) of CDSs were obtained from genome assembly GFF files downloaded from Genbank.[33] Makeblastdb v2.9.0+[3] was used to construct the protein and nucleotide blast databases for the BLAST search functionality featured on bacteria.guru (https://bacteria.sbs.ntu.edu.sg/blast/).

To demonstrate how our database can be used to generate novel insights, we use *Pseudomonas aeruginosa* (*P. aeruginosa*) as a case study. Among the most common ICU-acquired infections in critically ill patients,[23] the pathogen is not only multidrug-resistant but was also recently discovered to have adaptive resistance capabilities.[36] To better comprehend antibiotic resistance in *P. aeruginosa*, 'antibiotic metabolic process' (GO:0016999) was queried using the 'Find enriched clusters' tool (https://bacteria.sbs.ntu.edu.sg/search/enriched/clusters), where cluster 12 (https://bacteria.sbs.ntu.edu.sg/cluster/view/467,Fig. 1(A)), containing 238 genes, was identified to be significantly enriched for the term (p-value < 0.05). Displayed in the gene cluster profile is how its expression in samples changes with varying treatments, genotypes, or growth phases (Fig. 1(B)). For example, we observed that the cluster has high expression in PA,cmr + genotype, while conversely, the strain D + 7bld has lowest expression (Fig. 1(B)). The experiment IDs that allow the identification of the relevant studies are found in Table S2. Further analysis of the cluster revealed gene members involved in several vital processes, including phenazine synthesis and quorum sensing (QS), alongside antibiotic metabolism (Fig. 1(A)), implicating these processes in *P. aeruginosa* resistance mechanisms. Production of phenazine has been proposed to alter antibiotic susceptibility[34] and affect swarming motility at early stages of biofilm formation,[26] whereas QS serves as an important regulatory mechanism for coordinating biofilm formation.[35] By exploring these interrelations, we could determine whether adaptive antibiotic resistance is

Table 1 17 bacterial species featured on Bacteria.guru and their statistics

Bacterial species	Number of genes	RNA-seq experiments
Campylobacter jejuni	1635	219
Clostridioides difficile	3769	182
Enterococcus faecalis	2579	81
Escherichia coli	4140	2494
Haemophilus influenzae	2098	189
Helicobacter pylori	1720	84
Klebsiella pneumoniae	5141	536
Listeria monocytogenes	2817	370
Mycobacterium tuberculosis	4018	4018
Mycoplasma pneumoniae	629	365
Neisseria gonorrhoeae	2159	102
Pseudomonas aeruginosa	6512	1372
Salmonella enterica	4554	611
Staphylococcus aureus	2638	1112
Streptococcus pneumoniae	2043	422
Streptococcus pyogenes	1659	1340
Vibrio cholerae	3648	167

achieved through the mechanism of biofilm aggregation. The presence of several proteins related to flagellum-hooking, fimbrial assembly, and cell-adhesion in the cluster, which are essential in biofilm formation,[8,30] supports this premise; in fact, Miller and colleagues observed biofilm-induction by aminoglycoside antibiotics in *P. aeruginosa*,[10] demonstrating how our database can be used to unravel potential antibiotic-induced epigenetic changes.

The 'Similar clusters' tool discloses conserved clusters in other bacterial species for further analysis. Through this feature, we identified and compared an analogous cluster in *Vibrio cholerae* (Jaccard index = 0.062) to that of number 12 in *P. aeruginosa* (Fig. 2(A)). For any selected gene, bacteria.guru will also identify similar coexpression neighborhoods, which are listed under the 'Expression context conservation' (ECC) table of the expression profile page. To illustrate this tool, Mycobacterial persistence regulator A from cluster 12 (ID: CRN75072, gene family: OG_02_0000001), a protein involved in stress resistance and persistent host infection,[42] was chosen for inquiry. Selecting the 'View ECC as graph' icon located in its corresponding ECC table disclosed seven other genes from the OG_02_0000001 family, belonging to three other species, whose coexpression neighborhood possesses similar gene family content to that of CRN75072 (Fig. 2(B)) (https://bacteria.sbs.ntu.edu.sg/ecc/graph/52806/11/2). A detailed comparison of the composition of each neighborhood with that of CRN75072 may be viewed by clicking 'View ECC pair as graph.' Analogous to the 'Similar clusters' tool, gene neighborhood comparison will disclose conserved gene families and Pfam domains, as specified by the color and shape of each node, which can pertain to the evolutionary history and perhaps utility of the gene modules.

In addition to comparative gene cluster and neighborhood analysis, our database provides ortholog information for individual genes. For example, clicking on the gene family of autoinducer SdiA (IDs: CRO08731; sdiA_1; gene family: OG_02_0001636) located on its gene page (Fig. 2(C)), followed by the corresponding phylogenetic tree link, disclosed orthologs found in three other bacterial species (Fig. 2(E)). Furthermore, the presence of four *sdiA* paralogs denotes a critical role of QS in *P. aeruginosa* antibiotic resistance. With the interplay of QS in formation and regulation of resistance mechanisms necessitating further study,[43] additional scrutiny of this biological process overlap could illuminate the nexus of microbial resistance.

Aside from the tools through which we illustrated our case study, the user may also identify genes significantly overexpressed under selected sample conditions, as well as compare expression profiles and generate heat maps or custom coexpression networks for any group of genes. Selected expression profiles of genes (each with aliases, if applicable) may be located via either the standard search bar or the 'Advanced search' feature (https://bacteria.sbs.ntu.edu.sg/search/advanced). While the former returns results upon entries of specific gene IDs, the latter may be used to identify any gene of interest using pertinent gene descriptions, gene family identifiers, GO terms, and Interpro domains. Altogether, bacteria.guru supplies gene pages (https://bacteria.sbs.ntu.edu.sg/sequence/view/2), each containing functional annotations, cDNA (https://bacteria.sbs.ntu.edu.sg/sequence/modal/coding/2) and protein sequences (https://bacteria.sbs.ntu.edu.sg/sequence/modal/protein/2), the assigned gene family (https://bacteria.sbs.ntu.edu.sg/family/view/19489) and corresponding phylogenetic tree (https://bacteria.sbs.ntu.edu.sg/tree/view/3667),

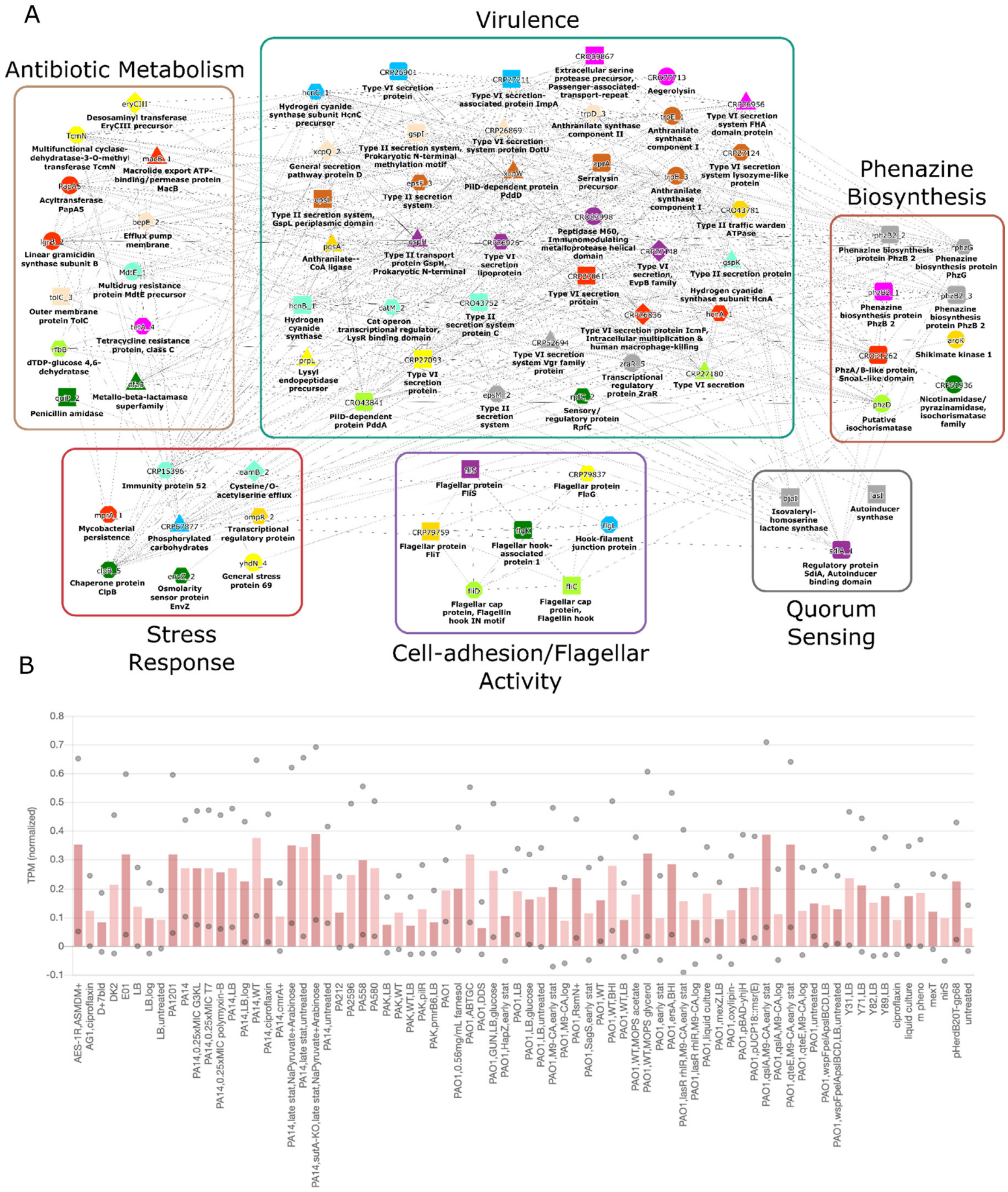

Fig. 1. Antibiotic metabolic process analysis in *Pseudomonas aeruginosa*. (A) Coexpression cluster 12 from *P. aeruginosa* enriched for GO:0016999 (Antibiotic Metabolic Process). Nodes, colored shapes, and gray edges represent genes, orthogroups, and co-expressed genes, respectively. For concision, co-expressed genes that are not involved in the processes discussed were collapsed as shown. (B) Average expression profile of cluster 12 for *P. aeruginosa.* The x-axis and y-axis represent different samples (capturing genotype and growth condition) and gene expression levels (in transcripts per million), respectively. Bars indicate the average expression, while points represent the maximum/minimum expression in a given experiment.

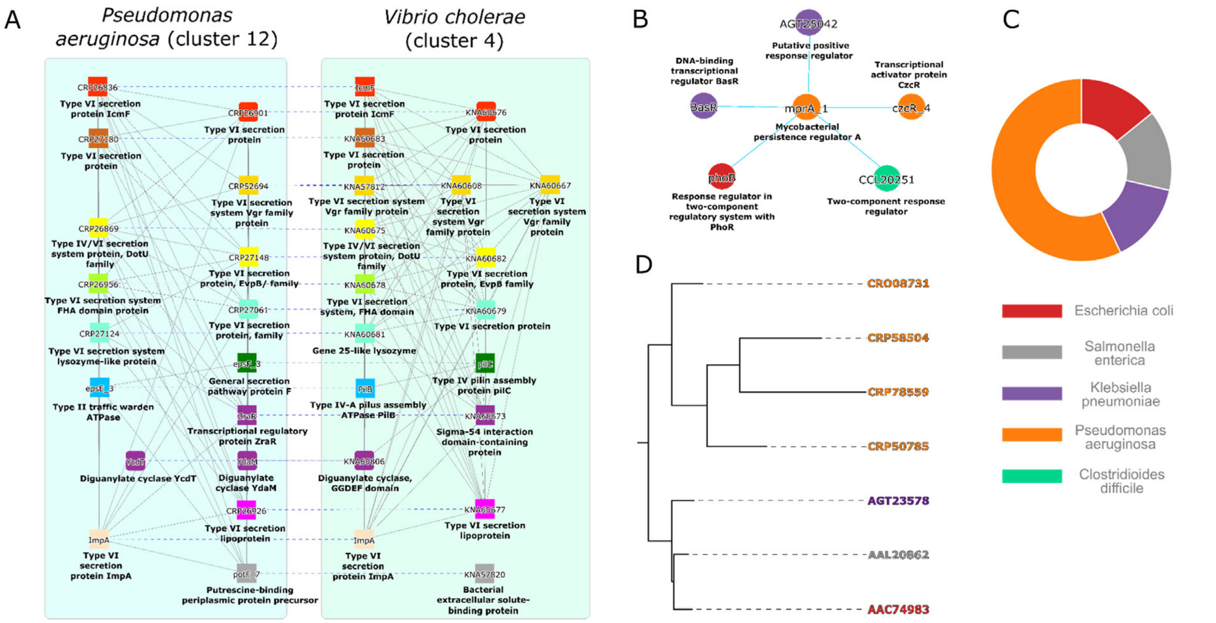

Fig. 2. Cross-species analyses using gene families (orthgroups). (A) Similar co-expression clusters in *P. aeruginosa* (cluster 12, blue box) and *Vibrio cholerae* (cluster 4, green box) were identified using gene family memberships to reveal conserved virulence-associated orthologs between the two. Dashed edges connect genes belonging to the same orthogroup. (B) Expression context conservation (ECC) network of gene CRN75072 (mprA_1). Gene co-expression neighborhoods are represented by nodes with blue edges linking conserved neighborhoods. Node colors specify the species origin of the neighborhood (see legend in figure (C)). (C) The donut chart indicates the proportion of genes belonging to *P. aeruginosa* (4 genes), *Escherichia coli* (1 gene), *Klebsiella pneumoniae* (1 gene), and *Salmonella enterica* (1 gene) in the OG_02_0001636 orthogroup. F) Gene tree of the OG_02_0001636 orthogroup (*P. aeruginosa* paralogs comprise the upper clade). The color coding between the donut charts and gene trees is conserved.

expression profiles (https://bacteria.sbs.ntu.edu.sg/profile/view/2), the co-expression neighborhood (https://bacteria.sbs.ntu.edu.sg/network/graph/2) and cluster (https://bacteria.sbs.ntu.edu.sg/cluster/graph/14), significantly similar neighborhoods (https://bacteria.sbs.ntu.edu.sg/ecc/graph/2/1/2), Gene Ontology information (https://bacteria.sbs.ntu.edu.sg/go/view/4800) and Pfam/Interpro domains (https://bacteria.sbs.ntu.edu.sg/interpro/view/19166) (https://bacteria.guru/features). Moreover, the database can be used to detect condition-specific genes (https://bacteria.sbs.ntu.edu.sg/search/specific/profiles), compare condition-specific expression between two species (https://bacteria.sbs.ntu.edu.sg/specificity_comparison/), and identify co-expression clusters enriched for a specific Gene Ontology process (https://bacteria.sbs.ntu.edu.sg/search/enriched/clusters).

Conclusion

The need to better comprehend antibiotic resistance drove the construction of bacteria.guru. The database offers inter-and intraspecific analyses of co-expressed gene networks and neighborhoods, expression profiles, gene families, phylograms, and ontology terms in 17 of the most notorious pathogens. Although we demonstrated how bacteria.guru can be used to study *P. aeruginosa* antibiotic metabolism, we envision that its function can be extended to explore any biological pathway and evolutionary relationship in these bacteria.

DECLARATION OF COMPETING INTEREST

The authors declare that they have no known competing financial interests or personal relationships that could have appeared to influence the work reported in this paper. We thank Nanyang Technological University for the Start Up Grant funding this research.

Appendix A. Supplementary data

Supplementary data to this article can be found online at https://doi.org/10.1016/j.jmb.2021.167380.

Received 21 September 2021;
Accepted 21 November 2021;
Available online 25 November 2021

Keywords:
pathogens;
networks;
expression;
co-expression;
function

References

1. Bray, N.L., Pimentel, H., Melsted, P., Pachter, L., (2016). Near-optimal probabilistic RNA-seq quantification. *Nature Biotechnol.* **34**, 525–527.
2. Buchfink, B., Xie, C., Huson, D.H., (2015). Fast and sensitive protein alignment using DIAMOND. *Nature Methods* **12** (1), 59–60.
3. Camacho, C., Coulouris, G., Avagyan, V., Ma, N., Papadopoulos, J., Bealer, K., Madden, T.L., (2009). BLAST+: architecture and applications. *BMC Bioinf.* **10**, 421.
4. Creecy, J.P., Conway, T., (2015). Quantitative bacterial transcriptomics with RNA-seq. *Curr. Opin. Microbiol.* **23**, 133–140.
5. Davies, J., Davies, D., (2010). Origins and evolution of antibiotic resistance. *Microbiol. Mol. Biol. Rev.: MMBR* **74**, 417–433.
6. Emms, D.M., Kelly, S., (2015). OrthoFinder: solving fundamental biases in whole genome comparisons dramatically improves orthogroup inference accuracy. *Genome Biol.* **16**, 157.
7. Gerlt, J.A., Babbitt, P.C., (2000). Can sequence determine function? *Genome Biol.* **1** REVIEWS0005.
8. Haiko, J., Westerlund-Wikström, B., (2013). The role of the bacterial flagellum in adhesion and virulence. *Biology* **2**, 1242–1267.
9. Hew, B., Tan, Q.W., Goh, W., Ng, J., Mutwil, M., (2020). LSTrAP-Crowd: prediction of novel components of bacterial ribosomes with crowd-sourced analysis of RNA sequencing data. *BMC Biol.* **18**, 114.
10. Hoffman, L.R., D'Argenio, D.A., MacCoss, M.J., Zhang, Z., Jones, R.A., Miller, S.I., (2005). Aminoglycoside antibiotics induce bacterial biofilm formation. *Nature* **436**, 1171–1175.
11. Hruz, T., Laule, O., Szabo, G., Wessendorp, F., Bleuler, S., Oertle, L., Widmayer, P., Gruissem, W., et al., (2008). Genevestigator v3: a reference expression database for the meta-analysis of transcriptomes. *Adv. Bioinform.* **2008**, 420747
12. Jiang, J., Sun, X., Wu, W., Li, L., Wu, H., Zhang, L., Yu, G., Li, Y., (2016). Construction and application of a co-expression network in Mycobacterium tuberculosis. *Sci. Rep.* **6**, 28422.
13. Jones, P., Binns, D., Chang, H.Y., Fraser, M., Li, W., McAnulla, C., McWilliam, H., Maslen, J., et al., (2014). InterProScan 5: genome-scale protein function classification. *Bioinformatics (Oxford, England)* **30**, 1236–1240.
14. Land, M., Hauser, L., Jun, S.R., Nookaew, I., Leuze, M.R., Ahn, T.H., Karpinets, T., et al., (2015). Insights from 20 years of bacterial genome sequencing. *Funct. Integr. Genomics* **15**, 141–161.
15. Leinonen, R., Sugawara, H., Shumway, M., International Nucleotide Sequence Database Collaboration, (2011). The sequence read archive. *Nucleic Acids Res.* **39**, D19–D21.
16. Lim, J., Koh, J., Moo, J.R., Villanueva, E., Putri, D.A., Lim, Y.S., Seetoh, W.S., Mulupuri, S., et al., (2020). Fungi.guru: Comparative genomic and transcriptomic resource for the fungi kingdom. *Comput. Struct. Biotechnol. J.* **18**, 3788–3795.
17. Lowe, R., Shirley, N., Bleackley, M., Dolan, S., Shafee, T., (2017). Transcriptomics technologies. *PLOS Comput. Biol.* **13**, (5) e1005457
18. Moretto, M., Sonego, P., Dierckxsens, N., Brilli, M., Bianco, L., Ledezma-Tejeida, D., Gama-Castro, S., Galardini, M., et al., (2016). COLOMBOS v3.0: leveraging gene expression compendia for cross-species analyses. *Nucleic Acids Res.* **44**, D620–D623.
19. Mutwil, M., Klie, S., Tohge, T., Giorgi, F.M., Wilkins, O., Campbell, M.M., Fernie, A.R., Usadel, B., et al., (2011). PlaNet: combined sequence and expression comparisons across plant networks derived from seven species. *Plant Cell* **23**, 895–910.
20. Mutwil, M., Usadel, B., Schütte, M., Loraine, A., Ebenhöh, O., Persson, S., (2010). Assembly of an interactive correlation network for the Arabidopsis genome using a novel heuristic clustering algorithm. *Plant Physiol.* **152**, 29–43.
21. Obayashi, T., Aoki, Y., Tadaka, S., Kagaya, Y., Kinoshita, K., (2018). ATTED-II in 2018: A Plant Coexpression Database Based on Investigation of the Statistical Property of the Mutual Rank Index. *Plant Cell Physiol.* **59**, e3
22. Obayashi, T., Kagaya, Y., Aoki, Y., Tadaka, S., Kinoshita, K., (2019). COXPRESdb v7: a gene coexpression database for 11 animal species supported by 23 coexpression platforms for technical evaluation and evolutionary inference. *Nucleic Acids Res.* **47** (D1), D55–D62.
23. Pachori, P., Gothalwal, R., Gandhi, P., (2019). Emergence of antibiotic resistance Pseudomonas aeruginosa in intensive care unit; a critical review. *Genes Dis.* **6**, 109–119.
24. Price, M.N., Dehal, P.S., Arkin, A.P., (2009). FastTree: computing large minimum evolution trees with profiles instead of a distance matrix. *Mol. Biol. Evol.* **26**, 1641–1650.
25. Proost, S., Mutwil, M., (2018). CoNekT: an open-source framework for comparative genomic and transcriptomic network analyses. *Nucleic Acids Res.* **46**, W133–W140.
26. Ramos, I., Dietrich, L.E., Price-Whelan, A., Newman, D.K., (2010). Phenazines affect biofilm formation by Pseudomonas aeruginosa in similar ways at various scales. *Res. Microbiol.* **161** (3), 187–191.
27. Rao, X., Dixon, R.A., (2019). Co-expression networks for plant biology: why and how. *Acta Biochim. Biophy. Sin.* **51** (10), 981–988.
28. Rhee, S.Y., Mutwil, M., (2014). Towards revealing the functions of all genes in plants. *Trends Plant Sci.* **19** (4), 212–221.

29. Ruan, J., Dean, A.K., Zhang, W., (2010). A general co-expression network-based approach to gene expression analysis: comparison and applications. *BMC Syst. Biol.* **4**, 8.
30. Ruer, S., Stender, S., Filloux, A., de Bentzmann, S., (2007). Assembly of fimbrial structures in Pseudomonas aeruginosa: functionality and specificity of chaperone-usher machineries. *J. Bacteriol.* **189** (9), 3547–3555.
31. Ruprecht, C., Vaid, N., Proost, S., Persson, S., Mutwil, M., (2017). Beyond Genomics: Studying Evolution with Gene Coexpression Networks. *Trends Plant Sci.* **22** (4), 298–307.
32. Saliba, A.E., Santos, C.S., Vogel, J., (2017). New RNA-seq approaches for the study of bacterial pathogens. *Curr. Opin. Microbiol.* **35**, 78–87.
33. Sayers, E.W., Cavanaugh, M., Clark, K., Ostell, J., Pruitt, K.D., Karsch-Mizrachi, I., (2020). GenBank. *Nucleic Acids Res.* **48**, D84–D86.
34. Schiessl, K.T., Hu, F., Jo, J., Nazia, S.Z., Wang, B., Price-Whelan, A., Min, W., Dietrich, L., (2019). Phenazine production promotes antibiotic tolerance and metabolic heterogeneity in Pseudomonas aeruginosa biofilms. *Nature Commun.* **10** (1), 762.
35. Subramani, R., Jayaprakashvel, M., (2019). Bacterial Quorum Sensing: Biofilm Formation, Survival Behaviour and Antibiotic Resistance. In: Bramhachari, P. (Ed.), *Implication of Quorum Sensing and Biofilm Formation in Medicine, Agriculture and Food Industry*. Springer, Singapore.
36. Taylor, P.K., Yeung, A.T., Hancock, R.E., (2014). Antibiotic resistance in Pseudomonas aeruginosa biofilms: towards the development of novel anti-biofilm therapies. *J. Biotechnol.* **191**, 121–130.
37. Tseng, K.C., Li, G.Z., Hung, Y.C., Chow, C.N., Wu, N.Y., Chien, Y.Y., Zheng, H.Q., Lee, T.Y., et al., (2020). EXPath 2.0: An Updated Database for Integrating High-Throughput Gene Expression Data with Biological Pathways. *Plant Cell Physiol.* **61** (10), 1818–1827.
38. UniProt Consortium, (2021). UniProt: the universal protein knowledgebase in 2021. *Nucleic Acids Res.* **49** (D1), D480–D489.
39. van Dam, S., Craig, T., de Magalhães, J.P., (2015). GeneFriends: a human RNA-seq-based gene and transcript co-expression database. *Nucleic Acids Res.* **43**, D1124–D1132.
40. Van Dam, S., Võsa, U., van der Graaf, A., Franke, L., de Magalhães, J.P., (2018). Gene co-expression analysis for functional classification and gene-disease predictions. *Briefings Bioinf.* **19**, 575–592.
41. Wirka, R.C., Pjanic, M., Quertermous, T., (2018). Advances in Transcriptomics. *Circ. Res.* **122**, 1200–1220.
42. Zahrt, T.C., Wozniak, C., Jones, D., Trevett, A., (2003). Functional Analysis of the Mycobacterium tuberculosis MprAB Two-Component Signal Transduction System. *Infect. Immun.* **71**, 6962–6970.
43. Zhao, X., Yu, Z., Ding, T., (2020). Quorum-sensing regulation of antimicrobial resistance in bacteria. *Microorganisms* **8**, 425.
