# Supplementary material for: PDBx/mmCIF Ecosystem: Foundational Semantic Tools for Structural Biology
Source: J Mol Biol. Author manuscript; Available in PMC 2023 Jun 26. (PMC10292674; doi:10.1016/j.jmb.2022.167599)
Supplement: Article [file NIHMS1907597-supplement-Article.zip › Outside-Front-Cover_2022_Journal-of-Molecular-Biology.pdf]

15 June 2022 / Volume 434 / Issue 11

ELSEVIER

jmb
Journal of Molecular Biology

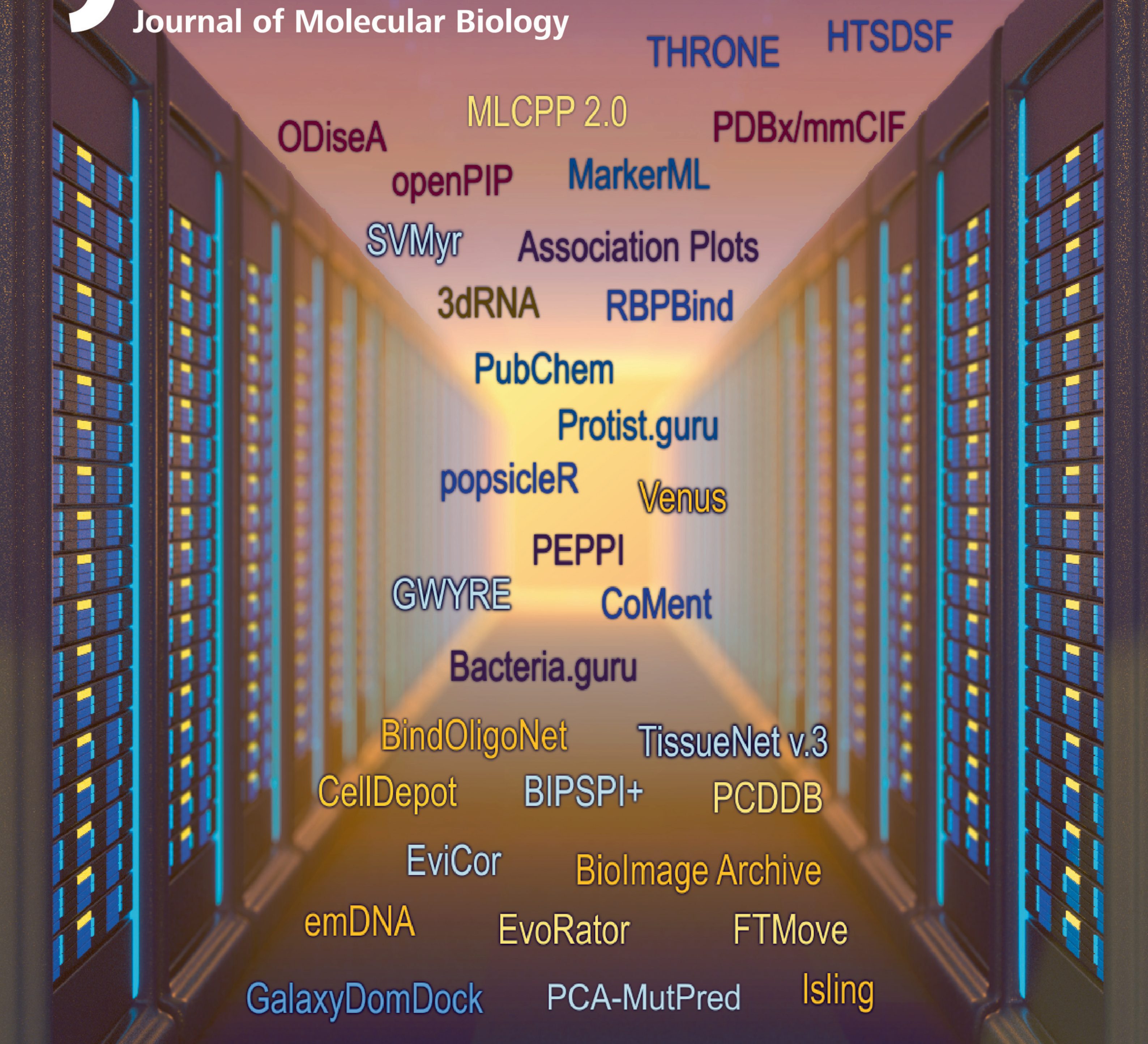

Special Issue:

Computation Resources for Molecular Biology

Edited by

Rita Casadio, David H. Mathews and Michael J. E. Sternberg
