# Supplementary material for: PDBx/mmCIF Ecosystem: Foundational Semantic Tools for Structural Biology
Source: J Mol Biol. Author manuscript; Available in PMC 2023 Jun 26. (PMC10292674; doi:10.1016/j.jmb.2022.167599)
Supplement: Article [file NIHMS1907597-supplement-Article.zip › The-Four-mRNA-Bases-Have-Quite-Different--Un-folding-Free-E_2022_Journal-of-.pdf]

Research Article

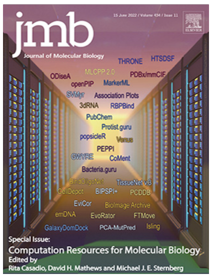

The Four mRNA Bases Have Quite Different (Un)folding Free Energies, Applications to RNA Splicing and Translation Initiation with BindOligoNet

Julian M. Hess[†] **William K. Jannen and Daniel P. Aalberts**[*]

Williams College, *Williamstown, MA 01267, USA* [1]

Correspondence to Daniel P. Aalberts: aalberts@williams.edu *(D.P. Aalberts)*
https://doi.org/10.1016/j.jmb.2022.167578
Edited by David Mathews

Abstract

Expression of mRNA is often regulated by the binding of a small RNA (miRNA, snoRNA, siRNA). While the pairing contribution to the net free energy is well parameterized and can be computed in $O(N)$ time, the cost of removing pre-existing mRNA secondary structure has not received sufficient attention. Conventional methods for computing the unfolding free energy of a target mRNA are costly, scaling like the cube of the number of target bases $O(N^3)$. Here we introduce a model to describe the unfolding costs of the binding site, which features surprisingly big differences in the free energy parameters for the four bases. The model is implemented in our $O(N)$ algorithm, BINDOLIGONET. Donor splice site prediction is more accurate when using our calculation of spliceosomal U1-snRNA to mRNA net binding free energy. Our base-dependent free energies also correlate with efficient ribosome docking near the start codon.
© 2022 The Author(s). Published by Elsevier Ltd.

Introduction

Genes are often regulated when small RNAs pair to longer folded messenger RNAs. The net free energy of binding the nucleic acids consists of the pairing energy, offset by the cost to unpair the target's binding site. Determining the unpairing costs has been particularly expensive computationally. In this paper, we build a model of unpairing from measurements of the average unpairing costs for each of the four bases. The free energies of guanine and adenine differ significantly. We then use the model to study the binding of mRNA splicing factor U1 to mRNA targets and show that our net free energy model improves splice site prediction accuracy. While previous binding algorithms run in time proportional to the cube of the length of the full target sequence, our BINDOLIGONET algorithm calculates the optimal net energy to bind an oligo to a target region in time proportional to the length of the target.

Important examples of bimolecular nucleic acid interactions are mRNA splicing,[1,2] microRNA interactions,[3–6] PCR primers,[7,8] and DNA microarrays,[9] retrotransposons,[10] Shine–Dalgarno sequences,[11] the snoRNA–rRNA associations that guide methylation and pseudouridinylation,[12] and CRISPR.[13] In most of these situations, there is a short oligo s, and a long target t. Oligos ($\lesssim$ 20 bases) are generally devoid of secondary structure while targets have about half of their bases paired. Physical descriptions of pairs or networks of interactions require computing net binding free energies, a computation that may be prohibitively expensive. Here we present a method to compute net free energies that offers physical insight, as well as a dramatic improvement in speed and accuracy.

Bimolecular association requires clearing the binding region τ of target t of any pre-existing secondary structure. The net free energy of binding is

$$\Delta G_{\text{net}} = \Delta G_{\text{bind}} + \Delta G_{\text{unpair}}, \quad (1)$$

where ΔG_{unpair} represents the cost of breaking secondary structure in region τ of t, and ΔG_{bind} represents the gain from base-pairs binding oligo s to region τ.

Computing ΔG_{unpair} is related to the RNA folding problem. The free energy of folding RNA can be computed using a thermodynamic model[14] and a dynamic programming algorithm. Well-known software packages such as MFOLD,[15] UNAFOLD,[16] VIENNARNA,[17] or RNASTRUCTURE[18,19] compute Minimum Free Energy (MFE) or Partition Functions in $O(|t|^3)$ time.

Binding free energies ΔG_{bind}, on the other hand, take only $O(|s| \cdot |t|)$ to compute using our BINDIGO,[20] the RNAHYBRID,[21] the RNAPLEX,[22,23] or the RISEARCH[24] programs. All employ a Smith-Waterman[25] local alignment approach to compute binding free energies.

Neither BINDIGO, RNAHYBRID, nor RISEARCH account for the secondary structure removal cost ΔG_{unpair} of Eq. (1). If both s and t are oligomeric, then $\Delta G_{\text{unpair}} = 0$ because there are no pairs on either strand. But if target t is long, then $\Delta G_{\text{unpair}} > 0$ to remove pre-existing secondary structure.

One approach to computing ΔG_{unpair} is to recompute the MFE after prohibiting a binding region τ from forming pairs. This can be very computationally expensive since each folding computation conventionally requires $O(|t|^3)$ time, along with $O(|t|^2)$ possible intervals for general s or $O(|t|)$ if s is a reverse complement of fixed length. Thus the brute force approach can take $O(|t|^5)$ time, as in PITA[26] or $O(|t|^4)$ for OLIGOWALK.[27] More recently RNAPLFOLD[28] has instead cleverly reused the dynamic programming matrix to obtain all ΔG_{unpair} values in $O(|t|^3)$. And $O(|t|^3)$ ACCESSFOLD[29] approximates net unpairing free energies from the partition-function probabilities of each base i being paired. An approach[4] based on removing pairs from stochastically sampled structures has also been used.

In this paper, we describe an $O(|s| \cdot |t|)$ algorithm that instead uses the *mean* unfolding cost to compute the net folding free energy

$$\Delta G_{\text{net}} \approx \Delta G_{\text{bind}} + \langle \Delta G_{\text{unpair}} \rangle, \tag{2}$$

in place of brute-force computation. In addition to speed, a benefit of our regression approach is to reveal intriguing differences in the mean unfolding and folding free energies of the four different RNA bases.

In the next Section, we introduce a series of models for $\langle \Delta G_{\text{unpair}} \rangle$, investigate their accuracy, and discuss their implementation into our BINDOLIGONET algorithm. Later, we apply our composition-dependent model to predict donor splice sites more accurately than brute-force methods and to observe correlations with the efficiency of translating the mRNA into protein.

Methods

Models for ΔG_{unpair}

Because brute-force computation of ΔG_{unpair} is costly, we shall first propose several linear models for $\langle \Delta G_{\text{unpair}} \rangle$ consistent with better algorithmic performance. Model parameters are subsequently fixed through regression.

RNAPLEX[22] introduced an average free energy penalty per removed base, $g_L = 0.3\ \text{kcal}/(\text{mol} \cdot \text{base})$ in binding region τ:

$$\Delta G_1 = g_L |\tau|. \tag{3}$$

We now generalize Eq. (3) to a linear two-parameter model

$$\Delta G_2 = g_0 + g_L |\tau|, \tag{4}$$

which includes costs g_0 to initiate and g_L to extend the unpaired region. Note that models ΔG_1 and ΔG_2 depend only on the length of the binding region.

Hypothesizing that the composition of the bases in the binding region may play a significant role, we introduce composition-based models. Our five parameter model for $\langle \Delta G_{\text{unpair}} \rangle$ is

$$\Delta G_5 = g_0 + \sum_{\alpha} g_\alpha N_\alpha, \tag{5}$$

where g_α is the mean cost of removing base $\alpha = \{A, C, G, U\}$ from the secondary structure, and N_α counts α in τ. For example, if $\tau = \text{AGAUAG}$, then $N_A = 3, N_G = 2$, etc.

We also explored a model with dinucleotides as its sequence parameters,

$$\Delta G_{17} = g_0 + \sum_{\alpha\beta} g_{\alpha\beta} N_{\alpha\beta}, \tag{6}$$

where $g_{\alpha\beta}$ is the mean cost of removing dinucleotide $\alpha\beta$ from secondary structure and $N_{\alpha\beta}$ is the number of dinucleotides $\alpha\beta$ in sequence τ. For example, if $\tau = \text{AGAUAG}$, then $N_{AG} = 2, N_{GA} = 1$, etc.

Calculating model parameters

To determine the parameters $\{g\}$ of the unpairing models we perform multiple linear regression on data sets of computed unpairing costs,

$$\Delta G_{\text{exp}}(\tau) = \Delta G'_{\text{fold}}(\tau) - \Delta G_{\text{fold}}, \tag{7}$$

where ΔG_{fold} is the MFE and $\Delta G'_{\text{fold}}(\tau)$ is the MFE of the same sequence with the added constraint of prohibiting region τ from pairing as computed by UNAFOLD.

Our datasets derive from 10^5 sequences t of length $L \in \{200, 250, 300, 350, 400\}$ randomly drawn from the *E. coli* genome or from random 30mer chunks of human precursor mRNA. For each t, a random binding region τ is chosen with length $2 \leqslant |\tau| \leqslant 20$ and position $i > 20$ and $(i + |\tau|) < (L - 20)$; the sequence ends were avoided because we observe a reduced probability of pairing of the outermost five bases (see Figure S1). These data sets are available in supplemental files.

In Table 1, we present the adjusted-R^2 values obtained from multiple linear regression with R.[30] Better models have higher values of R^2_{adj}. Table 1 shows that ΔG_5 is clearly superior to ΔG_2, indicating that the four bases are significantly different. Interestingly, the predictive power of the dinucleotide ΔG_{17} model is comparable to the simpler ΔG_5 model, despite its added complexity. For simplicity, we shall choose the easily interpretable ΔG_5 model for our implementation and analysis.

The ΔG_2 and ΔG_5 model parameters are presented in Table 2 (ΔG_{17} in Table S2). A few aspects of the parameters stand out. First, the average unpairing cost is highly base dependent; mean free energies to unpair A and G bases vary by more than $1\ \text{kcal/mol} \cdot \text{base}$ ($\approx 2RT$). Second, adenine's effect is destabilizing. After paying initiation cost g_0, forcing adenines to be unpaired produces a relative benefit of $-0.1\ \text{kcal/mol} \cdot \text{base}$. Adenine has been known to be often unpaired,[31] but its destabilizing effect on RNA secondary structure is curious. Third, the regression parameters are quite consis-

Table 1 The adjusted R^2 values for the *E. coli* and human data sets shows that the 5 parameter model is much more predictive than the 2 parameter model, demonstrating that the different free energies of the four bases are significant. The 17 parameter di-nucleotide model does not yield a significant improvement despite its greater complexity.

Model	R^2_{Ecoli}	R^2_{Human}
ΔG_2	0.336	0.277
ΔG_5	0.523	0.490
ΔG_{17}	0.516	0.492

Table 2 The ΔG_2 and ΔG_5 model parameters in (kcal/mol) to unpair bases. The parameters are similar for *E. coli* and Human despite genomic differences. The parameter g_L is the composition-weighted average of g_A, g_C, g_G, g_U.

$\{g\}$	ΔG_{Ecoli}	ΔG_{Human}
g_0	0.99	0.89
g_L	0.38	0.32
g_0	0.97	0.87
g_A	−0.10	−0.04
g_C	0.42	0.46
g_G	1.07	0.99
g_U	0.11	0.09

tent across *E. coli* and human data, despite differences in the genomic base frequencies of these organisms; thus, we expect relative consistency in other organisms.

Implementation

We modified our earlier BINDIGO program[20] to implement the five-parameter model for $\langle \Delta G_{unpair} \rangle$ creating BINDOLIGONET.[32] One- and two-parameter models, ΔG_1 and ΔG_2 and classic BINDIGO (i.e. $\Delta G_0 \equiv \Delta G_{unpair} = 0$) are all special cases of the more general five-parameter model ΔG_5. The recursion formulas for BINDOLIGONET are presented in the Supplement. BINDOLIGONET reports the best net binding free energy, traceback alignments, and free energy landscape $\Delta G(j)$ described below.

We visualize the pairing landscape by plotting, for each base *j* in the target strand, the lowest net free energy among alignments in which target base *j* is paired to the oligo. To find $\Delta G(j)$, we trace back from all negative net free energy final alignments and report the minimum states in which *j* is occupied in a pair.

Results

Application: splicing of human mRNA

As one application of our composition-dependent free-energy model ΔG_5, we considered the binding of U1-snRNA to *Real* donor splice sites versus other *Decoy* locations. Our physical-chemical hypothesis is that *Real* donor splice sites can be discriminated by their lower net binding free energy. More strongly bound states have a greater Boltzmann/Gibbs probability of occupancy.

In Figure 1, we present plots of the local free energy $\Delta G(j)$ of U1 binding to the first 3000 nucleotides of gene NM_000374.4 with the *Real* donor splice sites annotated in red. Relative to $\Delta G_{bind}(j)$ alone, we see that background pairing is suppressed in $\Delta G_{net}(j)$. Non-specific binding is reduced because the five-parameter unpairing model penalty grows with the size of the target binding region. The splice junctions do not perfectly correspond to the largest peaks in Figure 1, but we shall show next that the accuracy of predictions does indeed improve.

Donor splice sites contain a conserved GU dinucleotide surrounded by a disperse distribution of bases. The CCDS database (release 11) lists the *Real* donor splice sites within coding regions of the human genome.[33] There are 164 948 *Reals* with the consensus GU (and 1491 non-GU variants which are discarded).

Our *Decoy* set is the 51 192 651 GU dinucleotides within CCDS coding boundaries that are not in the *Real* set. For both *Real* and *Decoy* sets, we create 102-mer target sequences *t* consisting of the GU and its surrounding 50-mer flanking sequences. We then calculate the net free energy of pairing the U1 binding site, $s =$ AUACUUACCUGGC to each *Real/Decoy* target *t*. Since there may be other *Reals* or *Decoys* in the flank regions, we select the lowest free energy configuration in which the consensus GU of the target $t_{51..52}$ is paired to $s_{7..8} =$ AC of the U1-snRNA.

Different models for ΔG_{unpair} were evaluated: the composition-dependent ΔG_5 model, the length-only ΔG_2 model, the original BINDIGO with zero unfolding cost ΔG_0, and the explicit ΔG_{enum} and ΔG_{plfold}, described next. For ΔG_{enum} we use RNASTRUCTURE in MFE mode and enumerate over 169 possible alignment windows (starting 0 to 12 bases before the consensus site and ending 0 to 12 bases after the consensus site). RNAPLFOLD[28] calculations of partition-function free energies are used to compute ΔG_{plfold}; this is similar to hybrid MFE/partition-function based approaches (IntaRNA,[34] RNAxs[35]) that estimate ΔG_{net} by calculating ΔG_{bind} using an MFE traditional local alignment approach and obtaining ΔG_{unpair} from the partition-function method RNAPLFOLD. The binding contribution ΔG_{bind} is common to all methods.

The accuracy of all methods is constrained by the fact that local 9-mer sequences in the *Real* data set also appear in the *Decoy* data set. Because the sets of local sequences are overlapping, every True Positive sequence generates some False Positives, which places an upper bound on the possible accuracy. The Primary Sequence Ranking (PSR) method[36] rank orders the 4^7 different local sequences (the GU is conserved) to maximize prediction accuracy, which estimates the upper bound in Figure 2. Beyond local primary sequence, additional effects such as splicing

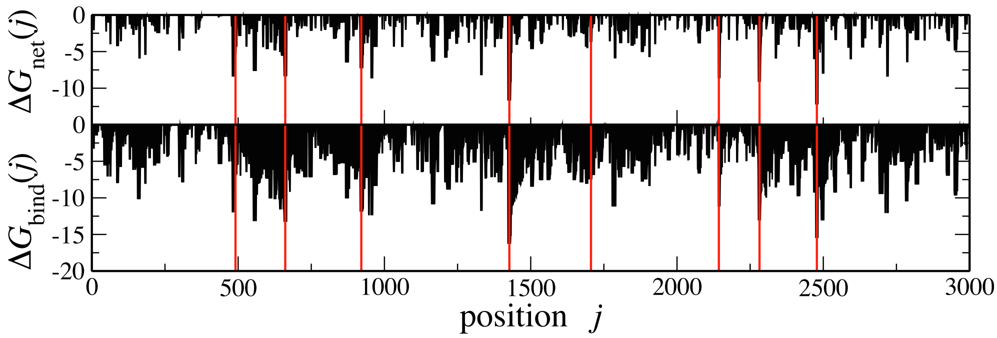

Figure 1. The optimal binding free energy $\Delta G_{bind}(j)$ that includes a pair at position j, and $\Delta G_{net}(j)$ that adds the five parameter model for unpairing, of the U1-snRNA binding site to NM_000374.4. Annotated splice sites are denoted by vertical red lines.

enhancers and silencers must be considered.[37] We note that the models are based solely on the Turner thermodynamic model[14] and have no free parameters.

The ROC curves of Figure 2 show the relative performance of the methods. Methods that produce curves closer to the top left corner are preferable. BINDIGO predicts low free energies for complementary sequences. But most *Real* sequences have one or two mismatches from the consensus and every base appears at every position flanking the GU. BINDIGO does not penalize

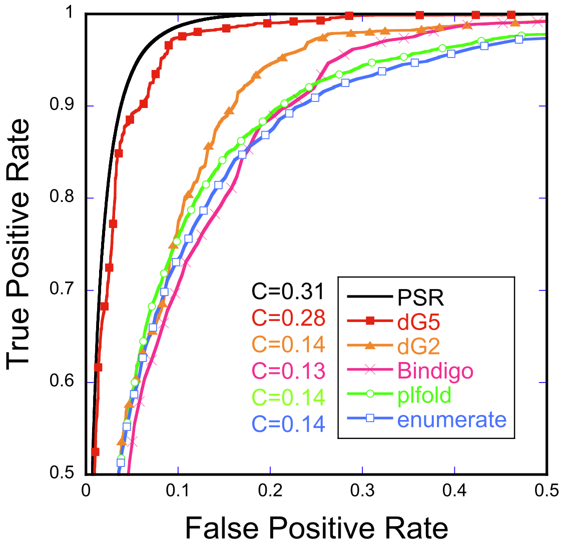

Figure 2. ROC curves for different models of ΔG_{unpair}. For *Real* and *Decoy* sites, the configurations with the lowest free energy featuring consensus GU/AC pairing are selected. Matthews correlation coefficients for BINDOLIGONET with different ΔG_{unpair} models: BINDIGO ($\Delta G_0 = 0$), and removal costs as calculated by RNAPLFOLD and by enumerating MFE states with prohibited pairs. The Matthews correlation coefficient C is reported for each method.

unpairing the target, and so pairing to a larger target region by introducing bulge/internal loops sometimes minimizes the pairing free energy.

ΔG_2 imposes a penalty proportional to the number of unpaired bases on the target strand, so the ΔG_2 model penalizes big binding sites. As is evident from the ROC curve, ΔG_2 reduces the False Positive Rate (FPR), particularly for matches with intermediate free energy (e.g., with True Positive Rate (TPR) between 0.8 and 0.95). The Area Under the Curve (AUC) increases.

ΔG_5 imposes sequence specific penalties for unpairing the target strand. The performance of ΔG_5 is quite remarkable. Using a purely thermodynamic, no-free-parameters model, the False Positive Rate of ΔG_5 is reduced almost to the limit of what can be achieved with PSR by rank-ordering 9mer sequences to maximize the TPR/FPR ratio.

Enumeration and PLFOLD are slightly more predictive than BINDIGO, consistent with the above. However, we speculate that they suffer from discretization noise — the MFE is one particular microstate in which each base is either unpaired and consequently has no free energy unpairing cost, or is paired and has a full unpairing energy. Partition-function calculations reduce the discretization noise, but not dramatically. Our regression approach replaces intrinsically noisy all-or-nothing values with smoothed-out expected values. Discretization noise also shows up in MFE calculations when the sequence window changes: adding or subtracting a base can make a distant location in the free-energy landscape the minimum, with a dramatically different structure. In living cells, mRNA structures will be continuously remodeled by thermal fluctuations, random interactions, and translating ribosomes, so we speculate that our ΔG_5 mean values may better represent reality than MFEs.

The net free energy G_τ is compared to a threshold Γ to assign:

$$\begin{aligned}
\text{true positives TP} &= \textit{Reals} \text{ with } G_\tau < \Gamma,\\
\text{false negatives FN} &= \textit{Reals} \text{ with } G_\tau > \Gamma,\\
\text{false positives , FP} &= \textit{Decoys} \text{ with } G_\tau < \Gamma, \text{ and}\\
\text{true , negatives , TN} &= \textit{Decoys} \text{ with } G_\tau > \Gamma.
\end{aligned}$$

The Matthews correlation coefficient,[38] which is the optimal binary Pearson correlation coefficient between prediction and reality,

$$C = \frac{(\mathrm{TP}\cdot\mathrm{TN}) - (\mathrm{FN}\cdot\mathrm{FP})}{\sqrt{(\mathrm{TP}+\mathrm{FN})(\mathrm{TN}+\mathrm{FP})(\mathrm{TP}+\mathrm{FP})(\mathrm{TN}+\mathrm{FN})}},$$

provides another convenient measure of the relative accuracy of the methods. The maximal C values are also reported in Figure 2.

Including unfolding costs aids in discriminating *Reals* from *Decoys* because intramolecular pairs competing for the binding site exclude poor matches and non-compact binding configurations. With $\Delta G_{\text{unpair}} = 0$, it is often energetically favorable to extend internal loops until s encounters a complementary region, often far from the *Decoy* consensus site. By imposing a secondary structure removal penalty, large loops become too costly, forcing optimal alignments to be localized around the *Decoy* GU.

Application: ribosome initiation

To initiate translation, bacterial ribosomes bind to single-stranded mRNA at the start site. Strong mRNA secondary structure in that region inhibits protein expression.[39] Our ΔG_5 model predicts that more guanine content should increase mRNA folding stability and thus slow the translation initiation rate, while adenine should have the opposite effect.

Protein expression levels of 6348 genes drawn from diverse organisms and over-expressed using a pET21 vector in *E. coli* with a T7 polymerase were measured; of these, 1973 were high expression (E5) and 1754 were undetectable expression (E0), with 2621 intermediate expression categories.[40]

In Figure 3, we observe that the probability of high (E5) versus high + low (E5 + E0) expression does indeed depend on the base composition, particularly in the first 18 bases of the coding sequences where unpaired bases are physically protected by the ribosome in the initiation complex.[41] Because a pET21 expression vector was used for all experiments, only in the coding region can the difference in expression between the four bases be observed in Figure 3(a).

Similar biases of the four bases are also present in native *E. coli* genes, see Figure 3(b). This is consistent with a selective pressure for weak RNA secondary structure to help define the Ribosome binding site at the start codon.

Discussion

Binding small RNA oligos to long target mRNA is important in many biological contexts. To allow for efficient computation of the binding free energy, we introduce a base-dependent free-energy model for the pairing region. Our BINDOLIGONET algorithm has $O(|s|\cdot|t|)$ run-time. The recent LINEARFOLD and LINEARPARTITON algorithms[42,43] can now speed up explicit computations to $O(|t|)$, from the conventional $O(|t|^3)$; however, Figure 2 shows that enumerative approaches perform less well than our ΔG_5 model.

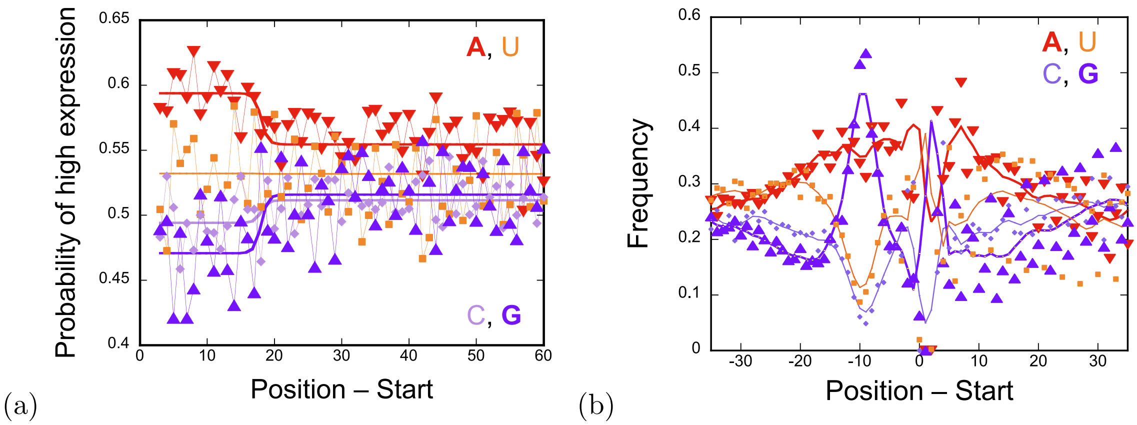

Figure 3. (a) The probability of high expression of a gene is correlated with the composition of the first 18 bases, where the ribosome initiation complex requires single-stranded mRNA. Consistent with our model of unpairing costs, adenines are particularly beneficial and guanines are the most costly. (b) Surrounding the *E. coli* genomic start codons, the frequency of adenines increases and the frequency of guanines decreases. T and C frequencies are less biased, consistent with the suggestion from our parameters that A and G would be under greater selective pressure for their stronger influence on the single strandedness at the initiation site. The G peak at $-9, -10$ is known, and attributed to the Shine-Dalgarno interaction. The symbols give the raw counts, indicating a codon periodicity of 3nt. The trend line is after smoothing.

Our main findings are that: (1) the typical unfolding energies vary dramatically for different bases, with guanine most costly to unpair and adenine the least costly, (2) most of the variation in ΔG_{unpair} is captured by taking the mean value for each base type, plus a penalty g_0 to initiate the gap, (3) including mean unfolding costs greatly improves accuracy of predicting donor splice sites and reducing background pairing, and (4) the average unfolding cost trends can also be observed in the base composition of the ribosome initiation site.

While pleased with the performance of model ΔG_5, we wondered if it could be improved upon. Although a dinucleotide model was not especially beneficial, we found that augmenting with six quadratic terms $(N_C f_G, N_G f_C, N_C f_C, N_G f_G, N_A f_T, N_T f_A)$ raises R^2_{Ecoli} to 0.542 from 0.523. The quadratic terms couple the unpaired base with the frequency of bases in the entire mRNA sequence, where $f_\alpha = (N_\alpha/L - 0.25)$ gives the deviation of the frequency of base α from uniform. The coefficient of a quadratic term like $N_G f_C$ is the slope $\partial g_G/\partial f_C$, and reflects how the fraction of cytosines in the mRNA affects the likelihood of a guanine base being paired in equilibrium, and thus its cost to be unpaired. The ΔG_{11} *E. coli* model parameters

$$\begin{aligned} g_0 &= 0.97, \\ g_A &= -0.10 + 1.4 f_T, \\ g_C &= 0.46 + 4.6 f_G - 3.2 f_C, \\ g_G &= 1.07 + 3.6 f_C - 2.5 f_G, \\ g_T &= 0.10 + 1.0 f_A, \end{aligned} \tag{8}$$

hint at how the ΔG_5 parameters might vary for organisms with different base composition.

BINDOLIGONET is available online via http://rna.williams.edu/ where users can upload sequences s and t, either one at a time or in batches. The system defaults to RNA with ΔG_5, but users can also select DNA, with pairing free energies from Ref.[19] and ΔG_5^{DNA} unpairing parameterized in Table S3. BINDOLIGONET outputs the optimal net free energies, optimal alignments, and free energy landscape $\Delta G_{\text{net}}(j)$. The source code is available from the https://github.com/wjannen/BindOligoNet repository.

Modeling networks of interacting nucleic acids should be easier with BINDOLIGONET. Our use of the mean unfolding free energy removes a computational bottleneck and may represent the thermodynamic ensemble of mRNA configurations better than MFE configurations.

CRediT authorship contribution statement

Julian M. Hess: Software, Visualization. **William K. Jannen:** Conceptualization, Software. **Daniel P. Aalberts:** Conceptualization, Methodology, Supervision.

DECLARATION OF COMPETING INTEREST

The authors declare the following financial interests/personal relationships which may be considered as potential competing interests: DPA is a co-inventor on a patent to increase protein expression with designed synonymous mRNA.

Acknowledgments

This work was supported by the National Institutes of Health [grant GM080690 and GM106372 to D.P.A.]. The authors thank Nathan O. Hodas for his work on the original BINDIGO algorithm, Joel T. Clemmer for discussions, Nagarajan Nandagapal for developing the program used to package Turner parameter tables, and the Williams College Computer Science Department for generous use of their computing cluster.

Appendix A. Supplementary Data

Supplementary data associated with this article can be found, in the online version, at https://doi.org/10.1016/j.jmb.2022.167578.

Received 24 November 2021;
Accepted 1 April 2022;
Available online 6 April 2022

Keywords:
nucleic acids;
Small RNA;
messenger RNA;
gene regulation;
ribosome initiation

† Present address: The Broad Institute, Cambridge, MA 02142, USA.

References

1. Nilsen, T.W., (1994). RNA-RNA interactions in the spliceosome—unraveling the ties that bind. *Cell* **78**, 1–4.
2. Garland, J.A., Aalberts, D.P., (2004). Thermodynamic modeling of donor splice site recognition in pre-mRNA. *Phys. Rev. E* **69**, 041903.
3. Grimson, A., Farh, K.K.H., Johnston, W.K., Garrett-Engele, P., Lim, L.P., Bartel, D.P., (2007). MicroRNA targeting specificity in mammals: Determinants beyond seed pairing. *Mol. Cell* **27**, 91–105.
4. Long, D., Lee, R., Williams, P., Chan, C.Y., Ambros, V., Ding, Y., (2007). Potent effect of target structure on microRNA function. *Nat. Struct. Mol. Biol.* **14**, 287–294.
5. Hibio, N., Hino, K., Shimizu, E., Nagata, Y., Ui-Tei, K., (2012). Stability of miRNA 5′ terminal and seed regions is correlated with experimentally observed miRNA-mediated silencing efficacy. *Sci. Rep.* **2**, 996.

6. Meng, Y., Aalberts, D.P., (2013). Free Energy Cost of Stretching mRNA Hairpin Loops Inhibits Small RNA Binding. *Biophys. J.* **104**, 482–487.
7. Saiki, R.K., Gelfand, D.H., Stoffel, S., Scharf, S., Higuchi, R., Horn, G.T., Mullis, K.B., Erlich, H.A., (1988). Primer-directed enzymatic amplification of DNA with a thermostable polymerase. *Science* **239**, 487–491.
8. Mann, T., Humbert, R., Dorschner, M., Stamatoyannopoulos, J., Noble, W.S., (2009). A thermodynamic approach to PCR primer design. *Nucleic Acids Res.* **37**, e95.
9. Pease, A.C., Solas, D., Sullivan, E.J., Cronin, M.T., Holmes, C.P., Fodor, S.P., (1994). Light-generated oligonucleotide arrays for rapid DNA sequence analysis. *Proc. Natl. Acad. Sci. USA* **91**, 5022–5026.
10. Ichiyanagi, K., Beauregard, A., Lawrence, S., Smith, D., Cousineau, B., Belfort, M., (2002). Retrotransposition of the Ll. LtrB group II intron proceeds predominantly via reverse splicing into DNA targets. *Mol. Microbiol.* **46**, 1259–1272.
11. Shine, J., Dalgarno, L., (1975). Determinant of cistron specificity in bacterial ribosomes. *Nature* **254**, 34–38.
12. Lowe, T.M., Eddy, S.R., (1999). A computational screen for methylation guide snoRNAs in yeast. *Science* **283**, 1168–1173.
13. Sternberg, S.H., Redding, S., Jinek, M., Greene, E.C., Doudna, J.A., (2014). DNA interrogation by the CRISPR RNA-guided endonuclease Cas9. *Nature* **507**, 62–67.
14. (a) Mathews DH, Sabina J, Zuker M, Turner, DH (1999) Expanded sequence dependence of thermodynamic parameters improves prediction of RNA secondary structure. *J. Mol. Biol.*, 288, 911–940. (b) Turner DH, Mathews DH (2010) NNDB: the nearest neighbor parameter database for predicting stability of nucleic acid secondary structure. *Nucleic Acids Res.*, 38, D280–D282.
15. Zuker, M., (1989). On finding all suboptimal foldings of an RNA molecule. *Science* **244**, 48–52.
16. Markham NR, Zuker M (2008) UNAFold: software for nucleic acid folding and hybriziation. In Keith JM, editor, *Bioinformatics, Volume II. Structure, Functions and Applications*, number 453 in *Methods in Molecular Biology*, chapter 1, pages 3–31. Humana Press, Totowa, NJ.
17. Hofacker, I.L., Fontana, W., Stadler, P.F., Bonhoeffer, L.S., Tacker, M., Schuster, P., (1994). Fast folding and comparison of RNA secondary structures. *Monatshefte Fur Chemie* **125**, 167–188.
18. Mathews, D.H., Disney, M.D., Childs, J.L., Schroeder, S.J., Zuker, M., Turner, D.H., (2004). Incorporating chemical modification constraints into a dynamic programming algorithm for prediction of RNA secondary structure. *Proc. Natl. Acad. Sci. USA* **101**, 7287–7292.
19. Reuter, J.S., Mathews, D.H., (2010). RNAstructure: software for RNA secondary structure prediction and analysis. *BMC Bioinformatics* **11**
20. Hodas, N.O., Aalberts, D.P., (2004). Efficient computation of optimal oligo-RNA binding. *Nucleic Acids Res.* **32**, 6636–6642.
21. Rehmsmeier, M., Steffen, P., Hochsmann, M., Giegerich, R., (2004). Fast and effective prediction of microRNA/target duplexes. *RNA* **10**, 1507–1517.
22. Tafer, H., Hofacker, I.L., (2008). RNAplex: a fast tool for RNA-RNA interaction search. *Bioinformatics* **24**, 2657–2663.
23. Tafer, H., Amman, F., Eggenhofer, F., Stadler, P.F., Hofacker, I.L., (2011). Fast accessibility-based prediction of RNA-RNA interactions. *Bioinformatics* **27**, 1934–1940.
24. Wenzel, A., Akbasli, E., Gorodkin, J., (2012). RIsearch: fast RNA-RNA interaction search using a simplified nearest-neighbor energy model. *Bioinformatics* **28**, 2738–2746.
25. Smith, T.F., Waterman, M.S., (1981). Identification of common molecular subsequences. *J. Mol. Biol.* **147**, 195–197.
26. Kertesz, M., Iovino, N., Unnerstall, U., Gaul, U., Segal, E., (2007). The role of site accessibility in microRNA target recognition. *Nat. Genet.* **39**, 1278–1284.
27. Mathews, D.H., Burkard, M.E., Freier, S.M., Wyatt, J.R., Turner, D.H., (1999). Predicting oligonucleotide affinity to nucleic acid targets. *RNA* **5**, 1458–1469.
28. Bernhart, S.H., Muckstein, U., Hofacker, I.L., (2011). RNA Accessibility in cubic time. *Algorithms Mol. Biol.* **6**, 3.
29. DiChiacchio, L., Sloma, M.F., Mathews, D.H., (2016). AccessFold: predicting RNA-RNA interactions with consideration for competing self-structure. *Bioinformatics* **32**, 1033–1039.
30. R Core Team (2021). R: A language and environment for statistical computing. https://www.R-project.org/.
31. Gutell, R.R., Cannone, J.J., Shang, Z., Du, Y., Serra, M.J., (2000). A story: Unpaired adenosine bases in ribosomal RNAs. *J. Mol. Biol.* **304**, 335–354.
32. https://github.com/wjannen/BindOligoNet.
33. Pruitt, K.D., Harrow, J., Harte, R.A., Wallin, C., Diekhans, M., Maglott, D.R., et al., (2009). The consensus coding sequence (CCDS) project: Identifying a common protein-coding gene set for the human and mouse genomes. *Genome Res.* **19**, 1316–1323.
34. Busch, A., Richter, A.S., Backofen, R., (2008). IntaRNA: efficient prediction of bacterial sRNA targets incorporating target site accessibility and seed regions. *Bioinformatics* **24**, 2849–2856.
35. Tafer, H., Ameres, S.L., Obernosterer, G., Gebeshuber, C. A., Schroeder, R., et al., (2008). The impact of target site accessibility on the design of effective siRNAs. *Nat. Biotechnol.* **26**, 578–583.
36. Aalberts, D.P., Daub, E.G., Dill, J.W., (2005). Quantifying optimal accuracy of local primary sequence bioinformatics methods. *Bioinformatics* **21**, 3347–3351.
37. Fairbrother, W.G., Yeh, R.F., Sharp, P.A., Burge, C.B., (2002). Predictive identification of exonic splicing enhancers in human genes. *Science* **297**, 1007–1013.
38. Matthews, B.W., (1975). Comparison of the predicted and observed secondary structure of T4 phage lysozyme. *Biochim. Biophys. Acta* **405**, 442–451.
39. Kudla, G., Murray, A.W., Tollervey, D., Plotkin, J.B., (2009). Coding-Sequence Determinants of Gene Expression in Escherichia coli. *Science* **324**, 255–258.
40. Boël, G., Letso, R., Neely, H., Price, W.N., Wong, K.-H., Su, M., Luff, J.D., Valecha, M., Everett, J.K., Acton, T.B., et al., (2016). Codon influence on protein expression in E. coli correlates with mRNA levels. *Nature* **529**, 358–363.
41. Duval, M., Korepanov, A., Fuchsbauer, O., Fechter, P., Haller, A., Fabbretti, A., Choulier, L., Micura, R., Klaholz, B. P., Romby, P., et al., (2013). Escherichia coli Ribosomal Protein S1 Unfolds Structured mRNAs Onto the Ribosome for Active Translation Initiation. *PLoS Biol.* **11**, 15.
42. Huang, L., Zhang, H., Deng, D., Zhao, K., Liu, K., David, A., (2019). Hendrix DA, and Mathews DH. *Bioinformatics* **35**, i295–i304.
43. Zhang, H., Zhang, L., Mathews, D.H., Huang, L., (2020). *Bioinformatics* **36**, i258–i267.
