# Supplementary material for: PDBx/mmCIF Ecosystem: Foundational Semantic Tools for Structural Biology
Source: J Mol Biol. Author manuscript; Available in PMC 2023 Jun 26. (PMC10292674; doi:10.1016/j.jmb.2022.167599)
Supplement: Article [file NIHMS1907597-supplement-Article.zip › EvoRator--Prediction-of-Residue-level-Evolutionary-Rates_2022_Journal-of-Mol.pdf]

Web Server

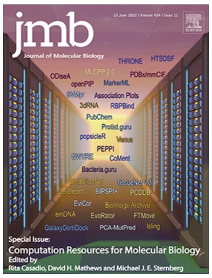

EvoRator: Prediction of Residue-level Evolutionary Rates from Protein Structures Using Machine Learning

Natan Nagar [1], Nir Ben Tal [2] and Tal Pupko [1]*

*1 - **The Shmunis School of Biomedicine and Cancer Research,** George S. Wise Faculty of Life Sciences, Tel Aviv University, Tel Aviv 69978, Israel*
*2 - **School of Neurobiology,** Biochemistry & Biophysics, George S. Wise Faculty of Life Sciences, Tel Aviv University, Tel Aviv 69978, Israel*

***Correspondence to Tal Pupko:** talp@tauex.tau.ac.il (T. Pupko)*
https://doi.org/10.1016/j.jmb.2022.167538
Edited by Rita Casadio

Abstract

Measuring evolutionary rates at the residue level is indispensable for gaining structural and functional insights into proteins. State-of-the-art tools for estimating rates take as input a large set of homologous proteins, a probabilistic model of evolution and a phylogenetic tree. However, a gap exists when only few or no homologous proteins can be found, e.g., orphan proteins. In addition, such tools do not take the three-dimensional (3D) structure of the protein into account. The association between the 3D structure and site-specific rates can be learned using machine-learning regression tools from a cohort of proteins for which both the structure and a large set of homologs exist. Here we present EvoRator, a user-friendly web server that implements a machine-learning regression algorithm to predict site-specific evolutionary rates from protein structures. We show that EvoRator outperforms predictions obtained using traditional physicochemical features, such as relative solvent accessibility and weighted contact number. We also demonstrate the application of EvoRator in three common scenarios that arise in protein evolution research: (1) orphan proteins for which no (or few) homologs exist; (2) When homologous sequences exist, our algorithm contrasts structure-based estimates of the evolutionary rates and the phylogeny-based estimates. This allows detecting sites that are likely conserved due to functional rather than structural constraints; (3) Algorithms that only rely on homologous sequence often fail to accurately measure the evolutionary rates of positions in gapped sequence alignments, which frequently occurs as a result of a clade-specific insertion. Our algorithm makes use of training data and known 3D structure of such gapped positions to predict their evolutionary rates. EvoRator is freely available for all users at: **https://evorator.tau.ac.il/**.

© 2022 Elsevier Ltd. All rights reserved.

Introduction

The quantification of residue-level evolutionary rates is a fundamental task in evolutionary studies, with implications in genetics, phylogenetic inference, biochemistry, structural biology, and drug discovery.[1–4] The Rate4Site program,[5] which is embedded in the ConSurf web server[6–10] and ConSurf database[11] uses a probabilistic modelling framework for calculating residue-level evolutionary rates. Given a multiple sequence alignment (MSA) of amino-acids, the relative conservation score for each position in the MSA is estimated, with the number of available homologues and their degree of divergence substantially impacting the estimated accuracy. Consequently, it follows that rates cannot

be accurately calculated for proteins with few or no homologs. Such proteins, also known as orphans, comprise about 10–20% of animal genomes, and are suggested to be involved in the evolution of species-specific adaptive traits.[12] In other cases, the obtained rates profile is incomplete due to missing data, i.e. positions in the MSA that only have a few un-gapped amino-acids. Such local divergences in sequence can result from clade-specific insertion events, which act as major drivers of evolution in various species.[13]

The primary determinant of site-specific evolutionary rates is thought to be structural by nature.[14–18] Proteins must be stably folded into their active conformation in order to function properly. As a result of purifying selection, the tightly-packed, rigid hydrophobic core of proteins is highly conserved compared to the loosely-packed and flexible parts.[18] Functionally important sites, such as catalytic and allosteric sites, as well as their neighbors, tend to be especially conserved.[18] By contrast, selection for rapid evolution due to a changing environment (i.e. positive selection), is expected to affect sites that are directly involved in the selected function of a given protein-coding gene.[19]

A slowly evolving site may suggest either a structural or functional constrain, or a combination of both. It is difficult to determine the relative contribution of each factor, without collecting structural information. Indeed, Echave *et al.*[20] suggested that by trying to find congruence between conservation scores and the structural constraints of protein evolution, one can potentially identify functionally important sites, such as those whose conservation cannot be well explained by structural constraints alone.

Previous studies attempted to predict evolutionary rates from structure using traditional regression modelling strategies.[14–16] The primary structural predictors of rates, relative solvent accessibility (RSA) and weighted contact number (WCN), explain approximately 40% of the observed variation in rates.[15] Structural flexibility and disorder were also shown to be predictive of rates, although to a lesser extent.[21–22] Given the general dominance of structural over functional constraints in shaping protein evolution, this observation suggests that the precise structural determinants have yet to be discovered. Moreover, most studies were mainly focused on a few features at a time, which may not fully utilize the predictive potential of the structural information at hand.[14–16] To date, such analyses are available only as standalone executables.[16,23] It is expected that a web server for computing rates and contrasting these rates with structural information should increase the accessibility of such analyses to the research community at-large.

Machine-learning (ML) approaches are commonly applied in various fields of protein research, including prediction of protein folding, degradation rates, and impact of an amino acid substitution on protein structure and function.[24–27] Here we present EvoRator, a user-friendly web server that implements an ML regression algorithm that predicts residue-level evolutionary rates from protein structure. EvoRator is designed to: (1) predict evolutionary rates for orphans; (2) predict evolutionary rates for MSA positions for which there are few homologous sequences, e.g., as a result of clade-specific insertion; (3) for positions with sufficiently large number of homologs, identify sites for which there is a discrepancy between the inferred evolutionary rate and the predicted rate based on structural features. Such sites are potentially functionally important. For the task of predicting rates from structural information, EvoRator uses in addition to traditional physicochemical measures, features extracted from a network analysis of the protein structure. We show that EvoRator outperforms predictions obtained by traditional predictors of residue-level rates and demonstrate its applicability in the three aforementioned scenarios.

Materials and methods

Dataset. We used a previously published dataset of 77,152 positions (MSA columns) assembled from 213 monomeric enzymes of diverse sizes, functional, and structural classes for training and evaluation of EvoRator.[14–15] The list of analyzed proteins and their corresponding evolutionary rates, calculated using the Rate4Site algorithm,[5] were obtained from Echave *et al.*[16] Specifically, we used the Z-normalized rates calculated by Rate4Site assuming the Jukes and Cantor replacement model.[28]

Feature extraction. We extracted for each residue physicochemical features, such as RSA and C_α-based WCN, as described in previous works on prediction of residue-level evolutionary rates from protein structures.[15,21,29–30] We also extracted features from network representations of protein structures that were previously reported to be highly predictive of various aspects of protein structure and function, such as clustering coefficient and betweenness centrality.[31–33] The network representations of protein structures were obtained from the NAPS web server[34] using default parameters. In these representations, the C_α atoms are taken as the nodes and an edge is formed if the distance between the C_α atoms is less than the default 7 Å. The full list of features that were used in this study is summarized in supplementary Table S1.

Data preprocessing. Scikit-learn is used for data preprocessing and ML.[35] EvoRator applies the following preprocessing steps: features with missing values are filled with the median of their existing values; categorical features are one-hot encoded, and

numerical features are scaled by subtracting the mean of the feature from each of its values and dividing by the standard deviation of the feature. These preprocessing steps are done based on the training set, i.e. the missing feature values of a given position in the test data are filled with the median of that feature in the training data; analogously, the feature values of a given position in the test data is standardized by subtracting the mean of that feature in the training data from it, and dividing the result by the standard deviation of that feature in the training data.

Evolutionary rates prediction using ML. Three different ML tasks can be performed using EvoRator: (1) Predicting evolutionary rates for orphans (“PERfO”); (2) Predicting evolutionary rates for gapped regions (“PERfGR”); (3) Predicting evolutionary rates for identifying functional regions (“PERfIFR”). This is done by calculating the difference between rates predicted from structure and rates inferred using Rate4Site.[5] For all three tasks, the coefficient of determination (R^2) of predicted vs. actual evolutionary rates was used as the optimization metric for algorithm training.

Multiple ML algorithms for regression were evaluated for each prediction scenario. The ML algorithms that were tested are linear regression model with L1 regularization (LASSO, sklearn.linear_model.Lasso), a support vector regression model with a radial basis function kernel (SVR, sklearn.svm.SVR), and an ensemble of randomized decision trees (Random Forest, sklearn.ensemble.RandomForestRegressor). In order to promote the generalizability of the SVR algorithm, feature selection was employed before its training. Specifically, we used the features that were selected by LASSO to train the SVR algorithm; the features were selected if their corresponding coefficients as determined by LASSO were greater or equal to the default 0.00001 (sklearn.feature_selection.SelectFromModel).

In PERfO, the learning is based on features 1–33 (Table S1). To select the best ML regression algorithm for PERfO, we used the hold-out set method. We partitioned the dataset of 213 enzymes to training, validation and test sets, comprising 133, 30 and 50 proteins, respectively. The hyper-parameters of each algorithm were determined by a grid search as follows: each algorithm was fitted to the training set using a different combination of parameters. For each algorithm, the combination with the highest R^2 over the validation set was obtained. This optimal combination was next used to evaluate the performance over the test dataset.

For PERfGR and PERfIFR, we repeated the same analysis that we performed in PERfO, only this time the learning is based on features 1–34 (Table S1). Note that the learning phase is done once for both PERfGR and PERfIFR. The learning is not specific to gapped positions, but rather, for all positions within the protein. The need for a separate learning for PERfGR and PERfIFR, is that for the latter we assume that adjacent rates might be known and can be used to help estimate the role of the position studied. Rates of neighboring sites are unknown in PERfO mode. The added feature (number 34) is based on the Rate4Site evolutionary rates of neighboring sites, where the definition of neighboring sites is based on the graph-representation of the protein structure, i.e., it may include rates of sites that are not adjacent in the primary sequence. Furthermore, it only considers neighboring sites whose rates are categorized as reliable by Rate4Site. Of note, in the learning (and also when applying the trained model), only the rates of its neighboring sites are considered, but not the Rate4Site estimate of the site in question. Similarly, the predicted rate based on EvoRator of neighboring sites are also not considered. Finally, we note that similar to the PERfO learning step described above, a single learning phase is done by considering all positions in all the proteins that are in the learning step, i.e., we do not learn a different model for each protein.

Overview of EvoRator web server

EvoRator is available as a public web server accessible at: https://evorator.tau.ac.il/. The only mandatory inputs for EvoRator are the protein data bank[36] (PDB) coordinate file and a corresponding chain identifier of the query protein. The user is allowed to either fill the PDB identifier or to upload an external coordinate file. The user can further choose to let EvoRator attempt to retrieve a ConSurf table of rates from ConSurf-DB,[5–11,37] or provide a ConSurf table that matches the input structure. The user can also provide an e-mail address to which a link to the results page is sent. The server accepts a single protein structure per job. After submission, the user is automatically redirected to the results page, and, once the job is completed, the output is shown. The results are kept on the server for three months.

The input that is provided by the user determines the task for which EvoRator will be executed. When only a structure is supplied, EvoRator assumes the user is interested in PERfO. To predict the evolutionary rates for the query protein in this case, EvoRator extracts and processes features 1–33 (Table S1), which are subsequently fed to the ML algorithm that was trained on 213 enzymes with the same set of features. The rates predicted by the trained ML algorithm are mapped onto the 3D structure of the query protein.

In case that a ConSurf table of rates is also provided, the user must choose between PERfGR and PERfIFR. In both cases, the features

extracted and processed by EvoRator are features 1–34 (Table S1). These features are subsequently fed to an ML algorithm that was trained on 213 enzymes with the same set of features. For PERfIFR, the differences between the homology-based estimates of the evolutionary rates and those predicted by the ML algorithm are mapped onto the 3D structure of the query protein. For PERfGR, predicted rates for reliable positions are based on homology and for the remaining positions, the predicted rates are based on the ML; both types of predictions are mapped onto the 3D structure. For PERfIFR we provide a contrast score, which is the difference between the evolutionary rate of ConSurf and that predicted by EvoRator based on structural features. Extreme contrast values suggest that the structural features fail to explain the observed conservation, suggesting that additional unknown factors are involved.

In all tasks, PERfO, PERfGR, and PERfIFR, the results page contains a link to the NGL viewer,[38] which is used to visualize the structure, a link to a 3D model of the network representation of the structure, and a link to a csv file that summarizes the predictions and the features that were used to obtain them. For PERfIFR, the results page also displays the regression results of the actual versus predicted rates.

EvoRator uses a visualization coloring scheme identical to ConSurf for PERfO and PERfGR. Briefly, the predicted rates are split into nine discrete bins whereas bins 1 and 9 representing the most variable and most conserved positions, respectively. For PERfIFR, we also use nine bins, in which bin 1 represents positions for which the difference between the ConSurf score and the ML score is highest, i.e., predicted functionally constrained sites. In bin 9, the difference is the lowest.

Results

Predictive performance of EvoRator

EvoRator is designed to predict evolutionary rates for orphans ("PERfO"), gapped regions ("PERfGR") and for identifying functional regions ("PERfIFR"), using ML regression. In all three tasks, EvoRator receives a protein structure as an input and the output is the predicted rates. For a detailed description of the EvoRator methodology, please refer to the Methods section.

To evaluate the predictive performance of EvoRator, we used a previously published data set of 213 enzymes. The evolutionary rates per site for the amino acid positions of each of the proteins in this set, which we consider ground truth, have been calculated using the Rate4Site algorithm based on up to 300 homologues for each enzyme, taking their phylogeny into account explicitly. This dataset was first introduced by Yeh *et al*,[15] to explore the relationship between site-specific evolutionary rates and two features: RSA and C_α-based WCN, using linear regression. We therefore compared the two following evolutionary rates prediction models: (1) a baseline model that only exploits RSA and C_α-based WCN; (2) an integrative model that combines these two features with a plethora of structural and functional features, including features that are extracted from a network representation of the protein 3D structure (Table S1). We term the baseline and integrative models as 'baseline' and 'EvoRator', respectively.

For PERfO, we compared the predictions that were obtained by the baseline method to those obtained by the EvoRator. In PERfO, the training of EvoRator is based on features 1–33 (Table S1). The comparison between the two methods is done based on the results obtained over a hold-out test set comprising 50 proteins that were randomly chosen from the set of 213 enzymes. Compared to the baseline model, the EvoRator best model that employs SVR with LASSO feature selection showed a mean R^2 of 0.42, a significant and substantial improvement of approximately 40% over the baseline model, which obtained a mean R^2 of 0.3 (Table 1; one-sided paired t-test, mean difference = 0.12, d.f. = 49, $P = 1.21 \times 10^{-15}$). To determine the best performing ML regression algorithm for EvoRator, model selection was employed (see Methods). SVR combined with LASSO feature selection obtained a mean R^2 of 0.42, a small yet significant improvement over alternative regression models such as random forest (Table S2).

For PERfGR and PERfIFR, we repeated the same analysis as described above for PERfO, using the same hold-out set, only this time using an additional feature, which measures the average evolutionary rates of neighboring positions (feature 34, Table S1). Note that in PERfO predictions are aimed for orphan proteins, and therefore Rate4Site estimation of evolutionary

Table 1 **Comparison between the performance of EvoRator and the baseline model in different prediction tasks.** R^2 averaged over the hold-out test set of fifty proteins with its standard deviation. The features used in this study are given in **Table S1**. PERfO – Prediction of evolutionary rates for orphans, PERfGR – Prediction of evolutionary rates for gapped regions, PERfIFR – Prediction of evolutionary rates for identifying functional regions.

Task	Method	Features	R^2
PERfO	EvoRator	1–32, 34	0.42 ± 0.13
	Baseline	10–11	0.3 ± 0.1
PERfGR and PERfIFR	EvoRator	1–34	0.52 ± 0.13
	Baseline	10–11	0.3 ± 0.1

rates cannot be used as a feautre in the learning. In contrast, for PERfGR and PERfIFR we assume that a large number of homologous sequences exist for some positions, and those estimates are used for predicting the rates of all positions. It should be stressed that to predict the conservation of a given site, we only use the Rate4Site evolutionary rates of its neighbors; the rate of the residue at hand, if available, is not included as a feature. Compared to the baseline model, the EvoRator model that employs SVR with LASSO feature selection obtained a mean R^2 of 0.52, demonstrating a significant and substantial improvement of approximately 73% over the baseline model, which obtained a mean R^2 of 0.3 (Table 1; one-sided paired t-test, mean difference = 0.22, d.f. = 49, $P = 5.78 \times 10^{-22}$). Model selection indicated that SVR combined with LASSO feature selection obtained the highest mean R^2 of 0.52, a significant improvement over other regression models such as random forest (Table S2). Since SVR with LASSO feature selection obtained the highest scores in all prediction scenarios, we implemented this algorithm in the EvoRator web server.

Demonstration of EvoRator web server for PERfIGR

We predicted the evolutionary rates of gapped regions in SARS-CoV-2 spike glycoprotein in its open state (PDB ID: **6VYB**) by running EvoRator in PERfIGR mode (Figure 1). For this protein, ConSurf automatically generated an alignment of 150 sequences and 1,281 alignment positions. Of these 1,281 positions, 966 positions could be reliably mapped to the known 3D structure. Out of 966 resolved residues, 157 are assigned with unreliable rates of evolution by ConSurf (Supplementary Data 1), mainly due to gapped regions in the MSA (Supplementary Data 2). EvoRator in PERfIGR mode predicted rates for these 157 sites. Interestingly, six of these sites (positions H69, V143, S373, K417, Y505, D614) were previously reported to both differentiate SARS-CoV-2 variant Omicron from the ancestral Wuhan-Hu-1 spike and were shown to impact viral infectivity and antigenicity.[45–49]

We therefore hypothesized that among gapped sites, functionally important residues that also differentiate Omicron from the ancestral Wuhan-Hu-1 spike will have higher evolutionary rates than rest of the residues. We note that the Omicron sequences were not included in the analyzed MSA. Indeed, the six functional and differentiating positions show significantly higher variability compared to the remaining 151 positions (Figure S1(A), one-sided student's t-test, mean difference = 0.57, d.f. = 155, $P = 1.83 \times 10^{-5}$).

Interestingly, H69 and V143, which experienced deletion events,[46,48] are the first and second most variable positions among the 157 gapped positions, with rates highly above the average value (red points in Figure S1(B)). In contrast, positions S373, K417, 505Y, and 614D, which were substituted in the lineage leading to the Omicron variant,[45,47,49] were predicted to be significantly less variable than H69 and V143 (ranks 16, 32, 107, and 144), with rates much closer to the average value (black points around the horizontal line in Figure S1(B)).

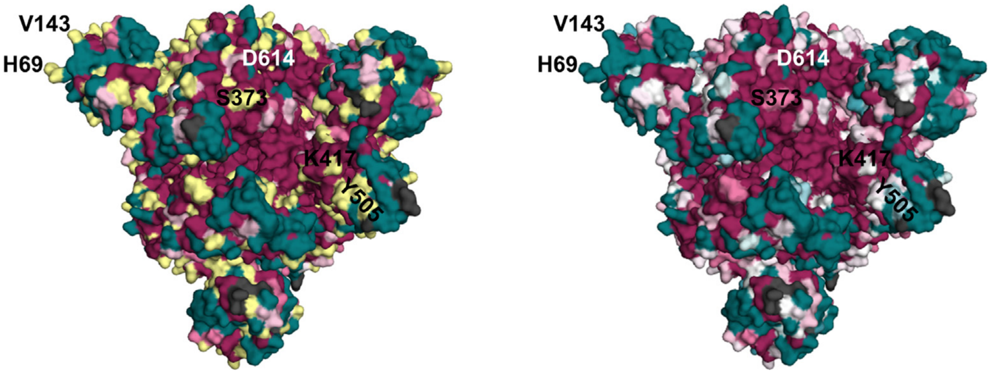

Figure 1. Visualization of EvoRator prediction for SARS-CoV-2 spike glycoprotein ectodomain structure in PERfGR mode. Electron microscopy structure of the homotrimeric spike glycoprotein (PDB ID: **6VYB**) shown in surface representation. Residues that were resolved in chains B and C, but not A, are marked in gray. (A) Visualization of ConSurf prediction with unreliable rates marked in yellow. Sites that were assigned by ConSurf with unreliable rates, at which mutations with functional implications occurred, are highlighted. H69 and V143 were deleted whereas S373, K417, Y505, and D614 were substituted in SARS-CoV-2 variant Omicron compared with the ancestral Wuhan-Hu-1 spike. (B) Visualization of EvoRator prediction. Residues that were previously assigned with unreliable rates by ConSurf are instead marked by EvoRator prediction. The predicted rates for sites H69, V143, S373, K417, Y505, and D614, are 1.30, 1.25, −0.50, −0.13, 0.1, −0.68, respectively.

Demonstration of EvoRator web server for PERfO

We used human dermcidin (PDB ID: **2YMK**) to demonstrate the applicability of EvoRator in PERfO mode (Figure S2(A)). The C-terminal of dermcidin (DCD), DCD-1L is a negatively charged anti-microbial peptide that can be found on human skin.[38] DCD-1L is involved in host defense against bacterial infections, including antibiotic-resistant strains of *Mycobacterium tuberculosis*.[39]

In the presence of zinc ions, DCD-1L monomers form a hexameric channel, which binds to membranes, thus creating a site of severe membrane disturbance.[40] Zinc binding involves coordination that is not easily achieved, and imposes very strong preference for specific polar residues. Thus, residues E5, D9, H38, and D42, which bind zinc, are expected to be highly conserved. This hypothesis is also supported by the observation that mutating H38 to alanine diminishes the antimicrobial activity of DCD-1L.[40] However, for estimating the residue-level evolutionary rates for DCD-1L, one cannot reliably use ConSurf because only 10 unique homologous proteins are found by HMMER. This resulted in unreliable estimates of rates for 36 out of 48 residues of DCD-1L, including the zinc-binding residues H38, D42 and D9. EvoRator predicts that most neighboring residues of H38, D42 and E5 should be highly conserved (Figure S2 (B) and Table S3). L3 and L4, which are neighbors of E5, are ranked 2nd and 5th most conserved residues. G35, which is a neighbor of H38, is the 3rd most conserved residue. K41, which is a neighbor of both H38 and D42, is the fourth most conserved residue. H38, which binds zinc, is the 6th most conserved residue. This shows that EvoRator can correctly indicate the location of highly conserved residues in orphan proteins, and complement ConSurf when one is interested in gaining functional insights into the structure of orphan proteins. We note, however, that EvoRator predicts that L3 and L48, which are located at the terminal ends of DCD-1L and are not involved in zinc binding, should be the most conserved residues, whereas residues E5, D9 and D42, should be much less conserved. We suspect that these discrepancies may simply reflect lack of sufficient training data for end of helices and metal-binding residues.

To further demonstrate the applicability of EvoRator to orphan proteins, we analyzed the structure of endolysin (PDB ID: **2LZM**), a well-characterized protein in PERfO mode (Figure S3). Endolysin has six residues that when mutated lead to loss of its catalytic activity.[41–42] Therefore, these residues are expected to be highly conserved due to purifying selection. EvoRator predicted that these residues have significantly lower evolutionary rates than rest of the protein (Figure S4(A), one-sided student's t-test, mean difference = -0.61, d.f. = 162, P = 0.015), further supporting the notion that EvoRator's predictions can effectively identify functional residues in orphan proteins.

Importantly, the functional features that EvoRator is designed to extract (features 6–9, Table S1) were missing for endolysin, such that EvoRator's prediction in this case is purely based on structural features. EvoRator obtained an R^2 of 0.49 for endolysin and accurately predicted the rates of two out of six functional positions (red points close to the $y = x$ line in Figure S4(B)), suggesting that the primary role of these residues is structural rather than functional. For the remaining four residues, EvoRator predicted moderately higher rates compared with ConSurf (red points above the $y = x$ line in Figure S4(B)), in line with the notion that the conservation of functional sites cannot be fully explained by structural features alone, such as those used by EvoRator.

Demonstration of EvoRator web server for PERfIFR

Echave *et al.*[20] suggested that by regressing evolutionary rates on structural constraints of protein evolution, one can identify functionally important sites. To test this hypothesis, we provided the structure of Enolase 1 asymmetric unit (PDB ID: **7ENL**), which includes a single polypeptide chain, as an input for EvoRator in PERfIFR mode (Figure S5).

Enolase 1 is a homodimer,[43] and thus the interfacing residues that are involved in its dimerization are expected to be conserved due to function (in this case, protein–protein interactions) in addition to their expected conservation based on structure (in this case, interactions between amino-acids within the same polypeptide chain). This discrepancy should be detected by contrasting the evolutionary rates based on ConSurf and those based on EvoRator, which received as input only a single chain. Specifically, the interfacing residues are expected to be more conserved than predicted by EvoRator.

Based on the PISA database,[44] there are 55 interfacing residues involved in Enolase 1 dimerization and 381 other sites. EvoRator obtained an R^2 of 0.56 for Enolase 1. The average contrast (reflecting difference between ConSurf conservation scores and those predicted by EvoRator, see Methods) among interfacing residue was found to be significantly lower (highly negative) compared with other residues of the protein (Figure S6(A), one-sided student's t-test, mean difference = -0.21, d.f. = 432, P = 0.014). As expected, the contrasts for the vast majority of interfacing residues are either close to zero or negative (red points close to or below the y = 0 in Figure S6(B)). This shows that when no prior knowledge about residue functionality is available, the contrast between ConSurf and EvoRator predictions can be highly informative for assessing residue functionality.

Discussion

In this paper we present EvoRator, the first web server that implements ML regression to predict residue-level evolutionary rates from protein 3D structure. EvoRator leverages various features to predict evolutionary rates, ranging from traditional structural features commonly used in this field, such as RSA and C_α-based WCN, to features extracted from network representation of protein 3D structure. This diverse set of features captures the rich structural information that is encoded by a protein 3D structure.

Previous studies explored the relationship between structural and biophysical features and evolutionary rates.[15–16] Although these studies and we analyzed the same dataset, their methodologies differ from that of EvoRator to an extent that does not allow a direct comparison to our results. Specifically, they used a regression for each protein analyzed, while in our study, we trained a ML regressor for all proteins together. An indirect comparison in which we compared the performance based on the RSA and C_α-based WCN features versus all features clearly shows the benefit of using a large set of features.

Our study relies on a training data of 58,554 alignment positions taken from 163 proteins (18,598 alignment positions from 50 proteins were used as test data). Clearly, the training data can be substantially increased by analyzing additional MSAs. Such an increase would allow training deep learning networks, which may lead to increased performance. It is interesting to test the accuracy of our algorithm when the 3D structure is not determined, but rather is predicted, e.g., based on AlphaFold.[26] Currently, our algorithm does not consider a large set of post-translational modifications such as myristoylation and phosphorylation. Accounting for such factors may further increase accuracy. Additionally, an alignment position may be conserved because of structural constraints at the DNA and mRNA level, e.g., to mentain correct splicing or RNA folding stability.[50] The rates of such sites is expected to be lower than those predicted by EvoRator, and those sites are expected to have high contrast when running the PERfIFR mode.

The measure of accuracy reported in this work is the R^2, which reflects the agreement between ConSurf scores and the scores predicted by EvoRator. However, caution must be taken when interpreting these accuracy measures, mainly because the conservation scores of ConSurf are also predicted, and thus should not be considered as gold standard. ConSurf also reports confidence intervals around the estimated site-specific rates. These confidence intervals tend to be high for variable sites. The R^2 accuracy score does not consider this uncertainty in the estimated ConSurf rates, which may partially explain why R^2 is less than 0.52 for all analyses.

An MSA with "true" rates can be computed using simulations (e.g., [7]) However, such standard simulations do not capture the 3D-structural constraints of the protein at hand, and hence cannot be used to test the performance of EvoRator. Ideally, simulations that reflect protein evolution that explicitly consider the protein structural constraints[51–52] should be applied to better quantify the accuracy estimation of methods such as EvoRator.

The EvoRator web server is freely available for the scientific community at https://evorator.tau.ac.il. The user interface is both simple and useful, and provide visual outputs as well as tabular output in CSV format.

Acknowledgements

Israel Science Foundation (ISF) [2818/21 to T.P. and 1764/21 to N.B.-T.]; Edmond J. Safra Center for Bioinformatics at Tel Aviv University Fellowship to N.N. T.P.'s research is supported in part by the Edouard Seroussi Chair for Protein Nanobiotechnology, Tel Aviv University. N.B.-T.'s research is supported in part by the Abraham E. Kazan Chair in Structural Biology, Tel Aviv University.

Declaration of Competing Interest

The authors declare that they have no known competing financial interests or personal relationships that could have appeared to influence the work reported in this paper.

Appendix A. Supplementary data

Supplementary data to this article can be found online at https://doi.org/10.1016/j.jmb.2022.167538.

Received 30 November 2021;
Accepted 7 March 2022;
Available online 11 March 2022

Keywords:
protein evolution;
machine learning;
protein structure;
protein function;
ConSurf;
orphan genes;
gapped alignment

References

1. Walls, A.C., Park, Y.J., Tortorici, M.A., Wall, A., McGuire, A.T., Veesler, D., (2020). Structure, function, and antigenicity of the SARS-CoV-2 spike glycoprotein. *Cell* **181**, 281–292.
2. Mongiardino Koch, N., Thompson, J.R., (2021). A total-evidence dated phylogeny of echinoidea combining phylogenomic and paleontological data. *Syst. Biol.* **70**, 421–439.
3. Bednar, D., Beerens, K., Sebestova, E., Bendl, J., Khare, S., Chaloupkova, R., Prokop, Z., Brezovsky, J., Baker, D., Damborsky, J., (2015). FireProt: energy- and evolution-based computational design of thermostable multiple-point mutants. *PLoS Comput. Biol.* **11**, e1004556
4. Kessel, A., Ben-Tal, N., (2018). Introduction to proteins: structure, function, and motion. Taylor & Francis LLC, Boca Raton, Florida.
5. Pupko, T., Bell, R.E., Mayrose, I., Glaser, F., Ben-Tal, N., (2002). Rate4Site: An algorithmic tool for the identification of functional regions in proteins by surface mapping of evolutionary determinants within their homologues. *Bioinformatics* **18**, S71–S77.
6. Celniker, G., Nimrod, G., Ashkenazy, H., Glaser, F., Martz, E., Mayrose, I., Pupko, T., Ben-Tal, N., (2013). ConSurf: Using evolutionary data to raise testable hypotheses about protein function. *Isr. J. Chem.* **53**, 199–206.
7. Glaser, F., Pupko, T., Paz, I., Bell, R.E., Bechor-Shental, D., Martz, E., Ben-Tal, N., (2003). ConSurf: Identification of functional regions in proteins by surface-mapping of phylogenetic information. *Bioinformatics* **19**, 163–164.
8. Ashkenazy, H., Abadi, S., Martz, E., Chay, O., Mayrose, I., Pupko, T., Ben-Tal, N., (2016). ConSurf 2016: an improved methodology to estimate and visualize evolutionary conservation in macromolecules. *Nucleic Acids Res.* **44**, W344–W350.
9. Landau, M., Mayrose, I., Rosenberg, Y., Glaser, F., Martz, E., Pupko, T., Ben-Tal, N., (2005). ConSurf 2005: The projection of evolutionary conservation scores of residues on protein structures. *Nucleic Acids Res.* **33**, W299–W302.
10. Ashkenazy, H., Erez, E., Martz, E., Pupko, T., Ben-Tal, N., (2010). ConSurf 2010: Calculating evolutionary conservation in sequence and structure of proteins and nucleic acids. *Nucleic Acids Res.* **38**, W529–W533.
11. Ben Chorin, A., Masrati, G., Kessel, A., Narunsky, A., Sprinzak, J., Lahav, S., Ashkenazy, H., Ben-Tal, N., (2020). ConSurf-DB: An accessible repository for the evolutionary conservation patterns of the majority of PDB proteins. *Protein Sci.* **29**, 258–267.
12. Khalturin, K., Hemmrich, G., Fraune, S., Augustin, R., Bosch, T.C.G., (2009). More than just orphans: are taxonomically-restricted genes important in evolution? *Trends Genet.* **25**, 404–413.
13. Sjödin, P., Bataillon, T., Schierup, M.H., (2010). Insertion and deletion processes in recent human history. *PLoS ONE* **5**, e8650
14. Huang, T.T., Del Valle Marcos, M.L., Hwang, J.K., Echave, J., (2014). A mechanistic stress model of protein evolution accounts for site-specific evolutionary rates and their relationship with packing density and flexibility. *BMC Evol. Biol.* **14**, 1–10.
15. Yeh, S.W., Liu, J.W., Yu, S.H., Shih, C.H., Hwang, J.K., Echave, J., (2014). Site-specific structural constraints on protein sequence evolutionary divergence: local packing density versus solvent exposure. *Mol. Biol. Evol.* **31**, 135–139.
16. Echave, J., Jackson, E.L., Wilke, C.O., (2015). Relationship between protein thermodynamic constraints and variation of evolutionary rates among sites. *Phys. Biol.* **12**, 25002.
17. Perutz, M.F., Kendrew, J.C., Watson, H.C., (1965). Structure and function of haemoglobin: II. Some relations between polypeptide chain configuration and amino acid sequence. *J. Mol. Biol.* **13**, 669–678.
18. Kimura, M., Ota, T., (1974). On some principles governing molecular evolution. *Proc. Natl. Acad. Sci. U. S. A.* **71**, 2848–2852.
19. Nielsen, R., Yang, Z., (1998). Likelihood models for detecting positively selected amino acid sites and applications to the HIV-1 envelope gene. *Genetics* **148**, 929–936.
20. Echave, J., Spielman, S.J., Wilke, C.O., (2016). Causes of evolutionary rate variation among protein sites. *Nat. Rev. Genet.* **17**, 109–121.
21. Brown, C.J., Takayama, S., Campen, A.M., Vise, P., Marshall, T.W., Oldfield, C.J., Williams, C.J., Keith Dunker, A., (2002). Evolutionary rate heterogeneity in proteins with long disordered regions. *J. Mol. Evol.* **55**, 104–110.
22. Nevin Gerek, Z., Kumar, S., Banu Ozkan, S., (2013). Structural dynamics flexibility informs function and evolution at a proteome scale. *Evol. Appl.* **6**, 423–433.
23. Wilke, C.O., Sydykova, D.K., Jack, B.R., Spielman, S.J., (2017). Measuring evolutionary rates of proteins in a structural context. *F1000Research* **6**, 1845.
24. Nagar, N., Ecker, N., Loewenthal, G., Avram, O., Ben-Meir, D., Biran, D., Ron, E., Pupko, T., (2021). Harnessing machine learning to unravel protein degradation in Escherichia coli. *MSystems.* **6**, e01296–e1320.
25. Adzhubei, I., Jordan, D.M., Sunyaev, S.R., (2013). Predicting functional effect of human missense mutations using PolyPhen-2. *Curr. Protoc. Hum. Genet.* **1**, 7–20.
26. Jumper, J., Evans, R., Pritzel, A., Green, T., Nature, M.F., et al., (2021). Highly accurate protein structure prediction with AlphaFold. *Nature* **596**, 583–589.
27. Wainreb, G., Wolf, L., Ashkenazy, H., Dehouck, Y., Ben-Tal, N., (2011). Protein stability: A single recorded mutation aids in predicting the effects of other mutations in the same amino acid site. *Bioinformatics* **27**, 3286–3292.
28. Jukes, T.H., Cantor, C.R., (1969). Evolution of protein molecules. In: Munro, H.N. (Ed.), *Mammalian Protein Metabolism*. Academic Press, New York, pp. 21–132.
29. Franzosa, E.A., Xia, Y., (2009). Structural determinants of protein evolution are context-sensitive at the residue level. *Mol. Biol. Evol.* **26**, 2387–2395.
30. Dean, A.M., Neuhauser, C., Grenier, E., Golding, G.B., (2002). The pattern of amino acid replacements in α/β-barrels. *Mol. Biol. Evol.* **19**, 1846–1864.
31. Amitai, G., Shemesh, A., Sitbon, E., Shklar, M., Netanely, D., Venger, I., Pietrokovski, S., (2004). Network analysis of protein structures identifies functional residues. *J. Mol. Biol.* **344**, 1135–1146.

32. Del Sol, A., O'Meara, P., (2005). Small-world network approach to identify key residues in protein-protein interaction. *Proteins Struct. Funct. Genet.* **58**, 672–682.
33. Brinda, K.V., Vishveshwara, S., (2005). A network representation of protein structures: Implications for protein stability. *Biophys. J.* **89**, 4159–4170.
34. Chakrabarty, B., Parekh, N., (2016). NAPS: Network analysis of protein structures. *Nucleic Acids Res.* **44**, W375–W382.
35. Pedregosa, F., Grisel, O., Weiss, R., Passos, A., Brucher, M., Varoquax, G., Gramfort, A., Michel, V., Thirion, B., Grisel, O., Blondel, M., Prettenhofer, P., Weiss, R., Dubourg, V., Brucher, M., (2011). Scikit-learn: Machine Learning in Python. *J. Mach. Learn. Res.* **12**, 2825–2830.
36. Berman, H.M., Westbrook, J., Feng, Z., Gilliland, G., Bhat, T.N., Weissig, H., Shindyalov, I.N., Bourne, P.E., (2000). The Protein Data Bank. *Nucleic Acids Res.* **28**, 235–242.
37. Goldenberg, O., Erez, E., Nimrod, G., Ben-Tal, N., (2009). The ConSurf-DB: Pre-calculated evolutionary conservation profiles of protein structures. *Nucleic Acids Res.* **37**, D323–D327.
38. Schittek, B., Hipfel, R., Sauer, B., Bauer, J., Kalbacher, H., Stevanovic, S., Schirle, M., Schroeder, K., Blin, N., Meier, F., Rassner, G., Garbe, C., (2001). Dermcidin: A novel human antibiotic peptide secreted by sweat glands. *Nat. Immunol.* **2**, 1133–1137.
39. Paulmann, M., Arnold, T., Linke, D., Özdirekcan, S., Kopp, A., Gutsmann, T., Kalbacher, H., Wanke, I., Schuenemann, V.J., Habeck, M., Bürck, J., Ulrich, A.S., Schittek, B., (2012). Structure-activity analysis of the dermcidin-derived peptide DCD-1L, an anionic antimicrobial peptide present in human sweat. *J. Biol. Chem.* **287**, 8434–8443.
40. Song, C., Weichbrodt, C., Salnikov, E.S., Dynowski, M., Forsberg, B.O., Bechinger, B., Steinem, C., De Groot, B.L., Zachariae, U., Zeth, K., (2013). Crystal structure and functional mechanism of a human antimicrobial membrane channel. *Proc. Natl. Acad. Sci. U. S. A.* **110**, 4586–4591.
41. Shoichet, B.K., Baase, W.A., Kuroki, R., Matthews, B.W., (1995). A relationship between protein stability and protein function. *Proc. Natl. Acad. Sci. U. S. A.* **92**, 452–456.
42. Kuroki, R., Weaver, L.H., Matthews, B.W., (1993). A covalent enzyme-substrate intermediate with saccharide distortion in a mutant T4 lysozyme. *Science* **262**, 2030–2033.
43. Larsen, T.M., Wedekind, J.E., Rayment, I., Reed, G.H., (1996). A carboxylate oxygen of the substrate bridges the magnesium ions at the active site of enolase: Structure of the yeast enzyme complexed with the equilibrium mixture of 2-phosphoglycerate and phosphoenolpyruvate at 1.8 Å resolution. *Biochemistry* **35**, 4349–4358.
44. Krissinel, E., Henrick, K., (2007). Inference of macromolecular assemblies from crystalline state. *J. Mol. Biol.* **372**, 774–797.
45. Starr, T.N., Greaney, A.J., Hilton, S.K., Ellis, D., Crawford, K.H.D., Dingens, A.S., Navarro, M.J., Bowen, J.E., Tortorici, M.A., Walls, A.C., King, N.P., Veesler, D., Bloom, J.D., (2020). Deep mutational scanning of SARS-CoV-2 receptor binding domain reveals constraints on folding and ACE2 binding. *Cell* **182**, 1295–1310.e20.
46. Kemp, S.A., Collier, D.A., Datir, R.P., ATM Ferreira, I., Jahun, A., Hosmillo, M., Rees-Spear, C., Mlcochova, P., Ushiro, I., Roberts, D.J., Chandra, A., Temperton, N., Blane, E., Modis, Y., Leigh, K., Briggs, J., van Gils, M., Smith, K.G., Bradley, J.R., Smith, C., Doffinger, R., Ceron-, L., Barcenas-Morales, G., Pollock, D.D., Goldstein, R.A., Skittrall, J.P., Gouliouris, T., Goodfellow, I.G., Illingworth, C.J., McCoy, L.E., Gupta, R.K., (2021). SARS-CoV-2 evolution during treatment of chronic infection. *Nature.* **592**, 277–282.
47. Li, Q., Wu, J., Nie, J., Zhang, L., Hao, H., Liu, S., Zhao, C., Zhang, Q., Liu, H., Nie, L., Qin, H., Wang, M., Lu, Q., Li, X., Sun, Q., Liu, J., Zhang, L., Li, X., Huang, W., Wang, Y., (2020). The impact of mutations in SARS-CoV-2 spike on viral infectivity and antigenicity. *Cell* **182**, 1284–1294.
48. Cao, Y., Wang, J., Jian, F., Xiao, T., Song, W., Yisimayi, A., Huang, W., Li, Q., Wang, P., An, R., Wang, J., Wang, Y., Niu, X., Yang, S., Liang, H., Sun, H., Li, T., Yu, Y., Cui, Q., Liu, S., Yang, X., Du, S., Zhang, Z., Hao, X., Shao, F., Jin, R., Wang, X., Xiao, J., Wang, Y., Xie, X.S., (2021). Omicron escapes the majority of existing SARS-CoV-2 neutralizing antibodies. *Nature*, 1–9.
49. Wang, P., Nair, M.S., Liu, L., et al., (2021). Antibody resistance of SARS-CoV-2 variants B. 1.351 and B. 1.1. 7. *Nature* **593**, 130–135.
50. Rubinstein, N., Pupko, T., (2012). Detection and analysis of conservation at synonymous sites. *Codon Evol. Mech. Model.*, 218–228.
51. Choi, S.C., Hobolth, A., Robinson, D.M., Kishino, H., Thorne, J.L., (2007). Quantifying the impact of protein tertiary structure on molecular evolution. *Mol. Biol. Evol.* **24**, 1769–1782.
52. Kleinman, C.L., Rodrigue, N., Lartillot, N., Philippe, H., (2010). Statistical potentials for improved structurally constrained evolutionary models. *Mol. Biol. Evol.* **27**, 1546–1560.
