# Supplementary material for: PDBx/mmCIF Ecosystem: Foundational Semantic Tools for Structural Biology
Source: J Mol Biol. Author manuscript; Available in PMC 2023 Jun 26. (PMC10292674; doi:10.1016/j.jmb.2022.167599)
Supplement: Article [file NIHMS1907597-supplement-Article.zip › FTMove--A-Web-Server-for-Detection-and-Analysis-of-Cryptic_2022_Journal-of-M.pdf]

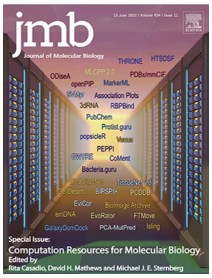

FTMove: A Web Server for Detection and Analysis of Cryptic and Allosteric Binding Sites by Mapping Multiple Protein Structures

Megan Egbert [1], George Jones [2], Matthew R. Collins [1], Dima Kozakov [2] and Sandor Vajda [1,3*]

1 - ***Department of Biomedical Engineering,*** *Boston University, Boston, MA 02215, USA*
2 - ***Department of Applied Mathematics and Statistics,*** *Stony Brook University, Stony Brook, NY 11794, USA*
3 - ***Department of Chemistry,*** *Boston University, Boston, MA 02215, USA*

Correspondence to Sandor Vajda: Department of Biomedical Engineering, Boston University, 44 Cummington Mall, Boston, MA 02215, USA. *vajda@bu.edu (S. Vajda)*
https://doi.org/10.1016/j.jmb.2022.167587
Edited by Rita Casadio

Abstract

Protein mapping distributes many copies of different molecular probes on the surface of a target protein in order to determine binding hot spots, regions that are highly preferable for ligand binding. While mapping of X-ray structures by the FTMap server is inherently static, this limitation can be overcome by the simultaneous analysis of multiple structures of the protein. FTMove is an automated web server that implements this approach. From the input of a target protein, by PDB code, the server identifies all structures of the protein available in the PDB, runs mapping on them, and combines the results to form binding hot spots and binding sites. The user may also upload their own protein structures, bypassing the PDB search for similar structures. Output of the server consists of the consensus binding sites and the individual mapping results for each structure - including the number of probes located in each binding site, for each structure. This level of detail allows the users to investigate how the strength of a binding site relates to the protein conformation, other binding sites, and the presence of ligands or mutations. In addition, the structures are clustered on the basis of their binding properties. The use of FTMove is demonstrated by application to 22 proteins with known allosteric binding sites; the orthosteric and allosteric binding sites were identified in all but one case, and the sites were typically ranked among the top five. The FTMove server is publicly available at https://ftmove.bu.edu.

© 2022 Elsevier Ltd. All rights reserved.

Introduction

Detection of binding sites on proteins has long been an important area of research for structure-based drug development. We have previously published a series of methods, the FTMap family of web servers, that identify important binding regions on proteins from a static X-ray structure.[1] The servers are based on a computational analog of the experimental method called multiple solvent crystal structures (MSCS),[2] where different types of small molecule probes are co-crystalized with a protein and then overlaid to determine the hot spots, defined as regions of the protein that contribute disproportionately to the binding free energy.[2,3] This method is performed computationally in FTMap by distributing millions of copies of 16 different small-molecule probe types around the protein followed by minimization and clustering of the probes, and then further clustering of the low-energy probe clusters to create 'consensus sites' (CSs) that represent the binding hot spots.[3] The consensus sites are in

0022-2836/© 2022 Elsevier Ltd. All rights reserved.

accordance with binding sites determined by experimental methods, and they are ranked by the number of probe clusters included in each site, where the strength of each site is linked to the energetic importance of the site with regards to druggability.[4–11] The FTSite server joins adjacent consensus sites predicted by FTMap to create the more commonly described binding sites.[1,12]

While FTMap has proven to be very successful in identifying the active site, identification of allosteric and cryptic sites tends to be more structure-dependent, in the sense that these sites may be present in some structures and absent in others.[13–16] For example, some cryptic sites are typically absent or occluded in ligand-free structures, but present in ligand-bound structures.[16,17] Thus, analysis of FTMap results for an ensemble of conformations will yield a more complete picture of a protein's binding map compared to FTMap results for a single snapshot of the structure. We have demonstrated this approach by developing an atlas of all allosteric sites in kinases.[18] It was shown that any specific site may occur in some structures of a kinase but not in others, and determining which sites occur in many structures provided valuable information.[18] More generally, identification of cryptic allosteric sites requires the mapping of multiple structures. One possible approach to solving this problem is the use of methods that merge molecular dynamics (MD) simulation with probe mapping, as in MixMD,[19,20] SILCS,[21,22] and MDMix.[23] These methods are based on MD simulation of the protein in a mixture of water and a few types of probe molecules. Binding hot spots are determined by the probe distribution at the end of the simulations. In a recent paper,[19] MixMD was used to identify cryptic-allosteric binding regions in seven proteins, most of them kinases. In contrast, FTMap of the initial (static) protein conformations showed no hot spots in the allosteric regions. However, methods such as MixMD[19,20] and SILCS[21,22] are not publicly available, and while MDMix[23] is available for download, setting up the calculation requires some efforts, and the program must be run with your own computational resources. All MD-based methods are computationally expensive, and the MD-component often requires target-specific parameterization and tailored protocols, making these approaches difficult to apply to a large, diverse set of targets. We note that the server FTDyn was created to identify general binding sites from thousands of MD structures, by running a simplified probe mapping on each structure (i.e., no locally minimized probes). However, such general binding site information lacks the detail needed to understand how conformational changes impact binding properties, and has been designed to be applicable to MD output only. In addition, these methods do not show whether the presence of a ligand may be required for the opening of a binding site, or whether ligand binding at an allosteric binding site affects the active site. More generally, modeling large conformational transitions using MD may be difficult.[24] To this end, a binding site detection program that considers multiple conformations of a given protein structure and reports on the varying strengths of the binding sites is desirable.

Mechanisms of cryptic site opening and allosteric regulation are typically elucidated on a case-by-case basis, by studying the conformational changes between two or a few X-ray protein structures. However, for many widely studied proteins there are often hundreds of structures deposited in the Protein Data Bank (PDB). As a result, there is an untapped opportunity to study binding site dynamics utilizing the multitude of protein conformers deposited in the PDB. We present a publicly available web server, FTMove (https://ftmove.bu.edu), that determines the protein binding sites considering all known conformers. The binding sites are determined from FTMap results for each protein conformation. To utilize the PDB conformer search, users may submit a single PDB code and chain of their protein of interest, and the server will run a sequence-based search to identify all conformers in the PDB. When experimental data is lacking, the server allows users to submit their own ensemble of protein conformations. This allows users to submit specific conformations for analysis, clustered snapshots from a molecular dynamics simulation, or even multiple predicted protein structures, as recent advances in protein structure prediction emphasize the potential of analyzing surface properties from protein models.[25] Output of the server includes the consensus binding sites, mapping of individual conformers, the binding site strengths for each conformer, and clustering of the structures on the basis of their binding site strengths. Taken together, this information permits the user to assess how binding sites open, close, and interact with other sites and ligands. Naturally, this tool will also detect active and orthosteric sites, but the primary motivation is improving the detections of challenging cryptic and allosteric sites. We also note that FTMove is not intended to replace MD-based mapping methods, but it provides a complementary and computationally less expensive approach.

Methods

Overall workflow

The purpose of the FTMove web server is to apply FTMap to an ensemble of protein conformations, and identify binding sites that consistently appear across multiple different conformations. A high-level overview of the FTMove algorithm is depicted in Figure 1. In summary, a protein structure is input to the server, and all known

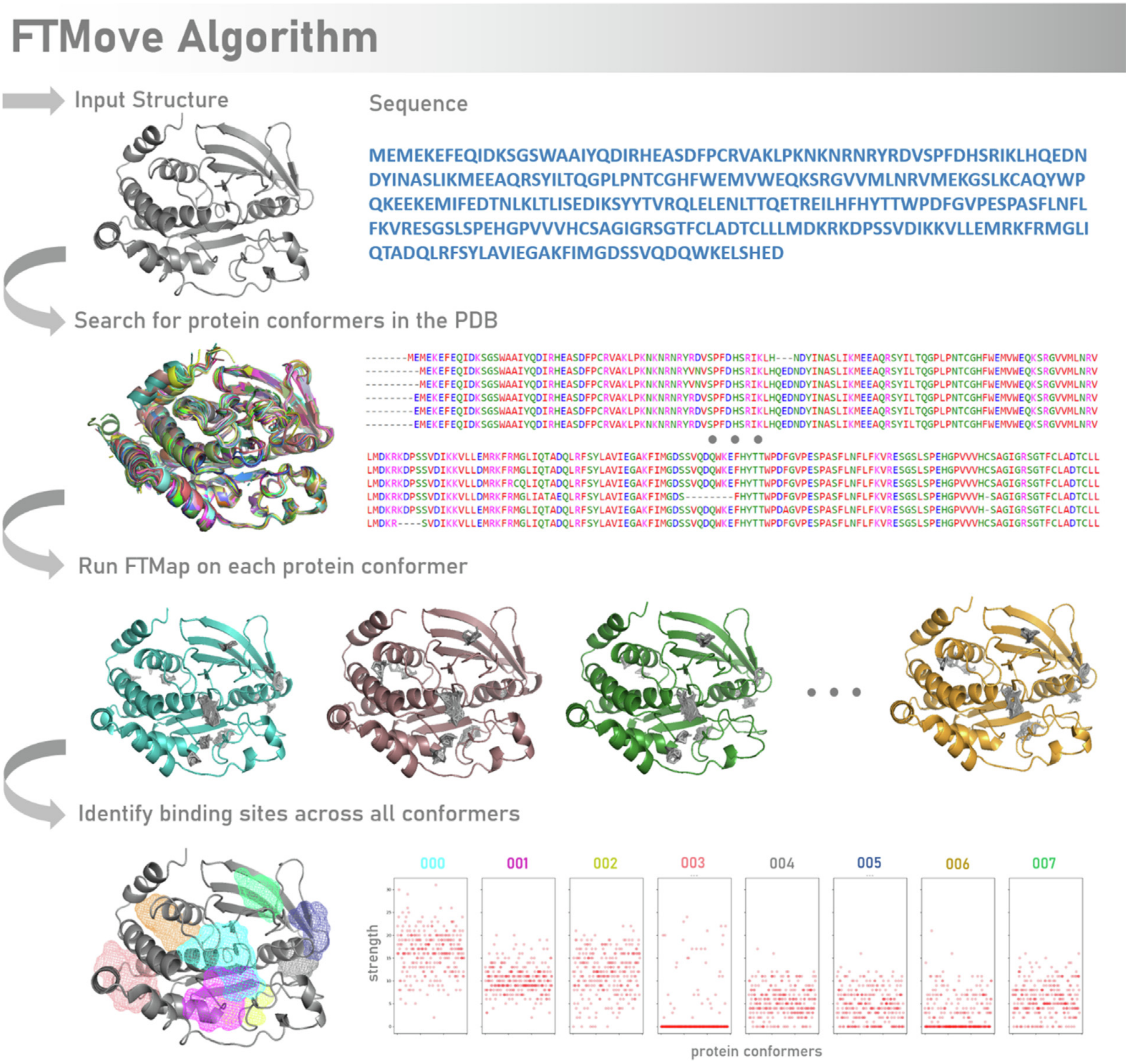

Figure 1. High-level workflow of FTMove algorithm, starting with a PDB ID input from the user. The sequence of the structure is extracted from the PDB website, and similar structures are identified by searching the sequence against the PDB database. Each structure identified is mapped by FTMap. The resultant consensus sites, representing the binding hot spots, for each structure are clustered together using FTMove's clustering algorithm to identify the binding sites. Finally, the binding sites are ranked according to their strength across all structures and output to the user.

structures of the same protein in the PDB are identified. FTMap is run on each protein structure, and the resulting hot spots for each conformer are locally-aligned to the input structure. The hot spots are then clustered and joined together to create the binding sites, which are ranked according to their strength across all structures. The binding sites and the number of FTMap probe clusters identified within each binding site are output to the user for evaluation. Details of the algorithm implemented in FTMove are described in the supplementary information.

Selection of protein structures

For input in the form of a PDB code and chain, the sequence of the input structure is extracted from the PDB-provided FASTA sequence, for the specific chain entered.[26] Related protein structures representing the available conformations are identified by running a sequence search (via MMseqs2) on all structures deposited in the PDB and selecting only those with sequence identity $\geq$90% and sequence coverage $\geq$90% (and $\leq$120%, where this second threshold is provided to avoid adding

domains that are not part of the starting target protein).[27] Alternatively, an ensemble of protein conformations may be directly uploaded to the web server, as structural files with a 'pdb' extension, allowing the user to specifically select which structures are considered conformers, or analyze, for example, a molecular-dynamics ensemble of structures. For this use-case, all uploaded structures must have the same chain designation. Finally, the user may select to domain split the structures prior to mapping, which implies that both the entire protein and its separate domains will be mapped. If this option is selected, the domains of each protein structure are determined by the Protein Domain Parser,[28] see the supplementary information for additional details.

Mapping multiple protein structures

Each of the protein structures is mapped using the algorithm described by FTMap.[3] First, all bound ligands and water molecules are removed. Next, rigid body docking of probe molecules is performed - this step is repeated for 16 different small-molecule probe types.[3] For each probe type, millions of different probe placements are evaluated using an energy function that includes the van der Waals energy (both attractive and repulsive contributions), electrostatic interaction energy, a hydrophobic cavity term, and a statistical knowledge-based pairwise potential representing solvation effects.[3] The 2,000 lowest energy probes are retained for minimization using the CHARMM potential with a continuum electrostatic term.[29] Next, a greedy algorithm is used to cluster the individual probe structures, starting with the lowest-energy probe structure and including all same-type probe structures within 3 Å. For each probe type a number of low energy clusters are retained. To create the consensus sites representative of the binding hot spots, clusters of different probe types are clustered again, this time using a slightly loosened threshold of 4 Å to cluster the probe clusters.[1]

Identification of binding sites

The binding sites are created by clustering the consensus sites output from FTMap for each protein conformer. Because the number of probe clusters (PC) in each consensus site is related to the energetic importance of the binding region, once the binding sites are determined, the total number of proximal locally-aligned probe clusters are used to rank the binding sites.[12]

The steps taken to create the binding sites in FTMove are outlined in Figure S1. First, the consensus sites from each protein structure are locally aligned to the reference structure with PyMOL, using the residues within 5 Å of the consensus site.[30] The reference structure is taken as the PDB input to the server, or the first structure uploaded in the case up multiple structures uploaded. Corresponding residues in the reference structure are determined from multiple sequence alignment with Clustal Omega.[31,32] Next, the locally-aligned consensus sites from all structures are clustered together using a greedy algorithm, starting with the strongest consensus site and including all sites within a 4 Å radius, to create 'clustered sites'. Overlapping clustered sites are then joined together to create the final binding sites, with special consideration to large sites with surface area >1000 Å^2. For large binding sites, stricter joining of the clustered sites is employed – this is especially important for proteins with many binding sites concentrated in a small area, such as kinases and the ATP/DFG binding region, where it is useful to designate the sites separately. Details of the joining protocol are provided in the supplementary information. Finally, the total number of probe clusters in each binding site are calculated for each structure, and these values are used to rank the binding sites. Ranking is based on equal weights of: the average number of probe clusters in the site (all structures), the average number of probe clusters in the site (considering only structures that have bound probe clusters in the site), and the maximum number of probe clusters in the site. This ranking gives representation to strong binding sites that are only present in some fraction of the structures.

Clustering of structures based on mapping profiles

The structures considered by FTMove can be clustered on the basis of their binding properties, allowing the user to identify structures with similar binding properties. We perform hierarchical clustering of the structures based on the correlation of the mapping profiles. Each structure's mapping profile is defined as the number of probe clusters within 2 Å of each binding site identified. Hierarchical clusters are formed based on the average correlation, using the seaborn clustermap feature, and the resulting heatmap image is output to the user once the FTMove job is complete.[33]

Output to the user

FTMove outputs all binding sites identified for the protein, in both a pymol session file and as individual structure files ('pdb' extension). Furthermore, for each structure, the strength of each binding site, represented as the number of probe clusters within 2 Å of the binding site, is also reported in a comma-separated-file (CSV). This structure-strength information indicates which conformers have binding sites detected, and to what degree, allowing users to understand the impacts of conformational changes.

Web server functionality

The FTMove web server is available at https://ftmove.bu.edu. It can be used with an account to ensure confidentiality of the job submissions, or without an account with results available to the public. Input to the server can be in one of two forms as follows. (1) Enter the 'PDB ID' and 'PDB chain ID' of the subject protein. For example, for the protein PTP1B, one possible input is 'PDB ID' = 2F70 and 'PDB Chain ID' = A. (2) Upload a set of protein structures with the file extension '.pdb', and enter the corresponding 'Chain ID' (all uploaded structures must have the same chain ID). After submission, the job status will progress from 'Preparing Structures', to 'Running FTMap on Structures', to 'Evaluating Binding Sites'. Once the job is complete, users will be able to view the binding sites overlaid on their input structure in the job details web page, and will be able to download a PyMOL session with the binding sites and individual FTMap results for each conformer. The binding site strength information for each conformer will also be available to view in a table in the web page, and for download as a CSV table. Additionally, the hierarchical clustered heatmap of the structures, based on the mapping profiles correlation, is available for download. To answer any questions regarding job submission and analysis of results, we provide a 'Help' page with detailed instructions, and an 'Examples' page with results of the 22 cases discussed in this paper. Finally, a contact email is provided for any further questions or comments.

The computational cost of each job run on FTMove is dependent on the number of structures mapped; we estimate 16 core-hours per structure mapped, plus an additional 28 core hours to cover preparation of structures and evaluation of the consensus binding sites. We note the FTMove web server maps structures in parallel, and therefore the time it takes to complete a job depends greatly on the availability of the shared computing cluster used to process the jobs. Typically, jobs with less than 50 structures will complete within 24 hours, whereas jobs with upwards of 300 structures may take a couple days to complete. However, this is the worst case scenario. The server runs on the Boston University Shared Computing Cluster (SCC), which includes 21,000 CPU cores, and in most cases due to the high number of CPU cores the execution time does not grow linearly with the number of structures, and execution times can be much shorter. In addition, if the user is registered, an e-mail will be sent when the job is completed.

Results

Targets with allosteric sites

Proteins with known allosteric binding sites were selected to demonstrate the FTMove algorithm. The proteins were extracted from a number of allostery focused publications[34,35] and the Allosteric Site Database.[36] We limited our selection to targets with known orthosteric and allosteric binding sites. At this time FTMove is limited to single chain PDB input, so the analysis was restricted to targets where mapping of only one chain was needed to identify the binding sites. Proteins that bind DNA were also excluded. Based on our selection criteria, we selected 22 proteins with known orthosteric and allosteric ligands for assessment of the FTMove methodology, see Table 1. It is worth noting that all seven proteins tested by the MixMD method[19] (PDK1 Kinase, Glucokinase, PTP1B, CHK1 Kinase, ABL1 Kinase, Farnesyl Pyrophosphate Synthase (FPPS), and Androgen Receptor) are included in our set of 22 allosteric binding sites. Finally, the PDB code submitted to FTMove for each protein was selected to represent a structure with no bound allosteric ligand.

FTMove identified both the orthosteric and allosteric binding sites in all proteins selected, apart from the Androgen Receptor allosteric binding site, see Table 1 and Figure S2. Notably, this shows that FTMove is able to identify the allosteric binding sites for all MixMD targets, apart from the Androgen Receptor (details are provided in the supplementary information). Furthermore, for the overwhelming majority of proteins (18/22), the orthosteric ligand binding site is the strongest site identified by FTMove (Site 000), and the allosteric ligand binding site is ranked within the top five for 19/22 targets. The number of structures for individual targets ranged from 14 to 298, with 12 proteins having more than 100 structures deposited in the PDB.

FTMove outputs the binding site strengths for each structure, represented as the number of probe clusters (from FTMap consensus sites) overlapping with the binding site. The usefulness of this information is exemplified in proteins with conformational changes that impact the binding site strengths. For example, kinases have a highly conserved Asp-Phe-Glu (DFG) motif that flips between the DFG-in and DFG-out conformations and impacts the activity of the protein and formation of the so-called DFG back pocket. For insulin-like growth factor-1 receptor (IFG-1R), there are 59 structures deposited in the PDB (including all chain variations). Using the DFG-classification provided by Modi and Dunbrack,[37] 50 are classified as DFG-in, four as DFG-inter, and five as DFG-out. Within each class, Modi and Dunbrack further classify the structures based on

Table 1 FTMove Binding Site Predictions for 22 proteins with known allosteric binding sites.

Protein	Active Site[a]	Allosteric Site[a]	Regulation Type	FTMove Input	Domain Split?	Number of Structures	Total Number of Sites	Active Site Rank[b]	Allosteric Site Rank[b]
DAHP Synthase	1KFL_A_PEP	1KFL_A_PHE	Inhibitory	1GG1_A	No	24	7	002	000
PDK1	3HRF_A_ATP	3HRF_A_P47	Activator	1H1W_A	Yes	79	19	000	002
	3HRF_A_ATP	3NAX_A_MP7	Inhibitory					000	005
Fructose 1,6-bisphosphate	1FBP_A_F6P	1FBP_A_AMP	Inhibitory	2FBP_A	No	114	10	000	002
	1FBP_A_F6P	1KZ8_A_PFE	Inhibitory					000	004
Glycogen Phosphorylase	1BX3_A_PLP	1Z6Q_A_195	Inhibitory	1BX3_A	Yes	254	23	003	007
p38 Alpha Kinase	3ZSH_A_469	1KV1_A_BMU	Inhibitory	1WBO_A	No	298	15	000	001
	3ZSH_A_469	4E6A_A_0O9	Activator					000	005
MEK1	3EQI_A_ADP	3PP1_A_IZG	Inhibitory	3EQI_A	Yes	66	25	003	000
Glucokinase	1V4S_A_GLC	1V4S_A_MRK	Activator	3IDH_A	No	33	11	002	000
Kinesin	1Q0B_A_ADP	1Q0B_A_NAT	Inhibitory	3HQD_A	No	79	11	002	000
	1Q0B_A_ADP	3ZCW_A_4A2[c]	Inhibitory					002	003
PTP1B	2F70_A_UN6	1T48_A_BB3	Inhibitory	2F70_A	No	290	17	000/002	010
TEM-1 beta-lactamase	1ERQ_A_BJH	1PZO_A_CBT[d]	Inhibitory	1JWP_A	No	150	14	000	003
CHK1	2E9N_A_76A	3JVS_A_AGY	Inhibitory	2E9N_A	Yes	137	16	001	002
ABL1	2G2I_A_ADP	3K5V_B_STJ	Inhibitory	2G2I_A	Yes	109	25	000	015
SRC	3F6X_A_IHH	3G6G_A_G6G	Inhibitory	3F6X_A	No	157	12	000	000/001
Tryptophan-tRNA ligase	1MAU_A_LTN	1MAU_A_ATP	Inhibitory	1D2R_A	No	52	8	000	000/003
Acetylcholinesterase	2C5F_A_NWA	2C5F_A_CHH	Inhibitory	1AMN_A	No	140	6	000	000
IGF-1 Receptor	1JQH_A_ANP	3LW0_A_CCX[e]	Inhibitory	1JQH_A	No	59	11	000	001
B-RAF	4E26_A_734	3IDP_B_L1E	Inhibitory	4H58_B	No	164	10	000	000
Monoamine oxidase B	2XCG_B_FA8	2XCG_B_XCG	Inhibitory	1GOS_A	No	102	6	000/004	001
PDK2	2BU2_A_ATP	2BU2_A_TF1	Inhibitory	5J71_A	Yes	33	22	000/002	001
Glutamate Racemase	2JFZ_A_DGL	2JFZ_A_003	Inhibitory	2JFY_A	No	14	8	005	001
FPPS	4DEM_F_YS4	3N5J_A_G01[f]	Inhibitory	4DEM_F	No	91	7	000	003
Androgen Receptor	2AM9_A_TES	2PIX_A_FLF	Inhibitory	2AM9_A	No	102	5	000	—

[a] PDB ID_Chain_Ligand ID.
[b] Rank of the site according to the FTMap convention (sites 000, 001, etc.; starting with the strongest site).
[c] The allosteric ligand 3CZW_A_4A2 binds in two locations on chain A; the strongest binding location, formed by helices α4 and α6, is referenced here.
[d] The allosteric ligand 1PZO_A_CBT binds in two places on chain A; both are encapsulated by site 003.
[e] The allosteric ligand 3LW0_A_CCX binds in two places on chain A; the site referenced here is adjacent to the ATP binding site and is reported to be allosteric.
[f] The allosteric ligand 3N5J_A_G01 binds in two places; the site referenced here is closest to the active site and is reported to be allosteric.

the angles of the DFG-loop and pre-curser residue.[37] For IGF-1R, within the DFG-in class, 24 are clustered as ABAminus, 3 are BLAminus, and the remaining 23 are noise.[37] Considering all 59 structures, FTMove identified the ATP binding site as the strongest site (numbered as 000 by the FTMap convention),[1] and the DFG-back pocket as the second strongest site (001), see Figure 2(A). The strength of the DFG back pocket site (001) is impacted by the orientation of the DFG-motif, where unsurprisingly the site is strongest in the DFG-out structures (on the average, 21 probe clusters). Among the DFG-in structures, a range of strengths are reported, with the weakest strength reported in the DFG-in ABAminus class (0 probe clusters), see Figure 2(B).

While the conformational shift of the DFG-motif (and resulting binding site impacts) is well known in kinases, we sought to more generally understand how varying conformations of a protein impact binding site strength. To this end, FTMove performs hierarchical clustering on all structures of the protein according to the binding site strengths for each structure, referred to here as the binding fingerprints. For IGF-1R, the DFG-classifications mentioned above can generally be observed within the hierarchical clustering heatmap, see Figure 2(C). For example, most DFG-in ABAminus structures clustered together; they all have fairly strong probe representation in the ATP binding site (000) and weak probe presence in all other binding sites. The DFG-inter and DFG-out structures also cluster together, and are slightly distinguished from each other by their probe counts in sites 001, 002, and 005. However, the binding profiles of the DFG-in noise structures vary significantly. Some DFG-in noise structures have binding profiles similar to DFG-out structures, and some have properties similar to DFG-in ABAminus structures. This is not surprising based on the bimodal distribution of strengths in the DFG back pocket (001) for this class, as shown in Figure 2(B). Interestingly, one group of DFG-in noise structures (PDB 5HZN, chains A–H) is distinguished from both the DFG-out and DFG-in ABAminus classes by their probe counts in site 004, a site not observed in other IGF-1R structures. This site is present in the 5HZN structures because the activation loop is shifted towards the C-helix, causing probes to congregate in this region and create binding site 004 (see Figure S4).

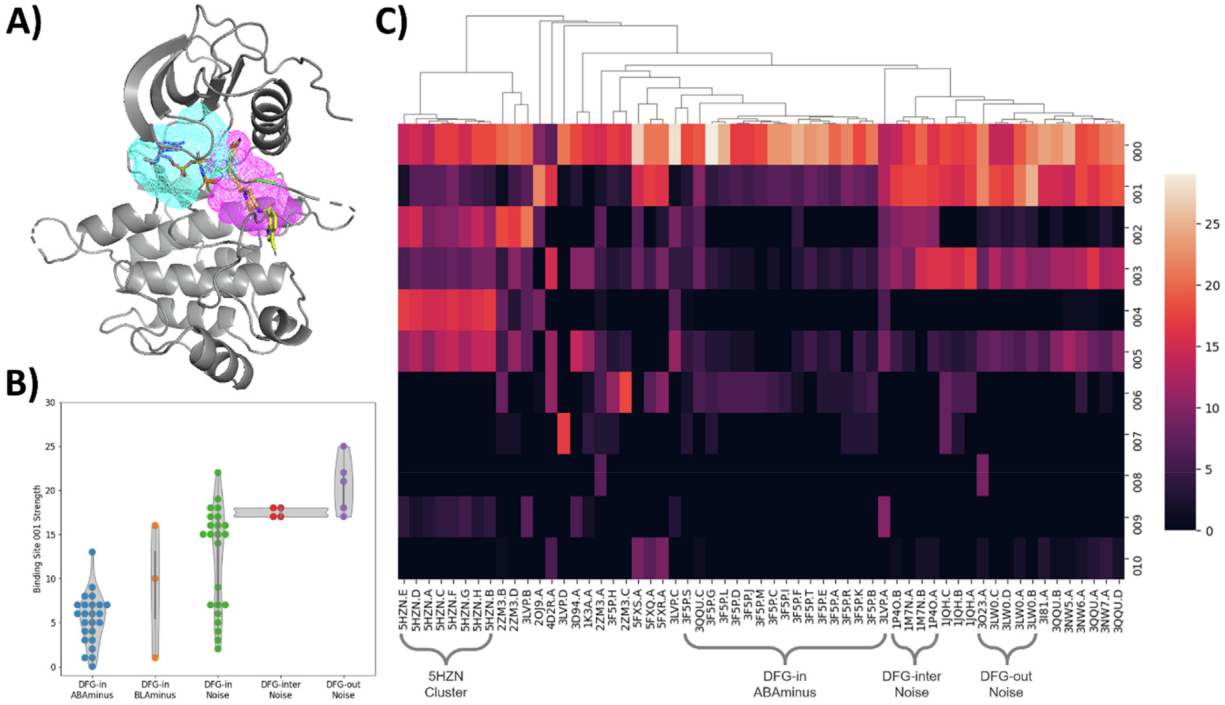

Figure 2. Binding Sites Strengths of IGF-1R conformations, distinguished by orientations of the DFG-motif. (A) The structure of IGF-1R, represented by PDB 1JQH chain A, with ANP bound (blue sticks), and allosteric ligand CCX (3LW0 chain A) overlaid (yellow sticks). The ATP binding site (000 – cyan) and DFG-back pocket site (001 – magenta) identified by FTMove are overlaid in mesh. The DFG-motif is colored green. (B) Strength of the DFG back pocket site (001) in IGF-1R conformations for each DFG-classification. Strength is represented as the number of probe clusters within 2 Å of the binding site. The violin plot is represented in light gray, with a swarmplot overlay to show individual structure-strength values. (C) Clustered heatmap of IGF-1R structures, based on correlation of FTMove binding profiles. Heatmap indicates the number of probe clusters within 2 Å of the binding site, ranging from no clusters (black) to 30 clusters (white). Groups of structures discussed in the text are indicated.

Targets with cryptic sites

The allosteric binding sites are cryptic binding sites in the proteins PTP1B (1T48_A_BB3), PDK2 (2BU2_A_TF1), and TEM β-lactamase (1PZO_A_CBT).[14] In all three of these cases, the cryptic/allosteric binding sites are only identified in a fraction of structures, consistent with the notion of a cryptic site. For example, in PDK2 the cryptic-allosteric site is identified by FTMove as site 001. Only 33 structures are identified in the PDB for PDK2, and about half have no probes in site 001, while the other half have strong probe representation in the site. The strength of the cryptic site appears to be strongly linked to the orientation of the proximal phenylalanine residue, which acts as a lid opening and closing the site.[38] For example, in PDB structure 2BU2 chain A, the Phe lid is open, and the cryptic site (001) is strong with 34 probe clusters. However, in structure 5J6A chain A, the Phe lid is closed and the pocket does not appear, thus no probe clusters are identified in the cryptic site (001), see Figure S5. Cryptic site availability impacts the hierarchical clustering of the PDK2 structures, as structures with no probes found in site 001 are clustered and distinguished from structures with many probes in the cryptic site (see Figure S5).

Conclusion

The advantage of predicting binding sites on an ensemble of protein conformations, rather than a single structure, is elucidation of transient binding sites. The FTMove web server achieves this goal by utilizing a previously-established binding hot spot detection algorithm, FTMap, to evaluate binding properties across many conformations of a protein. The server outputs conformation-specific strengths of binding sites in each structure, allowing users to investigate potential mechanisms of opening cryptic allosteric binding sites. Utilizing the multitude of protein structures deposited in the PDB, we have demonstrated the server's ability to identify orthosteric and allosteric binding sites in 22 proteins. Furthermore, we have demonstrated that clustering of the structures based on their binding site profiles is able to group the structures by allosteric and cryptic site availability. Beyond assessing binding sites in experimentally determined protein structures in the PDB, the server is also able to analyze an ensemble of snapshots from molecular dynamics simulations, including predictive models of proteins, permitting detailed analysis of binding site dynamics in a multitude of protein structures.

CRediT authorship contribution statement

Megan Egbert: Conceptualization, Methodology, Software, Investigation, Writing – original draft. **George Jones:** Software, Investigation. **Matthew Collins:** Investigation. **Dima Kozakov:** Investigation. **Sandor Vajda:** Conceptualization, Methodology, Investigation, Writing – original draft.

Acknowledgements

This investigation was supported by grants R35GM118078, R21GM127952, and RM1135136 from the National Institute of General Medical Sciences.

Declaration of competing interest

The authors declare that they have no known competing financial interests or personal relationships that could have appeared to influence the work reported in this paper.

Appendix A. Supplementary material

Supplementary data to this article can be found online at https://doi.org/10.1016/j.jmb.2022.167587.

Received 11 December 2021;
Accepted 7 April 2022;
Available online 18 April 2022

Keywords:
protein binding site;
allosteric binding;
drugability;
binding hot spot;
protein mapping

References

1. Kozakov, D., Grove, L.E., Hall, D.R., Bohnuud, T., Mottarella, S.E., Luo, L., Xia, B., Beglov, D., et al., (2015). The FTMap family of web servers for determining and characterizing ligand-binding hot spots of proteins. *Nat. Protoc.* **10**, 733–755.
2. Mattos, C., Ringe, D., (1996). Locating and characterizing binding sites on proteins. *Nat. Biotechnol.* **14**, 595–599.
3. Brenke, R., Kozakov, D., Chuang, G.-Y., Beglov, D., Hall, D., Landon, M.R., Mattos, C., Vajda, S., (2009). Fragment-based identification of druggable 'hot spots' of proteins using Fourier domain correlation techniques. *Bioinformatics* **25**, 621–627.
4. Dennis, S., Kortvelyesi, T., Vajda, S., (2002). Computational mapping identifies the binding sites of organic solvents on proteins. *Proc. Natl. Acad. Sci. U. S. A.* **99**, 4290–4295.
5. Landon, M.R., Lieberman, R.L., Hoang, Q.Q., Ju, S., Caaveiro, J.M., Orwig, S.D., Kozakov, D., Brenke, R., et al., (2009). Detection of ligand binding hot spots on protein surfaces via fragment-based methods: application to DJ-1 and glucocerebrosidase. *J. Comput. Aided Mol. Des.* **23**, 491–500.

6. Landon, M.R., Lancia Jr., D.R., Yu, J., Thiel, S.C., Vajda, S., (2007). Identification of hot spots within druggable binding regions by computational solvent mapping of proteins. *J. Med. Chem.* **50**, 1231–1240.
7. Chuang, G.Y., Kozakov, D., Brenke, R., Beglov, D., Guarnieri, F., Vajda, S., (2009). Binding hot spots and amantadine orientation in the influenza a virus M2 proton channel. *Biophys. J.* **97**, 2846–2853.
8. Buhrman, G., O'Connor, C., Zerbe, B., Kearney, B.M., Napoleon, R., Kovrigina, E.A., Vajda, S., Kozakov, D., et al., (2011). Analysis of binding site hot spots on the surface of Ras GTPase. *J. Mol. Biol.* **413**, 773–789.
9. Zerbe, B.S., Hall, D.R., Vajda, S., Whitty, A., Kozakov, D., (2012). Relationship between Hot Spot Residues and Ligand Binding Hot Spots in Protein-Protein Interfaces. *J. Chem. Inf. Model.* **52**, 2236–2244.
10. Golden, M.S., Cote, S.M., Sayeg, M., Zerbe, B.S., Villar, E. A., Beglov, D., Sazinsky, S.L., Georgiadis, R.M., et al., (2013). Comprehensive experimental and computational analysis of binding energy hot spots at the NF-kappaB essential modulator/IKKbeta protein-protein interface. *J. Am. Chem. Soc.* **135**, 6242–6256.
11. Kozakov, D., Hall, D.R., Napoleon, R.L., Yueh, C., Whitty, A., Vajda, S., (2015). New Frontiers in Druggability. *J. Med. Chem.* **58**, 9063–9088.
12. Ngan, C.H., Hall, D.R., Zerbe, B., Grove, L.E., Kozakov, D., Vajda, S., (2012). FTSite: high accuracy detection of ligand binding sites on unbound protein structures. *Bioinformatics* **28**, 286–287.
13. Oleinikovas, V., Saladino, G., Cossins, B.P., Gervasio, F. L., (2016). Understanding Cryptic Pocket Formation in Protein Targets by Enhanced Sampling Simulations. *J. Am. Chem. Soc.* **138**, 14257–14263.
14. Cimermancic, P., Weinkam, P., Rettenmaier, T.J., Bichmann, L., Keedy, D.A., Woldeyes, R.A., Schneidman-Duhovny, D., Demerdash, O.N., et al., (2016). CryptoSite: Expanding the Druggable Proteome by Characterization and Prediction of Cryptic Binding Sites. *J. Mol. Biol.* **428**, 709–719.
15. Sun, Z., Wakefield, A.E., Kolossvary, I., Beglov, D., Vajda, S., (2020). Structure-Based Analysis of Cryptic-Site Opening. *Structure* **28** 223–235 e222.
16. Vajda, S., Beglov, D., Wakefield, A.E., Egbert, M., Whitty, A., (2018). Cryptic binding sites on proteins: definition, detection, and druggability. *Curr. Opin. Chem. Biol.* **44**, 1–8.
17. Beglov, D., Hall, D.R., Wakefield, A.E., Luo, L., Allen, K.N., Kozakov, D., Whitty, A., Vajda, S., (2018). Exploring the structural origins of cryptic sites on proteins. *Proc. Natl. Acad. Sci.* **115**, E3416–E3425.
18. Yueh, C., Rettenmaier, J., Xia, B., Hall, D.R., Alekseenko, A., Porter, K.A., Barkovich, K., Keseru, G., et al., (2019). Kinase Atlas: Druggability Analysis of Potential Allosteric Sites in Kinases. *J. Med. Chem.* **62**, 6512–6524.
19. Ghanakota, P., Carlson, H.A., (2016). Moving Beyond Active-Site Detection: MixMD Applied to Allosteric Systems. *J. Phys. Chem. B* **120**, 8685–8695.
20. Lexa, K.W., Carlson, H.A., (2013). Improving Protocols for Protein Mapping through Proper Comparison to Crystallography Data. *J. Chem. Inf. Model.* **53**, 391–402.
21. Yu, W., Lakkaraju, S.K., Raman, E.P., Mackerell Jr., A.D., (2014). Site-Identification by Ligand Competitive Saturation (SILCS) assisted pharmacophore modeling. *J. Comput. Aided Mol. Des.*.
22. Raman, E.P., Yu, W., Lakkaraju, S.K., MacKerell Jr., A.D., (2013). Inclusion of multiple fragment types in the site identification by ligand competitive saturation (SILCS) approach. *J. Chem. Inf. Model.* **53**, 3384–3398.
23. Alvarez-Garcia, D., Barril, X., (2014). Molecular simulations with solvent competition quantify water displaceability and provide accurate interaction maps of protein binding sites. *J. Med. Chem.* **57**, 8530–8539.
24. Echols, N., Milburn, D., Gerstein, M., (2003). MolMovDB: analysis and visualization of conformational change and structural flexibility. *Nucleic Acids Res.* **31**, 478–482.
25. Egbert, M., Ghani, U., Ashizawa, R., Kotelnikov, S., Nguyen, T., Desta, I., Hashemi, N., Padhorny, D., et al., (2021). Assessing the binding properties of CASP14 targets and models. *Proteins: Struct. Funct. Bioinf.*.
26. Berman, H., Henrick, K., Nakamura, H., Markley, J.L., (2007). The worldwide Protein Data Bank (wwPDB): ensuring a single, uniform archive of PDB data. *Nucleic Acids Res.* **35**, D301–D303.
27. Mirdita, M., Steinegger, M., Soding, J., (2019). MMseqs2 desktop and local web server app for fast, interactive sequence searches. *Bioinformatics* **35**, 2856–2858.
28. Alexandrov, N., Shindyalov, I., (2003). PDP: protein domain parser. *Bioinformatics* **19**, 429–430.
29. Brooks, B.R., Bruccoleri, R.E., Olafson, B.D., States, D.J., Swaminathan, S., Karplus, M., (1983). Charmm – a Program for Macromolecular Energy, Minimization, and Dynamics Calculations. *J. Comput. Chem.* **4**, 187–217.
30. Schrodinger, LLC, 2015.
31. Sievers, F., Higgins, D.G., (2018). Clustal Omega for making accurate alignments of many protein sequences. *Protein Sci.* **27**, 135–145.
32. Sievers, F., Wilm, A., Dineen, D., Gibson, T.J., Karplus, K., Li, W., Lopez, R., McWilliam, H., et al., (2011). Fast, scalable generation of high-quality protein multiple sequence alignments using Clustal Omega. *Mol. Syst. Biol.* **7**, 539.
33. Waskom, M.L., (2021). Seaborn: statistical data visualization. *J. Open Source Softw.* **6**, 3021.
34. Hardy, J.A., Wells, J.A., (2004). Searching for new allosteric sites in enzymes. *Curr. Opin. Struct. Biol.* **14**, 706–715.
35. Zorn, J.A., Wells, J.A., (2010). Turning enzymes ON with small molecules. *Nature Chem. Biol.* **6**, 179–188.
36. Shen, Q., Wang, G., Li, S., Liu, X., Lu, S., Chen, Z., Song, K., Yan, J., et al., (2016). ASD v3. 0: unraveling allosteric regulation with structural mechanisms and biological networks. *Nucleic Acids Res.* **44**, D527–D535.
37. Modi, V., Dunbrack Jr., R.L., (2019). Defining a new nomenclature for the structures of active and inactive kinases. *Proc. Natl. Acad. Sci. U. S. A.* **116**, 6818–6827.
38. Knoechel, T.R., Tucker, A.D., Robinson, C.M., Phillips, C., Taylor, W., Bungay, P.J., Kasten, S.A., Roche, T.E., et al., (2006). Regulatory roles of the N-terminal domain based on crystal structures of human pyruvate dehydrogenase kinase 2 containing physiological and synthetic ligands. *Biochemistry* **45**, 402–415.
