# Supplementary material for: PDBx/mmCIF Ecosystem: Foundational Semantic Tools for Structural Biology
Source: J Mol Biol. Author manuscript; Available in PMC 2023 Jun 26. (PMC10292674; doi:10.1016/j.jmb.2022.167599)
Supplement: Article [file NIHMS1907597-supplement-Article.zip › Visualizing-Cluster-specific-Genes-from-Single-cell-Tra_2022_Journal-of-Mole.pdf]

Research Article

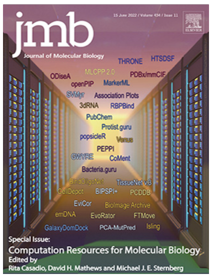

Visualizing Cluster-specific Genes from Single-cell Transcriptomics Data Using Association Plots

Elzbieta Gralinska, Clemens Kohl, Bita Sokhandan Fadakar and Martin Vingron *

Department of Computational Molecular Biology, *Max Planck Institute for Molecular Genetics, Berlin, Germany*

Correspondence to Martin Vingron: *vingron@molgen.mpg.de (M. Vingron), @ela_gralinska (E. Gralinska), @ClemensKohl (C. Kohl), @VingronLab (M. Vingron)*
https://doi.org/10.1016/j.jmb.2022.167525
Edited by David Mathews

Abstract

Visualizing single-cell transcriptomics data in an informative way is a major challenge in biological data analysis. Clustering of cells is a prominent analysis step and the results are usually visualized in a planar embedding of the cells using methods like PCA, t-SNE, or UMAP. Given a cluster of cells, one frequently searches for the genes highly expressed specifically in that cluster. At this point, visualization is usually replaced by studying a list of differentially expressed genes. Association Plots are derived from correspondence analysis and constitute a planar visualization of the features which characterize a given cluster of observations. We have adapted Association Plots to address the challenge of visualizing cluster-specific genes in large single-cell data sets. Our method is made available as a free R package called *APL*. We demonstrate the application of *APL* and Association Plots to single-cell RNA-seq data on two example data sets. First, we present how to delineate novel marker genes using Association Plots with the example of Peripheral Blood Mononuclear Cell data. Second, we show how to apply Association Plots for annotating cell clusters to known cell types using Association Plots and a predefined list of marker genes. To do this we will use data from the human cell atlas of fetal gene expression. Results from Association Plots will also be compared to methods for deriving differentially expressed genes, and we will show the integration of *APL* with Gene Ontology Enrichment.

© 2022 The Author(s). Published by Elsevier Ltd.

Introduction

A key step in single-cell (sc) RNA-seq data analysis is clustering of cells. Two goals motivate clustering. Firstly, one is interested in the cell types represented in the experiment, and, secondly, one wants to determine which genes characterize a particular cell cluster. While there are many clustering algorithms readily available today, exploration of marker genes which characterize a cluster may still require tedious sifting through long lists of program output. We will here present a technique for determining and visualizing cluster-specific genes for given clusters of cells.

The terms ‘marker genes’ or ‘cluster-specific genes’ refer to genes with expression profiles characteristic for a given cell type or a cell cluster. Due to biological variability of cell types as well as current technological limitations there is no catalogue of marker genes covering existing cell types or cell identities, and our knowledge on marker genes is limited. Often times, a cell type is defined based on the expression of ‘historical’ marker genes, i.e. genes that have been found over many years of research on a given cell type. Yet, many more genes may be specific for a given cell type. This might be the case for genes less studied in the past, and thus, less present in the literature than other, more frequently studied

0022-2836/© 2022 The Author(s). Published by Elsevier Ltd.

genes. As a consequence, focusing on marker genes derived from the literature might lead to overlooking of a heterogeneity within a cluster, followed by a wrong biological interpretation of clustering results.

To tackle this problem we present *APL* – an R package which allows for identification and visualization of cluster-specific genes from transcriptomics data. Based on a scRNA-seq data set and precomputed clusters of cells, *APL* generates Association Plots, a planar representation of gene-cluster associations.[1] The R package *APL* computes Association Plots for single-cell transcriptomics data in such a way that the user can interactively query the plots. *APL* can also be integrated into single-cell data analysis pipelines.

Association Plots provide for identification of cluster-specific genes in single-cell data by plotting genes in a two-dimensional coordinate system. This is achieved by applying correspondence analysis (CA), a method allowing for a simultaneous embedding of both genes and cells in one space, to single-cell data. The location of genes in the CA space reveals information on their expression across cells from the data. By measuring distances in this space we are able to represent associations between genes and a cluster of cells in a two-dimensional coordinate system, the so-called Association Plot. The horizontal axis indicates how strongly the gene is associated to a cluster and the farther to the right it lies, the stronger is the association. The vertical axis indicates whether other clusters also show expression of this gene, such that the most characteristic genes for a cluster can be found near the x-axis far to the right.

Although the arrangement of the genes in the Association Plot is derived from a high-dimensional embedding, Association Plots are always planar. Yet, they do not rely on simply projecting data into a plane for reduction of data dimension, and do not incur the information loss associated, e.g., with projecting a principal component analysis into two or three dimensions. This is particularly important when working with large, complex data, where projection into a few dimensions tends to be associated with a large information loss. Association Plots, thanks to their dimension-independence, address this issue and allow for accurate prediction of cluster-specific genes even in high-dimensional data. This will be further discussed in the Results Section.

Applying *APL* to single-cell data offers a wide range of applications. First of all, it facilitates exploration of high-dimensional data, and thereby enables a better understanding of complex data sets. Second, generating Association Plots for selected clusters from the data allows for identification of novel marker genes characterizing different cell clusters and cell identities. Based on this, Association Plots can also be applied for cluster annotation purposes. This is effected by comparing literature-derived marker genes for different cell types with the marker genes derived from the Association Plots generated for the given cell clusters.

This paper is organized as follows. First, we will explain the idea behind Association Plots and present how to use them for visualization of cluster-specific genes in high-dimensional single-cell RNA-seq data. Next, we will demonstrate the applications of Association Plots using two single-cell RNA-seq data sets - the 3k Peripheral Blood Mononuclear Cell (PBMC) data set from 10X Genomics[2,3] and the human cell atlas of fetal gene expression.[4] We will present our newly developed R package *APL* for generating Association Plots. Finally, we will explore the relationships between *APL*-derived cluster-specific genes and those obtained by other computational approaches, and provide examples of gene enrichment analysis in the framework of Association Plots.

Results

Association Plots are defined based on the geometry of correspondence analysis

Association Plots derive their capability of depicting genes associated to a cluster of cells from a feature of correspondence analysis (CA). CA is a data embedding method resembling Principal Component Analysis (PCA), albeit with a few characteristic differences. CA embeds both cells and genes in one real-valued space. While for visualization purposes, points from this space are traditionally projected down into two or three dimensions, we refrain from doing so. Instead we maintain a large number of dimensions of the original data so as to reduce noise while maintaining the defining information. By default we retain the number of dimensions calculated using the so-called elbow rule. See Methods for more information on this and other methods for the choice of dimension number. We call the resulting space with points for cells and for genes *CA-space*. In this space, CA places cells with similar transcript profiles near each other, and, likewise, arranges genes with similar distribution over cells near each other. Most importantly for our application, in CA-space a cluster of similar cells defines a direction from the origin to that cluster, and genes which are highly expressed in this cluster but not elsewhere lie in that very direction.

In Figure 1(a) this direction is defined by the vector from the origin to the centroid (grey dot with orange border) of the cell cluster (orange dots). Genes that are associated to the cluster (black dots) lie in this direction in space. Note that this would be the same geometry even in a space of much higher dimension. The stronger an

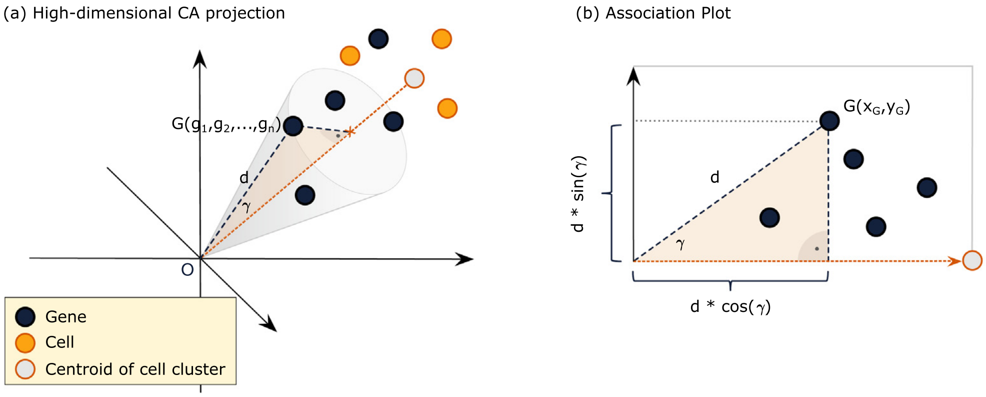

Figure 1. Association Plots delineate cluster-specific genes. (**a**) In a high-dimensional CA space a cluster of cells (orange dots) defines a direction, here represented by the orange line pointing from the origin to the centroid of the cell cluster. The genes (black dots) associated to this cluster of cells are located close to this line along its direction. (**b**) For the Association Plot we only use the length from the origin to the genes's projection onto the orange line (d*cos(γ)) as the first coordinate of the gene in the Association Plot. The length of the perpendicular distance from the gene to the line (d*sin(γ)) is the second coordinate. Thus, the x-axis of the Association Plot corresponds to the line pointing towards the cluster centroid shown at the end of the vector.

association between a gene and a cell cluster, the farther out towards this cluster a gene will be located. Therefore, the length of the orthogonal projection of the gene-point onto the vector towards the centroid is an indicator of the strength of the association. As shown in Figure 1(b), we use this length ($d * cos(\gamma)$) as the x-axis for the gene in the Association Plot. The perpendicular distance from a gene to this vector ($d * sin(\gamma)$) constitutes the y-axis of the gene in the Association Plot. This distance will be short when the gene is very specific for the cluster. When the gene is also expressed in other clusters, then it will be farther away from the direction to the centroid, and therefore have a larger y-coordinate in the Association Plot. The Association Plot for a given cell cluster depicts all genes with these two coordinates in a two-dimensional space. Thus, despite being planar, Association Plots are independent of the dimension of the CA-space and they capture information from the high-dimensional space without discarding dimensions. A mathematical description of Association Plots is given in (Gralinska and Vingron, 2021).[1]

Visualizing and scoring cluster-specific genes

Association Plots are primarily a visualization tool for genes that are associated to a cluster of cells. Genes positively associated with the selected cluster of cells will be located in the right bottom part of the plot. This is due to the fact mentioned above that in the CA space such genes align with the direction towards this cluster and are located in close proximity to the cluster centroid. On the other hand, genes which do not show any association with the cluster will be located close to the Association Plot's origin. In the CA space such genes do not align with the direction towards the selected cluster and are located closer towards other clusters.

To facilitate the interpretation of Association Plots, we also implemented a scoring system which aims at ranking genes according to their cluster-specificity. To this end, we use random permutation of the data to determine an angle α in the Association Plot, above which 99% of genes might lie due to chance. The score S_α[1] for a gene at coordinates (x, y) in the Association Plot is then computed according to the formula:

$$S_\alpha(x, y) = x - \frac{y}{\tan\alpha}.$$

S_α will be high for genes far away from the origin and near the x-axis of the Association Plot. S_α will be constant along parallel level lines of degree α in the Association Plot. The higher S_α, the higher the cluster-specificity of a gene. Figure 2(a), e.g., displays genes in the Association Plot colored according to the S_α level.

Association Plots for the 3k PBMC data

To demonstrate how to use Association Plots for studying gene-cluster associations and for identification of novel marker genes characterizing a cell cluster from single-cell data, we applied our method to the 3k Peripheral Blood Mononuclear Cell (PBMC) single-cell RNA-seq data.[2,3] PBMC data was generated by 10X Genomics and allows for studying the immune populations within PBMCs from a healthy donor.

When applying Association Plots to a single-cell transcriptomics data, clustering of cells will typically be part of data pre-processing. For this we followed the vignette from the Seurat package[5] (more details in the Methods Section). Using Seurat the clusters were annotated to different cell types

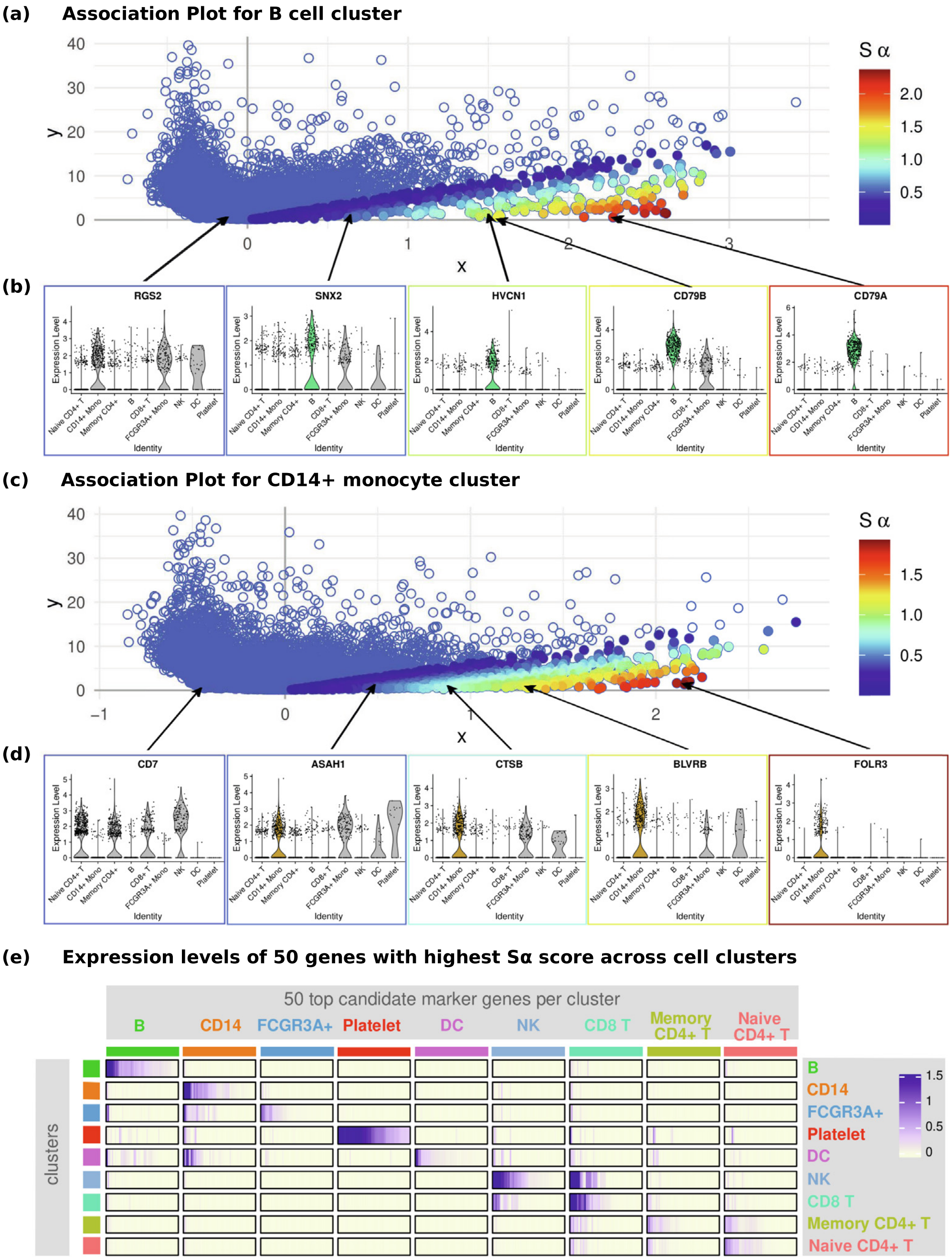

Figure 2. Cluster-specific genes from 3 k PBMC data. (**a**) Association Plot generated for the B cell cluster. Each circle represents one gene from the input data. Genes with the positive S_α score are highlighted in color according to the color map given. (**b**) Comparison of the expression levels of five example genes from (a) across nine cell types. (**c**) Association Plot generated for the CD14+ monocyte cluster. (**d**) Comparison of the expression levels of five example genes from (c) across nine cell types. (**e**) Average expression levels of 50 candidate marker genes per cell type, identified using the Association Plots, across all nine cell types.

based on the expression of canonical marker genes for immune cells (Supplementary Figure 1).[5] This annotation allows us to address clusters by their cell-type rather than by a number. The following cell types were identified: B cells, naive CD4+ T cells, memory CD4+ T cells, CD8+ T cells, FCGR3A+ monocytes, natural killer (NK) cells, CD14+ monocytes, dendritic cells (DC), and platelets.

We also generated Association Plots for each of the clusters of cells using the first 223 CA dimensions. Figure 2 presents two example Association Plots for the B cell- (Figure 2(a)) and CD14+ monocyte clusters (Figure 2(c)). Genes with the positive S_α score are highlighted in color according to the color map given. To illustrate the linkage between S_α score and gene expression

patterns across cell clusters we focus on 10 example genes with different S_α values. In Figure 2(b) we present the expression levels of five random genes from the B cell cluster Association Plot across nine clusters for comparison: RGS2, SNX2, HVCN1, CD79B, and CD79A. As shown in the violin plots, with increasing S_α score the over-expression of a given gene in the B cell cluster gets more pronounced. For instance, while in the case of CD79A, a gene with the highest S_α score equal 2.18, we observe a clear over-expression in the B cell cluster, in the case of SNX2, a gene with the S_α score of 0.51, the over-expression signal in the B cell cluster is almost not visible. On the other hand, for RGS2, a gene with a negative S_α score located in the left bottom part of the Association Plot, we observe the over-expression in three other cell clusters and not in the B cell cluster.

Figure 2(d) demonstrates analogous observations as in Figure 2(b), this time for five genes from the CD14+ monocyte Association Plot: CD7, ASAH1, CTSB, BLVRB, and FOLR3. For CD7, a gene located in the bottom left part of the Association Plot, no over-expression signal in the CD14+ monocyte cluster is observed. Instead, it is over-expressed in four other cell clusters: naive CD4+ T, memory CD+, CD8+ T, and NK cells. On the other hand, a gene with the highest S_α value of 1.93, FOLR3, shows a strong over-expression in the CD14+ monocyte cluster. This, together with the three remaining plots generated for ASAH1, CTSB and BLVRB confirms the link between S_α score and gene expression patterns across cell clusters.

Next, we employ Gene Set Enrichment Analysis (GSEA)[6–8] to show that genes with high S_α values are associated to the cell type for which the respective Association Plot was computed. GSEA was performed on the 100 genes with the highest S_α values. The results for two cell clusters, B cell- and CD14+ monocytes cluster, are presented in Table 1 and Table 2, respectively. For the B cell cluster nine out of the top 10 enriched gene sets are linked to the B cell population. For the CD14+ monocyte cluster four out of the top 10 enriched gene sets are directly related to the monocyte population. Two further gene sets are related to the myeloid cell population, which reflects the monocyte-specificity of the gene markers from the CD14+ monocyte cluster. Thus, the genes associated to a cell type based on high S_α are indeed characteristic for the respective cell types.

Figure 2(e) provides an overview of uniqueness vs sharing of cluster-specific genes for the cell-type clusters in the PBMC data. From each of the nine Association Plots we extracted the 50 genes with the highest S_α score. Each of the nine rectangles on the main diagonal of the matrix represents those 50 genes from the respective Association Plot each as a little heatmap: The genes' within-cluster average expression strength is encoded in color, with genes sorted by expression from left to right. The rectangles in the same column contain the genes from the main, diagonal rectangle in the same order, and with the color commensurate to the average expression level in the other cluster.

While marker genes obtained from well-separated clusters such as B cells, platelet or dendritic cells show a strong over-expression in only one cluster, the identified marker genes from clusters located in close proximity to another cluster in correspondence analysis space are also partially up-regulated in the neighboring clusters. This is observed for natural killer cell- and CD8+ T cell cluster, as well as for the CD8+ T cell cluster, the memory CD4+ T cell cluster, and the naive CD4+ T cell cluster. Cell clusters located in close proximity to each other share similar gene expression profiles, which results in a low number of genes characteristic for only one of these clusters.

Association Plots aid in identification of novel marker genes

When a set of established marker genes for a cell type is given, an Association Plot for a cell cluster corresponding to that cell type may serve to support the identification of novel marker genes. This is an important task in the context of less well characterized cell types. We demonstrate how to proceed on the example of the Association Plot generated for the B cell cluster from the 3k PBMC data.

Figure 3(a) presents the Association Plot for the B cell cluster, with 242 B-cell enriched genes highlighted using grey filling. This set of genes was obtained from the Human Blood Atlas,[9] a collection of information on the human protein-coding genes across distinct human blood cell types. In the Human Blood Atlas all genes with at least four times higher normalized expression values in B cells than in any other cell type are qualified as B-cell enriched genes.[10] In the Association Plot these genes obtained statistically higher S_α scores than the remaining genes (Wilcoxon test, p-value 1.505e-35). This is also visible in the Association Plot with the majority of the B-cell enriched genes located within the area of positive S_α values, which confirms a substantial overlap between the Association Plot results and the marker gene set from the Human Blood Atlas. In addition to this, single marker genes are located outside of the positive S_α area. This might be caused by the differences in data sets used for computing Association Plots and for extracting cell type enriched genes in the Human Blood Atlas.

Novel marker genes will be located among genes with high S_α scores, and which at the same time are not annotated yet as marker genes for a given cell

Table 1 GSEA results of 100 top genes from B cells.

Gene Set Name	# Genes in Gene Set (K)	Description	# Genes in Overlap (k)	k/K	p-value	FDR q-value
HAY_BONE_MARROW_ FOLLICULAR_B_CELL	142	–	48	0.338	1.01E−95	2.07E−91
GSE10325_CD4_TCELL_ VS_BCELL_DN	194	Genes down-regulated in comparison of healthy CD4 [GeneID = 920] T cells versus healthy CD19 [GeneID = 920] B cells.	51	0.2629	1.33E−95	2.07E−91
GSE10325_LUPUS_CD4_ TCELL_VS_LUPUS_ BCELL_DN	195	Genes down-regulated in comparison of systemic lupus erythematosus CD4 [GeneID = 920] T cells versus systemic lupus erythematosus B cells.	47	0.241	1.65E−85	1.71E−81
GSE4984_UNTREATED_ VS_GALECTIN1_ TREATED_DC_DN	191	Genes down-regulated in monocyte-derived dendritic cells: control versus treated with LGALS1 [GeneID = 3956].	40	0.2094	2.63E−69	2.04E−65
GSE29618_BCELL_VS_ MONOCYTE_DAY7_FLU_ VACCINE_UP	195	Genes up-regulated in comparison of B cells from influenza vaccinee at day 7 versus monocytes from influenza vaccinee at day 7.	39	0.2	1.27E−66	7.92E−63
GSE29618_BCELL_VS_ MONOCYTE_UP	194	Genes up-regulated in comparison of B cells versus monocytes.	38	0.1959	1.88E−64	9.75E−61
GSE29618_BCELL_VS_ MDC_DAY7_FLU_ VACCINE_UP	192	Genes up-regulated in comparison of B cells from influenza vaccinee at day 7 post-vaccination versus myeloid dendritic cells (mDC) at day 7 post-vaccination.	37	0.1927	2.16E−62	9.6E−59
GSE10325_BCELL_VS_ MYELOID_UP	196	Genes up-regulated in comparison of healthy B cells versus healthy myeloid cells.	35	0.1786	1.28E−57	5E−54
GSE3982_MEMORY_CD4_ TCELL_VS_BCELL_DN	197	Genes down-regulated in comparison of memory CD4 [GeneID = 920] T cells versus B cells.	34	0.1726	2.3E−55	7.95E−52
GSE22886_TCELL_VS_ BCELL_NAIVE_DN	198	Genes down-regulated in comparison of naive CD4 [GeneID = 920] CD8 T cells versus naive B cells.	34	0.1717	2.77E−55	8.63E−52

Table 2 GSEA results of 100 top genes from CD14+ monocytes.

Gene Set Name	# Genes in Gene Set (K)	Description	# Genes in Overlap (k)	k/K	p-value	FDR q-value
HAY_BONE_MARROW_ NEUTROPHIL	450	–	56	0.1244	3.91E−85	1.22E−80
GSE11057_PBMC_VS_ MEM_CD4_TCELL_UP	197	Genes up-regulated in comparison of peripheral mononuclear blood cells (PBMC) versus memory T cells.	30	0.1523	1.38E−46	2.15E−42
GSE29618_MONOCYTE_ VS_MDC_DAY7_FLU_ VACCINE_UP	200	Genes up-regulated in comparison of monocytes from influenza vaccinee at day 7 post-vaccination versus myeloid dendritic cells at day 7 post-vaccination.	30	0.15	2.24E−46	2.33E−42
GSE29618_MONOCYTE_ VS_PDC_UP	199	Genes up-regulated in comparison of monocytes versus plasmacytoid dendritic cells (pDC).	29	0.1457	2.06E−44	1.25E−40
GSE29618_MONOCYTE_ VS_MDC_UP	200	Genes up-regulated in comparison of monocytes versus myeloid dendritic cells (mDC).	29	0.145	2.41E−44	1.25E−40
GSE29618_MONOCYTE_ VS_PDC_DAY7_FLU_ VACCINE_UP	200	Genes up-regulated in comparison of monocytes from influenza vaccinee at day 7 post-vaccination versus plasmacytoid dendritic cells (mDC) at day 7 post-vaccination.	29	0.145	2.41E−44	1.25E−40
GSE10325_LUPUS_CD4_ TCELL_VS_LUPUS_ MYELOID_DN	200	Genes down-regulated in comparison of systemic lupus erythematosus CD4 [GeneID = 920] T cells versus systemic lupus erythematosus myeloid cells.	27	0.135	2.36E−40	9.33E−37
GSE6269_HEALTHY_VS_ STAPH_PNEUMO_INF_ PBMC_DN	170	Genes down-regulated in comparison of peripheral blood mononuclear cells (PBMC) from healthy donors versus PBMC from patients with acute S. pneumoniae infection.	26	0.1529	2.4E−40	9.33E−37
DURANTE_ADULT_ OLFACTORY_ NEUROEPITHELIUM_ DENDRITIC_CELLS	117	–	21	0.1795	3.55E−34	1.23E−30
GSE10325_BCELL_VS_ MYELOID_DN	200	Genes down-regulated in comparison of healthy B cells versus healthy myeloid cells.	23	0.115	1.13E−32	3.2E−29

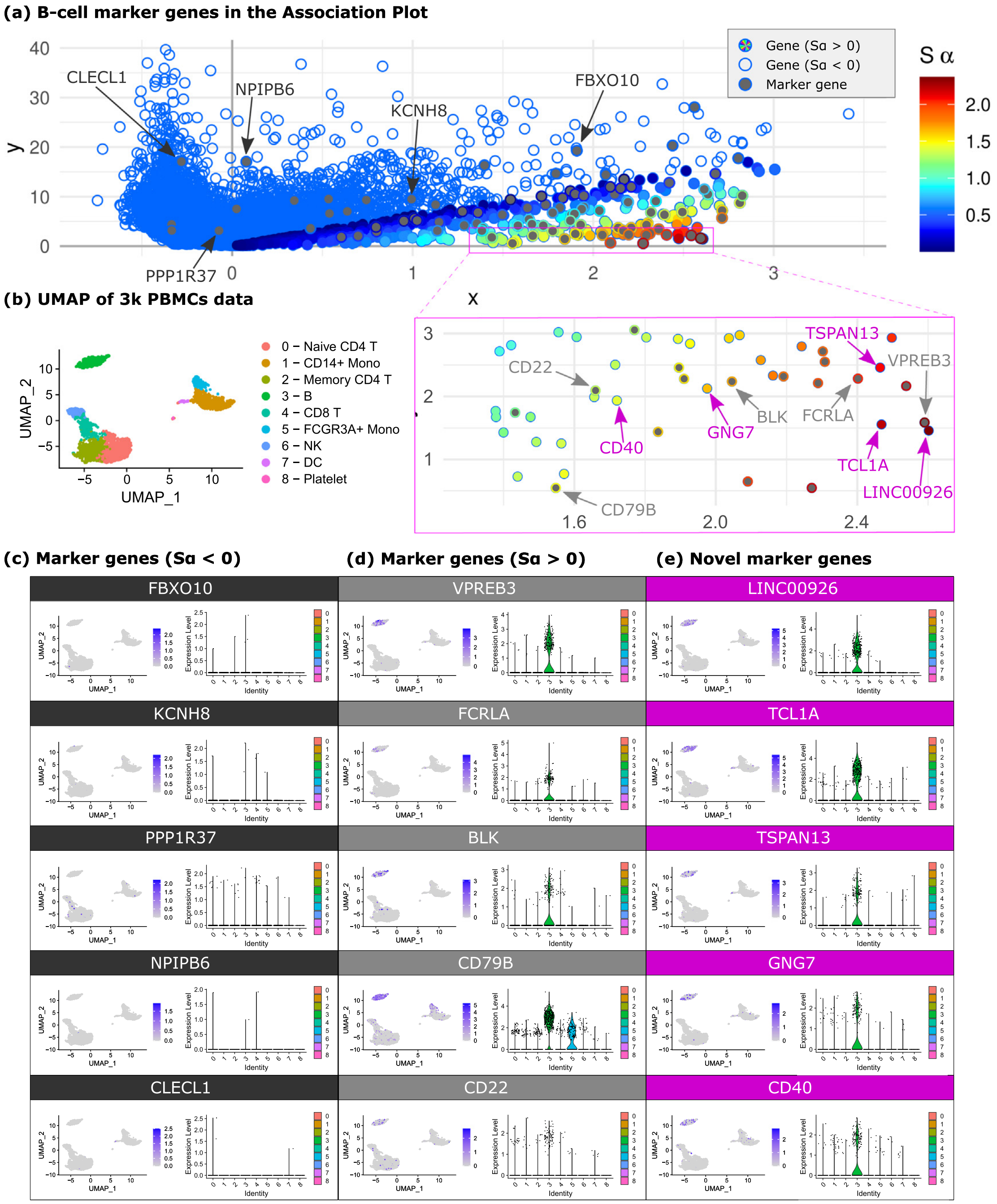

Figure 3. Identification of novel marker genes using Association Plots. (**a**) Association Plot for B cell cluster from the 3k PBMC data. Genes known to be enriched in B cells according to the Human Blood Atlas are highlighted in grey. (**b**) Cell clusters in 3k PBMC data. (**c–d**) Expression levels of example genes enriched in B cells according to the Human Blood Atlas with (c) negative and (d) positive S_α score. (**e**) Expression levels of B-cell specific genes detected using the Association Plot, which are not listed among B-cell enriched genes according to the Human Blood Atlas.

type. In the Association Plot generated for the B cell cluster we highlighted five example genes which can be considered as marker gene candidates: LINC00926, TCL1A, TSPAN13, GNG7, and CD40 (Figure 3(a)). As presented in Figure 3(e), each of these genes is over-expressed in the B cell cluster. For comparison, in the generated Association Plot we also highlighted 10 further example genes listed among the B-cell enriched genes in the Human Blood Atlas. As expected, the first five genes (FBXO10, KCNH8, PPP1R37, NPIPB6, CLECL1) located outside of the positive S_α area do not show any over-expression signal in the B cell cluster (Figure 3(c)), while the further five genes (VPREB3, FCRLA, BLK, CD79B, CD22) are characterized by positive S_α values

and show over-expression in the B cell cluster (Figure 3(d)). Moreover, their expression profiles resemble the profiles of the newly detected candidate marker genes.

The first detected gene is LINC00926, a long non-coding RNA over-represented in the B cell cluster. Even though LINC00926 has not been well-characterized yet, its abnormal expression was observed in several cancer types.[11] For instance, the up-regulation of LINC00926 in B cells in lung adenocarcinoma patients was observed to improve their overall survival.[12] Due to this, LINC00926 was suggested to be a B-cell specific marker gene protecting against lung adenocarcinoma. Moreover, it was also observed to suppress breast cancer growth by down-regulating the expression of phosphoglycerate kinase 1 (PGK1).[13] LINC00926 was also described in the context of acute myeloid leukemia[14] and Hodgkin lymphoma.[15] To our knowledge, there is only one publication available which describes LINC00926 as a B cell marker,[16] together with another gene, TCL1A, the second marker gene candidate identified using the Association Plot.

TCL1A, T-cell leukemia/lymphoma protein 1A, is a gene involved in the regulation and differentiation of B cells. Over-expression of this gene is linked to the T- and B-cell lymphomas.[17,18] Although TCL1A is not present in the B-cell enriched gene list from the Human Blood Atlas, it is classified there as a “cell lineage group enriched gene”, and thus, its up-regulation is simultaneously observed in B cells and plasmacytoid DCs. However, in the 3k PBMC data TCL1A is up-regulated only in some cells from the DC cluster and, thus, it still scores high in the Association Plot for B cells.

Further example genes from the Association Plot showing B cell cluster specificity are GNG7, CD40, or TSPAN13. Similar to TCL1A, GNG7 is classified as a “cell lineage group enriched gene” in the Human Blood Atlas and its up-regulation is observed both in B cells and plasmacytoid DCs. However, according to the 3k PBMC data this gene is partially up-regulated only in the B cells. The fourth detected gene, CD40, according to the Human Blood Atlas is only enhanced in the B cells, while in the 3k PBMC data it is visibly over-expressed in the B cell cluster. TSPAN13, the last gene identified from the Association Plot, is classified as enhanced both in the naive B cells and plasmacytoid DCs according to the Human Blood Atlas, while in the 3k PBMC data it is up-regulated only in the B cell cluster.

Association Plots aid in annotating cell clusters to known cell identities

Another typical task in single-cell data analysis is the annotation of clusters of cells to known cell types. We proceed to demonstrate this on the example of stomach single-cell data from the human cell atlas of fetal gene expression.[4]

Among the existing methods for cluster annotation one can distinguish two main types. The first group encompasses methods that rely on a reference database. In this case expression profiles of cells from a given cluster are compared against expression profiles of various cell types from a reference database. Alternatively, cluster annotation can be conducted using a literature-derived list of marker genes for various cell types, where the expression analysis of such markers allows then for matching a given cell cluster to a cell type. We proceed according to this second paradigm and work with given lists of marker genes for different cell types.

The single-cell data from the stomach comes from the human cell atlas of fetal gene expression and according to the subcluster analysis conducted by the authors of the original study it consists of 16 subclusters (see Methods Section “Subclustering analysis” in (Junyue et al., 2020)[4]). For each subcluster, we generated its Association Plots, yielding 16 Association Plots depicted in Figure 4(a)–(p). The plots were generated using the first 4047 CA dimensions, as determined by the elbow rule. To annotate the generated Association Plots to 16 cell types we use a set of within-tissue marker genes from stomach provided by the authors of the original study. Altogether, among the sets of within-tissue marker genes reported by them, there were 64 marker genes with subsets characteristic of individual cell types from stomach. Thus, in each generated plot we highlighted the complete set of 64 marker genes for all 16 stomach cell types, leading to the images of Figure 4(a)–(p).

In each of the generated plots the majority of the highlighted genes are located on the left side of the plot, which indicates no association between them and the depicted cell subcluster. However, in each plot a few genes are located on the right hand side. These are the marker genes for that respective subcluster. Therefore, in the last step of the analysis we focused only on these genes and used them to match each Association Plot to one of the 16 stomach cell types based on the provided list of within-tissue marker genes. This allows to easily assign the identity of all 16 stomach clusters from the data as shown in a UMAP (Figure 5). To illustrate the results, in Figure 4(a)–(p) we show all the 16 Association Plots together with their real (as given by the original authors) cell type, and the corresponding marker genes for a cell type highlighted in color.

We have used this example to demonstrate how easy it is to obtain cell-type assignments based on Association Plots. There is no need to search for the right set of marker genes for a cell cluster, but mapping the union of all marker genes into the Association Plots yields an easy to interpret visualization from which the identity of the cluster can be inferred. Our results were in agreement with

the cluster information from the original data, which demonstrates that Association Plots can be applied for annotating the cell clusters to known cell types based on the predefined list of marker genes.

R package

APL is a freely available R package for identification of cluster-specific genes using Association Plots. The package was developed in a way that allows for applying it to single-cell transcriptomics data and extracting a list of genes specific for any selected cell cluster.

When working with single-cell transcriptomics data we recommend providing the input data as a *Seurat* or *SingleCellExperiment* object. Alternatively, the input data can be provided in form of a normalized count matrix, with rows representing genes and the columns representing cells. In addition to this, *APL* can also be applied to any type of the data represented in form of a matrix with non-negative entries. To run the analysis, the input data should be specified as the *obj* parameter in the function *cacomp*.

Association Plots are computed using a function *apl_coords*. To run this function the user needs to specify the *group* parameter indicating for which cells from the input data the Association Plot should be computed. Therefore, the user should use the indices or names of cells belonging to a cluster of interest, e.g. according to the clustering information provided beforehand. We additionally

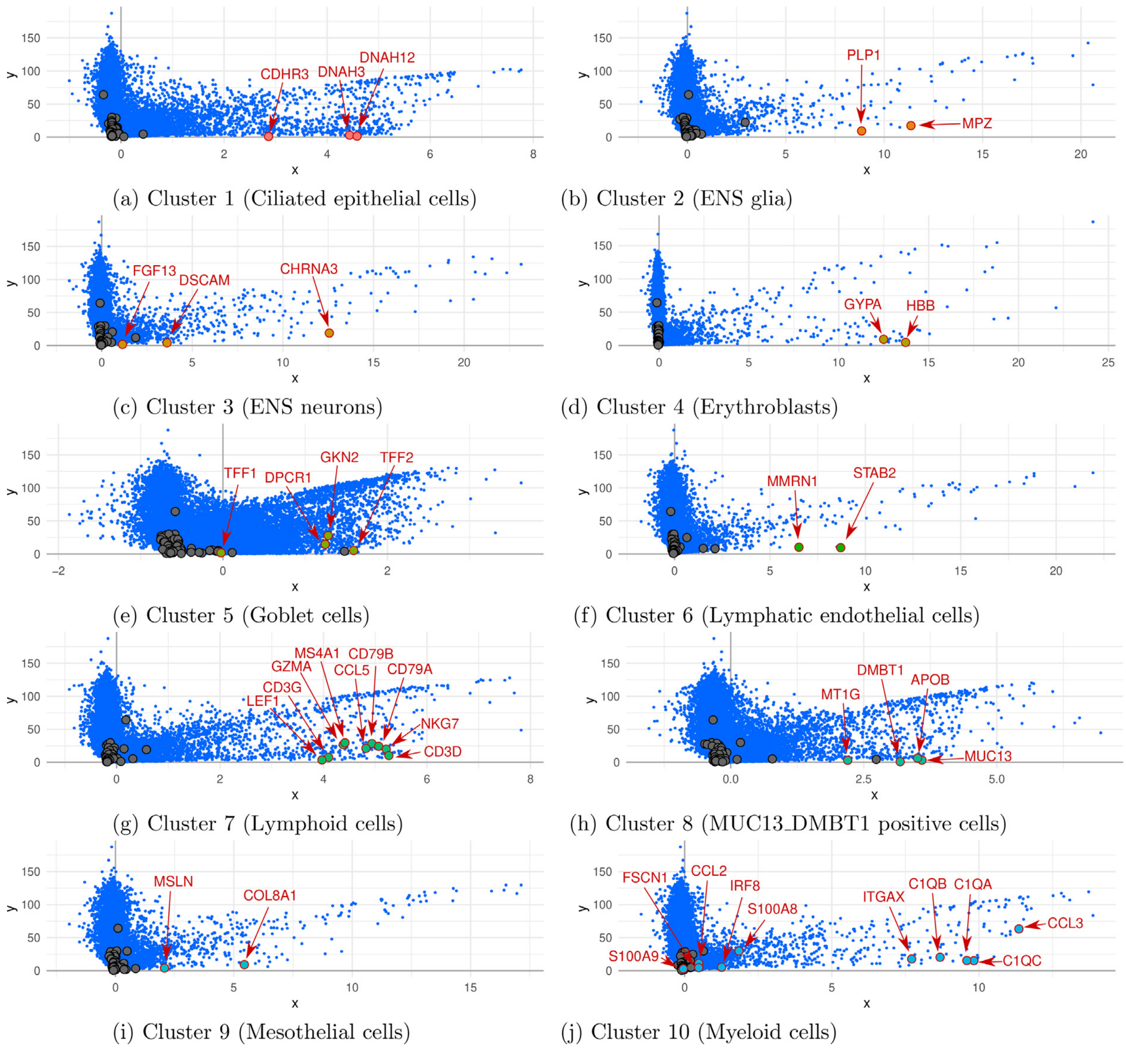

(a) Cluster 1 (Ciliated epithelial cells) (b) Cluster 2 (ENS glia)

(c) Cluster 3 (ENS neurons) (d) Cluster 4 (Erythroblasts)

(e) Cluster 5 (Goblet cells) (f) Cluster 6 (Lymphatic endothelial cells)

(g) Cluster 7 (Lymphoid cells) (h) Cluster 8 (MUC13_DMBT1 positive cells)

(i) Cluster 9 (Mesothelial cells) (j) Cluster 10 (Myeloid cells)

Figure 4. Annotation of cell clusters from the human cell atlas of fetal gene expression using known marker genes. (**a–p**) Association Plots for each of the 16 stomach subclusters with a set of 64 within-tissue marker genes. Marker genes specific for a given cell type are highlighted in color. The colors correspond to the UMAP color scheme (Figure 5).

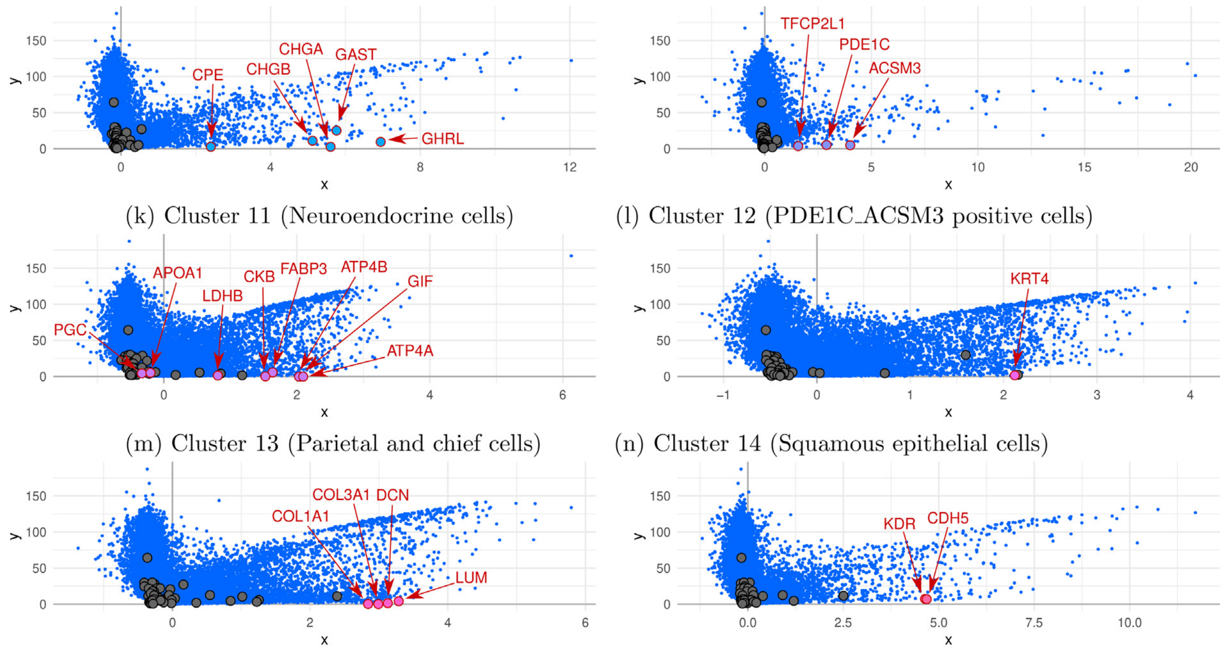

(k) Cluster 11 (Neuroendocrine cells)
(l) Cluster 12 (PDE1C_ACSM3 positive cells)
(m) Cluster 13 (Parietal and chief cells)
(n) Cluster 14 (Squamous epithelial cells)
(o) Cluster 15 (Stromal cells)
(p) Cluster 16 (Vascular endothelial cells)

Fig 4. *(continued)*

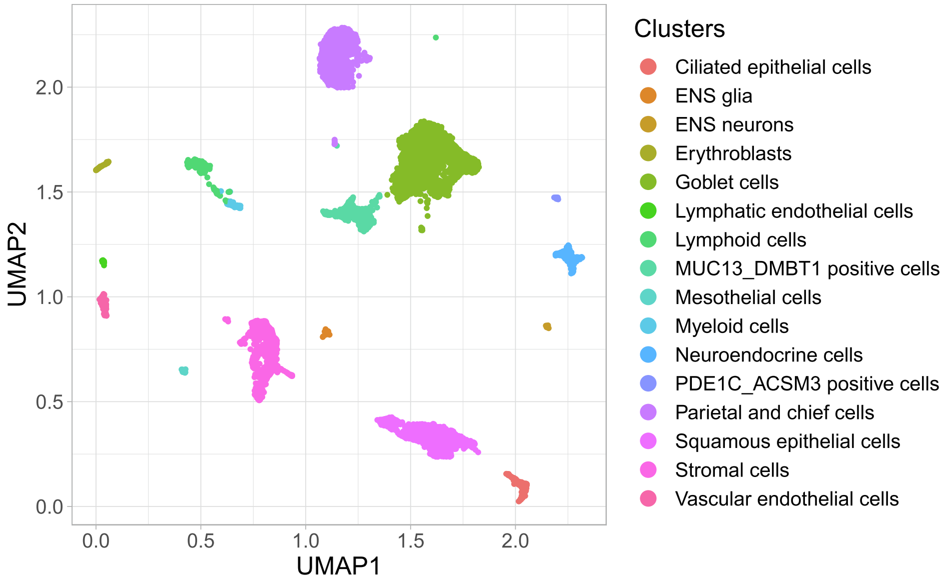

Figure 5. UMAP visualization of stomach cells from human cell atlas of fetal gene expression. The cells were annotated to cell types using Association Plots from Figure 4 and the provided list of within-tissue marker genes for each of the 16 cell types. The plot was generated using the UMAP coordinates of stomach cells obtained from the processed data from the original publication.[4]

implemented a wrapper function *runAPL*, which automates the above-described steps. Finally, to display the computed Association Plot a function *apl* should be called.

S_α scores for ranking genes are computed with the function *apl_score*. The S_α scores are then stored in the *APL_score*attribute of a *ca* object.

To investigate the expression of a gene that was identified as interesting in an Association Plot across the clusters of the single-cell transcriptomics data, external plotting functions such as *VlnPlot*, for generating a violin plot, and *FeaturePlot*, for generating a feature plot, from the *Seurat* package can be used.

By default the computation of Association Plots is done using 5,000 genes with the highest variance across cells. This number can be changed using the parameter *top* in the functions *cacomp* or *runAPL*. The wrapper function *runAPL* uses by default the number of CA dimensions computed using the elbow rule (see below). When using the *cacomp* function the user should specify the number of CA dimensions using the parameter *dims*. We implemented three methods for selecting a dimension number: elbow rule (`elbow_rule`), 80% rule (`maj_inertia`), and average rule (`avg_inertia`). The user can also estimate the number of dimension using a scree plot, which can be generated using the function `scree_plot`. For more details see Methods Section.

Association Plots are computed based on the geometry of correspondence analysis. Therefore it is possible to plot the two- or three-dimensional input data projection of the correspondence analysis space. This is done by the functions `ca_biplot` and `ca_3Dplot`, respectively.

The *APL* package can be integrated into existing pipelines, and Association Plot results can be used as an input for functions from other packages. For instance, to conduct GO enrichment analysis of cluster-specific genes identified using Association Plots we developed a function *apl_topGO*, which allows for conducting a GO enrichment analysis using the *topGO* package. By default the function is applied to the genes from an Association Plot with S_α score above a defined threshold.

Further information about the package, installation, usage details and examples can be found in the Methods Section and the vignette provided with the package. The package is available from https://github.com/VingronLab/APL.

Comparison to differential expression testing tools

As a tool for visualizing genes that are characteristic for a cell cluster, Association Plots can also be seen as a way of determining genes that are differentially expressed between the cells in the cluster vs all other cells. Thus, we need to answer the question how comparable are the results obtained using Association Plots to the results from commonly used differential expression testing tools.

To address this question we need to choose a set of differential expression (DE) tools for single-cell RNA-seq data to include in a comparison. There is no consensus in the community on which of the existing differential expression testing methods is the best one for single-cell RNA-seq data. Comparative studies of various existing tools revealed an unsatisfying agreement among them.[19,20] Even though there also exist tools developed especially for single-cell RNA-seq data, it was recently suggested that the standard tools for bulk RNA-seq data do not perform worse than the specialized single-cell RNA-seq tools.[19] Therefore, we decided to follow recent recommendations[21] and focused on two differential expression testing tools for bulk RNA-seq data, DESeq2[22] and edgeR,[23,24] combined with ZINB-WaVE weight estimation method.[25] In addition to those we include in the comparison the FindAllMarkers function from Seurat,[5] which is specifically designed for delineating marker genes from single-cell data.

To investigate the agreement among results obtained with DESeq2, edgeR, Seurat, and Association Plots, we applied them to the 3k PBMC data. First of all, sets of 1000 most up-regulated or cell-type specific genes were extracted for each cluster and tool (see Methods for details), and the overlaps between them were investigated. For Association Plots the top 1000 genes refer to the ranking by S_α score. Figure 6 shows the overlap between the results of the four approaches for all nine cell types of the 3k PBMC data. For all cell types, results obtained using Association Plots agree the most with Seurat results. The overall lighter color of the matrix for dendritic cells (DC) indicates that in this cell type the different methods agree the least. In DC, Association Plots share 547 out of 1000 genes with Seurat, whereas in natural killer cells (NK) 833 genes are shared between these two methods. DESeq2 and edgeR, in turn agree more with each other than with either Seurat or Association Plots, as can be seen for eight out of nine cell types.

We proceed to demonstrate how mapping the sets of differentially expressed genes into an Association Plot allows to visualize and study the differences or agreements. We focus on the case of the dendritic cells since there the agreement between methods was smallest. Figure 7 shows three times the same Association Plot for the DC cluster, overlaid respectively with the 250 most differentially expressed genes from Seurat, edgeR, and DESeq2. The genes are chosen according to thresholds selected appropriately for the individual method (see Methods).

Comparison of the highlighted differential gene sets in the three subfigures shows general agreement with interesting particular differences. For example, Seurat classifies a few genes very far to the right as differential. Those genes are characterized by high expression of the respective gene albeit only in a subset of cells in the cluster. As a consequence, a method like DESeq2 may assign a high fold-change but a non-significant p-value. For most of the genes that one would judge as differential from the visual impression provided by the Association Plot, there is at least one of the other methods that would also identify that gene. Thus, the Association Plot serves well as a summary of the relevant genes to be explored further. The *APL* package allows for clicking on

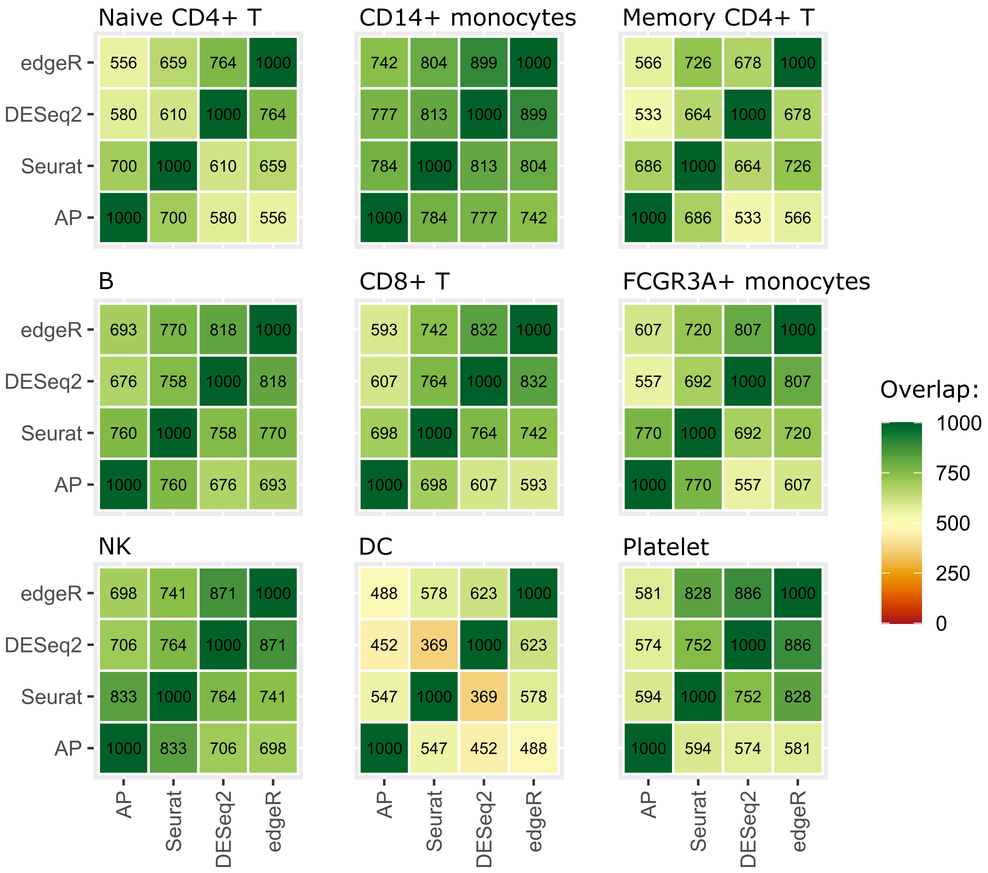

Figure 6. Agreement among the results obtained with DESeq2, edgeR, Seurat, and Association Plots for the 3 k PBMC data. For each cell cluster and tool 1000 most up-regulated or cell-type specific genes were extracted. The overlaps between them are shown in the heatmaps. AP, Association Plot.

dots in the Association Plot to learn about the identity of the gene.

Gene ontology enrichment analysis

To interpret the biological meaning of a cluster-specific gene set identified using Association Plots the *APL* package allows for conducting and visualizing Gene Ontology (GO) enrichment analysis using the R package *topGO*.[26] We demonstrate this on the example of the lymphoid cell cluster from stomach, as obtained from the human cell atlas of fetal gene expression.

From the Association Plot generated for these lymphoid cells we extracted genes with S_α values above 1. This resulted in a set of 358 genes which we subsequently subjected to GO enrichment analysis (see Methods). From the result, the 10 most significantly enriched GO terms are shown in Figure 8. A prominent role of GO terms related to T cells and immunity is apparent in the table. The majority of lymphoid cells in stomach are T cells,[4] which is in line with most of the terms being related to T cells.

These results can be made more intuitive by mapping the information into the Association Plot. Genes belonging to a given GO term can be highlighted in the Association Plot. As an example, in the Association Plot for lymphoid cells (Figure 9) we highlighted all genes annotated to a GO term ‘GO:0050853 B cell receptor signaling pathway’. Most of these genes are significantly enriched (located in rainbow area). Other genes, which are not in the region where $S_\alpha > 1$ are visibly still close to this region. In particular, one can recognize those genes of a GO category which are strongly associated to the cluster as opposed to others which are apparently shared with other clusters or are lowly expressed in the cluster.

Although in the presented example we demonstrated the results for lymphoid cells obtained using the *topGO* package, the results from Association Plots can be smoothly integrated with various R packages.

Discussion

We have presented the use of Association Plots for visualization and analysis of single cell transcriptomics data. This type of data set tends to be particularly large such that visualization and, in particular, interactive querying of the data is

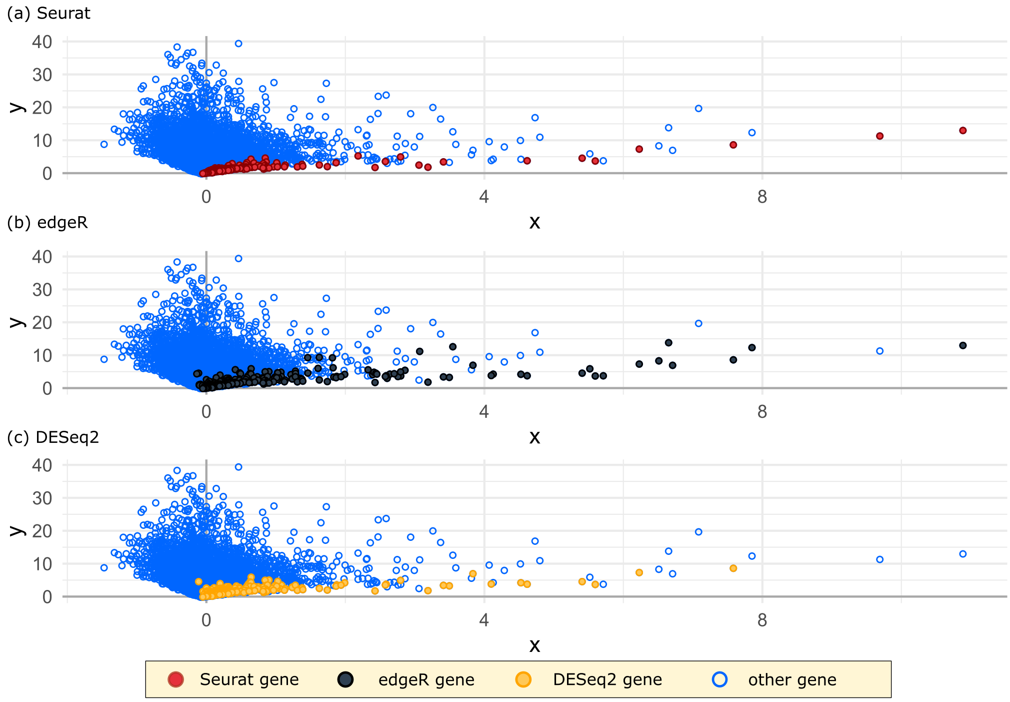

Figure 7. Association Plot for the DC cluster from 3k PBMC data, overlaid with 250 most differentially expressed genes from (**a**) Seurat, (**b**) edgeR, and (**c**) DESeq2.

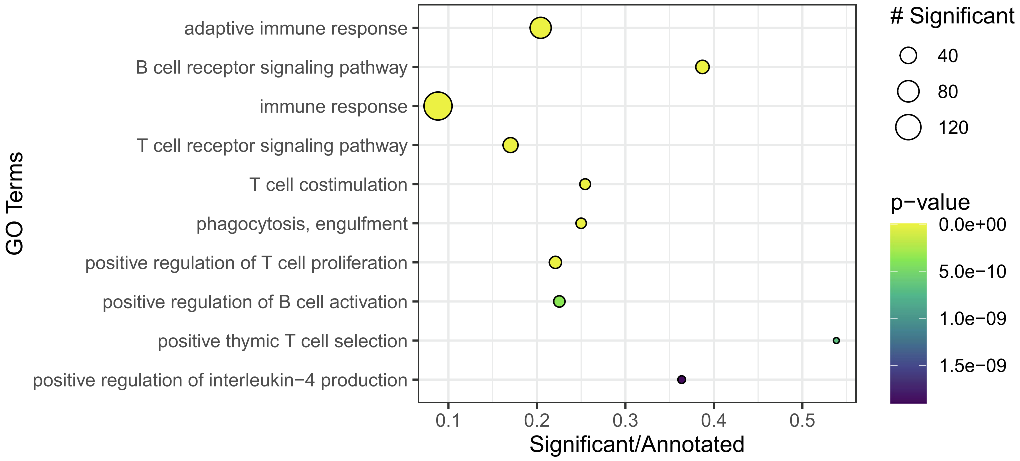

Figure 8. GO enrichment analysis of lymphoid cells from the stomach cluster obtained from human cell atlas of fetal gene expression. The figure presents our visualization of the *topGO* results, implemented in the *APL* package. Only 10 most significantly enriched GO terms are shown.

challenging. The question we address is the identification of genes that are associated to individual clusters in the data. We assume that the clustering is given, although in other ongoing work we focus on the clustering problem.

We developed an R package *APL* that is freely available on GitHub. It computes and displays Association Plots and allows for querying various aspects of the data. The plots generated by *APL* are interactive and allow for investigating the identity of each gene by moving the mouse cursor over a given point in the plot. Moreover, the plots can also be dragged and zoomed, which facilitates the investigation of genes of interest. Thanks to this, using *APL*, one can extract sets of marker genes for a given cell cluster, map marker genes into an Association Plot for the purpose of cell type annotation, or visualize gene set enrichment in a cell cluster.

We applied Association Plots to two example single-cell data sets: The 3k PBMC data set containing information on gene expression in

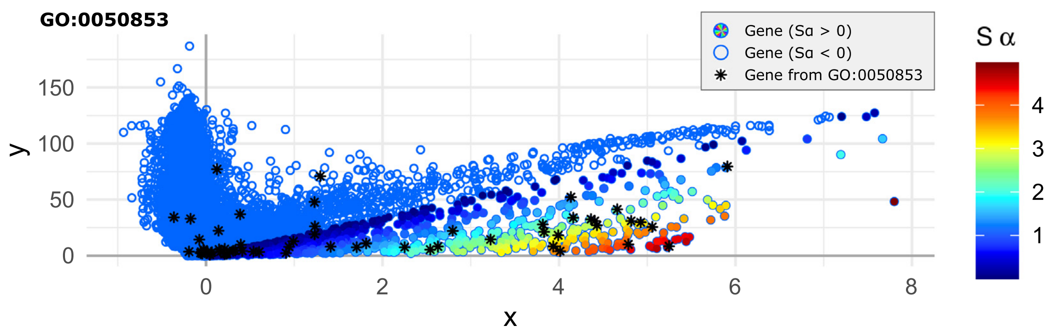

Figure 9. Location of genes annotated to the GO term 'GO:0050853 B cell receptor signaling pathway' in the Association Plot for the lymphoid cells. Genes belonging to this GO category are marked using black stars.

peripheral blood mononuclear cells, and the human cell atlas of fetal gene expression containing information on *in vivo* gene expression across diverse organs and cell types. The clustering we relied on came from the Louvain algorithm in the Seurat package.

Association Plots can depict the association of genes to a cluster in a planar coordinate system, independent of the original dimension of the data. Unlike with, e.g., PCA, this is not achieved by simple projection. Given the complexity of single cell data, projection into the plane would lead to a huge loss of information. Rather, the two dimensions of the planar Association Plot represent characteristic measures for the association between a gene and a cluster centroid as derived from high dimensional correspondence analysis space. Thus, it constitutes a non-linear mapping of the high-dimensional image into a plane, while preserving the gene-cluster association features.

From the practice of projecting data along principal coordinates into lower dimensions we borrow only the noise reduction aspect when defining CA space. Given very large data that reside in thousands of dimensions, after singular value decomposition typically many dimensions will essentially be noise. These are associated to small singular values. Thus we employ standard methods, like the elbow method, for estimating the number of dimensions to keep in our representation of the data in CA space. This will typically be way more dimensions than three, but also way less than the full dimensionality of the data. A positive side effect of reducing the number of dimensions in this way is that computations become faster than when done on the original data. In the *APL* package we implemented three alternative methods for computing a number of dimensions.

A marker gene is meant to distinguish a particular cell type from other cell types. In the context of single cell transcriptomics, a marker gene can highlight one cluster over the other clusters in the data set. The marker genes delineated by an Association Plot thus need to be understood as 'relative' marker genes depending on the given data set, and the composition of the set of marker genes may vary depending on the cell identities present in the data. Clearly, this is no different from other commonly-used tools for differential expression testing, and thus, searching for novel marker genes should be always accompanied by the appropriate experimental design.

Conclusions

In conclusion, we demonstrated the application of Association Plots, a method for visualization of associations of genes to cell clusters in single-cell transcriptomics data. We developed an R package *APL* implementing this concept. *APL* is freely available and serves to identify and interactively visualize cluster-specific genes. It allows to annotate clusters to cell types using known marker genes, or to generalize from known marker genes to additional, novel ones. It is integrated with Gene Ontology enrichment to further support the annotation process.

Methods

3k PBMC data

The UMI count matrix ("Gene/ cell matrix (filtered)") of peripheral blood mononuclear cells (PBMCs) from a healthy donor[2,3] was downloaded on 02.08.2021 from https://support.10xgenomics.com/single-cell-gene-expression/datasets/1.1.0/pbmc3k and analyzed according to the Seurat Guided Clustering Tutorial available from https://satijalab.org/seurat/articles/pbmc3k_tutorial.html. Below we present the steps of the conducted analysis. Features detected in less than three cells were removed from the data. Additionally, the cells for which less than 200 or more than 2,500 features were detected, as well as cells with expression of mitochondrial genes higher than 5% of total counts, were also removed from the data. The filtered data was then normalized using a method "LogNormalize" from Seurat 4.0 package[5] and linearly transformed using its "ScaleData" function. Subsequently, PCA was performed on the matrix of the 2,000 most variable genes and the first 10 PCs were selected for constructing K-nearest neighbor graph. Next, cells were clustered using the Louvain algorithm (resolution parameter of 0.2) and the UMAP visu-

alization was generated using the first 10 PCs. To match the clusters to known cell types the expression of canonical markers was investigated (Supplementary Figure 1). Finally, correspondence analysis was applied to the normalized UMI count matrix using all 13,713 genes. For generating the Association Plots, the first 223 CA dimensions (number obtained using the "elbow rule") were considered.

Analysis of the human cell atlas of fetal gene expression data

A processed data set with normalized counts from all cells ("Human_RNA_processed.loom") was downloaded on 01.15.2021 from https://descartes.brotmanbaty.org/bbi/human-gene-expression-during-development/.[4] The normalized counts were obtained using the protocol described in the publication.[4]
Correspondence analysis was applied to the normalized count matrix using all genes across 12,106 cells from the stomach clusters. The Association Plots for each of the 16 stomach cell types were generated using the first 4047 CA dimensions as determined by the elbow rule.

R package

The R package *APL* is available from the GitHub repository at https://github.com/VingronLab/APL. The package requires the R program, which is freely available from CRAN at http://cran.r-project.org. For using *APL* we highly recommend installing *pytorch* because it provides a fast implementation of the singular value decomposition. For more details on package installation and usage please refer to the vignette available on GitHub.

Methods for choosing a number of dimensions

To facilitate the choice of an reasonable number of dimensions from input data to retain in the analysis three methods from the literature were implemented in *APL*:

1. *elbow rule*: the number of dimensions to retain is computed from scree plots of randomized data, and corresponds to a point in the scree plot where the original singular values enter the band of randomized singular values,[28]
2. *80% rule*: a minimal number of first dimensions which in total explain above 80% of the total inertia are retained,[29]
3. *average rule*: only those dimensions which account for more inertia than one dimension on average are retained.[29]

In the examples presented in this manuscript the number of dimensions to retain was selected using the *elbow rule*.

Gene set enrichment analysis

The gene set enrichment analysis of the PBMC cell clusters was conducted using the Molecular Signatures Database (MSigDB) v7.2[8] together with the GSEA method[6,7] available from http://www.gsea-msigdb.org/gsea/msigdb/annotate.jsp. The analysis was run using the default parameters and all available MSigDB gene sets. The results were sorted according to the size of the overlap between the input gene set and the gene sets from the MsigDB collection.

GO enrichment analysis

The GO enrichment test of lymphoid cells from the stomach cluster from human cell atlas of fetal gene expression was conducted using the *topGO* package. For this purpose the following parameters were used: algorithm = elim, statistic = fisher, ontology = BP, mapping = org.Hs.eg.db. A set of 358 genes with S_α score above 1 from the Association Plot for lymphoid cells vs other cells from cluster stomach was tested against a gene universe consisting of the remaining genes from the Association Plot. The GO results were sorted afterwards according to Fisher's exact test p-values and the 10 most significantly enriched GO terms were shown in Figure 8.

Differential expression testing tools

To investigate the agreement among results obtained with Association Plots, DESeq2,[22] edgeR,[23,24] and Seurat 4.0,[5] we applied them to each cell type from the 3k PBMC data. The gene rankings from each tool were computed in the following way.
For DESeq2,[22] the analysis was performed by combining DESeq2 package with the zinbwave function from ZINB-WaVE package,[25] as recommended in the DESeq2 vignette from 10/27/2020 for single-cell analysis. For this purpose we applied DESeq2 to zimbwave-weighted count matrix using test="LRT" for significance testing, and the following DESeq arguments: useT = TRUE, minmu = 1e-6, and minReplicatesForReplace = Inf. Finally, the genes were sorted by p-value in increasing order and the genes with non-positive log2 fold-change values were removed from the analysis.
For edgeR,[23,24] the differential expression analysis for each cluster was also performed using the observational weights computed by zinbwave function. We followed the zinbwave vignette from 10/28/2020. The genes were then sorted by p-values in an increasing order, and genes with non-positive log2 fold-change values were discarded.
For Seurat,[5] the gene rankings were computed for each cluster separately using the FindAllMarkers function with the parameters: only.pos = FALSE, min.pct = 0, logfc.threshold = 0, return.thresh = 1.01. The genes were then sorted by p-value in increasing order and the genes with non-negative log2 fold-change values were removed from the analysis.
For Association Plots, genes were ranked by S_α in decreasing order.
To generate the heatmaps from Figure 6 1,000 top genes from each gene ranking were extracted, and the size of the gene overlaps between the rankings was computed.
To generate the Association Plots for the dendritic cells from Figure 7, from each gene ranking we selected 250 genes with the lowest p-values, which passed a log2 fold-change threshold of: 1.9 (DESeq2), 0.5 (Seurat), 1 (edgeR), and highlighted them in the Association Plots.

CRediT authorship contribution statement

Elzbieta Gralinska: Methodology, Software, Writing - original draft, Writing - review & editing. **Clemens Kohl:** Software. **Bita Sokhandan Fadakar:** Software. **Martin Vingron:** Supervision, Conceptualization, Methodology, Writing – review & editing.

DECLARATION OF COMPETING INTEREST

The authors declare that they have no known competing financial interests or personal relationships that could have appeared to influence the work reported in this paper.

Appendix A. Supplementary Data

Supplementary data associated with this article can be found, in the online version, at https://doi.org/10.1016/j.jmb.2022.167525.

Received 30 November 2021;
Accepted 28 February 2022;
Available online 7 March 2022

Keywords:
marker genes;
gene expression;
correspondence analysis;
single-cell data;
Association Plot

Abbreviations:
sc, single-cell; CA, correspondence analysis; PCA, Principal Component Analysis; DE, differential expression

References

1. Gralinska, Elzbieta, Vingron, Martin, (2020). Association plots: Visualizing associations in high-dimensional correspondence analysis biplots. *bioRxiv*.
2. Zheng, Grace X.Y., Terry, Jessica M., Belgrader, Phillip, Ryvkin, Paul, Bent, Zachary W., Wilson, Ryan, Ziraldo, Solongo B., Wheeler, Tobias D., et al., (2017). Massively parallel digital transcriptional profiling of single cells. *Nature Commun.* **8**, 14049.
3. 10x Genomics, (2016, May 26). 3k PBMCs from a Healthy Donor, Single Cell Gene Expression Dataset by Cell Ranger 1.1.0.
4. Cao, Junyue, O'Day, Diana R., Pliner, Hannah A., Kingsley, Paul D., Deng, Mei, Daza, Riza M., Zager, Michael A., Aldinger, Kimberly A., et al., (2020). A human cell atlas of fetal gene expression. *Science* **370** (6518)
5. Hao, Yuhan, Hao, Stephanie, Andersen-Nissen, Erica, Mauck, William M., Zheng, Shiwei, Butler, Andrew, Lee, Maddie J., Wilk, Aaron J., et al., (2020). Integrated analysis of multimodal single-cell data. *bioRxiv*.
6. Subramanian, Aravind, Tamayo, Pablo, Mootha, Vamsi K., Mukherjee, Sayan, Ebert, Benjamin L., Gillette, Michael A., Paulovich, Amanda, Pomeroy, Scott L., et al., (2005). Gene set enrichment analysis: a knowledge-based approach for interpreting genome-wide expression profiles. *Proc. Natl. Acad. Sci.* **102** (43), 15545–15550.
7. Mootha, Vamsi K., Lindgren, Cecilia M., Eriksson, Karl-Fredrik, Subramanian, Aravind, Sihag, Smita, Lehar, Joseph, Puigserver, Pere, Carlsson, Emma, et al., (2003). Pgc-1α-responsive genes involved in oxidative phosphorylation are coordinately downregulated in human diabetes. *Nature Genet.* **34** (3), 267–273.
8. Liberzon, Arthur, Birger, Chet, Thorvaldsdóttir, Helga, Ghandi, Mahmoud, Mesirov, Jill P., Tamayo, Pablo, (2015). The molecular signatures database hallmark gene set collection. *Cell Syst.* **1** (6), 417–425.
9. Uhlén, Mathias, Fagerberg, Linn, Hallström, Björn M., Lindskog, Cecilia, Oksvold, Per, Mardinoglu, Adil, Sivertsson, Åsa, Kampf, Caroline, et al., (2015). Tissue-based map of the human proteome. *Science* **347** (6220), 1260419.
10. Uhlen, Mathias, Karlsson, Max J., Zhong, Wen, Tebani, Abdellah, Pou, Christian, Mikes, Jaromir, Lakshmikanth, Tadepally, Forsström, Björn, et al., (2019). A genome-wide transcriptomic analysis of protein-coding genes in human blood cells. *Science* **366** (6472)
11.. Wang, Yanbo, Liu, Jing, Ren, Fenghai, Chu, Yanjie, Cui, Binbin, (2021). Identification and validation of a four-long non-coding rna signature associated with immune infiltration and prognosis in colon cancer. *Front. Genet.* **12**
12. Li, J., Guo, H., Ma, Y., Chen, H., Qiu, M., (2021). 11p linc00926 is a b cell-specific long non-coding rna in lung adenocarcinoma and is associated with the prognosis of patients with this disease. *J. Thoracic Oncol.* **16** (4), S703.
13. Chu, Zhong, Huo, Nan, Zhu, Xiang, Liu, Hanxiao, Cong, Rui, Ma, Luyuan, Kang, Xiaofeng, Xue, Chunyuan, et al., (2021). Foxo3a-induced linc00926 suppresses breast tumor growth and metastasis through inhibition of pgk1-mediated warburg effect. *Mol. Ther.*.
14. Wang, Fangce, Tian, Xiaoxue, Zhou, Jie, Wang, Guangming, Yu, Wenlei, Li, Zheng, Fan, Zhuoyi, Zhang, Wenjun, et al., (2018). A three-lncrna signature for prognosis prediction of acute myeloid leukemia in patients. *Mol. Med. Rep.* **18** (2), 1473–1484.
15. Liang, Yuexiong, Zhu, Haifeng, Chen, Jing, Lin, Wei, Li, Bing, Guo, Yusheng, (2020). Construction of relapse-related lncrna-mediated cerna networks in hodgkin lymphoma. *Arch. Med. Sci. AMS* **16** (6), 1411.
16. Sellers, Subhashini A., Fischer, William A., Heise, Mark T., Schughart, Klaus, (2021). Highly dampened blood transcriptome response in hiv patients after respiratory infection. *Sci. Rep.* **11** (1), 1–7.
17.. Brinas, François, Danger, Richard, Brouard, Sophie, (2021). Tcl1a, b cell regulation and tolerance in renal transplantation. *Cells* **10** (6), 1367.
18. Aggarwal, Mohit, Villuendas, Raquel, Gomez, Gonzalo, Rodriguez-Pinilla, Socorro M., Sanchez-Beato, Margarita, Alvarez, David, Martinez, Nerea, Rodriguez, Antonia, et al., (2009). Tcl1a expression delineates biological and clinical variability in b-cell lymphoma. *Mod. Pathol.* **22** (2), 206–215.
19. Van den Berge, Koen, Perraudeau, Fanny, Soneson, Charlotte, Love, Michael I., Risso, Davide, Vert, Jean-Philippe, Robinson, Mark D., Dudoit, Sandrine, et al., (2018). Observation weights unlock bulk rna-seq tools for zero inflation and single-cell applications. *Genome Biol.* **19** (1), 1–17.
20. Wang, Tianyu, Li, Boyang, Nelson, Craig E., Nabavi, Sheida, (2019). Comparative analysis of differential gene expression analysis tools for single-cell rna sequencing data. *BMC Bioinformatics* **20** (1), 1–16.
21.. Luecken, Malte D., Theis, Fabian J., (2019). Current best practices in single-cell rna-seq analysis: a tutorial. *Mol. Syst. Biol.* **15** (6), e8746.
22. Love, Michael I., Huber, Wolfgang, Anders, Simon, (2014). Moderated estimation of fold change and dispersion for rna-seq data with deseq2. *Genome Biol.* **15** (12), 1–21.
23.. Robinson, Mark D., McCarthy, Davis J., Smyth, Gordon K., (2010). edger: a bioconductor package for differential expression analysis of digital gene expression data. *Bioinformatics* **26** (1), 139–140.
24. McCarthy, Davis J., Chen, Yunshun, Smyth, Gordon K., (2012). Differential expression analysis of multifactor rna-

seq experiments with respect to biological variation. *Nucl. Acids Res.* **40** (10), 4288–4297.

25. Risso, Davide, Perraudeau, Fanny, Gribkova, Svetlana, Dudoit, Sandrine, Vert, Jean-Philippe, (2018). A general and flexible method for signal extraction from single-cell rna-seq data. *Nat. Commun.* **9** (1), 1–17.
26. Alexa, Adrian, & Rahnenfuhrer, Jorg (2021). topGO: Enrichment Analysis for Gene Ontology. R package version 2.46.0.
28. Ciampi, Antonio, Marcos, Ana González, Limas, Manuel Castejón, (2005). Correspondence analysis and 2-way clustering. *SORT* **29** (1)
29. Greenacre, Michael J., Blasius, Jörg, (1994). Correspondence analysis in the social sciences: Recent developments and applications. Academic Press, London.
