# Supplementary material for: PDBx/mmCIF Ecosystem: Foundational Semantic Tools for Structural Biology
Source: J Mol Biol. Author manuscript; Available in PMC 2023 Jun 26. (PMC10292674; doi:10.1016/j.jmb.2022.167599)
Supplement: Article [file NIHMS1907597-supplement-Article.zip › The-PCDDB--Protein-Circular-Dichroism-Data-Bank---A-Bioinf_2022_Journal-of-M.pdf]

Research Article

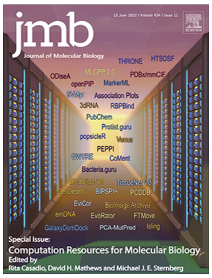

The PCDDB (Protein Circular Dichroism Data Bank): A Bioinformatics Resource for Protein Characterisations and Methods Development

Sergio Gomes Ramalli [1,†], Andrew John Miles [1,†], Robert W. Janes [2,*] and B. A. Wallace [1,*]

1 - ***Institute of Structural and Molecular Biology,*** *Birkbeck, University of London, Malet Street, London WC1E 7HX, UK*
2 - ***School of Biological and Behavioural Sciences,*** *Queen Mary University of London, London E1 4NS, UK*

Correspondence to Robert W. Janes and B.A. Wallace: *r.w.janes@qmul.ac.uk (R.W. Janes), b.wallace@mail.cryst.bbk.ac.uk (B.A. Wallace)*
https://doi.org/10.1016/j.jmb.2022.167441
Edited by Michael Sternberg

Abstract

The Protein Circular Dichroism Data Bank (PCDDB) [https://pcddb.cryst.bbk.ac.uk] is an established resource for the biological, biophysical, chemical, bioinformatics, and molecular biology communities. It is a freely-accessible repository of validated protein circular dichroism (CD) spectra and associated sample and metadata, with entries having links to other bioinformatics resources including, amongst others, structure (PDB), AlphaFold, and sequence (UniProt) databases, as well as to published papers which produced the data and cite the database entries. It includes primary (unprocessed) and final (processed) spectral data, which are available in both text and pictorial formats, as well as detailed sample and validation information produced for each of the entries. Recently the metadata content associated with each of the entries, as well as the number and structural breadth of the protein components included, have been expanded. The PCDDB includes data on both wild-type and mutant proteins, and because CD studies primarily examine proteins in solution, it also contains examples of the effects of different environments on their structures, plus thermal unfolding/folding series. Methods for both sequence and spectral comparisons are included. The data included in the PCDDB complement results from crystal, cryo-electron microscopy, NMR spectroscopy, bioinformatics characterisations and classifications, and other structural information available for the proteins via links to other databases. The entries in the PCDDB have been used for the development of new analytical methodologies, for interpreting spectral and other biophysical data, and for providing insight into structures and functions of individual soluble and membrane proteins and protein complexes.

© 2022 Elsevier Ltd. All rights reserved.

Introduction

The Protein Circular Dichroism Data Bank (PCDDB)[1,2] [https://pcddb.cryst.bbk.ac.uk] is a public archive for the deposition and dissemination of circular dichroism (CD) and synchrotron radiation circular dichroism (SRCD) spectra and their associated metadata. Inspired by the availability of other online resources such as the Protein Data Bank (PDB) structural database,[3] the UniProt sequence data base,[4] and the Enzyme Classification IntEnz database (EC),[5] the PCDDB was first released in 2010 as an accession-only database comprising widely-used CD reference spectra.[6] Later[2] the facility for external user depositions was added, enabling authors of papers containing CD data to make their data and metadata publically-available for use in other analyses and comparisons. Since then the PCDDB has been in continuous use (mostly for data downloads). However, increasingly,

0022-2836/© 2022 Elsevier Ltd. All rights reserved.

a number of publishers (such as the Nature Publishing Group and PLOS journals) recommend authors make data associated with their publications available in the PCDDB. The PCDDB is also a recommended repository by the beta.fairsharing.org and re3data.org websites. Moreover, a number of research councils/organisations (for example, the UK Biotechnology and Biological Sciences Research Council (BBSRC)) list it as a notable resource. It has been cited >200 times by papers that either produce or use the data therein, including papers describing methodological developments, and experimental and bioinformatics papers which use the PCDDB data for comparisons. It now incorporates and interfaces with a full suite of programmes for data processing, analyses, and display of CD spectroscopic data (Figure 1).

Access to data in the PCDDB is freely available (without registration or password requirements), and all datasets are downloadable via the website. However, author registration is required for deposition of data in order to ensure good practice and traceability of the data. Registration also enables users to perform more complex searches, plus logging and saving of repeat searches, and to receive notification of updates to the website contents or functions.

Results and Discussion: PCDDB Features

All PCDDB entries include a fully-processed (final) spectrum displayed in standard units of delta epsilon or mean residue ellipticity (MRE). They may also include the individual and averaged sample and baseline spectra (so the reproducibility can be seen), the net (averaged sample minus averaged baseline) spectrum and the associated high tension (HT) (also known as dynode) spectra, which are correlated with the sample absorbance signals, and are important for validity checks as well as for methods development. This full gamut of original data allows tests to be automatically carried out by the associated validation software ValiDichro.[7] The validation check results are provided to the depositor prior to release of the entry as a guide to potential issues and means of modifying the entry, and then, when the entry is released, to the user as a guide to the quality of the data. They also provide information essential for good practice and traceability and other types of methods development. Metadata for each entry (also monitored by ValiDichro for consistency and completeness) includes experimental conditions and details of the protein sample.

Entries also include links to the PDB,[3] UniProt,[4] the CATH[8] protein structure classification database, the AlphaFold protein structure database,[9,10] the IntEnz enzyme classification database[5] (where appropriate), and, (when provided by the authors) citations to the published article describing the original work that produced the data, plus keywords, which can be used to group and search for associated entries.

The accession/search features allow data series such as those produced in stability studies (e.g. thermal unfolding (melts) or pH changes), to be grouped together for easy access. Users can also identify (and download) spectral components of specially-developed reference datasets, comprising of a broad base of soluble proteins (designated SP175)[11] or membrane proteins (designated SMP180)[12] for bioinformatics develop-

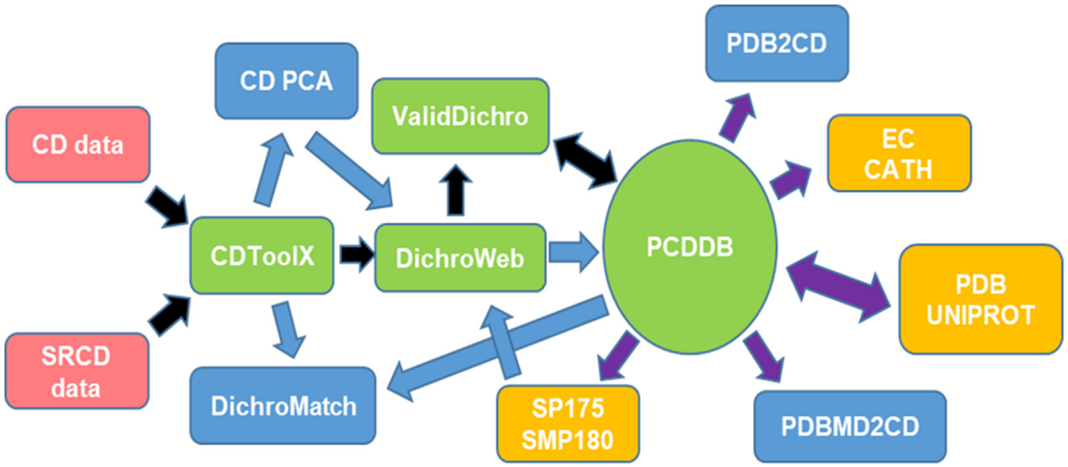

Figure 1. Schematic diagram indicating the relationships and data flow between the PCDDB, sister tools, and linked resources. Colour Codes: Green boxes: Associated core processing, validation and analysis tools and resources produced by the PCDDB team, Blue boxes: Associated analysis tools produced by the PCDDB team, Red boxes: Input experimental data (provided by the user), Orange boxes: Bioinformatics resources/sister tools, Black arrows: Main workflow, Blue arrows: alternative workflow to/from other tools; Purple arrows: Output to/from other resources.

ments. Spectra can be downloaded in ASCII format, either with or without the associated metadata; alternatively the entire contents of the database can be downloaded as a single compressed archive file. The former is commonly used for comparison studies and the latter for bioinformatics/software developments (for references see “Examples of Recent Applications of the PCDDB” Section below).

Since the most recent publication describing the PCDDB,[2] the website which provides access to the data bank has undergone significant redesign to improve user access, visualisation and file download and deposition, as well as enhancements to the information content of the entries. As a result of new collaborations and coordination with groups creating and maintaining other bioinformatics tools, each entry is now provided with two-way links to the PDB[3] and UniProt[4] databases (the latter enabled via a partnership with the ELIXIR consortium).[13] Recently the PCDDB has been included in the Google Bioschema network/dataset of bioinformatics resources and the DisProt RDMkit for intrinsically disordered proteins.[14]

The schematic diagram in Figure 1 shows the relationship between the PCDDB and other related tools (ValiDichro,[7] DichroMatch,[15] PDB2CD,[16] PDBMD2CD,[17] CDToolX,[18] and the DichroWeb analysis website[19] created by the PCDDB consortium, plus other linked resources: (PDB,[3] UniProt[4], and the CATH[8] and IntEnz[5] classification databases.[8]

New Developments in the PCDDB

Contents, organisation, and links

Developments since the previous publications about the PCDDB[1,2] include additional entries, identification and grouping of entries that comprise the SP175[11] and SMP180[12] reference databases, inclusion of thermal-melt unfolding series (entries in a thermal melt series are given related subcodes so they can be treated as a single experiment accessed from a single page (for example, see https://pcddb.cryst.bbk.ac.uk/series/CD0004000)), plus links of individual entries to external resources such as the PDB[3] and UniProt.[4] Upgrades include enhancements to the usability, display (Figure 2) and searchability of both individual components and the entire contents of the data bank, links for downloading the SP175,[11] SMP180[12] datasets (on the sidebar which is visible on all PCDDB webpages), and links to new associated tools.

Links to the back-calculation programmes PDB2CD[16] and PDBMD2CD[17] which enable the prediction of CD spectra for proteins with known structures (i.e. from their PDB coordinates), and to popular sites for the calculation of protein secondary structures from CD spectra, such as DichroWeb,[19] BeStSel[20], SESCA[21] and K3D3[22] are now included.

Search features

Searchability has been enhanced with a new ‘quick’ search bar equipped with an extensive dropdown list of search criteria (for example: protein name, protein type, or UniProt ID). An “advanced search page” with multiple search criteria, accessible via the side bar, now enables more targeted interrogations of the database. It can accept up to six criteria for a single search. For example, a search could be undertaken for all SRCD spectra of proteins from a specific organism with greater than 50% helix content, measured at a temperature of 20 degrees C.

Functions

The DichroMatch[15] spectral matching tool (Figure 3(a)) has been integrated into the PCDDB website and enables (based on several different methods) the identification of proteins with similar spectral characteristics as a query protein. The user can select from a choice of up to four comparison methods, with the results of each being displayed on the same plot (Figure 3(a)), along with a table including the normalised root mean square deviation (NRMSD)[19] between the spectra; this parameter provides a quantitative measure of the similarity between each of the identified “matched” spectra and the query spectrum. Another new feature of the DichroMatch facility enables the user to temporarily upload a spectrum, which is not already deposited in the database, so that it can be visually compared to a query spectrum in the database. The query and uploaded spectra are automatically scaled to the same maximum or minimum magnitude. This function can be particularly useful, for example, for comparisons of spectra of the same protein under different conditions or to scrutinise the effect of a mutation on the spectral characteristics.

There is also a completely new search facility included in the PCDDB (described for the first time here) enabling the user to undertake a Blastp[23] comparison (indicated as ‘sequence search’ on the PCDDB website front page side bar), for identification of spectra of sequence-related (rather than spectral-related) proteins (Figure 3(b)). This is orthogonal to the DichroMatch search function, in that it allows users to identify the spectra of proteins in the PCDDB that closely resemble a query protein, based on their sequence identities. As the numbers of proteins in the PCDDB are limited, the Blastp “short sequence” default parameter terms are used for the search,[23] although the user can define their own minimum cut-off percentage level for matching sequences. The output generated (Figure 3(b)) is a CD spectral overlay plot of all those entries found in the database with sequences matching within the specified parameters, plus a table of the PCDDB codes and names of those entries, their UniProt and PDB (if any) accession codes, protein class (e.g., soluble or mem-

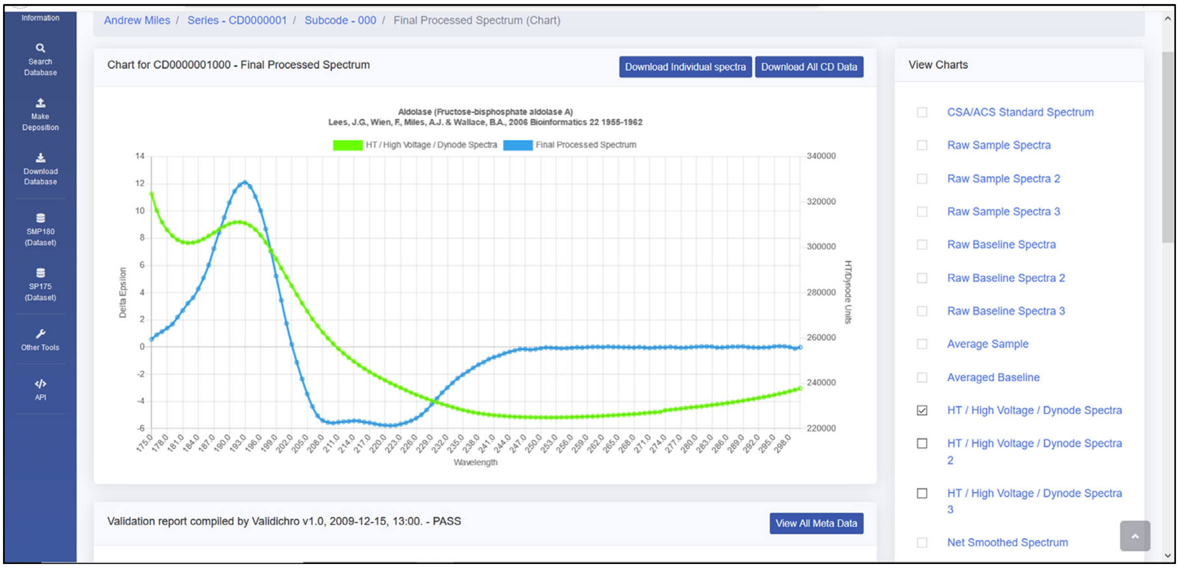

Figure 2. The Main Spectral Chart Display Page. Single or multiple CD (blue) spectra (either the raw data or the processed data) and the HT signal (green) can be viewed on the same plot. This can be used to give insight into the reliability and wavelength cutoff of the data, as described in the text. The figure shows the final processed SRCD spectrum of the protein aldolase and its HT signal. On the left is the sidebar, which is shown on every page of the PCDDB website, with links as described in the text; on the right is the list of spectra associated with this entry (raw data, CSA spectra, etc.) which can also be selected to be shown on the chart using the tick boxes.

brane), the percentage sequence identity, the sequence alignment length, and the percent of the sequence in the alignment. This new PCDDB feature provides a unique new method for identifying spectra of structural homologues.

Improved and enhanced spectral plots

Spectral plots have been redeveloped for better visualisation and there is now an option to view either the processed CD spectrum (or the raw spectra) and the HT (dynode) signal (which is proportional to the sample absorbance and therefore an indication of the data reliability) on the same plot using two y-axes as shown in Figure 2(a). Similarly, all repeat scans of unprocessed data can be viewed simultaneously to reveal, for example, any significant variations between them, or any spectral features (artifacts) that occur in just one of them. Thus the full visual inspection of data essential for ascertaining that the final processed spectrum is accurate and reliable is now available to both the depositor and the user (as long as the individual spectral data have been deposited for the entry, which is strongly encouraged). Another similar display upgrade means that all spectra constituting a data series (for example, a thermal melt) can also be displayed simultaneously.

Deposition procedure improvements

Data file formats enabled are those generated by most (benchtop) CD and SRCD instruments, along with the files produced by the data processing applications CDTool[24] and CDToolX[18] (Figure 1), and user-created text files with data in pre-defined formats. Only the final fully-processed CD spectrum is essential for the deposition; however, the inclusion of raw (unprocessed) data files (ie., repeat scans of the sample and baseline) is encouraged, and the procedures to do this have been simplified, with each file now uploadable separately rather than, as previously, in a single concatenated file, which had to be created by the user. This modification is aimed at encouraging users to include raw (unprocessed) data files of each entry for completeness, good practice and tracability. It also facilitates certain data cross-validation functions undertaken by the ValiDichro software[7] and allows public inspection of the complete set of experimental data. Multiple depositions of related data, for example a series of spectra from a thermal melt, have also been simplified by a duplication function which allows the user to copy those sections of the metadata, chosen by the user, to a new entry with a consecutive accession code (PCDDBid). The new PCDDBid can either have the same headcode (first 7 digits) with a new subcode (useful for data series) or new, but consecutive, headcodes for different spectra, not in a series, but related to the same experiment.

Validation features

Validation is carried out using the methods available in the ValiDichro server.[7] Both data

A

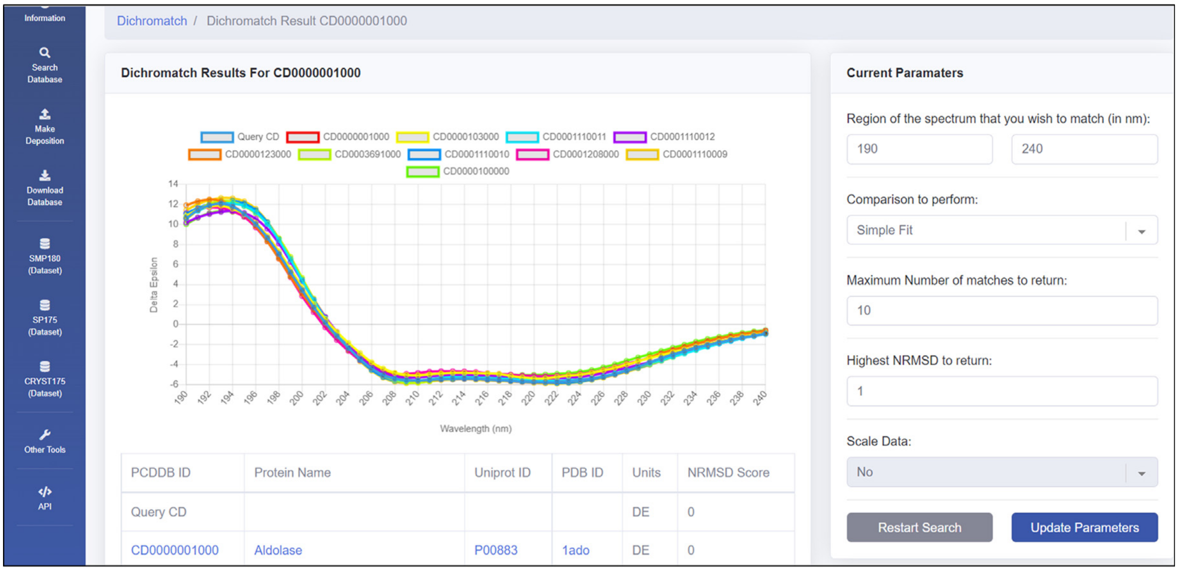

B

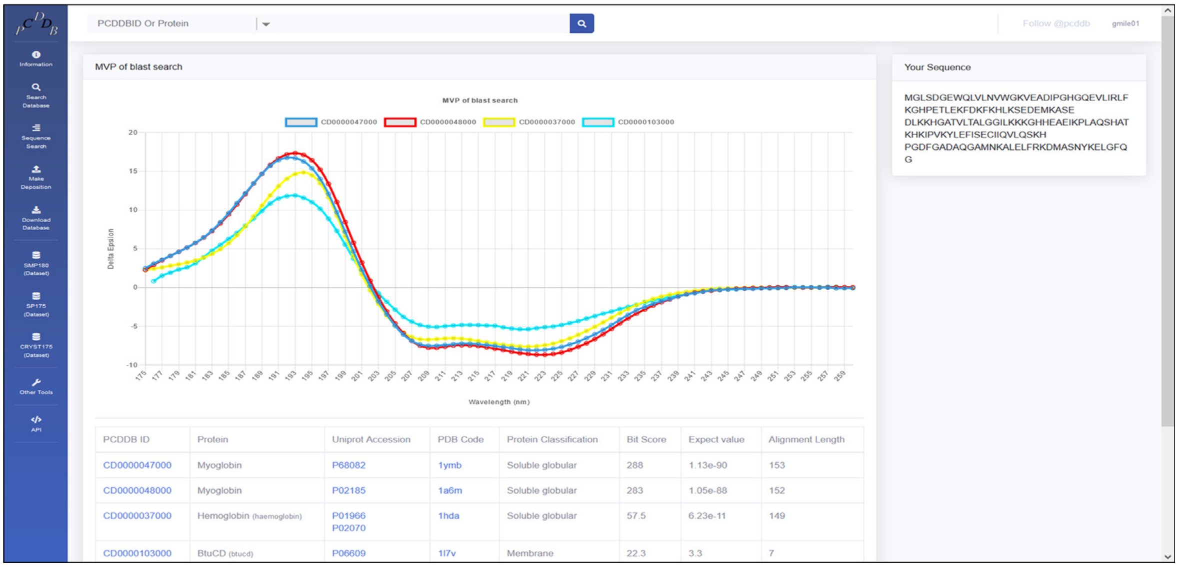

Figure 3. Analysis Functions. (a) (Top) DichroMatch (Spectral Match Function): Screen shot of the results of a search for spectra in the PCDDB (shown in the middle panel in multi-colours) that are similar to a query spectrum [in this case, aldolase] (shown in blue) using the parameters set in the right hand panel, with numerical values and identities of the matches listed below the figure. This illustrates the DichroMatch feature[13] which has now been integrated into the PCDDB so it can assess all database entries as possible matches. To the left is the sidebar (blue background), which is shown on every page and which lists the links described in the text; on the right is the list of search parameters that were used. (b) (Bottom) - Blastp (Sequence Match Function): Illustration of the new identification function, which identifies near neighbours using the sequence-matching BLASTp algorithm. This function is selected from the left hand sidebar of the website landing page. The sequence of human myoglobin (UniProt code P02144) (listed in the right hand panel) was submitted either in FASTA format or as a user-uploaded text file. The matched results in the PCDDB (listed in the table below the plot, with their spectra overlaid in the plot) are of two myoglobins (from sperm whale and horse), and bovine haemoglobin. The right hand column of the table indicates the percentage sequence match of the query proteins to the identified matched proteins (in this case between 97 and 100%). This new tool provides a way of identifying spectral features for an unknown protein based on proteins with similar sequences.

and metadata are checked for consistency, and any parameters that are unusual are highlighted as they may indicate experimental or data processing error issues. For example, an error in concentration may be highlighted in the final processed spectrum as having an unusually small or large spectral magnitude, or data may be distorted because the absorption of the sample is too high, as indicated by the HT signal. Validation can be carried out multiple times prior to submitting a deposition, allowing errors/inconsistencies to be identified and rectified, or, in the case of a serious issue, the experiment to be repeated under more appropriate conditions.

New information and help functions

Help pages are easily accessed via the information sidebar which is visible on the left hand side of all pages. Links are included to citations and to the PCDDB YouTube channel (https://www.youtube.com/user/ThePcddb/videos), which contains video tutorials on many aspects of protein CD spectroscopy, including how to deposit data into the PCDDB. There is also a link to the PCDDB Twitter page (https://twitter.com/pcddb), which includes live website status updates.

Findability and sharing of data with partners

Updates to the PCDDB also include improvements to the findability, accessibility, interoperability, and reusability of the circular dichroism data and associated metadata. These features were enabled by new collaborations with the DisProt server (https://disprot.org) produced by the ELIXIR Intrinsically Disordered Protein (IDP) community,[14] and the UniProt database,[4] using Bioschema and the PCDDB's "restful" API design that make it easier to access the PCDDB's data programmatically (ie., enabling the user to write scripts in (Python, Perl etc.) to extract data).

Examples of Recent Applications of the PCDDB

A wide range of studies have recently used spectra available in the PCDDB for comparisons in biological studies, including an investigation into the influence of nano-agents that could be used in radiotherapy on the structure and stability of human serum albumin,[25] a comparative study of lungfish myoglobins which display a significant functional diversity,[26] an investigation of the response of the metalloregulatory protein, CueR, which controls the concentration of copper ions in cells to environmental changes in solution,[27] a study of abiotic cofactor assembly in photosynthetic biomimetics,[28] and an investigation of how iron redox state affects aggregation of alpha-synuclein in the brain.[29]

Data from the PCCDB has also been valuable as an aid for developing computational tools. For example, a method of atomistically refining the structural ensemble of intrinsically-disordered peptides was facilitated by experimental measurements using circular dichroism spectroscopy, with the PCDDB being used as a source of 411 proteins with known structures and associated CD spectra for validating the results of this investigation.[30] In other studies, a Bayesian approach to secondary structure prediction[31] was investigated using the 71 SRCD spectra present in the SP175 dataset available in the PCDDB. A study using accelerated molecular dynamics methods was validated by reverse calculation of CD data, where 107 protein spectra from the PCDDB were used to validate the results,[32] and spectra prediction from classical electromagnetic theory was facilitated specifically using the specialist SRCD spectra that are available in the PCDDB.[33]

PCDDB data have also been used for regulatory purposes. For example, a series of investigations used data from the PCDDB to determine the uncertainties in the magnitude and wavelength positions of peaks inherent in protein CD data,[34,35] which is information that is vital for good practice and standardisation in the pharmaceutical industry.

Finally, a number of online resources have utilised data available in the PCDDB to develop new software, including the novel secondary structure analyses tool, BeStSel[20] (a computational tool with methods orthogonal to those in the DichroWeb analysis method),[19] and the CD spectral prediction tools PDB2CD,[17] PDBMD2CD,[18] and SESCA[21] (which generate CD spectra when presented with the PDB code of a protein crystal or NMR structure). All of these tools employ datasets which include spectra from the SP175 and/or SMP180 datasets and other proteins for their calculations.

Conclusions

The PCDDB has become a well-used, freely-accessible resource for protein structure and bioinformatics studies. Recent updates have aimed to improve the searchability, accessibility, interoperability, and reusability of circular dichroism spectroscopic data and associated metadata with sequence and structure data obtained through links with other databases, plus partner websites and resources, including the DichroMatch[15] and DichroWeb servers,[19] the UniProt[4] and PDB[3] databases, Bioschema,[14] Google, and the IDP Elixer community.[13] Following integration of links with the CATH protein structure classification database[8] and enyzme classification[5] databases, the reach and interoperability of PCDDB's curated data continues to increase.

In summary, the PCDDB has been established as a central resource for the development of new bioinformatics methods and for comparisons in structural biology and biochemistry based on CD

and SRCD spectroscopic data. It provides a valuable resource to the biophysical and biochemical communities for whom circular dichroism spectroscopy has become a well-used and standard methodology.[36,37]

Contributions

The concept for the PCDDB was originally created by B.A. Wallace and R.W. Janes, and developed and implemented over the past 11 years by L. Whitmore and A.J. Miles, with associated applications contributions developed by E.D. Drew, B. Woollett, R.W. Janes, and L. Mavridis. More recent website developments have been designed and implemented by S.G. Ramalli, A.J. Miles, and R.W. Janes.

Acknowledgements

This work has been supported by a series of grants from the Bioinformatics and Biological Resources Fund of the U.K. Biotechnology and Biological Research Council [BBSRC], most recently grant P024092 to BAW, and grant P024106 to RWJ.

Declaration of Competing Interest

The authors declare that they have no known competing financial interests or personal relationships that could have appeared to influence the work reported in this paper.

Received 21 November 2021;
Accepted 1 January 2022;
Available online 6 January 2022

Keywords:
circular dichroism spectroscopy;
protein structural and spectroscopic database;
data deposition and accession;
protein stability and environmental effects;
resource for bioinformatics developments

† These authors contributed equally to this paper.

Abbreviations used:
CD, circular dichroism; IDP, intrinsically disordered protein; MRE, mean residue ellipticity; NMR, nuclear magnetic resonance; NRMSD, normalised root mean square deviation; PCDDB, Protein Circular Dichroism Data Bank; PCDDBid, unique acquisition code for a PCDDB entry; PDB, Protein Data Bank; SRCD, synchrotron radiation circular dichroism; HT, high tension; IntEnz, enzyme classification

References

1. Whitmore, L., Woollett, B., Miles, A.J., Klose, D.P., Janes, R.W., Wallace, B.A., (2011). PCDDB: The Protein Circular Dichroism Data Bank, a repository for circular dichroism spectral and metadata. *Nucleic Acids Res.* **39**, D480–D486.
2. Whitmore, L., Miles, A.J., Mavridis, L., Janes, R.W., Wallace, B.A., (2017). PCDDB: New developments at the Protein Circular Dichroism Data Bank. *Nucleic Acids Res.* **45**, D303–D307.
3. Burley, S.K., Bhikadiya, C., Bi, C., Bittrich, S., Chen, L., Crichlow, G.V., Christie, C.H., Dalenberg, K., et al., (2021). RCSB Protein Data Bank: Powerful new tools for exploring 3D structures of biological macromolecules for basic and applied research and education in fundamental biology, biomedicine, biotechnology, bioengineering and energy sciences. *Nucleic Acids Res.* **49**, D437–D451.
4. The UniProt Consortium, (2021). UniProt: The universal protein knowledgebase in 2021. *Nucleic Acids Res.* **49**, D480–D489.
5. Fleischmann, A., Darsow, M., Degtyarenko, K., Fleischmann, W., Boyce, S., Axelsen, K.B., Bairoch, A., Schomburg, D., Tipton, K.F., Apweiler, R., (2004). IntEnz, the integrated relational enzyme database. *Nucleic Acids Res.* **32**, D434–D437.
6. Whitmore, L., Woollett, B., Miles, A.J., Janes, R.W., Wallace, B.A., (2010). The Protein Circular Dichroism Data Bank, A web-based site for access to circular dichroism spectroscopic data. *Structure* **18**, 1267–1269.
7. Woollett, B., Whitmore, L., Janes, R.W., Wallace, B.A., (2013). ValiDichro: a website for validating and quality control of protein circular dichroism spectra. *Nucleic Acids Res.* **41**, W417–W421.
8. Sillitoe, I., Lewis, T.E., Cuff, A., Das, S., Ashford, P., Dawson, N.L., Furnham, N., Laskowski, R.A., et al., (2015). CATH: comprehensive structural and functional annotations for genome sequences. *Nucleic Acids Res.* **43**, D376–D381.
9. Jumper, J., Evans, R., Pritzel, A., Green, T., Figurnov, M., Ronneberger, O., Tunyasuvunakool, K., Bates, R., et al., (2021). Highly accurate protein structure prediction with AlphaFold. *Nature* **596**, 583–589.
10. Varadi, M., Anyango, S., Deshpande, M., Nair, S., Natassia, C., Yordanova, G., Yuan, D., Stroe, O., et al., (2021). AlphaFold Protein Structure Database: massively expanding the structural coverage of protein-sequence space with high-accuracy models. *Nucleic Acids Res.* https://doi.org/10.1093/nar/gkab1061.
11. Lees, J.G., Miles, A.J., Wien, F., Wallace, B.A., (2006). A reference database for circular dichroism spectroscopy covering fold and secondary structure space. *Bioinformatics* **22**, 1955–1962.
12. Abdul-Gader, A., Miles, A.J., Wallace, B.A., (2011). A reference dataset for the analyses of membrane protein secondary structures and transmembrane residues using circular dichroism spectroscopy. *Bioinformatics* **27**, 1630–1636.
13. Davey, N.E., Babu, M.M., Blackledge, M., Bridge, A., Capella-Gutierrez, S., Dosztanyi, Z., Drysdale, R., Edwards, R.J., et al., (2019). An intrinsically disordered proteins community for ELIXIR.. *F1000Research* **8**, 1753.

14. Hatos, A., Hajdu-Soltész, B., Monzon, A.M., Palopoli, N., Álvarez, L., Aykac-Fas, B., Bassot, C., Benítez, G.I., et al., (2020). DisProt: Intrinsic protein disorder annotation in 2020. *Nucleic Acids Res.* **48**, D269–D276.
15. Klose, D.P., Wallace, B.A., Janes, R.W., (2012). DichroMatch: A website for similarity searching of circular dichroism spectra. *Nucleic Acids Res.* **40**, W547–W552.
16. Mavridis, L., Janes, R.W., (2017). PDB2CD: A web-based application for the generation of circular dichroism spectra from protein atomic coordinates. *Bioinformatics* **33**, 56–63.
17. Drew, E.D., Janes, R.W., (2020). PDBMD2CD: Providing predicted protein circular dichroism spectra from multiple molecular dynamics-generated protein structures. *Nucleic Acids Res.* **48**, W17–W24.
18. Miles, A.J., Wallace, B.A., (2018). CDToolX, A downloadable software package for processing and analyses of circular dichroism spectroscopic data. *Protein Sci.* **27**, 1717–1722.
19. Miles, A.J., Ramalli, S.G., Wallace, B.A., (2021). DichroWeb, a website for calculating protein secondary structure from circular dichroism spectroscopic data. *Protein Sci.*. https://doi.org/10.1002/pro.4153.
20. Micsonai, A., Wien, F., Bulyáki, E., Kun, J., Moussong, E., Lee, Y.H., Goto, Y., Réfrégier, M., et al., (2018). BeStSel: A web server for accurate protein secondary structure prediction and fold recognition from the circular dichroism spectra. *Nucleic Acids Res.* **46**, W315–W322.
21. Nagy, G., Maxim, I., Jones, N.J., Hoffmann, S.V., Grubmüller, H., (2019). SESCA: Predicting circular dichroism spectra from protein molecular structures. *J. Chem. Theory Comp.* **15**, 5087–5102.
22. Louis-Jeune, C., Andrade-Navarro, M.A., Perez-Iratxeta, C., (2012). Prediction of protein secondary structure from circular dichroism using theoretically derived spectra. Proteins: Struct. *Funct. Bioinf.* **80**, 374–381.
23. Camacho, C., Coulour, G., Avagya, V.M.N., Papadopoulos, J., Bealer, K., Madden, T.L., (2009). BLAST+: Architecture and applications. *BMC Bioinformatics* **10**, 421
24. Lees, J.G., Smith, B.R., Wien, F., Miles, A.J., Wallace, B. A., (2004). CDTool – An integrated software package for circular dichroism spectroscopic data processing, analysis and archiving. *Anal. Biochem.* **332**, 285–289.
25. Yang, X., Bolsa-Ferruz, M., Maric, L., Porcel, E., Salado-Leza, D., Lux, F., Tillement, O., Renault, J.-P., Pin, S., Wien, F., Lacombe, S., (2020). Human serum albumin in the presence of aguix nanoagents: Structure stabilisation without direct interaction. *Int. J. Mol. Sci.* **21**, 4673–4689.
26. Leudemann, J., Fago, A., Falke, S., Wisniewsky, M., Schneider, I., Fabrizius, A., Burmester, T., (2019). Genetic and functional diversity of the multiple lungfish myoglobins. *FEBS Letters* **287**, 1598–1611.
27. Balogh, R.K., Németh, E., Jones, N.C., Vrønning Hoffmann, S., Jancsó, A., Gyurcsik, B., (2021). A study on the secondary structure of the metalloregulatory protein, CueR: Effect of pH, metal ions and DNA. *Eur. Biophys. J.* **50**, 491–500.
28. Ponomarenko, N.S., Kokhan, O., Pokkuluri, P.R., Mulfort, K.L., Tiede, D.M., (2020). Examination of abiotic cofactor assembly in photosynthetic biomimetics: Site-specific stereoselectivity in the conjugation of a ruthenium (II) tris (bipyridine) photosensitizer to a multi-heme protein. *Photosynth. Res.* **143**, 99–113.
29. Abeyawardhane, D.L., Fernández, R.D., Murgas, C.J., Heitger, D.R., Forney, A.K., Crozier, M.K., Lucas, H.R., (2018). Iron redox chemistry promotes antiparallel oligomerization of α-synuclein. *J. Am. Chem. Soc.* **140**, 5028–5032.
30. Ezerski, J.C., Zhang, P., Jennings, N.C., Waxham, M.N., Cheung, M.S., (2020). Molecular dynamics ensemble refinement of intrinsically disordered peptides according to deconvoluted spectra from circular dichroism. *Biophys. J.* **118**, 1665–1678.
31. Spencer, S.E.F., Rodger, A., (2021). Bayesian inference assessment of protein secondary structure analysis using circular dichroism data – how much structural information is contained in protein circular dichroism spectra? *Anal. Methods* **13**, 359–368.
32. Granados-Ramírez, C.G., Carbajal-Tinoco, M.D., (2020). Secondary structure specified polarizabilities of residues for an evaluation of circular dichroism spectra of proteins. *J. Chem. Phys.* **153**, 155101
33. Khare, H., Dey, D., Madhu, C., Senapati, D., Raghothama, S., Govindaraju, T., Ramakumar, S., (2017). Conformational heterogeneity in tails of DNA-binding proteins is augmented by proline containing repeats. *Mol. BioSyst.* **12**, 2531–2544.
34. Jones, C., (2021). Impact of imperfect data on the performance of algorithms to compare near-ultraviolet circular dichroism spectra. *Appl. Spectrosccopy* **75**, 857–866.
35. Jones, C., (2021). Wavelength calibration uncertainty in protein circular dichroism databank spectra. *Appl. Spectroscopy* **75**, 1207–1211.
36. Miles, A.J., Janes, R.W., Wallace, B.A., (2021). Tools and methods for circular dichroism spectroscopy of proteins: a tutorial review. *Chem. Soc. Rev* **50**, 8400–8413.
37. Wallace, B.A., (2020). The role of circular dichroism spectroscopy in the era of integrative structural biology. *Curr. Opin. Struct. Biol.* **58**, 191–196.
