# Supplementary material for: PDBx/mmCIF Ecosystem: Foundational Semantic Tools for Structural Biology
Source: J Mol Biol. Author manuscript; Available in PMC 2023 Jun 26. (PMC10292674; doi:10.1016/j.jmb.2022.167599)
Supplement: Article [file NIHMS1907597-supplement-Article.zip › CoMent--Relationships-Between-Biomedical-Concepts-Infe_2022_Journal-of-Molec.pdf]

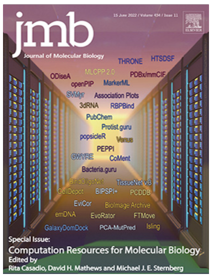

CoMent: Relationships Between Biomedical Concepts Inferred From the Scientific Literature

Florencio Pazos [1*], Mónica Chagoyen [1], Pedro Seoane [2,3,4] and Juan A. G. Ranea [2,3,4]

*1 - **Computational Systems Biology Group,** Systems Biology Department, National Center for Biotechnology (CNB-CSIC), c/ Darwin, 3, Madrid 28049, Spain*
*2 - **Department of Molecular Biology and Biochemistry,** University of Malaga, Malaga 29071, Spain*
*3 - **CIBER de Enfermedades Raras,** Instituto de Salud Carlos III, Madrid, Spain*
*4 - **Institute of Biomedical Research in Malaga (IBIMA),** Malaga, Spain*

***Correspondence to Florencio Pazos:** pazos@cnb.csic.es (F. Pazos), @fpazos_bioinf (F. Pazos)*
https://doi.org/10.1016/j.jmb.2022.167568
Edited by Rita Casadio

Abstract

The mining of the massive amounts of biomedical information is hindered by the still scarce representation of these data using formal vocabularies and ontologies, which is necessary for cross-linking conceptual entities between different resources and, in general, represent/ing the information in a computer-tractable way. Basic things such as retrieving a comprehensive list of associations between complex diseases and their reported symptoms or underlying biological processes, given in terms of formal identifiers, are not trivial and, in many cases, these have to be generated by manual curation or inferred/predicted from indirect evidences. In this work, using a text-mining approach based on detecting significant co-mentions in the scientific literature, we generated a resource with millions of relationships between thousands of terms representing diseases, symptoms, biological processes, molecular functions and cellular compartments, all given in terms of formal identifiers of these terms in the main resources dealing with them. We show some examples that highlight the differences between these relationships and those that are available in other resources. These relationships can be queried and inspected in an interactive web interface freely available at: https://sysbiol.cnb.csic.es/CoMent.
© 2022 The Author(s). Published by Elsevier Ltd. This is an open access article under the CC BY license (http://creativecommons.org/licenses/by/4.0/).

Introduction

As most areas of modern Biology, Biomedicine is characterized by the accumulation of massive amounts of data whose mining could provide valuable knowledge with eventual practical applications for disease diagnosis and treatment. Computational approaches that mine biomedical data require the usage of standardized vocabularies and formal identifiers in order to cross-link conceptual entities between different resources and, in general, to represent the information in a computer-tractable way. An example is to use standardized vocabularies to represent diseases, their underlying molecular processes and their symptoms, in such a way that the typical symptoms characterizing a disease and the molecular processes affected by it can be represented by links between these sets of formal entities. Resources containing these linkages between biomedical-related entities are still scarce, and many of the existing alternatives are non-free commercial systems or contain predicted/inferred links based on indirect evidences.

Clinical signs/symptoms are the basis for differential diagnosis in clinical practice. Due to the low prevalence of rare diseases, matching

0022-2836/© 2022 The Author(s). Published by Elsevier Ltd.This is an open access article under the CC BY license (http://creativecommons.org/licenses/by/4.0/).

patients with similar clinical manifestations at the national and international levels is essential for diagnosis and translational research. This fostered the development of the Human Phenotype Ontology (HPO),[1] a controlled vocabulary structured in an ontology that describes, among others, phenotypic abnormalities associated with disease conditions. The HPO has become the 'de-facto' standard vocabulary to describe the clinical manifestations of rare disease patients in resources like DECIPHER[2] or PhenomeCentral[3], and also for the annotation of Mendelian diseases compiled in genetic resources like OMIM[4] and ORPHANET[5] with their symptoms.

However currently public resources with comprehensive compilations of disease-sign relationships for all known diseases given in terms of controlled vocabularies are scarce. In the absence of curated resources, text-mining approaches have been previously used to extract common disease-signs connections. Zhou et al. mined PubMed to construct a disease network based on shared symptoms[6]. They used this network to study the clinical relationships of diseases and their underlying molecular interactions. An HPO pilot project to obtain common-disease annotations also used the literature.[7]

The main initiative that aims to represent links between different biomedical concepts, including disease-symptoms, is that carried out by Monarch.[8] This platform is an integrative data and analytic system that search for phenotype-genotype relationships across species, integrating basic and applied research using semantics-based analyses. To do this, it integrates data from resources such as Ensembl[9], "NCBI disease corpus"[10], ORPHANET, DECIPHER, STRING[11] or Reactome[12], via the ontologies used by them, such as EFO[13], HPO,[14] Gene Ontology (GO)[15] and DO[16], and others ontologies developed by Monarch itself, such as MONDO, to build a data graph called "Integrated Knowledge Graph"[8]. This combined graph allows data integration in a logical way and removes all the existing incoherencies between the different resources. Using the graph, the platform generates relations between concepts such as disease, phenotype, gene, variant, pathway, genotype, etc. and report them for human and animal models. With this approach, it is possible to generate relationship that connect OMIM, OMIA[17], ORPHA[5] and MONDO terms (disease terms) with terms related to functions and phenotypes (i.e. HPO, UBERON[18], MP[19] and some GO terms). However, although these relations could help in the diagnosis process, they cannot explain which mechanisms/functions could be affected.

Indeed, it is for the relationships between pathology-related concepts and molecular functions/biological processes where the situation is worse, in spite of the importance of these connections for linking diseases with their underlying molecular mechanisms. In the absence of curated resources, researchers have developed different strategies to predict or infer this kind of linkages, usually using genes as intermediates. For example, Davis et al.[20] inferred Gene Ontology – disease connections through the integration of GO-gene annotations with the gene-disease set from the Comparative Toxicogenomic Database (CTD).[21] Diseases are represented by MeSH or OMIM identifiers. Some relationships present in Monarch also point in this direction, such as the disease-pathway and gene-function linkages, but the first only relates OMIM and MONDO terms with Reactome pathways, no GO terms, and the latter is not a direct link between disease and functions.

Regarding relationships between biological processes/molecular functions/cellular compartments and phenotypes/clinical signs, the existing approaches also generate predictions based on shared genes. For example, HPO2GO[22] is based on co-annotations of the HPO and GO terms on the same genes/proteins.

In this work, using a text-mining based approach we generated a resource with relationships between thousands of diseases (as represented in the MONDO and EFO resources), symptoms/clinical signs (as represented in the HPO resource) and biological processes, molecular functions and subcellular compartments (as represented by these categories of GO). The approach looks for co-mentions of the textual descriptions of these items in the PubMed corpus of ∼34 M abstracts, applying a statistical test to evaluate the significance of the observed frequencies of co-mentions. The resource contains, represented in a structured way, millions of relationships between diseases and symptoms (EFO-HPO, EFO-MONDO), between diseases and possible underlying biological pathways/processes/molecular functions/compartments (EFO-GO, MONDO-GO), and between symptoms and pathways processes/molecular functions/compartments (HPO-GO). Some of these types of linkages, given in terms of the formal vocabularies of these widely-used databases, are not available in existing open resources. These literature-inferred relationships can be downloaded or queried through an interactive web interface where this information is presented together with additional data and links to the original resources.

Results

With the procedure described in Methods, we obtained 9,671,195 relationships between 58,298 terms of the four ontologies with a p-value $\leq$ 0.001. Table 1 shows the number of pairs between the different ontologies and the number of terms considered in each ontology.

Table 1 Number of terms considered in each ontology and fraction respect to the whole size of the ontology (diagonal). Number of co-mention relationships found for each pair of ontologies (p-val $\leq 1{\cdot}10^{-3}$).

	HPO	GO	EFO	MONDO
**HPO**	***15,648 (96%)***	1,524,475	466,289	3,610,377
**GO**		***18,538 (39%)***	665,693	3,404,361
**EFO**			***2,529 (8%)***	
**MONDO**				***21,583 (88%)***

Web interface

Figure 1 shows representative screenshots of the interactive web interface developed for querying these pairs of terms showing significant co-mentions in the literature. The system is freely available at https://sysbiol.cnb.csic.es/CoMent. The main interface has four areas to search in the four ontologies (HPO, GO, EFO and MONDO), identified with different colors. The user can search by the textual description of the terms, including synonyms, as well as by the corresponding IDs. A list with all terms matching the search criterion is shown (Figure 1(A)). The list contains the terms' IDs, descriptions and synonyms, and additional information can be obtained at the original resources following the links of the IDs. At this point, the user can restrict the search to reduce the list, select the ID he/she is interested in to limit the search to it, or leave the whole list and retrieve relationships for all these terms in the forthcoming steps. This last option is useful in cases where there are similar terms in the ontology and we are interested in all of them. For example, there are 15 biological processes representing different aspects of "lipid metabolism" in GO. If we want to retrieve all diseases (EFO and/or MONDO) related to lipid metabolism in general, we might want to use all these 15.

Once the search area contains the term or terms of interest, the user has to select in which other ontologies he/she wants to retrieve co-mention relationships by selecting the lines connecting them (Figure 1). There are five possible pairs of linkages: relationships between clinical signs/symptoms and biological processes/cellular compartments/molecular functions (HPO-GO); between complex (GWAS-related) pathologies and clinical signs (EFO-HPO), between other diseases, including mendelian, and clinical signs (MONDO-HPO); and between diseases and biological processes/cellular compartments/molecular functions (EFO-GO and MONDO-GO).

When the term(s) of interest are displayed and the type(s) of relationship(s) selected, pressing "Search relationships" will generate the list of co-mention relationships (Figure 1(B)). For each item in the list (pair of terms), the IDs and names of both terms are shown, being the firsts links to the entries for these terms in the corresponding resources. There are two links for performing text searches of both names together in Google ("G") and in PubMed ("P") to further investigate on the relationship. These searches do not include synonyms. The "string similarity" between both names (including their synonyms, not shown in this list) is also indicated and cases of high similarity ($\geq$0.7) are highlighted in red color. The next columns show the number of PubMed entries mentioning the first term, those mentioning the second, those mentioning both together (which is a link to this list of co-mentioning articles), and the ratio of these respect to the minimum of the first two (see Methods). The final column shows the p-value of the hypergeometric test for assessing the significance of the co-mention (see Methods). Only pairs with p-value $\leq$ 0.001 are shown, and those with p-value $\leq 1{\cdot}10^{-5}$ are highlighted in green color. The table can be sorted by any column by clicking the corresponding header, and by default it is sorted by the ratio of papers mentioning both terms respect to the minimum of the papers mentioning each term individually. There is a link to download the whole table in . TSV format so that it can be imported into an external spreadsheet program.

Comparison with other resources

We performed some systematic comparisons of the set of relationships we obtained with those in other resources for the relationships between pathological terms and GO functions, that are the most novel generated in this work. Although benchmarking performances is unfeasible due to the lack of a "gold standard" set for these kinds of relationships, we compare with other resources in terms of coverage and characteristics of the relationships. In the next section, we show some examples for which the performance was manually assessed and compared with other resources.

HPO2GO[22] infer HPO-GO links indirectly through shared genes. There are 17,238 HPO-GO pairs in common between ours (Table 1) and the 238,884 listed in that resource. To get more insight into that difference we checked, for the 25% top-scoring pairs in both resources, the specificity of the terms involved (i.e. level in the corresponding hierarchies). Supplementary Figure 1 clearly shows that our pairs point to more specific processes, both in GO and HPO hierarchies, while HPO2GO top pairs

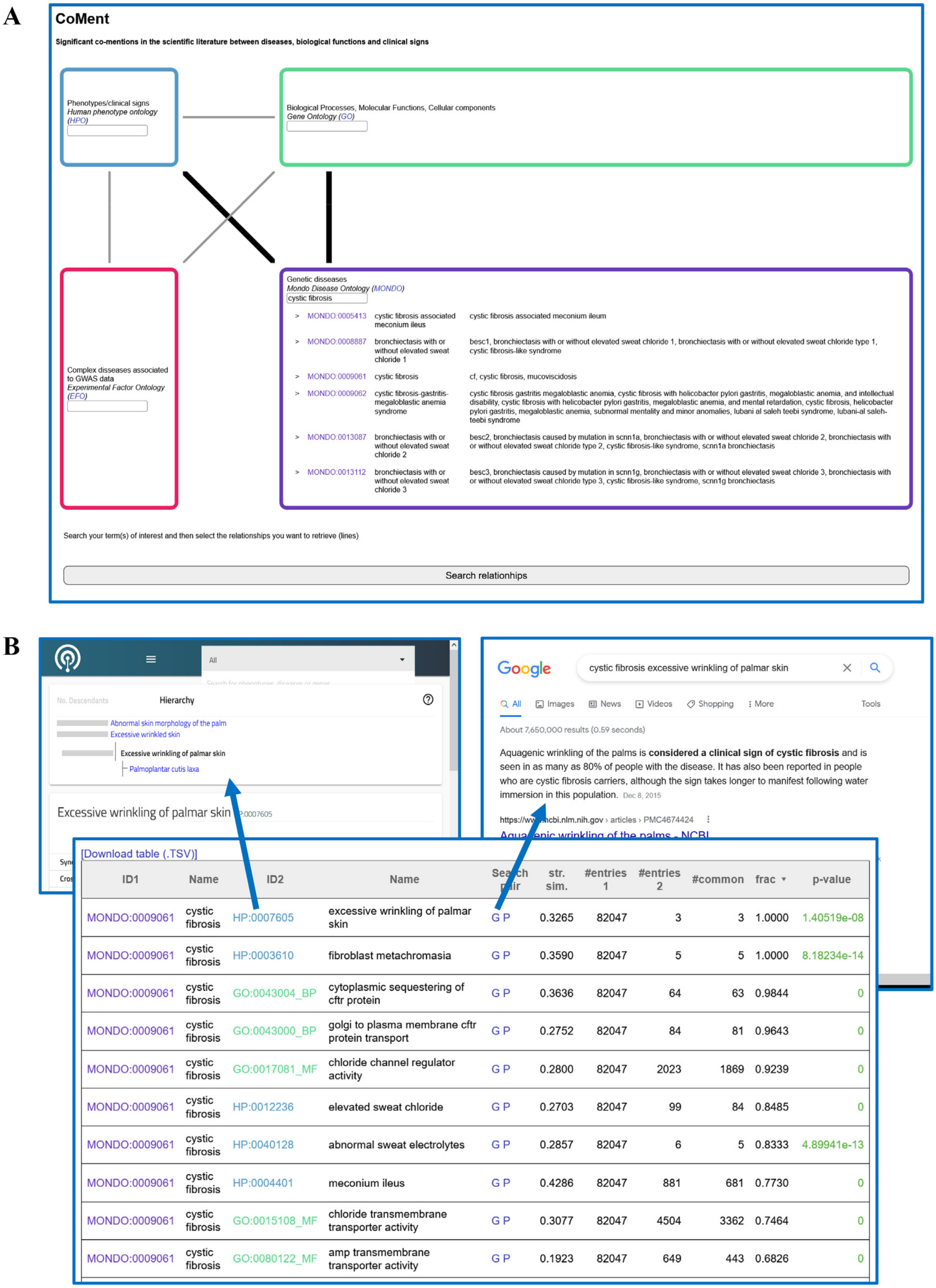

ID1	Name	ID2	Name	Search pair	str. sim.	#entries 1	#entries 2	#common	frac	p-value
MONDO:0009061	cystic fibrosis	HP:0007605	excessive wrinkling of palmar skin	G P	0.3265	82047	3	3	1.0000	1.40519e-08
MONDO:0009061	cystic fibrosis	HP:0003610	fibroblast metachromasia	G P	0.3590	82047	5	5	1.0000	8.18234e-14
MONDO:0009061	cystic fibrosis	GO:0043004_BP	cytoplasmic sequestering of cftr protein	G P	0.3636	82047	64	63	0.9844	0
MONDO:0009061	cystic fibrosis	GO:0043000_BP	golgi to plasma membrane cftr protein transport	G P	0.2752	82047	84	81	0.9643	0
MONDO:0009061	cystic fibrosis	GO:0017081_MF	chloride channel regulator activity	G P	0.2800	82047	2023	1869	0.9239	0
MONDO:0009061	cystic fibrosis	HP:0012236	elevated sweat chloride	G P	0.2703	82047	99	84	0.8485	0
MONDO:0009061	cystic fibrosis	HP:0040128	abnormal sweat electrolytes	G P	0.2857	82047	6	5	0.8333	4.89941e-13
MONDO:0009061	cystic fibrosis	HP:0004401	meconium ileus	G P	0.4286	82047	881	681	0.7730	0
MONDO:0009061	cystic fibrosis	GO:0015108_MF	chloride transmembrane transporter activity	G P	0.3077	82047	4504	3362	0.7464	0
MONDO:0009061	cystic fibrosis	GO:0080122_MF	amp transmembrane transporter activity	G P	0.1923	82047	649	443	0.6826	0

Figure 1. Screenshots of the CoMent web interface. (A) Initial screen with the four areas to search terms in the four ontologies (colors). A search for "cystic fibrosis" has been performed in MONDO, resulting in five terms, and it was chosen to retrieve relationships between MONDO terms and those in GO and HPO. (B) Top of the list of relationships between MONDO:0009061 ("cystic fibrosis") and HPO and GO terms.

include many general processes close to the root of the hierarchies.

CTD[21] contains disease-GO relationships inferred also from shared genes and chemical compounds, not associated to any score in this case. While diseases are given in terms of MeSH IDs, these can be converted to MONDO IDs as this last resource provides MeSH-MONDO equivalences. From the 2,096,159 CTD relationships that can be translated into MONDO-GO pairs, 175,037 are shared with our own list of pairs (Table 1). In this case there is no difference in the specificity of the terms involved in these pairs (Supplementary Figure 2). To get more insight into this difference in the set of pairs, we evaluated the intersection between both resources regarding the terms involved in these pairs (Supplementary Table 1). The results show that these pairs present almost the same set of GO terms in both resources but they largely differ on the set of MONDO terms, with CTD covering less than 1/3 of the MONDO terms involved in CoMent pairs.

Examples

As an example, we retrieved from the web interface all symptoms (HPO) and cellular processes/molecular functions/biological processes (GO) related to Alzheimer's disease (EFO:0000249).

All the terms that show up in the list make sense considering what we intuitively known about this complex pathology and, together, they provide a comprehensive picture of its internal causes and external symptoms. Regarding clinical signs (HPO), we see the typical molecular/physiological manifestations related to amyloid levels in cerebrospinal fluid, senile plaques, neurofibrillary tangles, cortical atrophy, hirano bodies, etc, as well as higher level mental and behavioral symptoms (apathy, delusions, dementia, memory problems, ...). Within the biological processes (GO_BP) terms, we find those related to amyloid-beta metabolism, neurofibrillary tangles. Regarding molecular functions (GO_MF), we can see those related to binding to amyloid-beta and X11-like proteins and peptidase activities associated to amyloid precursor cleavage, among others. Also the "cellular compartments" category of GO (GO_CC) reflects what is known on this disease, with terms such as gamma-secretase complex, alpha-ketoglutarate dehydrogenase complex, lewy bodies, etc.

This example also illustrates some drawbacks of the approach. An obvious one is the inability to distinguish "positive" from "negative" relationships. For example, this disease is linked in our data to both, "elevated SCF amyloid levels", as well as "decreased SCF amyloid levels". The first one is obvious but the second is due to many articles describing strategies aimed at decreasing amyloid levels in Alzheimer. In the list, we can also see the trivial relationship with the "symptom" "Alzheimer disease", as this is also a HPO term (HP:0002511), in spite of the filters we used trying to avoid redundancy between the four ontologies (see Methods). Nevertheless, these cases could be discarded automatically based on the string similarity parameter.

Table 2 shows a comparison of the symptoms annotated in the Mayo Clinic for this disease, which can be extracted manually from the textual description of this pathology (https://www.mayoclinic.org/diseases-conditions/alzheimers-disease/symptoms-causes/syc-20350447), and those obtained with our resource. Some of the symptoms listed in the Mayo Clinic do not have an equivalent in HPO and hence they could not be retrieved with this approach (e.g. "distrust in others"). For those with equivalences in the HPO vocabulary, all are picked up by our system except "difficulty planning and performing tasks", whose closest equivalent term in HPO ("impaired ability to plan", HP:0033055) was not detected. On the contrary, many symptoms detected by our system are not listed in the Mayo Clinic entry (Table 2). Those are mainly related to molecular a physiological manifestations of the disease, which are not the main focus of the Mayo Clinic resource, more oriented towards informing patients. Apart from generating a more comprehensive annotation of symptoms, a crucial feature of the system presented here is that those are annotated using a controlled vocabulary (HPO terms, in this case).

As another example, we retrieved from the system the symptoms (HPO), biological process, molecular functions and cellular compartments (GO) related to a prototypical mendelian/monogenic disease: cystic fibrosis (MONDO:0009061). The reported symptoms and biological processes/molecular functions are those intuitively associated to this disease: processes/molecular functions related to the CFTR protein, symptoms related to ion levels in sweat, palmar skin wrinkling, "meconius ileus", etc. The top of the list can be seen in Figure 1(B). All the symptoms listed in the textual description of this disease in the Mayo Clinic are picked up by the system (data not shown) and, as with the Alzheimer's disease example, many meaningful molecular and physiological symptoms are additionally recovered.

For mendelian/monogenic diseases it is possible to obtain lists of symptoms expressed in the HPO vocabulary from the HPO resource itself. So, for this example it is possible to compare the HPO terms retrieved by our system (not the GO terms) with those available at HPO. Table 3 lists all the symptoms annotated in HPO for "cystic fibrosis" (which were compiled from OMIN and ORPHANET), compared with the top of the list provided by our resource (score $\geq$ 0.4 and p-value

Table 2 Comparison of the Alzheimer's disease symptoms listed in the textual description of this disease in the Mayo Clinic with those retrieved by CoMent (down to score 0.4).

Mayo Clinic	CoMent
memory loss	HP:0002354 memory impairment<br>HP:0033688 long term memory impairment<br>HP:0033692 declarative memory loss
difficulty for thinking, reasoning and concentrating	*(no equivalent terms in HPO)*
difficulty making judgments and decisions	*(no equivalent terms in HPO)*
difficulty planning and performing tasks	
depression	HP:0000716 depressivity
apathy	HP:0000741 apathy
Social withdrawal	HP:0012433 abnormal social behavior<br>HP:0000735 impaired social interactions
mood swings	HP:0000720 mood swings<br>HP:0001575 mood changes
distrust in others	*(no equivalent terms in HPO)*
irritability and aggressiveness	HP:0000737 irritability<br>HP:0000718 aggressive behavior
changes in sleeping habits	HP:0002360 sleep disturbance
wandering	*(no equivalent terms in HPO)*
loss of inhibitions	HP:0000734 disinhibition
delusions	HP:0000746 delusions
	HP:0030860 abnormal csf amyloid level
	HP:0030861 decreased csf amyloid level
	HP:0500211 abnormal csf threonine concentration
	HP:0100256 senile plaques
	HP:0002185 neurofibrillary tangles
	HP:0002528 granulovacuolar degeneration
	HP:0003791 deposits immunoreactive to beta-amyloid protein
	HP:0030862 elevated csf amyloid level
	HP:0007112 temporal cortical atrophy
	HP:0500213 decreased csf threonine concentration
	HP:0007453 flexural lichenification
	HP:0100316 hirano bodies
	HP:0011970 cerebral amyloid angiopathy
	HP:0012662 parietal hypometabolism in fdg pet
	HP:0500212 increased csf threonine concentration
	HP:0033252 palmar hyperlinearity
	HP:0011232 infra-orbital fold
	HP:0000727 frontal lobe dementia

$\leq 1{\cdot}10^{-5}$). From the 35 symptoms annotated in HPO, only four are absent in our list: "hepatosplenomegaly", "hypercalciuria", "immunodeficiency" and "reduced forced vital capacity". On the other hand, many symptoms, whose association to the disease can be corroborated in the literature, are retrieved by our system but not included in HPO (Table 3).

Table 3 Comparison of the cystic fibrosis symptoms listed HPO with those retrieved by CoMent (down to score 0.4).

HPO (OMIN & ORPHANET)		CoMent	
HP:0001394	Cirrhosis	HP:0001394	
HP:0001433	Hepatosplenomegaly		
HP:0001508	Failure to thrive	HP:0001508	
HP:0001648	Cor pulmonale	HP:0001648	
HP:0001733	Pancreatitis	HP:0001733	
HP:0001738	Exocrine pancreatic insufficiency	HP:0001738	
HP:0001738	Exocrine pancreatic insufficiency	HP:0001738	
HP:0001944	Dehydration	HP:0001944	
HP:0002014	Diarrhea	HP:0002014	
HP:0002024	Malabsorption	HP:0002024	
HP:0002035	Rectal prolapse	HP:0002035	
HP:0002099	Asthma	HP:0002099	
HP:0002105	Hemoptysis	HP:0002105	
HP:0002110	Bronchiectasis	HP:0002110	
HP:0002150	Hypercalciuria		
HP:0002205	Recurrent respiratory infections	HP:0002205	
HP:0002206	Pulmonary fibrosis	HP:0002206	
HP:0002240	Hepatomegaly	HP:0002240	
HP:0002570	Steatorrhea	HP:0002570	
HP:0002595	Ileus	HP:0002595	
HP:0002613	Biliary cirrhosis	HP:0002613	
HP:0002721	Immunodeficiency		
HP:0003251	Male infertility	HP:0003251	
HP:0004313	Decreased circulating antibody level	HP:0004313	
HP:0004401	Meconium ileus	HP:0004401	
HP:0006528	Chronic lung disease	HP:0006528	
HP:0006532	Recurrent pneumonia	HP:0006532	
HP:0006538	Recurrent bronchopulmonary infections	HP:0006538	
HP:0011109	Chronic sinusitis	HP:0011109	
HP:0012236	Elevated sweat chloride	HP:0012236	
HP:0032341	Reduced forced vital capacity		
HP:0032342	Reduced forced expiratory volume in one second	HP:0032342	
HP:0032359	Decreased forced expiratory flow 25–75%	HP:0032359	
HP:0100582	Nasal polyposis	HP:0100582	
HP:0100759	Clubbing of fingers	HP:0100759	
		HP:0007605	excessive wrinkling of palmar skin
		HP:0003610	fibroblast metachromasia
		HP:0040128	abnormal sweat electrolytes
		HP:0005700	increased bone density with cystic changes
		HP:0008327	microscopic nephrocalcinosis
		HP:0032493	increased circulating trypsinogen
		HP:0003653	cellular metachromasia

Discussion

We have developed a system that can automatically retrieve significant co-mentions of biomedical concepts in the scientific literature and report them using controlled vocabularies. We have applied it for detecting relationships between GO molecular functions/biological processes/cellular compartments, HPO clinical signs, and EFO and MONDO diseases, and the results can be queried online through an interactive web server.

Looking for co-mentions in the scientific literature is a widely used strategy for the automatic extraction of information and its representation in a structured way (see for example.[6,23–24] The system described here is not going to discover new or unknown relationships. Its utility resides in the fact that it is able to comprehensively scan the whole scientific literature for (reported) relationships and, more importantly, to deliver them using controlled vocabularies and standardized identifiers. This is fundamental for carrying out large scale studies, for matching information between different resources and, in general, for representing the current biomedical knowledge in ways tractable by computers. The traditional way for representing biological knowledge using standardized vocabularies and IDs is by manual annotation. The system presented here can help manual annotators providing them an initial set of relationships to check, although in the examples we have seen, the quality of the results, as far as we can evaluate, is enough for using these relationships as they are.

Although some of the types of relationships generated here are available in other resources (leaving apart differences of coverage and methodology), others are novel. Of special importance are those relating GO terms (molecular functions, biological processes and cellular compartments) with pathology-related terms (diseases and symptoms). In spite of these relationships being crucial for performing systematic studies on the molecular basis of pathologies, they are scarce and usually derived from indirect evidences, such as shared genes. In comparison with HPO-GO pairs inferred from shared genes, our pairs point to more specific symptoms and biological functions. Our disease-GO pairs include many more diseases as our approach is not restricted to diseases with known associated genes. In any case, the fact that the sets of pairs reported by both approaches are quite different highlights their complementarity. While Monarch provides some links between symptoms/diseases and biological processes, these are restricted to the GO terms that can be interpreted as phenotypes and to those that are linked to Reactome pathways, and do not include "cellular compartment" terms (CC), for example. Even if some of the types of linkages we are reporting have been already generated using different approaches, for large scale studies which uses many of them concomitantly it is desirable to have all based on the same methodology and/or dataset, instead of, for example, mixing HPO-GO relationships based on gene sharing with HPO-disease relationships based on manual curation/annotation.

The two examples presented illustrate some important features of the system. First, it usually recovers relationships in a very comprehensive way, hence including many relationships that, without being necessary wrong, could be trivial or even anecdotal. In the case of the disease-symptoms relationships, these could include true relationships but with very low prevalence, for example, that could have been intentionally discarded in other resources for that reason. This seems to be the case for some of the cystic fibrosis symptoms picked up by our system and not included in HPO, but not for all: for example, "excessive wrinkling of palmary skin" is present in 80% of people with the disease[25] in spite of not being listed in HPO for cystic fibrosis. The comparison with Mayo Clinic's textual description of Alzheimer's disease symptoms illustrates how our system can recover many more symptoms, including those at the molecular level which are absent in many of these clinical repositories, and how these are automatically given in terms of controlled vocabularies, which allow further automatic processing.

An important drawback of this approach is that, right now, it cannot distinguish "positive" from "negative" relationships, as illustrated in one of the examples shown. Another example of a negative relationship we have found is that between Huntington's disease and "obesity": these two concepts are significantly mentioned together in the literature but to state that Huntington's disease "reduces" obesity. Nevertheless, in our experience so far, negative relationships are very rare and, moreover, in the web interface they can be easily grasped following the links to Google and PubMed.

Although here we applied the method to five types of relationships of biomedical interest, the approach is extensible to others, and for the future we plan to include other categories that also have associated controlled vocabularies, such as microorganisms and chemical compounds. In this way, this approach can be used to retrieve a large network of literature-based linkages between biomedical concepts of many categories, given in terms of standardized vocabularies.

Materials and Methods

The methodology for detecting significant co-mentions between terms of biomedical interest in the scientific literature is schematized in Figure 2.

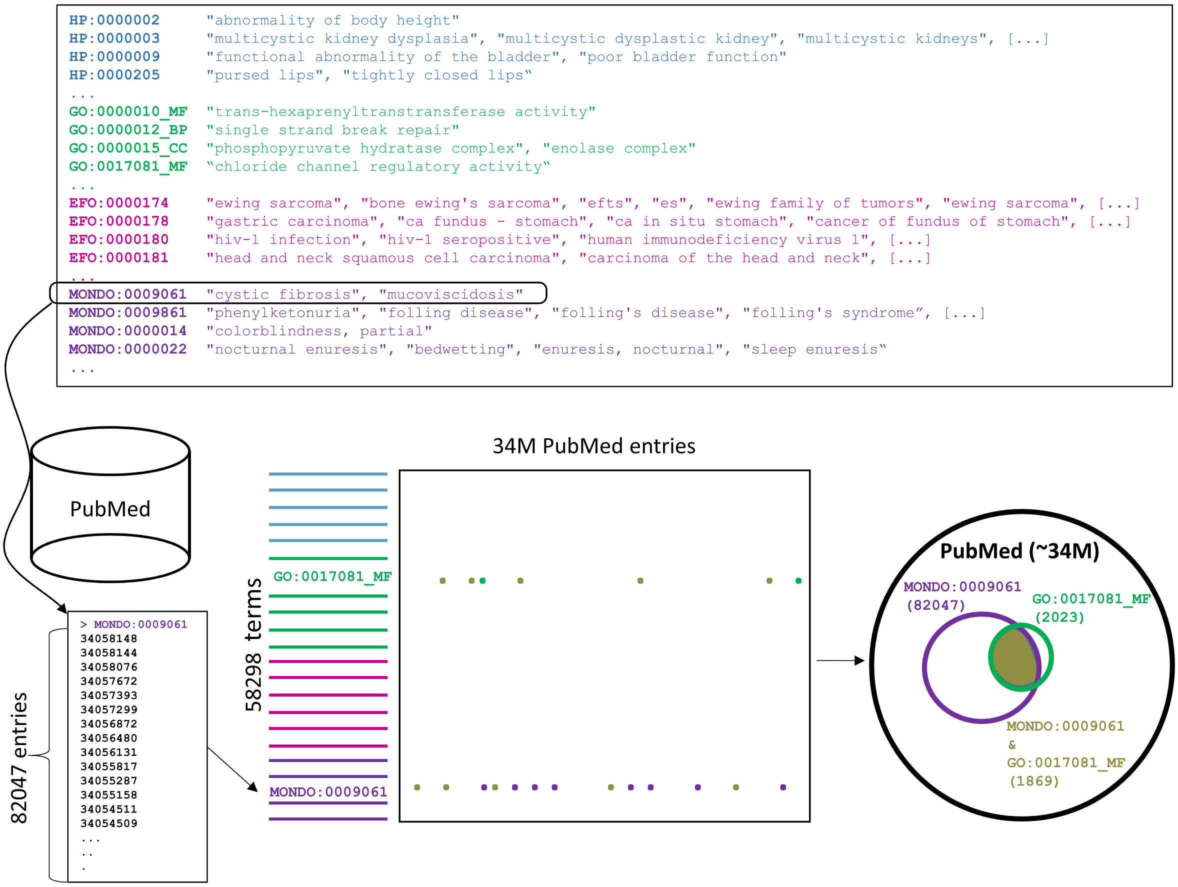

Figure 2. Schema of the methodology. An initial list of terms from the four ontologies (colors), including their names and synonyms, is generated (top). For each term, a PubMed search is performed aimed at retrieving the IDs of the entries mentioning the term or any of its synonyms. The result of these searches for all terms can be visualized as a matrix representing which articles mention each term. From two rows of this matrix, the frequencies of articles mentioning each term as well as that of entries mentioning the two together can be obtained. From these figures, a hypergeometric test is applied to assess the significance of the eventual co-mention (right).

Data retrieval

Sets of terms of biomedical interest are extracted from different ontologies, including their textual descriptions and synonyms. For the "Human Phenotype Ontology" (HPO)[1], we took all the terms that have "phenotypic abnormality" (HP:0000118) as an ancestor, in order to avoid terms not related to phenotypes or clinical signs. For "Gene Ontology" (GO),[15] we took all the terms from the three categories ("biological process" (BP), "molecular function" (MF) and "cellular compartment" (CC)) that are associated to at least one human gene in the "Gene Ontology Annotation" database (GOA)[26], in an attempt to filter out terms not related to human biology. For the "Mondo Disease Ontology"[8], we took all terms not cross-referenced to an HPO term in order to avoid MONDO terms describing symptoms, which are already represented in HPO. The final list of MONDO terms comprises mainly human pathologies although it includes other biomedical concepts as well. Finally, from the "Experimental Factor Ontology" (EFO)[13] we retrieve all the terms linked to a trait in the Catalog of Genome-Wide Association Studies (GWAS)[27] in an attempt to restrict to terms related to human diseases associated to genomic variations. Nevertheless, other biomedical concepts apart from diseases are included here as the GWAS catalog contains traits that are not diseases, as long as they are associated to genomic variations. Our intention was that EFO and MONDO jointly cover the current landscape of human pathologies, although some redundancy between these two sets exists. Our final list contains 58,298 terms of biomedical interest from the four ontologies (Figure 2), and its composition is detailed in Table 1.

For each term, we retrieved the list of associated PubMed identifiers (PMIDs) by querying NCBI's Entrez API.[28] For that, we searched for the textual description of the term (or its synonym(s) listed in the corresponding ontology) in any field of PubMed. For assembling the query string, a term's name and

its synonyms are combined with "OR" operators, and multi-word terms/synonyms are quoted. We tried to replicate how a user would search for a concept of interest in PubMed web interface. As performing searches of two terms together (combined with "AND") would be unfeasible for all pairs ($\sim 1 \cdot 10^9$ pairs), we took the intersection between each term's list of PMIDs as the set of articles mentioning the two together (Figure 2). All data were downloaded on June 2021.

Co-mention evaluation

At this point, for each pair of terms we have the number of papers mentioning each of them (n_1 and n_2), as well as the number of papers mentioning both together (b), within the universe of ∼34 M PubMed articles (P). With these figures we calculate the fraction of papers mentioning the term of lowest frequency that mention both:

$$s = b/MIN(n_1, n_2)$$

The p-value of the null-hypothesis that the co-mention of both terms occurs by chance due to their frequencies is calculated with the hypergeometric distribution:

$$pval = 1 - \sum_{i=0}^{b} \frac{\binom{n_1}{i}\binom{P - n_1}{n_2 - i}}{\binom{P}{n_2}}$$

Additionally, for each pair of terms we also calculate the string similarity between their textual descriptions, including all possible pairs of synonyms, and take the highest one as a measure of "textual similarity" between both terms. For that we used the algorithm implemented Perl's "String::Similarity" library, which basically measures the minimum number of edit operations required for converting one string into the other.[29] This was done as terms with identical or very similar descriptions (such as the same concept represented in different ontologies) lead to trivial co-mentions that should be eventually discarded.

Web interface

A web interface was developed where the user can search for his/her term(s) of interest in any of the four ontologies and retrieve the associated terms in the others. The system if freely available and includes a guided tutorial.

CRediT authorship contribution statement

Florencio Pazos: Conceptualization, Data curation, Formal analysis, Investigation, Methodology, Software, Writing – original draft, Writing – review & editing. **Mónica Chagoyen:** Conceptualization, Data curation, Formal analysis, Investigation, Methodology, Software, Writing – original draft, Writing – review & editing. **Pedro Seoane:** Conceptualization, Data curation, Formal analysis, Investigation, Methodology, Software, Writing – original draft, Writing – review & editing. **Juan A.G. Ranea:** Conceptualization, Data curation, Formal analysis, Investigation, Methodology, Software, Writing – original draft, Writing – review & editing.

DECLARATION OF COMPETING INTEREST

The authors declare that they have no known competing financial interests or personal relationships that could have appeared to influence the work reported in this paper.

Acknowledgements

The Spanish Ministry of Economy and Competitiveness with European Regional Development Fund [PID2019-108096RB-C21 and PID2019-108096RB-C22]; the Andalusian Government with European Regional Development Fund [UMA18-FEDERJA-102 and PAIDI 2020:PY20-00372]; The European Food Safety Authority [GP/EFSA/ENCO/2020/02]; and the Ramón Areces foundation for rare disease investigation (National call for research on life and material sciences, XIX edition). The conclusions, findings and opinions expressed in this scientific paper reflect only the view of the authors and not the official position of the European Food Safety Authority.

Appendix A. Supplementary data

Supplementary data to this article can be found online at https://doi.org/10.1016/j.jmb.2022.167568.

Received 29 November 2021;
Accepted 22 March 2022;
Available online 30 March 2022

Keywords:
biomedical ontology;
disease;
symptom;
biological process;
molecular function

References

1. Robinson, P.N., Köhler, S., Bauer, S., Seelow, D., Horn, D., Mundlos, S., (2008). The Human Phenotype Ontology: a tool for annotating and analyzing human hereditary disease. *Am. J. Hum. Genet.* **83**, 610–615. https://doi.org/10.1016/j.ajhg.2008.09.017.
2. Bragin, E., Chatzimichali, E.A., Wright, C.F., Hurles, M.E., Firth, H.V., Bevan, A.P., Swaminathan, G.J., (2014).

DECIPHER: database for the interpretation of phenotype-linked plausibly pathogenic sequence and copy-number variation. *Nucleic Acids Res.* **42**, D993–D1000. https://doi.org/10.1093/nar/gkt937.

3. Buske, O.J., Girdea, M., Dumitriu, S., Gallinger, B., Hartley, T., Trang, H., Misyura, A., Friedman, T., et al., (2015). PhenomeCentral: A Portal for Phenotypic and Genotypic Matchmaking of Patients with Rare Genetic Diseases. *Hum. Mutat.* **36**, 931–940. https://doi.org/10.1002/humu.22851.
4. Amberger, J.S., Bocchini, C.A., Scott, A.F., Hamosh, A., (2019). OMIM.org: leveraging knowledge across phenotype–gene relationships. *Nucleic Acids Res.* **47**, D1038–D1043. https://doi.org/10.1093/nar/gky1151.
5. Pavan, S., Rommel, K., Mateo Marquina, M.E., Höhn, S., Lanneau, V., Rath, A., (2017). Clinical Practice Guidelines for Rare Diseases: The Orphanet Database. *PLoS ONE* **12**, e0170365. https://doi.org/10.1371/journal.pone.0170365.
6. Zhou, X., Menche, J., Barabasi, A.L., Sharma, A., (2014). Human symptoms-disease network. *Nature Commun.* **5**, 4212. https://doi.org/10.1038/ncomms5212.
7. Groza, T., Köhler, S., Moldenhauer, D., Vasilevsky, N., Baynam, G., Zemojtel, T., Schriml, L.M., Kibbe, W.A., et al., (2015). The Human Phenotype Ontology: Semantic Unification of Common and Rare Disease. *Am. J. Hum. Genet.* **97**, 111–124. https://doi.org/10.1016/j.ajhg.2015.05.020.
8. Shefchek, K.A., Harris, N.L., Gargano, M., Matentzoglu, N., Unni, D., Brush, M., Keith, D., Conlin, T., et al., (2020). The Monarch Initiative in 2019: An integrative data and analytic platform connecting phenotypes to genotypes across species. *Nucleic Acids Res.*. https://doi.org/10.1093/nar/gkz997.
9. Yates, A.D., Achuthan, P., Akanni, W., Allen, J., Allen, J., Alvarez-Jarreta, J., Amode, M.R., Armean, I.M., et al., (2020). Ensembl 2020. *Nucleic Acids Res.*. https://doi.org/10.1093/nar/gkz966.
10. Doğan, R.I., Leaman, R., Lu, Z., (2014). NCBI disease corpus: A resource for disease name recognition and concept normalization. *J. Biomed. Inform.*. https://doi.org/10.1016/j.jbi.2013.12.006.
11. Szklarczyk, D., Gable, A.L., Lyon, D., Junge, A., Wyder, S., Huerta-Cepas, J., Simonovic, M., Doncheva, N.T., et al., (2019). STRING v11: Protein-protein association networks with increased coverage, supporting functional discovery in genome-wide experimental datasets. *Nucleic Acids Res.*. https://doi.org/10.1093/nar/gky1131.
12. Jassal, B., Matthews, L., Viteri, G., Gong, C., Lorente, P., Fabregat, A., Sidiropoulos, K., Cook, J., et al., (2020). The reactome pathway knowledgebase. *Nucleic Acids Res.*. https://doi.org/10.1093/nar/gkz1031.
13. Malone, J., Holloway, E., Adamusiak, T., Kapushesky, M., Zheng, J., Kolesnikov, N., Zhukova, A., Brazma, A., et al., (2010). Modeling sample variables with an Experimental Factor Ontology. *Bioinformatics*. https://doi.org/10.1093/bioinformatics/btq099.
14. Kohler, S., Carmody, L., Vasilevsky, N., Jacobsen, J.O.B., Danis, D., Gourdine, J.P., Gargano, M., Harris, N.L., et al., (2019). Expansion of the Human Phenotype Ontology (HPO) knowledge base and resources. *Nucleic Acids Res.* **47**, D1018–D1027. https://doi.org/10.1093/nar/gky1105.
15. Carbon, S., Douglass, E., Good, B.M., Unni, D.R., Harris, N.L., Mungall, C.J., Basu, S., Chisholm, R.L., et al., (2021). The Gene Ontology resource: Enriching a GOld mine. *Nucleic Acids Res.*. https://doi.org/10.1093/nar/gkaa1113.
16. Schriml, L.M., Arze, C., Nadendla, S., Chang, Y.W.W., Mazaitis, M., Felix, V., Feng, G., Kibbe, W.A., (2012). Disease ontology: A backbone for disease semantic integration. *Nucleic Acids Res.*. https://doi.org/10.1093/nar/gkr972.
17. Lenffer, J., Nicholas, F.W., Castle, K., Rao, A., Gregory, S., Poidinger, M., Mailman, M.D., Ranganathan, S., (2006). OMIA (Online Mendelian Inheritance in Animals): an enhanced platform and integration into the Entrez search interface at NCBI. *Nucleic Acids Res.*. https://doi.org/10.1093/nar/gkj152.
18. Mungall, C.J., Torniai, C., Gkoutos, G.V., Lewis, S.E., Haendel, M.A., (2012). Uberon, an integrative multi-species anatomy ontology. *Genome Biol.*. https://doi.org/10.1186/gb-2012-13-1-r5.
19. Smith, C.L., Eppig, J.T., (2015). Expanding the mammalian phenotype ontology to support automated exchange of high throughput mouse phenotyping data generated by large-scale mouse knockout screens. *J. Biomed. Semant.*. https://doi.org/10.1186/s13326-015-0009-1.
20. Davis, A.P., Wiegers, T.C., King, B.L., Wiegers, J., Grondin, C.J., Sciaky, D., Johnson, R.J., Mattingly, C.J., (2016). Generating Gene Ontology-Disease Inferences to Explore Mechanisms of Human Disease at the Comparative Toxicogenomics Database. *PLoS ONE* **11**, e0155530. https://doi.org/10.1371/journal.pone.0155530.
21. Davis, A.P., Grondin, C.J., Johnson, R.J., Sciaky, D., Wiegers, J., Wiegers, T.C., Mattingly, C.J., (2021). Comparative Toxicogenomics Database (CTD): update 2021. *Nucleic Acids Res.* **49**, D1138–D1143. https://doi.org/10.1093/nar/gkaa891.
22. Doğan, T., (2018). HPO2GO: prediction of human phenotype ontology term associations for proteins using cross ontology annotation co-occurrences. *PeerJ* **6**, e5298. https://doi.org/10.7717/peerj.5298.
23. Blaschke, C., Andrade, M.A., Ouzounis, C., Valencia, A., (1999). Automatic extraction of biological information from scientific text: Protein-Protein Interactions. *ISMB.*, 60–67.
24. Sartor, M.A., Ade, A., Wright, Z., States, D., Omenn, G.S., Athey, B., Karnovsky, A., (2012). Metab2MeSH: annotating compounds with medical subject headings. *Bioinformatics* **28**, 1408–1410. https://doi.org/10.1093/bioinformatics/bts156.
25. Katz, M., Ramot, Y., (2015). Aquagenic wrinkling of the palms. *CMAJ* **187**, E515. https://doi.org/10.1503/cmaj.150074.
26. Camon, E., Magrane, M., Barrell, D., Lee, V., Dimmer, E., Maslen, J., Binns, D., Harte, N., et al., (2004). The Gene Ontology Annotation (GOA) Database: sharing knowledge in Uniprot with Gene Ontology. *Nucleic Acids Res.* **32**, D262–D266.
27. MacArthur, J., Bowler, E., Cerezo, M., Gil, L., Hall, P., Hastings, E., Junkins, H., McMahon, A., et al., (2017). The new NHGRI-EBI Catalog of published genome-wide association studies (GWAS Catalog). *Nucleic Acids Res.* **45**, D896–D901. https://doi.org/10.1093/nar/gkw1133.
28. Entrez Programming Utilities Help, National Center for Biotechnology Information (US), 2010.
29. Myers, E., (1986). An O(ND) Difference Algorithm and its Variations. *Algorithmica* **1**, 251–266.
