# Supplementary material for: PDBx/mmCIF Ecosystem: Foundational Semantic Tools for Structural Biology
Source: J Mol Biol. Author manuscript; Available in PMC 2023 Jun 26. (PMC10292674; doi:10.1016/j.jmb.2022.167599)
Supplement: Article [file NIHMS1907597-supplement-Article.zip › BIPSPI---Mining-Type-Specific-Datasets-of-Protein-Compl_2022_Journal-of-Mole.pdf]

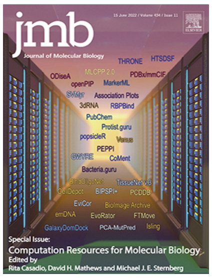

BIPSPI+: Mining Type-Specific Datasets of Protein Complexes to Improve Protein Binding Site Prediction

R. Sanchez-Garcia [1,2*], J. R. Macias [1], C. O. S. Sorzano [1], J. M. Carazo [1*] and J. Segura [3]

*1 - **Biocomputing Unit,** National Center for Biotechnology (CSIC), Darwin 3, Campus Univ. Autónoma de Madrid, Cantoblanco, 28049 Madrid, Spain*

*2 - **Oxford Protein Informatics Group,** Department of Statistics, University of Oxford, 29 St Giles' Oxford OX1 3LB, UK*

*3 - **Research Collaboratory for Structural Bioinformatics,** Protein Data Bank, San Diego Supercomputer Center, University of California San Diego, La Jolla, CA 92093, USA*

Correspondence to R. Sanchez-Garcia and J.M. Carazo: Oxford Protein Informatics Group, Department of Statistics, University of Oxford, Oxford OX1 3LB, UK, Biocomputing Unit, National Center for Biotechnology (CSIC), Darwin 3, Campus Univ. Autónoma de Madrid, Cantoblanco, 28049 Madrid, Spain. ruben.sanchez-garcia@stats.ox.ac.uk *(R. Sanchez-Garcia),* carazo@cnb.csic.es *(J.M. Carazo)* @cossStock *(C.O.S. Sorzano),* @JM_Carazo *(J.M. Carazo)*
https://doi.org/10.1016/j.jmb.2022.167556
Edited by Michael Sternberg

Abstract

Computational approaches for predicting protein-protein interfaces are extremely useful for understanding and modelling the quaternary structure of protein assemblies. In particular, partner-specific binding site prediction methods allow delineating the specific residues that compose the interface of protein complexes. In recent years, new machine learning and other algorithmic approaches have been proposed to solve this problem. However, little effort has been made in finding better training datasets to improve the performance of these methods. With the aim of vindicating the importance of the training set compilation procedure, in this work we present BIPSPI+, a new version of our original server trained on carefully curated datasets that outperforms our original predictor. We show how prediction performance can be improved by selecting specific datasets that better describe particular types of protein interactions and interfaces (e.g. homo/hetero). In addition, our upgraded web server offers a new set of functionalities such as the sequence-structure prediction mode, hetero- or homo-complex specialization and the guided docking tool that allows to compute 3D quaternary structure poses using the predicted interfaces. BIPSPI+ is freely available at https://bipspi.cnb.csic.es.
© 2022 The Authors. Published by Elsevier Ltd. This is an open access article under the CC BY license (http://creativecommons.org/licenses/by/4.0/).

Introduction

Protein-protein interactions (PPIs) play a pivotal role in most biological processes and thus, understanding how PPIs occur is an important step towards elucidating how these processes take place in cells and organisms. Studying the biochemical underpinnings behind PPIs can be better approached from a structural perspective. Experimental techniques such as X-ray crystallography, nuclear magnetic resonance or cryo-electron microscopy are capable of solving the 3D structure of PPIs, in many cases, reaching atomic resolutions. However, these techniques are expensive, time-consuming, and they cannot keep pace with the amount of interactomic data that every year is generated. As a result, many computational approaches have been developed

0022-2836/© 2022 The Authors. Published by Elsevier Ltd.This is an open access article under the CC BY license (http://creativecommons.org/licenses/by/4.0/).

to complement experimental methods and provide PPIs details at different levels of granularity.

In recent years, many computational methods have been designed to characterize PPIs when different levels of molecular information are available. For instance, protein docking methods can predict the full 3D structure of the PPI conformation when structural data of the interacting participants are available.[1–5] When protein atomic models are not available, new deep learning methods have been highly successful predicting the tertiary structure of proteins.[6,7] However quaternary structure prediction is more challenging and although initial steps have been conducted in that direction, they are computationally demanding and still require from manual intervention.[8] As an alternative, lower granularity predictions can be computed fully automatically with less computational requirements. For instance, some methods can predict what protein regions or amino acids pairs might be involved in the interaction using sequence information.[9–12] Another family of approaches predicts protein binding sites using sequence or structural information.[13–18] One of such approaches is partner-specific binding site prediction.[19–22] Contrary to conventional binding site prediction (non-partner specific), which aims to predict all the residues of a given protein that participate in any interaction, partner-specific methods seek to identify those residues that are involved in a particular PPI. Since proteins tend to interact with many distinct partners[23] and the involved interfaces can be quite different, partner-specificity is a convenient feature when studying a particular PPI.

Partner-specific predictors were firstly proposed by Ahmad and Mizaguchi[19] and, since then, many more have been developed.[20,24–30] Most of these methods aim to predict pairs of interacting residues, each belonging to a different protein partner, using machine learning algorithms trained over datasets derived from atomic models of protein complexes. Although several algorithmic approaches have been proposed, little emphasis has been made on the dataset used for training and developing these approaches. Thus, most, and especially the recently published partner-specific predictors, have been limited to small datasets, mainly the different versions of the Protein-Protein Docking Benchmark.[16,19,20,25] Indeed, to the best of our knowledge, only the works of Meyer et al. and Townshend et al. tried to build datasets for this particular problem, yet their impact on performance was not analysed in detail.[24,27] More importantly, only a single strategy for dataset compilation was considered.

In this work, we present BIPSPI+, a new version of our partner-specific binding site predictor that illustrates how a carefully selected training dataset can severely improve machine learning-based methods performance. BIPSPI+, as the original version,[21] can be employed to predict the binding sites of two interacting proteins given either their sequences or their structure. The new version offers a novel mode that can be used in those cases in which only the structure of one of the partners is known, exhibiting better performance than the sequence-only version. Additionally, the new approach was trained independently to predict binding sites for hetero- and homo-dimer cases. Overall, BIPSPI+ outperforms the original version in all studied datasets irrespectively of the input type, being especially worth noting the improvements for homo-complexes predictions.

In addition to offering better performance, the BIPSPI+ web server has been upgraded to include a new guided docking option that employs PatchDock[2] on BIPSPI+ predictions used as restraints. As a result, BIPSPI+ can now provide both binding site prediction and atomic models for the PPIs. To our knowledge, our method and the Ahmad and Mizaguchi one are the only partner-specific predictors available through web servers, and only ours allows the users to directly perform guided docking from the predictions.

BIPSPI+ web app is publicly available at https://bipspi.cnb.csic.es and as a stand-alone tool at https://github.com/rsanchezgarc/BIPSPI.

Methods

BIPSPI is a machine learning-based partner-specific binding site predictor trained on structurally solved protein assemblies deposited in the PDB.[31,32] The training set consists of interacting and non-interacting residue pairs obtained from the 3D structure of protein complexes using a distance threshold criterion. BIPSPI+ is an upgraded version of the BIPSPI v1 web platform that implements three new major features: a new input mode (sequence & structure), complex-type stratification (homo-complex vs hetero-complex mode), and an optional step of guided Protein-Protein Docking (PP-docking). The following section briefly presents these new features, summarized in Figure 1. For a complete description of the method, we refer the reader to the Supplementary Material section 1.

Input: sequence-sequence, structure-structure and sequence-structure modes

BIPSPI v1 could be employed to predict the interacting residues of two protein structures or two sequences. BIPSPI+ has been redesigned to work also for cases in which only the structure of one of the interacting partners is known. This new input mode, which we have termed as the “sequence-structure mode”, employs sequence-only features to describe the sequence amino acids of the partner with no atomic model whereas residues of the other partner of the complex are described employing all features as in structure-structure mode. Consequently, the result page for this mode (Figure 1(j)) is a hybrid of the structure-structure (Figure 1(i)) and the sequence-sequence mode (Figure 1(h)) viewers, consisting of a 3D-

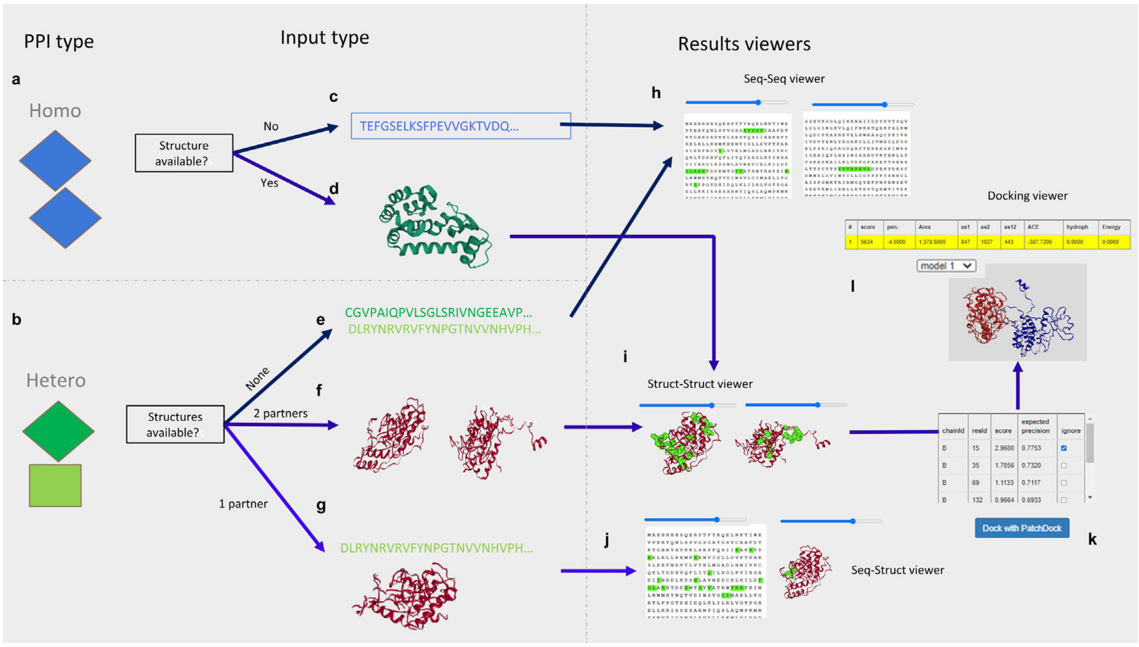

Figure 1. BIPSPI+ execution options. The protein complex to be predicted can be either a homo-complex (a) or a hetero-complex (b). Depending on the availability of atomic models, BIPSPI+ can be executed under different modes. The sequence-only mode is used if none of the structures is known (c and e), results being displayed in the Seq-Seq viewer (h). For the case of heterocomplexes in which only one of the structures is available (g), the sequence vs structure mode is used and the results are displayed in the Seq-Struct viewer (j). Finally, if the structure of the monomer is either known for homo-complexes (d) or the structure of the two interacting partners is known for hetero-complexes (f), the full structure mode is used instead. In this case, results are displayed in the Struct-Struct viewer (i), in which the structure of the two interacting partners, or two copies of the monomer, are shown. From this viewer, it is possible to execute guided docking, selecting the subset of residues to be employed as constraints (k). Docking results are displayed in the Docking viewer (l).

viewer for the partner with structure and a sequence panel for the partner with unknown structure.

Guided docking

BIPSPI+ web platform has been upgraded to perform an optional step of guided protein–protein docking using PatchDock[2]. Thus, after computing binding site predictions for protein structures, the user can select, using a threshold slider and a table with checkboxes, which are the residues that will be used as restraints for guided docking. Then, PatchDock is executed with default parameters. After execution, a results page allows for interactive visualization of the highest-score predicted poses as well as downloading the atomic models and raw files generated during the docking step (see Figure 1(k–l)).

Homo-complexes and hetero-complexes datasets

BIPSPI+ was trained on two different datasets, one dataset consisting only of hetero-complexes (HEMt) and another dataset containing only of homo-complexes (HODt), both being more than one order of magnitude larger than the original BIPSPI v1 training dataset. Supplementary Material section 1.1, 1.2 and 2.5 describe these and other studied datasets. Similarly, the performance of our method was also assessed against two testing datasets representing the two possible types of complexes. Particularly, we employed the Protein-Protein Docking Benchmark v5 (Bv5), composed of 230 hetero-complexes and a custom evaluation benchmark, which we termed HOe (Homo-complexes evaluation), composed of 223 homodimers. Since the HOe dataset contains only complexes in bound state, performance could be overestimated when using structural features, but since comparison against BIPSPI v1 was also carried out on this dataset, improvement conclusions could be considered robust. Moreover, it is important to notice that the model trained on sequence-based features only is not affected by this problem and thus, its performance estimation is reliable.

Results

BIPSPI+ usage

BIPSPI+ can be employed to obtain partner-specific binding site predictions for protein

complexes. First, the user needs to select the oligomerization state of the PPI as homo-complex or hetero-complex (Figure 1(a and b), Supplementary Figure 1). Then, the user needs to provide either a sequence or an atomic model for the monomer in the homo-complex case (Figure 1 (c and d), Supplementary Figure 1) or for both interacting partner in the hetero-complex case (Figure 1(e–g), Supplementary Figure 1). The 5 different oligomerization types and input types combinations (homo-sequence, homo-structure, hetero-sequence-sequence, hetero-sequence-structure, and hetero-structure-structure) are processed by 5 different models trained on the same types of data as the input.

After calculations, binding site predictions are displayed in one of the three different types of viewers depending on the input type (Figure 1(h–j)). In each of the viewers, the predicted interface residues with a score greater than the selected threshold are highlighted on the input sequences or structures (Figure 1(h–j)). Thresholds can be changed using a slider that displays the expected precision for the predictions given the current value of the threshold.[21] For easiness of visualization, homo-complexes results are displayed using the same graphical interface in which two exact copies of the input monomer and the predictions are displayed as independent partners.

Finally, for the case of homo-complexes with structure or heterocomplexes with structures for both partners, it is possible to launch a guided docking job using as restraints the binding site residues predicted by BIPSPI at different thresholds (Figure 1(k), Supplementary Figure 3) or a custom subset of them, by checking the ignore checkbox of some of the residues with scores above the selected threshold. Once the residues to be used as restraints are selected, the docking calculations are carried out, and the highest score docking results are displayed in the Docking viewer, in which the user can visually inspect or download the proposed models (Figure 1(l), Supplementary Figure 4).

Better training data enhances performance

Since the performance of machine learning methods is severely influenced by the amounts and quality of the available data, it seems reasonable to believe that partner-specific binding site prediction can also benefit from this strategy. However, obtaining PPI complexes for a training dataset is challenging. First of all, the total number of solved complexes represents only a small fraction of the interactome. For instance, in humans, less than 10% of the binary interactions have been structurally solved.[33] Second, there are very few examples for which we know the structure of both the bound and unbound structure, most of them contained in the Bv5. While the former problem cannot be directly tackled until more experimental data is obtained, the importance of the latter could be not so critical for methods like BIPSPI, which integrates both structural and sequence-based features.[21] Consequently, for the second version of our method, we constructed larger training datasets that, for the majority of the complexes, do not contain the unbound version of the interacting partners. Despite this limitation, as it is shown in Figure 2 blue and red curves and described in Supplementary Material section 2.2, the inclusion of more bound complexes in the dataset was able to significantly improve results over BIPSPI v1. Thus, for the Bv5 using structural information, we measured a mean ROC AUC for residue-residue pairs interactions of 0.927 (median ROC AUC of 0.951) and a ROC AUC of 0.848 for binding site prediction. The new version increased both metrics with respect to our original method (0.905 and 0.823, respectively), achieving state-of-the-art performance (see Supplementary Table 5). For a detailed description of the evaluation approach see Supplementary Material section 1.3.

In addition to the size of the dataset, we also studied some other parameters that affect the quality of the data. For instance, we showed (see Supplementary Material section 2.3 and Supplementary Figure 8) that the inclusion of multimers, despite multiple caveats such as automatic receptor/ligand definition, enhances the performance of the predictions for predicting both dimers and multimers. Other studied parameters are discussed in Supplementary Material section 2.4–5.

Another important challenge when increasing the size of the dataset is the fact that most of the protein complexes contained in the PDB correspond to homo-complexes while the standard testing dataset, the Bv5, only contains hetero-complexes. Although the physics behind homo-complexes is the same that in hetero-complexes, statistical analysis show that physicochemical features of hetero- and homo-complex interfaces differ in many aspects such as contact preference, composition or hydrophobicity.[11] Consequently, some difference in performance could be expected depending on the oligomerization state. However, when we first studied the impact of the oligomerization type, the observed difference in performance for BIPSPI v1 was beyond our expectations, with a difference in MCC of 0.15 (see Supplementary Table 1) and important precision drops in the high-threshold region (high precision and low recall), the most interesting one for experimental validation (see Figure 2 left vs right panel). For BIPSPI+ we included homo-complexes in the training dataset using two strategies: first, training using two different datasets, one for each complex type (HEMt and HODt) and second, combining HEMt and HODt into one single training dataset (HEHODt). Supplementary Table 1 and Supplementary Figures 5–6 show that the first strategy offers comparable or bet-

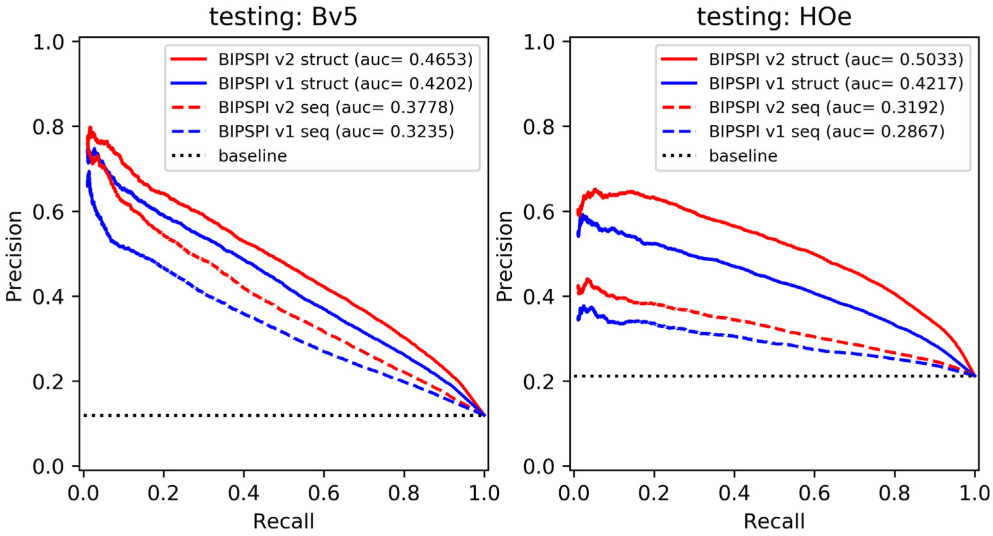

Figure 2. BIPSPI v1 and BIPSPI+ performance comparison. BIPSPI v1(blue) and BIPSPI+ (red) precision-recall curves evaluated on hetero-complexes (Bv5, left) and homo-complexes (HOe, right) using as input either the sequences of the interacting partners (dashed lines) or their structures (solid lines).

ter results for all the analysed benchmarks, suggesting that the information extracted by our method from one oligomerization state is of little value, if not harmful for the other type. Consequently, in BIPSPI+, the users are required to select the oligomerization state and the two trained models are applied accordingly.

Sequence-structure mode

Partner-specific binding site predictors require sequence information and/or structural data for both interacting partners as input. So far, existing methods only consider the symmetric cases in which either the two structures or the two sequences are present. However, it is quite common that only the structure of one of the interacting protein partners is available (e.g., modelling low-resolution regions in cryo-EM maps, synthetic designs, etc.). Given the fact that structural data allows for better prediction performance, the common alternative of approaching those cases as if only the sequences were available is not compelling. In order to overcome this shortcoming, we have developed for BIPSPI+ the sequence-structure (seq-struct) mode.

We evaluated the performance of the seq-struct mode using as evaluation benchmark Bv5 and we studied how the new mode performed on both the input provided as sequence and the one provided as structure (see Supplementary Material Table 3). As expected, the quality of the predictions for the seq-struct mode, with an MCC value of 0.331, lies between the performance of the model that only employs sequences (MCC of 0.311) and the model that employs structures from the two partners (MCC of 0.403). For more details, see Supplementary Results section 2.6).

Figure 3(a) illustrates the benefits of this new execution mode on 2OZA, one of the protein complexes of the Bv5 for which we computed the predictions providing as input either the two sequences of chains A and B (X in unbound) or the sequence of the chain B and the structure of chain A. From direct inspection, it could be noticed that, when the structure of the studied protein partner is employed, the quality of the predictions largely improves. Thus, for chain A, the accuracy at threshold 0.5 is 0.60 when only the sequences are employed. However, when the structure of chain A is employed, accuracy gets boosted to 0.89.

Guided docking

While binding site predictions are invaluable sources of hypothesis for multiple experimental scenarios (e.g. mutagenesis experiments), when possible, 3D atomic models of the protein complexes offer a much richer description of PPIs. BIPSPI predictions have been successfully used as guided PP-docking constraints,[34–36] improving the quality of 3D models. However, guided docking pipelines tend to be complicated, involving several computational steps and requiring a good understanding of the different tools.[37]

With the aim of facilitating the generation of 3D models, we have included a simple guided docking pipeline based on PatchDock, a rigid body docking algorithm based on geometric hashing. Our pipeline simply requires the users to select a threshold for the binding site predictions so that the selected residues will be provided to PatchDock as binding site restraints, limiting the

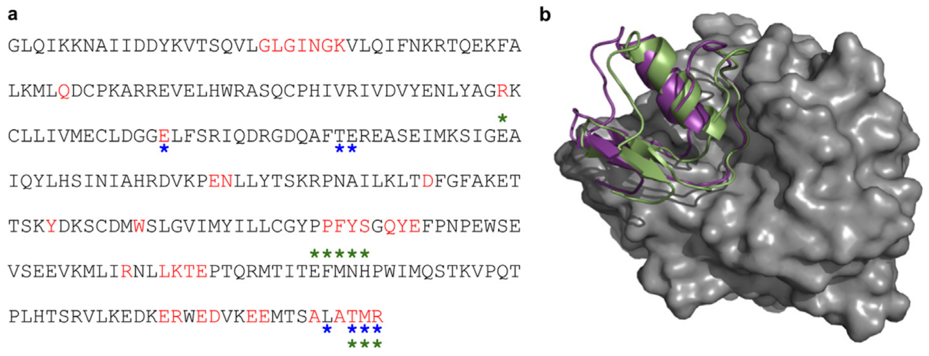

Figure 3. BIPSPI+ use cases. a) Sequence-structure mode improvement example. Sequence-only predictions (blue asterisks) and sequence-structure (green asterisks) predictions on structurally solved residues for Bv5 unbound complex 2OZA chain X (B in bound). Residues in contact with chain A are marked in red. Predictions above 0.5 score are marked with stars in blue when using only sequence information for both chain A and X and green when the structure of chain X is employed alongside the sequence of chain A. b) PatchDock docking model for the Subtilisin Carlsberg-OMTKY3 Complex (PDB code 1YU6, chains A and C respectively) obtained from BIPSPI+ web server. The crystallographic structures are depicted in grey for Chain A and green for Chain C whereas the docked model is depicted in purple.

search space to those poses compatible with the selected restraints. We acknowledge that our pipeline is simple and, consequently, better results could be easily obtained using more complicated pipelines and/or algorithms. However, our intention was to develop a user-friendly solution to retrieve fast initial structural models that could help the users to understand the binding site predictions.

Despite our pipeline's simplicity, accurate models can be obtained in many cases, providing conformational changes are not severe. Thus, Figure 3(b), illustrates an example of a 3D model for the protein complex Subtilisin Carlsberg-OMTKY3 Complex (PDB code 1YU6,[38] chains A and C respectively) computed using the BIPSPI+ web application. From direct inspection of the figure, it can be noticed that binding site predictions for this complex were of high quality, with an important part of the binding site accurately predicted. These accurate predictions ultimately allowed the docking algorithm to propose a high-ranked solution (3rd) of medium quality (iRMS 1.5 Å, DockQ = 0.64[39]) in a totally automatic fashion.

Conclusion

Partner-specific binding site predictions have proven to be a useful resource in several contexts, especially for guiding protein-protein docking. Consequently, new approaches have been developed in recent times. However, while most of the new methods make special emphasis on algorithmic aspects, the crucial impact that datasets have on performance was not deeply studied. With the aim of addressing this issue, we developed BIPSPI+, an improved version of our original method, trained on carefully selected datasets of complexes, that exhibit enhanced performance. While BIPSPI+ outperforms BIPSPI v1 in all the evaluated benchmarks, it is especially for the case of homo-complexes when performance is largely boosted. In addition to enhanced performance, the BIPSPI+ web application, freely available at https://bipspi.cnb.csic.es, has been updated to easily deal with homo-complexes and also for hetero-complexes in which only one of the interacting partners is structurally solved. Finally, the BIPSPI+ web application offers an optional step of guided protein-protein docking that can provide users with complete structural models of the protein interaction.

Data availability

Precomputed models and results are available at https://zenodo.org/record/5574182#.YYhiOrvLfmH. BIPSPI+ web server is available at https://bipspi.cnb.csic.es/ the predictor code is available at https://github.com/rsanchezgarc/BIPSPI.

CRediT authorship contribution statement

R. Sanchez-Garcia: Conceptualization, Methodology, Software, Writing – original draft. **J. R. Macias:** Software, Validation. **C.O.S. Sorzano:** Validation, Writing – review & editing. **J.M. Carazo:** Funding acquisition, Writing – review & editing. **J. Segura:** Conceptualization, Data curation, Supervision, Writing – review & editing.

Acknowledgments

We acknowledge the Centro de Supercomputación de Galicia (CESGA) for their computational resources that were kindly provided.

Funding

Grant PID2019-104757RB-I00 funded by MCIN/AEI/10.13039/501100011033/ and "ERDF A way of making Europe", by the "European Union"; "Comunidad Autónoma de Madrid" through Grant: S2017/BMD-3817; HighResCells (ERC - 2018 - SyG, Proposal: 810057); SEV-2017-0712 funded by MCIN/AEI/10.13039/501100011033; EOSC Life (INFRAEOSC-04-2018, Proposal: 824087); Grant DBI-1832184 by the National Science Foundation; Grant DE-SC0019749 by the US Department of Energy; and Grant R01GM133198 (Principal Investigator: Stephen K. Burley) by the National Cancer Institute, National Institute of Allergy and Infectious Diseases, and National Institute of General Medical Sciences of the National Institutes of Health.

Declaration of Competing Interest

The authors declare that they have no known competing financial interests or personal relationships that could have appeared to influence the work reported in this paper.

Appendix A. Supplementary data

Supplementary data to this article can be found online at https://doi.org/10.1016/j.jmb.2022.167556.

Received 27 November 2021;
Accepted 16 March 2022;
Available online 21 March 2022

Keywords:
protein interactions;
binding site;
web server;
machine learning

References

1. Dominguez, C., Boelens, R., Bonvin, A.M.J.J., (2003). HADDOCK: A protein-protein docking approach based on biochemical or biophysical information. *J Am Chem Soc* **125**, 1731–1737.
2. Schneidman-Duhovny, D., Inbar, Y., Nussinov, R., Wolfson, H.J., (2005). PatchDock and SymmDock: servers for rigid and symmetric docking. *Nucleic Acids Res* **33**, W363–W367.
3. Cheng, T.M.K., Blundell, T.L., Fernandez-Recio, J., (2007). pyDock: electrostatics and desolvation for effective scoring of rigid-body protein-protein docking. *Proteins* **68**, 503–515.
4. Ghoorah, A.W., Devignes, M.D., Smaïl-Tabbone, M., Ritchie, D.W., (2013). Protein docking using case-based reasoning. *Proteins Struct Funct Bioinf* **81**, 2150–2158.
5. Zhang, Q. et al, (2016). Recent advances in protein-protein docking. *Curr Drug Targets* **17**, 1586–1594.
6. Baek, M. et al, (2021). Accurate prediction of protein structures and interactions using a three-track neural network. *Science* **373**, 871–876.
7. Jumper, J. et al, (2021). Highly accurate protein structure prediction with AlphaFold. *Nature* **596**, 583–589.
8. Humphreys, I.R. et al, (2021). Computed structures of core eukaryotic protein complexes. *Science*. https://doi.org/10.1126/science.abm4805.
9. Segura, J. et al, (2016). 3DIANA: 3D domain interaction analysis: A toolbox for quaternary structure modeling. *Biophys J* **110**, 766–775.
10. Segura, J., Sorzano, C.O.S.S., Cuenca-Alba, J., Aloy, P., Carazo, J.M., (2015). Using neighborhood cohesiveness to infer interactions between protein domains. *Bioinformatics* **31**, 2545–2552.
11. Ofran, Y., Rost, B., (2003). Predicted protein-protein interaction sites from local sequence information. *FEBS Lett* **544**, 236–239.
12. Zhang, J., Kurgan, L., (2019). SCRIBER: Accurate and partner type-specific prediction of protein-binding residues from proteins sequences. *Bioinformatics*. Oxford Academic.
13. Segura, J., Jones, P.F., Fernandez-Fuentes, N., (2012). A holistic in silico approach to predict functional sites in protein structures. *Bioinformatics* **28**, 1845–1850.
14. Segura, J., Jones, P.F., Fernandez-Fuentes, N., (2011). Improving the prediction of protein binding sites by combining heterogeneous data and Voronoi diagrams. *BMC Bioinf* **12**, 352.
15. Porollo, A., Meller, J., (2007). Prediction-based fingerprints of protein-protein interactions. *In: Proteins: Structure, Function and Genetics*, pp. 630–645.
16. Savojardo, C., Fariselli, P., Martelli, P.L., Casadio, R., (2017). ISPRED4: Interaction sites PREDiction in protein structures with a refining grammar model. *Bioinformatics* **33**, 1656–1663.
17. Šikić, M., Tomić, S., Vlahoviček, K., (2009). Prediction of protein-protein interaction sites in sequences and 3D structures by random forests. *PLoS Comput Biol* **5**, e1000278.
18. Andreani, J., Quignot, C., Guerois, R., (2020). Structural prediction of protein interactions and docking using conservation and coevolution. *Wiley Interdiscipl Rev: Comput Mol Sci* **10**, e1470.
19. Ahmad, S., Mizuguchi, K., (2011). Partner-aware prediction of interacting residues in protein-protein complexes from sequence data. *PLoS ONE* **6**, e29104.
20. Minhas, F.ul A.A., Geiss, B.J., Ben-Hur, A., (2014). PAIRpred: partner-specific prediction of interacting residues from sequence and structure. *Proteins* **82**, 1142–1155.

21. Sanchez-Garcia, R., Sorzano, C.O.S., Carazo, J.M., Segura, J., (2019). BIPSPI: A method for the prediction of partner-specific protein-protein interfaces. *Bioinformatics* **35**, 470–477.
22. Xue, L.C., Dobbs, D., Bonvin, A.M.J.J., Honavar, V., (2015). Computational prediction of protein interfaces: A review of data driven methods. *FEBS Lett* **589**, 3516–3526.
23. Grigoriev, A., (2003). On the number of protein-protein interactions in the yeast proteome. *Nucleic Acids Res* **31**, 4157–4161.
24. Meyer, M.J. et al, (2018). Interactome INSIDER: a structural interactome browser for genomic studies. *Nat Methods* **15**, 107–114.
25. Fout, A., Shariat, B., Byrd, J., Ben-Hur, A., (2017). Protein interface prediction using graph convolutional networks. *Adv Neural Inform Process Syst* **30**, 6512–6521.
26. Chen, T., Guestrin, C., (2016). XGBoost: A Scalable Tree Boosting System. *In: Proceedings of the 22nd ACM SIGKDD International Conference on Knowledge Discovery and Data Mining - KDD '16*, pp. 785–794. https://doi.org/10.1145/2939672.2939785.
27. Townshend, R.J.L., Bedi, R., Suriana, P.A., Dror, R.O., (2019). End-to-end learning on 3D protein structure for interface prediction. *Advances in Neural Information Processing Systems*. Neural Information Processing Systems Foundation.
28. Dai, B., Bailey-Kellogg, C., (2021). Protein interaction interface region prediction by geometric deep learning. *Bioinformatics* **37**, 2580–2588.
29. Xue, L.C., Dobbs, D., Honavar, V., (2011). HomPPI: a class of sequence homology based protein-protein interface prediction methods. *BMC Bioinf* **12**, 244.
30. Yan, Y., Huang, S.Y., (2021). Accurate prediction of inter-protein residue–residue contacts for homo-oligomeric protein complexes. *Briefings Bioinf* **22**, 1–13.
31. Berman, H.M. et al, (2000). The protein data bank. *Nucleic Acids Res* **28**, 235–242.
32. Burley, S.K. et al, (2021). RCSB Protein Data Bank: Powerful new tools for exploring 3D structures of biological macromolecules for basic and applied research and education in fundamental biology, biomedicine, biotechnology, bioengineering and energy sciences. *Nucleic Acids Res* **49**, D437–D451.
33. Mosca, R., Céol, A., Aloy, P., (2012). Interactome3D: adding structural details to protein networks. *Nat Methods* **10**, 47–53.
34. Pozzati, G., Kundrotas, P. & Elofsson, A. Improved protein docking by predicted interface residues. *bioRxiv* 2021.08.25.457642 (2021) 10.1101/2021.08.25.457642.
35. Id, A.L.A., Barnes Id, A.B., Martin Id, A.T., Wang, L., Koid, D.C., (2021). Variation in Leishmania chemokine suppression driven by diversification of the GP63 virulence factor. *PLOS Neglected Tropical Dis* **15**, e0009224.
36. Sharma, A. et al, (2021). Ppar-responsive elements enriched with alu repeats may contribute to distinctive pparγ–dnmt1 interactions in the genome. *Cancers* **13**, 3993.
37. Segura, J., Marín-López, M.A., Jones, P.F., Oliva, B., Fernandez-Fuentes, N., (2015). VORFFIP-Driven Dock: V-D2OCK, a fast and accurate protein docking strategy. *PLoS ONE* **10**, e0118107.
38. Maynes, J.T., Cherney, M.M., Qasim, M.A., Laskowski, M., James, M.N.G., (2005). Structure of the subtilisin Carlsberg-OMTKY3 complex reveals two different ovomucoid conformations. *Acta Crystallogr. Section D, Biol Crystallogr* **61**, 580–588.
39. Basu, S., Wallner, B., (2016). DockQ: A quality measure for protein-protein docking models. *PLoS ONE* **11**, e0161879
