# Supplementary material for: PDBx/mmCIF Ecosystem: Foundational Semantic Tools for Structural Biology
Source: J Mol Biol. Author manuscript; Available in PMC 2023 Jun 26. (PMC10292674; doi:10.1016/j.jmb.2022.167599)
Supplement: Article [file NIHMS1907597-supplement-Article.zip › Editorial-Board_2022_Journal-of-Molecular-Biology.pdf]

jmb
Journal of Molecular Biology

Editor-in-Chief

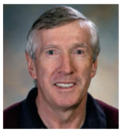

Peter Wright
Department of Integrative Structural and Computational Biology, The Scripps Research Institute, La Jolla, CA, U.S.A.
jmb@scripps.edu

Senior Scientific Editor

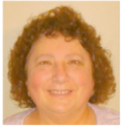

Denise Wells
Cambridge, MA, USA
d.wells@elsevier.com

Senior Managing Editor

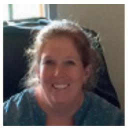

Jasmin Bakker
Amsterdam, Netherlands
ja.bakker@elsevier.com

Social Media Editor

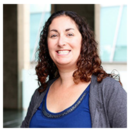

Rebecca Berlow
University of North Carolina School of Medicine, Department of Biochemistry and Biophysics, Chapel Hill, NC, USA

Associate Editors

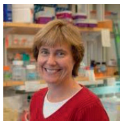

Anna Marie Pyle,
Yale University, New Haven, CT, U.S.A.

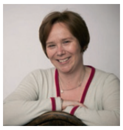

Sheena E. Radford
University of Leeds, Department of Biochemistry and Molecular Biology, Leeds, U.K.

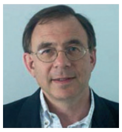

Michael J.E. Sternberg
Division of Molecular Biosciences, Imperial College London, London, U.K.

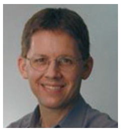

Michael F. Summers
Department of Chemistry and Biochemistry, University of Maryland, Baltimore, MD, U.S.A.

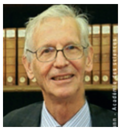

Moshe Yaniv
Institut Pasteur, Dept. of Developmental & Stem Cell Biology, Paris, France

Board of Editors

James M. Berger, Johns Hopkins University School of Medicine, Baltimore, Maryland, USA
Philip C. Bevilacqua, Pennsylvania State University, University Park, PA, USA
Johannes Buchner, Technische Universität München, Munich, Germany
Rita Casadio, Biocomputing Group, University of Bologna and IBIOM-CNR, Bologna, Italy
Eric Cascales, CNRS - Aix-Marseille Université, Marseille, France
Bil Clemons, California Institute of Technology, Pasadena, CA, USA
Yamini Dalal, Center for Cancer Research, Frederick, MD, USA
Eric Freed, NCI-Frederick, Frederick, MD, USA
Monika Fuxreiter, University of Debrecen, Hungary
Ruben L. Gonzalez, Jr., Columbia University, New York, NY, USA
Max Gottesman, Columbia University Medical Center, New York, NY, USA
Patrick Griffin, The Scripps Research Institute, Jupiter, FL, USA
Mitchell Guss, University of Sydney, Sydney, Australia
Barry Honig, Columbia University Medical Center, New York, NY, USA
Anne Houdusse, Institute Curie, Paris, France
Gerhard Hummer, Max Planck Institut für Biophysik, Frankfurt am Main, Germany
Urs Jenal, Universität Basel, Basel, Switzerland
Charalampos Kalodimos, St. Jude Children's Research Hospital, Memphis, TN, USA
Achillefs Kapanidis, University of Oxford, Oxford, UK
Amy Keating, Massachusetts Institute of Technology, Cambridge, MA, USA
Sepideh Khorasanizadeh, University of Oxford, UK
Shohei Koide, University of Chicago, Chicago, IL, USA
Richard W. Kriwacki, St. Jude Children's Research Hospital, Memphis, TN, USA
Gabriel Lander, The Scripps Research Institute, La Jolla, CA, USA
Edward Lemke, Johannes Gutenberg University, Institute of Molecular Biology, Mainz, Germany
Zhi-Jie (James) Liu, ShanghaiTech University, Pudong, Shanghai, China
Karolin Luger, Colorado State University, Fort Collins, CO, USA
Sascha Martens, University of Vienna, Austria
David H. Mathews, University of Rochester Medical Center, Rochester, NY, USA
Anthony Maxwell, John Innes Centre, Norwich, UK
Daniel L. Minor, University of California San Francisco, San Francisco, California, USA
Daniel Otzen, Aarhus University, Aarhus, Denmark
Anna Panchenko, Queen's University Department of Pathology and Molecular Medicine, Kingston, Ontario, Canada
Heather Pinkett, Northwestern University, Evanston, Illinois, USA
Bert Poolman, University of Groningen, Groningen, Netherlands
Owen Pornillos, University of Virginia School of Medicine, Charlottesville, VA, USA
Raul Rabadan, Columbia University Vagelos College of Physicians and Surgeons, New York, NY, USA
Lutz Schmitt, Heinrich Heine University Düsseldorf, Germany
Georg E. Schulz, Albert-Ludwigs-Universitat, Freiburg im Breisgau, Germany
James R. Sellers, National Health, Lung and Blood Institute, Bethesda, MA, USA
Louise Serpell, University of Sussex, Brighton, UK
Konstantin Severinov, Rutgers University, Piscataway, NJ, USA
Feng Shao, National Institute of Biological Sciences, Beijing (NIBS), Beijing, China
Arne Skerra, Technische Universität München , Freising-Weihenstephan, Germany
Yigong Shi, Tsinghua University, Beijing, China
Ichio Shimada, University of Tokyo, Tokyo, Japan
Sachdev Sidhu, University of Toronto, Toronto, Ontario, Canada
Titia Sixma, Nederlands Kanker Instituut (NKI), Amsterdam, Netherlands
Thomas J. Smith, Donald Danforth Plant Science Center, St. Louis, MO, USA
Igor Stagljar, University of Toronto, Toronto, Ontario, Canada
Dylan J. Taatjes, University of Colorado, Boulder, Colorado, USA
Sarah Teichmann, EMBL-European Bioinformatics Institute & Wellcome Trust Sanger Institute, Hinxton, Cambridge, U.K.
Yanli Wang, Institute of Biophysics Chinese Academy of Sciences, Beijing, China
Ronald Wetzel, University of Pittsburgh School of Medicine, Pittsburgh, PA, USA
Sarah Woodson, Johns Hopkins University, Baltimore, MD, USA
Nieng Yan, Princeton University, Princeton, NJ, USA
Kai Zhang, University of Illinois at Urbana-Champaign, Urbana, IL, USA
Mingjie Zhang, Hong Kong University of Science & Technology, Kowloon, Hong Kong

Founding Editor

Sir John Kendrew

Editorial Office

The full and complete Guide for Authors can be found at :

All inquiries to : jmb@elsevier.com
http://www.elsevier.com/locate/jmb.

Cover Illustration: Artwork by Rachel Davidowitz, Freelance Scientific Illustrator.
